# Supplementary material for: Genetics of trans-regulatory variation in gene expression
Source: eLife. 2018 Jul 17;7:e35471. doi: 10.7554/eLife.35471 (PMC6072440; doi:10.7554/eLife.35471)
Supplement: Supplementary file 1. — Sums of squares, degrees of freedom and F-values were computed using Type II analysis of variance as implemented in the R car package. (1) log2(TPM). [file elife-35471-supp1.docx]

**Table S1 – Multiple regression of heritability on various gene features.**

| Feature | Regression slope (change in heritability in units of percent per one unit of change in the given predictor) | Sums of squares | Degrees of freedom | F-value | p-value |
| --- | --- | --- | --- | --- | --- |
| Total expression variance | 7.6 | 5.9 | 1 | 264 | < 2.2e-16 |
| Expression level^1^ | 2.1 | 2.5 | 1 | 111 | < 2.2e-16 |
| Essential (yes/no) | -8.2 | 2.2 | 1 | 98 | < 2.2e-16 |
| dN/dS | -23 | 0.5 | 1 | 20 | 6e-6 |
| Number of protein-protein interactions | -0.01 | 0.2 | 1 | 8 | 0.005 |
| Number of genetic interactions | -0.01 | 0.3 | 1 | 15 | 1e-4 |
| Transcription factor (yes/no) | 0.4 | 0.001 | 1 | 0.06 | 0.8 |
| Human homolog (yes/no) | -0.6 | 0.02 | 1 | 1.1 | 0.3 |
| residuals | N/A | 61.5 | 2739 | N/A | N/A |
