# Supplementary material for: Genetics of trans-regulatory variation in gene expression
Source: eLife. 2018 Jul 17;7:e35471. doi: 10.7554/eLife.35471 (PMC6072440; doi:10.7554/eLife.35471)
Supplement: Supplementary file 2. — (1) Shown is the less significant p-value from the two ASE datasets. (2) The LOD score at the gene position itself irrespective of whether this eQTL is significant. (3) Positive values indicate higher expression in RM compared to BY. (4) These genes have strong eQTLs close to the gene, but with a confidence interval that just excludes the gene. The may be influenced by cis acting local eQTLs where the causal variant is located further away from the gene than captured by our definition of upstream regulatory regions as 1000 base pairs upstream of the start codon. [file elife-35471-supp2.docx]

**Table S2 – Genes with strong (more than 2-fold) and significant ASE in both datasets but no local eQTL**

| Gene | Local eQTL LOD^2^ | Local eQTL log2(fold change) | ASE p-value^1^ | ASE log2(fold change) ^3^ |
| --- | --- | --- | --- | --- |
| *OPT2*^4^ | 87 | 2.35 | 3e-13 | 2.8 |
| *CIN5* | 0.8 | 0.08 | 3e-14 | -1.8 |
| *UIP3*^4^ | 286 | -1.65 | 1e-14 | -1.4 |
| YAR028W^4^ | 300 | -2.52 | 1e-5 | -1.3 |
