# Supplementary material for: Genetics of trans-regulatory variation in gene expression
Source: eLife. 2018 Jul 17;7:e35471. doi: 10.7554/eLife.35471 (PMC6072440; doi:10.7554/eLife.35471)
Supplement: Supplementary file 3. — (1) Positive values indicate higher expression in RM compared to BY. (2) Shown is the more significant p-value from the two ASE datasets. (3) The nominally significant p-values in this column do not pass Bonferroni cutoff for significance. Therefore, ASE at these genes was not identified as significant. [file elife-35471-supp3.docx]

**Table S3 – Genes with a local eQTL but no ASE in spite of ≥80% power to detect ASE**

| Gene | Local eQTL LOD | Local eQTL log2(fold change) ^1^ | ASE p-value^2,3^ | ASE log2(fold change) ^1^ |
| --- | --- | --- | --- | --- |
| *TIF1* | 405 | -0.87 | 0.003 | -0.02 |
| *CBF1* | 349 | -1.36 | 0.8 | 1e-5 |
| *VPS63* | 200 | -4.4 | 0.09 | -0.36 |
| *UBA1* | 128 | -0.34 | 0.005 | -0.01 |
| *TPO4* | 108 | 0.97 | 9e-5 | 0.08 |
