# Supplementary material for: Genetics of trans-regulatory variation in gene expression
Source: eLife. 2018 Jul 17;7:e35471. doi: 10.7554/eLife.35471 (PMC6072440; doi:10.7554/eLife.35471)
Supplement: Supplementary file 4. — (1) Positive values indicate higher expression in RM compared to BY. (2) Shown is the less significant p-value from the two ASE datasets. (3) The table shows only genes where both ASE datasets agreed in the direction of effect. Shown is the average effect. [file elife-35471-supp4.docx]

**Table S4 – Genes with a local eQTL and significant ASE, and discordant direction of effect**

| Gene | Local eQTL LOD | Local eQTL log2(fold change)^1^ | ASE p-value^2^ | ASE log2(fold change)^1,3^ |
| --- | --- | --- | --- | --- |
| *TDH3* | 88 | -0.33 | 4e-239 | 0.37 |
| *YTA12* | 5.1 | -0.06 | 1e-6 | 0.25 |
| *DBP5* | 3.6 | -0.03 | 3e-6 | 0.18 |
