# Supplementary material for: Genetics of trans-regulatory variation in gene expression
Source: eLife. 2018 Jul 17;7:e35471. doi: 10.7554/eLife.35471 (PMC6072440; doi:10.7554/eLife.35471)

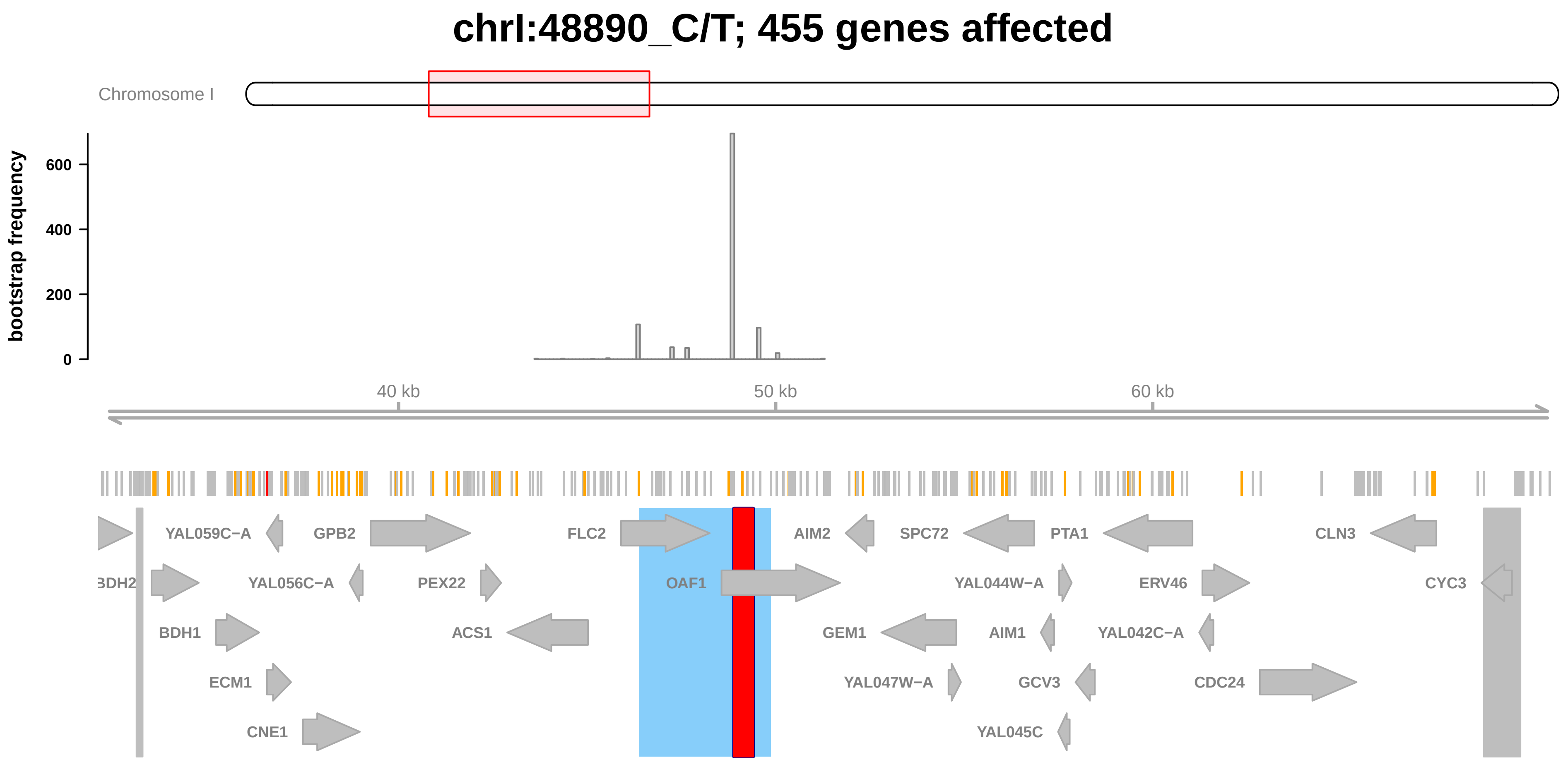

chrI:48890_C/T; 455 genes affected
Chromosome I
bootstrap frequency
0
200
400
600
40 kb
50 kb
60 kb
YAL059C−A
GPB2
FLC2
AIM2
SPC72
PTA1
CLN3
BDH2
YAL056C−A
PEX22
OAF1
YAL044W−A
ERV46
CYC3
BDH1
ACS1
GEM1
AIM1
YAL042C−A
ECM1
YAL047W−A
GCV3
CDC24
CNE1
YAL045C

chrI:109735_A/G; 162 genes affected

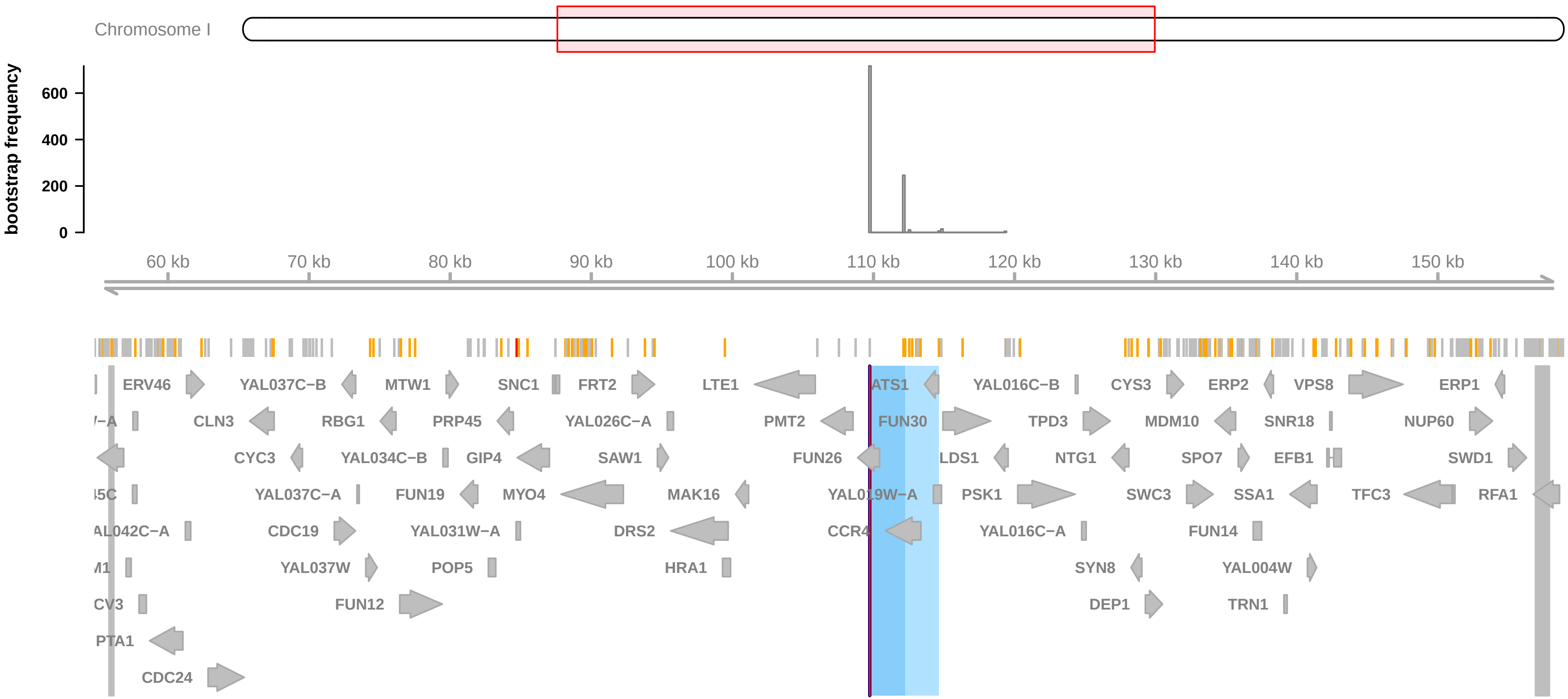

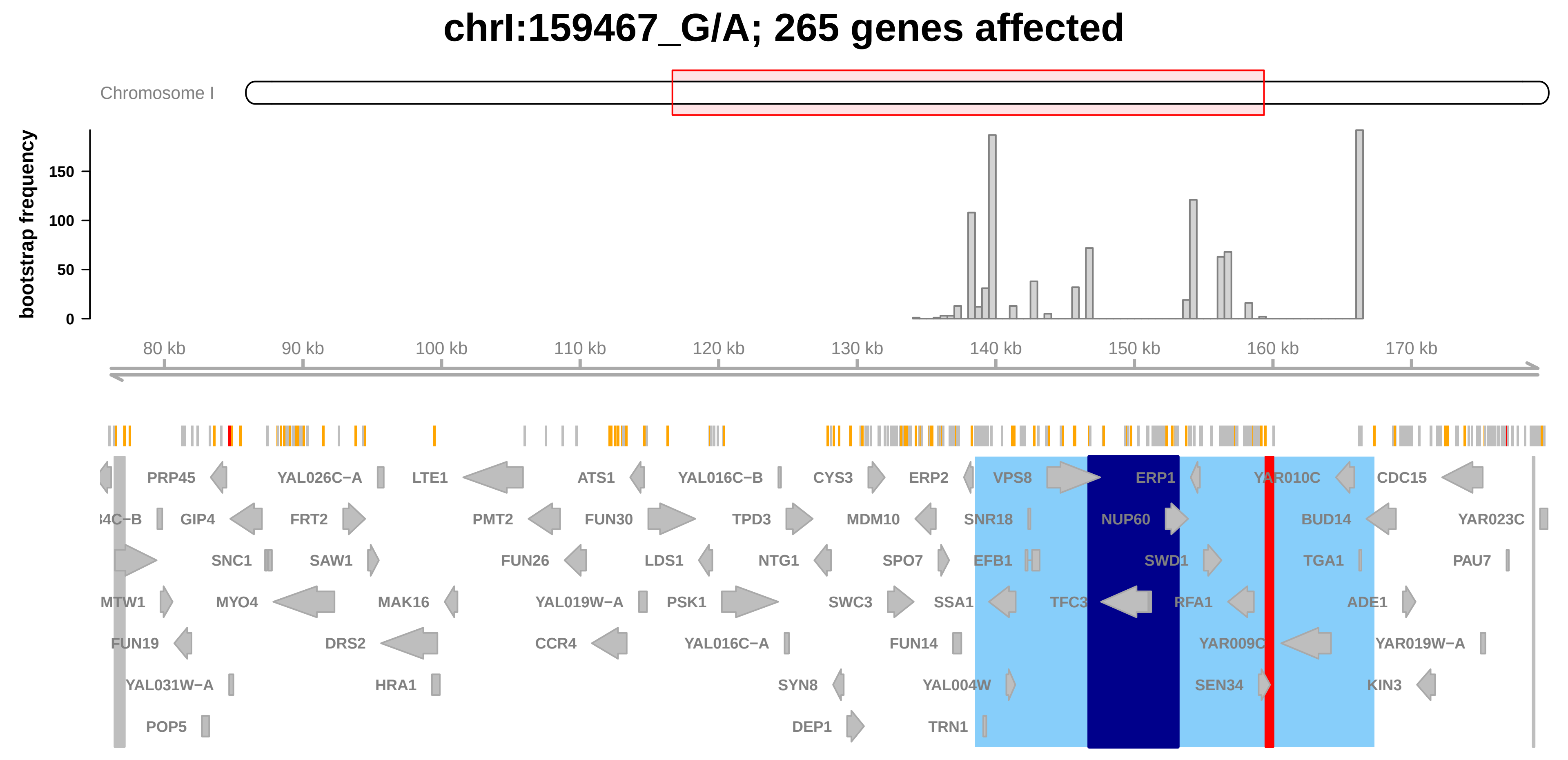
chrI:159467_G/A; 265 genes affected
Chromosome I
bootstrap frequency
0
50
100
150
80 kb
90 kb
100 kb
110 kb
120 kb
130 kb
140 kb
150 kb
160 kb
170 kb
PRP45
YAL026C−A
LTE1
ATS1
YAL016C−B
CYS3
ERP2
VPS8
ERP1
YAR010C
CDC15
GIP4
FRT2
PMT2
FUN30
TPD3
MDM10
SNR18
NUP60
BUD14
YAR023C
SNC1
SAW1
FUN26
LDS1
NTG1
SPO7
EFB1
SWD1
TGA1
PAU7
MTW1
MYO4
MAK16
YAL019W−A
PSK1
SWC3
SSA1
TFC3
RFA1
ADE1
FUN19
DRS2
CCR4
YAL016C−A
FUN14
YAR009C
YAR019W−A
YAL031W−A
HRA1
SYN8
YAL004W
SEN34
KIN3
POP5
DEP1
TRN1

chrI:189123_C/T; 45 genes affected

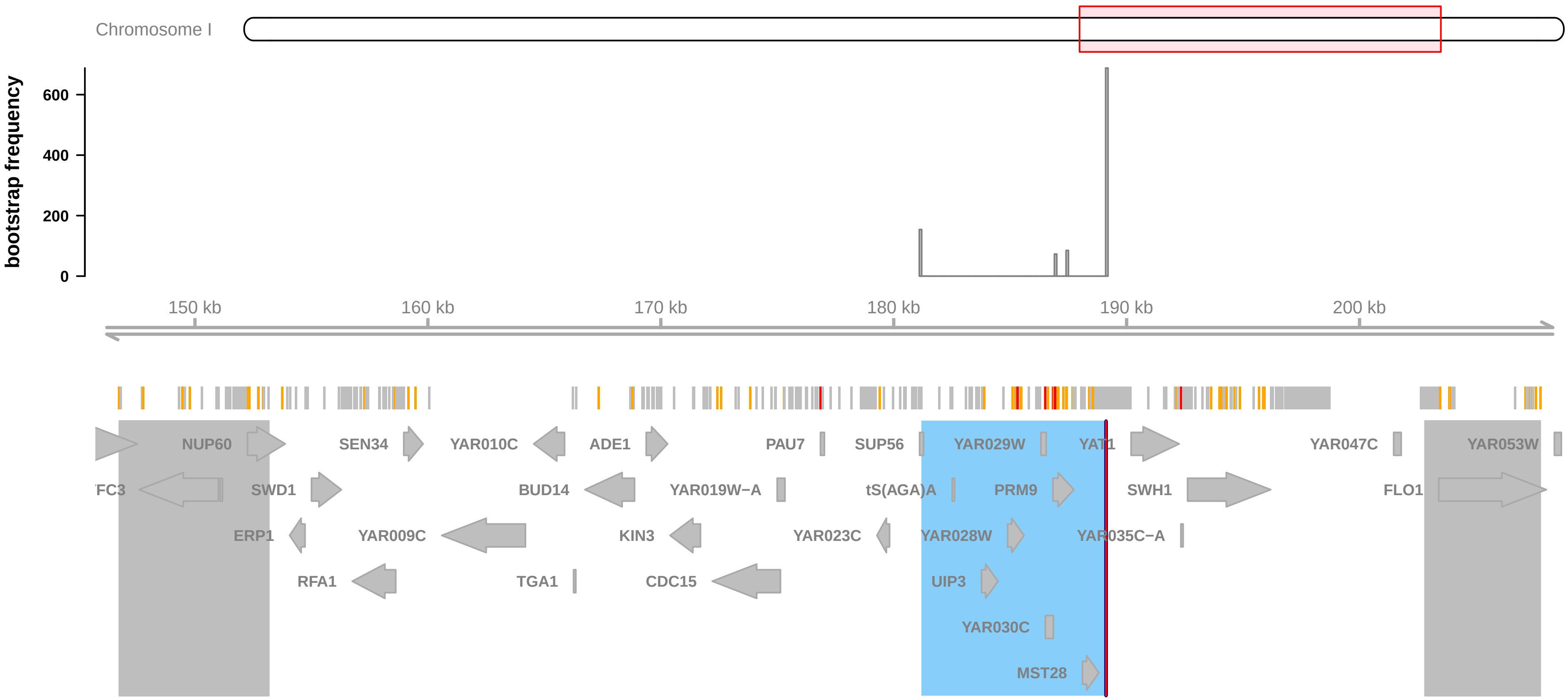

chrII:13061_G/T; 69 genes affected

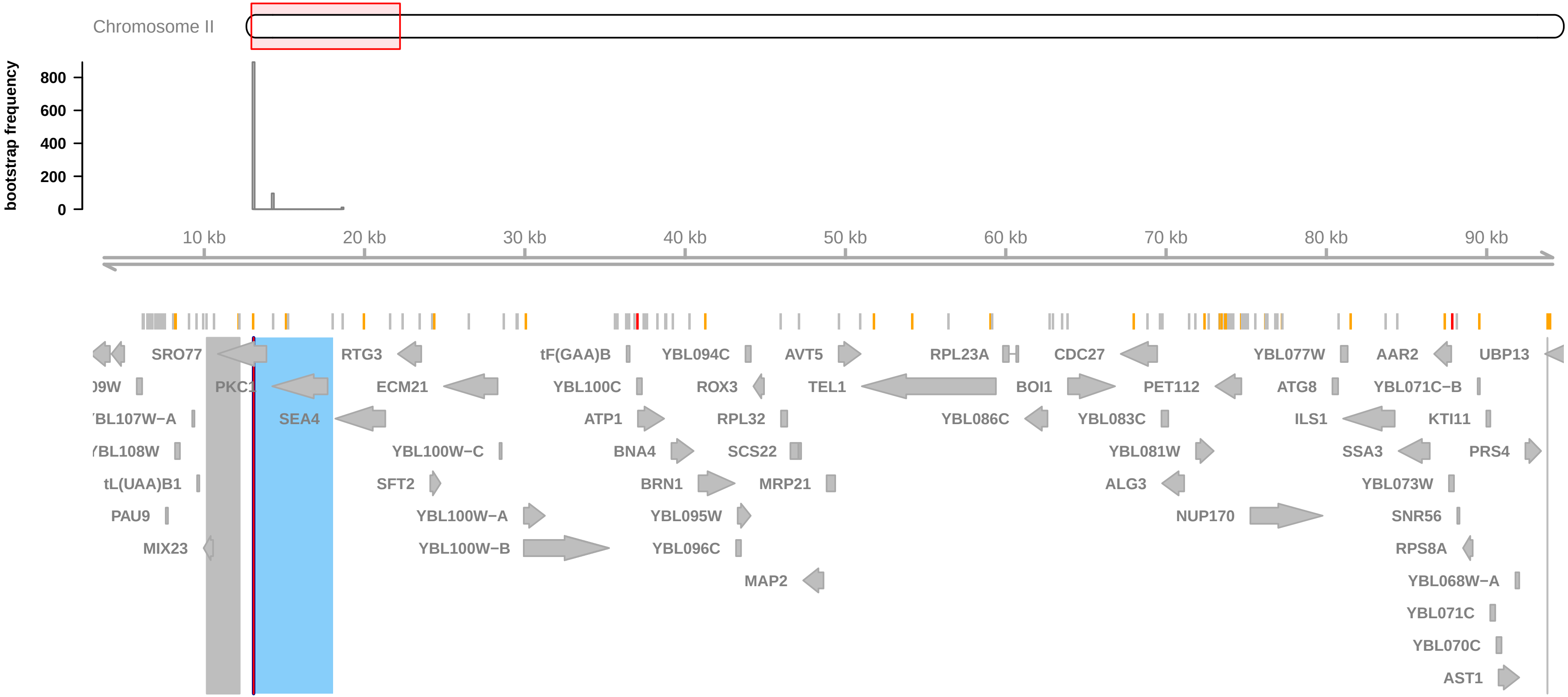

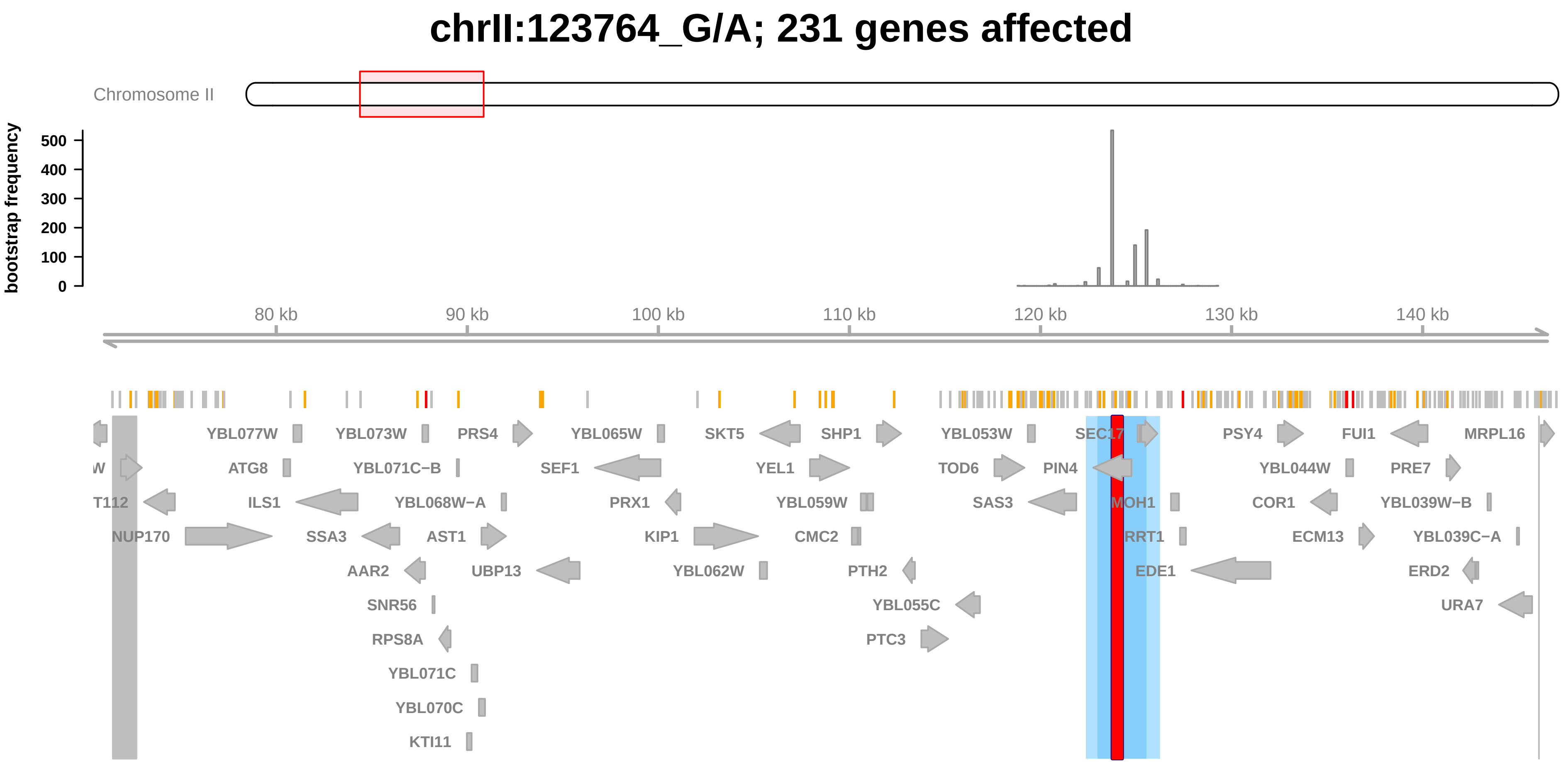

chrII:123764_G/A; 231 genes affected
Chromosome II
bootstrap frequency
500
400
300
200
100
0
80 kb
90 kb
100 kb
110 kb
120 kb
130 kb
140 kb
YBL077W
YBL073W
PRS4
YBL065W
SKT5
SHP1
YBL053W
SEC17
PSY4
FUI1
MRPL16
ATG8
YBL071C−B
SEF1
YEL1
TOD6
PIN4
YBL044W
PRE7
T112
ILS1
YBL068W−A
PRX1
YBL059W
SAS3
MOH1
COR1
YBL039W−B
NUP170
SSA3
AST1
KIP1
CMC2
RRT1
ECM13
YBL039C−A
AAR2
UBP13
YBL062W
PTH2
EDE1
ERD2
SNR56
YBL055C
URA7
RPS8A
PTC3
YBL071C
YBL070C
KTI11

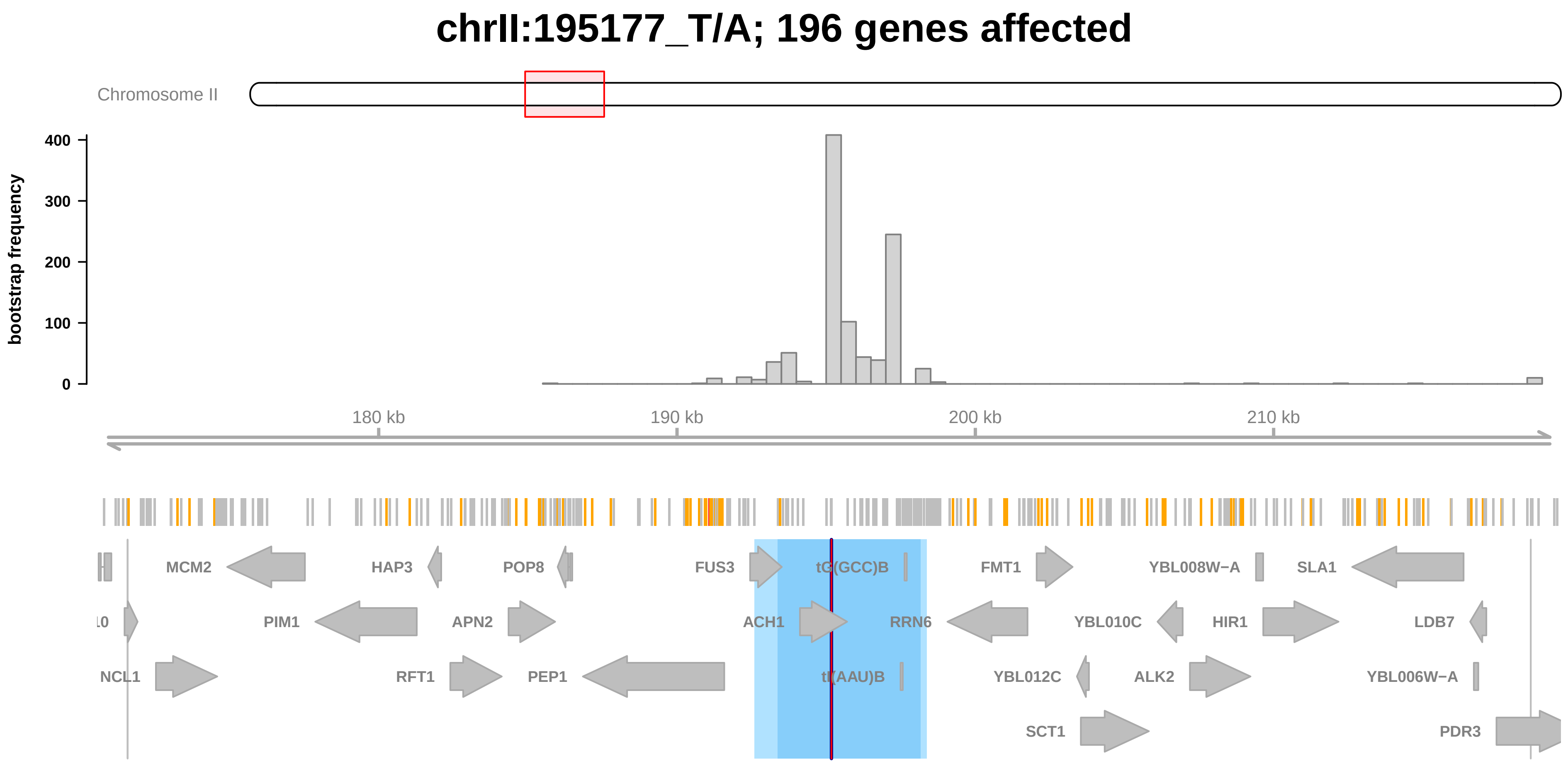
chrII:195177_T/A; 196 genes affected
Chromosome II
bootstrap frequency
400
300
200
100
0
180 kb
190 kb
200 kb
210 kb
MCM2
HAP3
POP8
FUS3
tG(GCC)B
FMT1
YBL008W−A
SLA1
PIM1
APN2
ACH1
RRN6
YBL010C
HIR1
LDB7
NCL1
RFT1
PEP1
tI(AAU)B
YBL012C
ALK2
YBL006W−A
SCT1
PDR3

chrII:370967_C/T; 290 genes affected

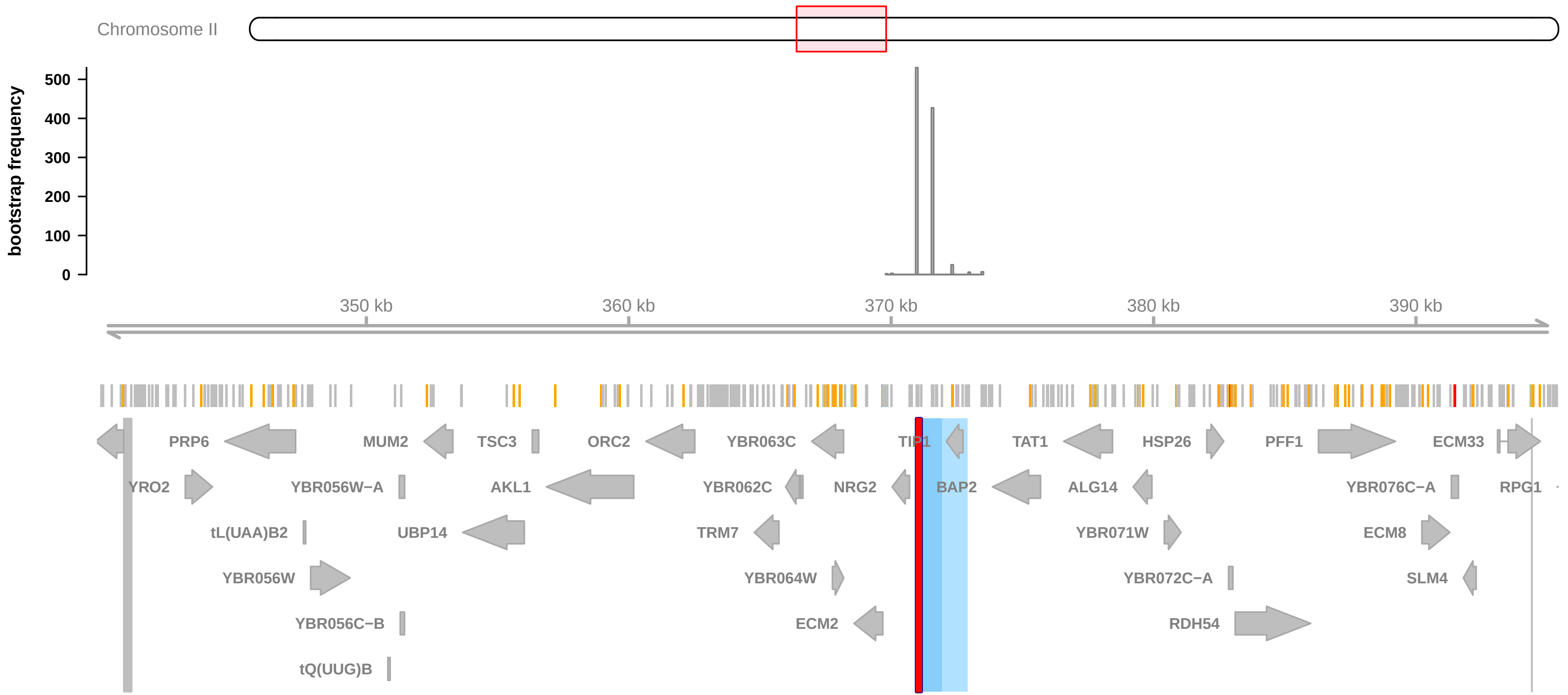

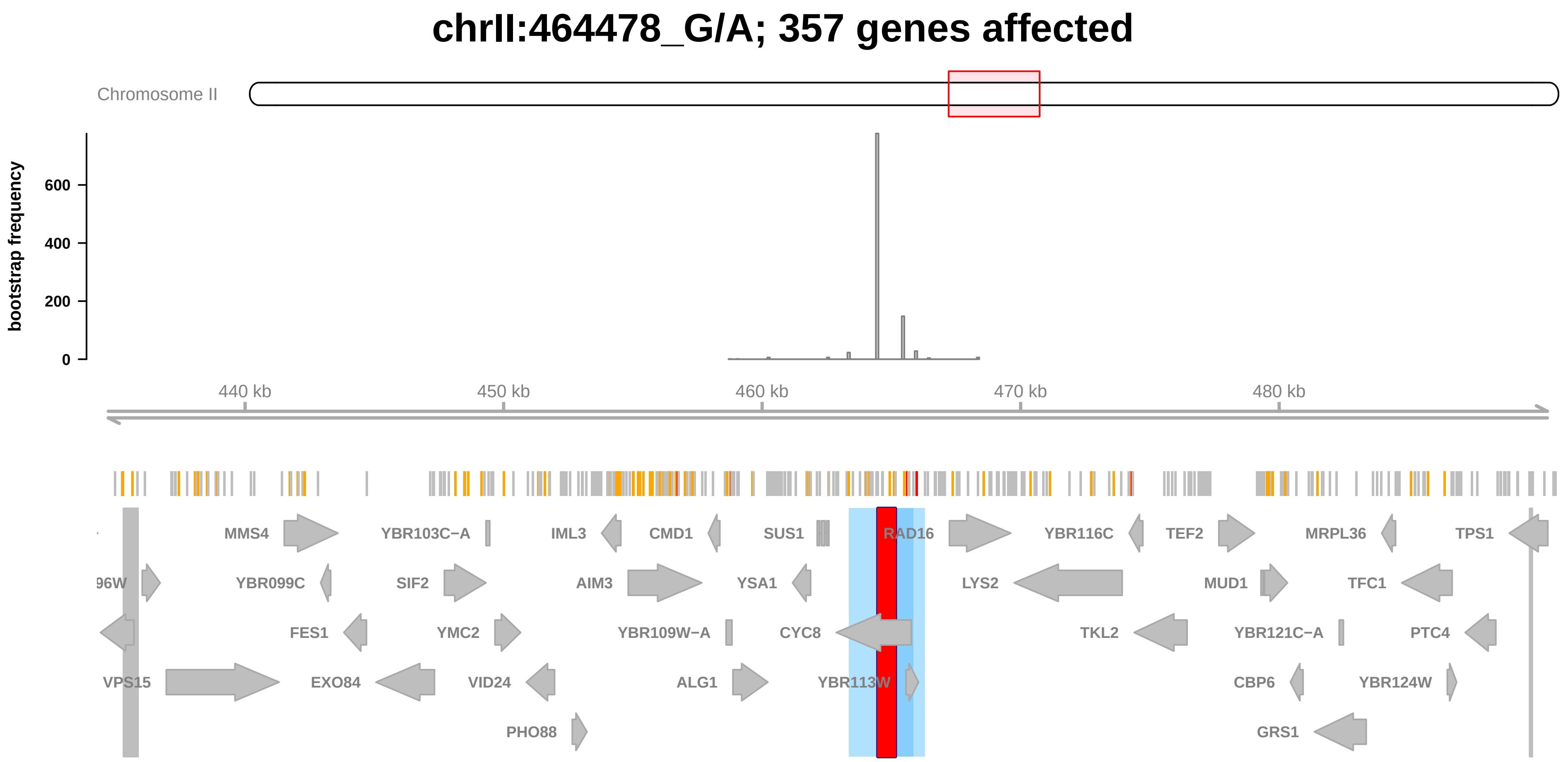

chrII:464478_G/A; 357 genes affected
Chromosome II
bootstrap frequency
600
400
200
0
440 kb
450 kb
460 kb
470 kb
480 kb
MMS4
YBR103C−A
IML3
CMD1
SUS1
RAD16
YBR116C
TEF2
MRPL36
TPS1
96W
YBR099C
SIF2
AIM3
YSA1
LYS2
MUD1
TFC1
FES1
YMC2
YBR109W−A
CYC8
TKL2
YBR121C−A
PTC4
VPS15
EXO84
VID24
ALG1
YBR113W
CBP6
YBR124W
PHO88
GRS1

chrII:541139_G/A; 230 genes affected

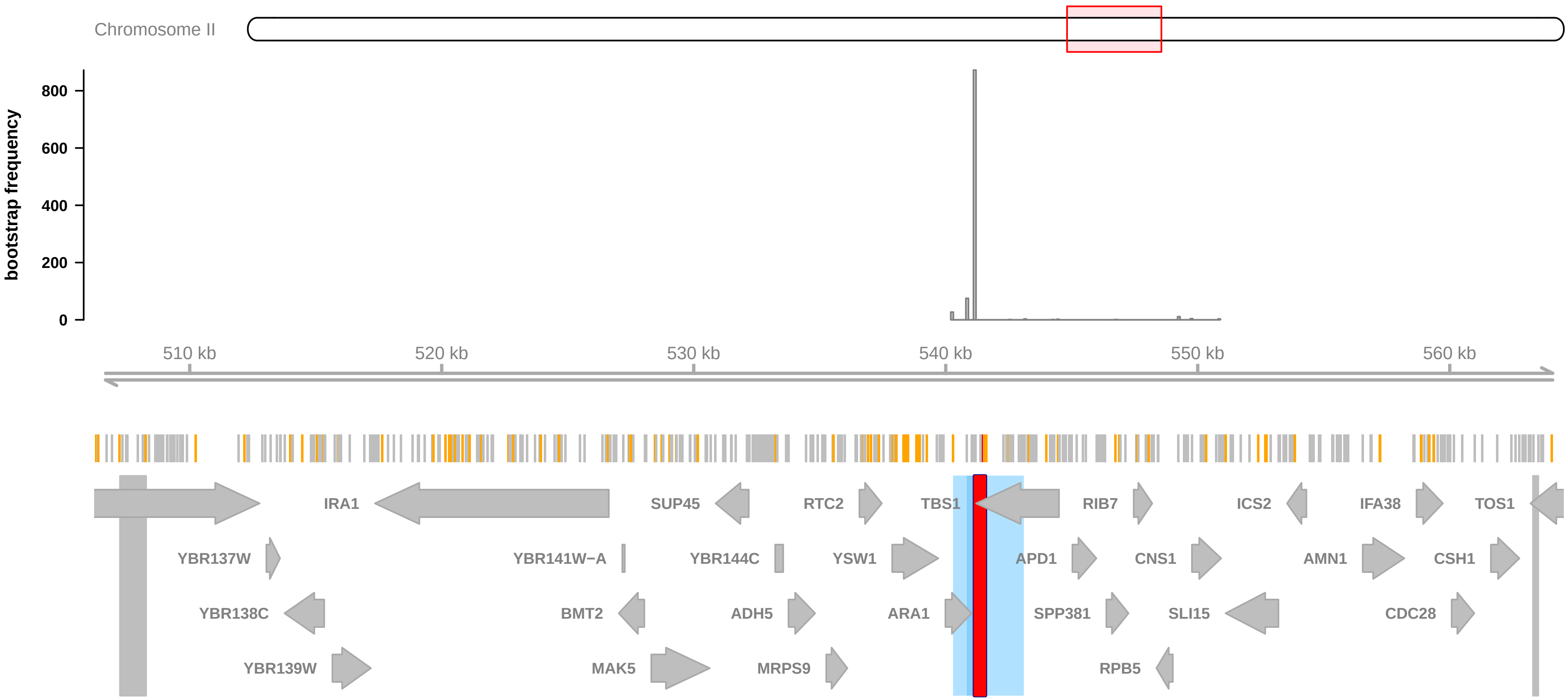

chrII:619028_C/A; 304 genes affected

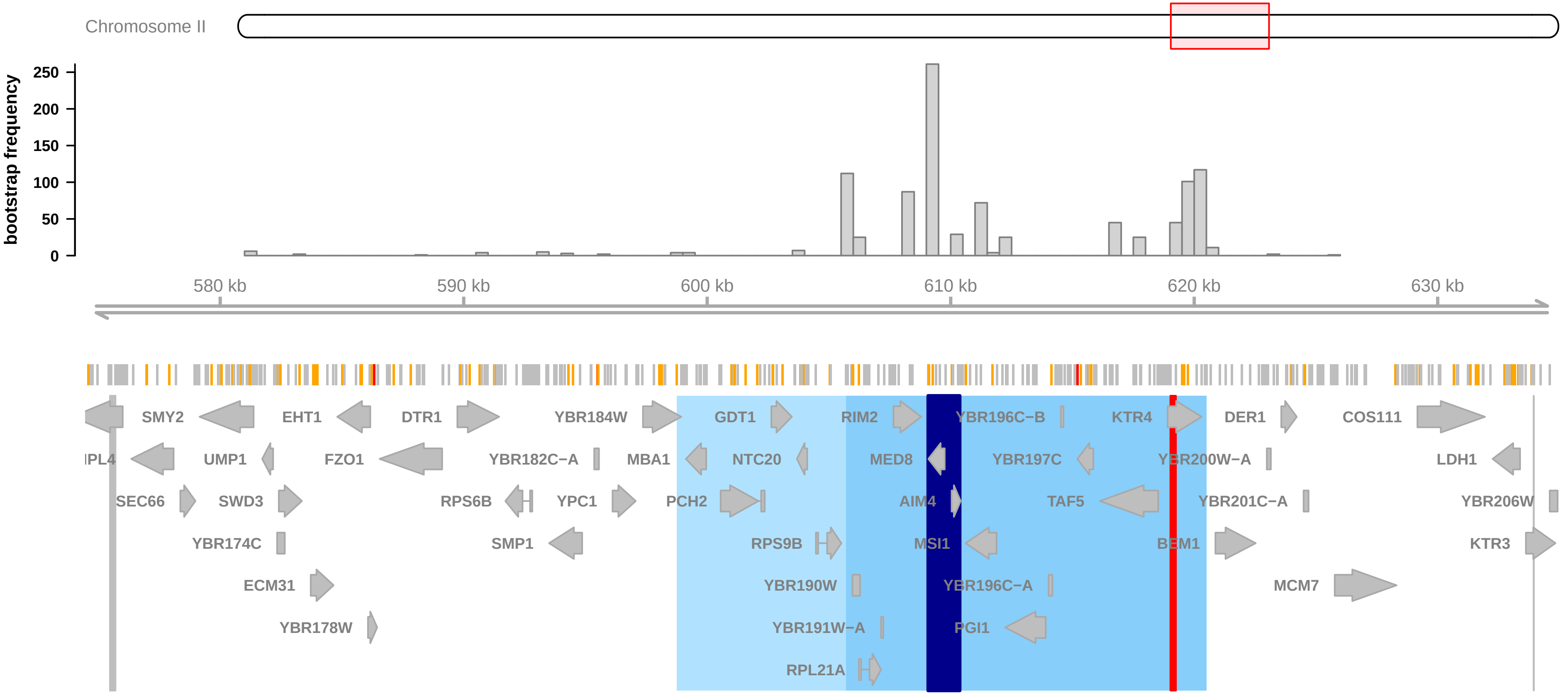

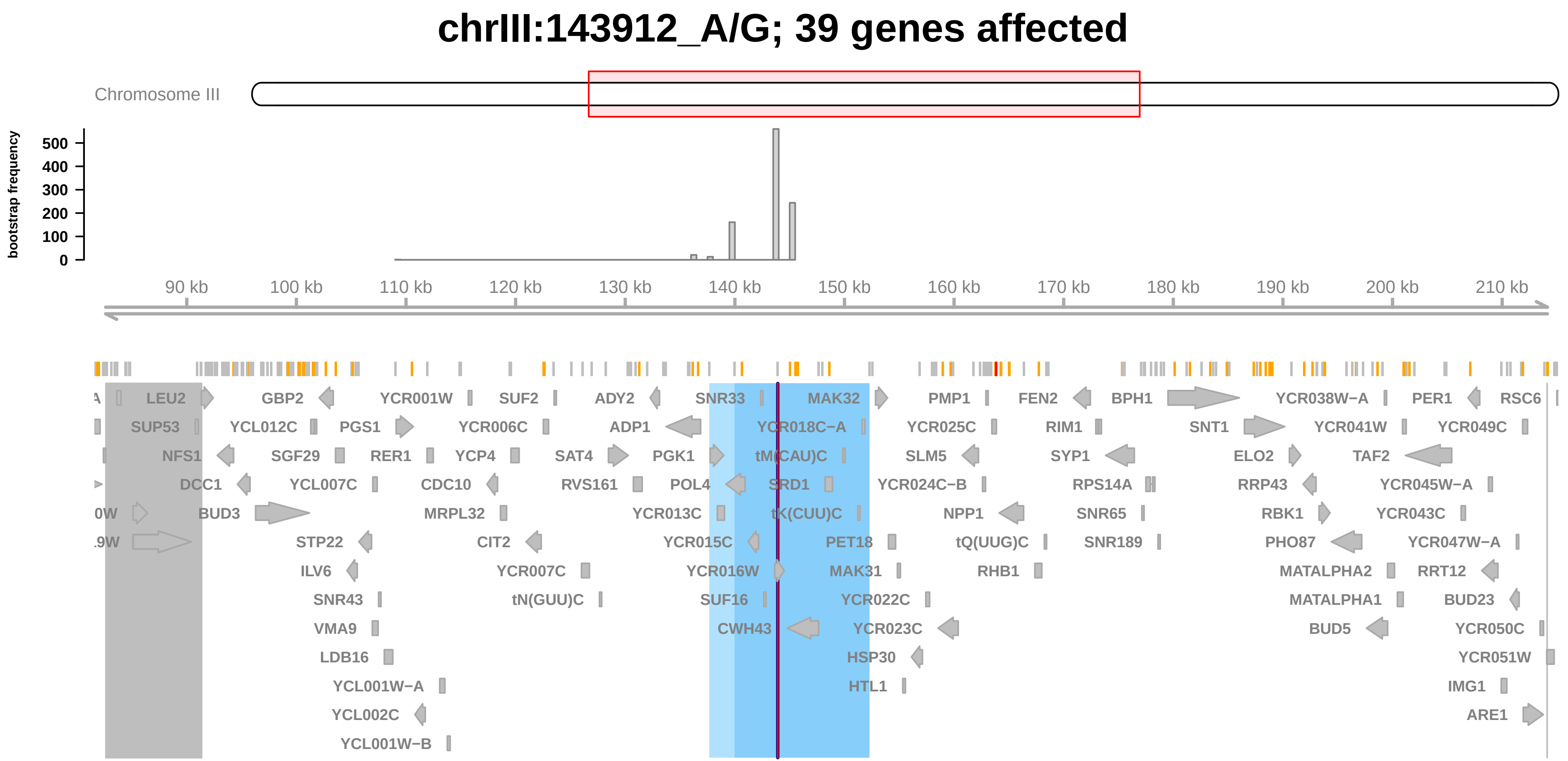

chrIII:143912_A/G; 39 genes affected
Chromosome III
bootstrap frequency
500
400
300
200
100
0
90 kb
100 kb
110 kb
120 kb
130 kb
140 kb
150 kb
160 kb
170 kb
180 kb
190 kb
200 kb
210 kb
LEU2
GBP2
YCR001W
SUF2
ADY2
SNR33
MAK32
PMP1
FEN2
BPH1
YCR038W−A
PER1
RSC6
SUP53
YCL012C
PGS1
YCR006C
ADP1
YCR018C−A
YCR025C
RIM1
SNT1
YCR041W
YCR049C
NFS1
SGF29
RER1
YCP4
SAT4
PGK1
tM(CAU)C
SLM5
SYP1
ELO2
TAF2
DCC1
YCL007C
CDC10
RVS161
POL4
SRD1
YCR024C−B
RPS14A
RRP43
YCR045W−A
BUD3
MRPL32
YCR013C
tK(CUU)C
NPP1
SNR65
RBK1
YCR043C
STP22
CIT2
YCR015C
PET18
tQ(UUG)C
SNR189
PHO87
YCR047W−A
ILV6
YCR007C
YCR016W
MAK31
RHB1
MATALPHA2
RRT12
SNR43
tN(GUU)C
SUF16
YCR022C
MATALPHA1
BUD23
VMA9
CWH43
YCR023C
BUD5
YCR050C
LDB16
HSP30
YCR051W
YCL001W−A
HTL1
IMG1
YCL002C
ARE1
YCL001W−B

chrIII:166390_G/C; 27 genes affected

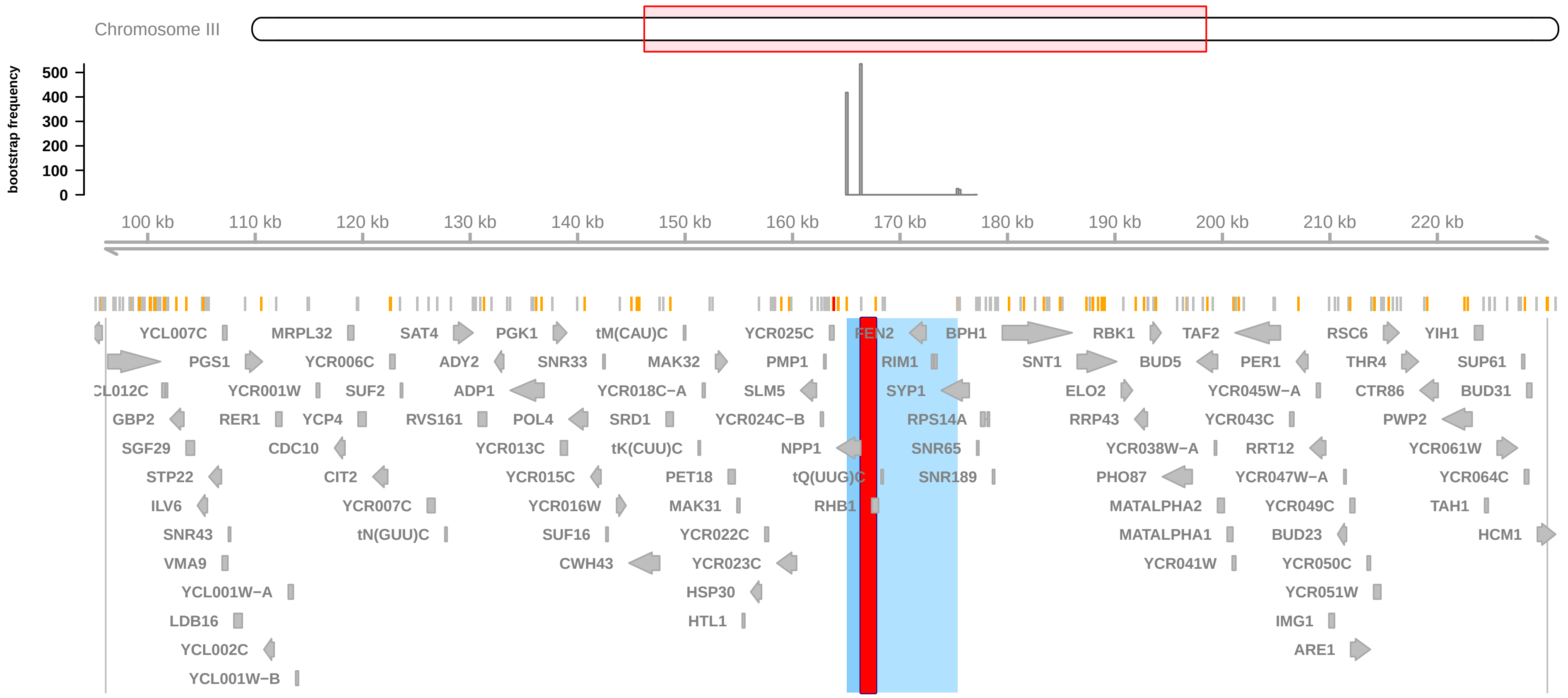

chrIII:201185_C/A; 80 genes affected

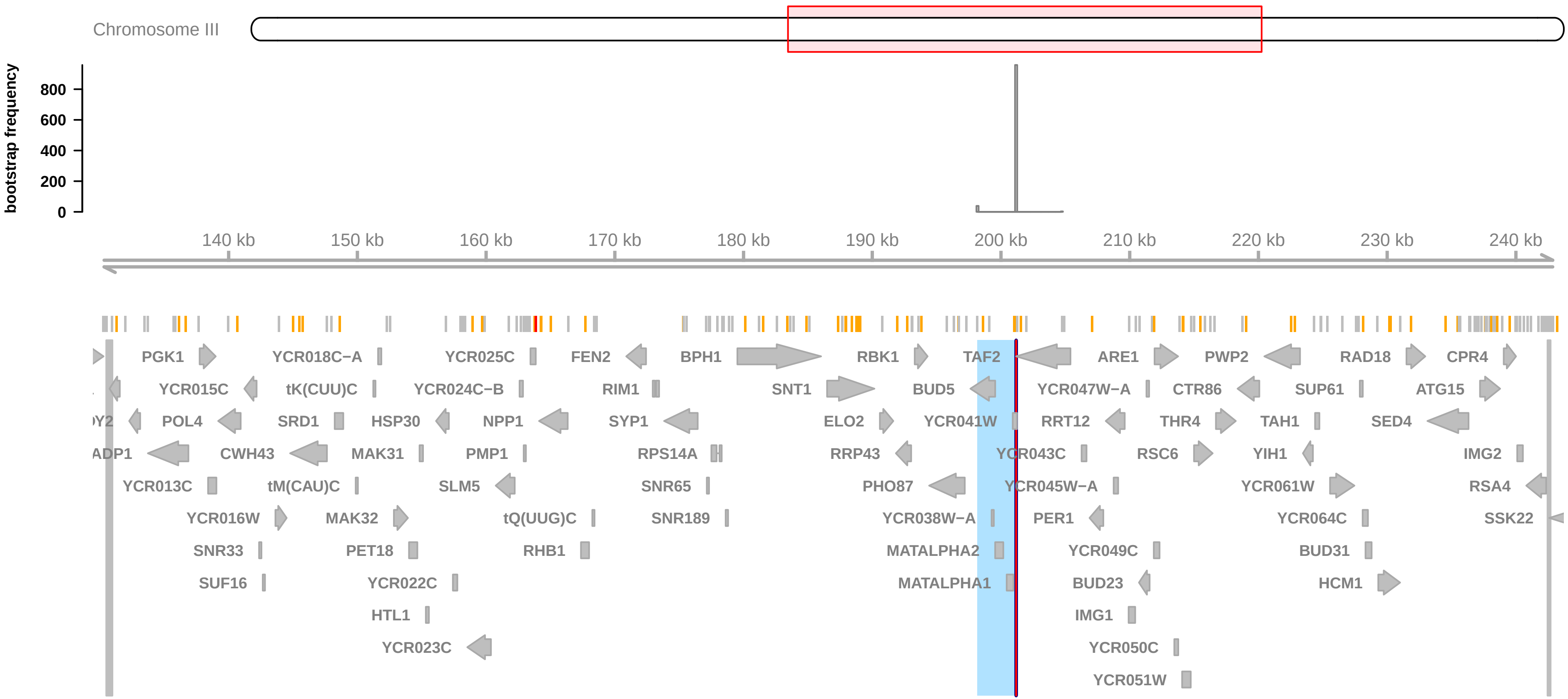

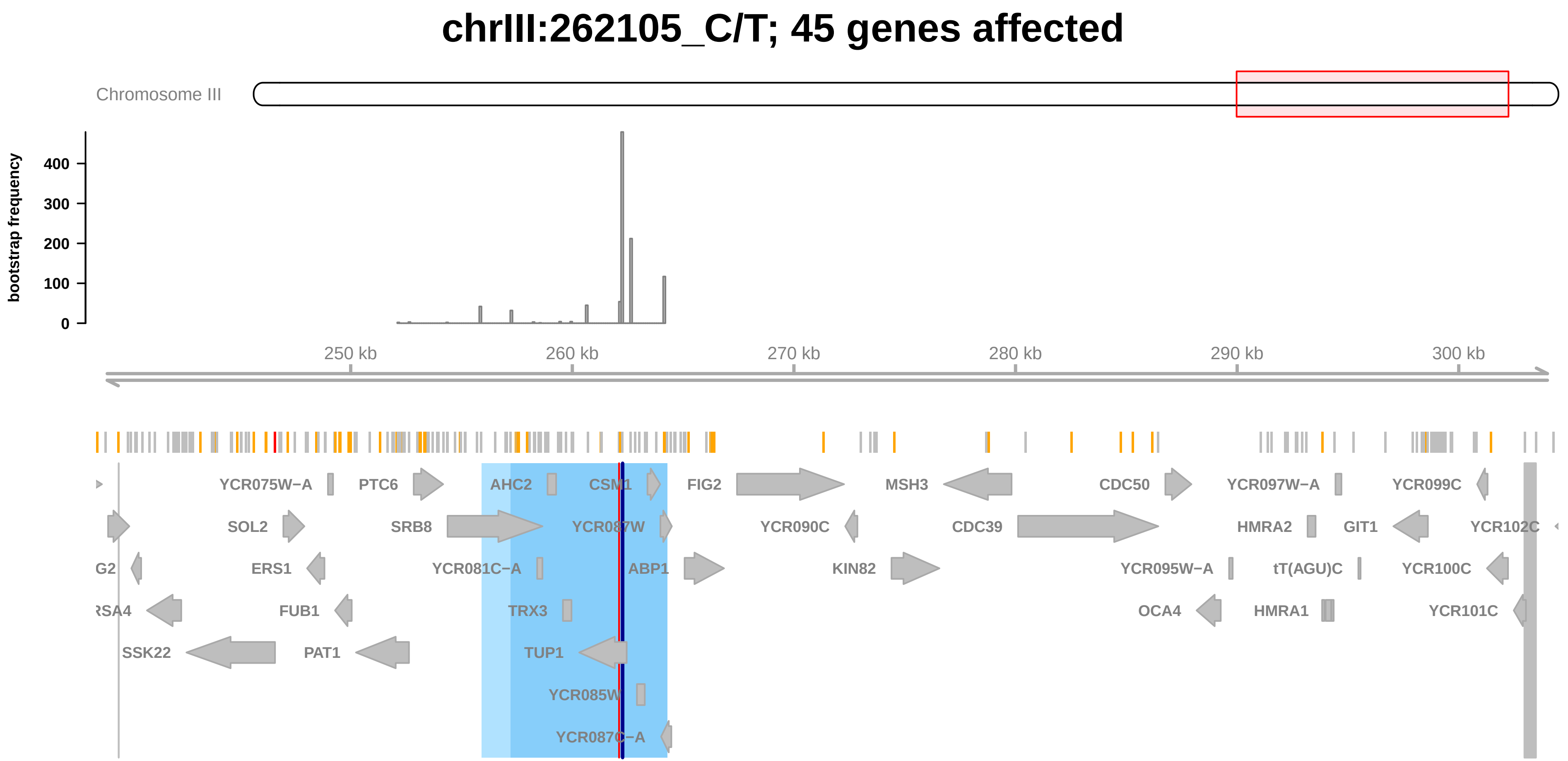
chrIII:262105_C/T; 45 genes affected
Chromosome III
bootstrap frequency
0
100
200
300
400
250 kb
260 kb
270 kb
280 kb
290 kb
300 kb
YCR075W−A
PTC6
AHC2
CSM1
FIG2
MSH3
CDC50
YCR097W−A
YCR099C
SOL2
SRB8
YCR087W
YCR090C
CDC39
HMRA2
GIT1
YCR102C
G2
ERS1
YCR081C−A
ABP1
KIN82
YCR095W−A
tT(AGU)C
YCR100C
RSA4
FUB1
TRX3
OCA4
HMRA1
YCR101C
SSK22
PAT1
TUP1
YCR085W
YCR087C−A

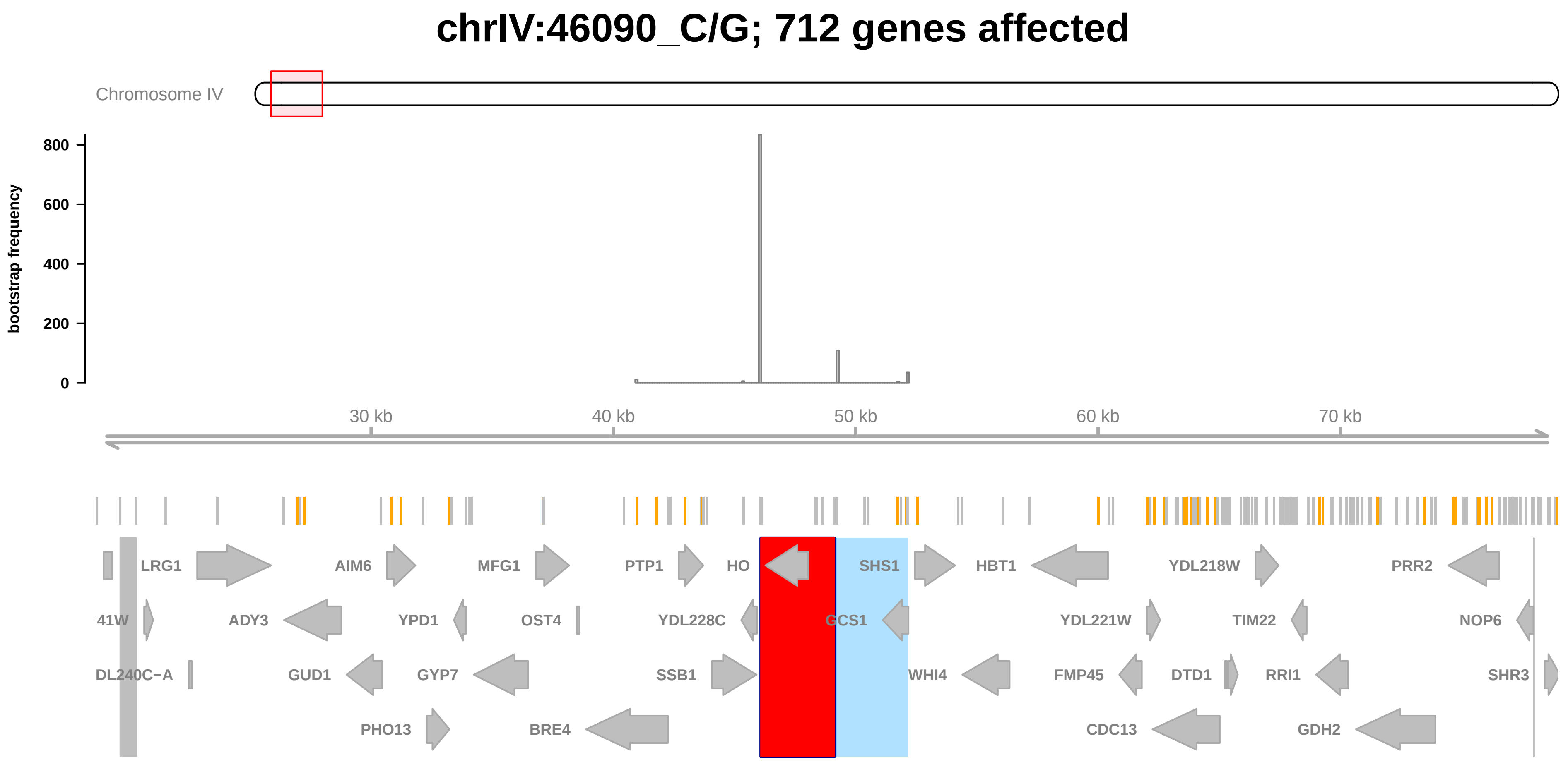
chrIV:46090_C/G; 712 genes affected
Chromosome IV
bootstrap frequency
800
600
400
200
0
30 kb
40 kb
50 kb
60 kb
70 kb
LRG1
AIM6
MFG1
PTP1
HO
SHS1
HBT1
YDL218W
PRR2
ADY3
YPD1
OST4
YDL228C
GCS1
YDL221W
TIM22
NOP6
GUD1
GYP7
SSB1
WHI4
FMP45
DTD1
RRI1
SHR3
PHO13
BRE4
CDC13
GDH2

chrIV:156943_A/G; 916 genes affected

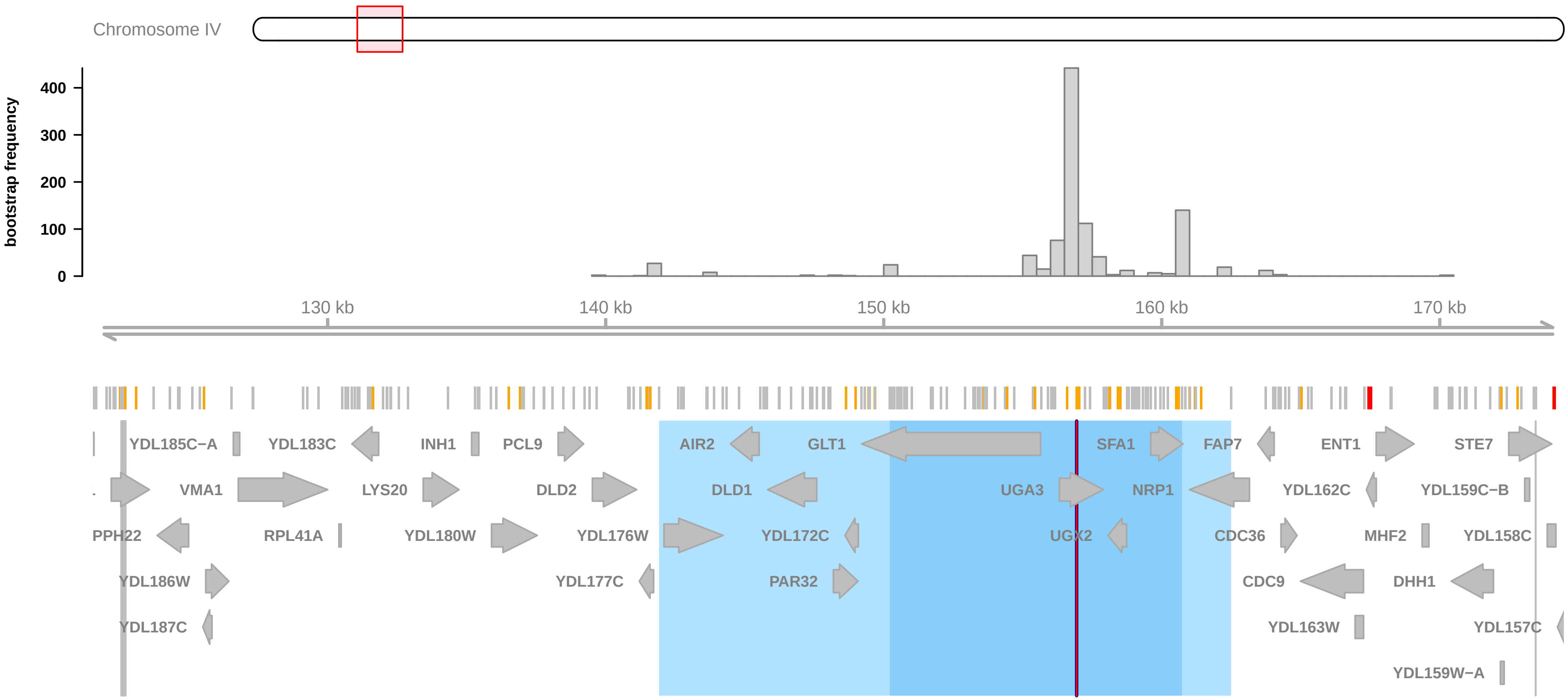

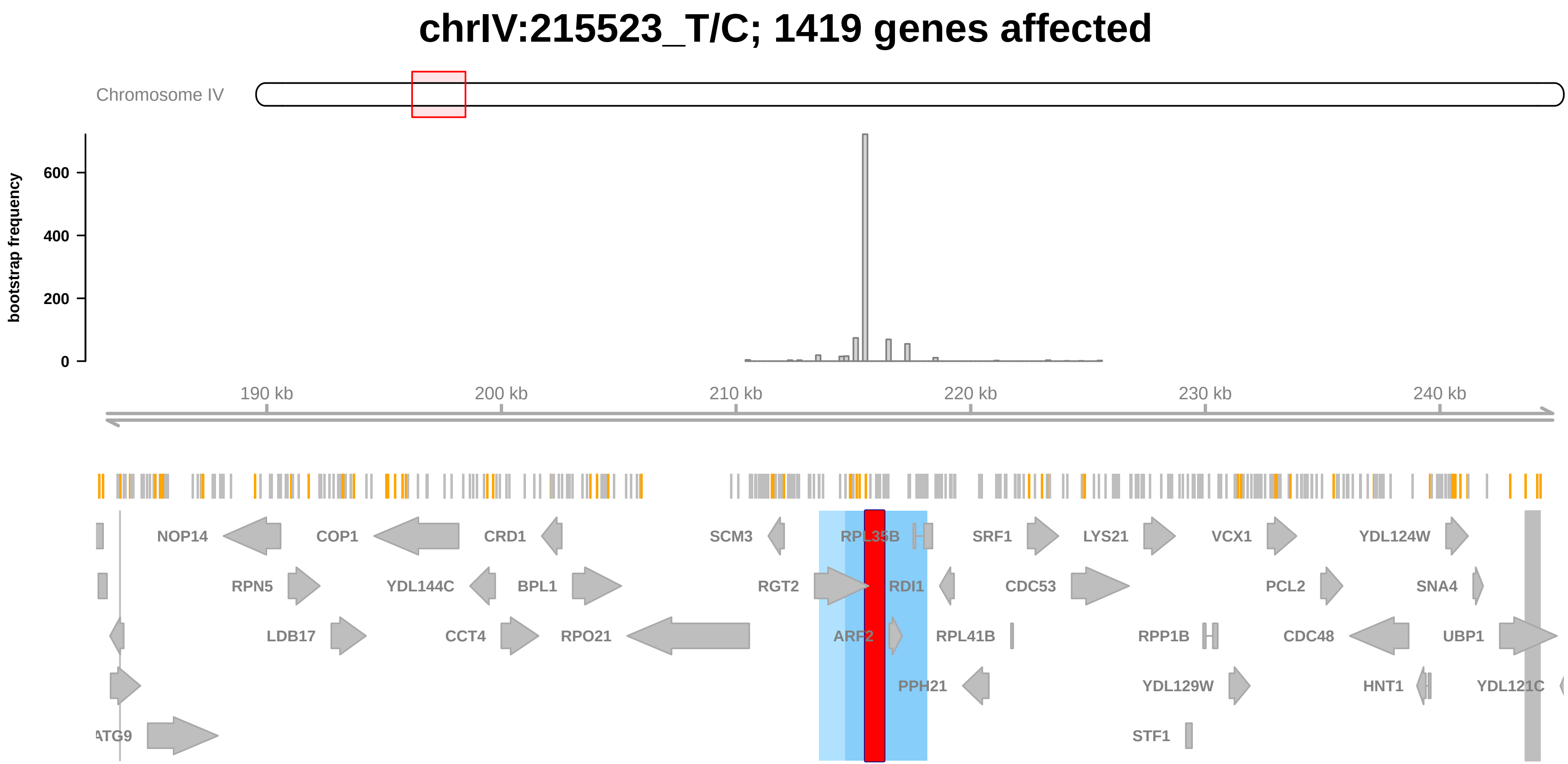
chrIV:215523_T/C; 1419 genes affected
Chromosome IV
bootstrap frequency
0
200
400
600
190 kb
200 kb
210 kb
220 kb
230 kb
240 kb
NOP14
COP1
CRD1
SCM3
RPL35B
SRF1
LYS21
VCX1
YDL124W
RPN5
YDL144C
BPL1
RGT2
RDI1
CDC53
PCL2
SNA4
LDB17
CCT4
RPO21
ARF2
RPL41B
RPP1B
CDC48
UBP1
PPH21
YDL129W
HNT1
YDL121C
STF1

chrIV:461656_A/G; 1154 genes affected

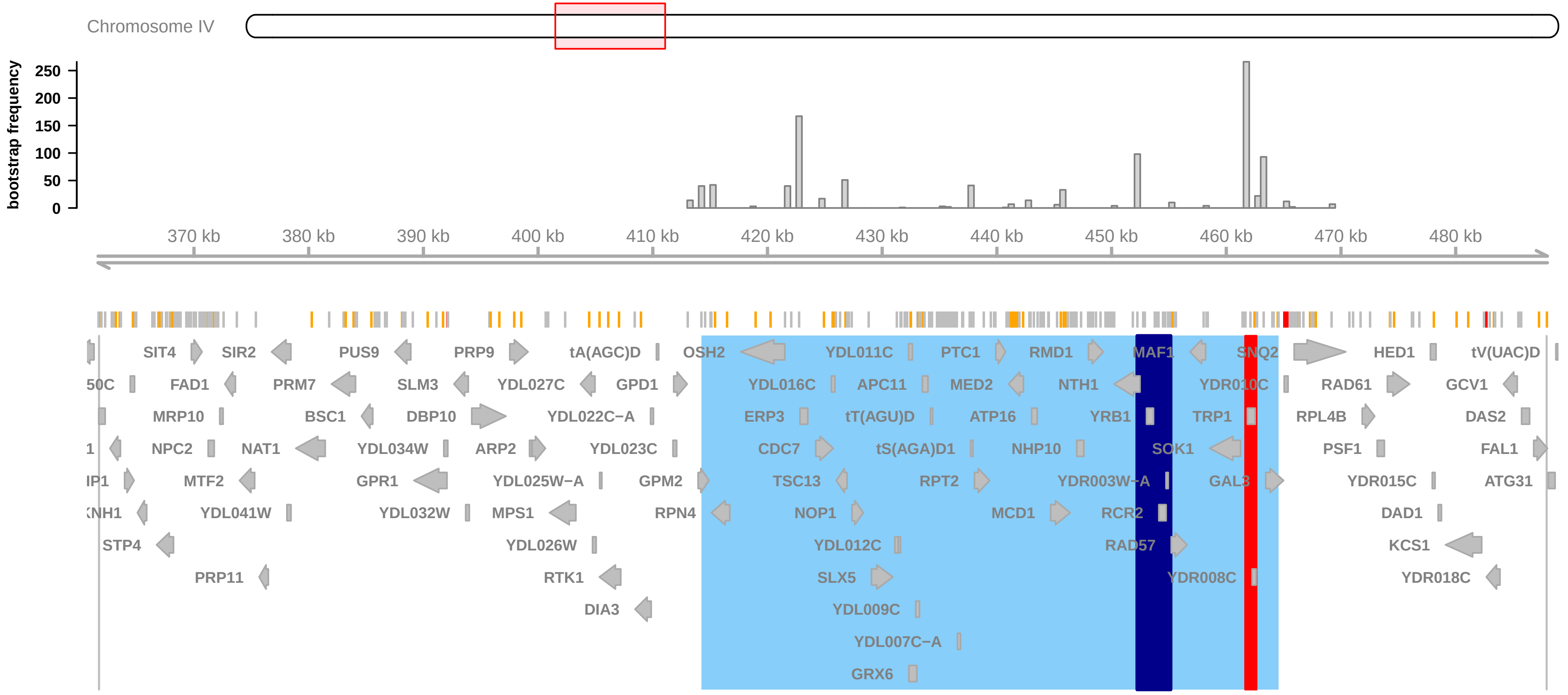

chrIV:509407_G/T; 757 genes affected

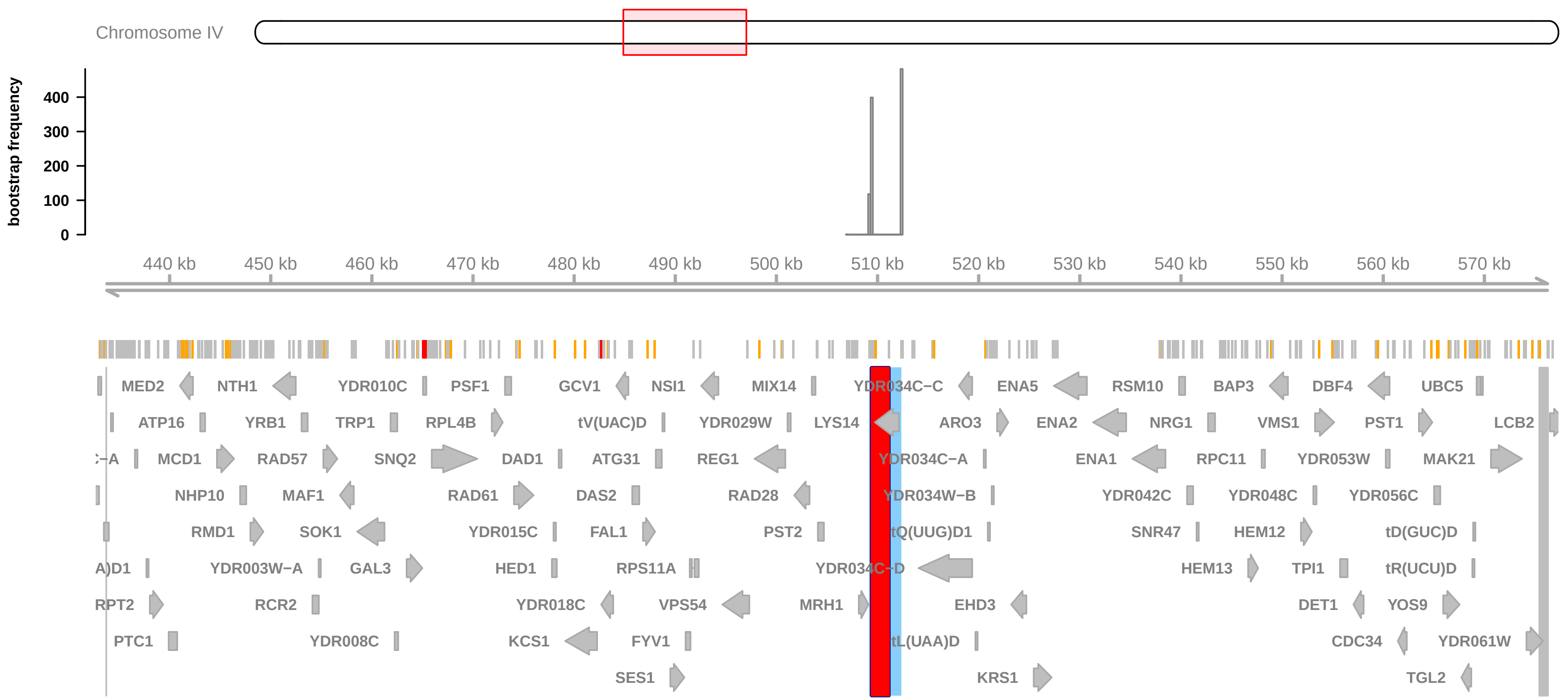

chrIV:637663_A/T; 913 genes affected

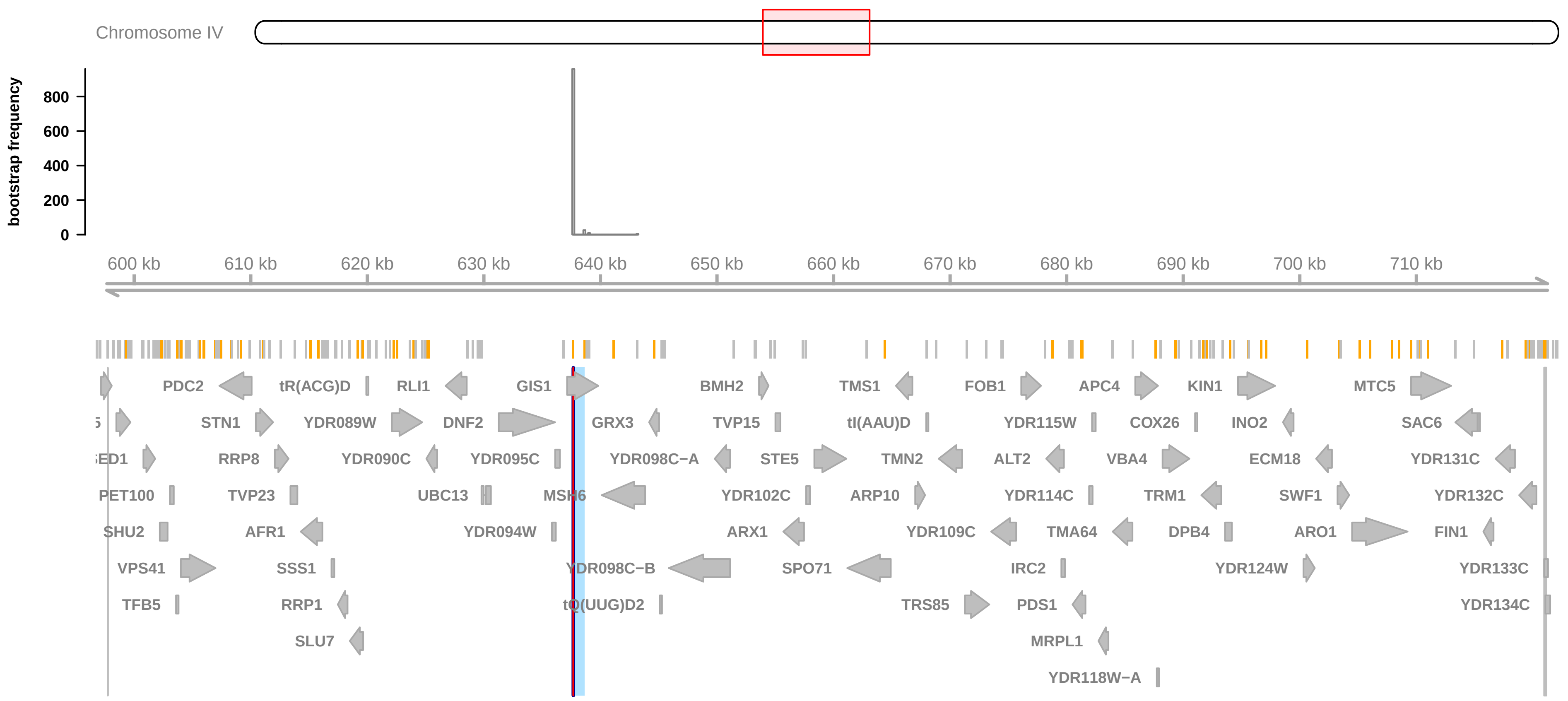

chrIV:681304_G/A; 1182 genes affected

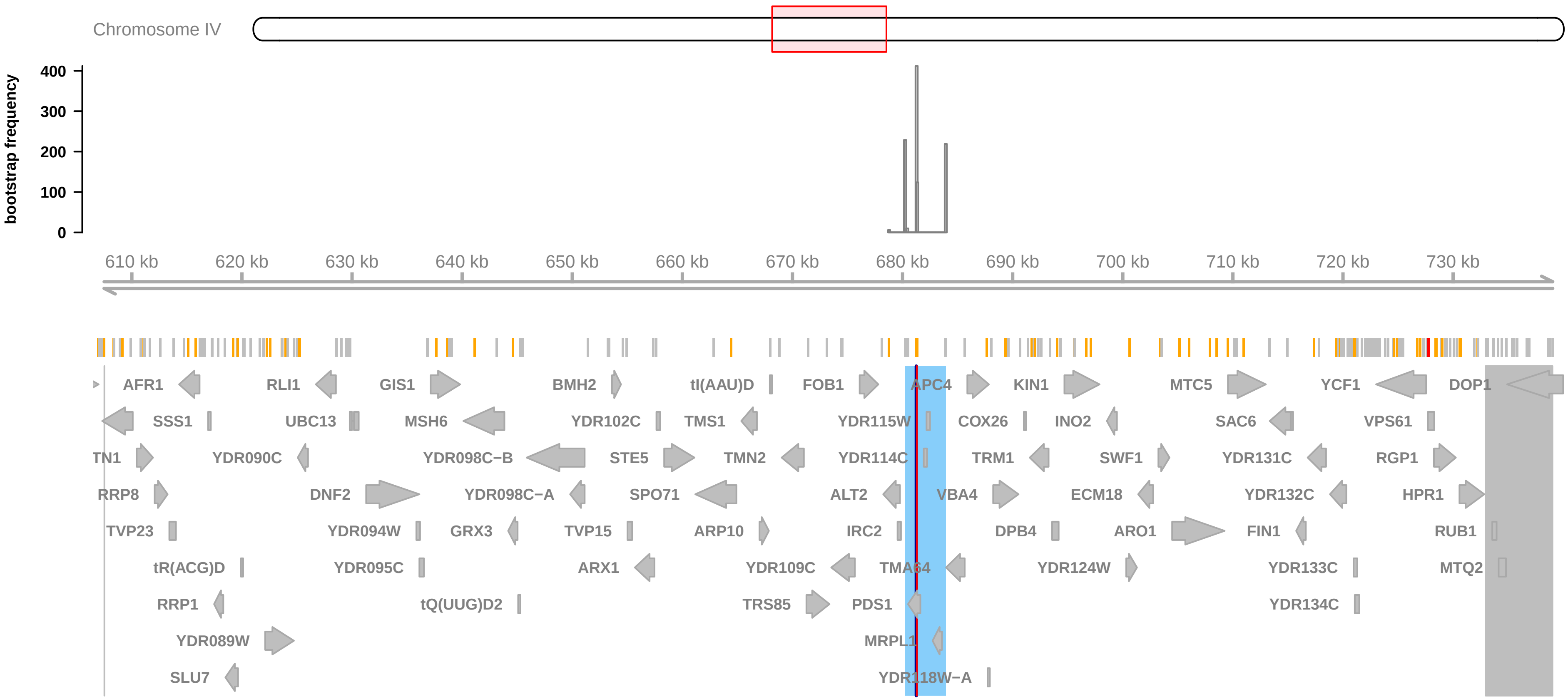

chrIV:745766_G/A; 858 genes affected

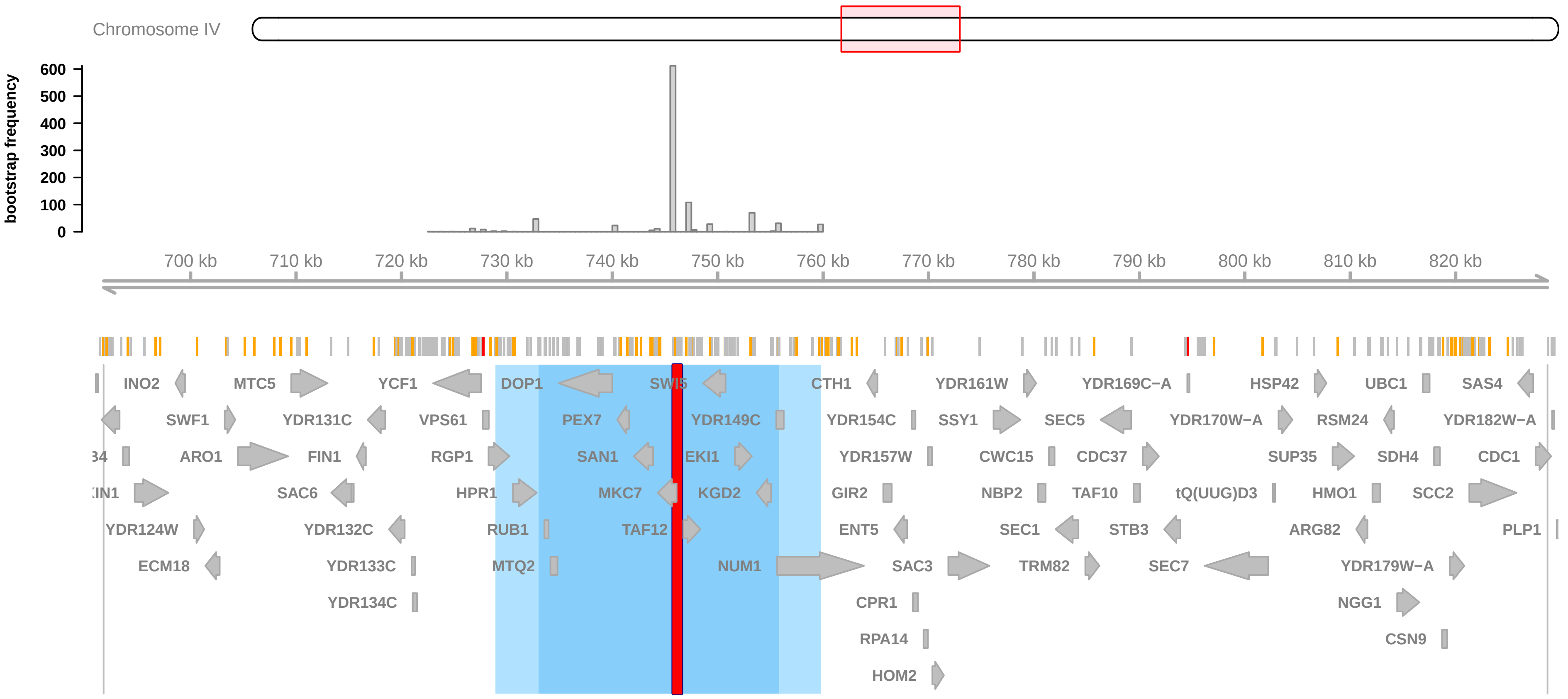

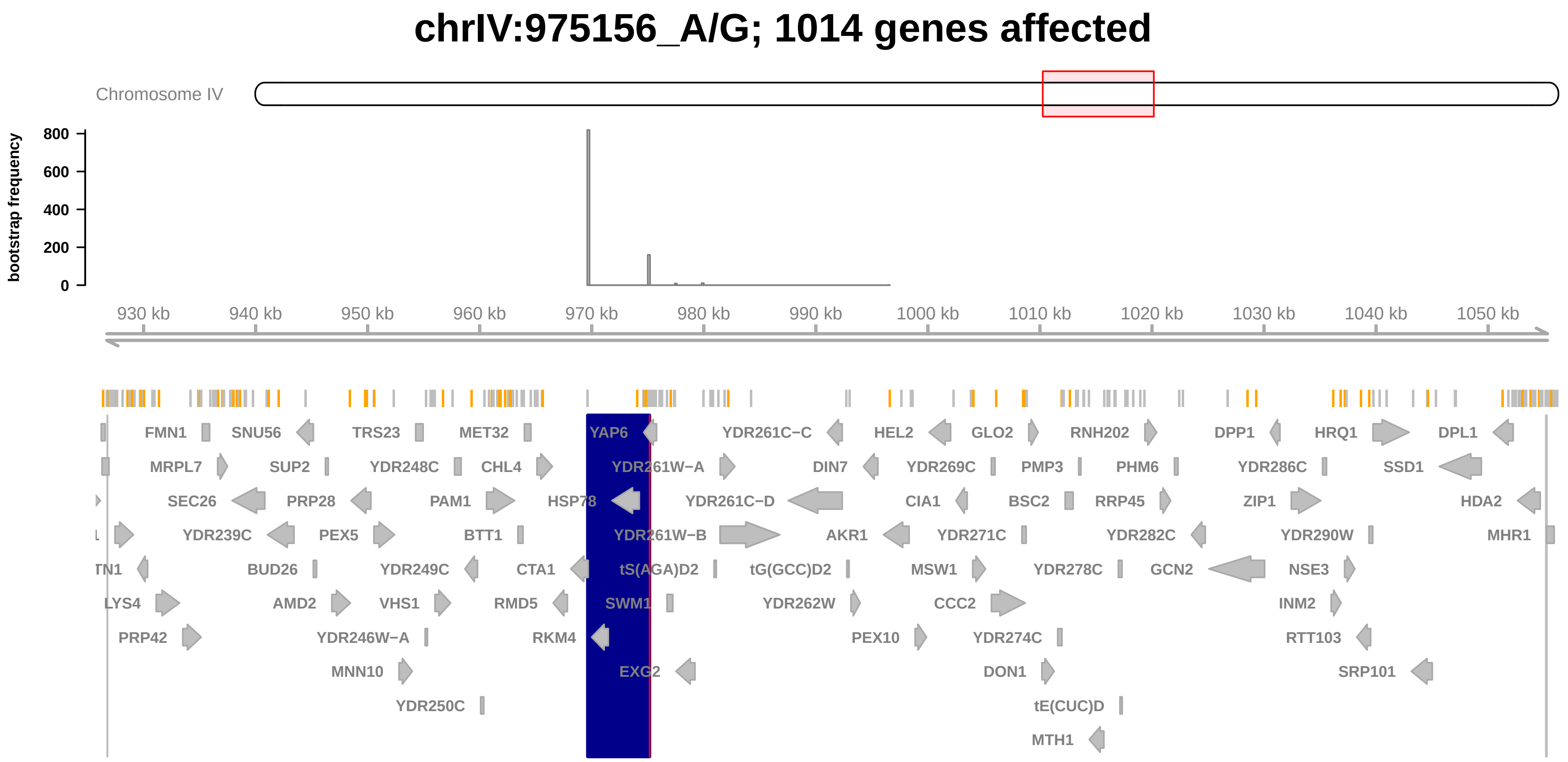
chrIV:975156_A/G; 1014 genes affected
Chromosome IV
bootstrap frequency
0
200
400
600
800
930 kb
940 kb
950 kb
960 kb
970 kb
980 kb
990 kb
1000 kb
1010 kb
1020 kb
1030 kb
1040 kb
1050 kb
FMN1
SNU56
TRS23
MET32
YAP6
YDR261C−C
HEL2
GLO2
RNH202
DPP1
HRQ1
DPL1
MRPL7
SUP2
YDR248C
CHL4
YDR261W−A
DIN7
YDR269C
PMP3
PHM6
YDR286C
SSD1
SEC26
PRP28
PAM1
HSP78
YDR261C−D
CIA1
BSC2
RRP45
ZIP1
HDA2
YDR239C
PEX5
BTT1
YDR261W−B
AKR1
YDR271C
YDR282C
YDR290W
MHR1
BUD26
YDR249C
CTA1
tS(AGA)D2
tG(GCC)D2
MSW1
YDR278C
GCN2
NSE3
LYS4
AMD2
VHS1
RMD5
SWM1
YDR262W
CCC2
INM2
PRP42
YDR246W−A
RKM4
PEX10
YDR274C
RTT103
MNN10
EXG2
DON1
SRP101
YDR250C
tE(CUC)D
MTH1

chrIV:1182010_T/C; 798 genes affected

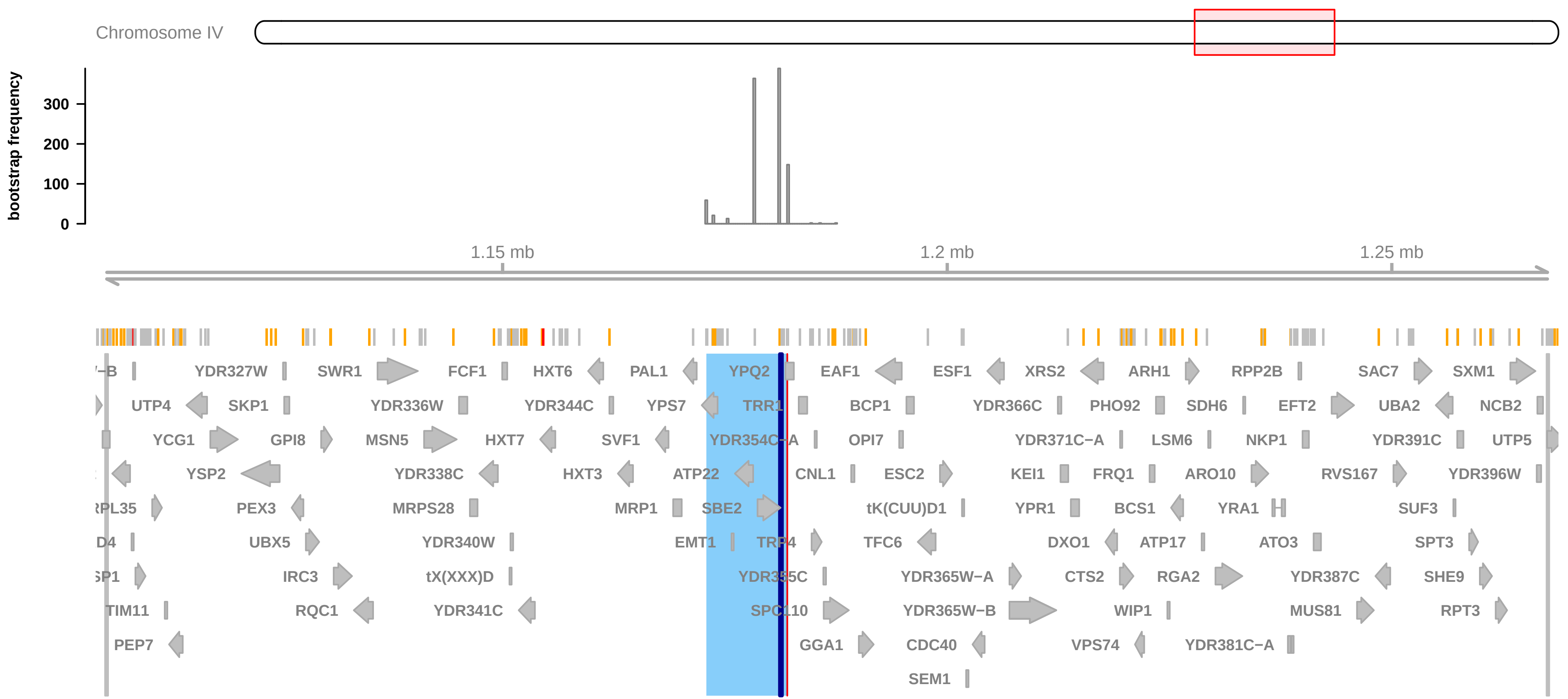

chrIV:1357789_T/G; 988 genes affected

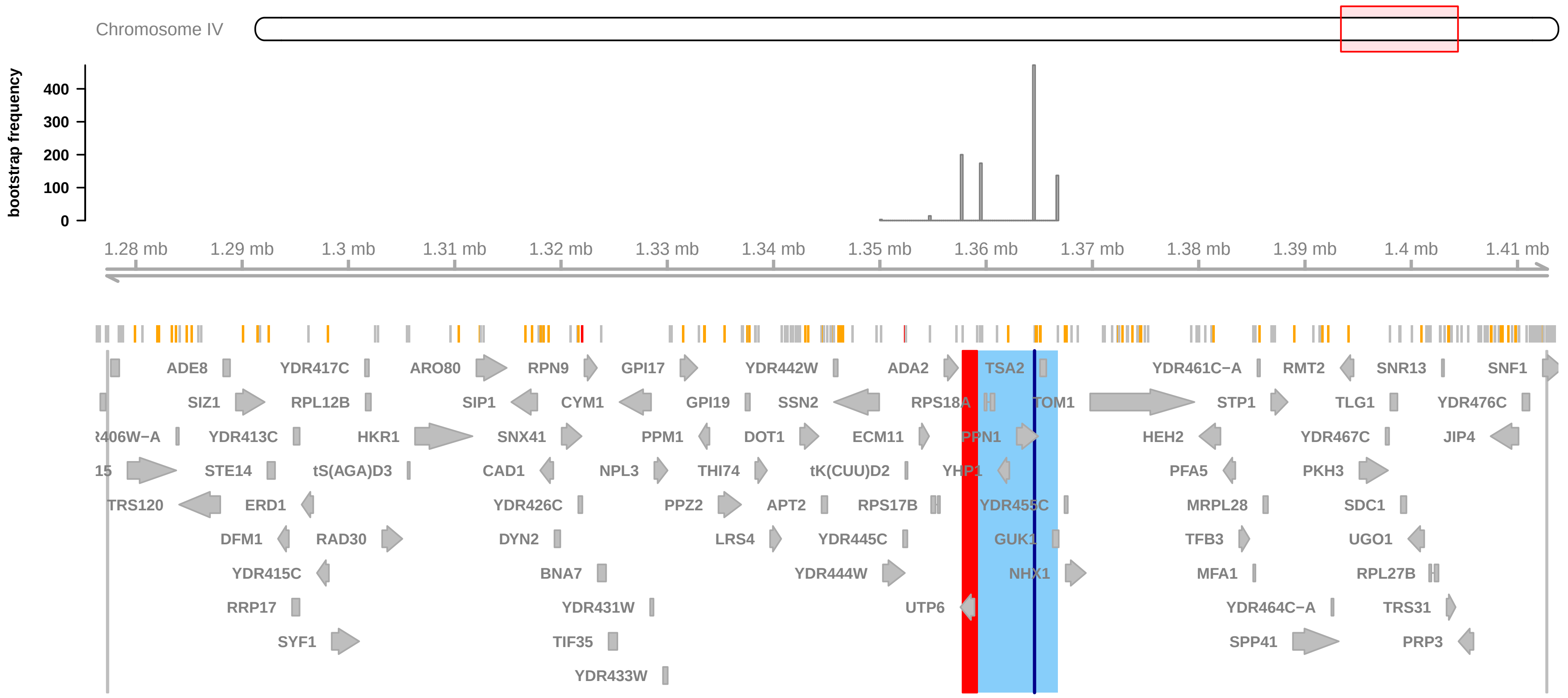

chrIV:1521369_A/C; 545 genes affected

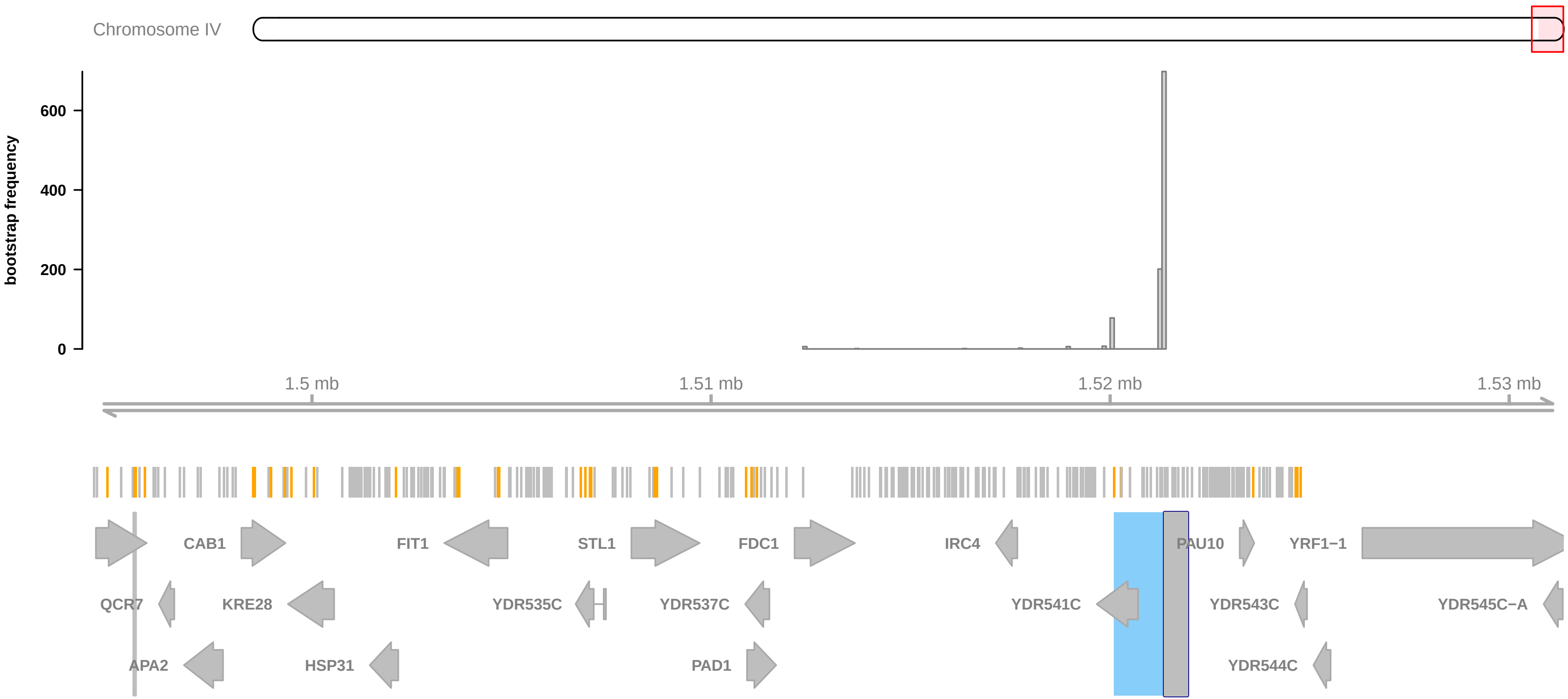

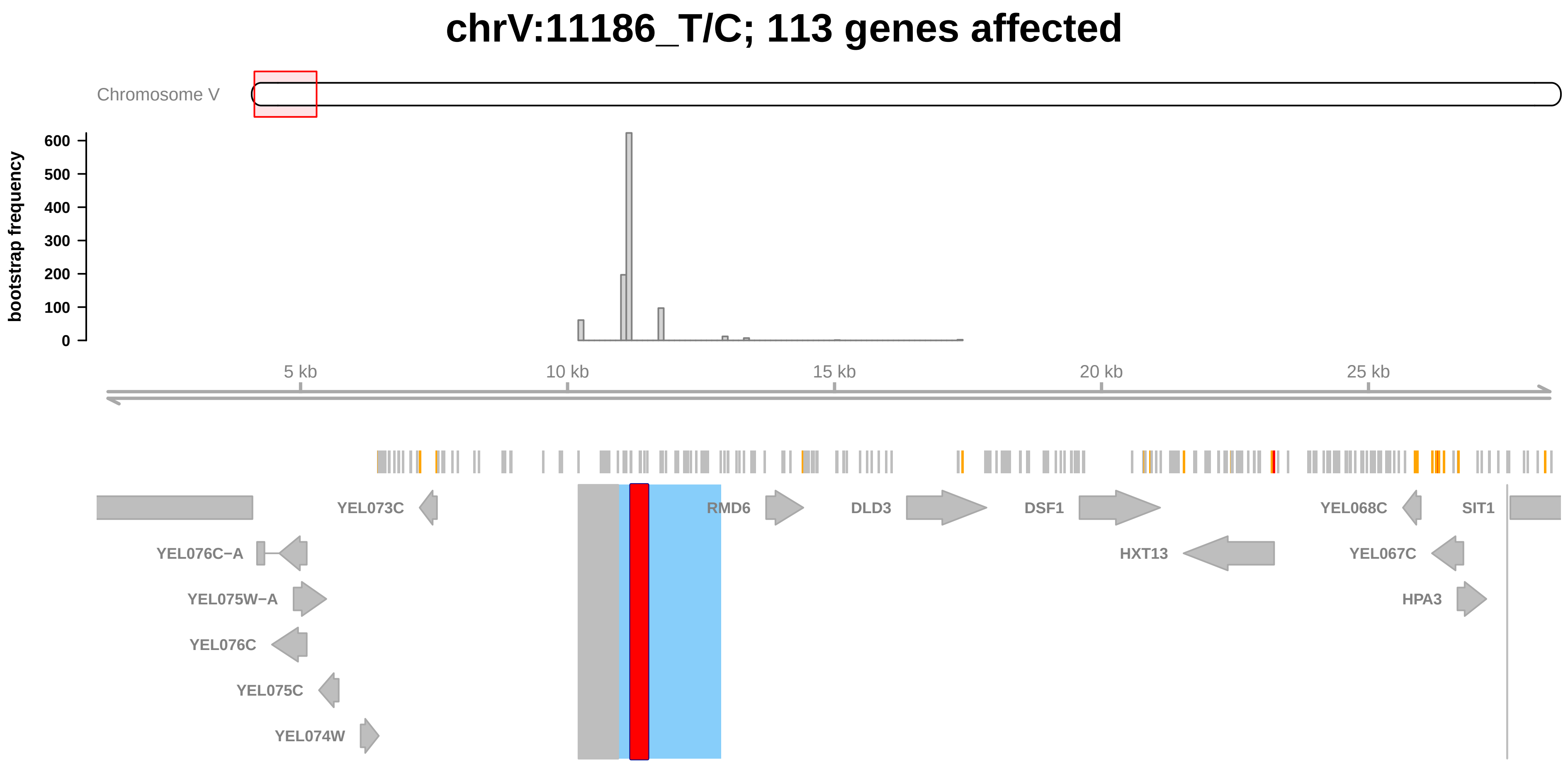
chrV:11186_T/C; 113 genes affected
Chromosome V
bootstrap frequency
600
500
400
300
200
100
0
5 kb
10 kb
15 kb
20 kb
25 kb
YEL073C
RMD6
DLD3
DSF1
YEL068C
SIT1
YEL076C−A
HXT13
YEL067C
YEL075W−A
HPA3
YEL076C
YEL075C
YEL074W

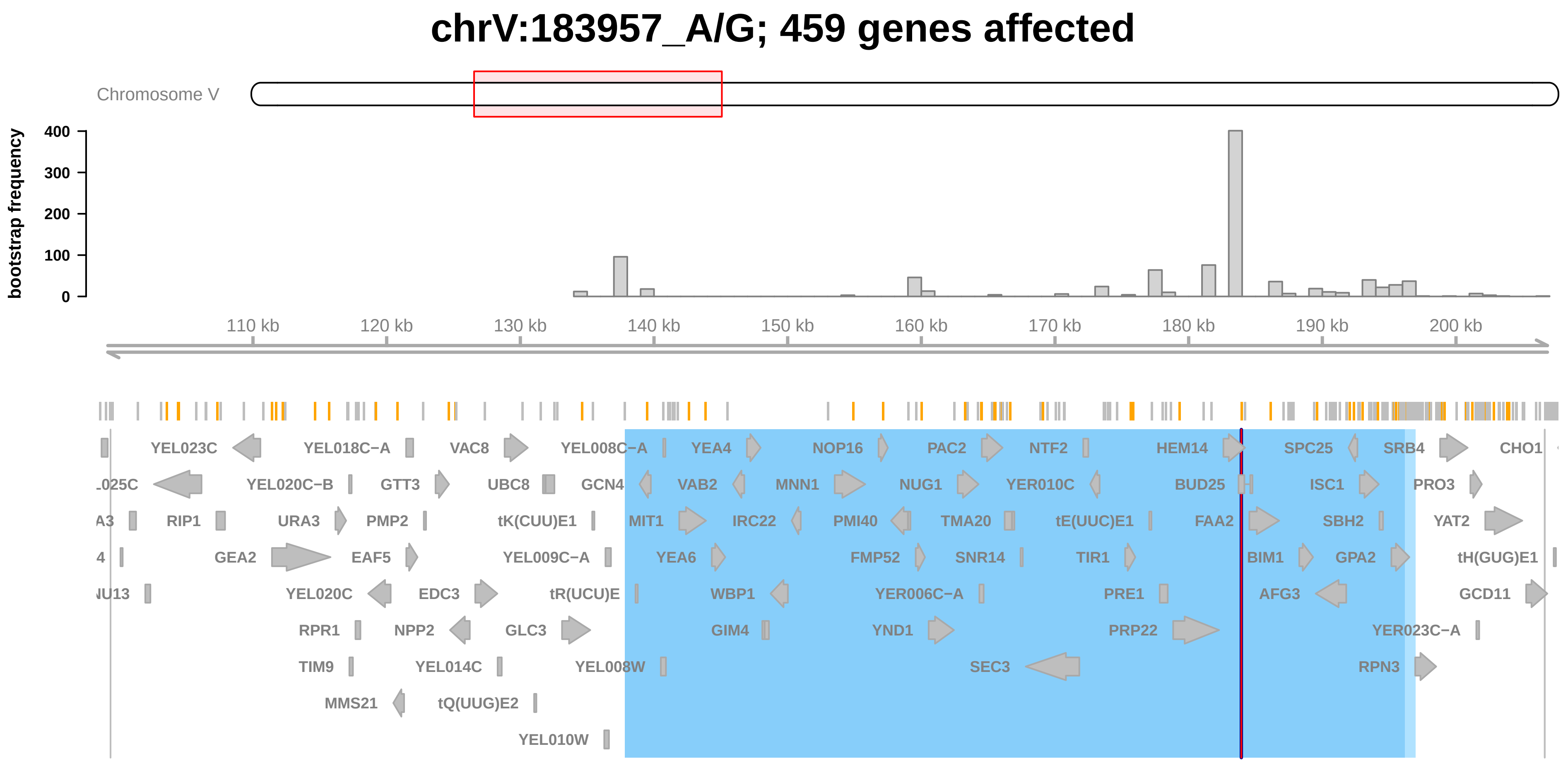
chrV:183957_A/G; 459 genes affected
Chromosome V
bootstrap frequency
400
300
200
100
0
110 kb
120 kb
130 kb
140 kb
150 kb
160 kb
170 kb
180 kb
190 kb
200 kb
YEL023C
YEL018C−A
VAC8
YEL008C−A
YEA4
NOP16
PAC2
NTF2
HEM14
SPC25
SRB4
CHO1
YEL020C−B
GTT3
UBC8
GCN4
VAB2
MNN1
NUG1
YER010C
BUD25
ISC1
PRO3
RIP1
URA3
PMP2
tK(CUU)E1
MIT1
IRC22
PMI40
TMA20
tE(UUC)E1
FAA2
SBH2
YAT2
GEA2
EAF5
YEL009C−A
YEA6
FMP52
SNR14
TIR1
BIM1
GPA2
tH(GUG)E1
YEL020C
EDC3
tR(UCU)E
WBP1
YER006C−A
PRE1
AFG3
GCD11
RPR1
NPP2
GLC3
GIM4
YND1
PRP22
YER023C−A
TIM9
YEL014C
YEL008W
SEC3
RPN3
MMS21
tQ(UUG)E2
YEL010W

chrV:418504_A/G; 160 genes affected

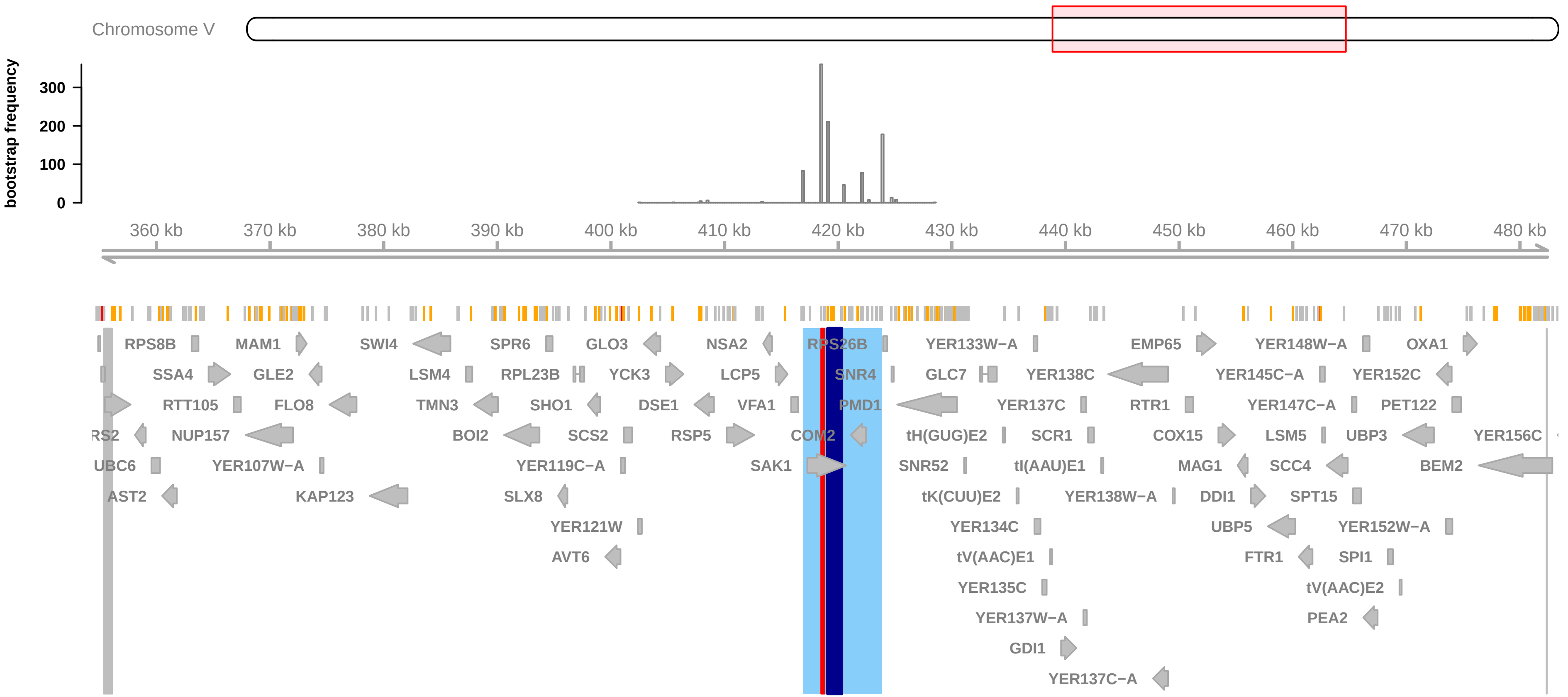

chrV:524625_A/T; 208 genes affected

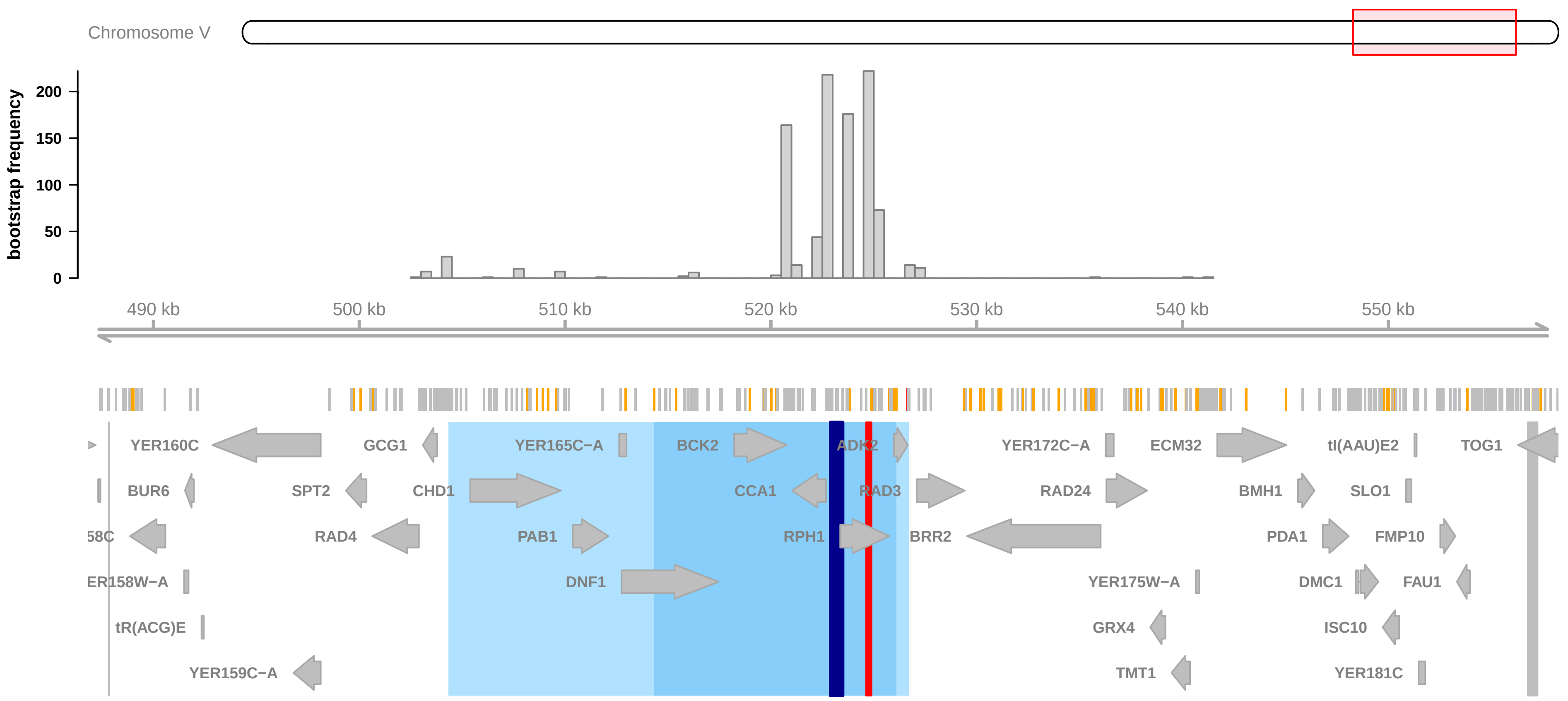

chrVI:71959_C/T; 160 genes affected

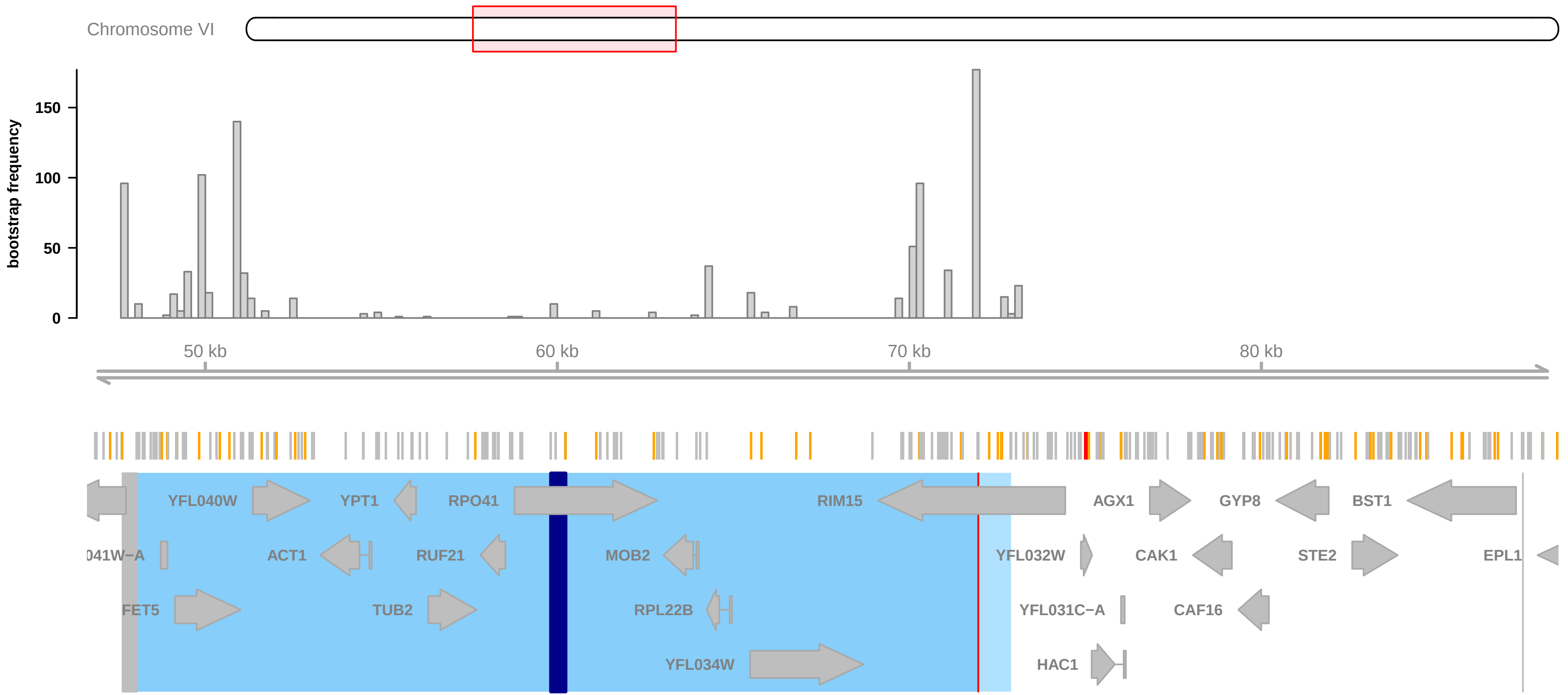

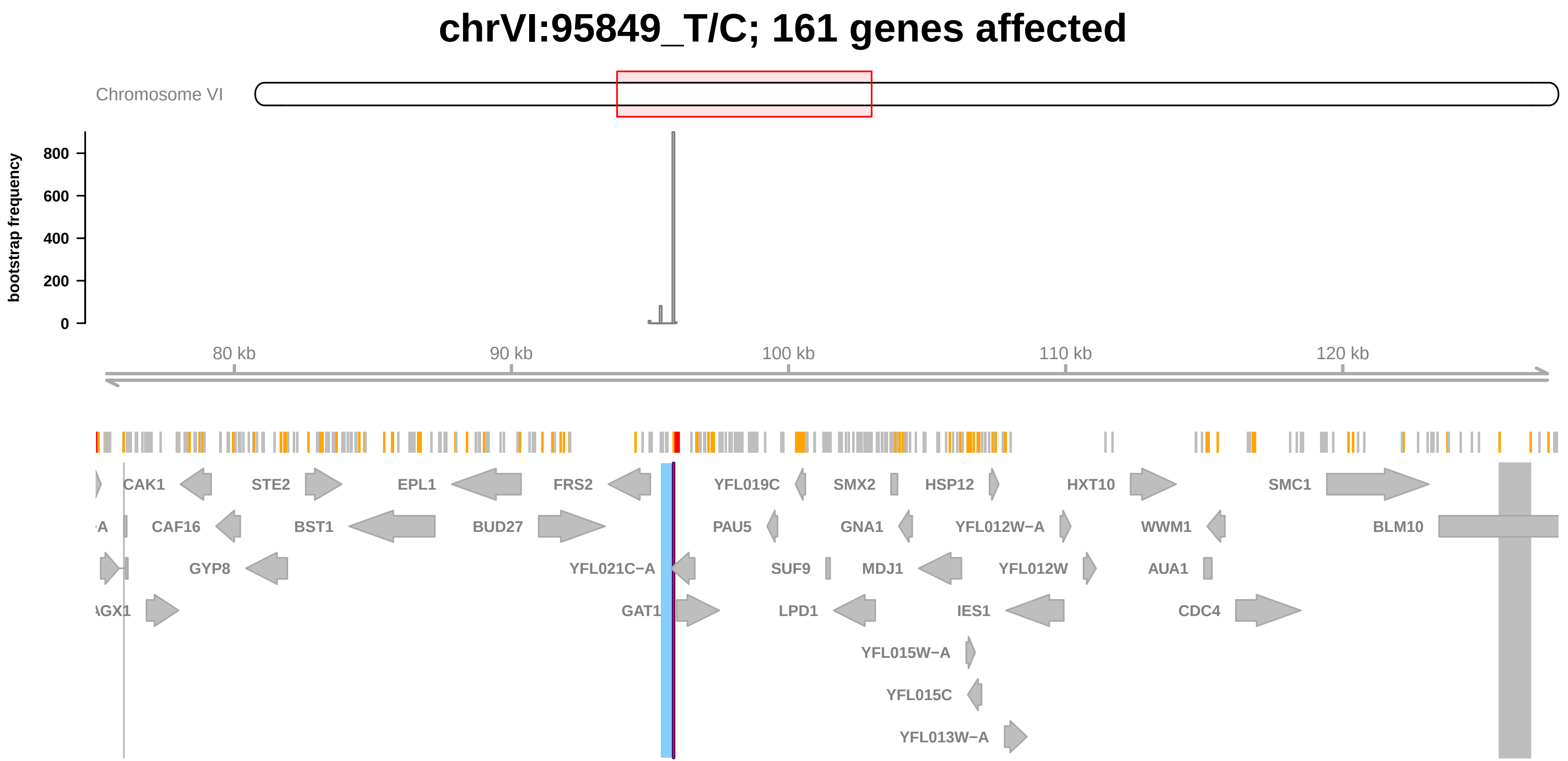
chrVI:95849_T/C; 161 genes affected
Chromosome VI
bootstrap frequency
800
600
400
200
0
80 kb
90 kb
100 kb
110 kb
120 kb
CAK1
STE2
EPL1
FRS2
YFL019C
SMX2
HSP12
HXT10
SMC1
CAF16
BST1
BUD27
PAU5
GNA1
YFL012W−A
WWM1
BLM10
GYP8
YFL021C−A
SUF9
MDJ1
YFL012W
AUA1
GAT1
LPD1
IES1
CDC4
YFL015W−A
YFL015C
YFL013W−A

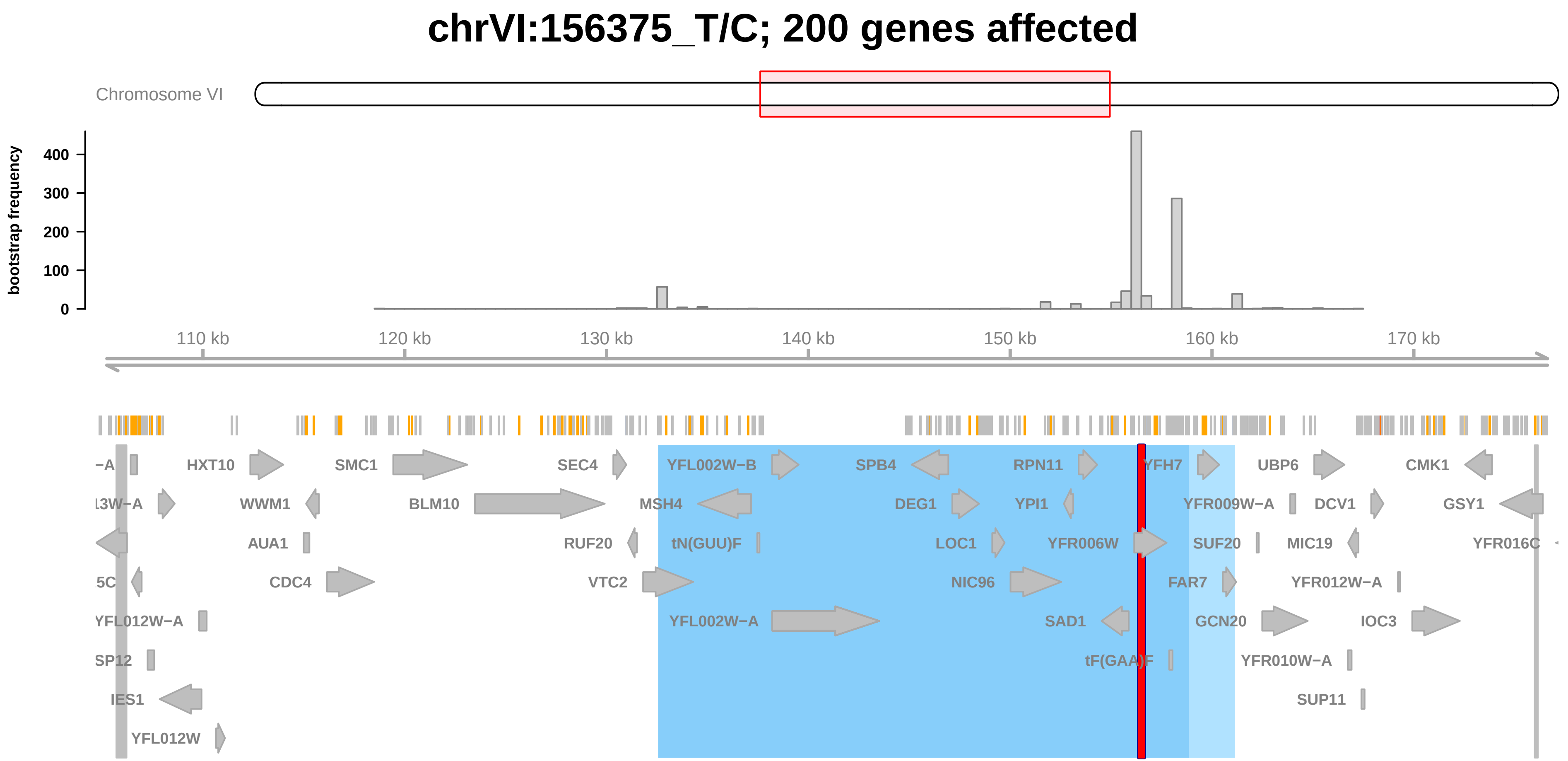
chrVI:156375_T/C; 200 genes affected
Chromosome VI
bootstrap frequency
400
300
200
100
0
110 kb
120 kb
130 kb
140 kb
150 kb
160 kb
170 kb
HXT10
SMC1
SEC4
YFL002W−B
SPB4
RPN11
YFH7
UBP6
CMK1
WWM1
BLM10
MSH4
DEG1
YPI1
YFR009W−A
DCV1
GSY1
AUA1
RUF20
tN(GUU)F
LOC1
YFR006W
SUF20
MIC19
YFR016C
CDC4
VTC2
NIC96
FAR7
YFR012W−A
YFL012W−A
YFL002W−A
SAD1
GCN20
IOC3
tF(GAA)F
YFR010W−A
IES1
SUP11
YFL012W

chrVI:229337_G/A; 73 genes affected

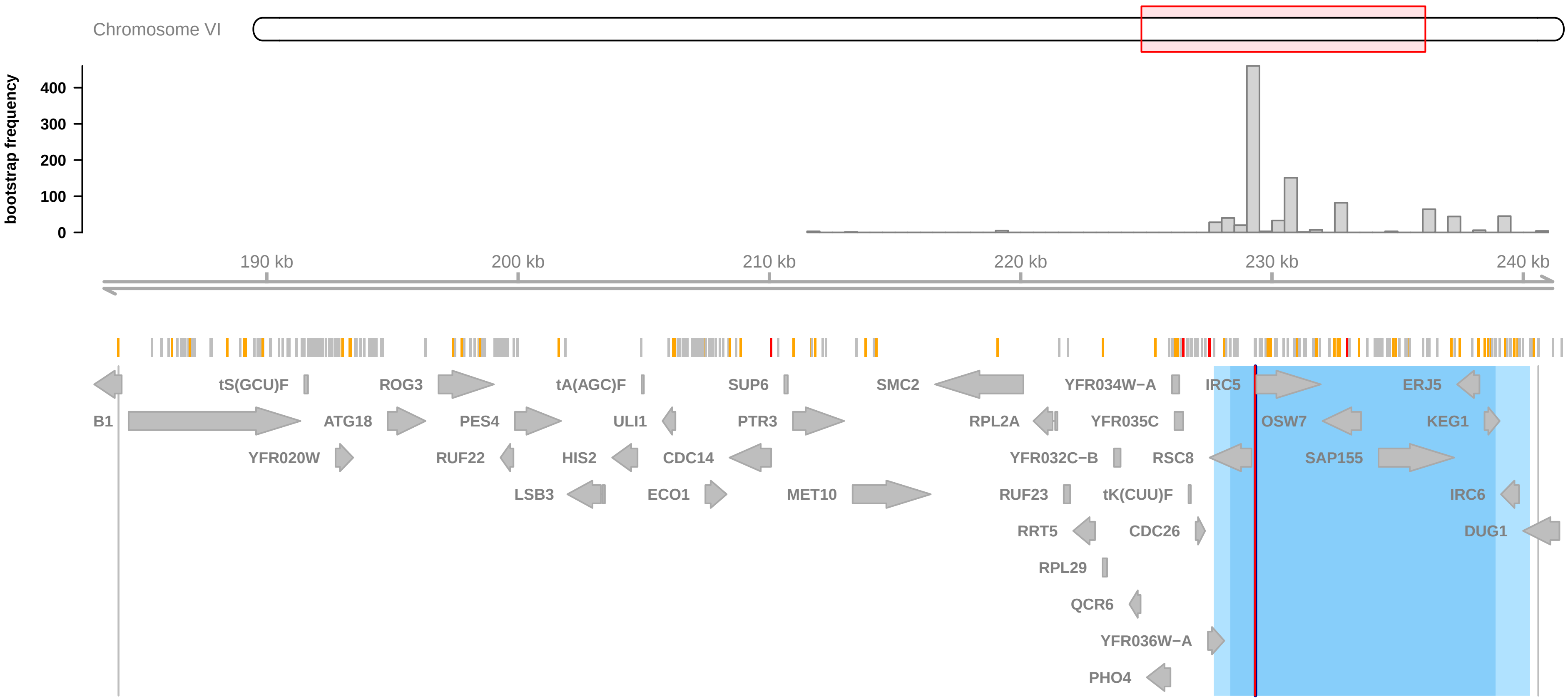

chrVII:122421_CA/C; 1581 genes affected

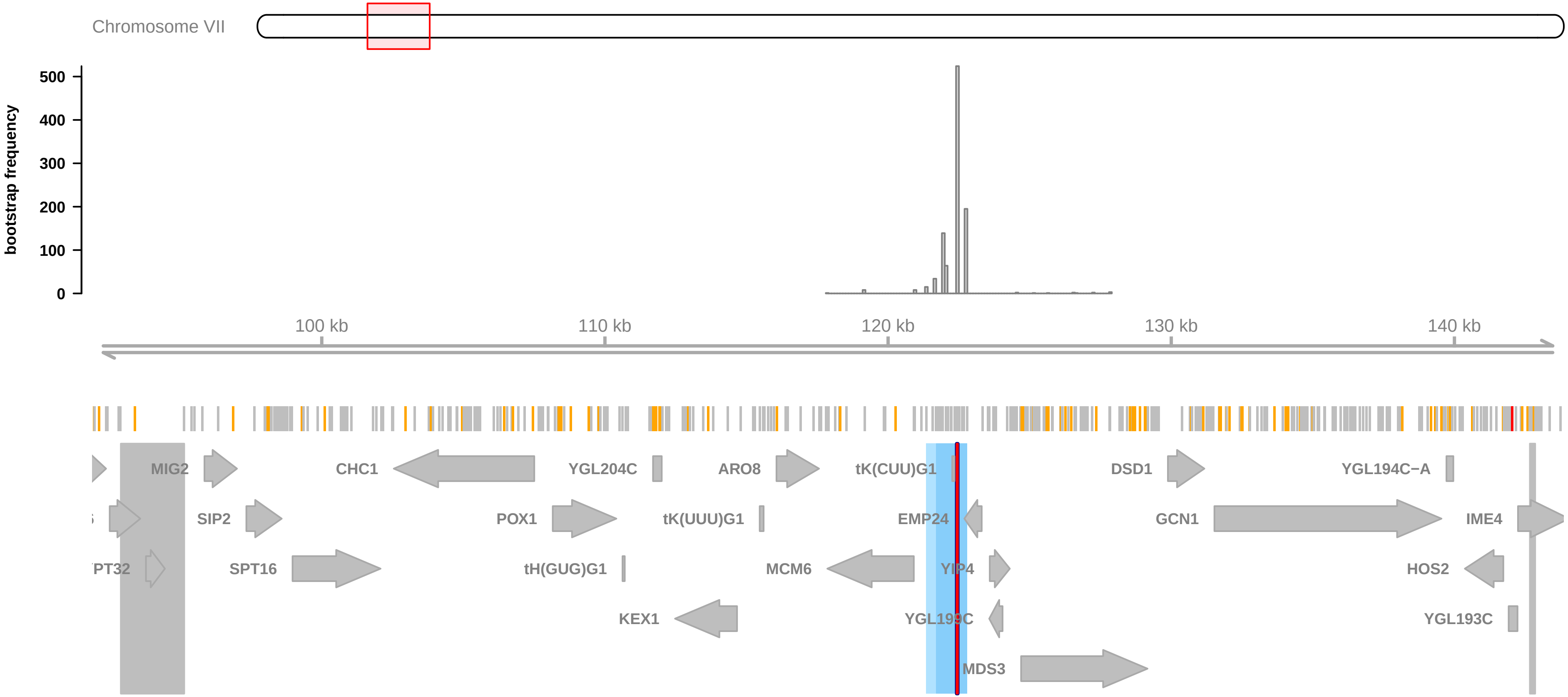

chrVII:141743_C/T; 1816 genes affected

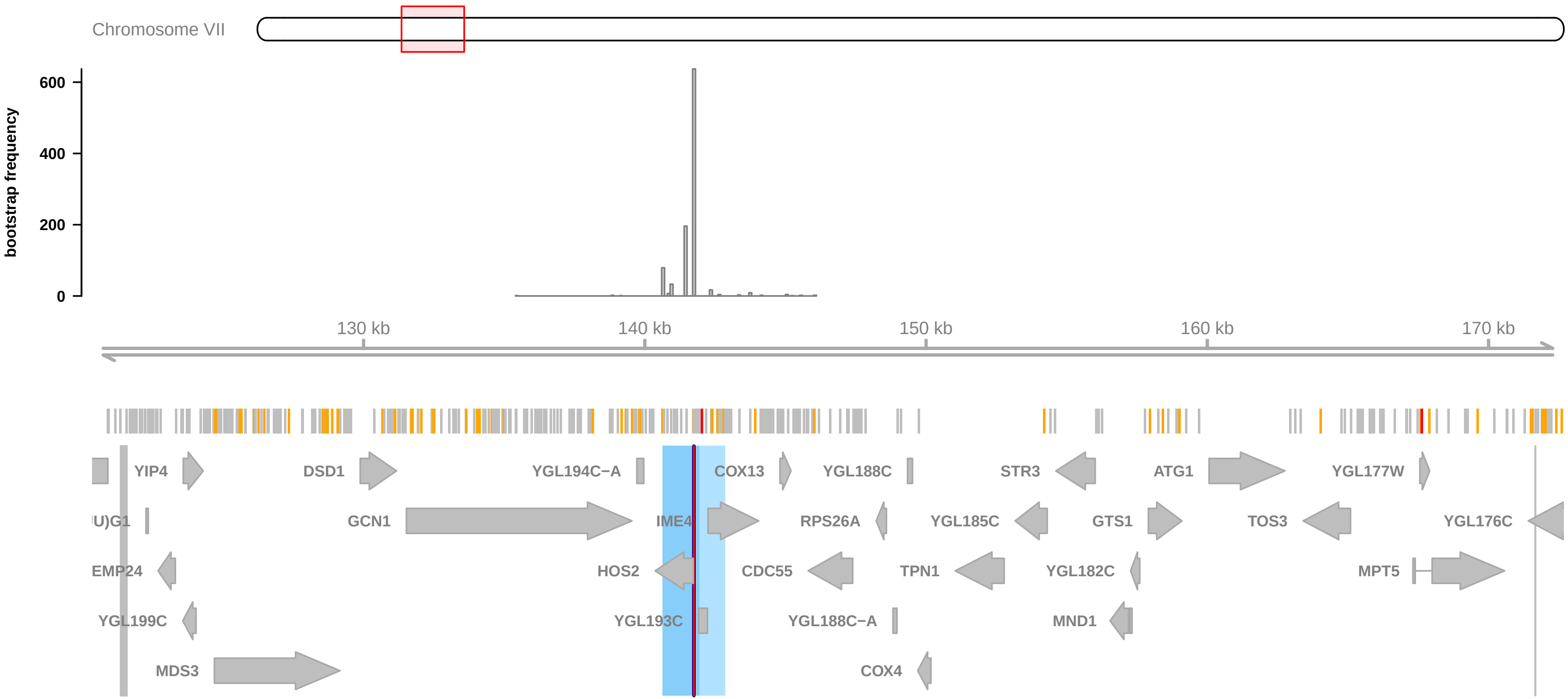

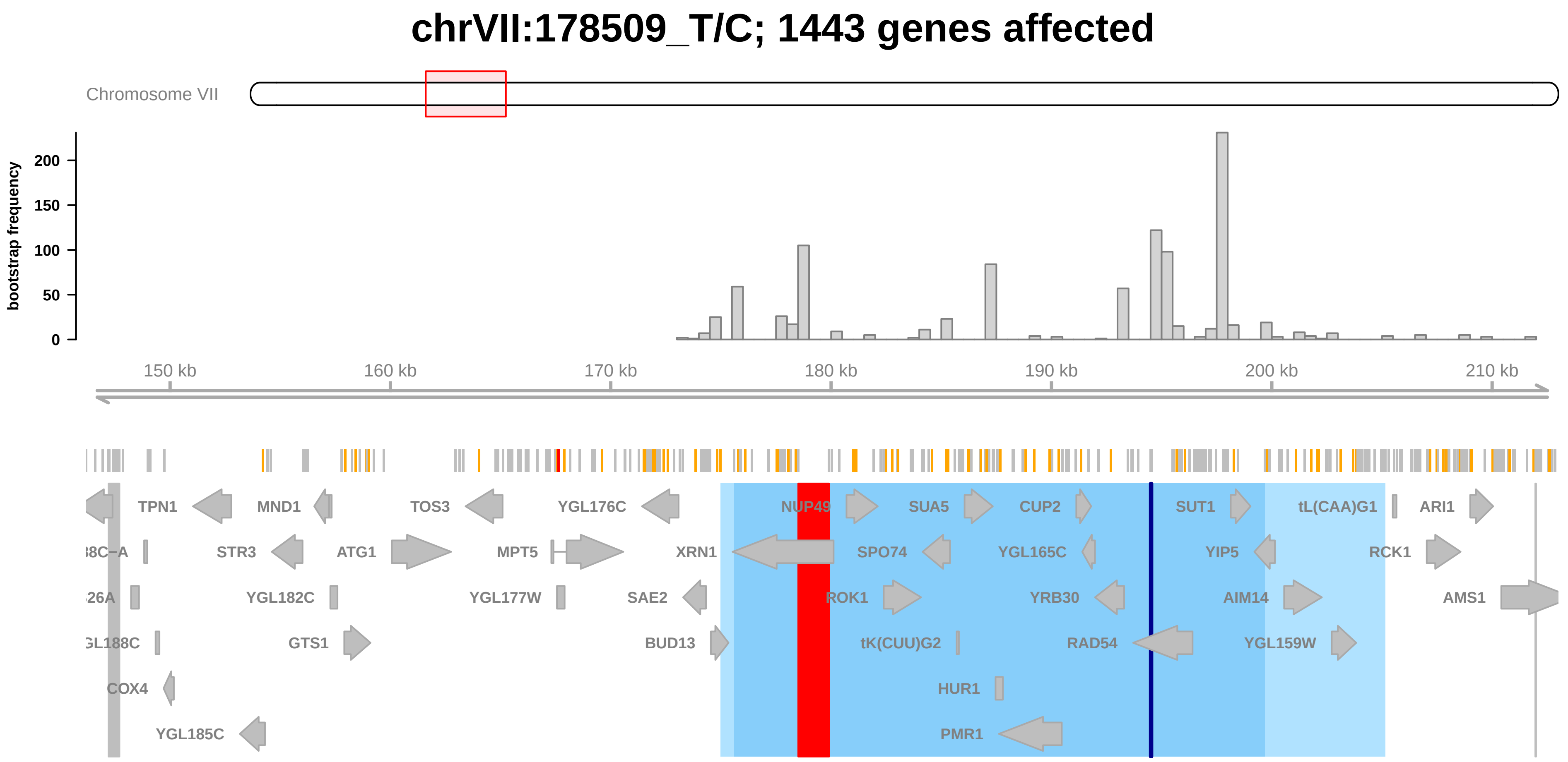
chrVII:178509_T/C; 1443 genes affected
Chromosome VII
bootstrap frequency
0
50
100
150
200
150 kb
160 kb
170 kb
180 kb
190 kb
200 kb
210 kb
TPN1
MND1
TOS3
YGL176C
NUP49
SUA5
CUP2
SUT1
tL(CAA)G1
ARI1
STR3
ATG1
MPT5
XRN1
SPO74
YGL165C
YIP5
RCK1
YGL182C
YGL177W
SAE2
ROK1
YRB30
AIM14
AMS1
GTS1
BUD13
tK(CUU)G2
RAD54
YGL159W
COX4
HUR1
YGL185C
PMR1

chrVII:372033_G/C; 1031 genes affected

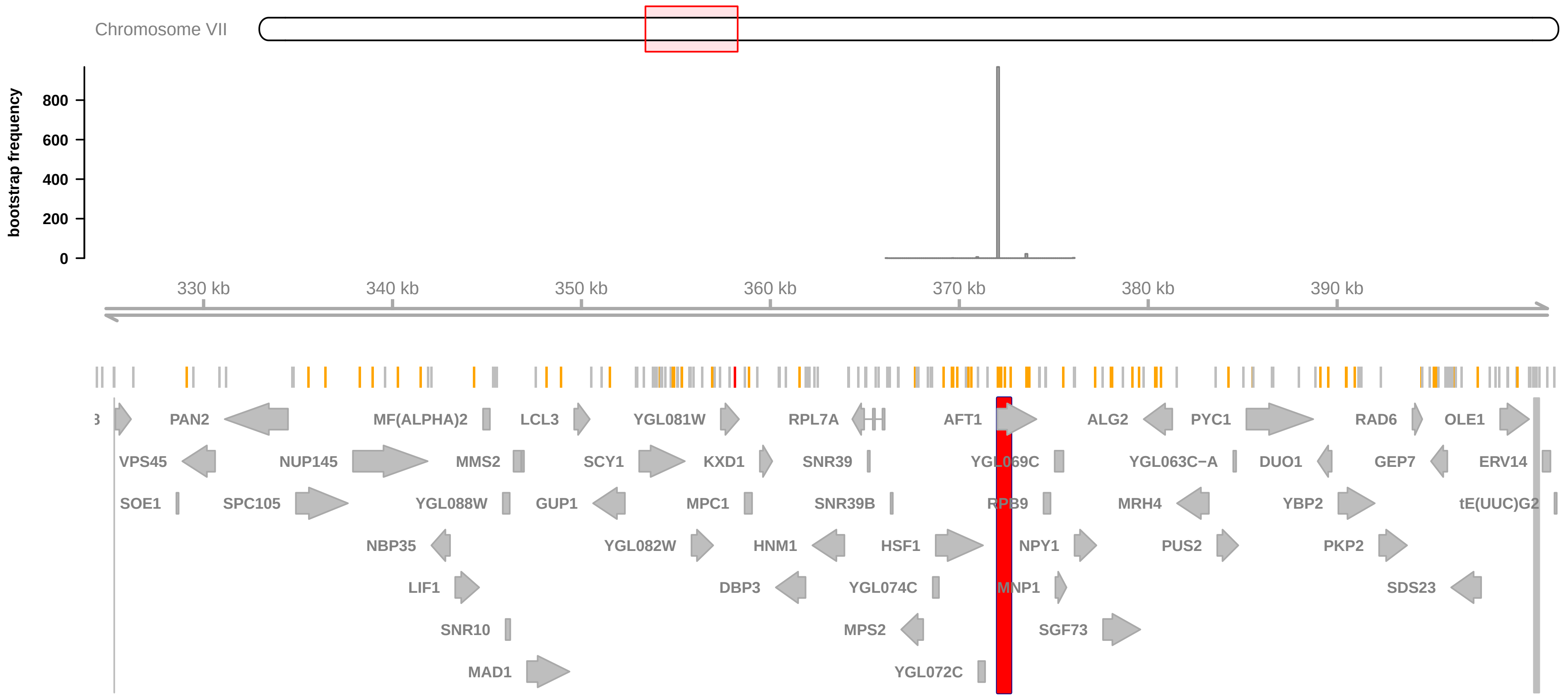

chrVII:397449_G/A; 977 genes affected

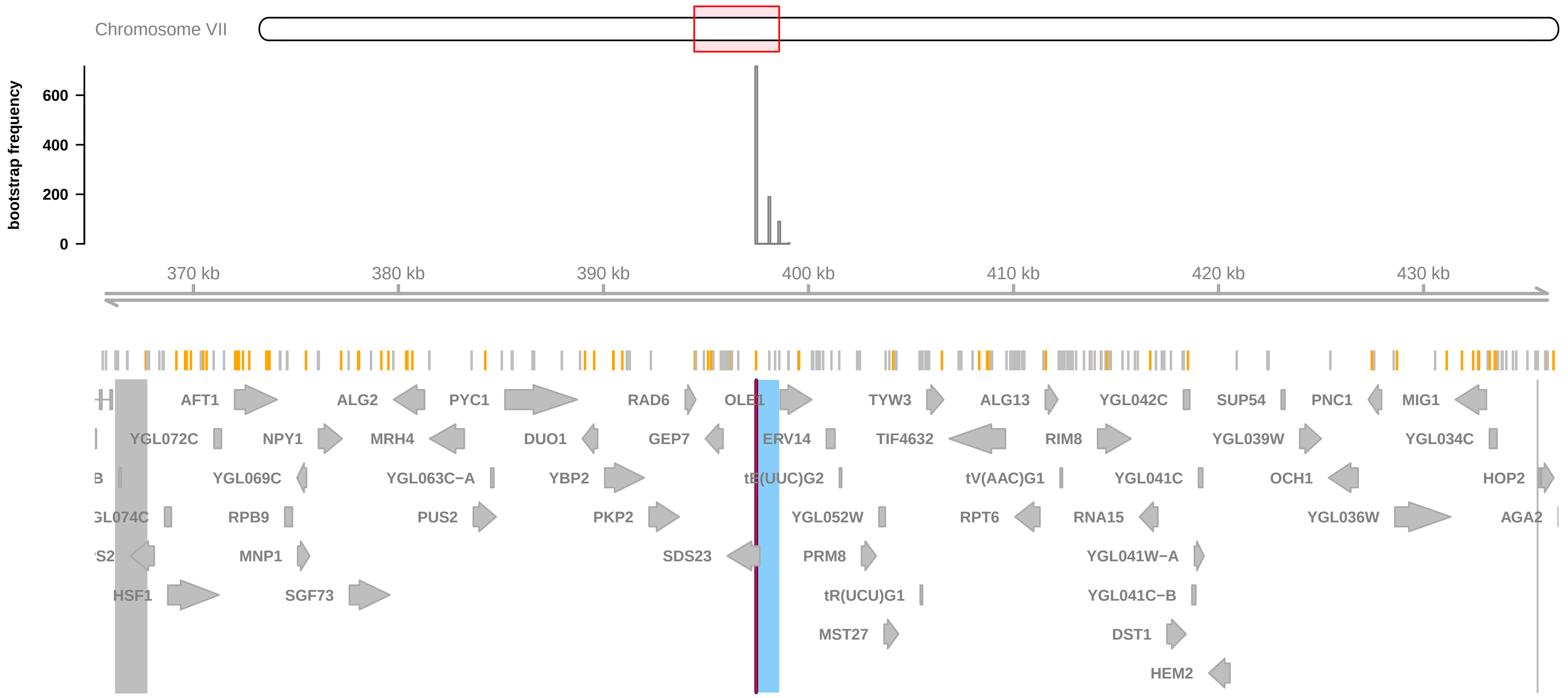

chrVII:470028_A/G; 1450 genes affected

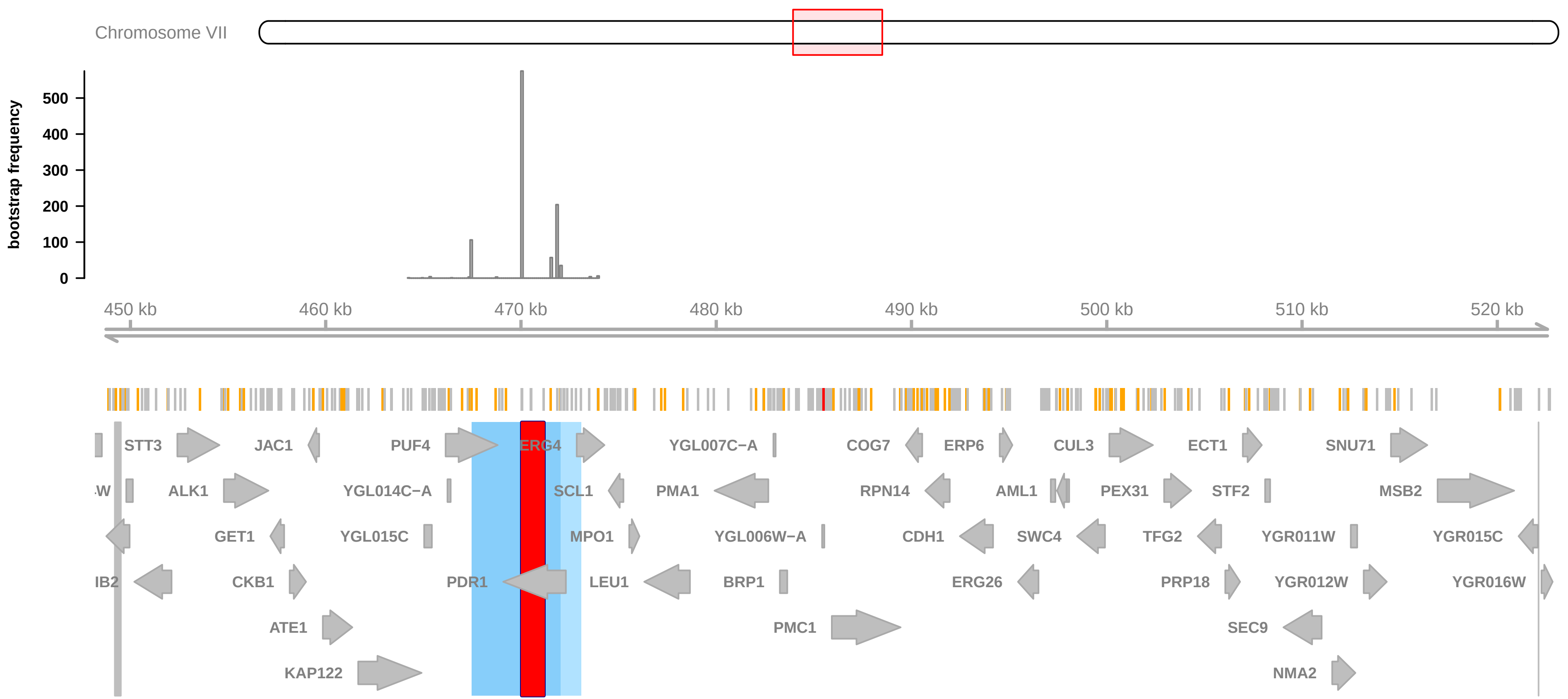

chrVII:656248_T/G; 682 genes affected

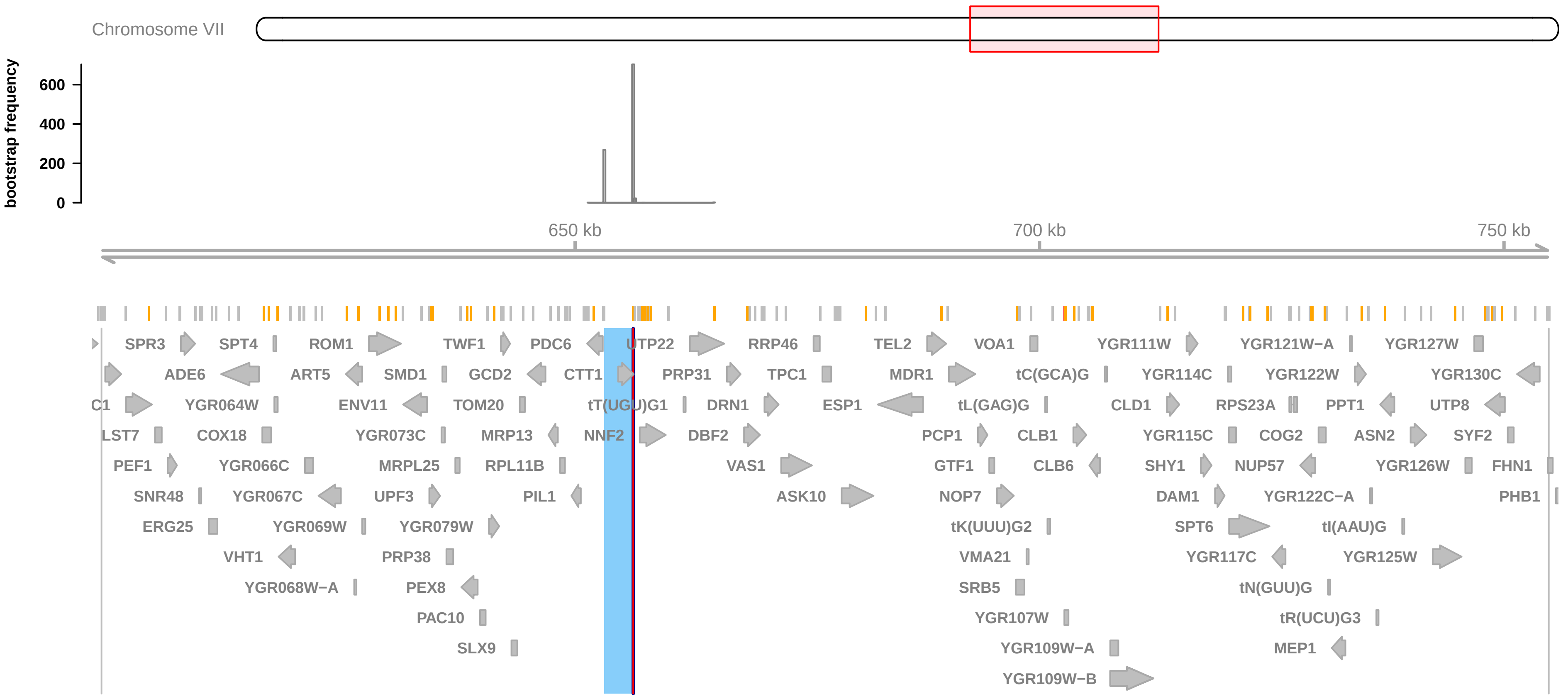

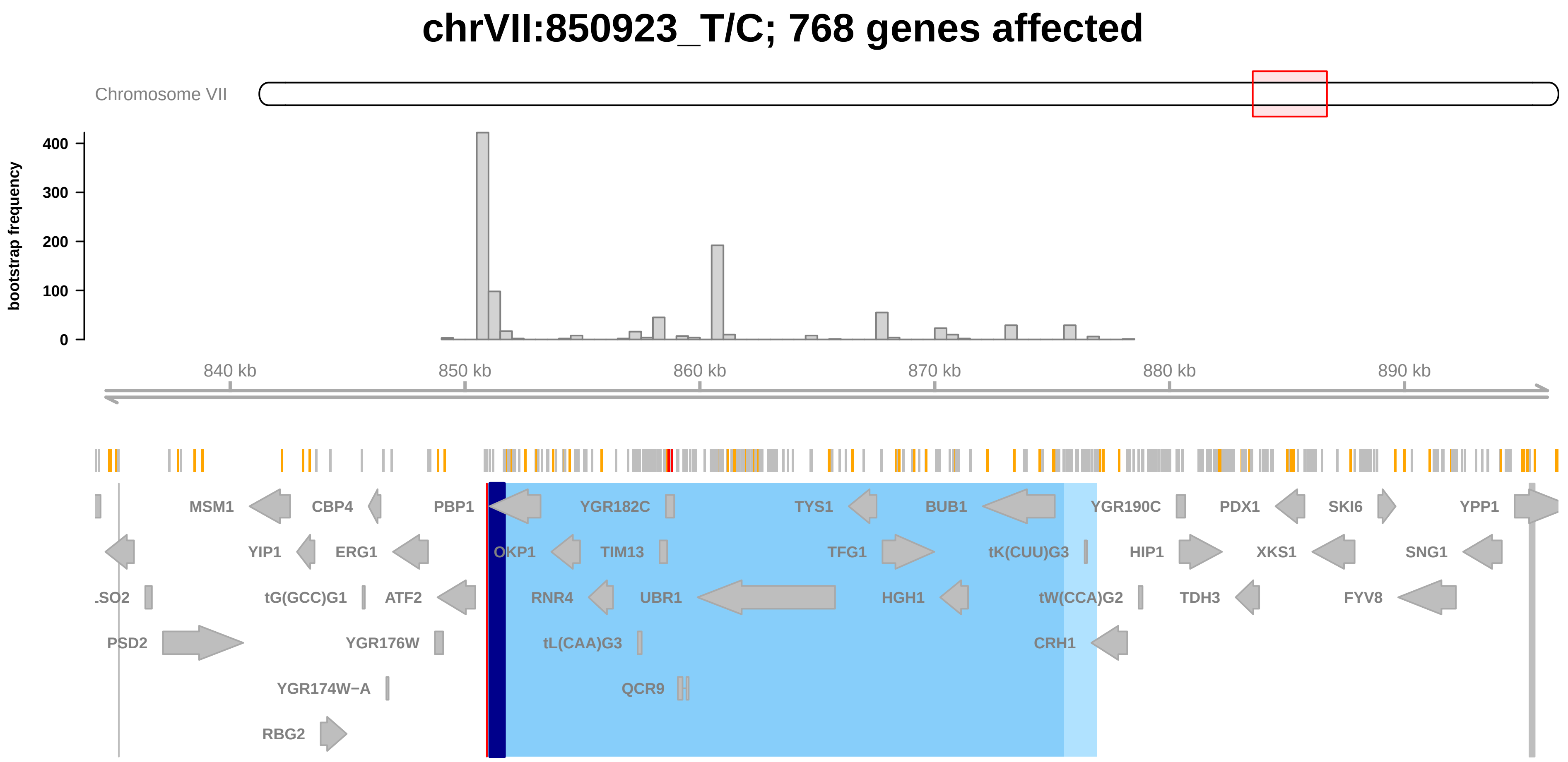
chrVII:850923_T/C; 768 genes affected
Chromosome VII
bootstrap frequency
400
300
200
100
0
840 kb
850 kb
860 kb
870 kb
880 kb
890 kb
MSM1
CBP4
PBP1
YGR182C
TYS1
BUB1
YGR190C
PDX1
SKI6
YPP1
YIP1
ERG1
OKP1
TIM13
TFG1
tK(CUU)G3
HIP1
XKS1
SNG1
tG(GCC)G1
ATF2
RNR4
UBR1
HGH1
tW(CCA)G2
TDH3
FYV8
PSD2
YGR176W
tL(CAA)G3
CRH1
YGR174W−A
QCR9
RBG2

chrVII:984362_C/T; 724 genes affected

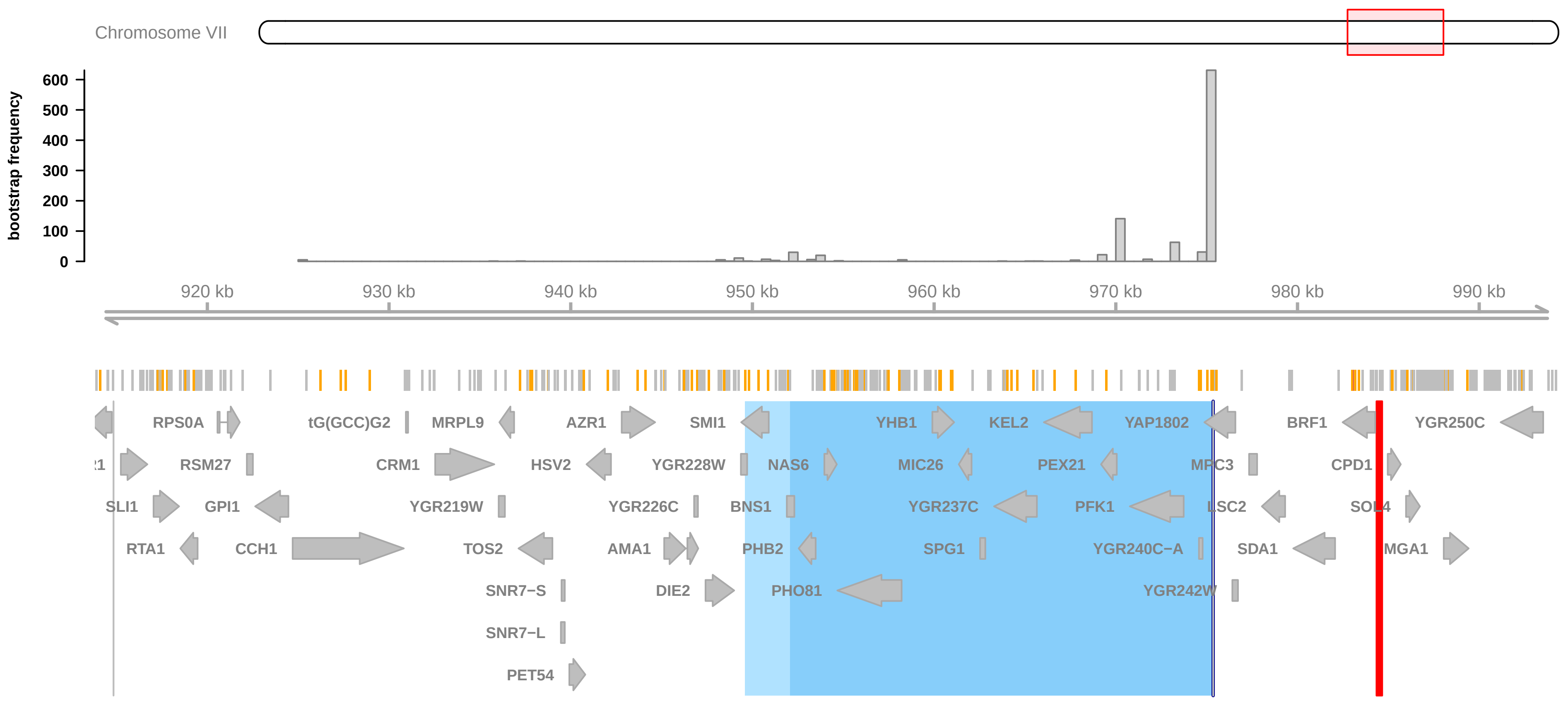

chrVIII:104423_C/A; 1029 genes affected

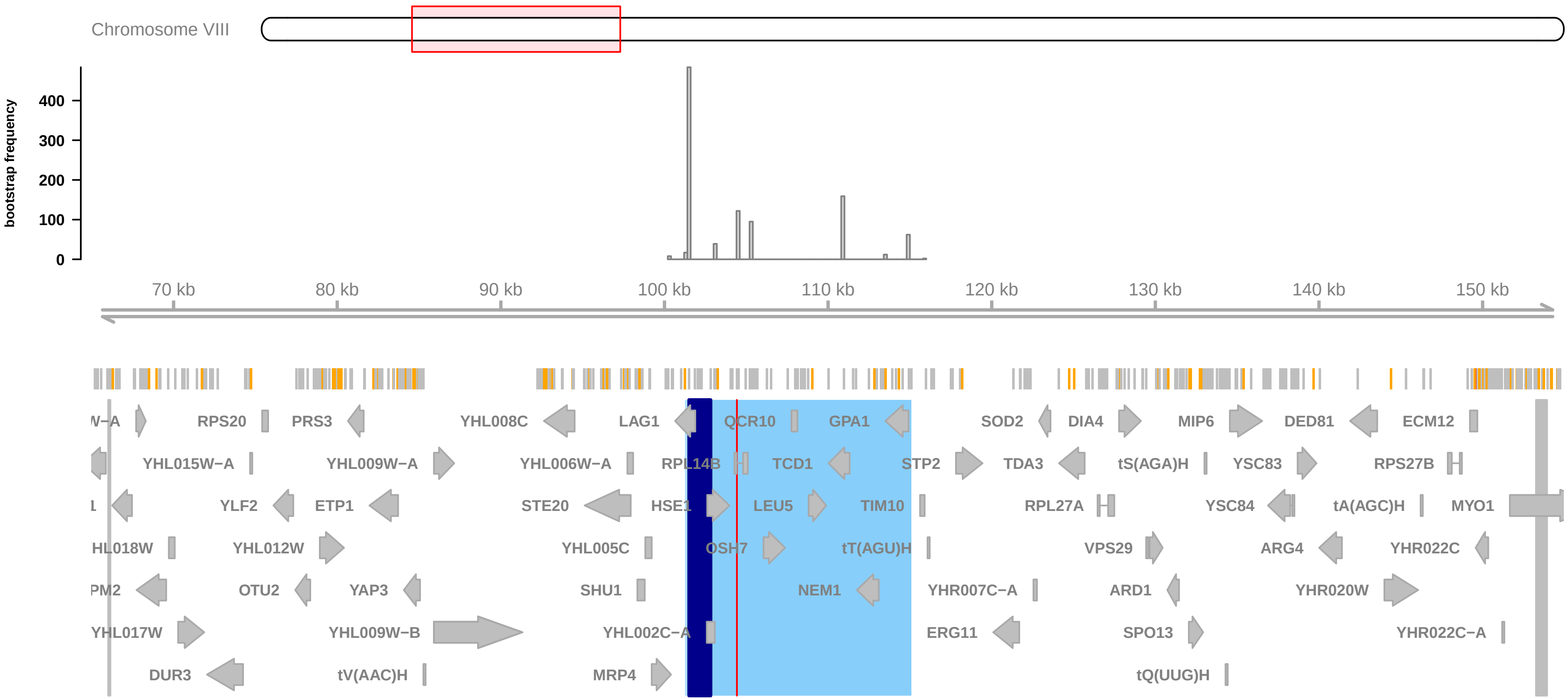

chrVIII:175012_C/T; 653 genes affected

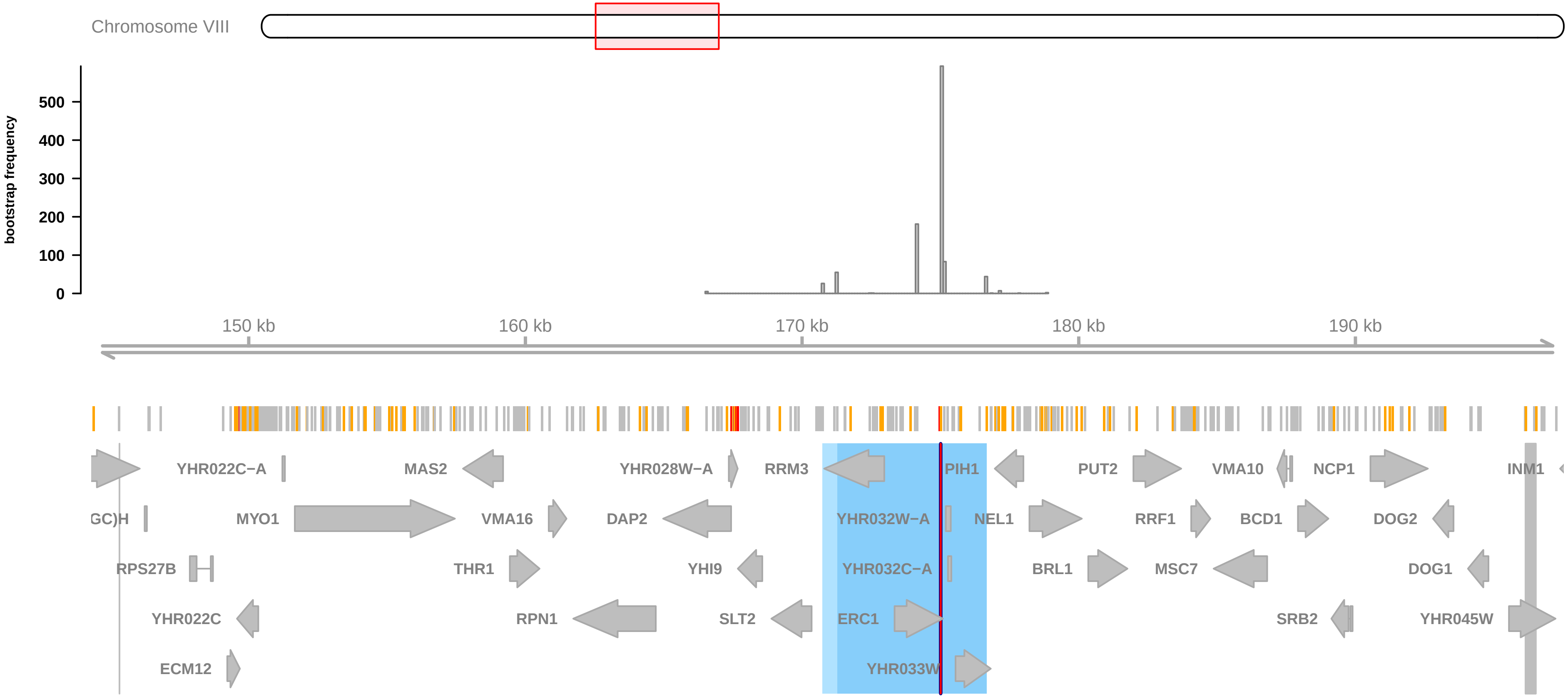

chrVIII:459310_C/G; 402 genes affected

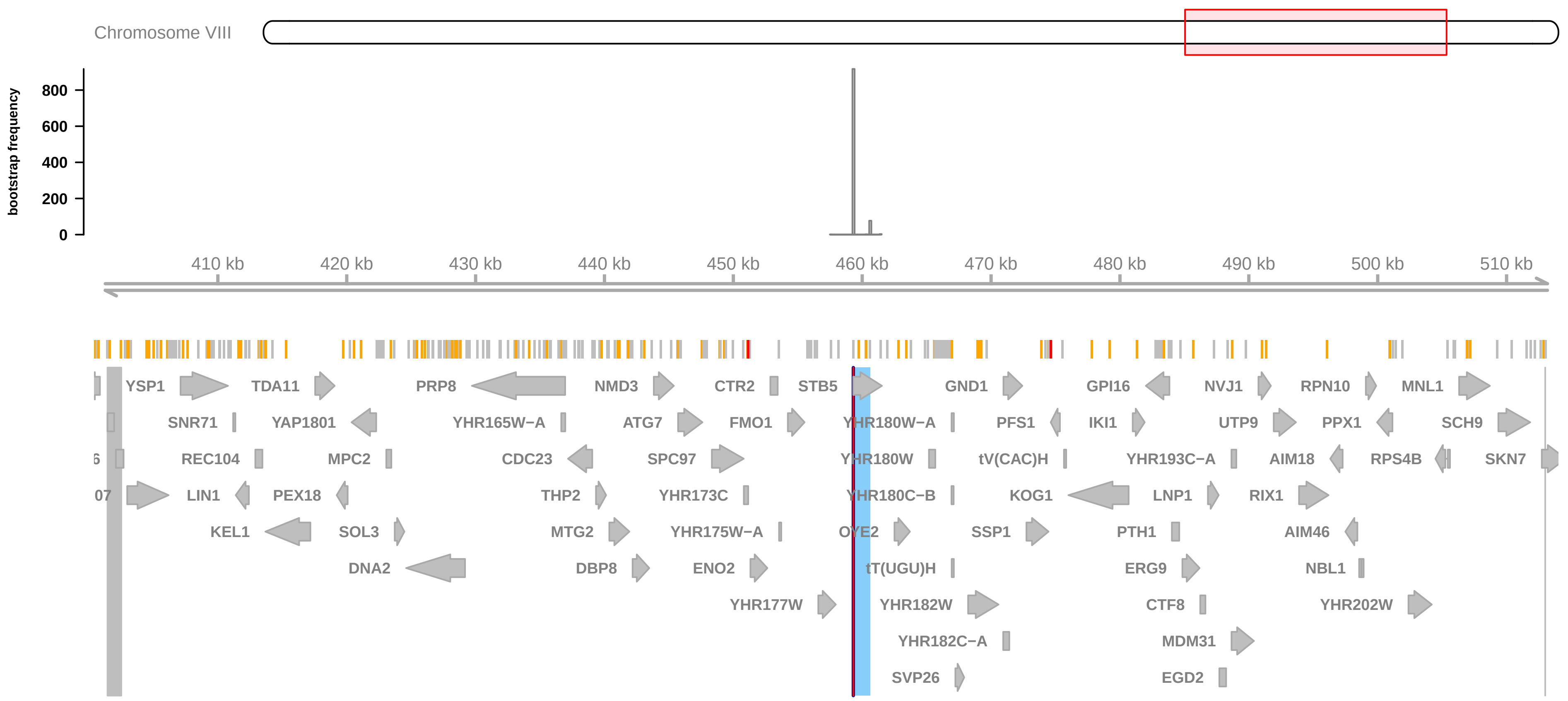

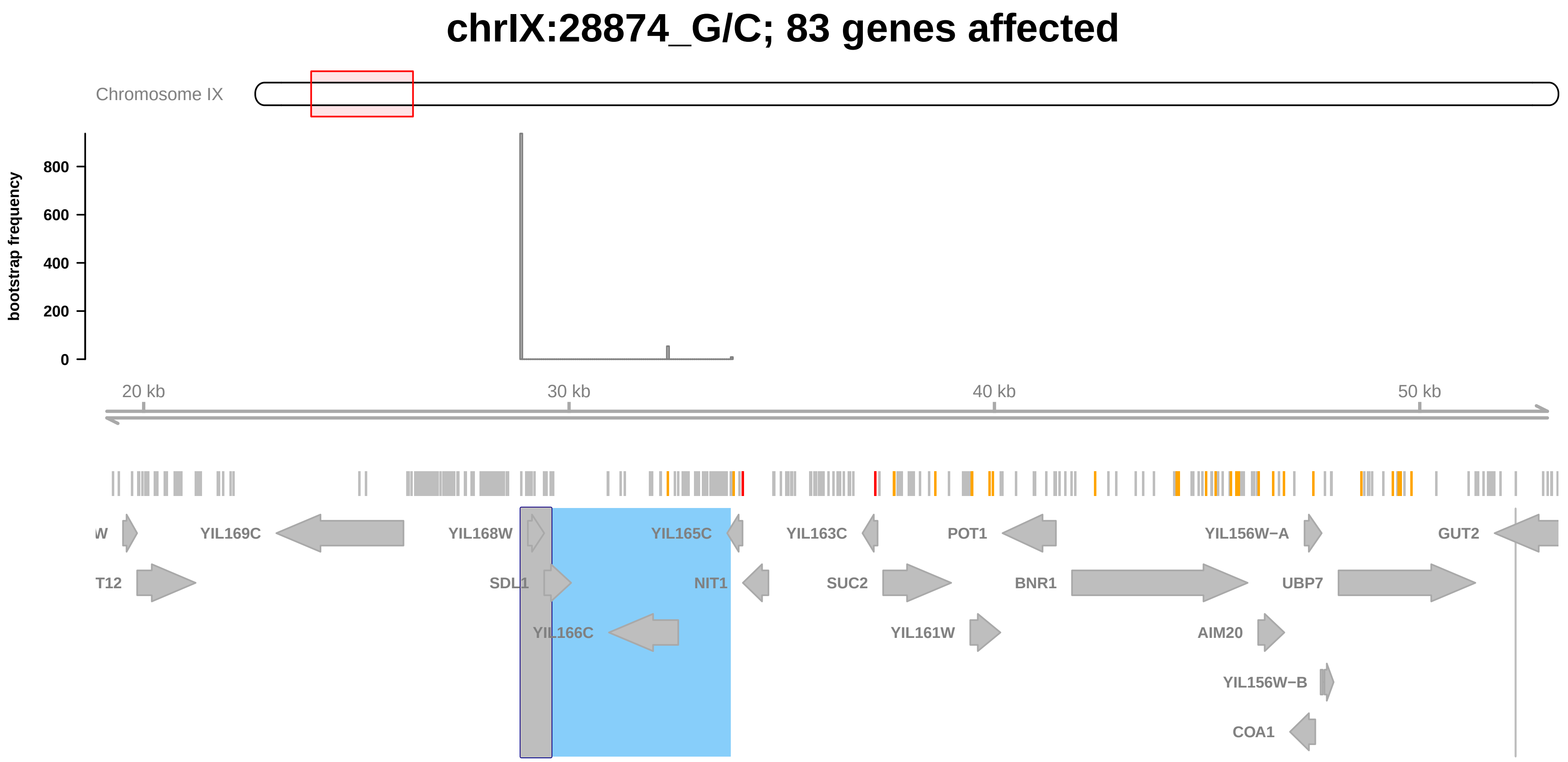
chrIX:28874_G/C; 83 genes affected
Chromosome IX
bootstrap frequency
800
600
400
200
0
20 kb
30 kb
40 kb
50 kb
W
YIL169C
YIL168W
YIL165C
YIL163C
POT1
YIL156W−A
GUT2
T12
SDL1
NIT1
SUC2
BNR1
UBP7
YIL166C
YIL161W
AIM20
YIL156W−B
COA1

chrIX:105090_T/C; 592 genes affected

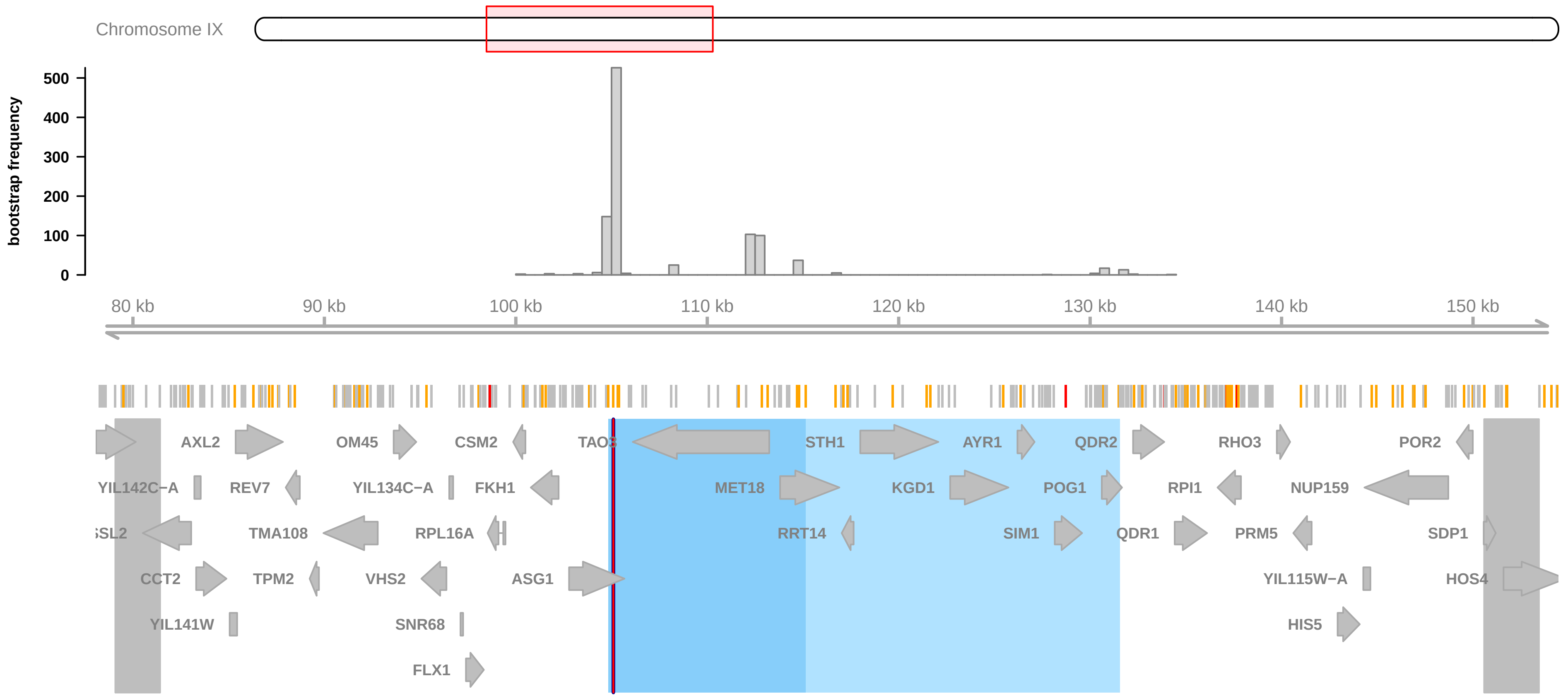

chrIX:178160_C/T; 170 genes affected

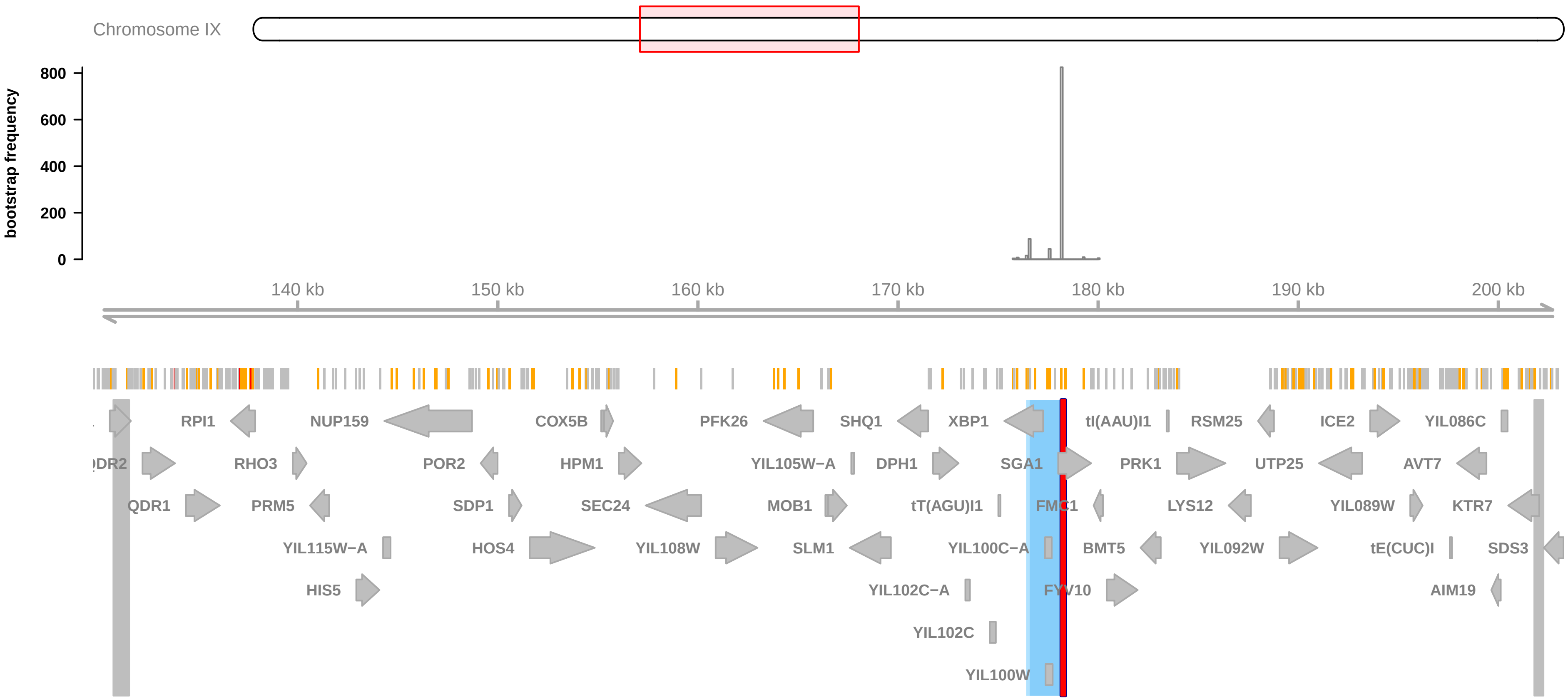

chrIX:227301_A/T; 151 genes affected

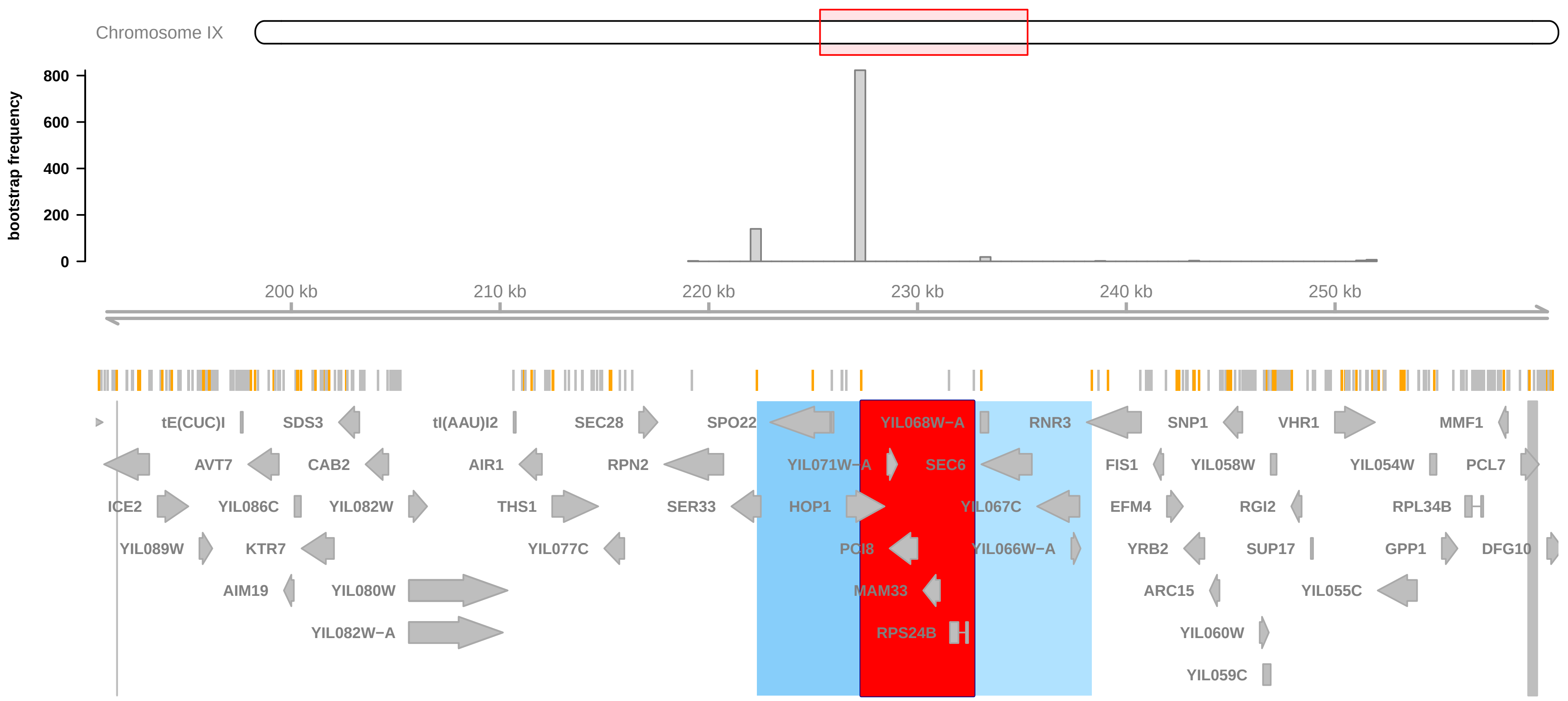

chrIX:386051_A/G; 79 genes affected

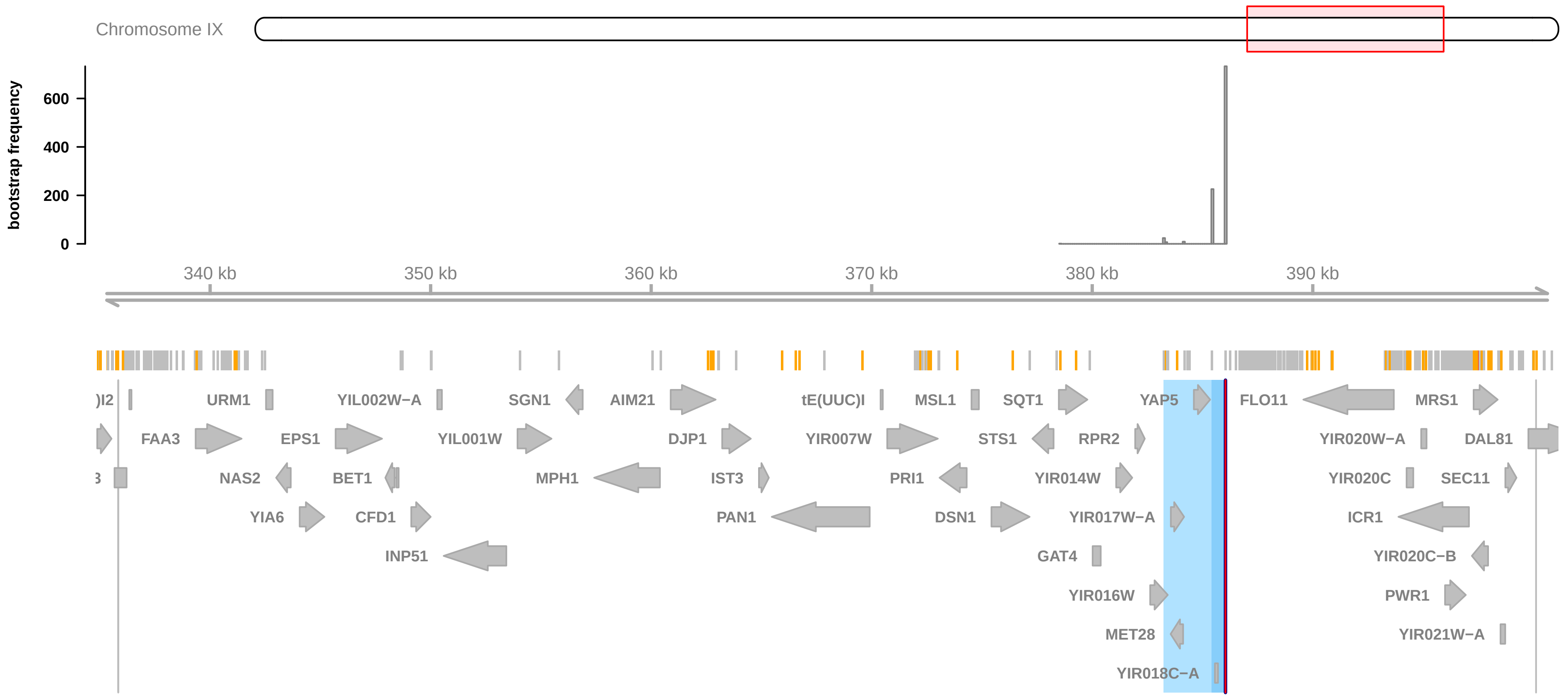

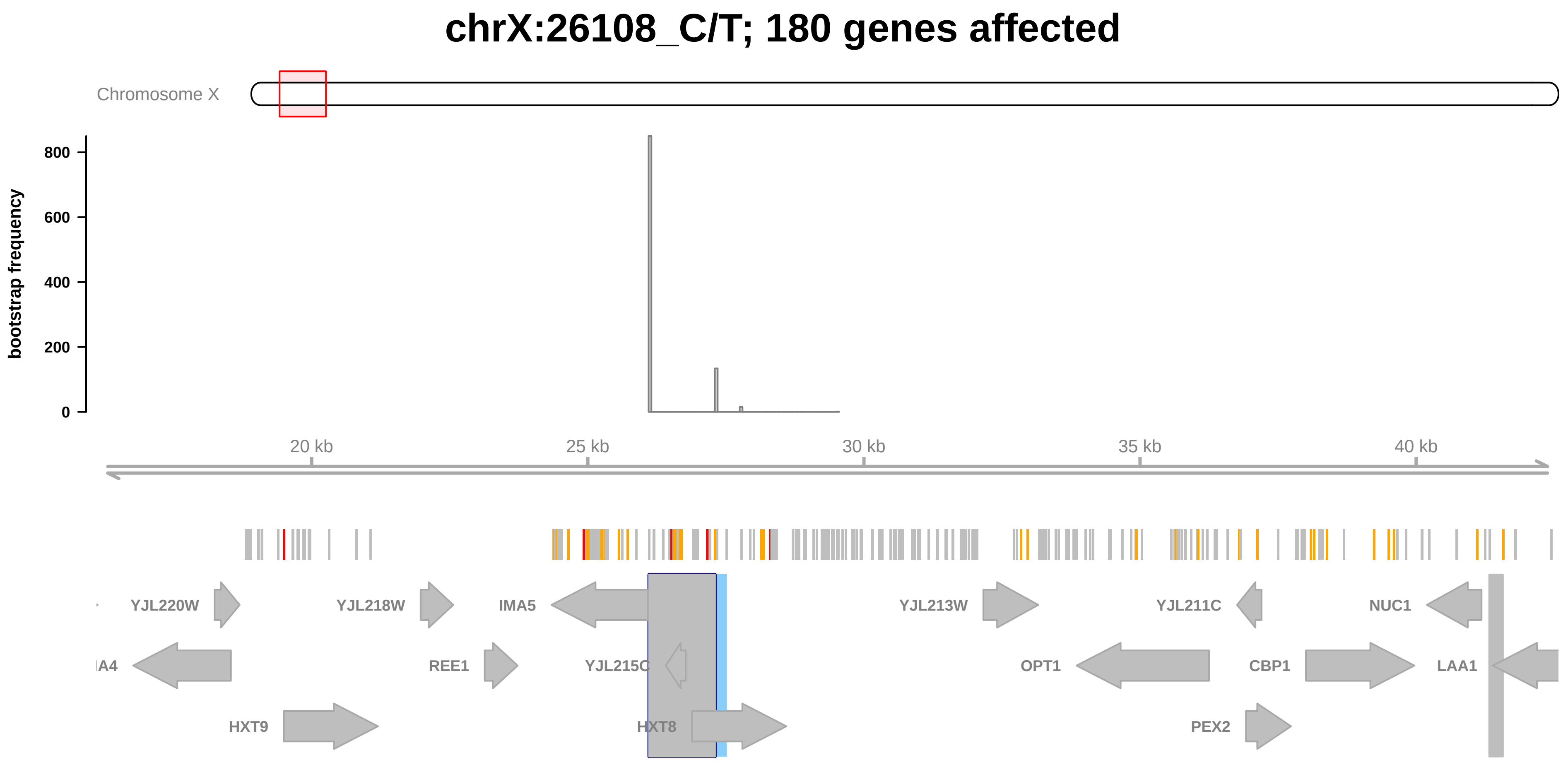
chrX:26108_C/T; 180 genes affected
Chromosome X
bootstrap frequency
800
600
400
200
0
20 kb
25 kb
30 kb
35 kb
40 kb
YJL220W
YJL218W
IMA5
YJL213W
YJL211C
NUC1
A4
REE1
YJL215C
OPT1
CBP1
LAA1
HXT9
HXT8
PEX2

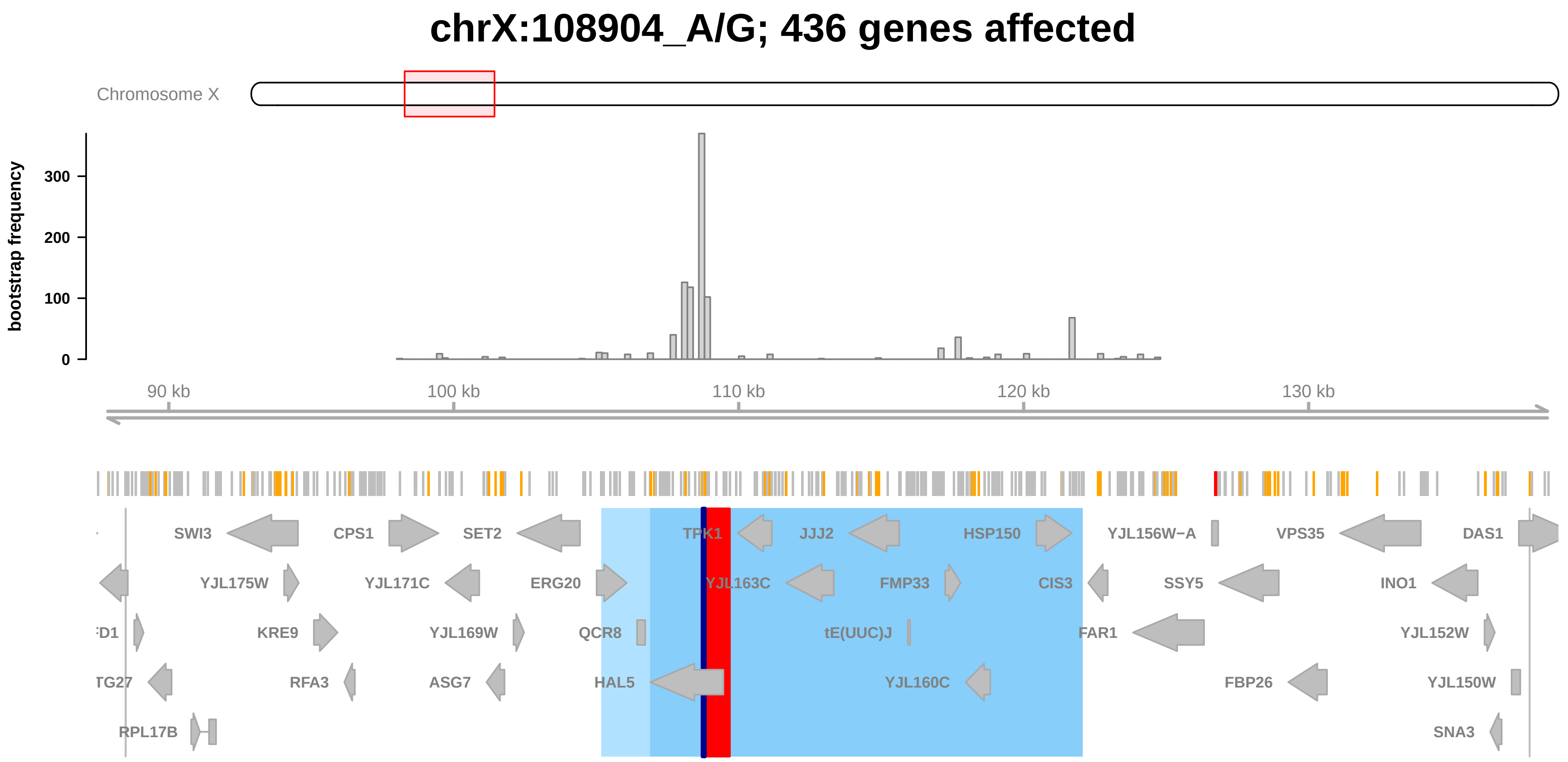
chrX:108904_A/G; 436 genes affected
Chromosome X
bootstrap frequency
0
100
200
300
90 kb
100 kb
110 kb
120 kb
130 kb
SWI3
CPS1
SET2
TPK1
JJJ2
HSP150
YJL156W−A
VPS35
DAS1
YJL175W
YJL171C
ERG20
YJL163C
FMP33
CIS3
SSY5
INO1
KRE9
YJL169W
QCR8
tE(UUC)J
FAR1
YJL152W
RFA3
ASG7
HAL5
YJL160C
FBP26
YJL150W
RPL17B
SNA3

chrX:181214_T/C; 411 genes affected

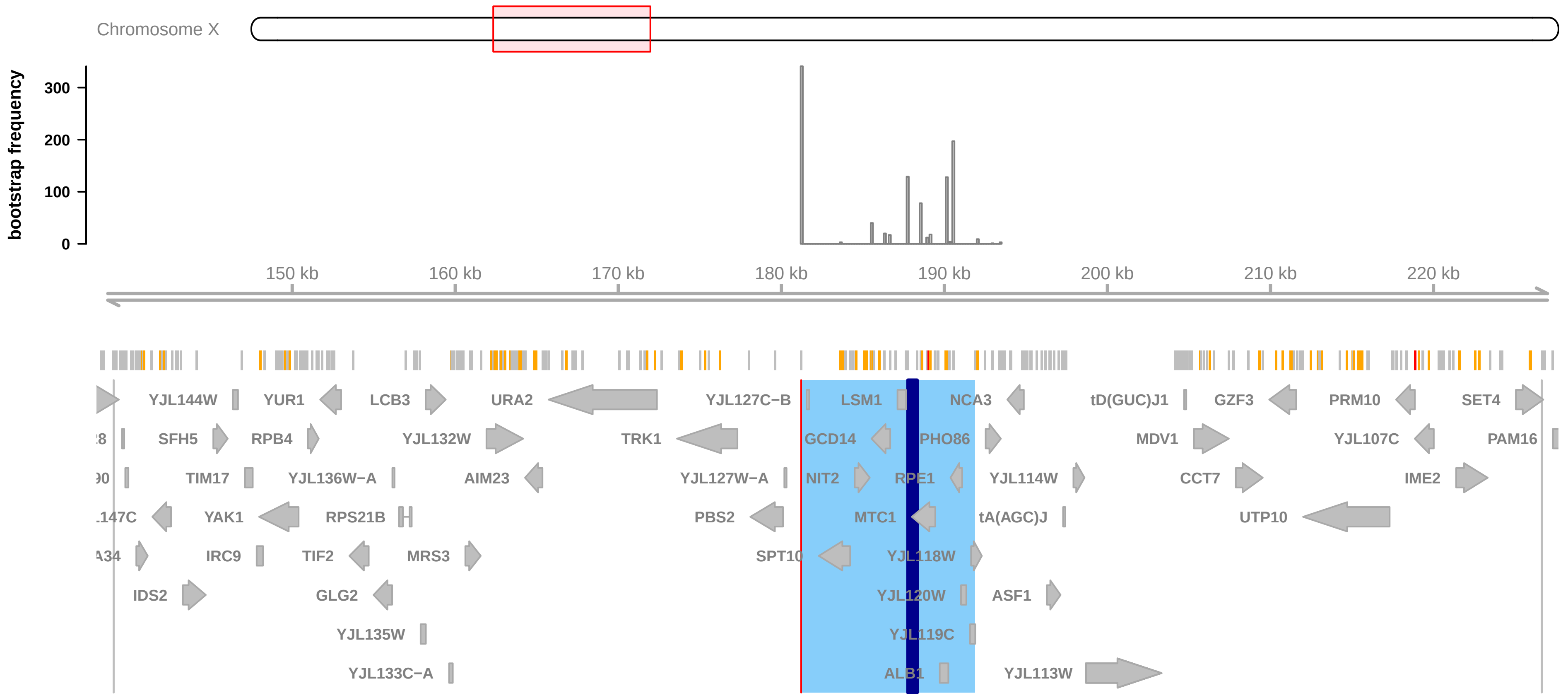

chrX:330144_C/T; 229 genes affected

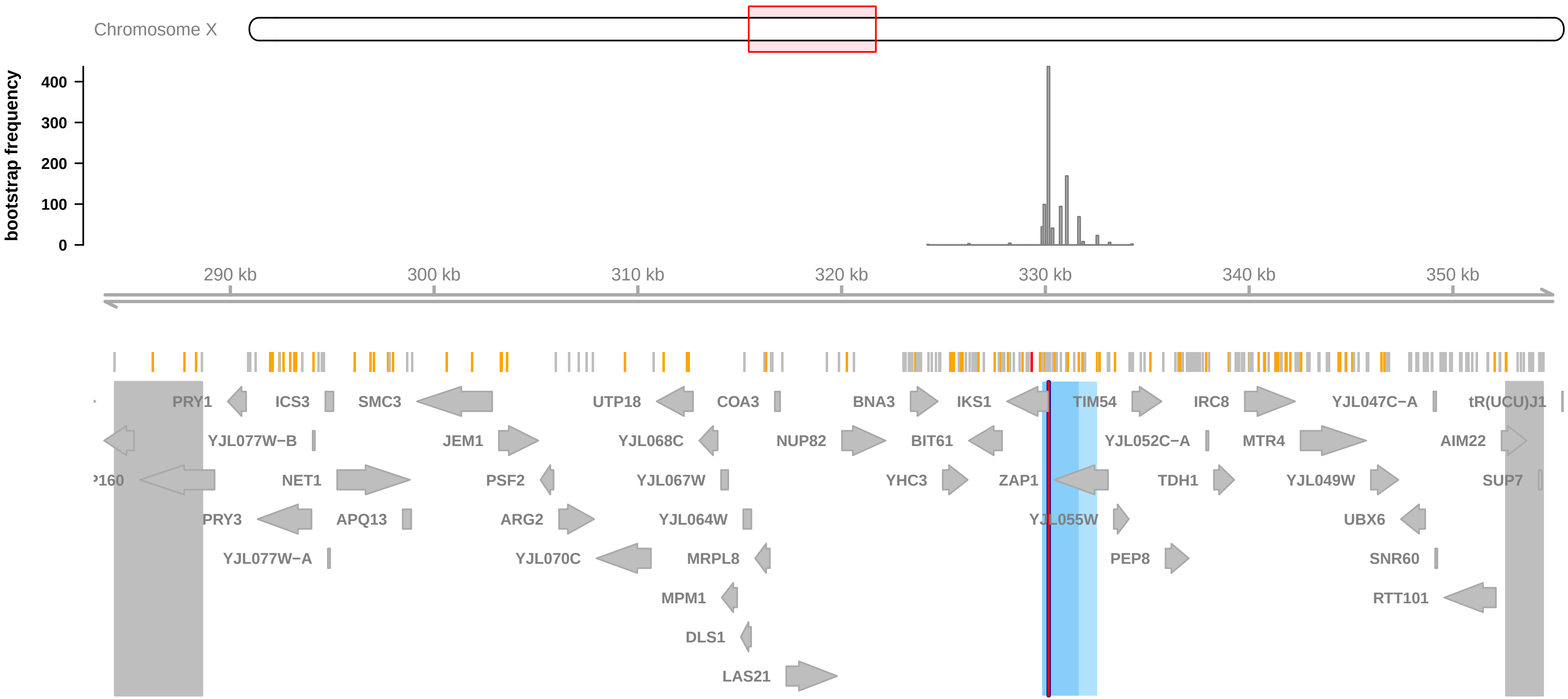

chrX:426287_C/A; 321 genes affected

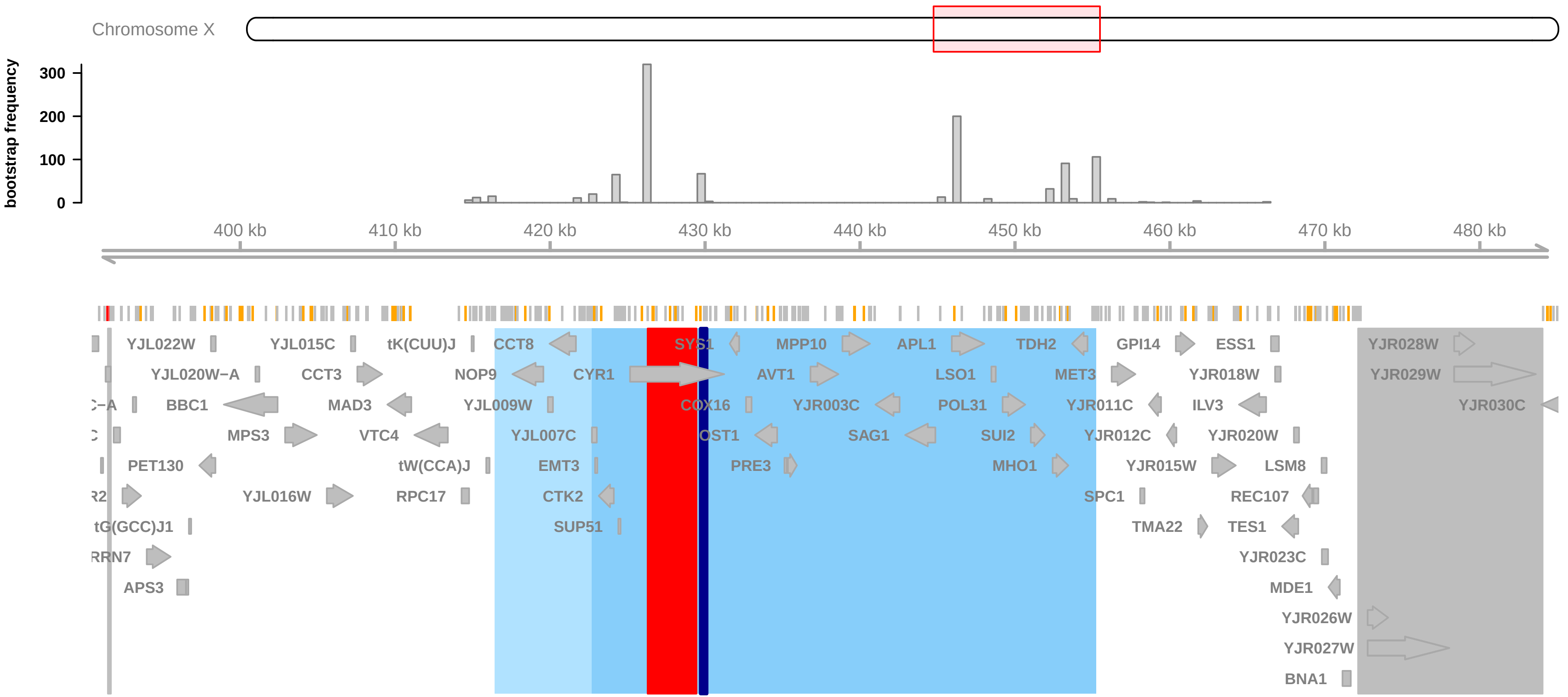

chrX:548174_T/C; 380 genes affected

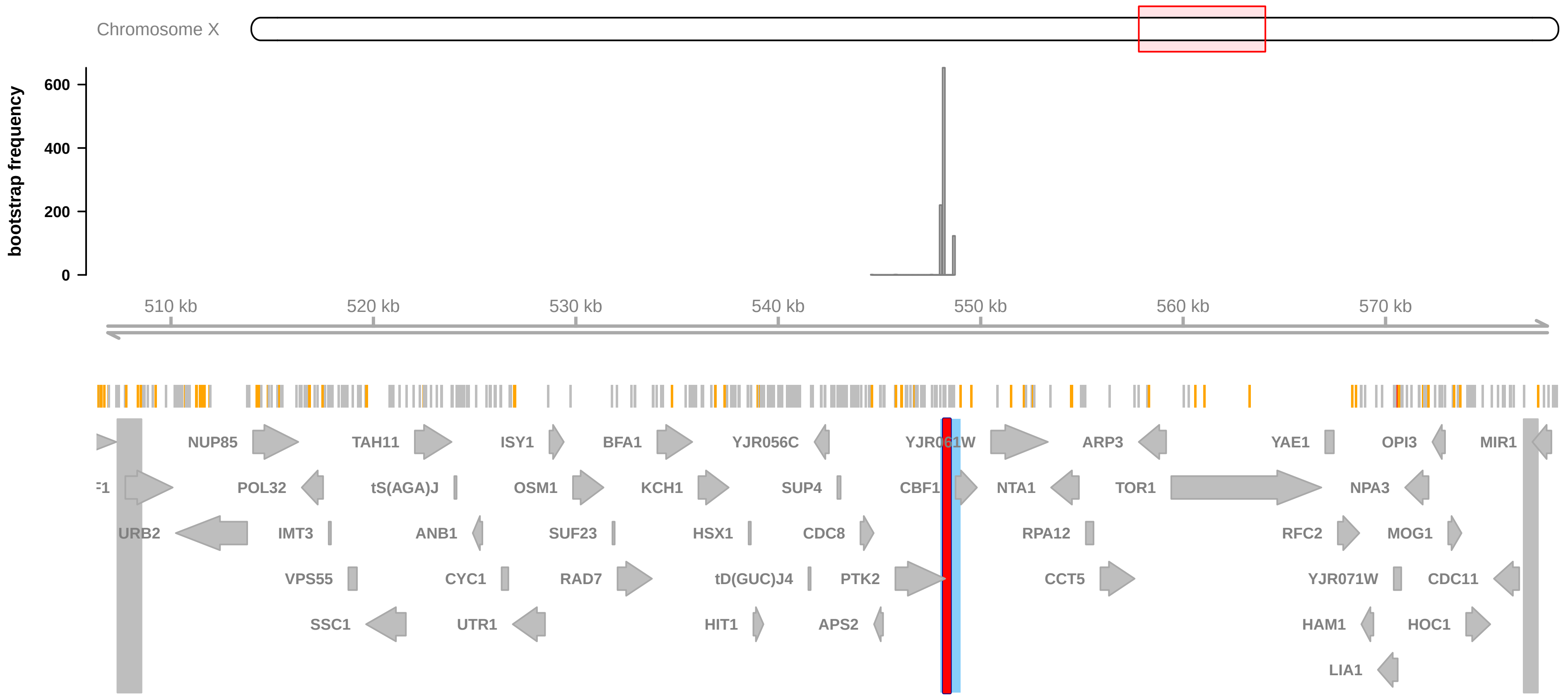

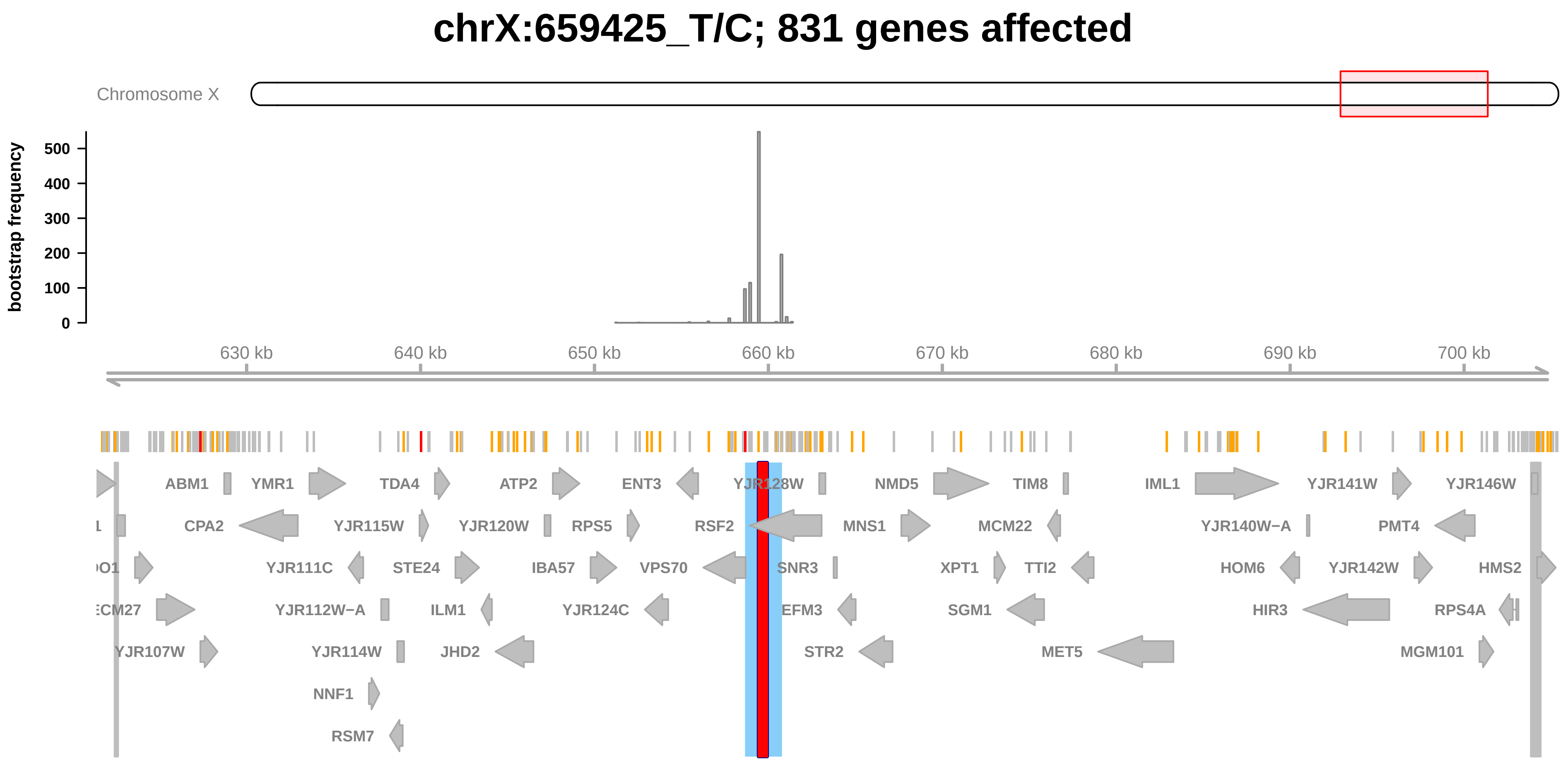
chrX:659425_T/C; 831 genes affected
Chromosome X
bootstrap frequency
0
100
200
300
400
500
630 kb
640 kb
650 kb
660 kb
670 kb
680 kb
690 kb
700 kb
ABM1
YMR1
TDA4
ATP2
ENT3
YJR128W
NMD5
TIM8
IML1
YJR141W
YJR146W
CPA2
YJR115W
YJR120W
RPS5
RSF2
MNS1
MCM22
YJR140W−A
PMT4
YJR111C
STE24
IBA57
VPS70
SNR3
XPT1
TTI2
HOM6
YJR142W
HMS2
YJR112W−A
ILM1
YJR124C
EFM3
SGM1
HIR3
RPS4A
YJR107W
YJR114W
JHD2
STR2
MET5
MGM101
NNF1
RSM7

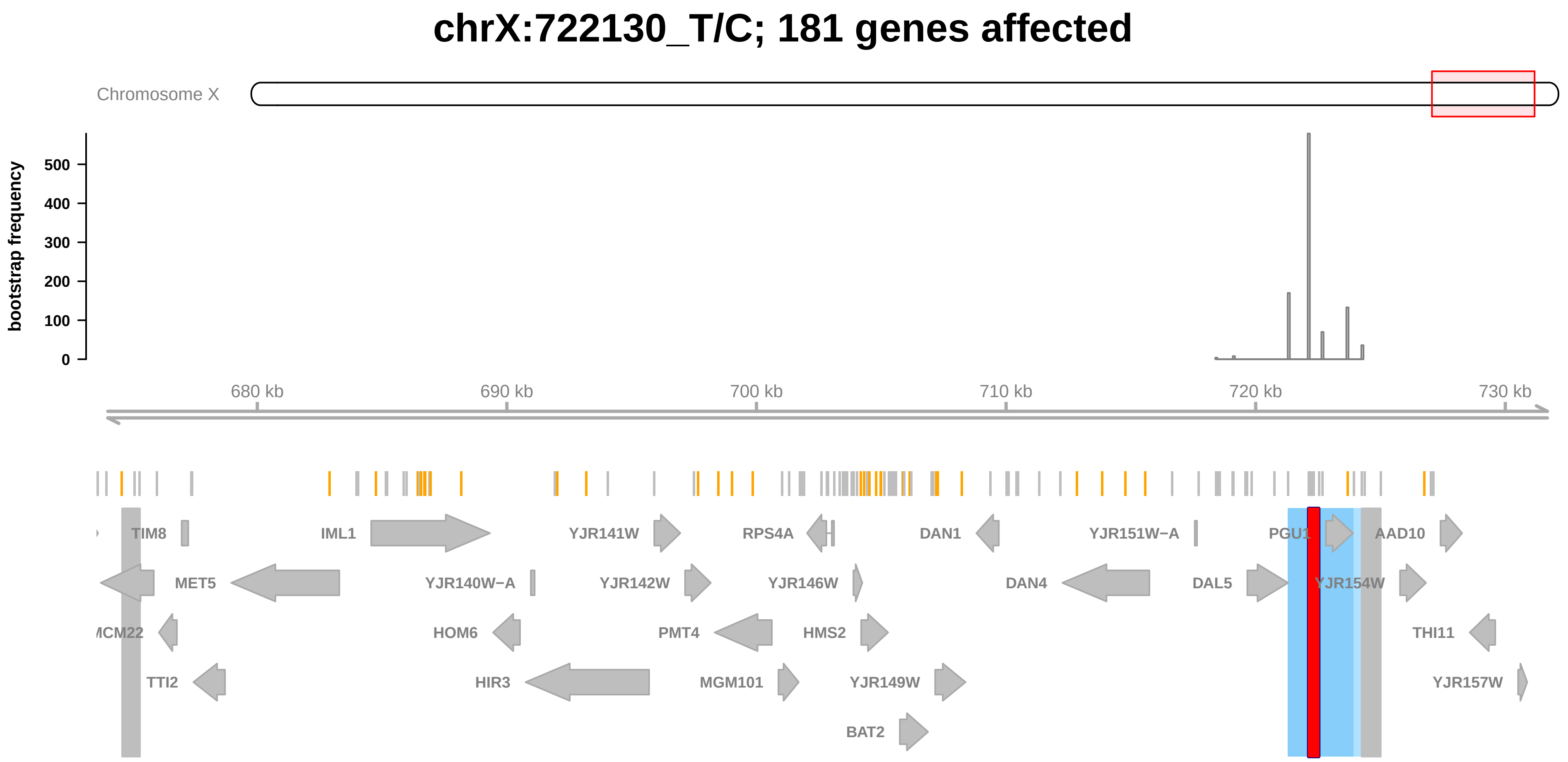

chrX:722130_T/C; 181 genes affected
Chromosome X
bootstrap frequency
500
400
300
200
100
0
680 kb
690 kb
700 kb
710 kb
720 kb
730 kb
TIM8
IML1
YJR141W
RPS4A
DAN1
YJR151W−A
PGU1
AAD10
MET5
YJR140W−A
YJR142W
YJR146W
DAN4
DAL5
YJR154W
HOM6
PMT4
HMS2
THI11
TTI2
HIR3
MGM101
YJR149W
YJR157W
BAT2

chrXI:4534_G/A; 123 genes affected

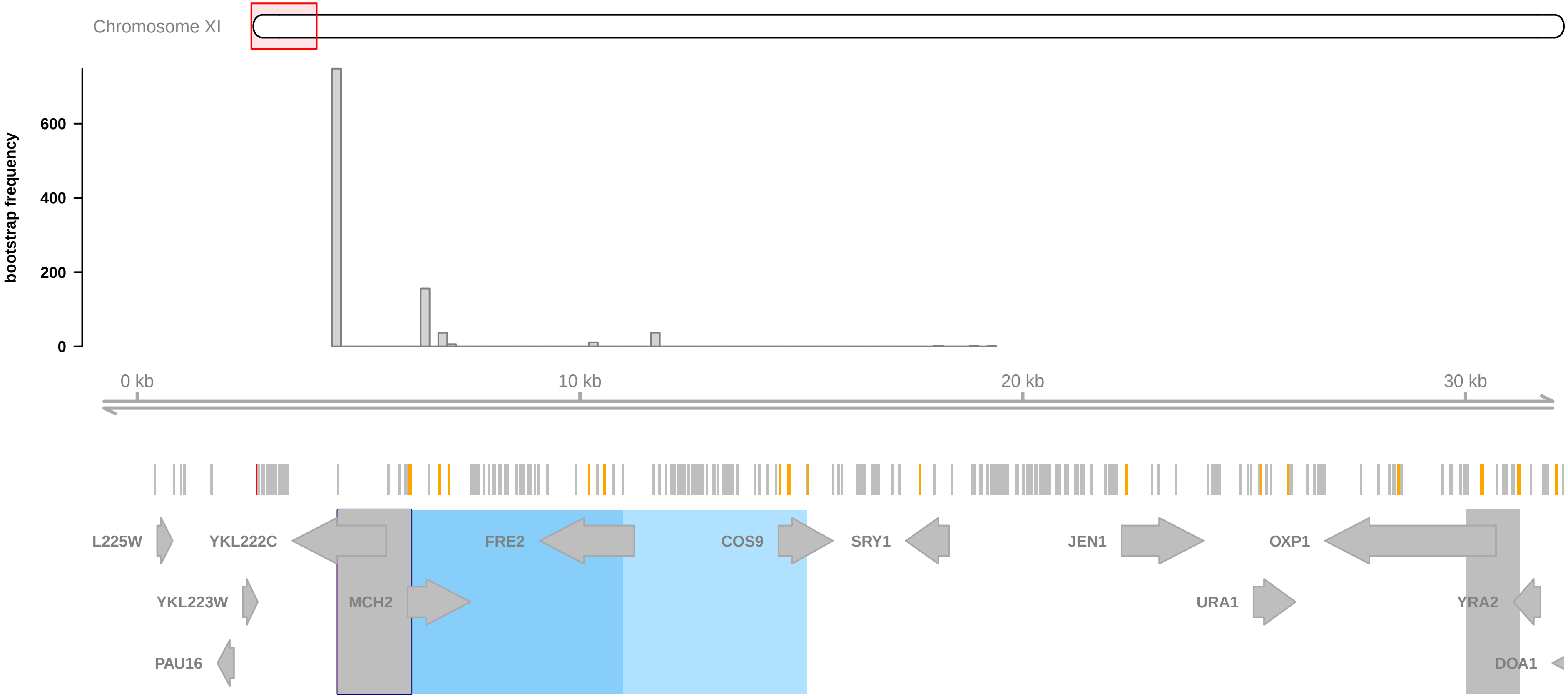

chrXI:102908_C/T; 387 genes affected

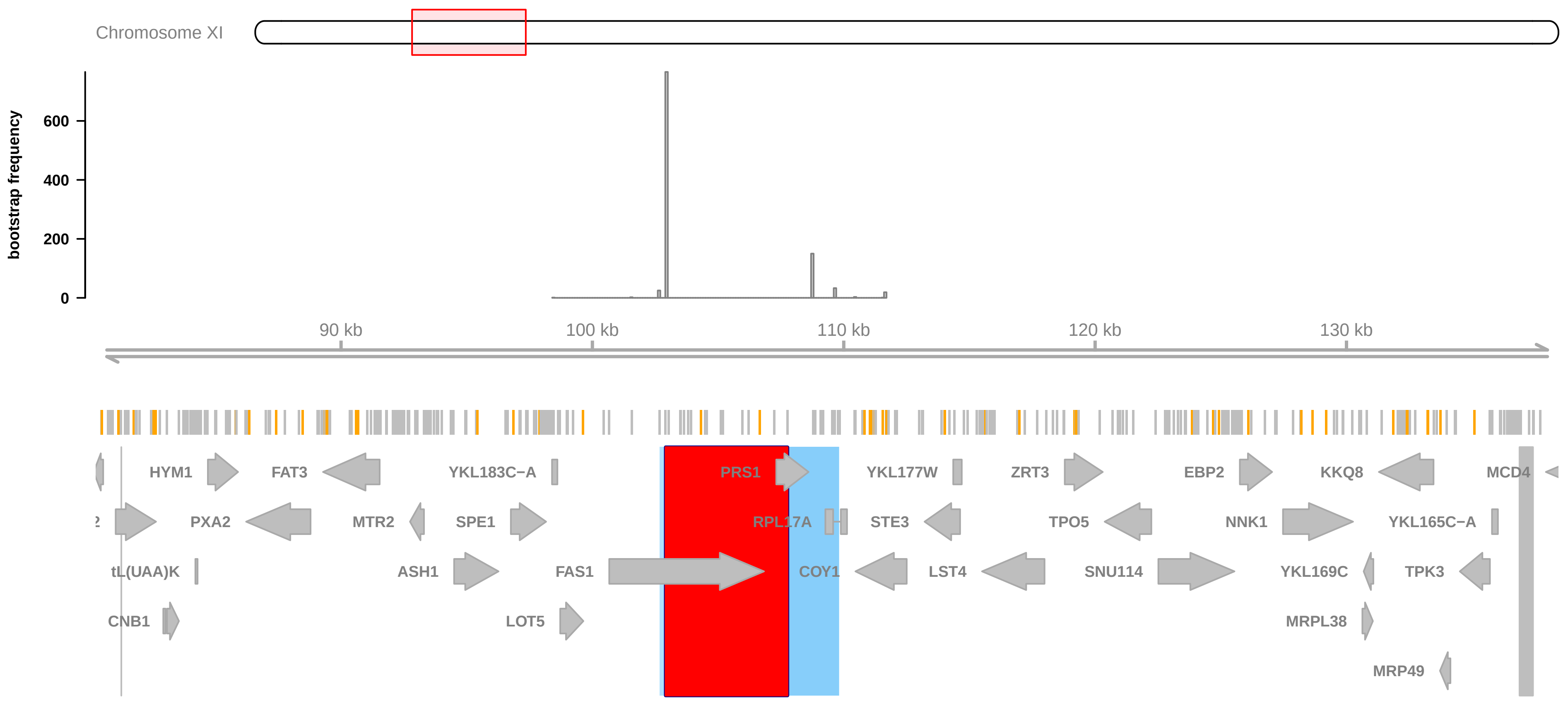

chrXI:232667_A/T; 833 genes affected

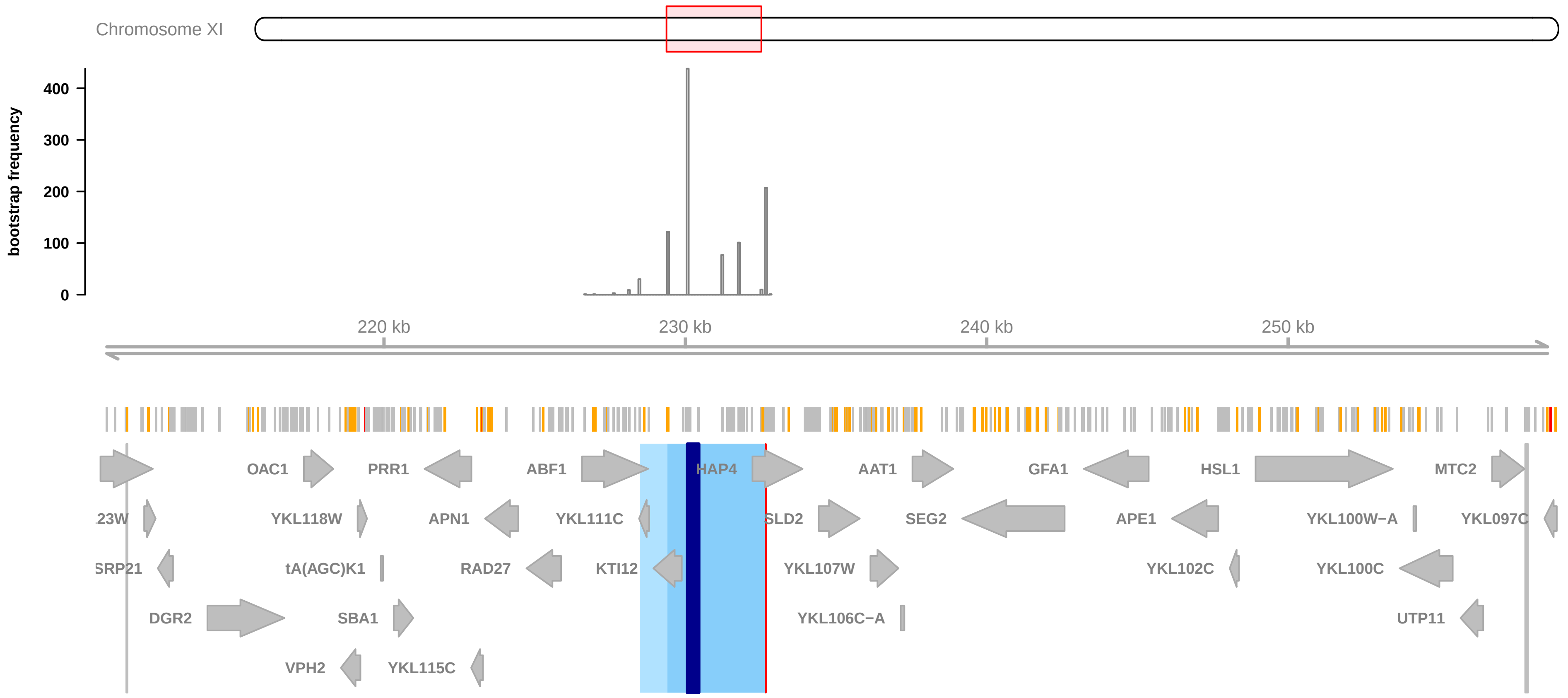

chrXI:411756_T/C; 208 genes affected

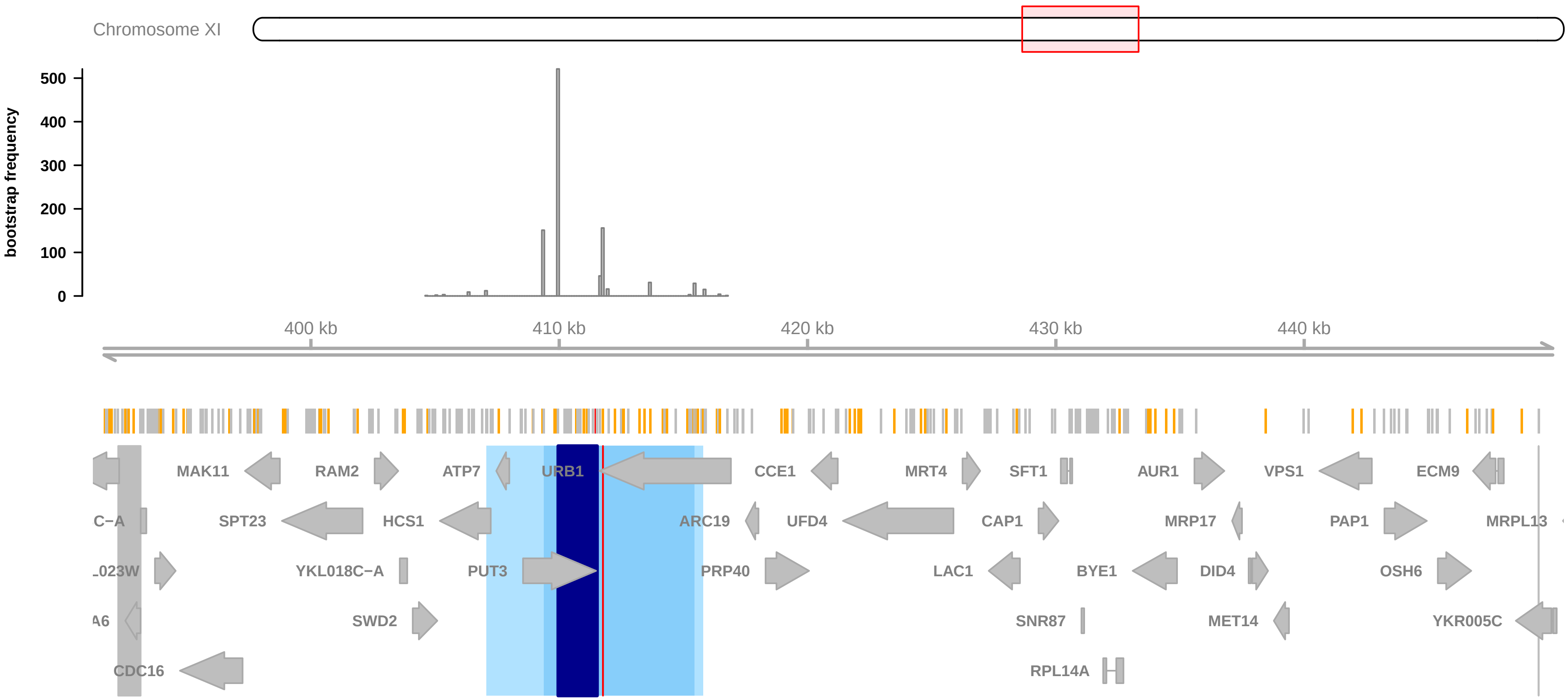

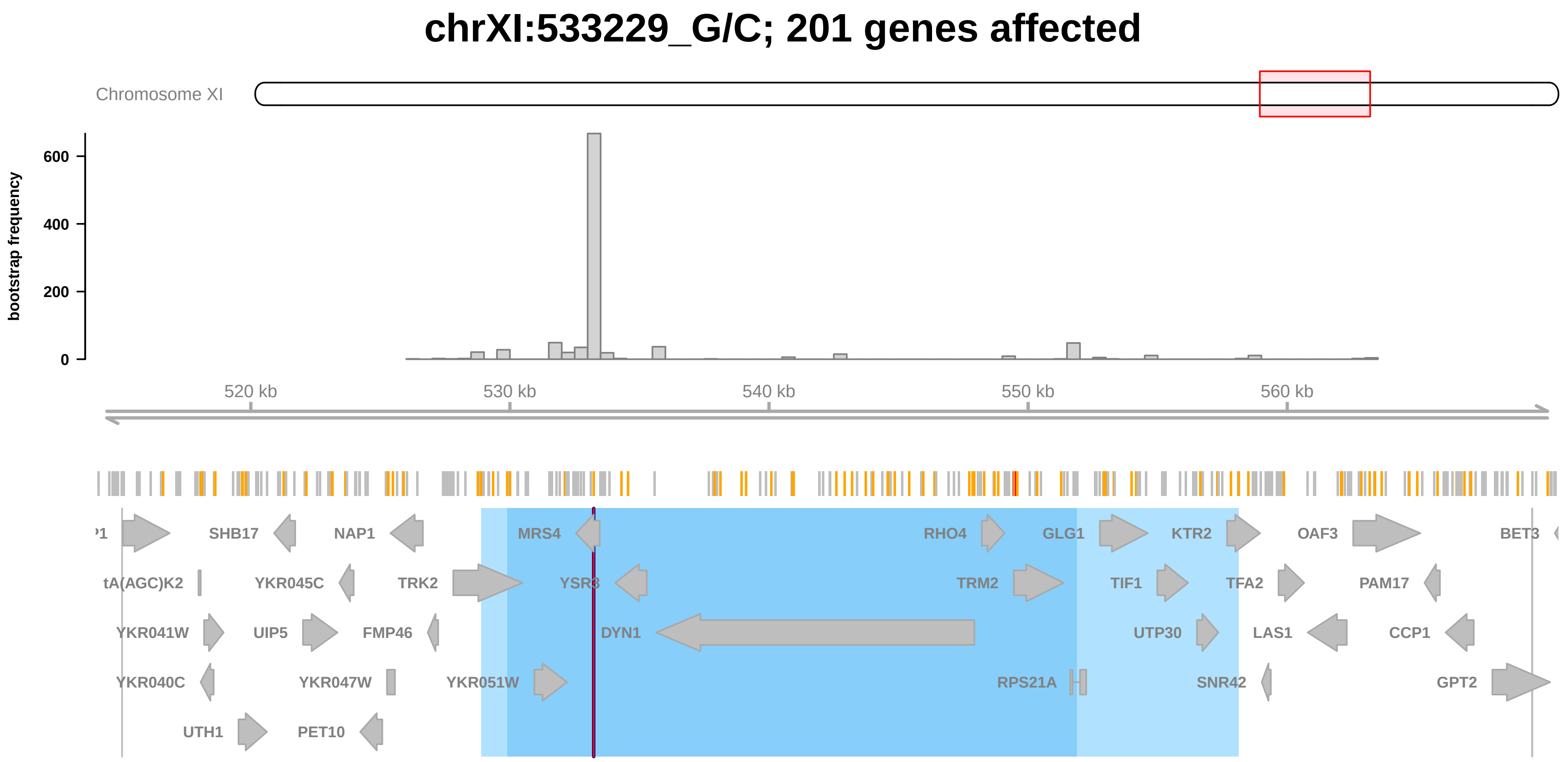
chrXI:533229_G/C; 201 genes affected
Chromosome XI
bootstrap frequency
600
400
200
0
520 kb
530 kb
540 kb
550 kb
560 kb
SHB17
NAP1
MRS4
RHO4
GLG1
KTR2
OAF3
BET3
tA(AGC)K2
YKR045C
TRK2
TRM2
TIF1
TFA2
PAM17
YKR041W
UIP5
FMP46
DYN1
UTP30
LAS1
CCP1
YKR040C
YKR047W
YKR051W
RPS21A
SNR42
GPT2
UTH1
PET10

chrXI:641098_A/G; 192 genes affected

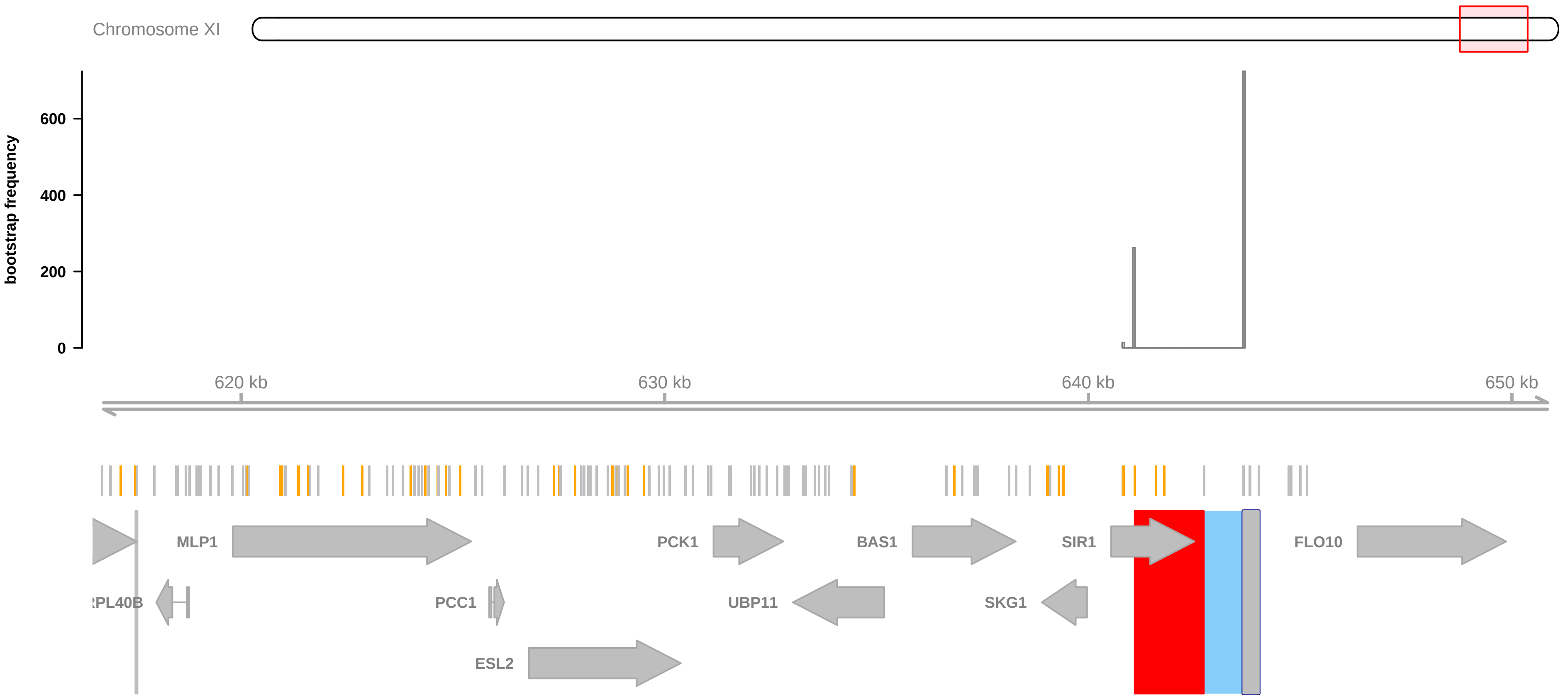

chrXII:136527_T/C; 2597 genes affected

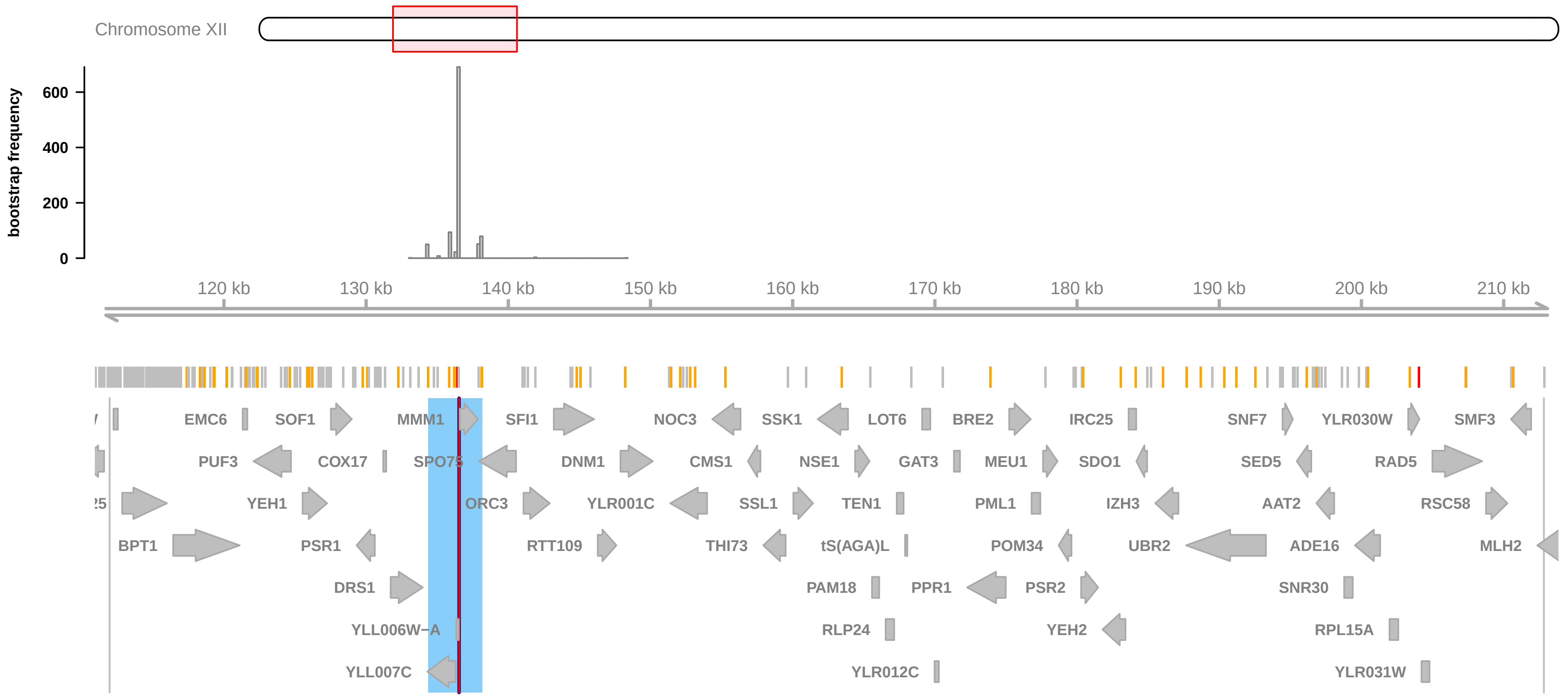

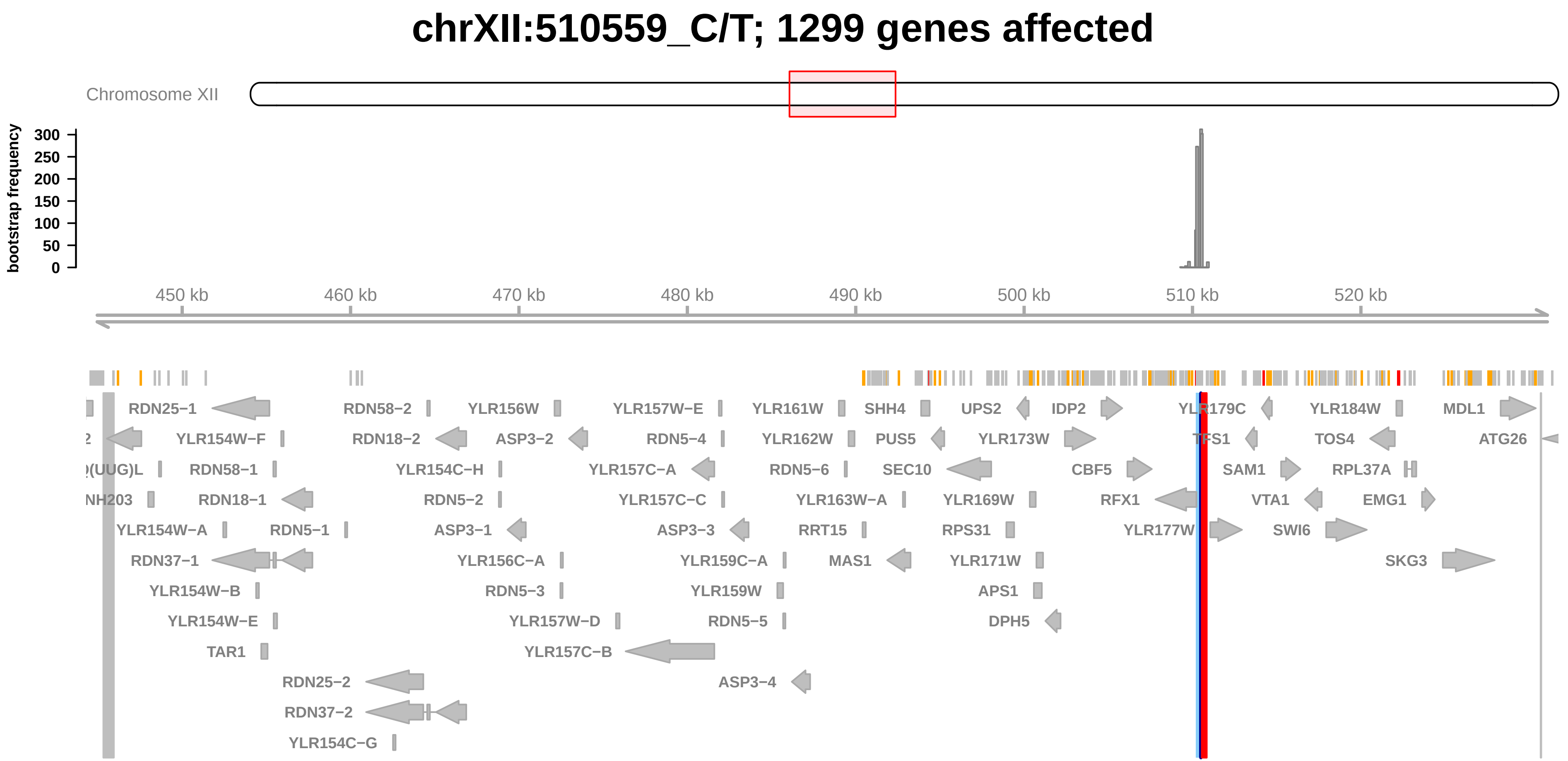
chrXII:510559_C/T; 1299 genes affected
Chromosome XII
bootstrap frequency
300
250
200
150
100
50
0
450 kb
460 kb
470 kb
480 kb
490 kb
500 kb
510 kb
520 kb
RDN25−1
RDN58−2
YLR156W
YLR157W−E
YLR161W
SHH4
UPS2
IDP2
YLR179C
YLR184W
MDL1
YLR154W−F
RDN18−2
ASP3−2
RDN5−4
YLR162W
PUS5
YLR173W
TFS1
TOS4
ATG26
RDN58−1
YLR154C−H
YLR157C−A
RDN5−6
SEC10
CBF5
SAM1
RPL37A
RDN18−1
RDN5−2
YLR157C−C
YLR163W−A
YLR169W
RFX1
VTA1
EMG1
YLR154W−A
RDN5−1
ASP3−1
ASP3−3
RRT15
RPS31
YLR177W
SWI6
RDN37−1
YLR156C−A
YLR159C−A
MAS1
YLR171W
SKG3
YLR154W−B
RDN5−3
YLR159W
APS1
YLR154W−E
YLR157W−D
RDN5−5
DPH5
TAR1
YLR157C−B
RDN25−2
ASP3−4
RDN37−2
YLR154C−G

chrXII:657022_T/C; 3755 genes affected

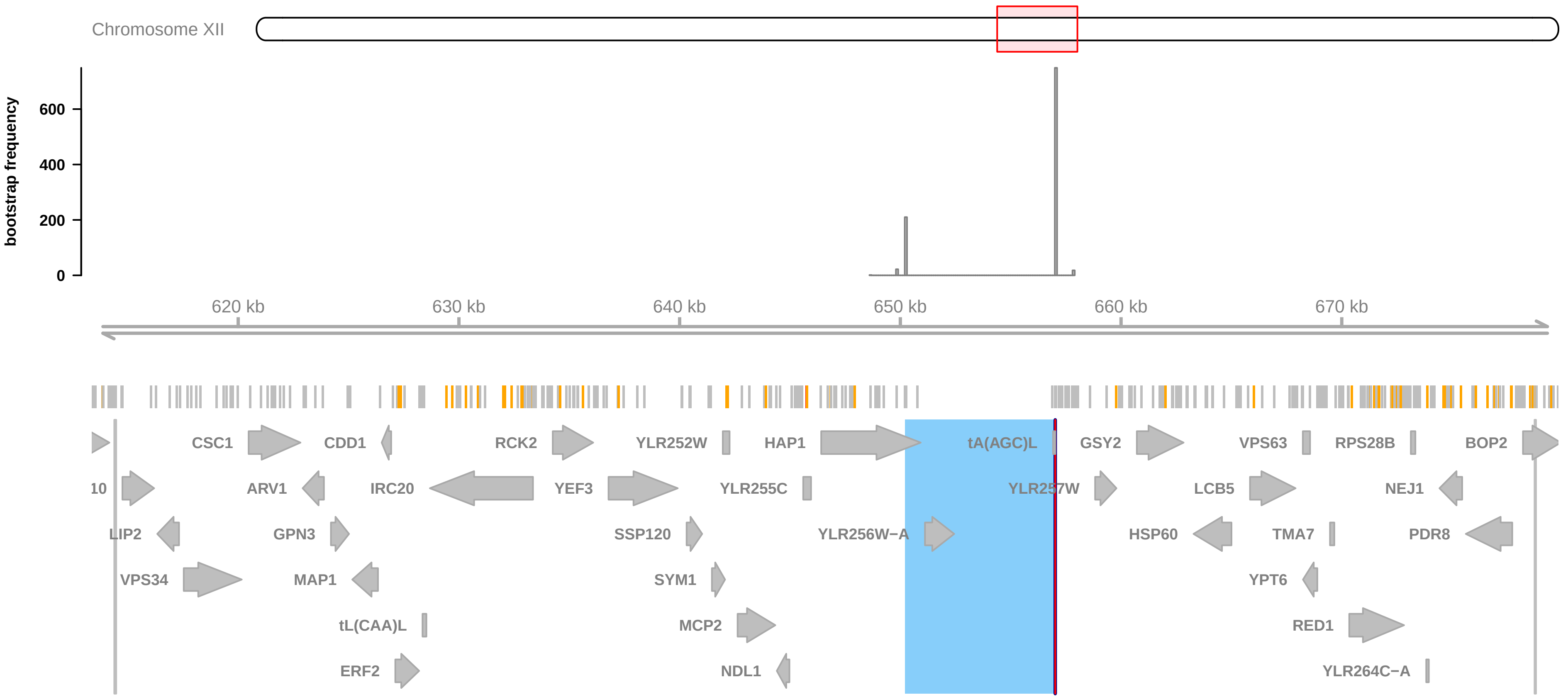

chrXII:694841_T/C; 2362 genes affected

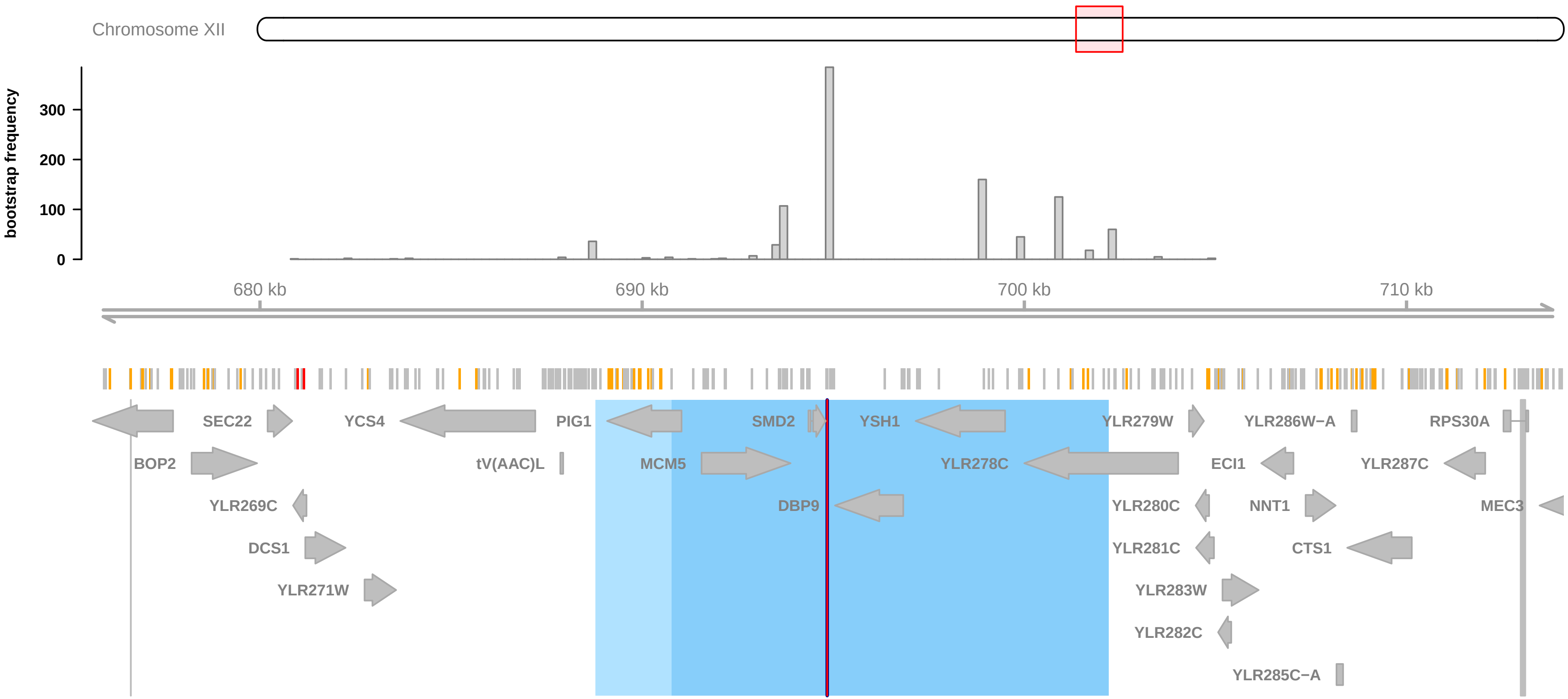

chrXII:848842_A/G; 2234 genes affected

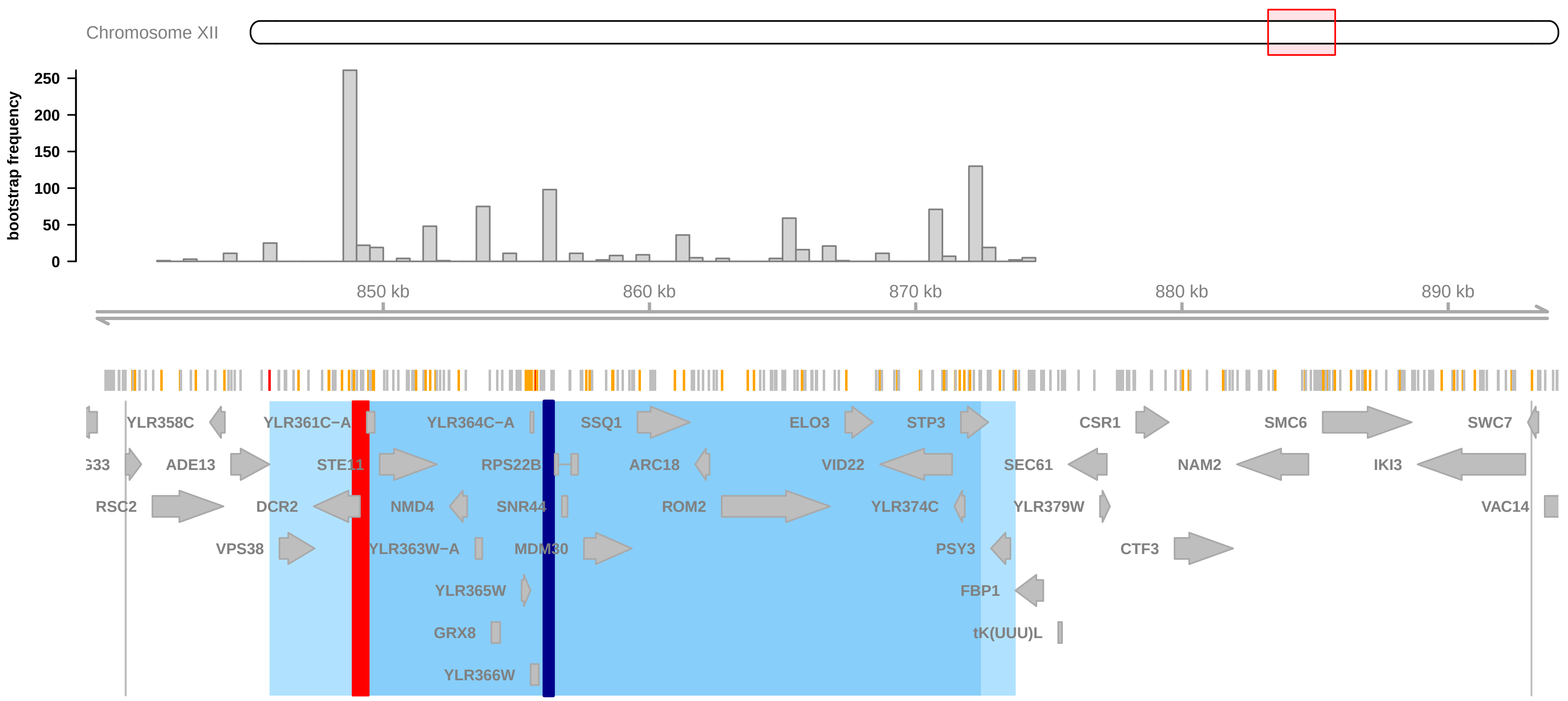

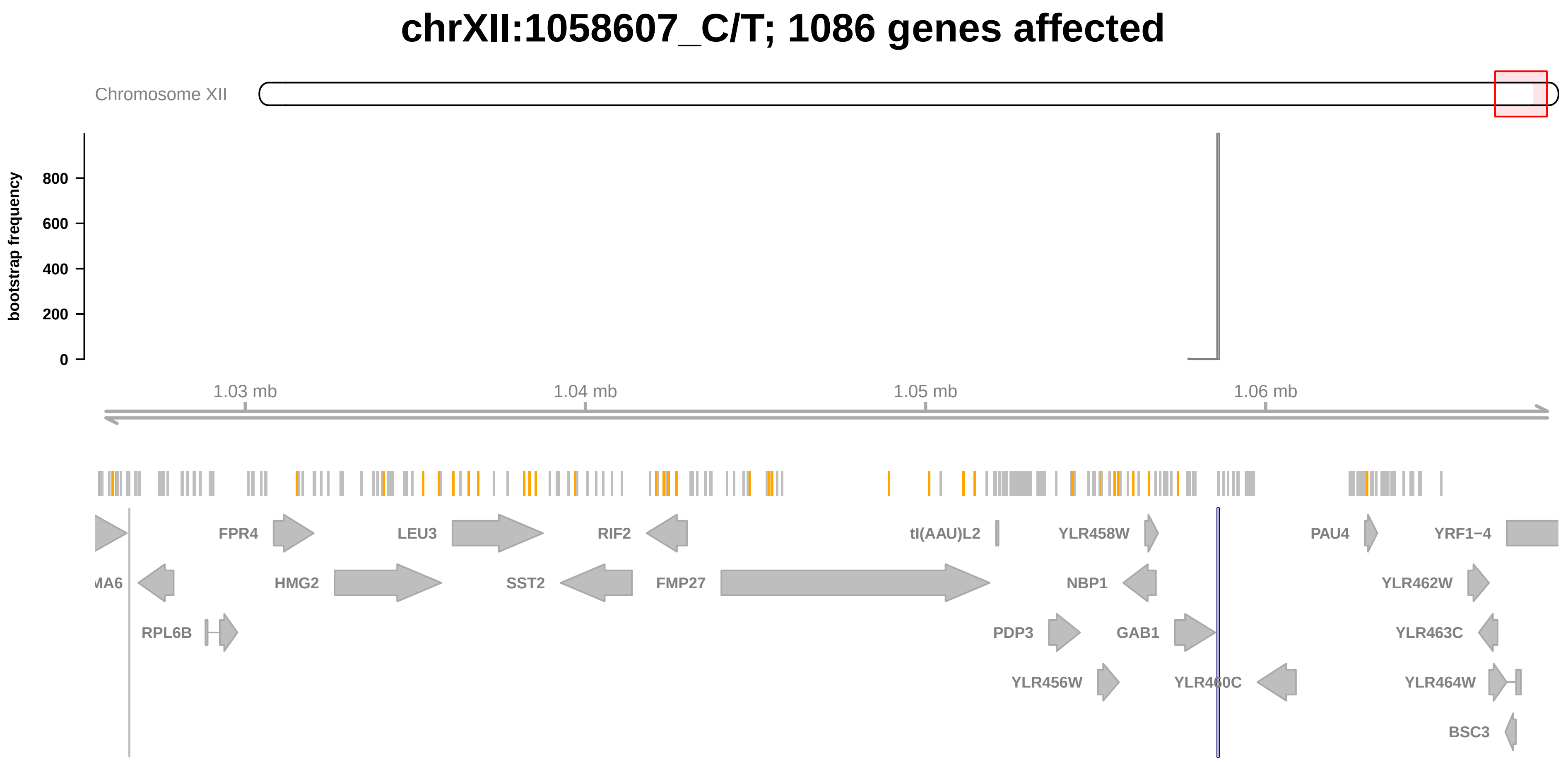
chrXII:1058607_C/T; 1086 genes affected
Chromosome XII
bootstrap frequency
800
600
400
200
0
1.03 mb
1.04 mb
1.05 mb
1.06 mb
FPR4
LEU3
RIF2
tI(AAU)L2
YLR458W
PAU4
YRF1−4
MA6
HMG2
SST2
FMP27
NBP1
YLR462W
RPL6B
PDP3
GAB1
YLR463C
YLR456W
YLR460C
YLR464W
BSC3

chrXIII:33836_G/A; 637 genes affected

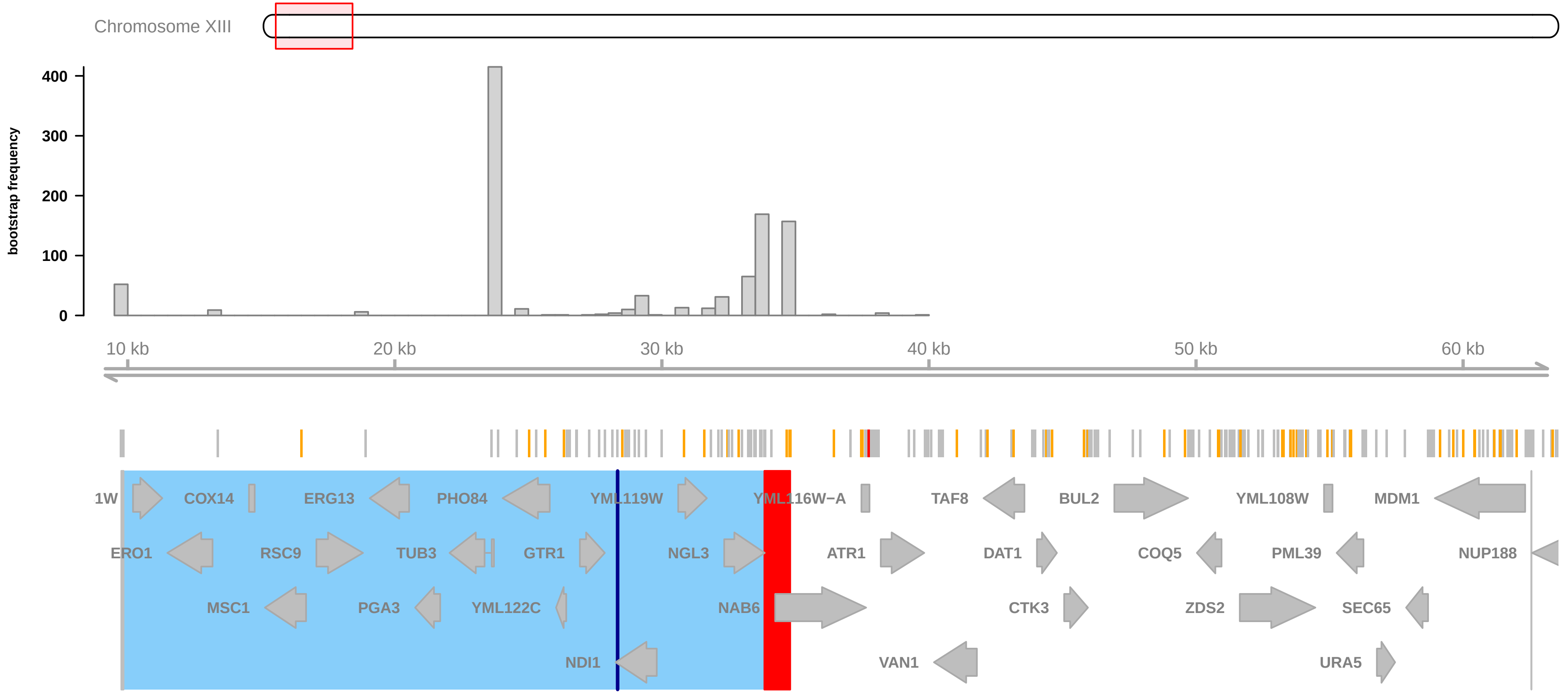

chrXIII:85889_GA/G; 801 genes affected

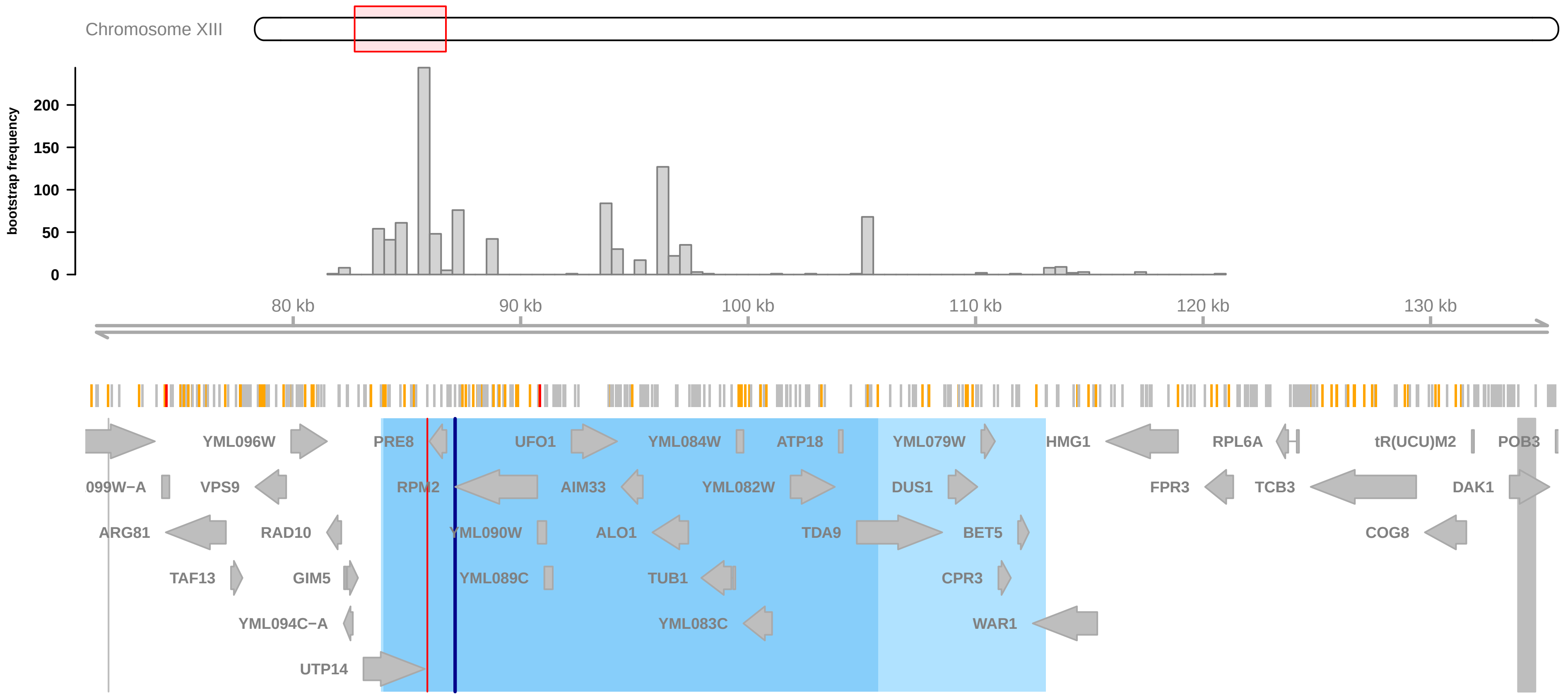

chrXIII:253667_T/C; 829 genes affected

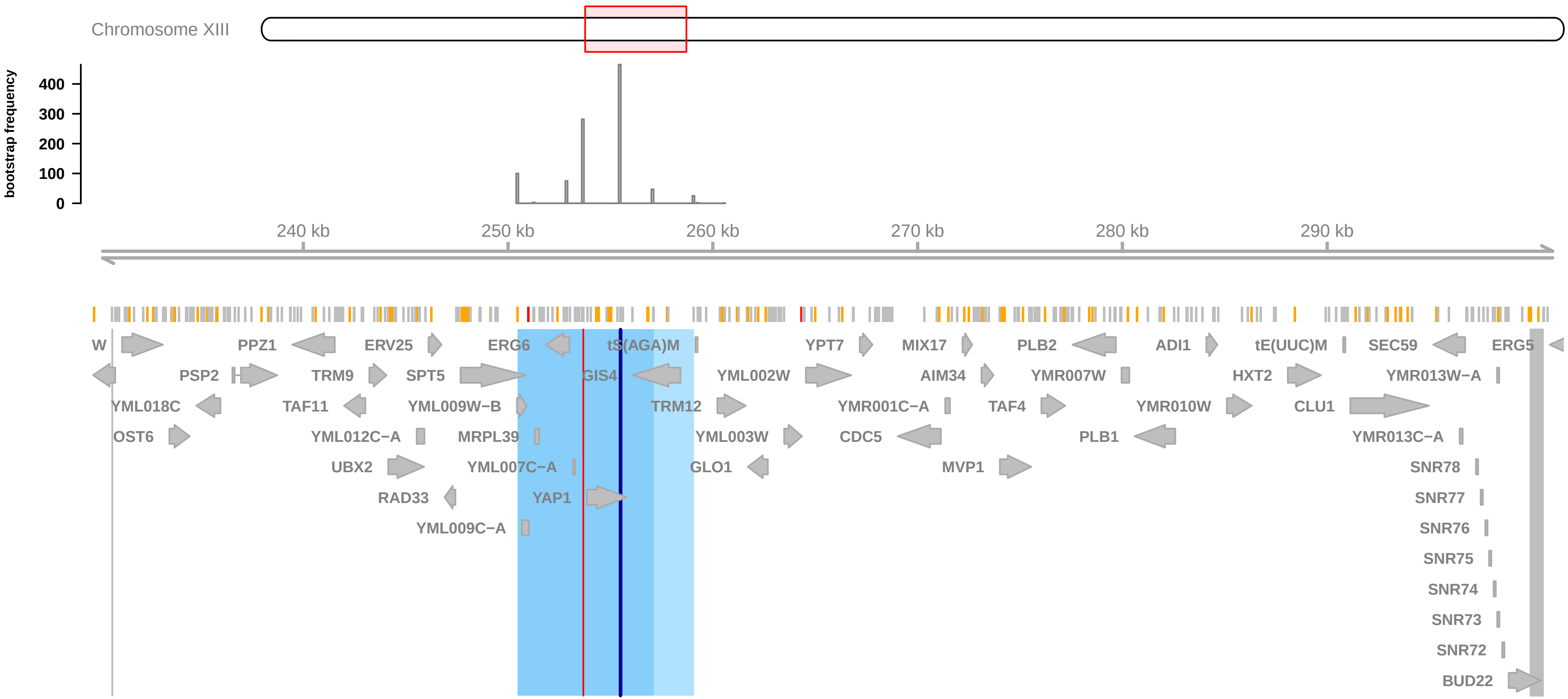

chrXIII:333449_G/A; 1324 genes affected

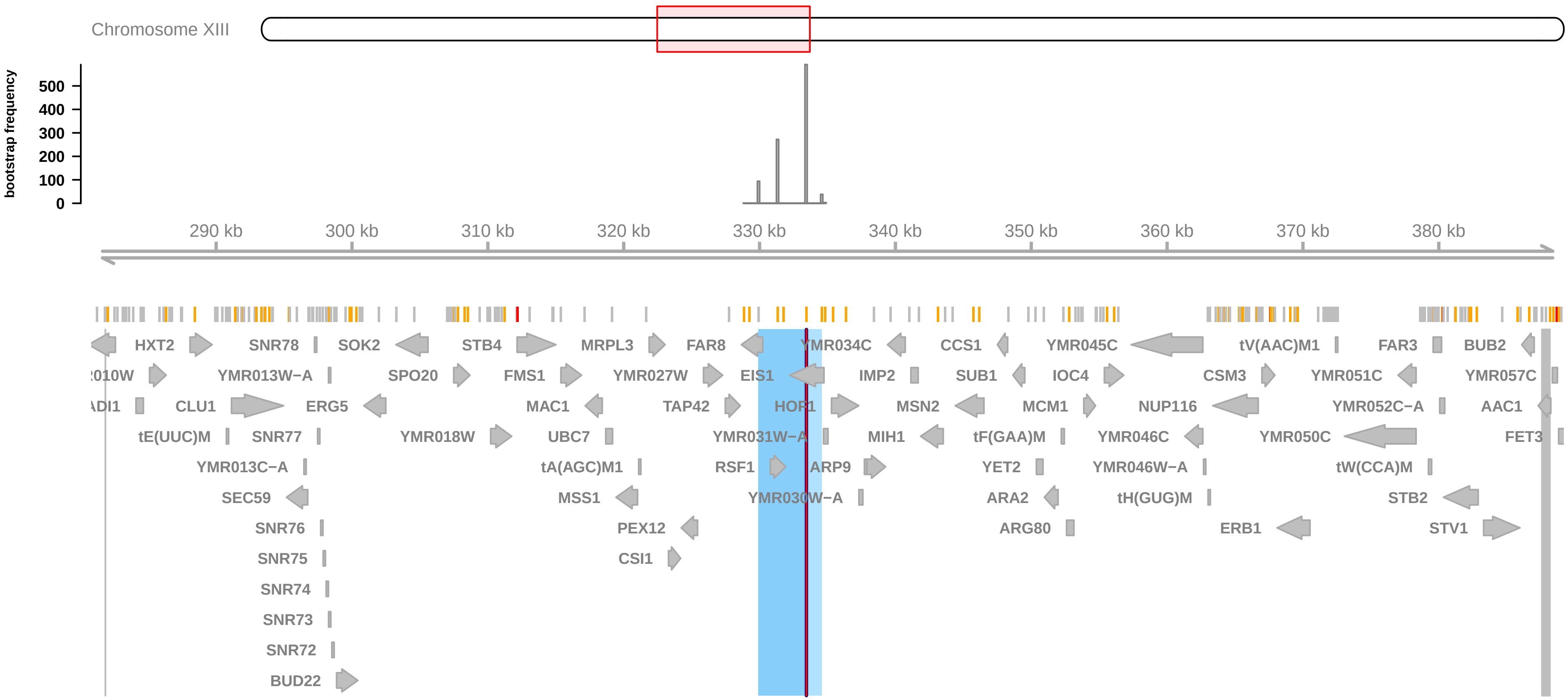

chrXIII:409778_G/T; 1293 genes affected

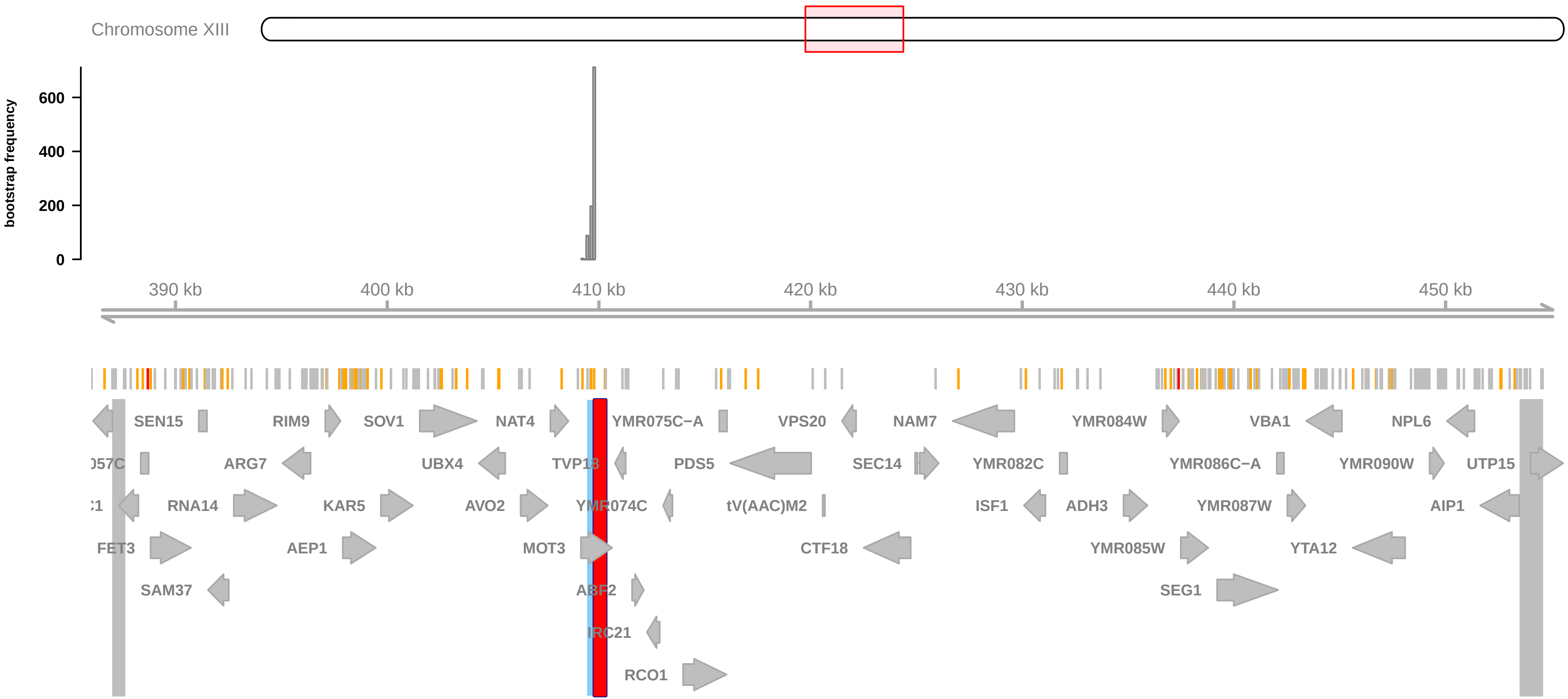

chrXIII:591723_A/G; 710 genes affected

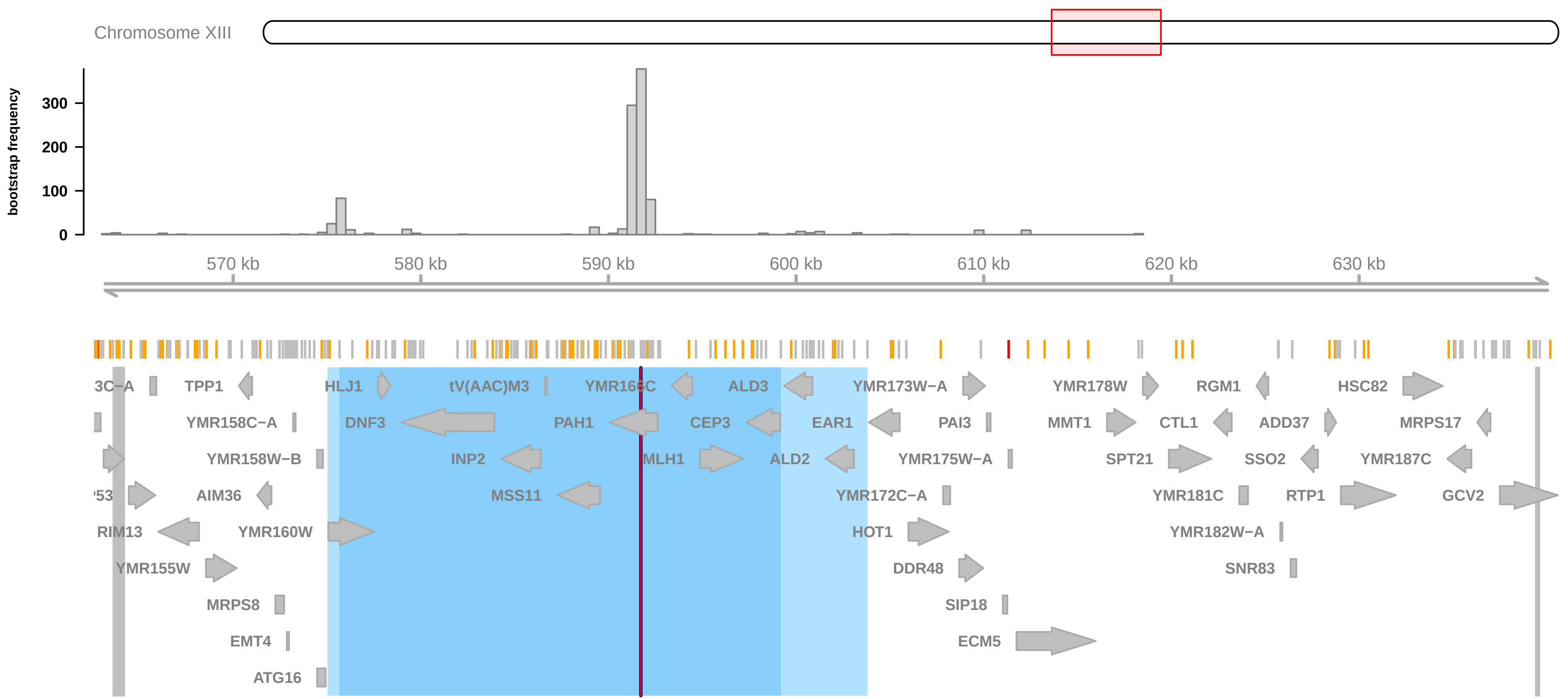

chrXIII:761318_T/A; 616 genes affected

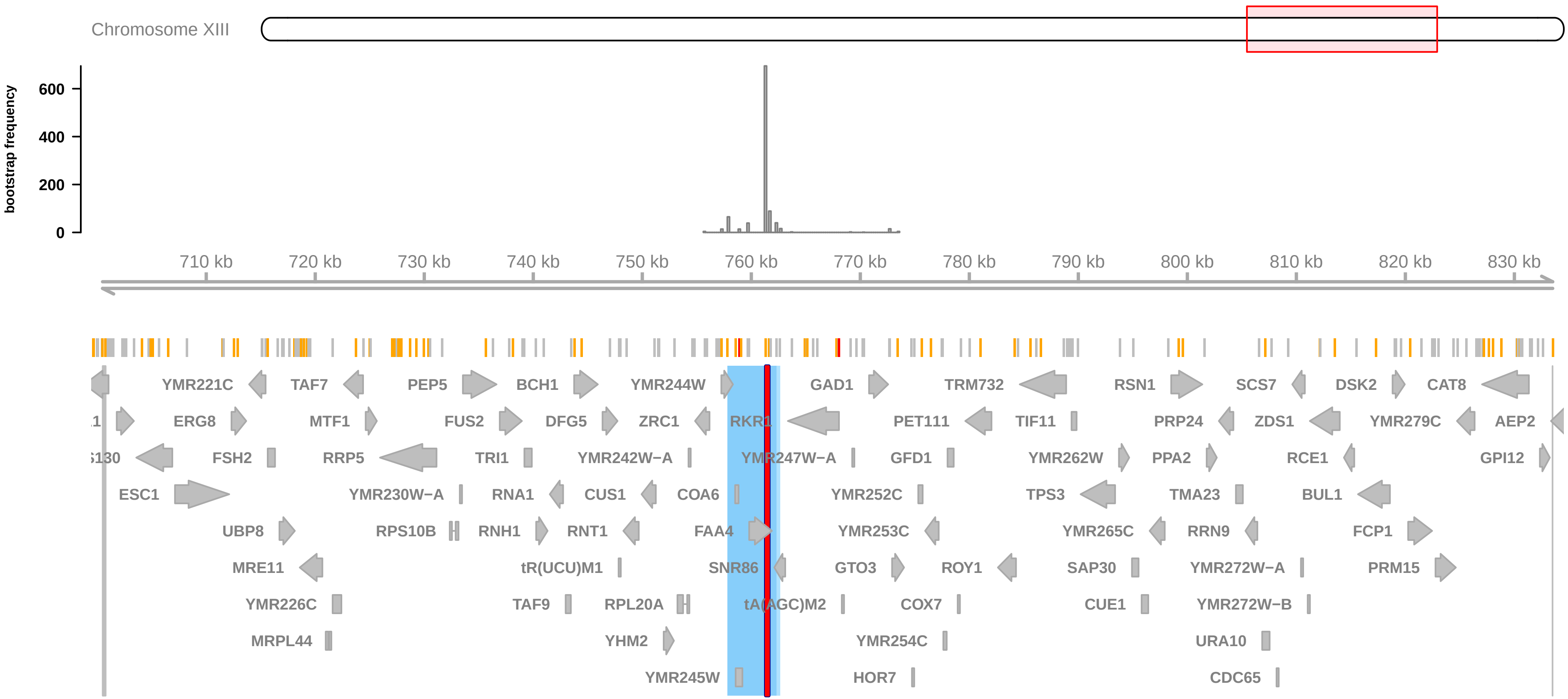

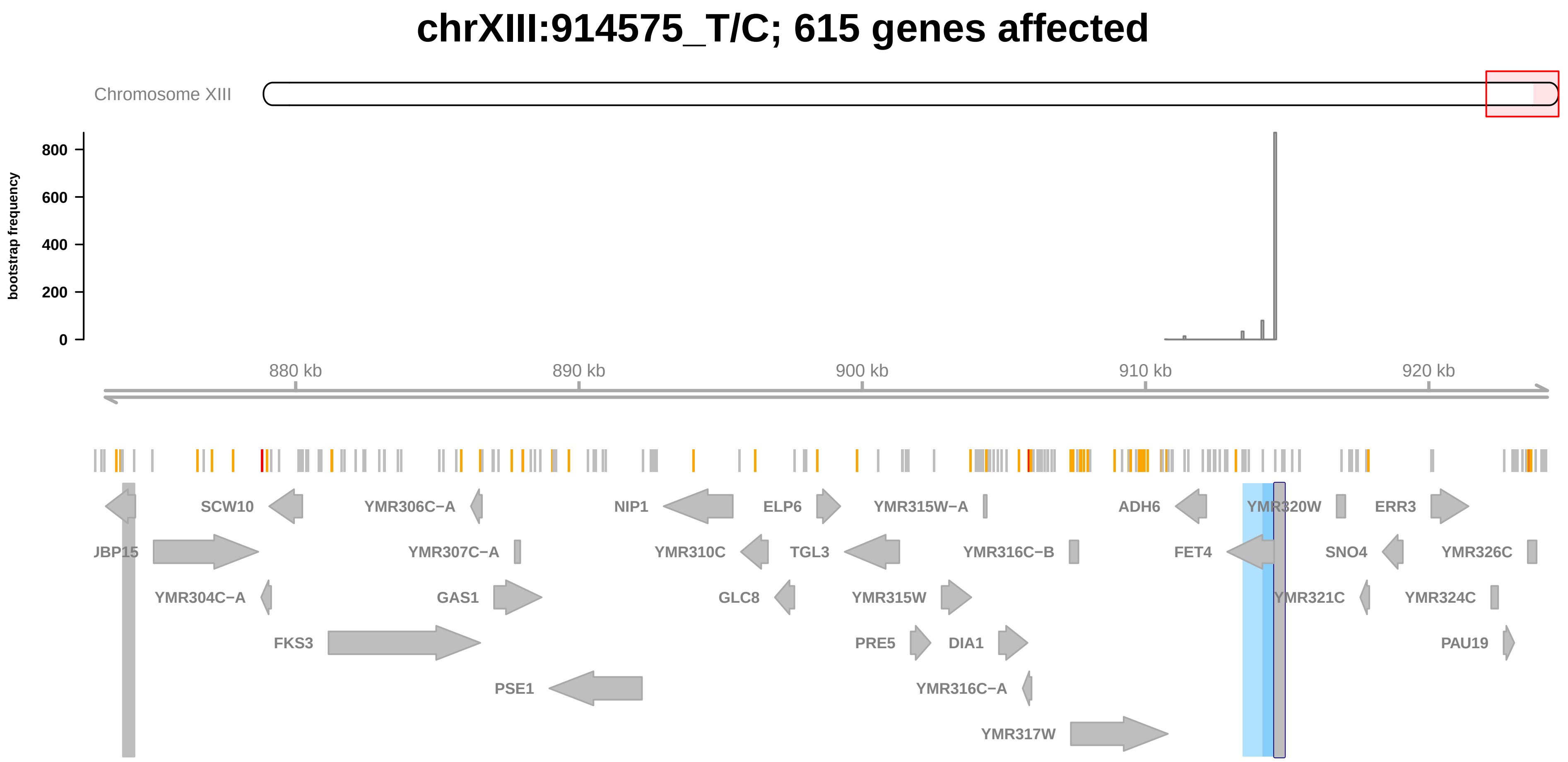

chrXIII:914575_T/C; 615 genes affected
Chromosome XIII
bootstrap frequency
800
600
400
200
0
880 kb
890 kb
900 kb
910 kb
920 kb
SCW10
YMR306C−A
NIP1
ELP6
YMR315W−A
ADH6
YMR320W
ERR3
JBP15
YMR307C−A
YMR310C
TGL3
YMR316C−B
FET4
SNO4
YMR326C
YMR304C−A
GAS1
GLC8
YMR315W
YMR321C
YMR324C
FKS3
PRE5
DIA1
PAU19
PSE1
YMR316C−A
YMR317W

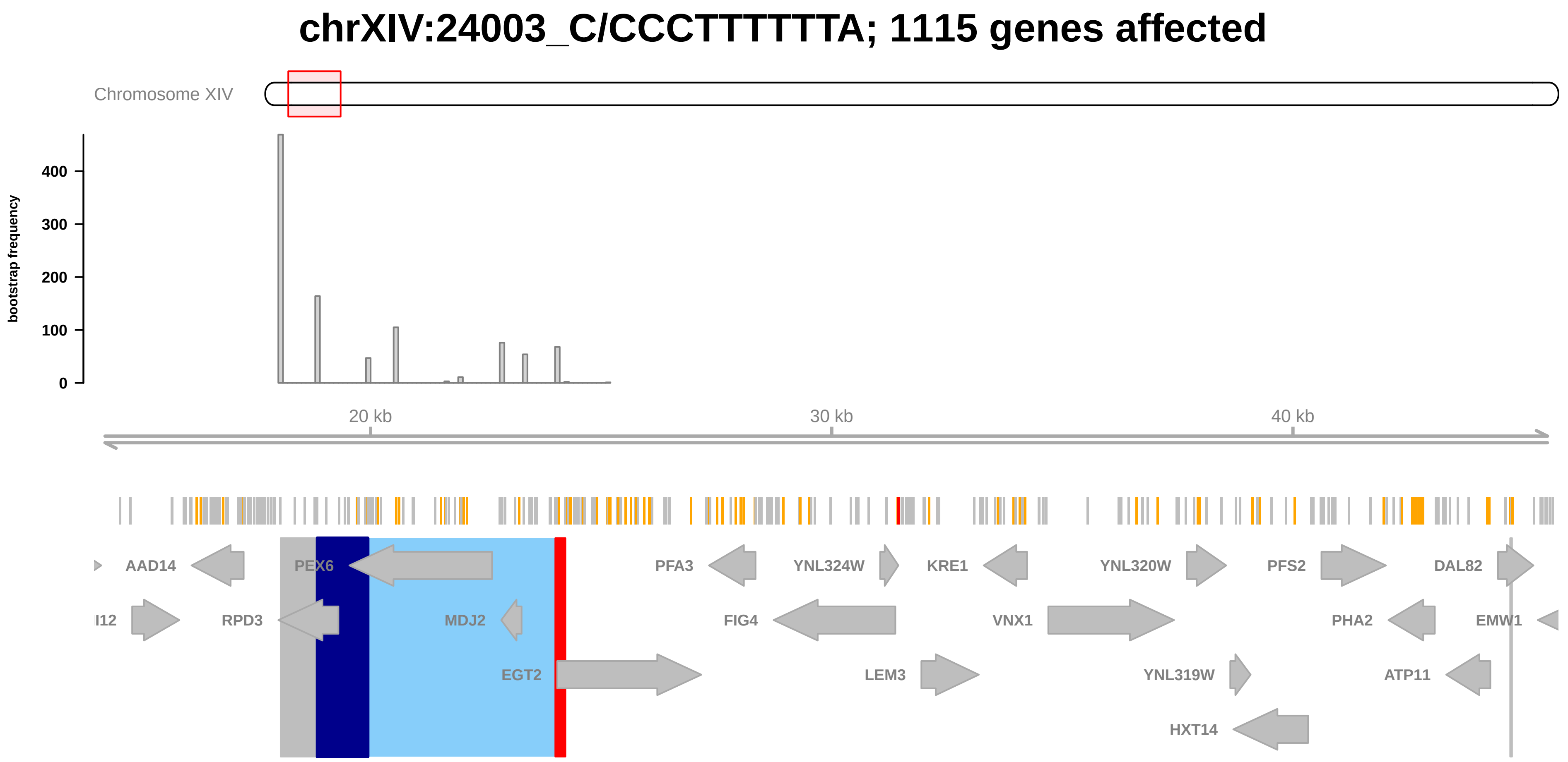

chrXIV:24003_C/CCCTTTTTTA; 1115 genes affected
Chromosome XIV
bootstrap frequency
0
100
200
300
400
20 kb
30 kb
40 kb
AAD14
PEX6
PFA3
YNL324W
KRE1
YNL320W
PFS2
DAL82
I12
RPD3
MDJ2
FIG4
VNX1
PHA2
EMW1
EGT2
LEM3
YNL319W
ATP11
HXT14

chrXIV:267161_G/A; 3166 genes affected

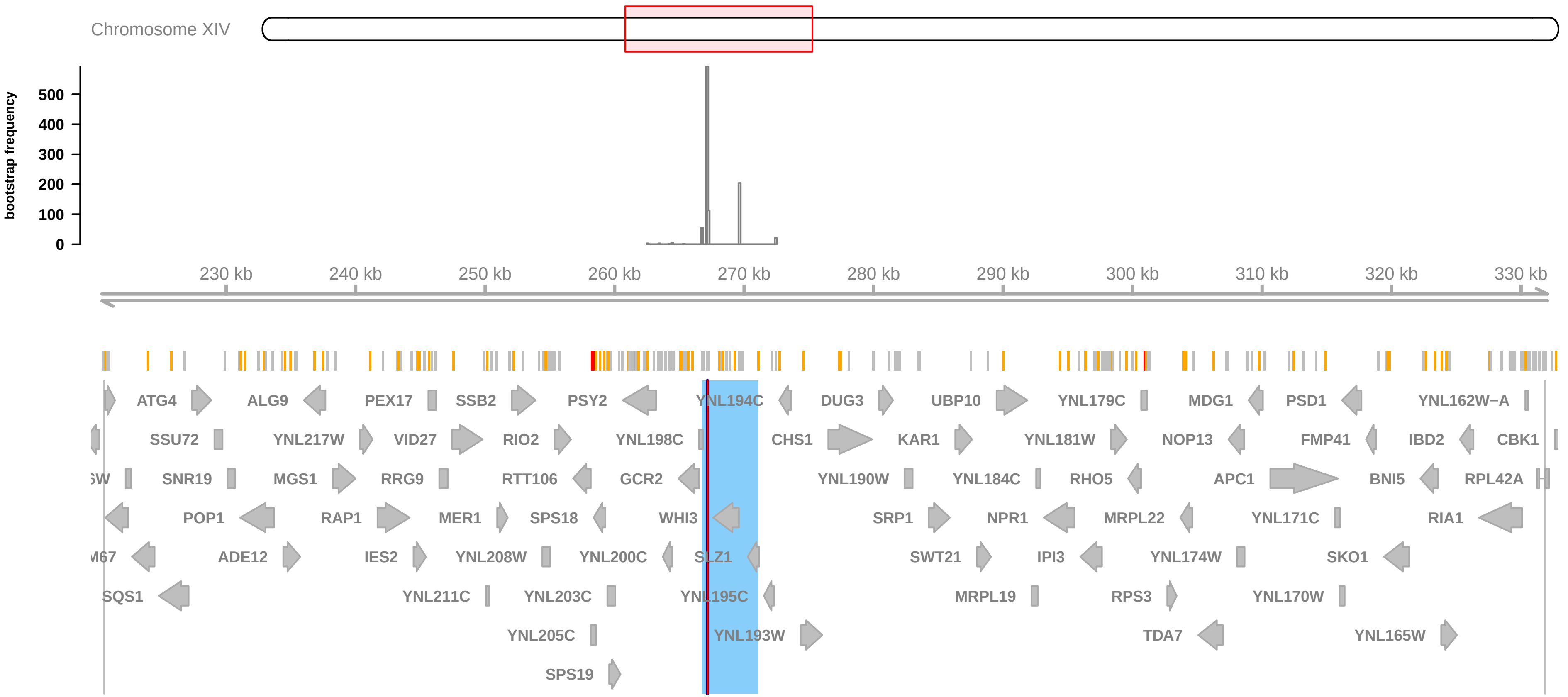

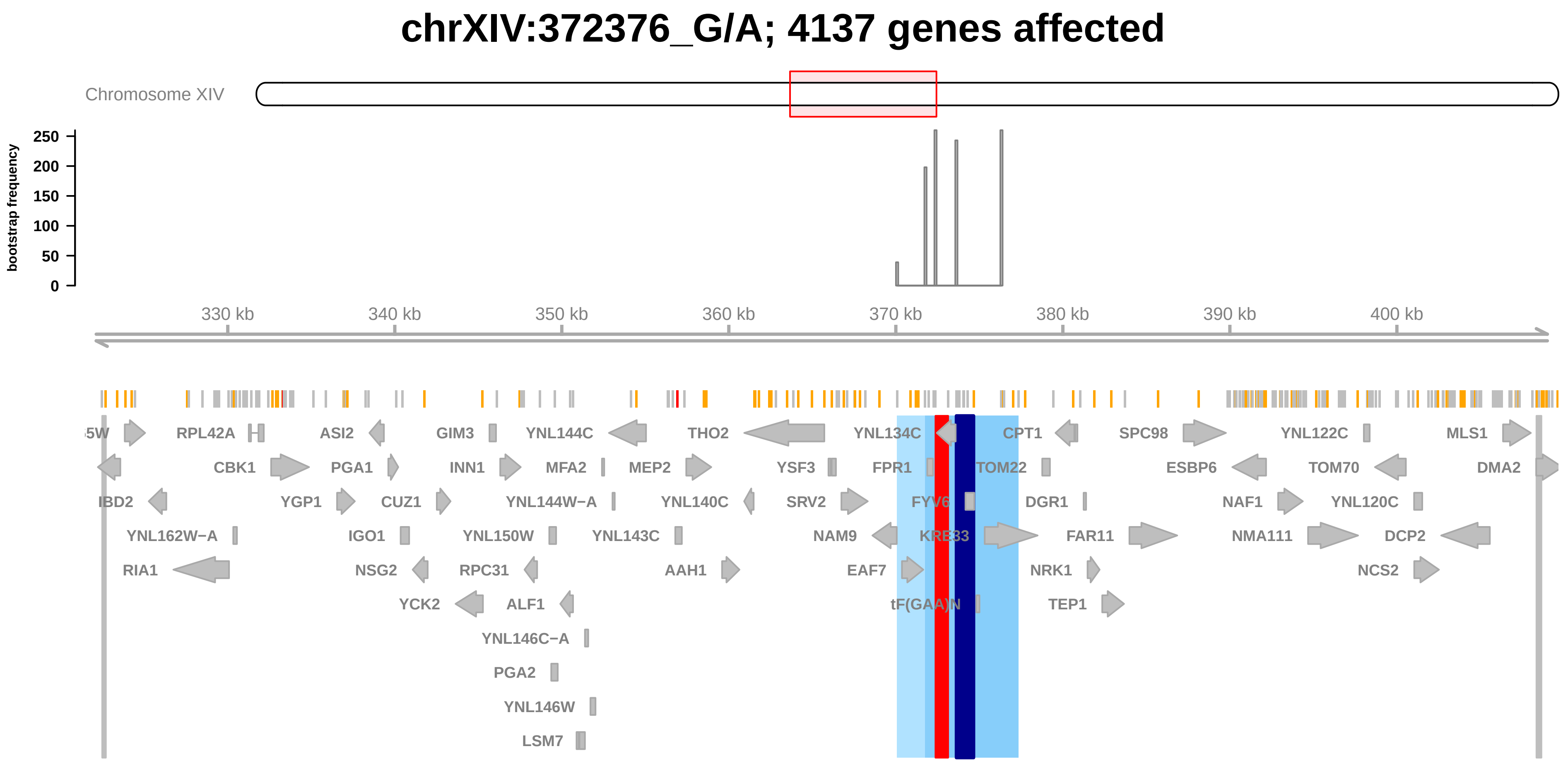
chrXIV:372376_G/A; 4137 genes affected
Chromosome XIV
bootstrap frequency
250
200
150
100
50
0
330 kb
340 kb
350 kb
360 kb
370 kb
380 kb
390 kb
400 kb
RPL42A
ASI2
GIM3
YNL144C
THO2
YNL134C
CPT1
SPC98
YNL122C
MLS1
CBK1
PGA1
INN1
MFA2
MEP2
YSF3
FPR1
TOM22
ESBP6
TOM70
DMA2
IBD2
YGP1
CUZ1
YNL144W−A
YNL140C
SRV2
FYV6
DGR1
NAF1
YNL120C
YNL162W−A
IGO1
YNL150W
YNL143C
NAM9
KRE33
FAR11
NMA111
DCP2
RIA1
NSG2
RPC31
AAH1
EAF7
NRK1
NCS2
YCK2
ALF1
tF(GAA)N
TEP1
YNL146C−A
PGA2
YNL146W
LSM7

chrXIV:466588_T/G; 4550 genes affected

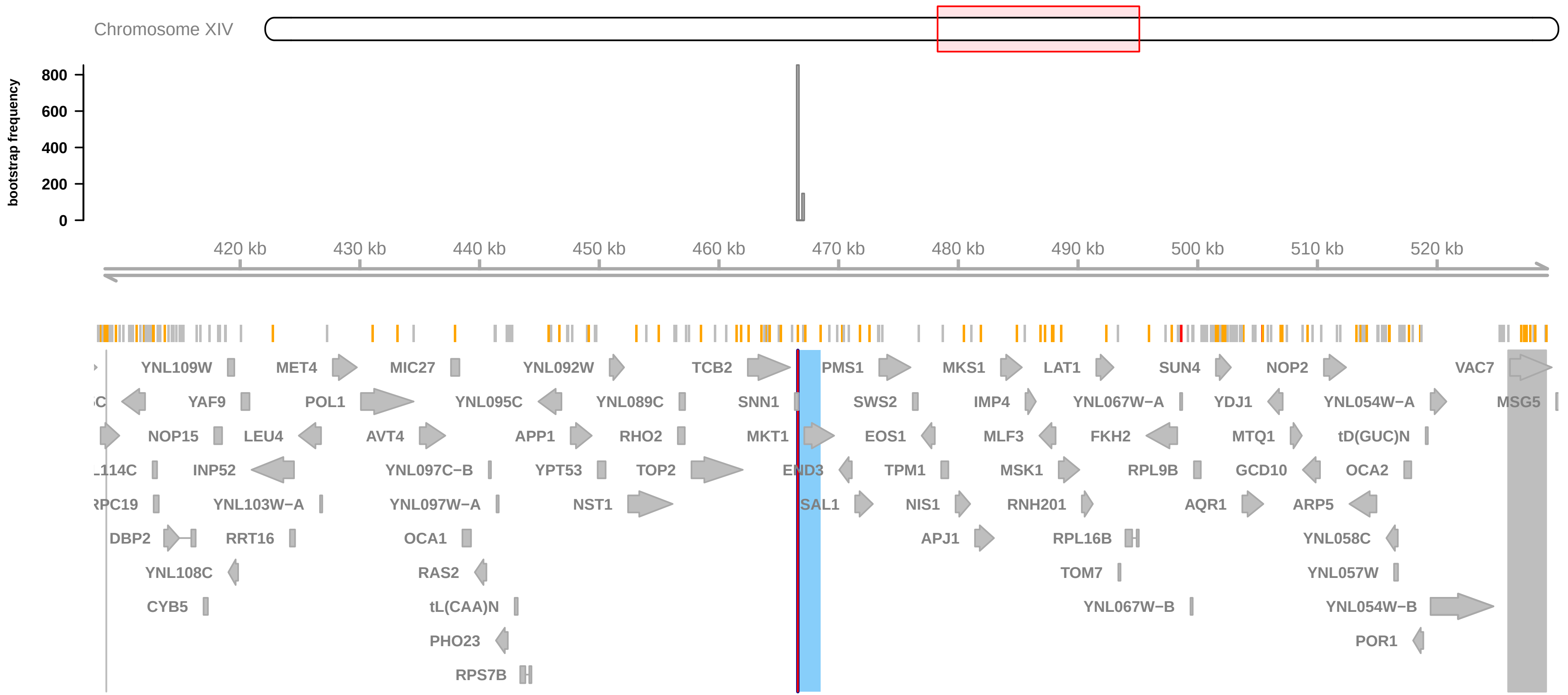

chrXIV:579945_GTTA/G; 1967 genes affected

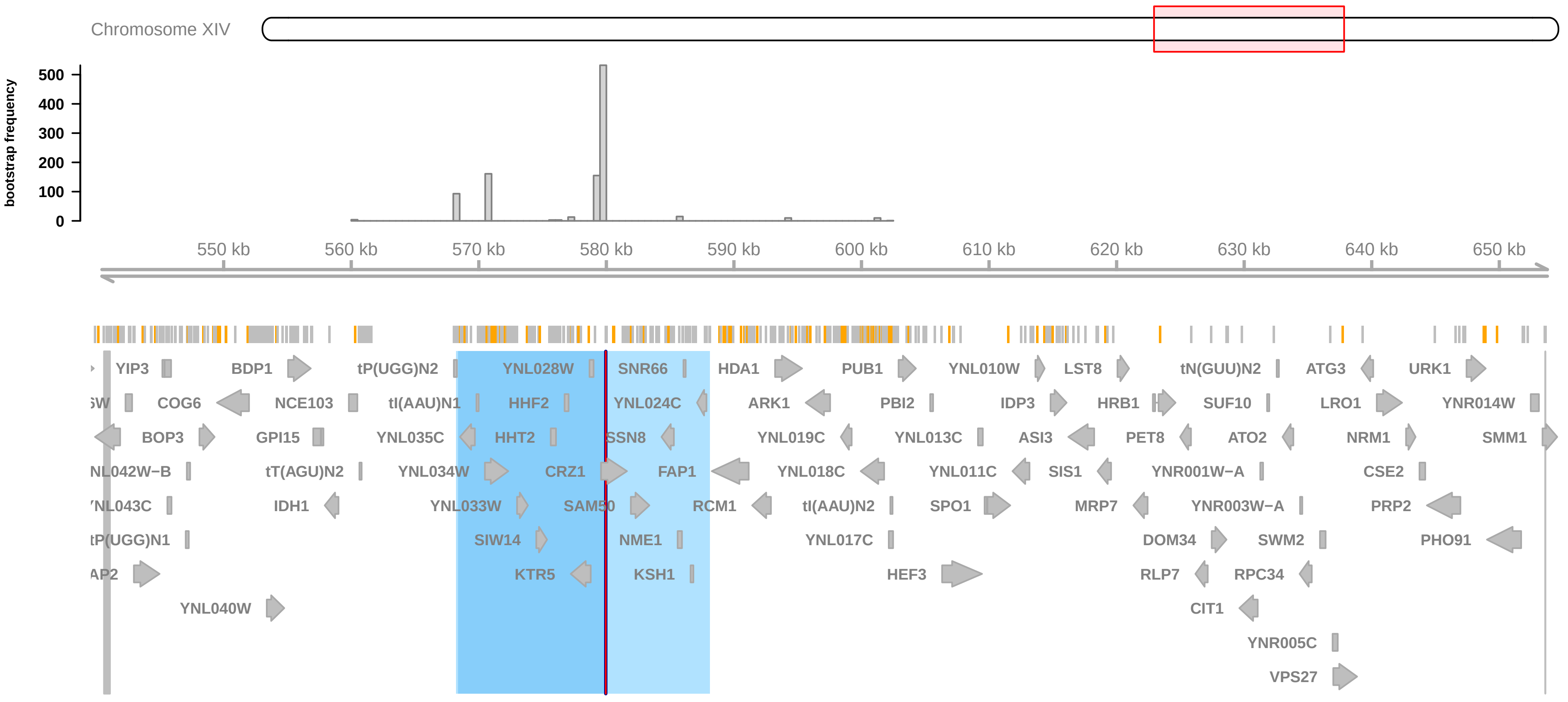

chrXV:56600_G/C; 1083 genes affected

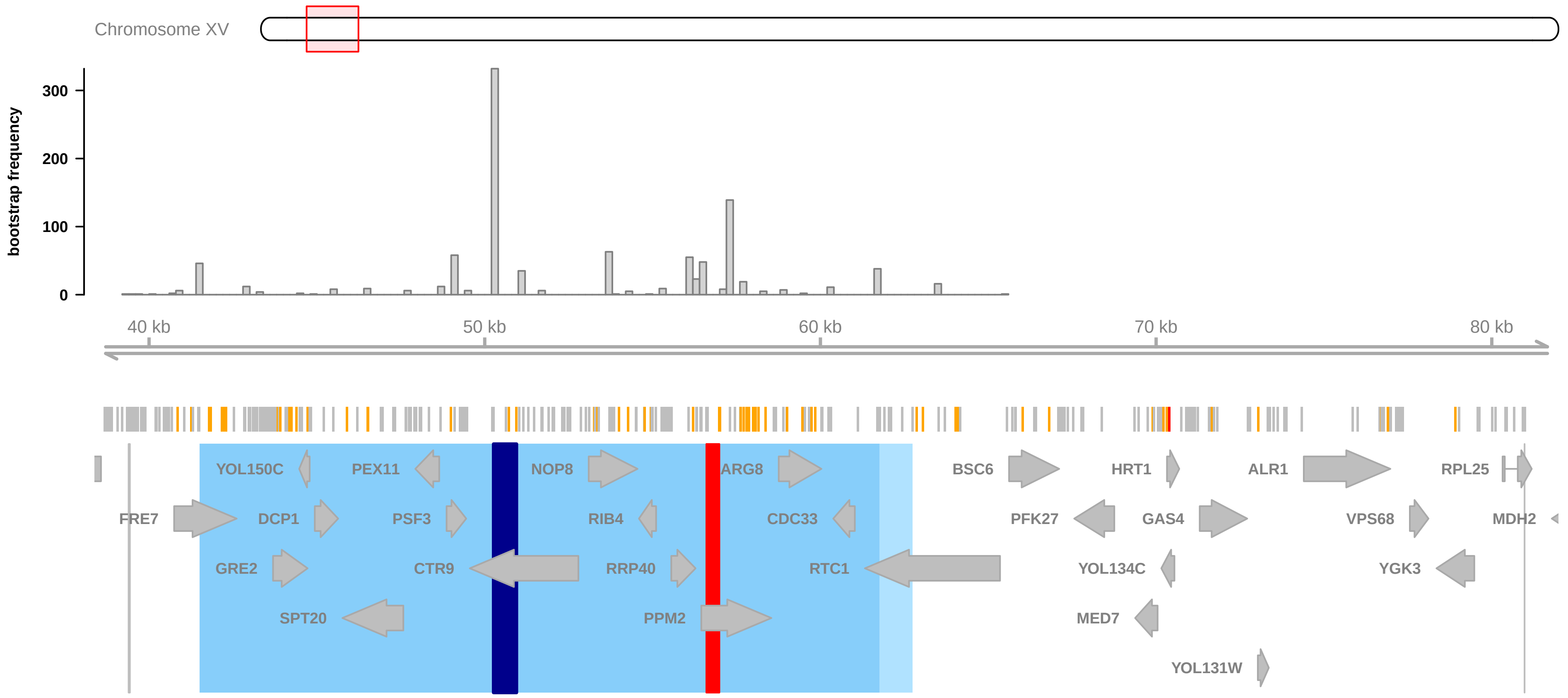

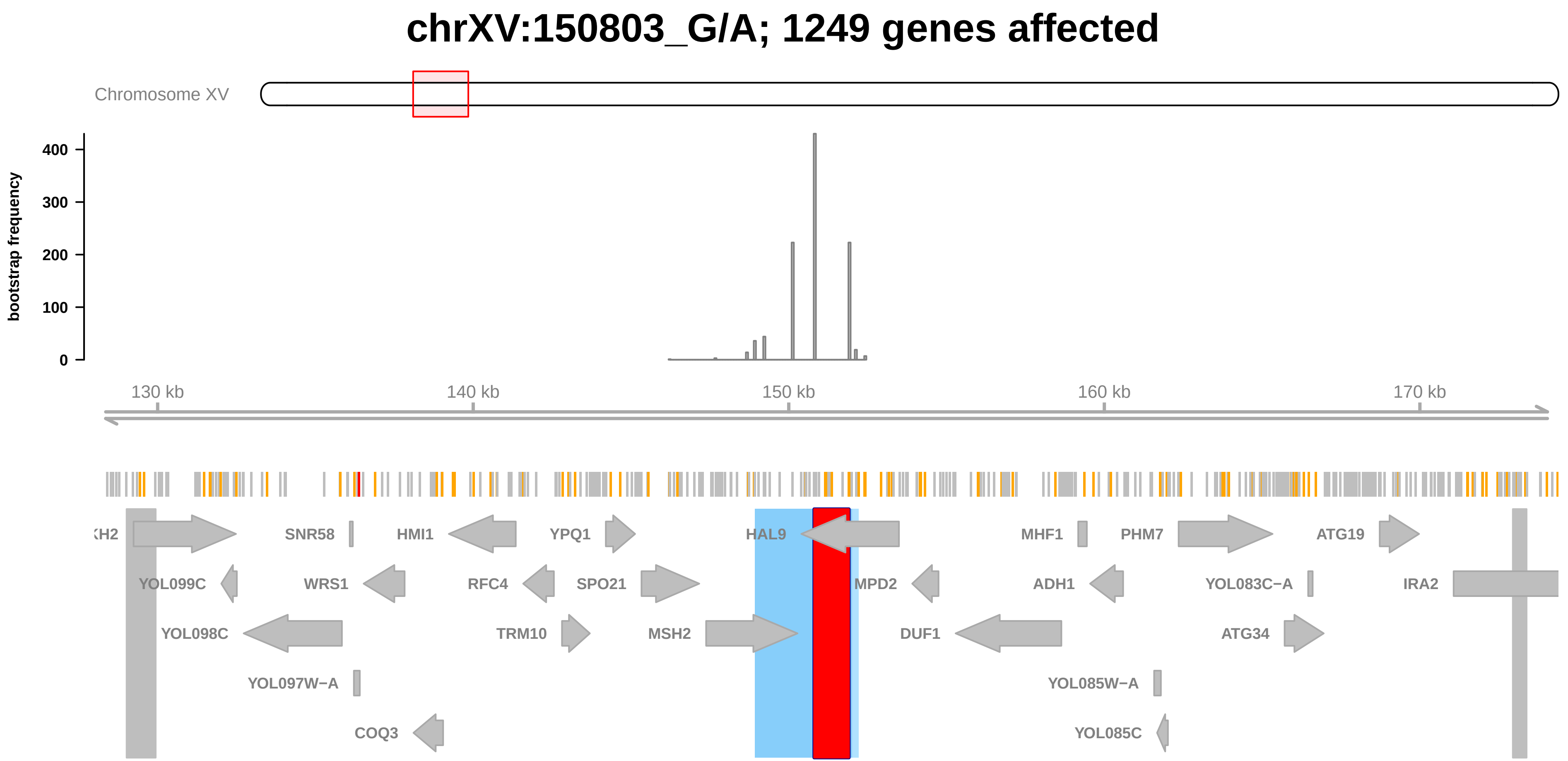

chrXV:150803_G/A; 1249 genes affected
Chromosome XV
bootstrap frequency
400
300
200
100
0
130 kb
140 kb
150 kb
160 kb
170 kb
SNR58
HMI1
YPQ1
HAL9
MHF1
PHM7
ATG19
YOL099C
WRS1
RFC4
SPO21
MPD2
ADH1
YOL083C−A
IRA2
YOL098C
TRM10
MSH2
DUF1
ATG34
YOL097W−A
YOL085W−A
COQ3
YOL085C

chrXV:171150_T/C; 1504 genes affected

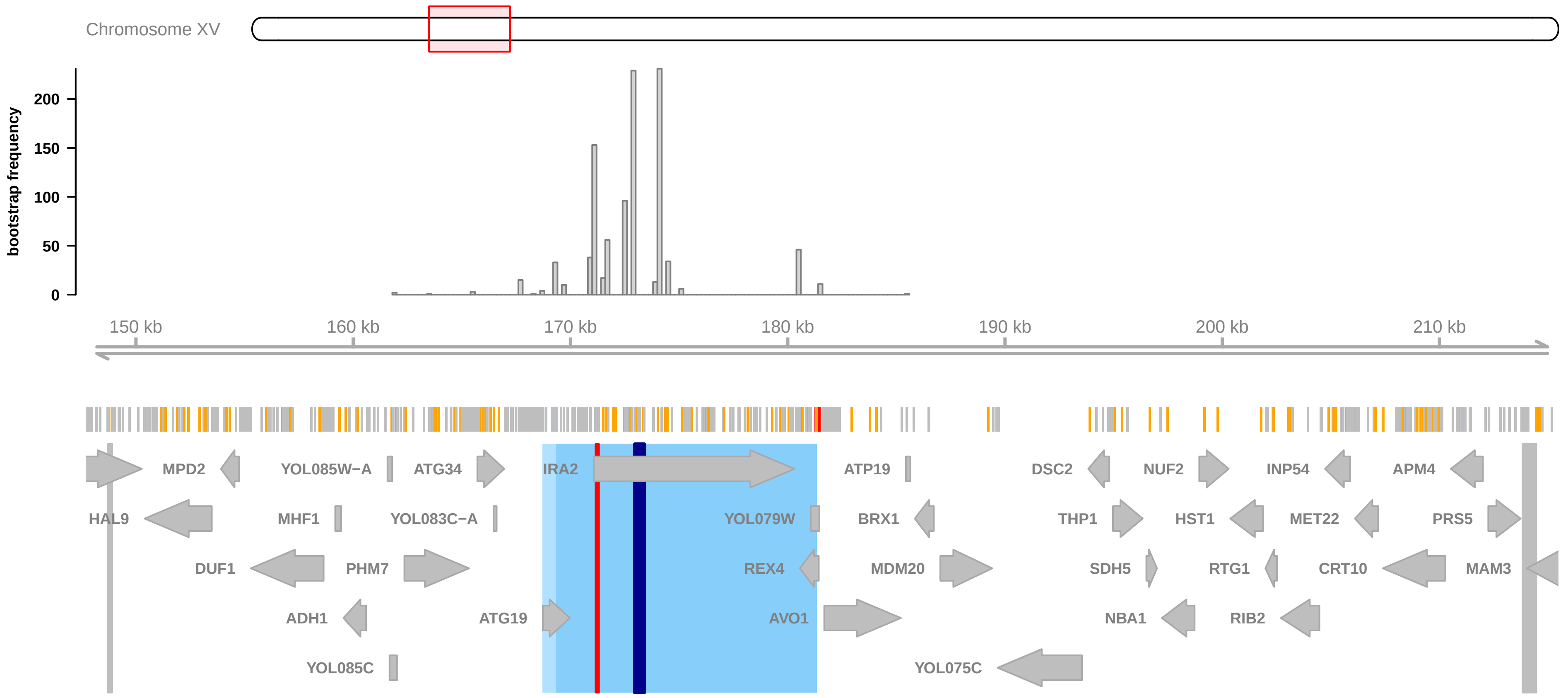

chrXV:377605_C/T; 1138 genes affected

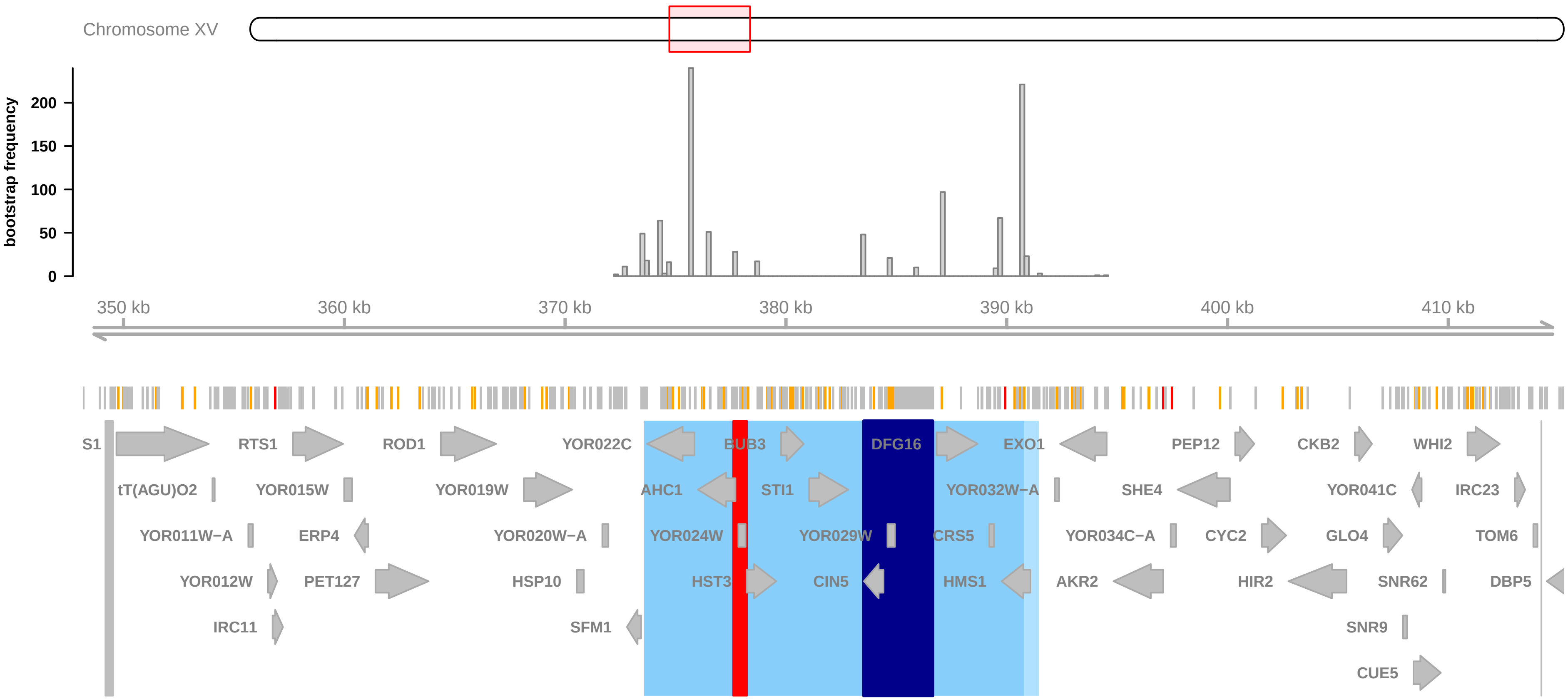

chrXV:521864_C/T; 1414 genes affected

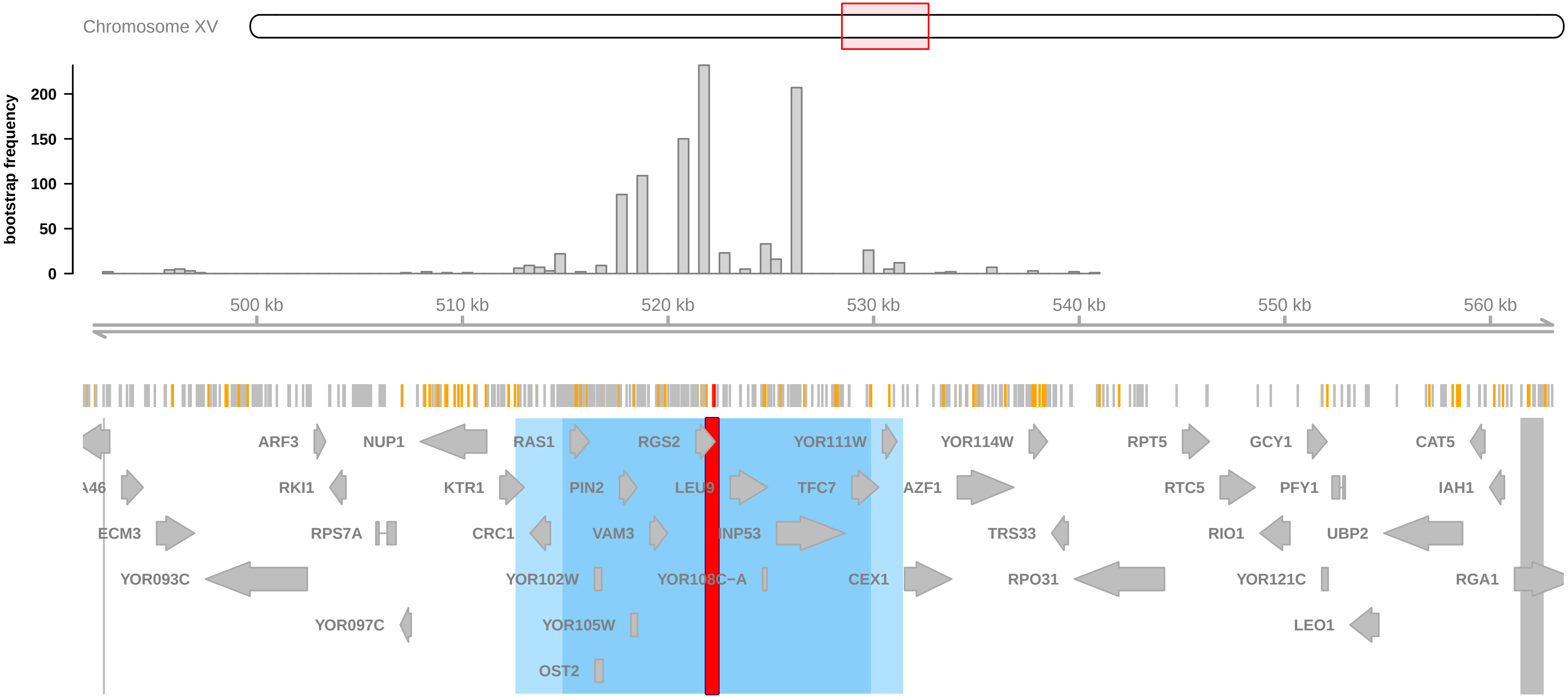

chrXV:589481_C/T; 1019 genes affected

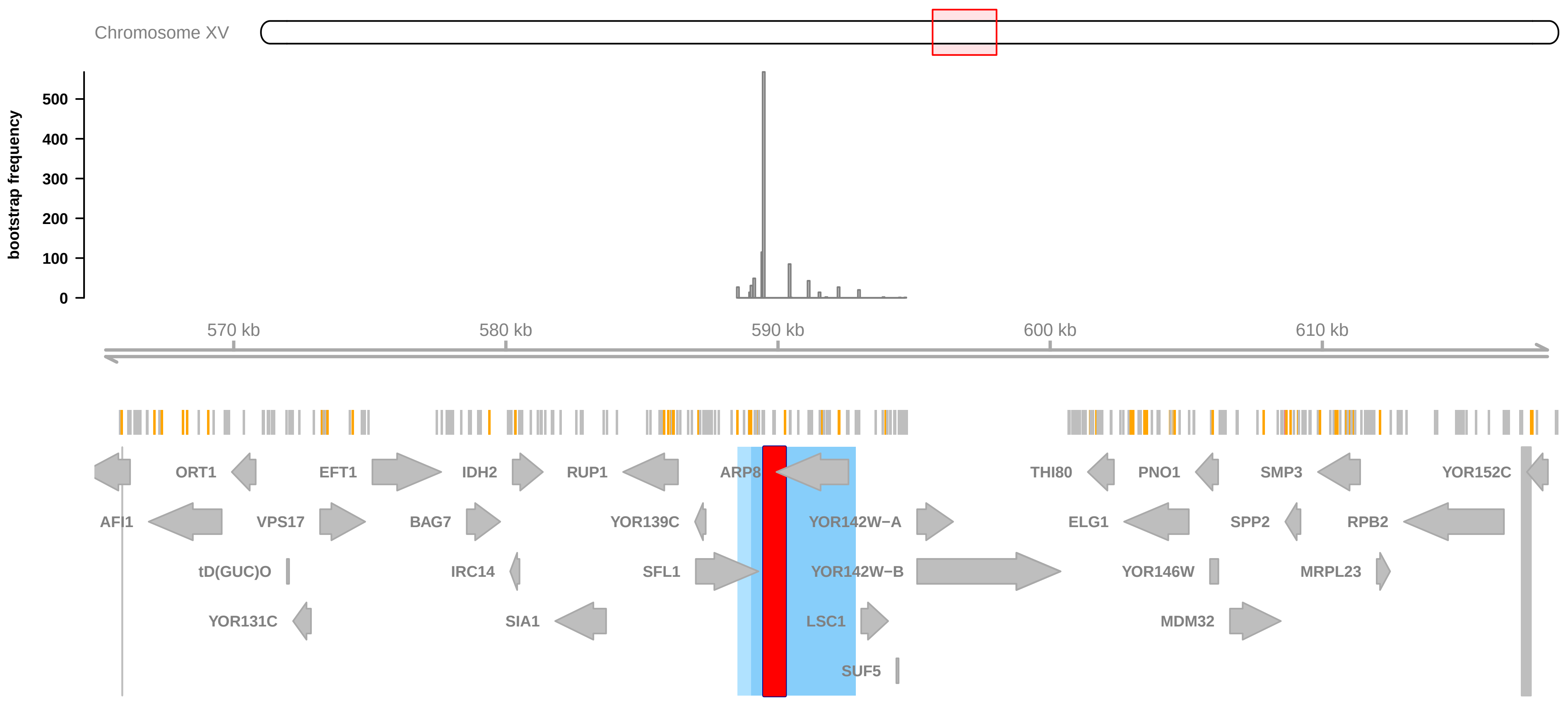

chrXV:725113_T/C; 822 genes affected

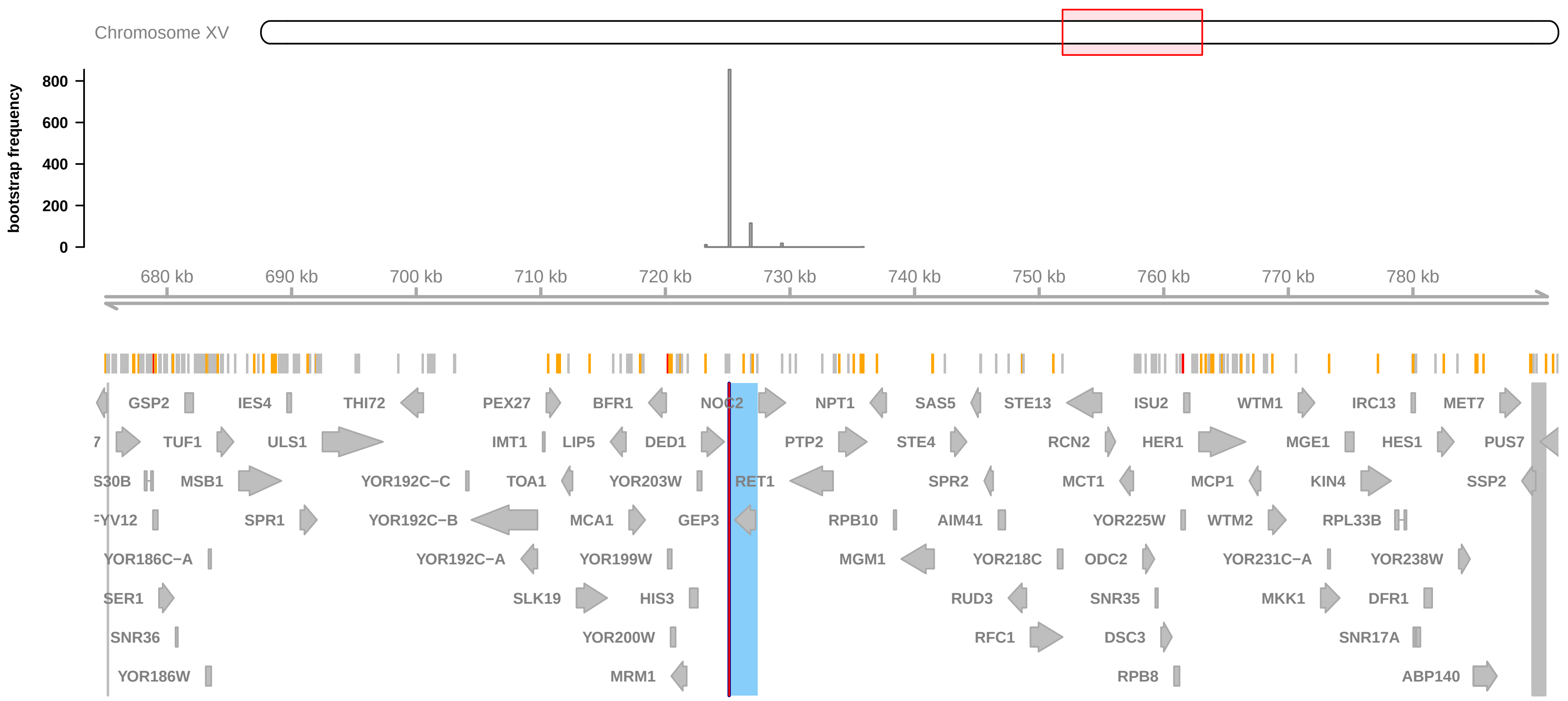

chrXV:845199_G/A; 832 genes affected

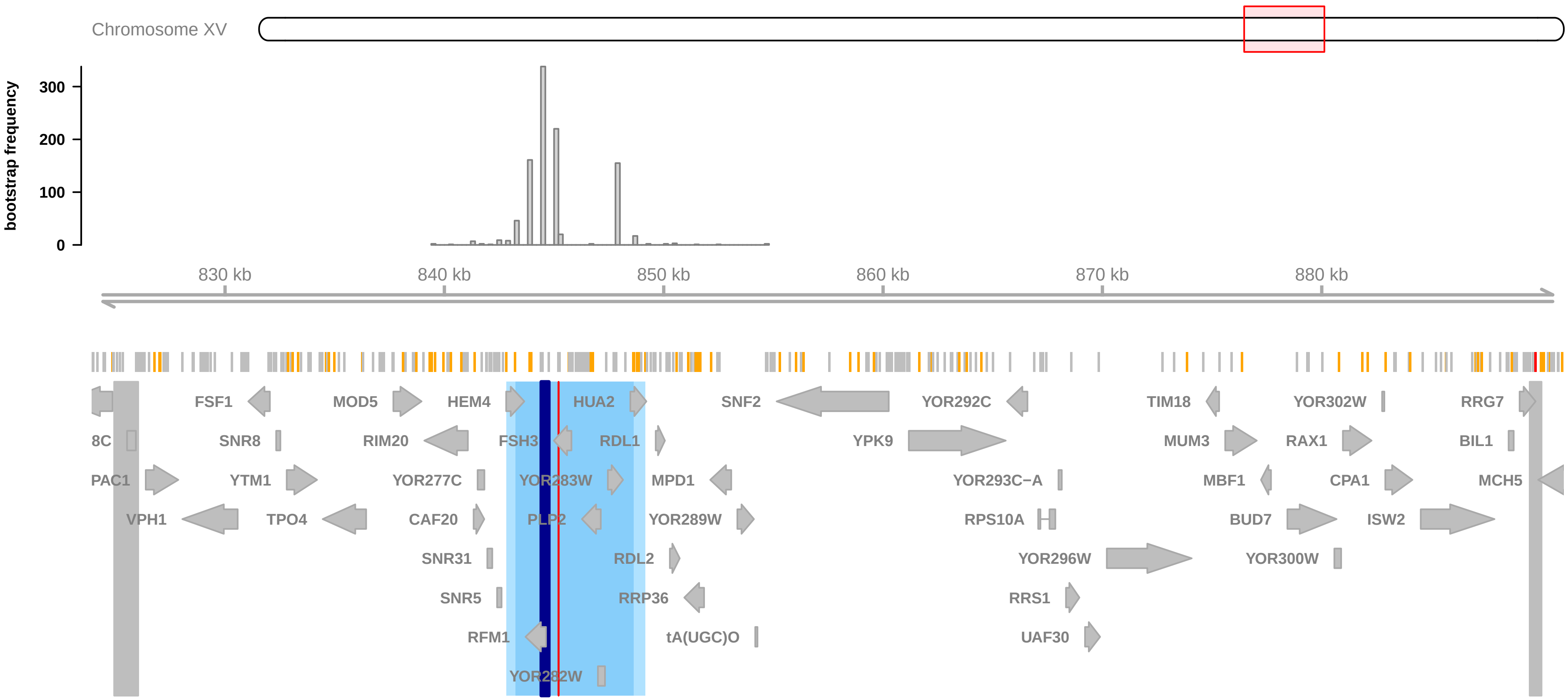

chrXV:958080_T/A; 593 genes affected

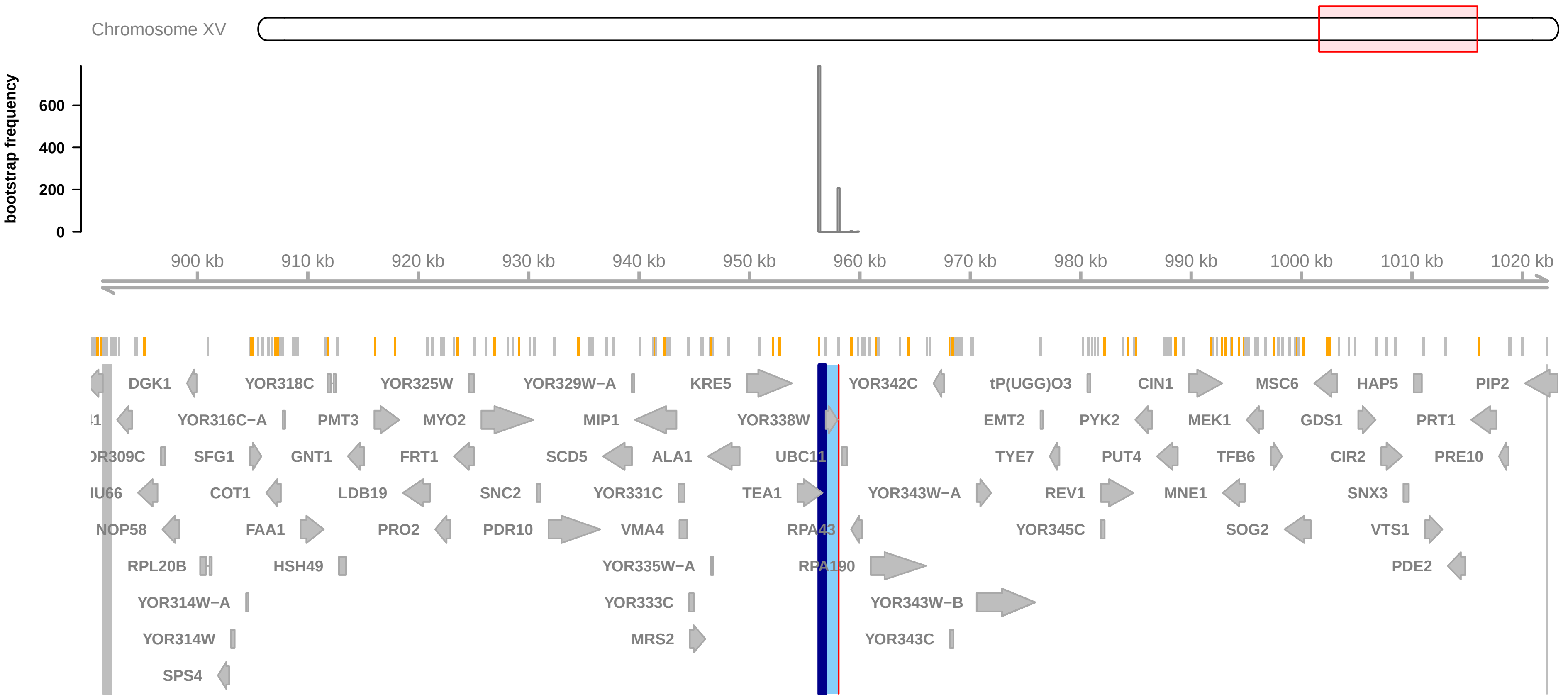

chrXV:1065815_A/G; 470 genes affected

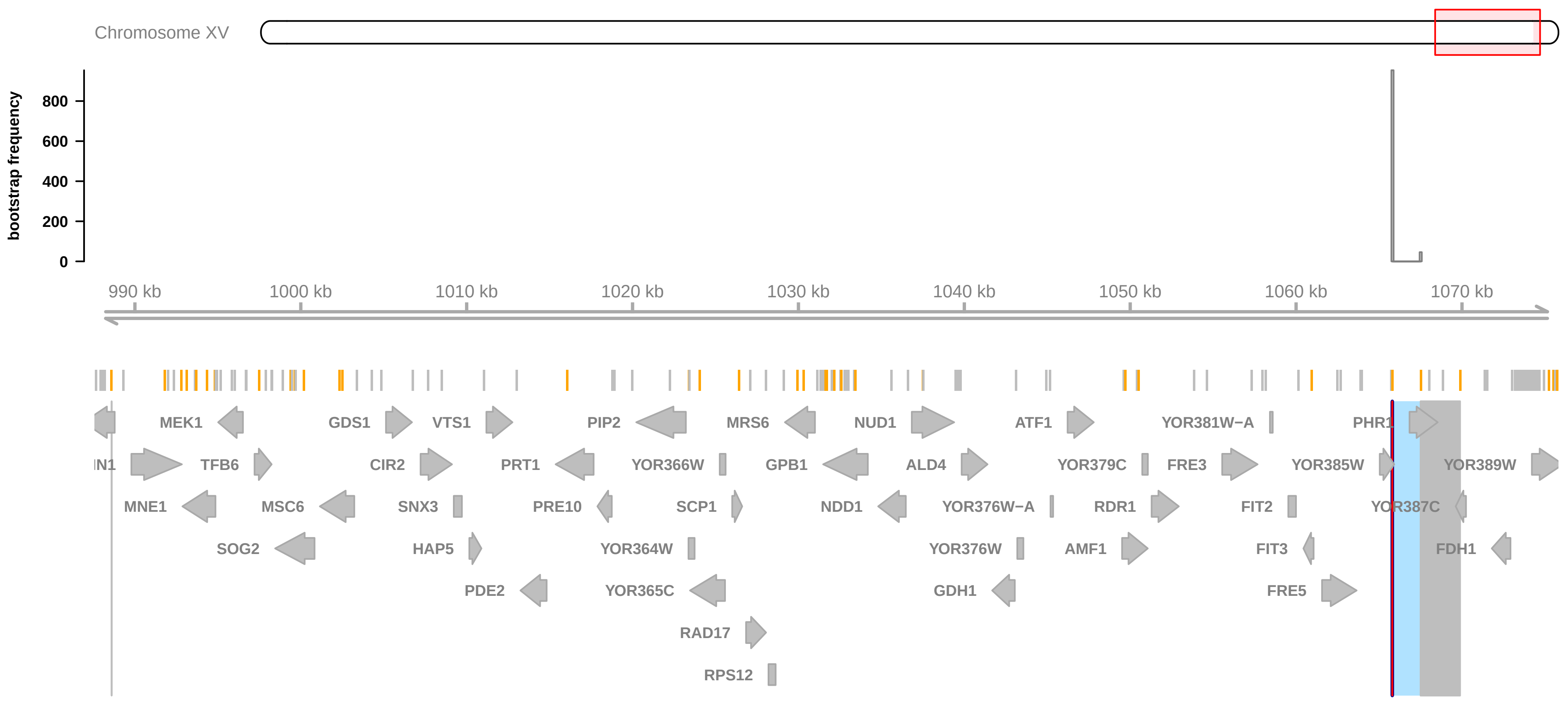

chrXVI:26510_T/C; 149 genes affected

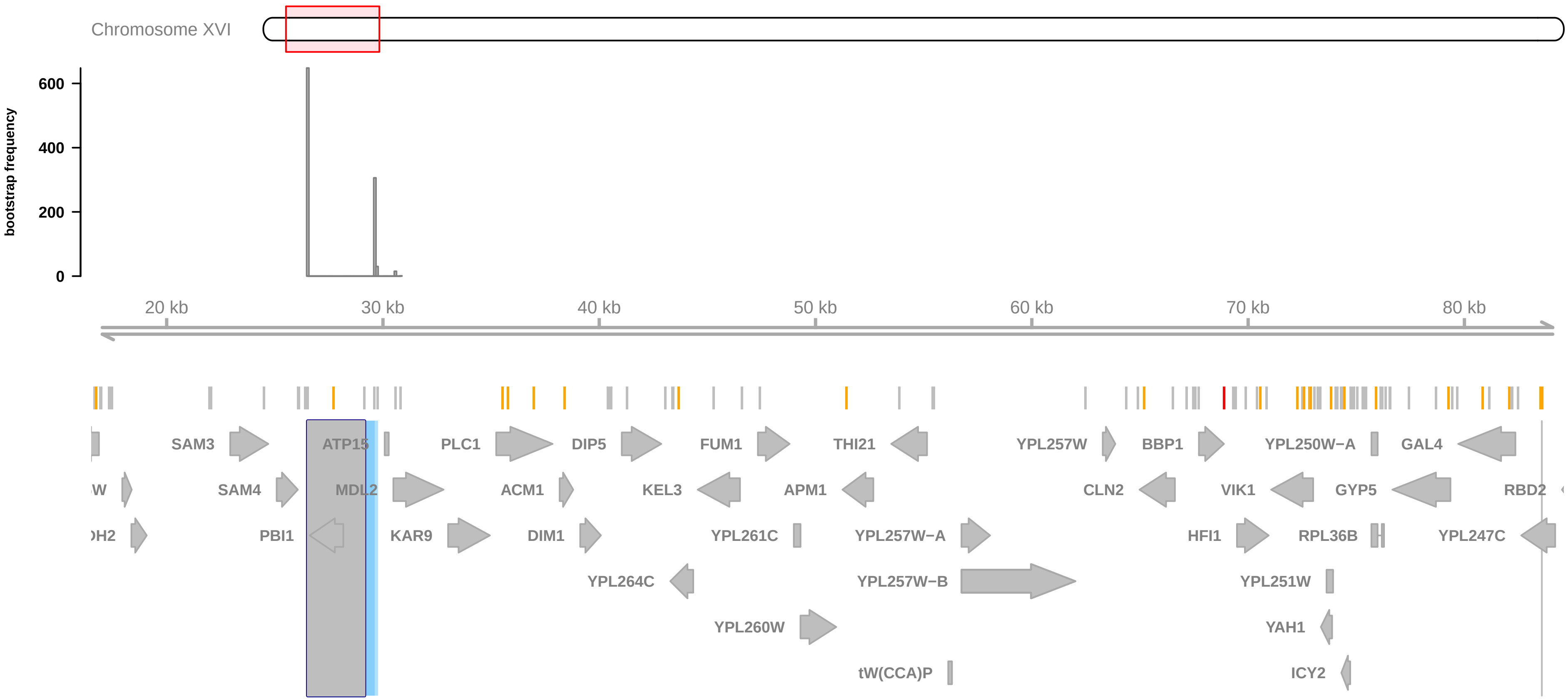

chrXVI:84945_G/A; 399 genes affected

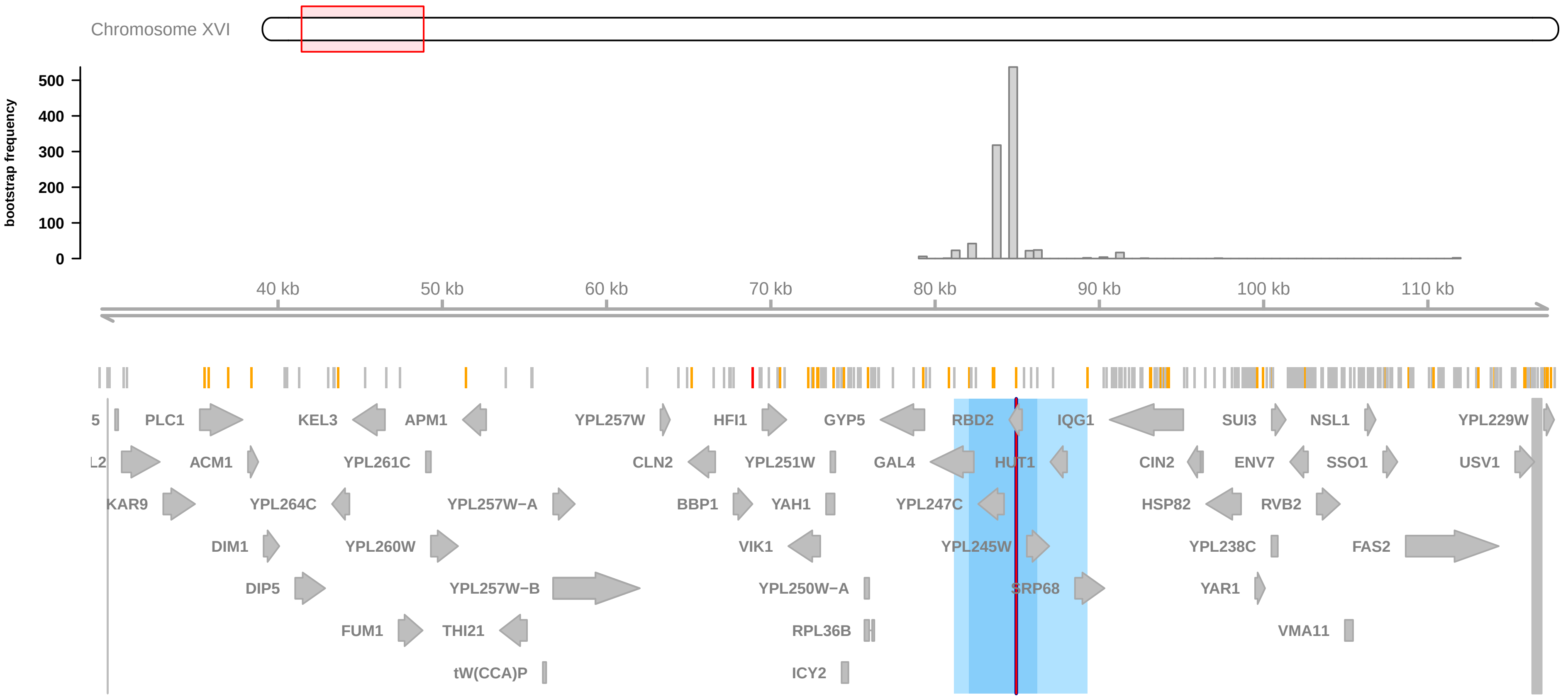

chrXVI:207364_C/T; 471 genes affected

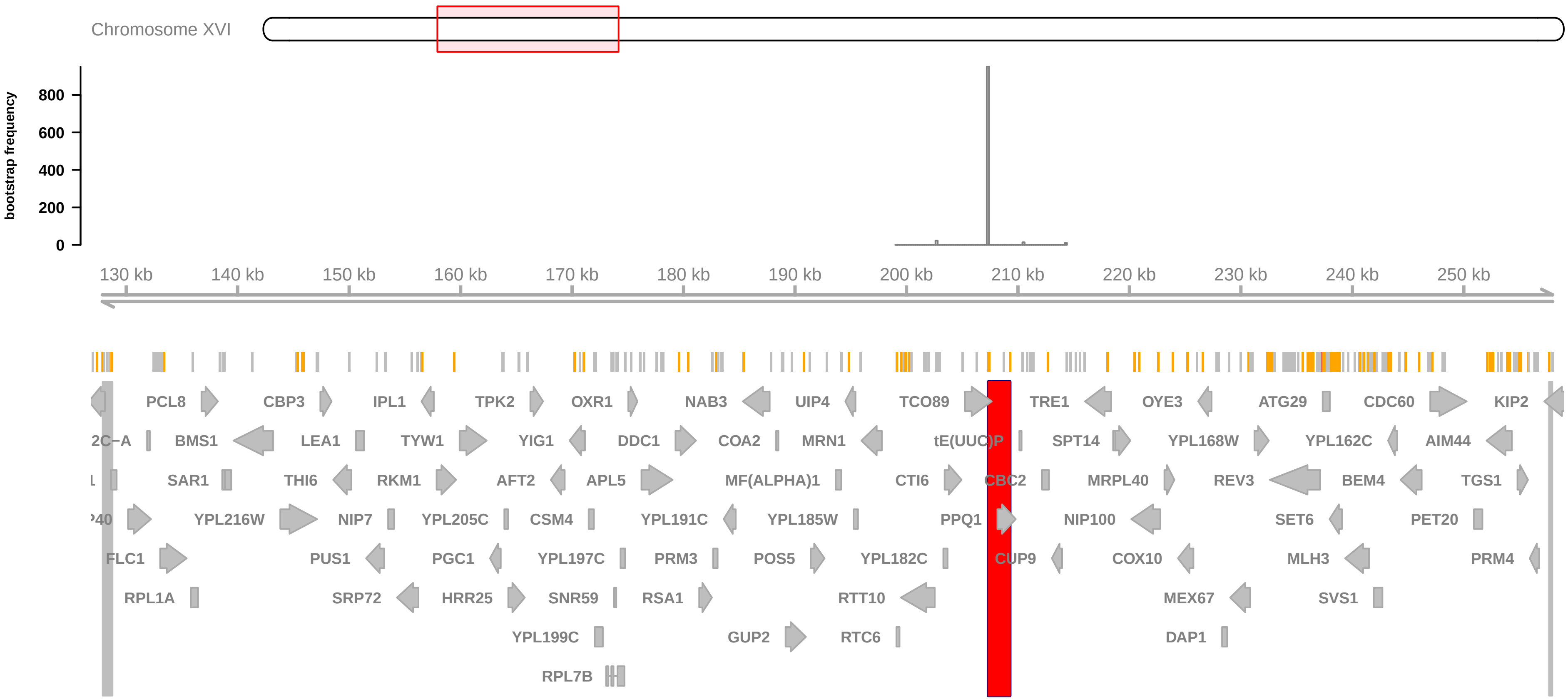

chrXVI:383928_G/A; 193 genes affected

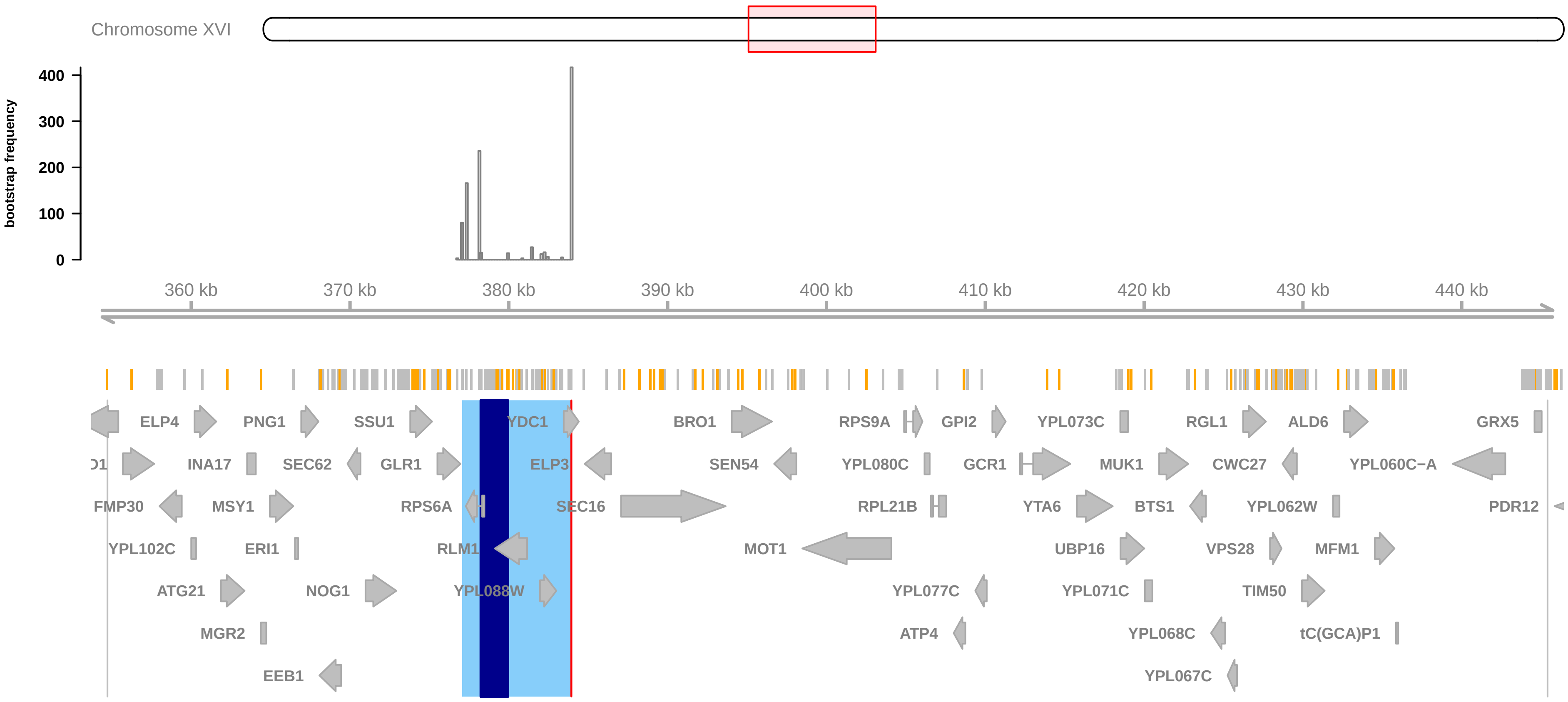

chrXVI:463543_G/A; 371 genes affected

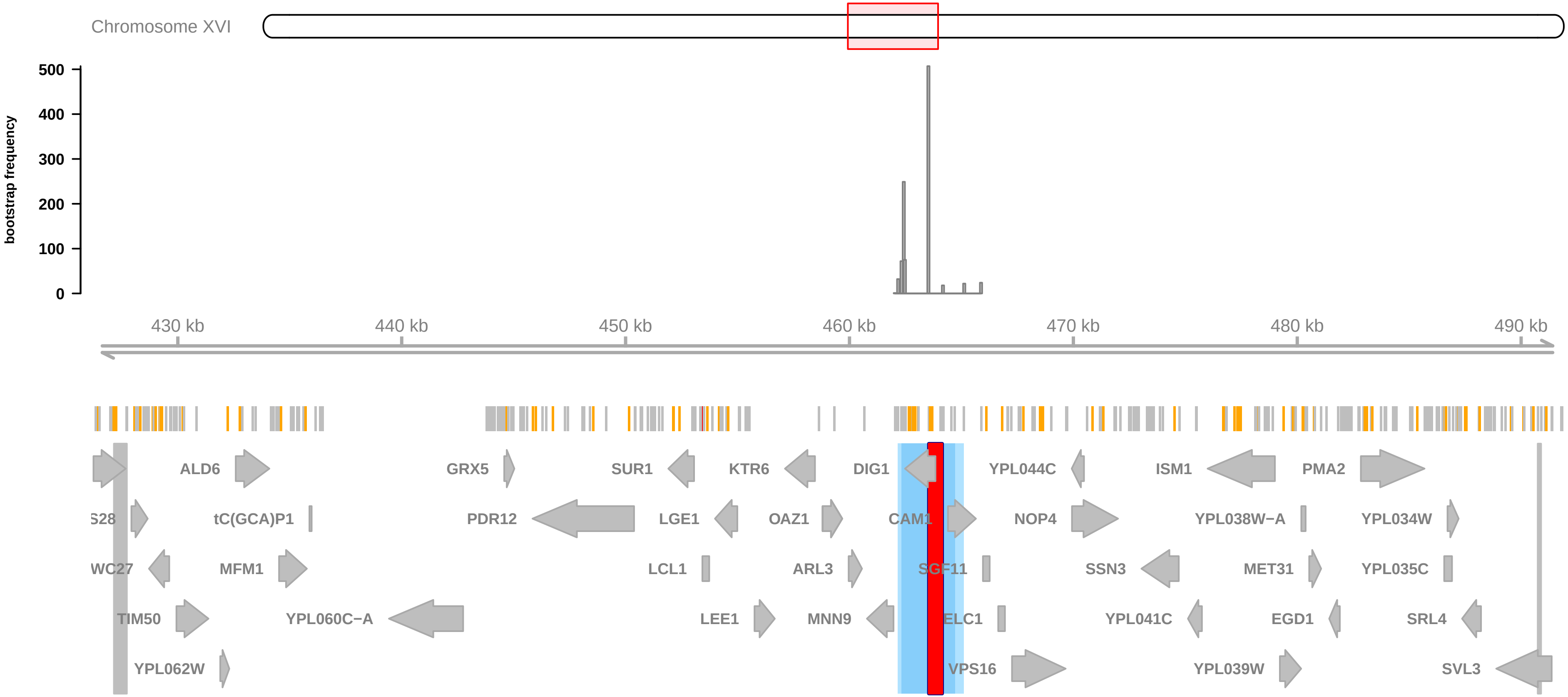

chrXVI:508199_C/T; 578 genes affected

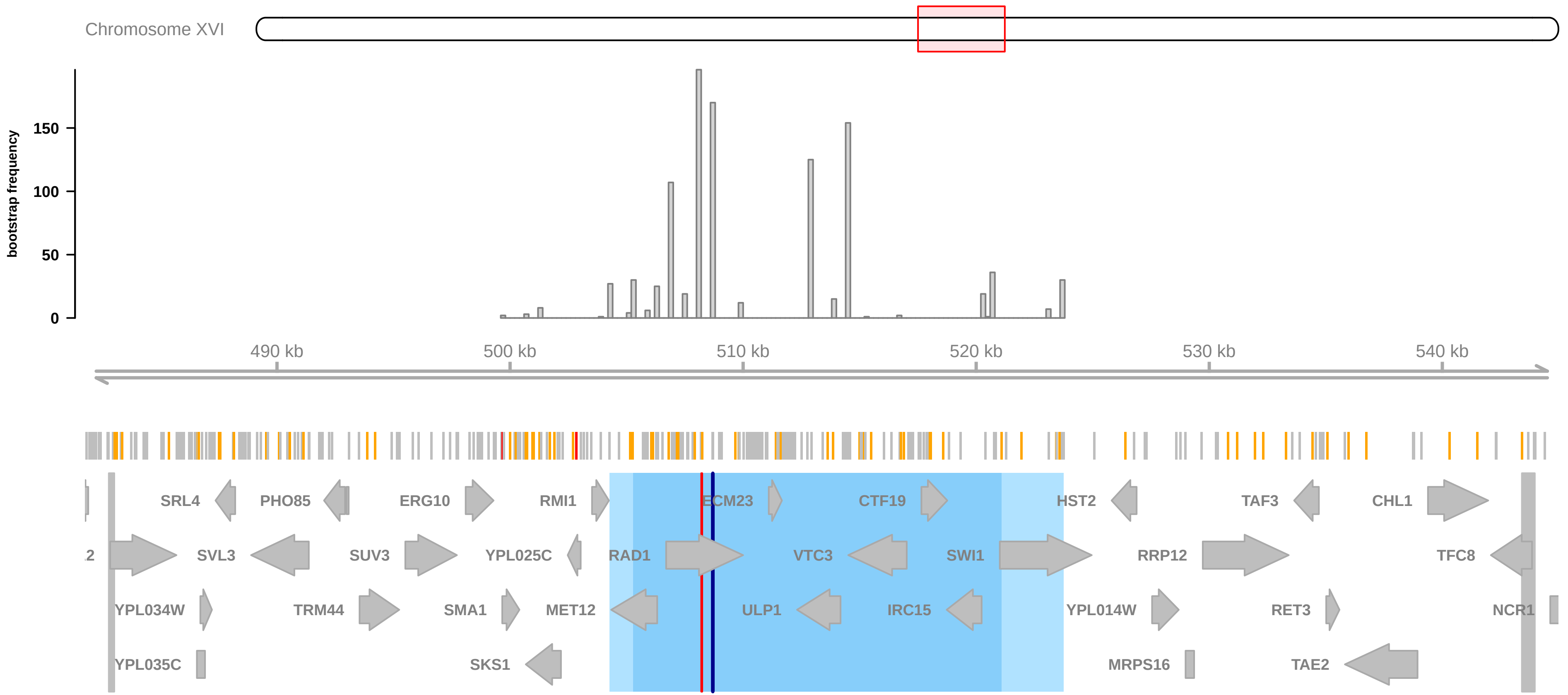

chrXVI:812990_T/C; 185 genes affected

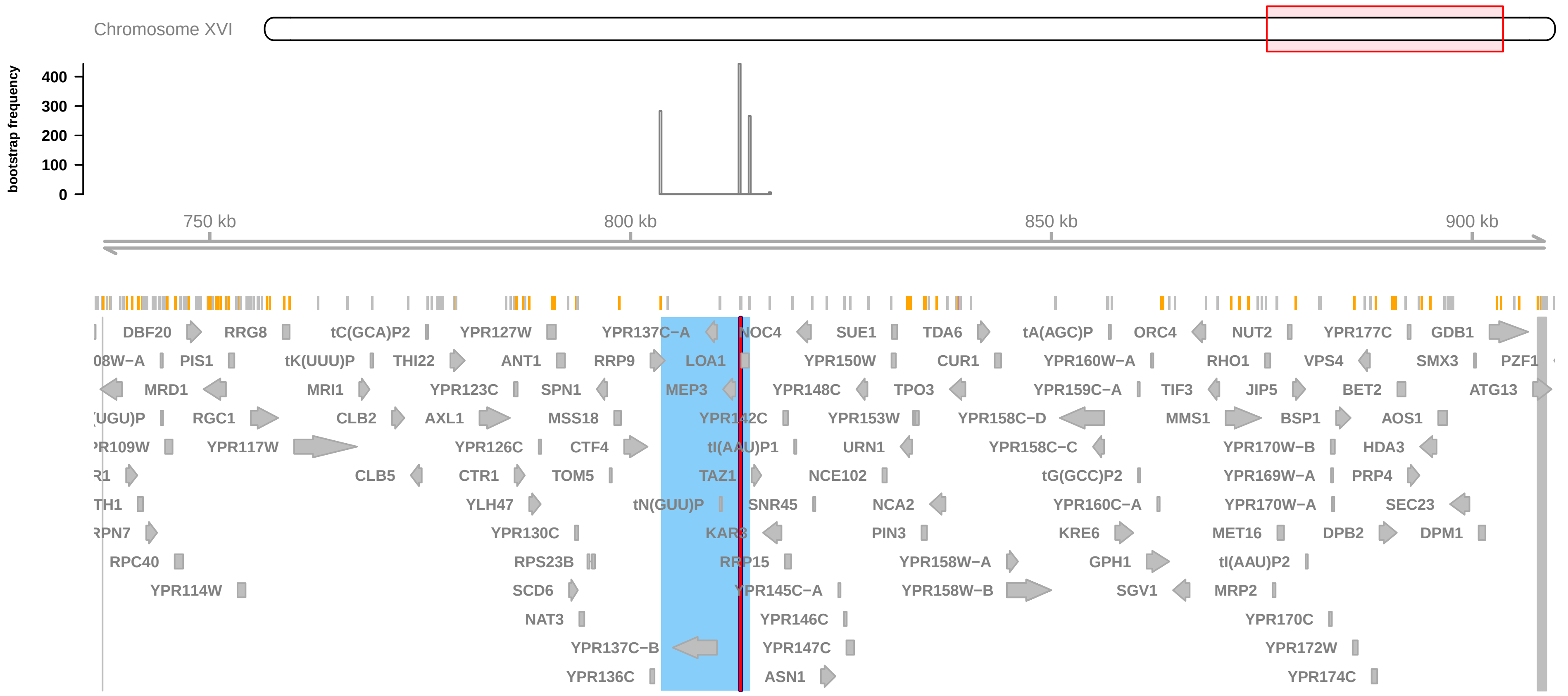

Supplement: Supplementary file 5. — This pdf file contains one page per hotspot region, showing the position, bootstrap distribution, variant and gene content for each hotspot. See legend of Figure 4 and Figure 4—figure supplement 4 for further details. [file elife-35471-supp5.pdf]
