# Supplementary material for: Genetics of trans-regulatory variation in gene expression
Source: eLife. 2018 Jul 17;7:e35471. doi: 10.7554/eLife.35471 (PMC6072440; doi:10.7554/eLife.35471)

chrI:48890_C/T top 50 up genes

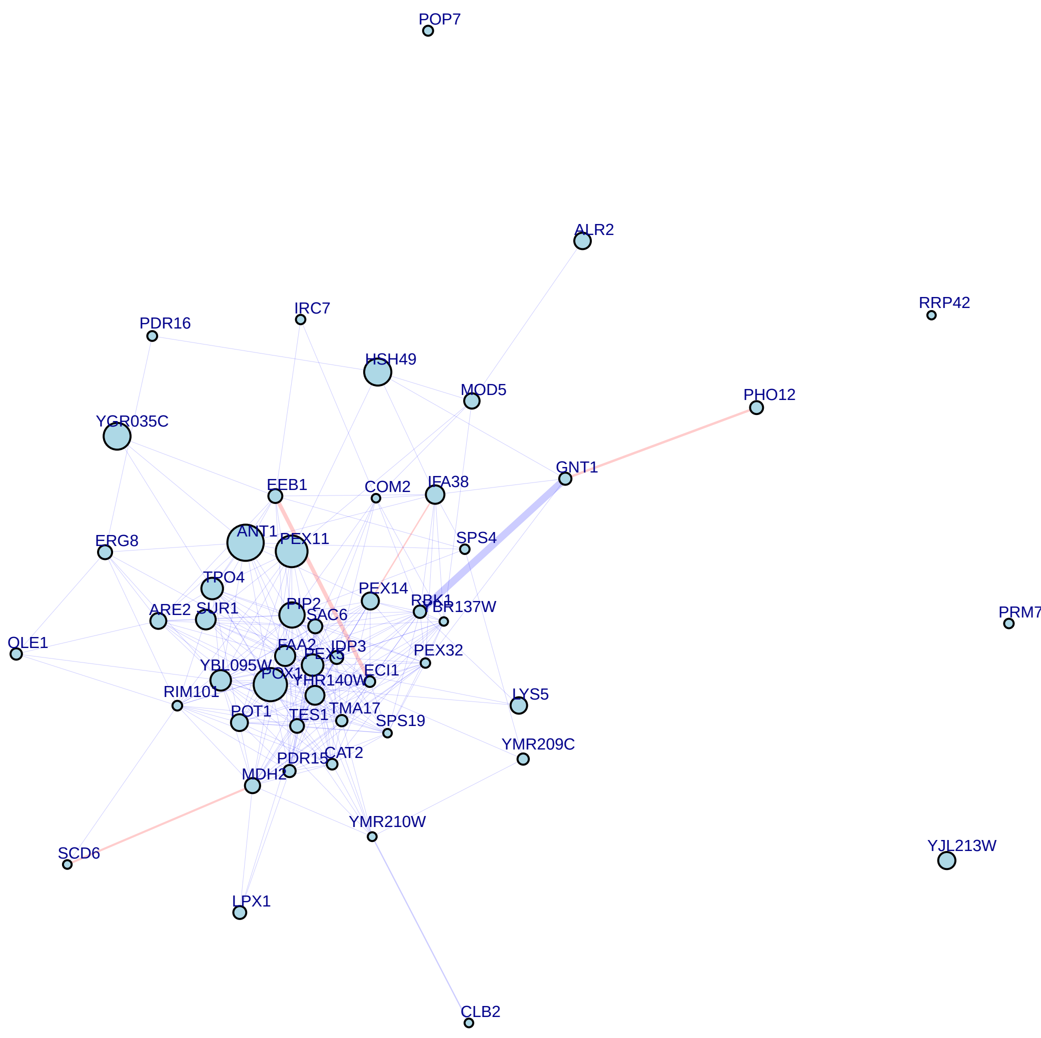

chrI:48890_C/T top 50 down genes

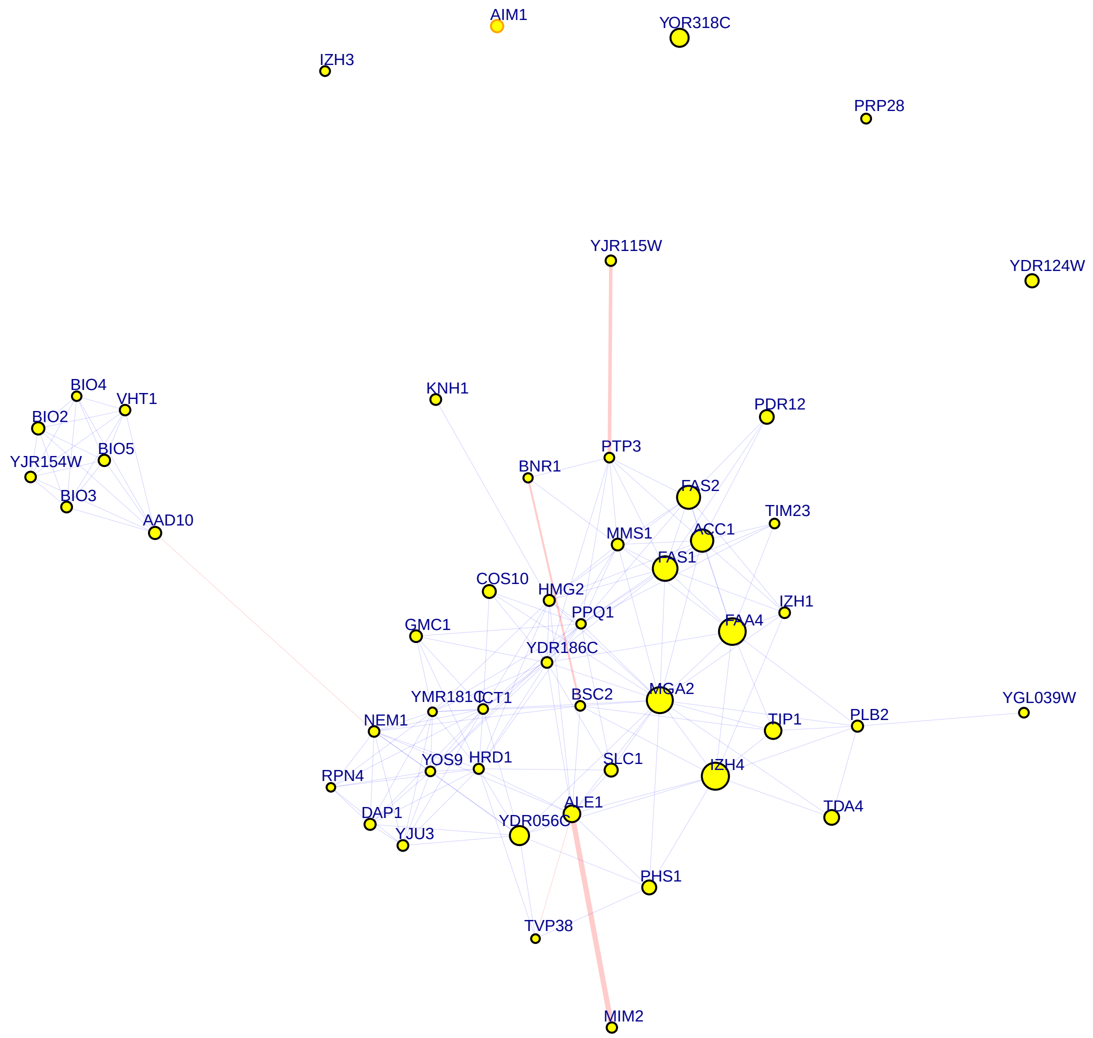

chrI:48890_C/T top 100 up and down genes

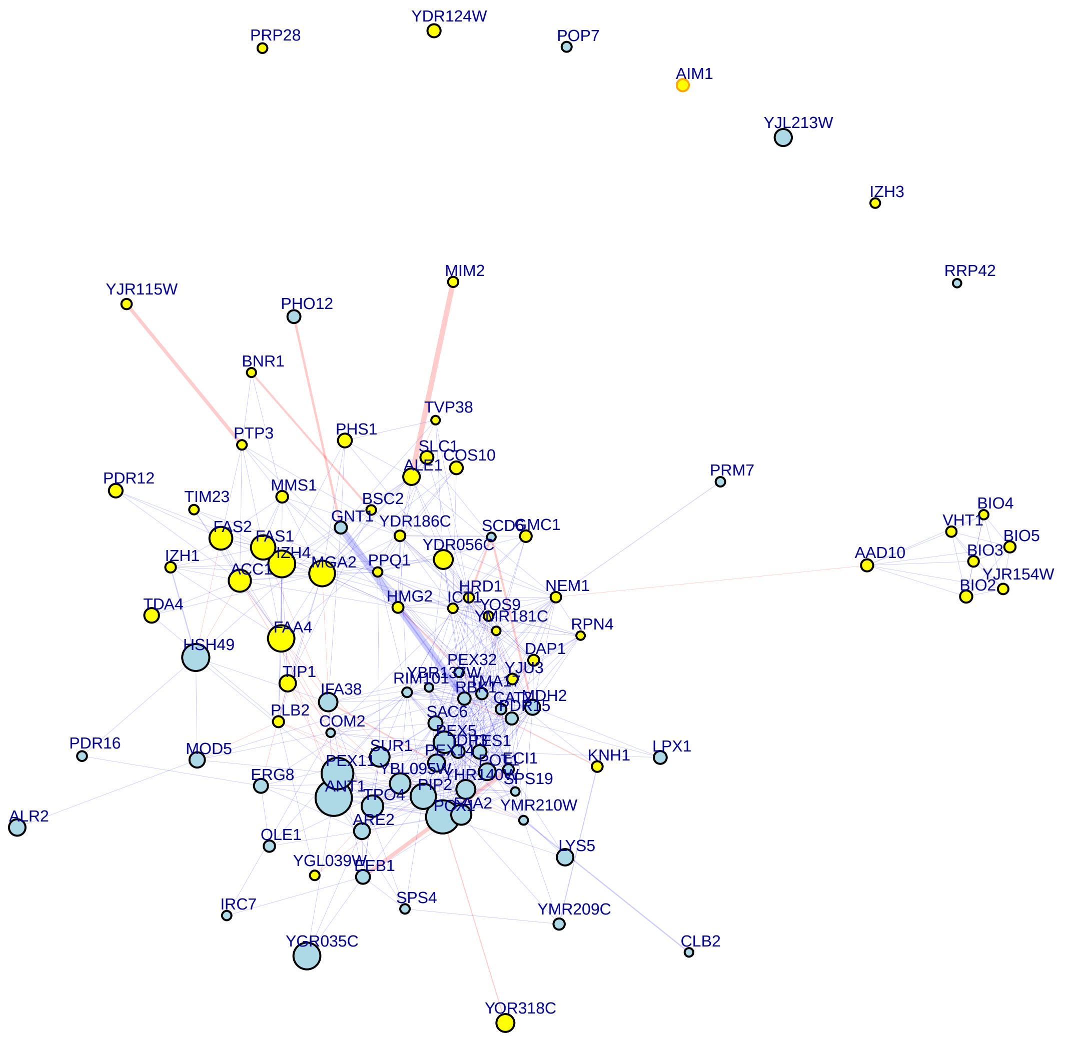

chrI:109735_A/G top 50 up genes

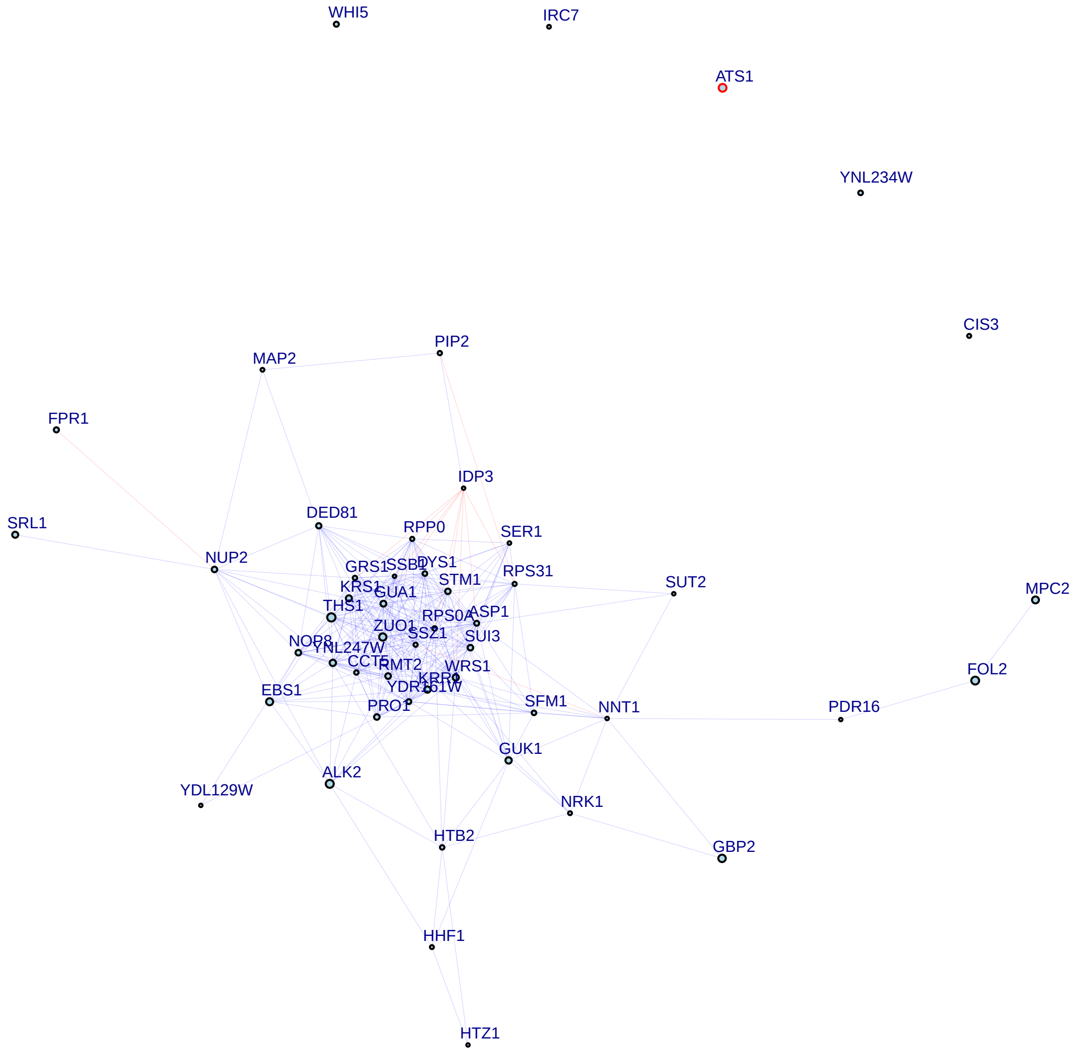

chrI:109735_A/G top 50 down genes

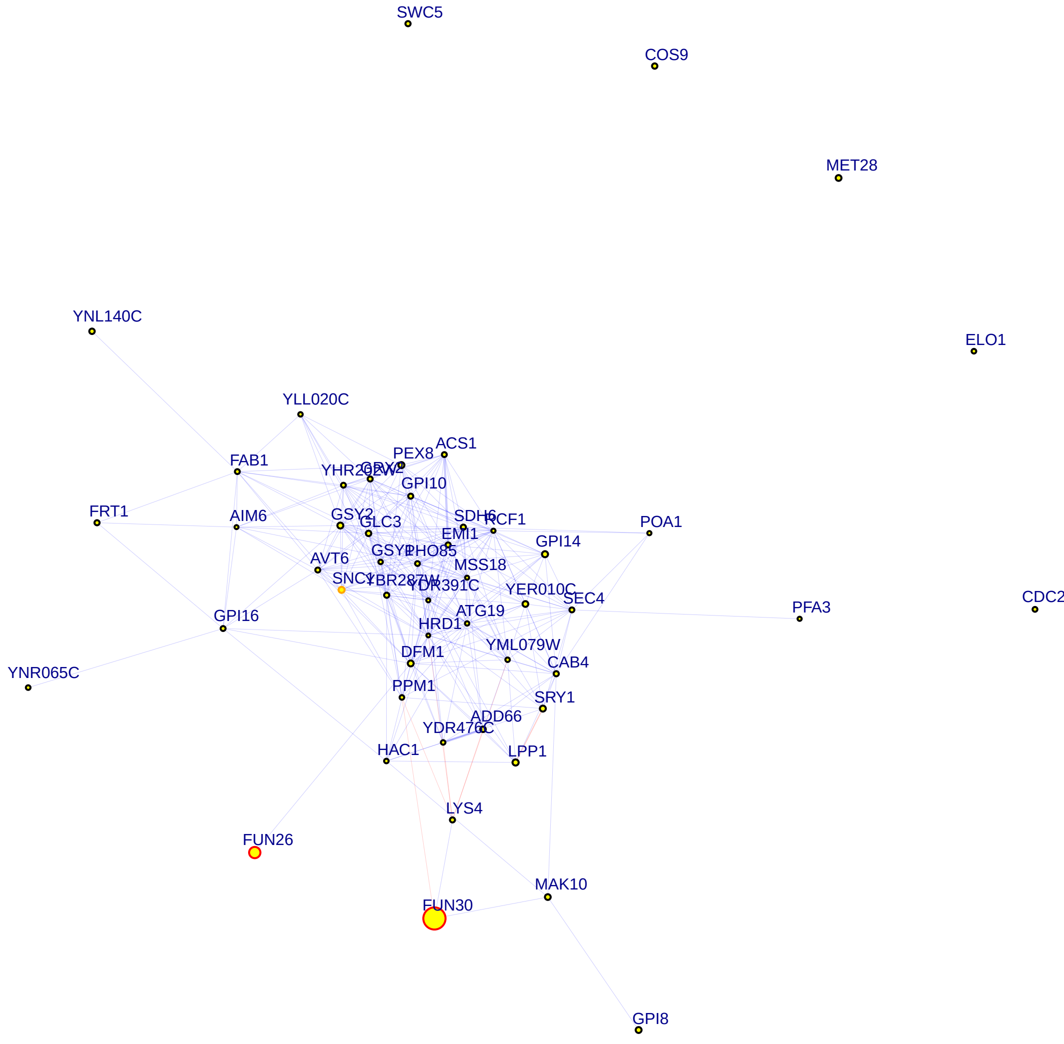

chrI:109735_A/G top 100 up and down genes

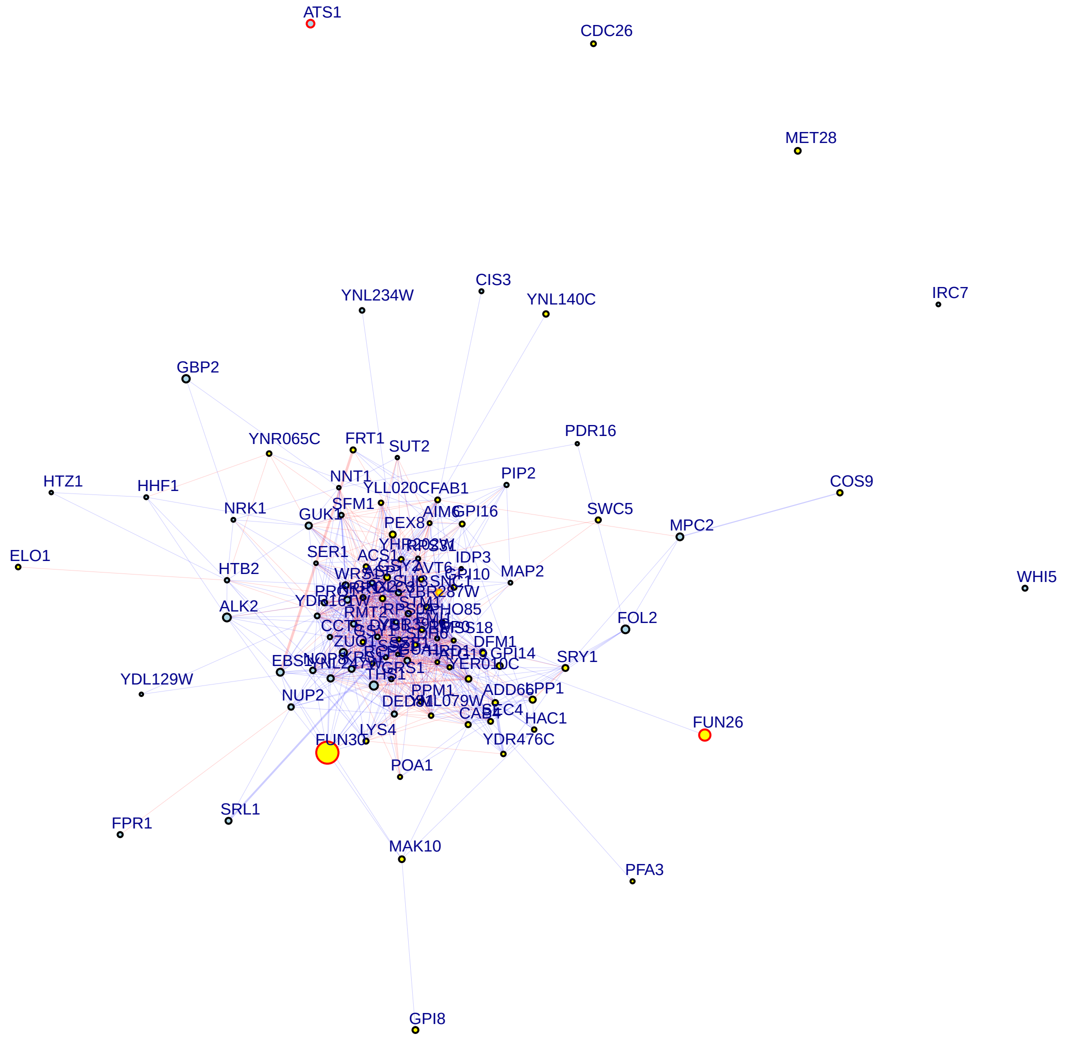

chrI:159467_G/A top 50 up genes

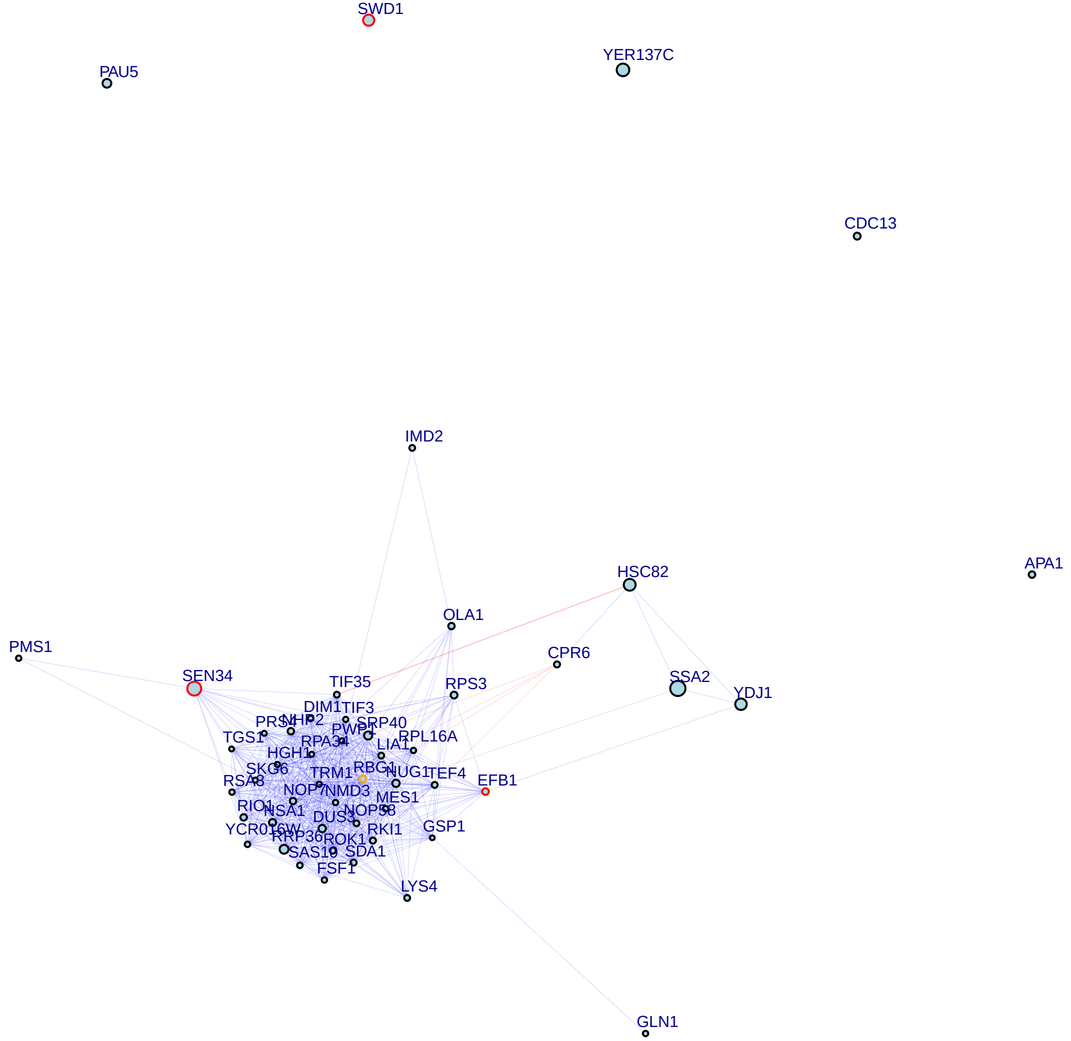

chrI:159467_G/A top 50 down genes

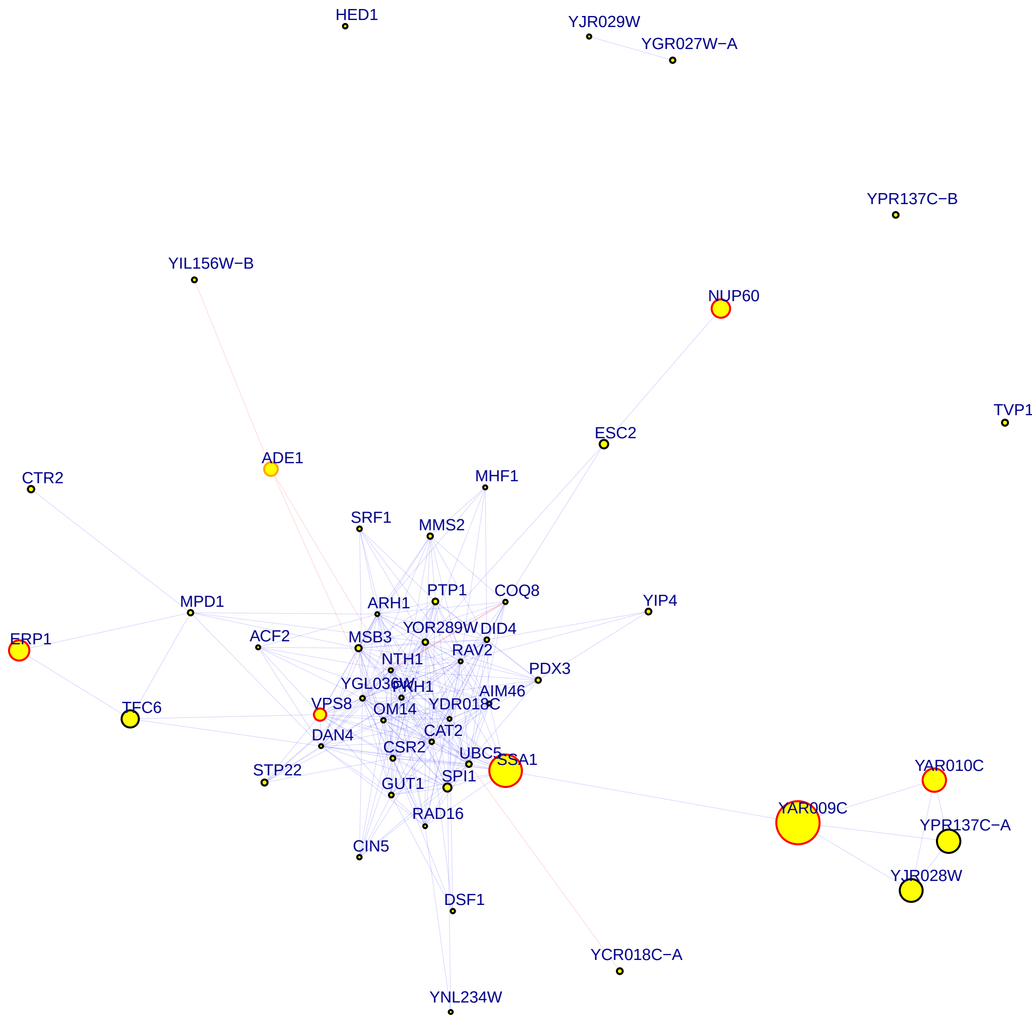

chrI:159467_G/A top 100 up and down genes

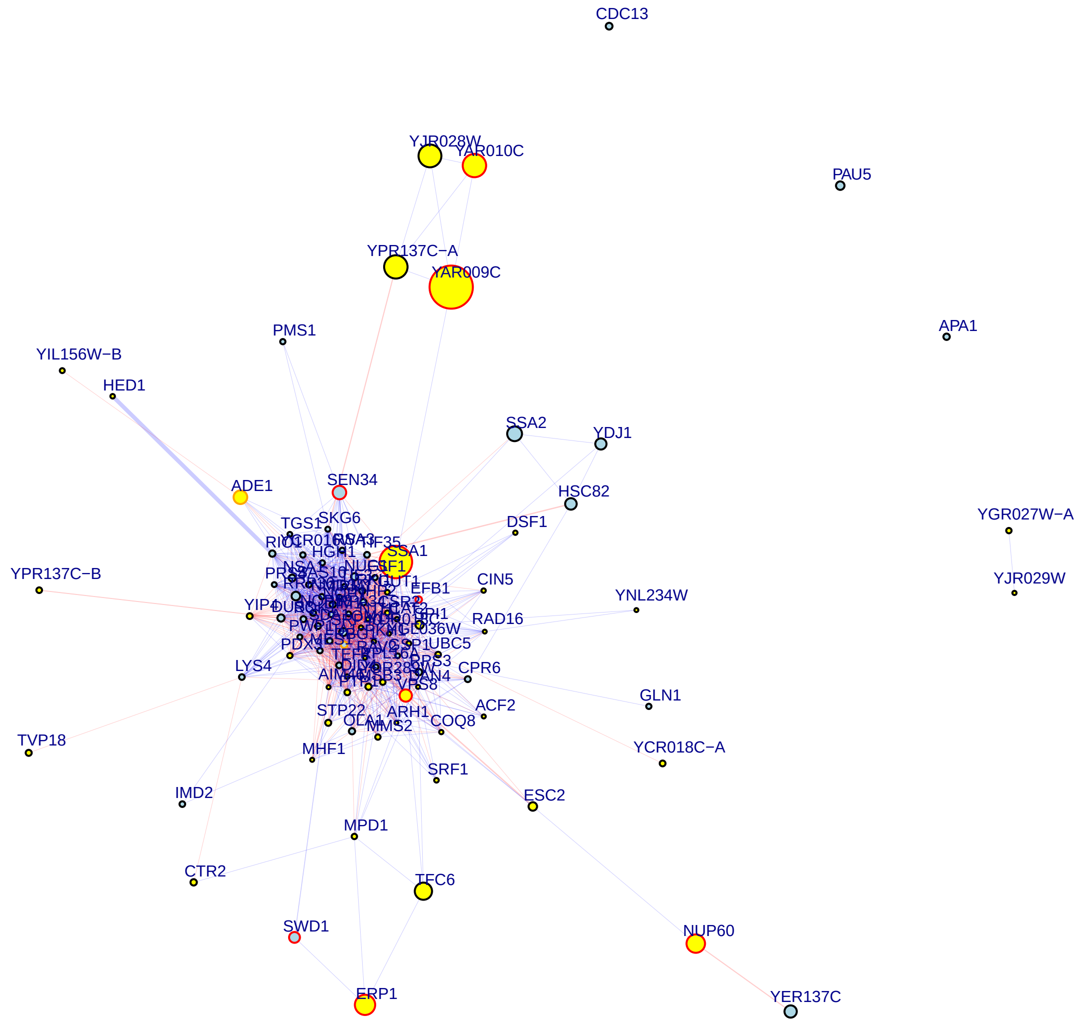

chrI:189123_C/T top 16 up genes

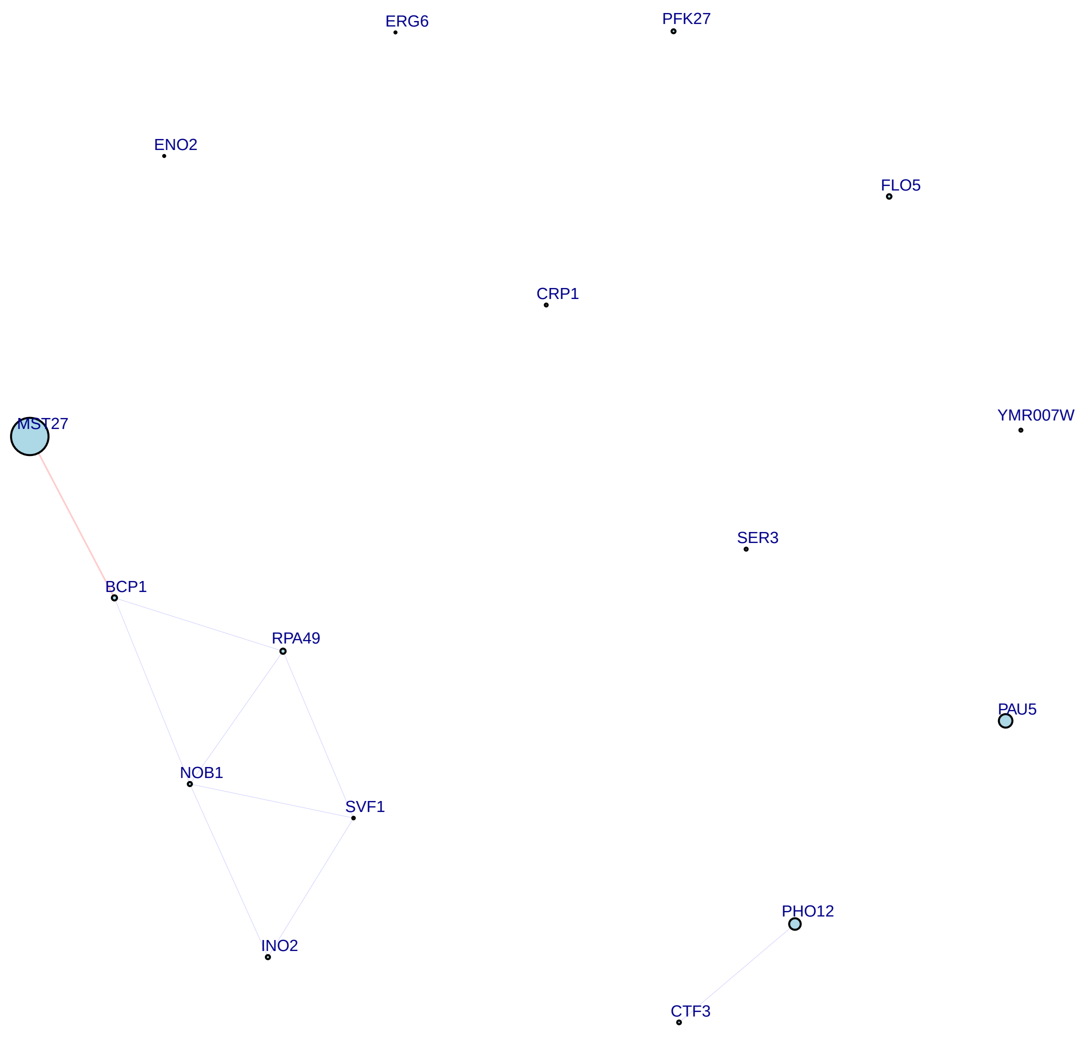

chrI:189123_C/T top 28 down genes

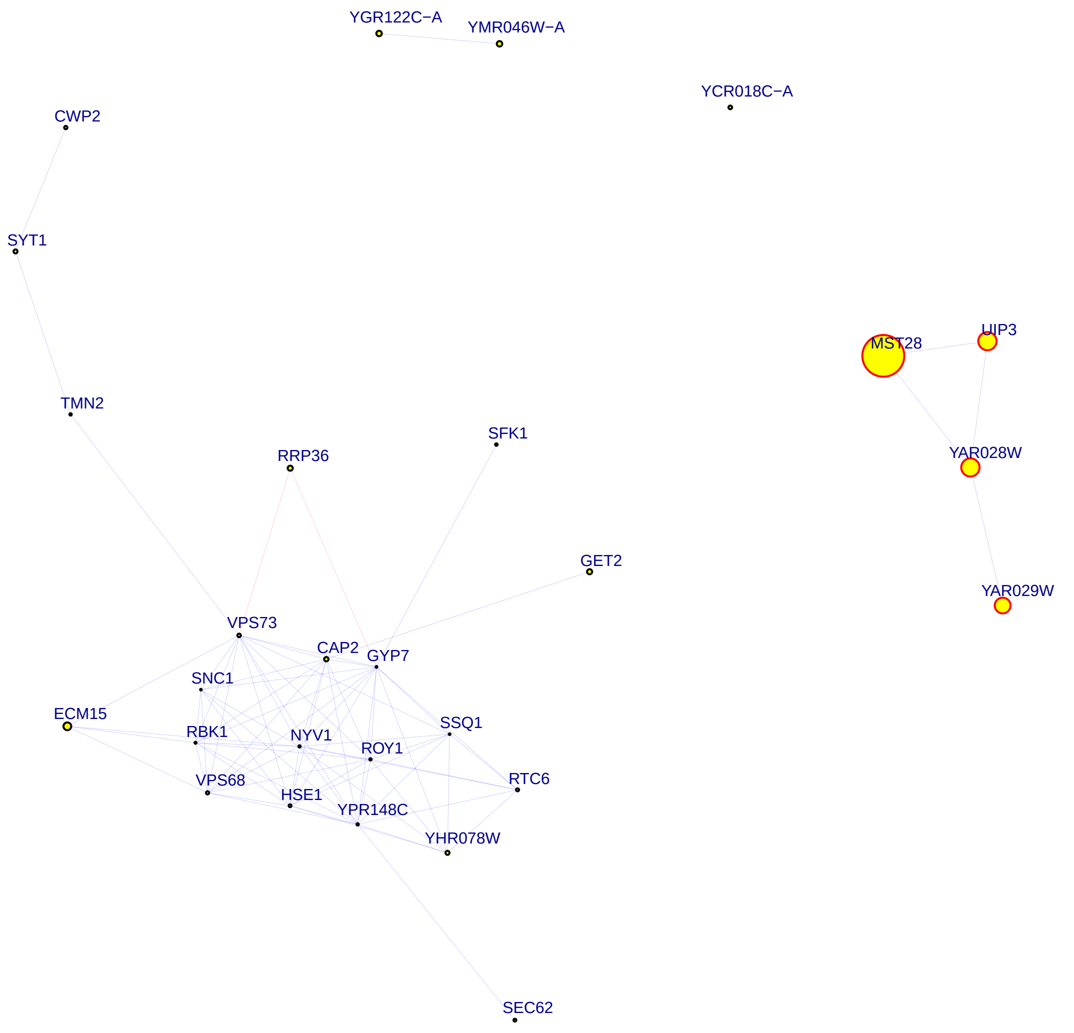

chrI:189123_C/T top 44 up and down genes

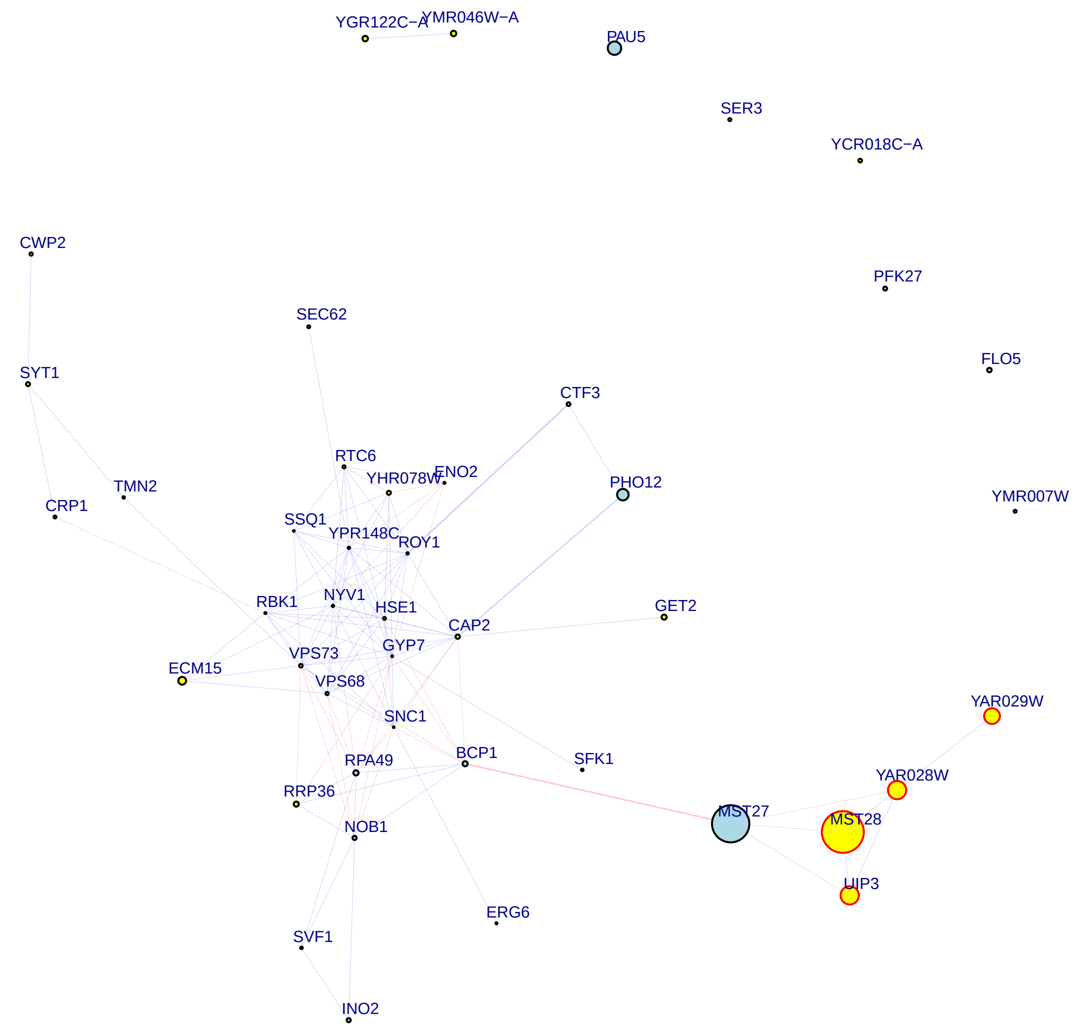

chrII:13061_G/T top 18 up genes

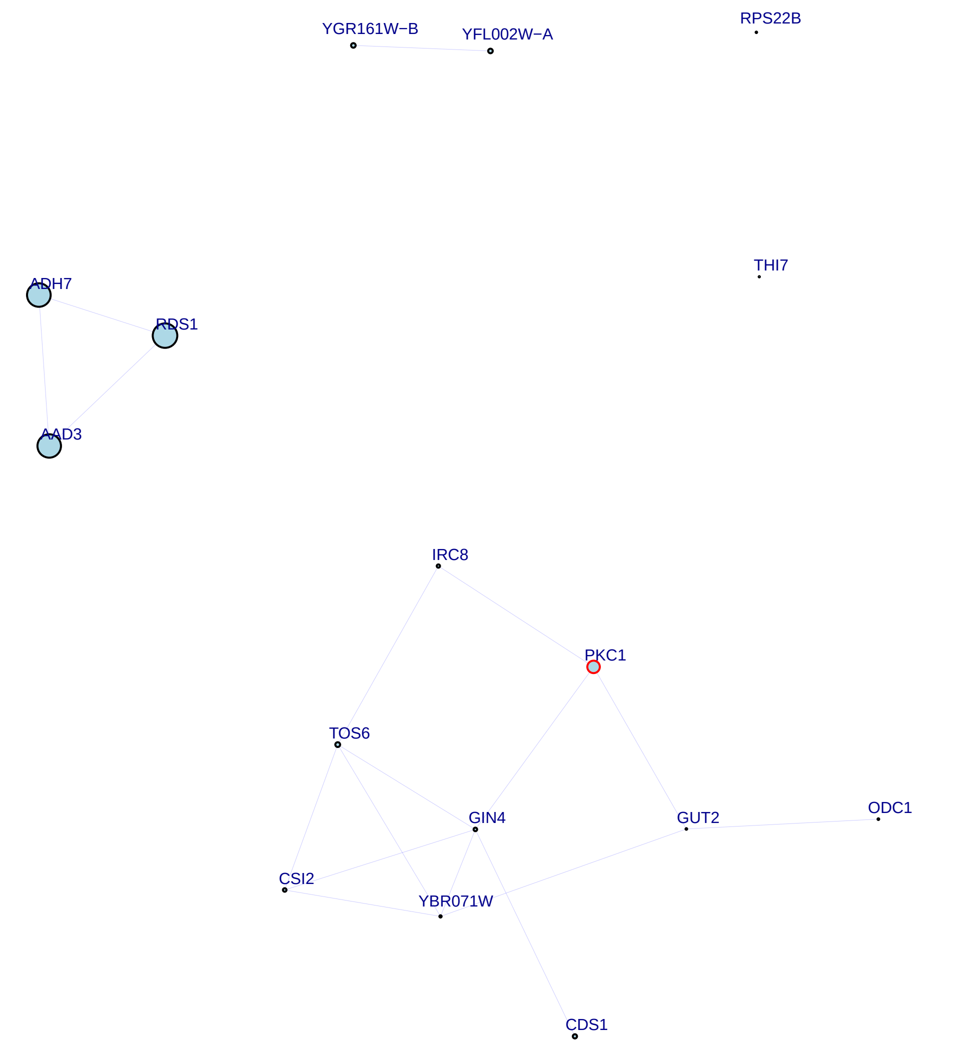

chrII:13061_G/T top 15 down genes

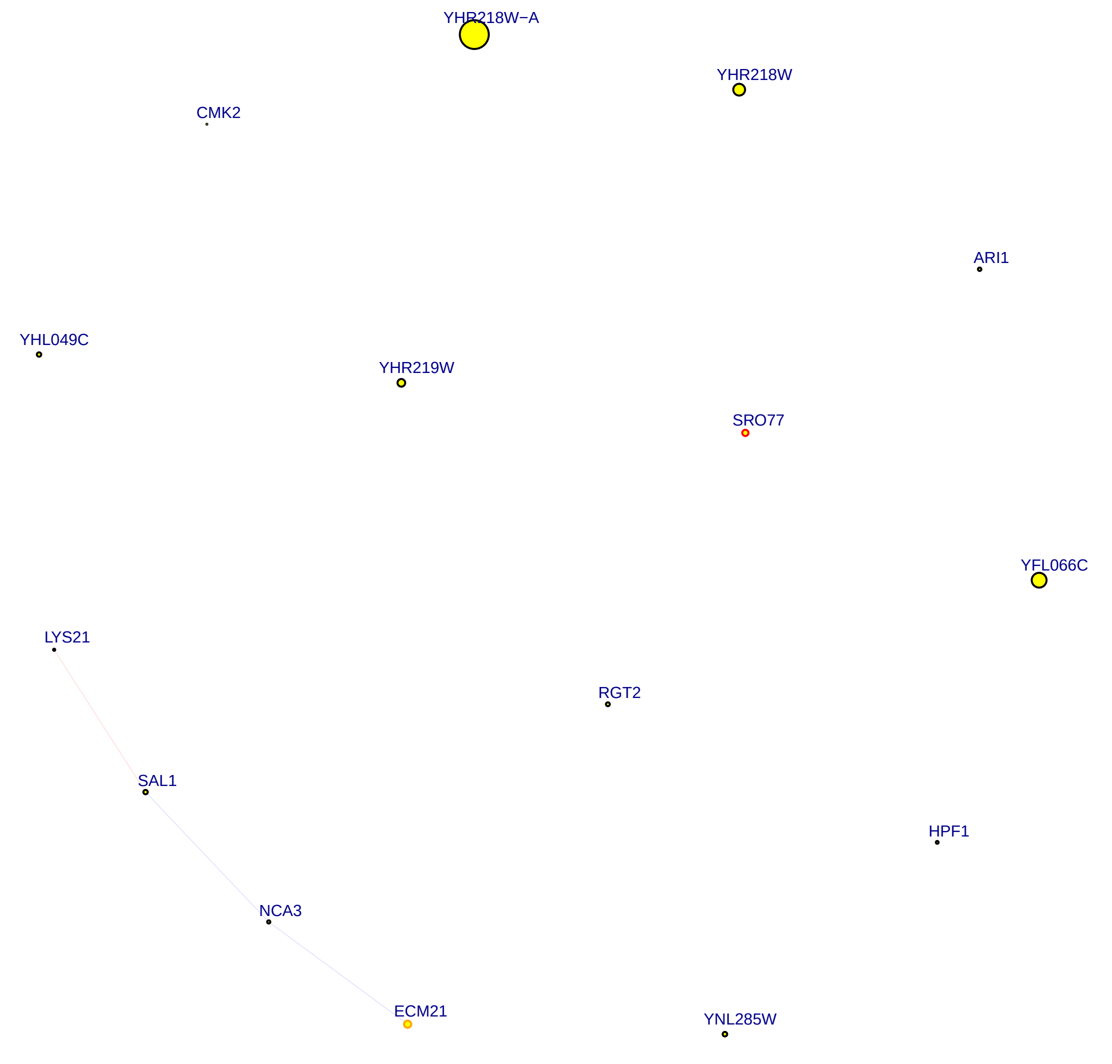

chrII:13061_G/T top 33 up and down genes

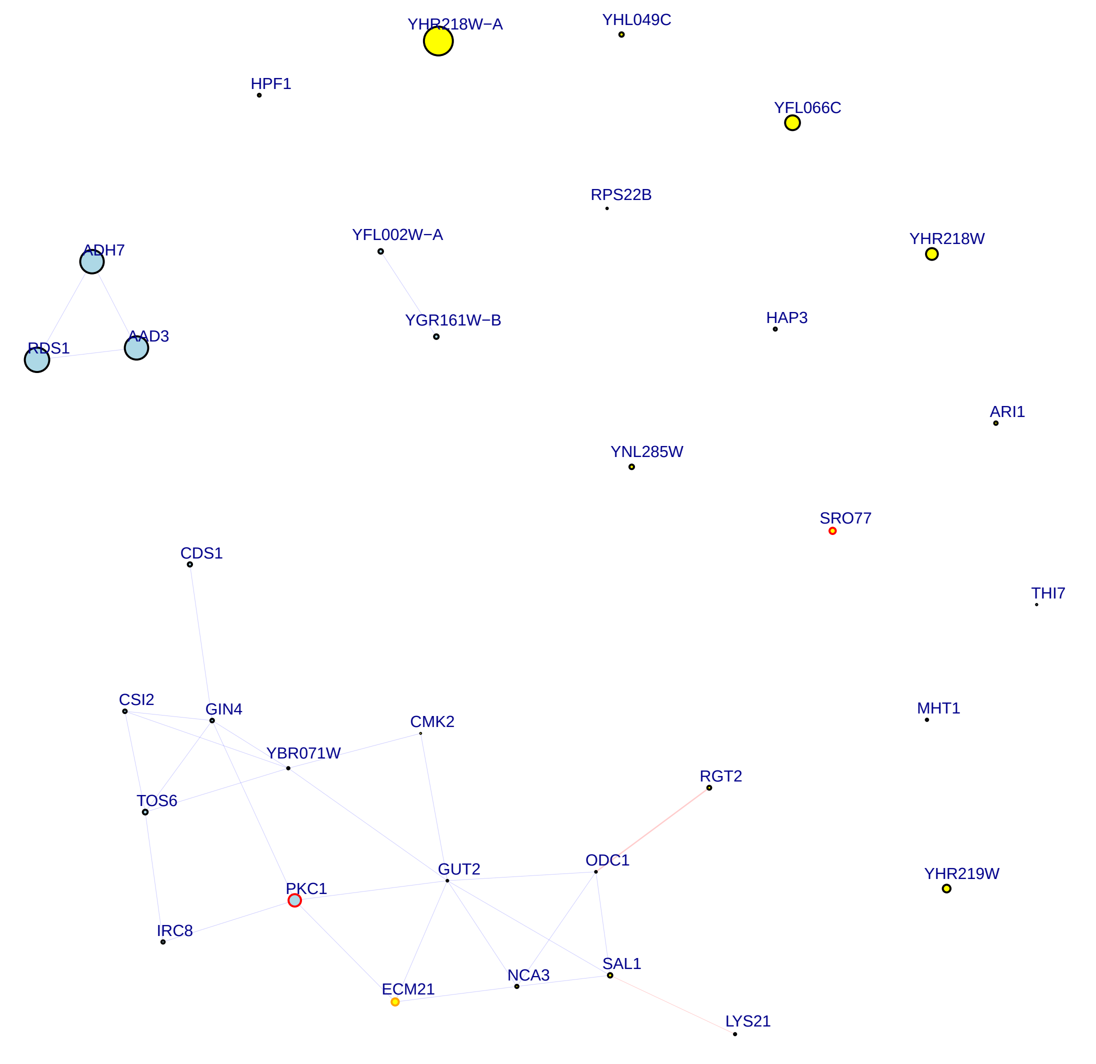

chrII:123764_G/A top 50 up genes

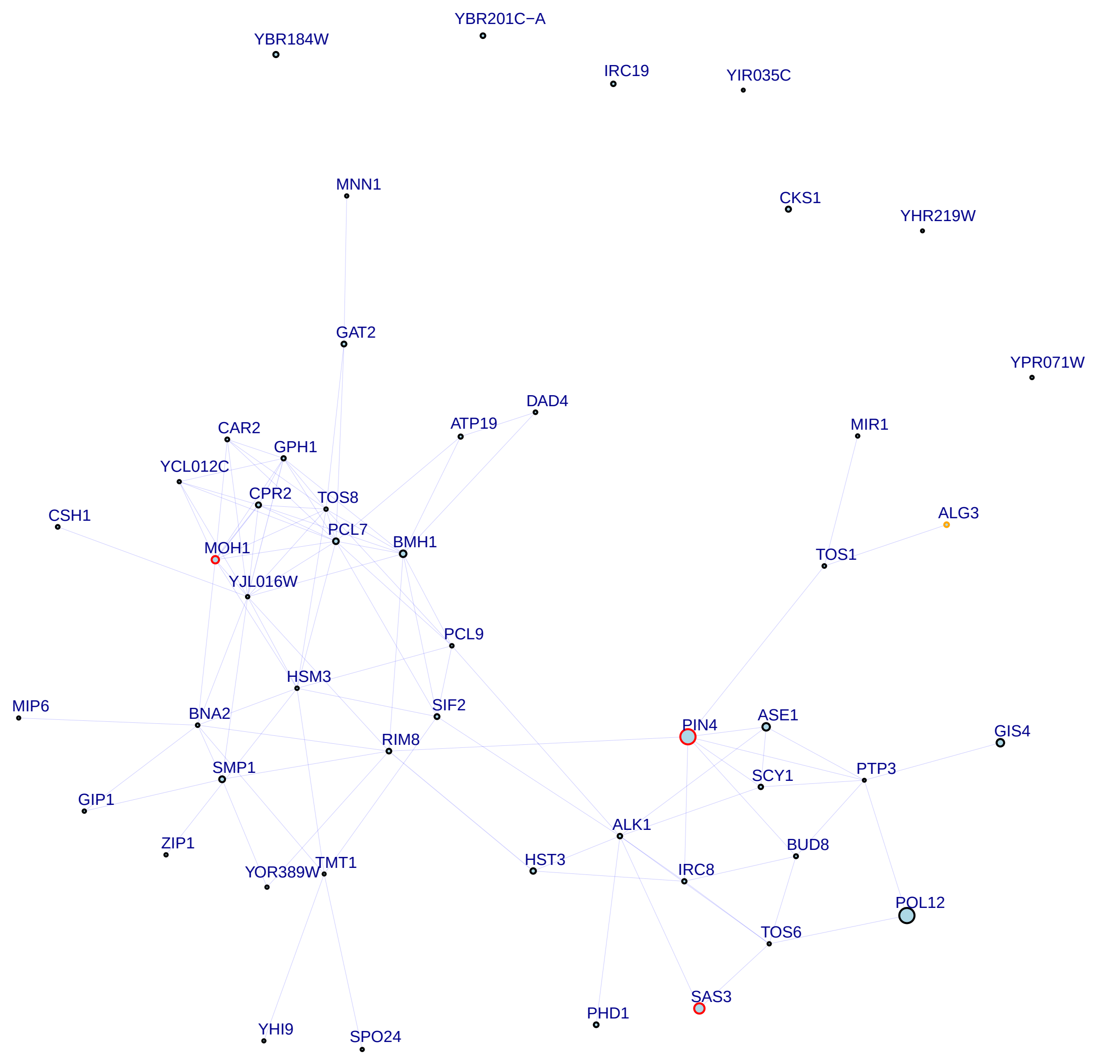

chrII:123764_G/A top 50 down genes

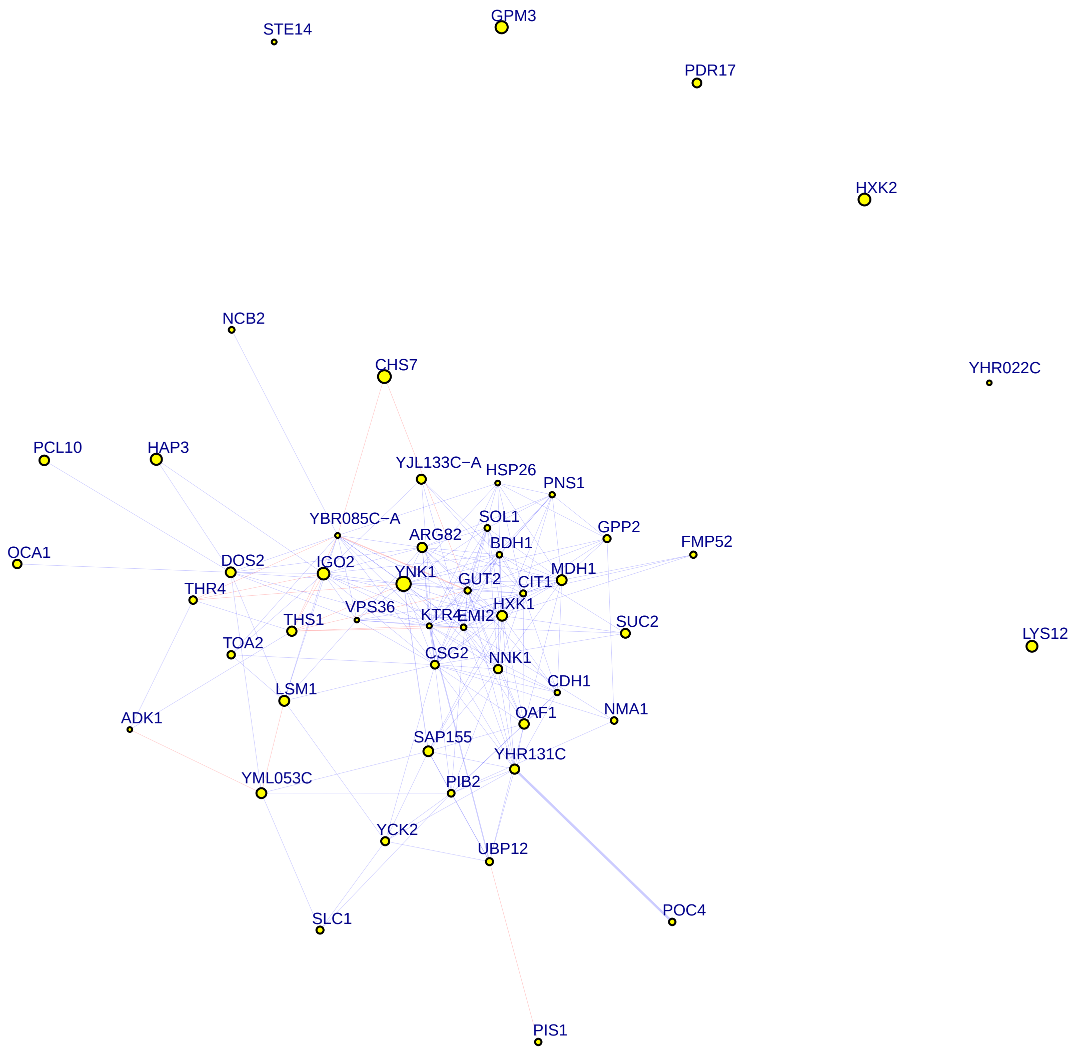

chrII:123764_G/A top 100 up and down genes

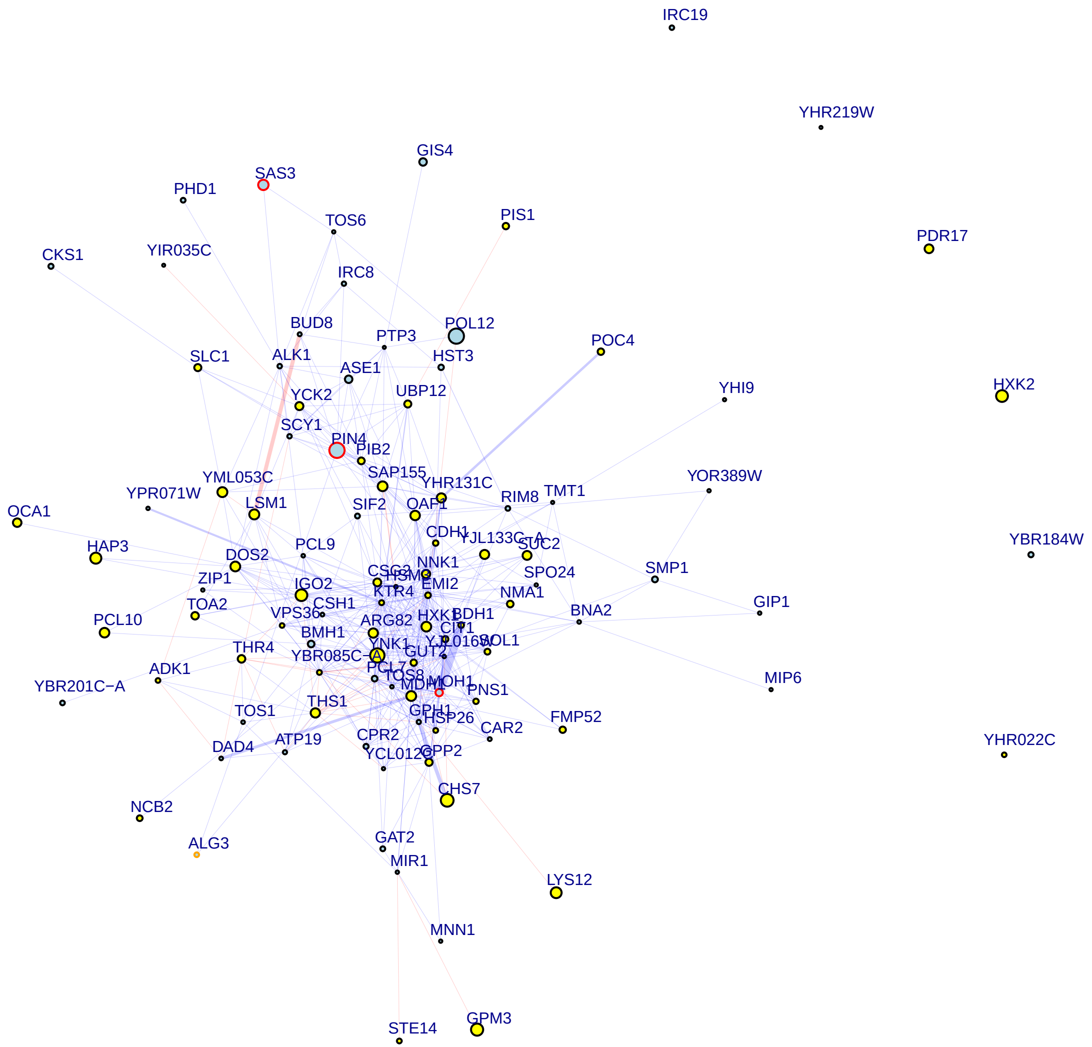

chrII:195177_T/A top 50 up genes

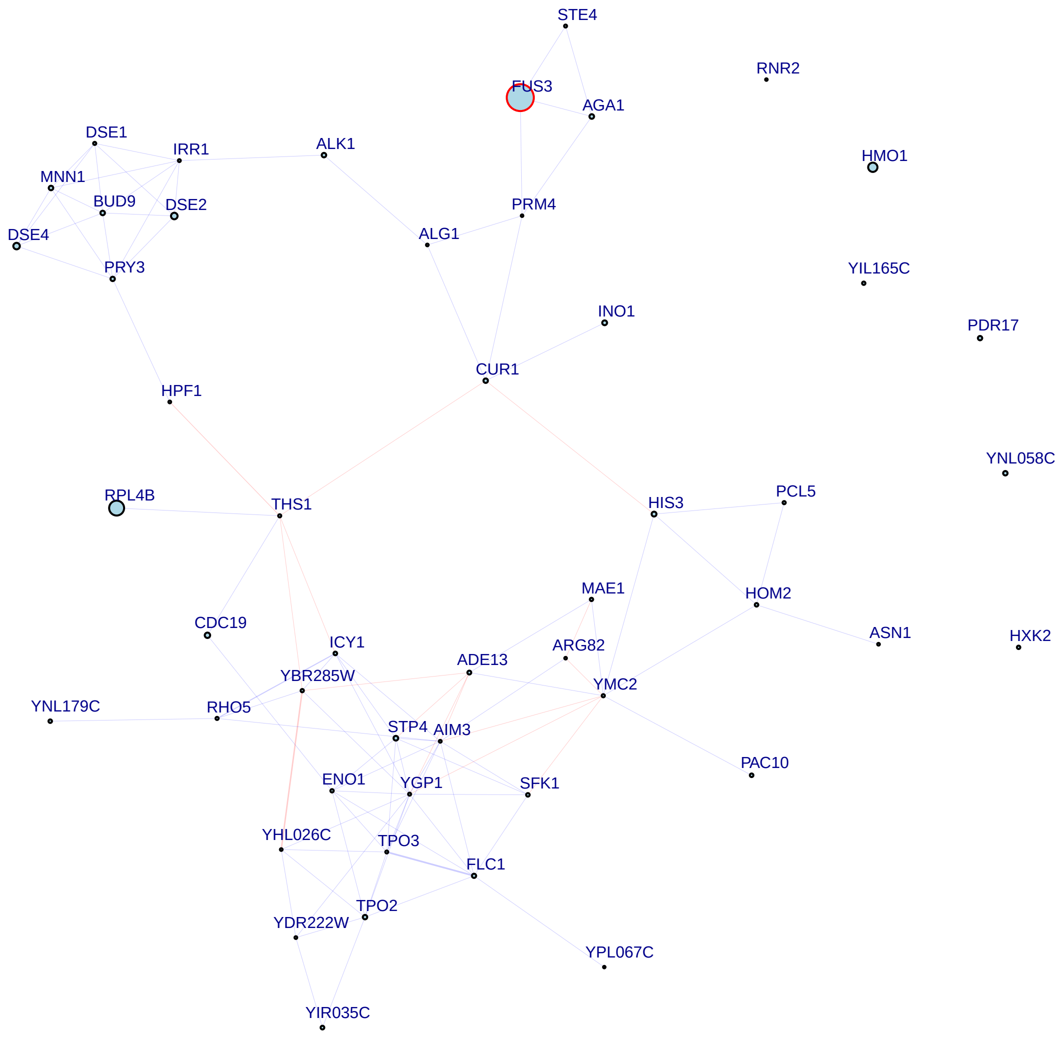

chrII:195177_T/A top 50 down genes

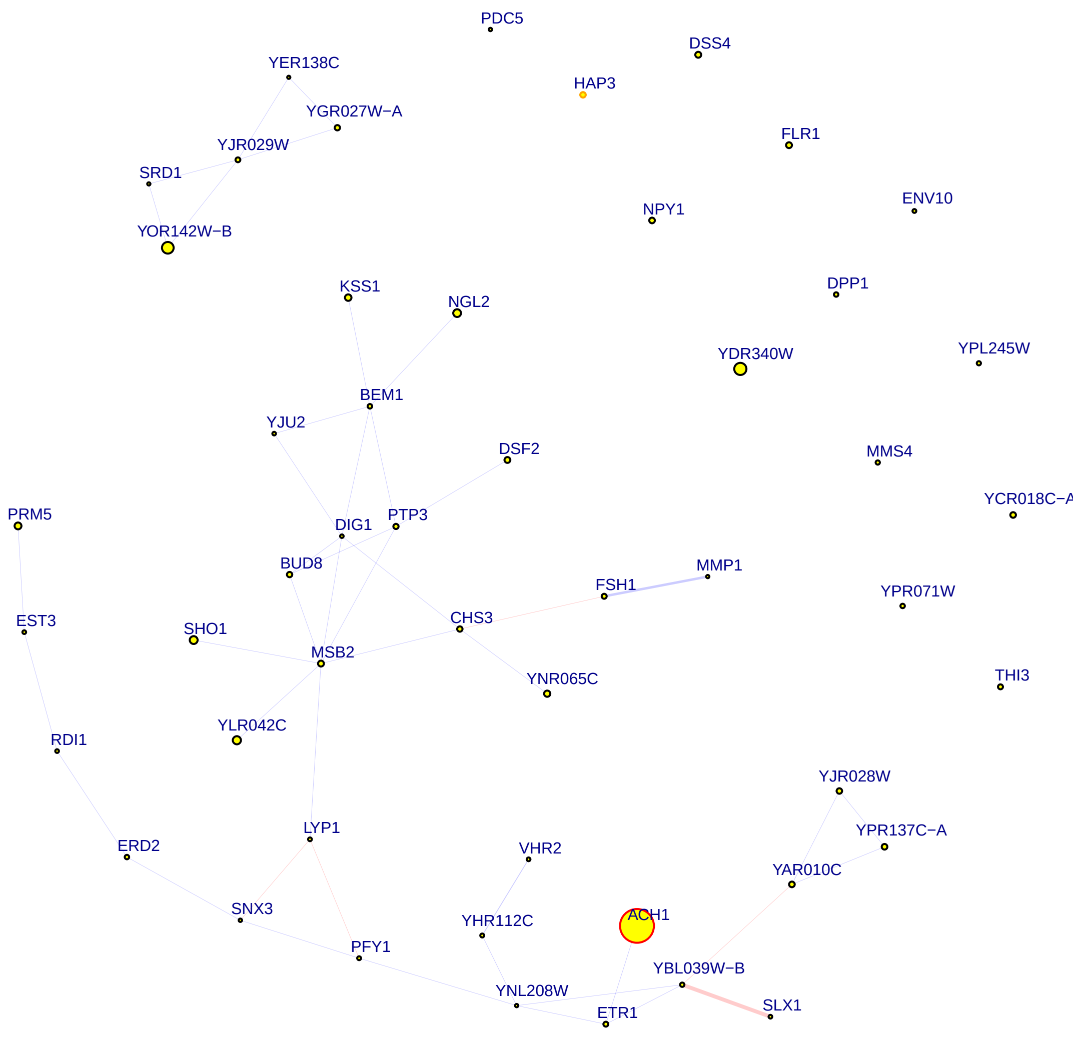

chrII:195177_T/A top 100 up and down genes

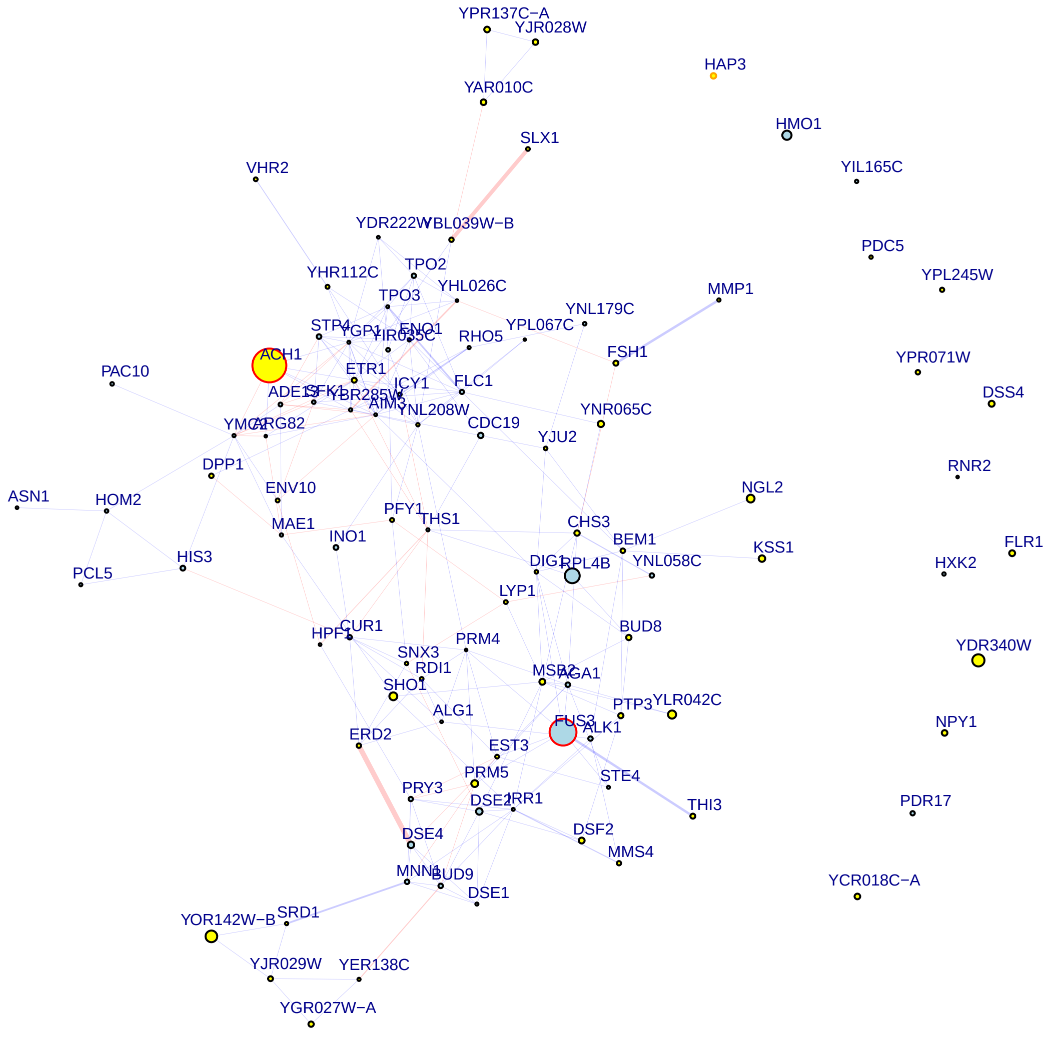

chrII:370967_C/T top 50 up genes

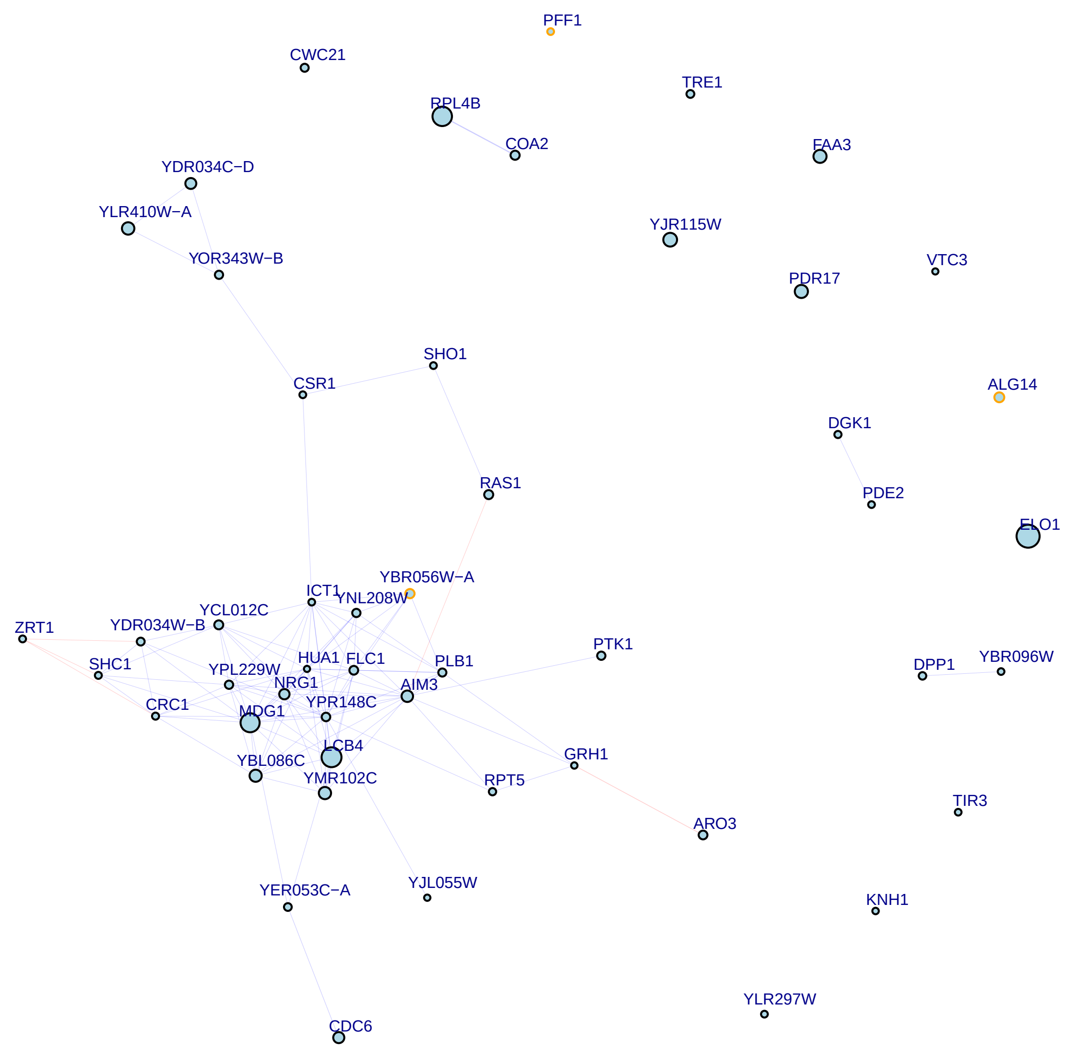

chrII:370967_C/T top 36 down genes

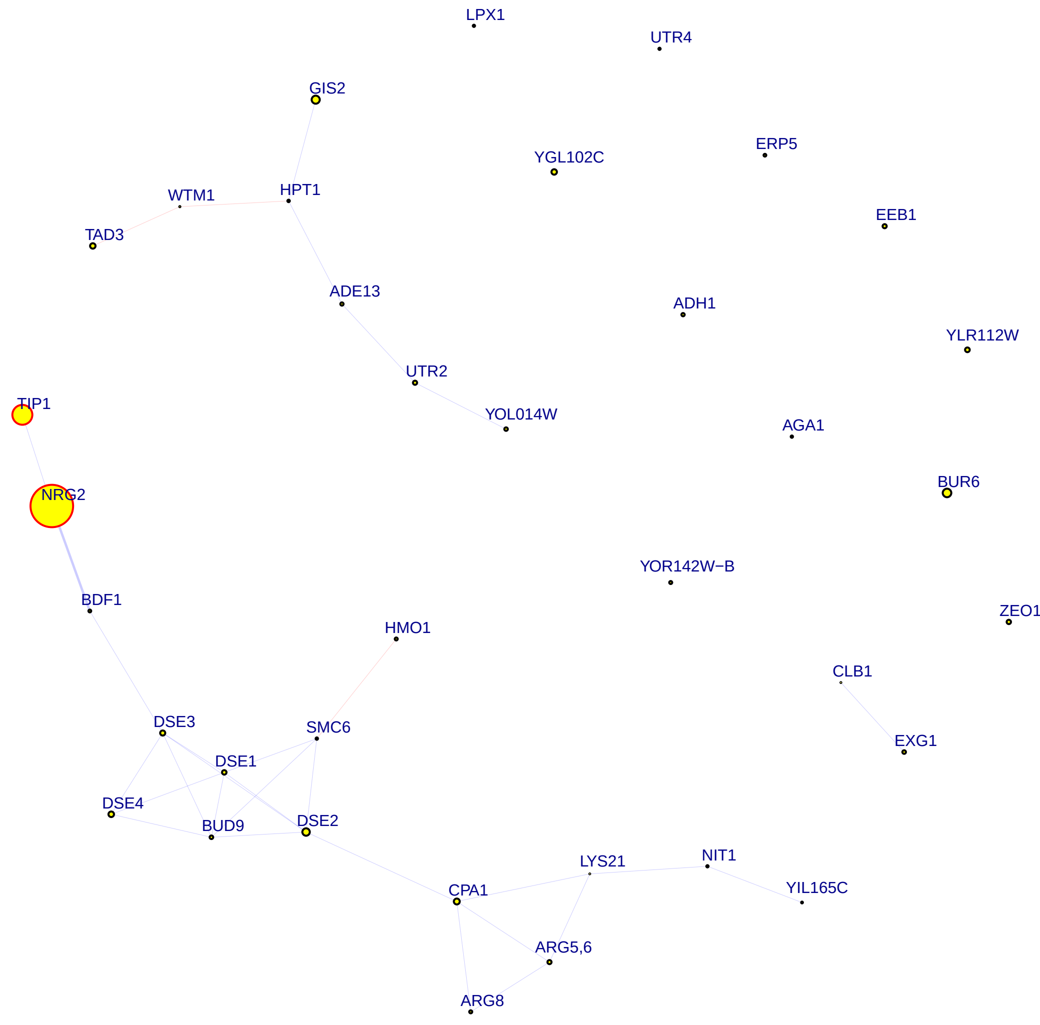

chrII:370967_C/T top 86 up and down genes

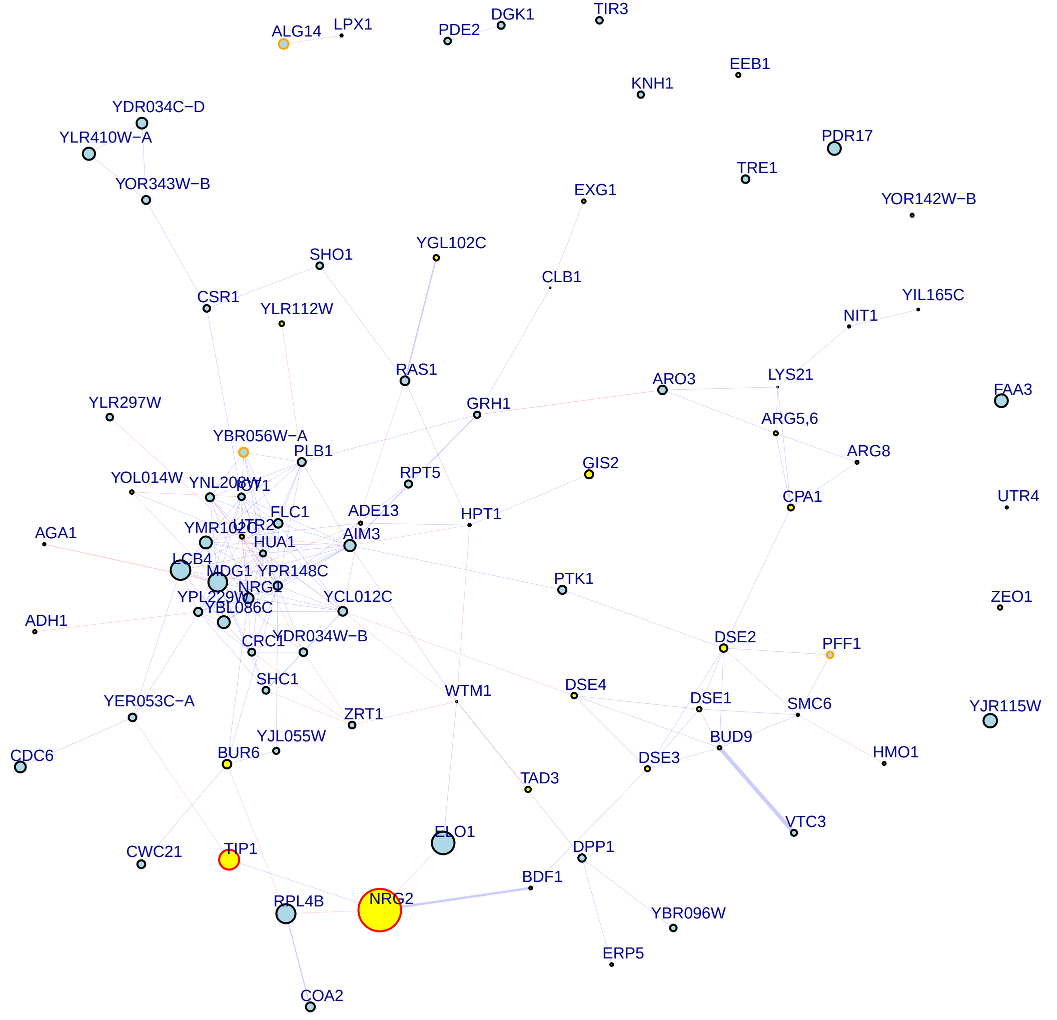

chrII:464478_G/A top 50 up genes

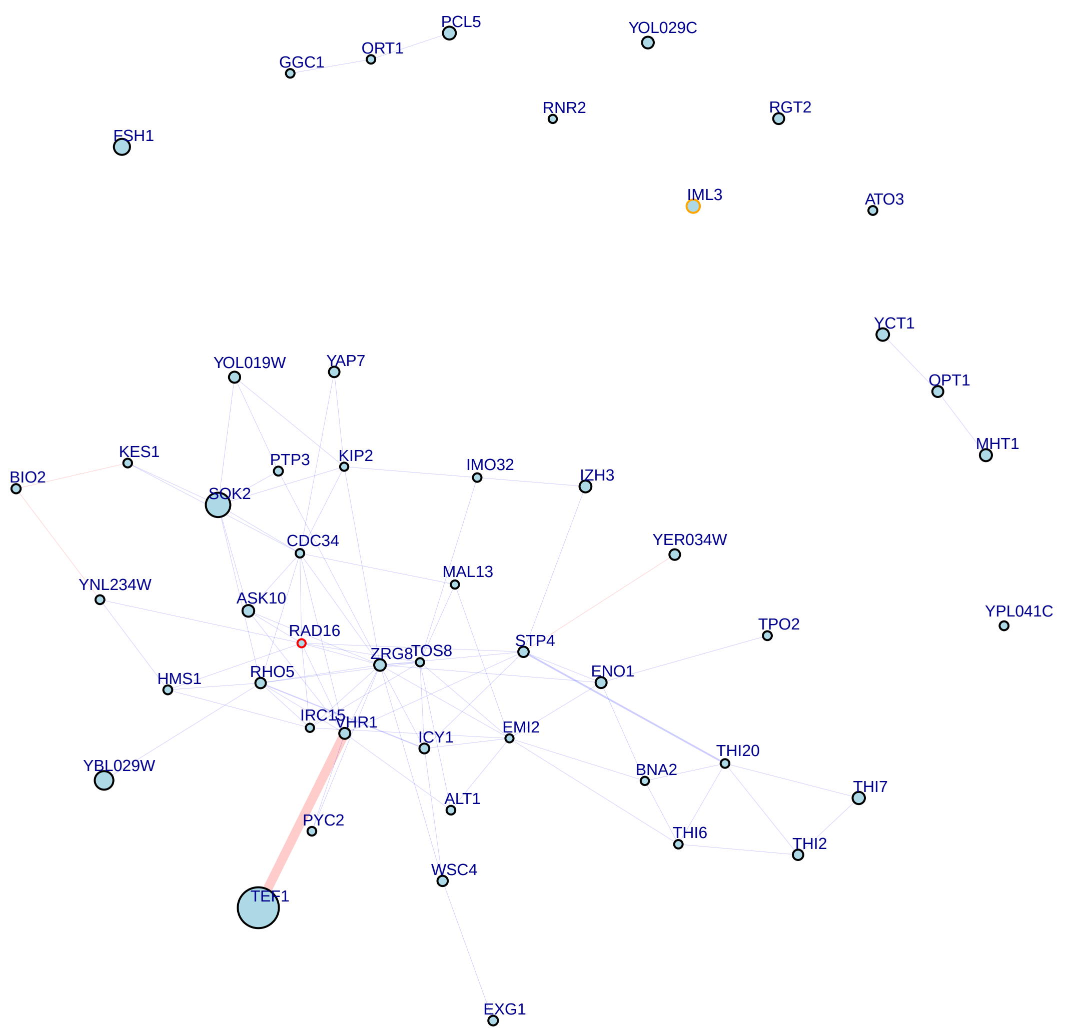

chrII:464478_G/A top 50 down genes

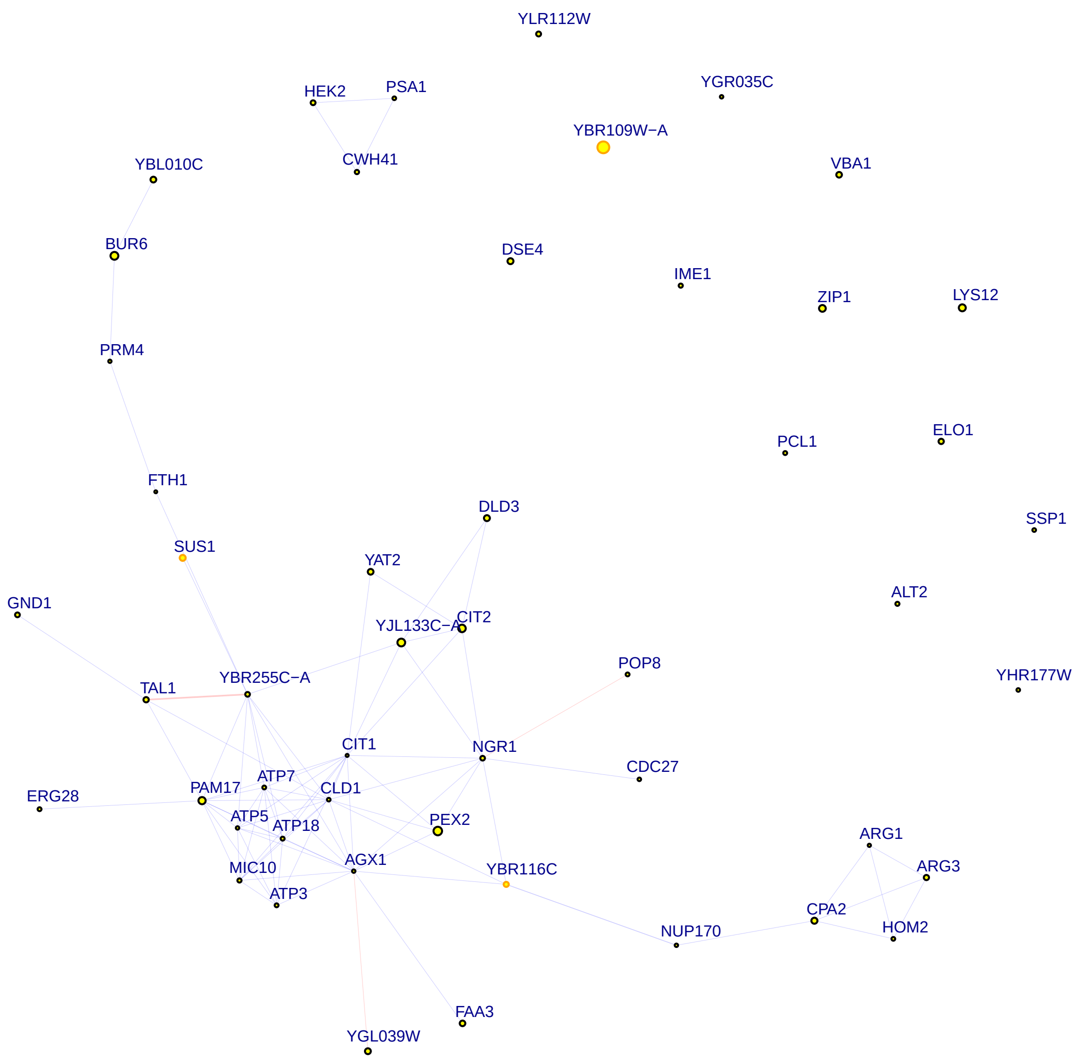

chrII:464478_G/A top 100 up and down genes

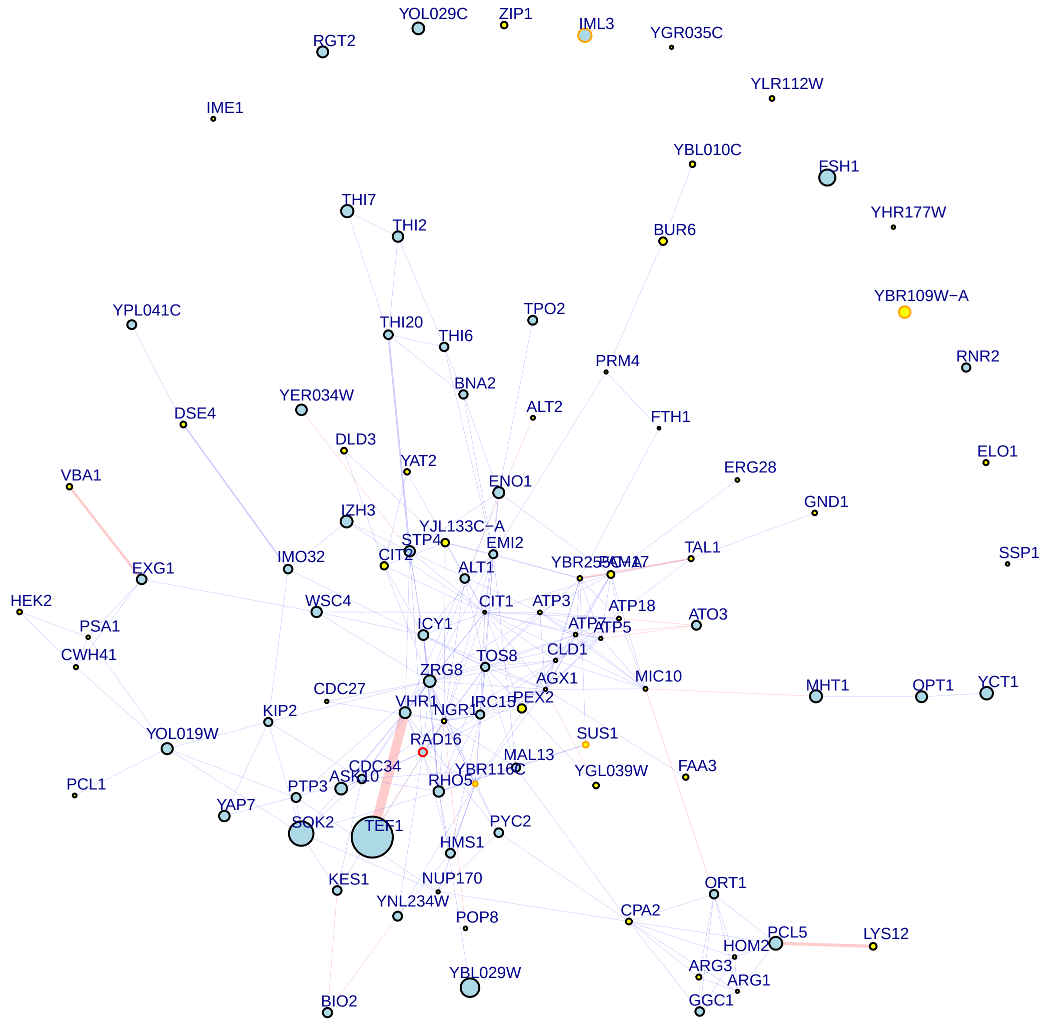

chrII:541139_G/A top 50 up genes

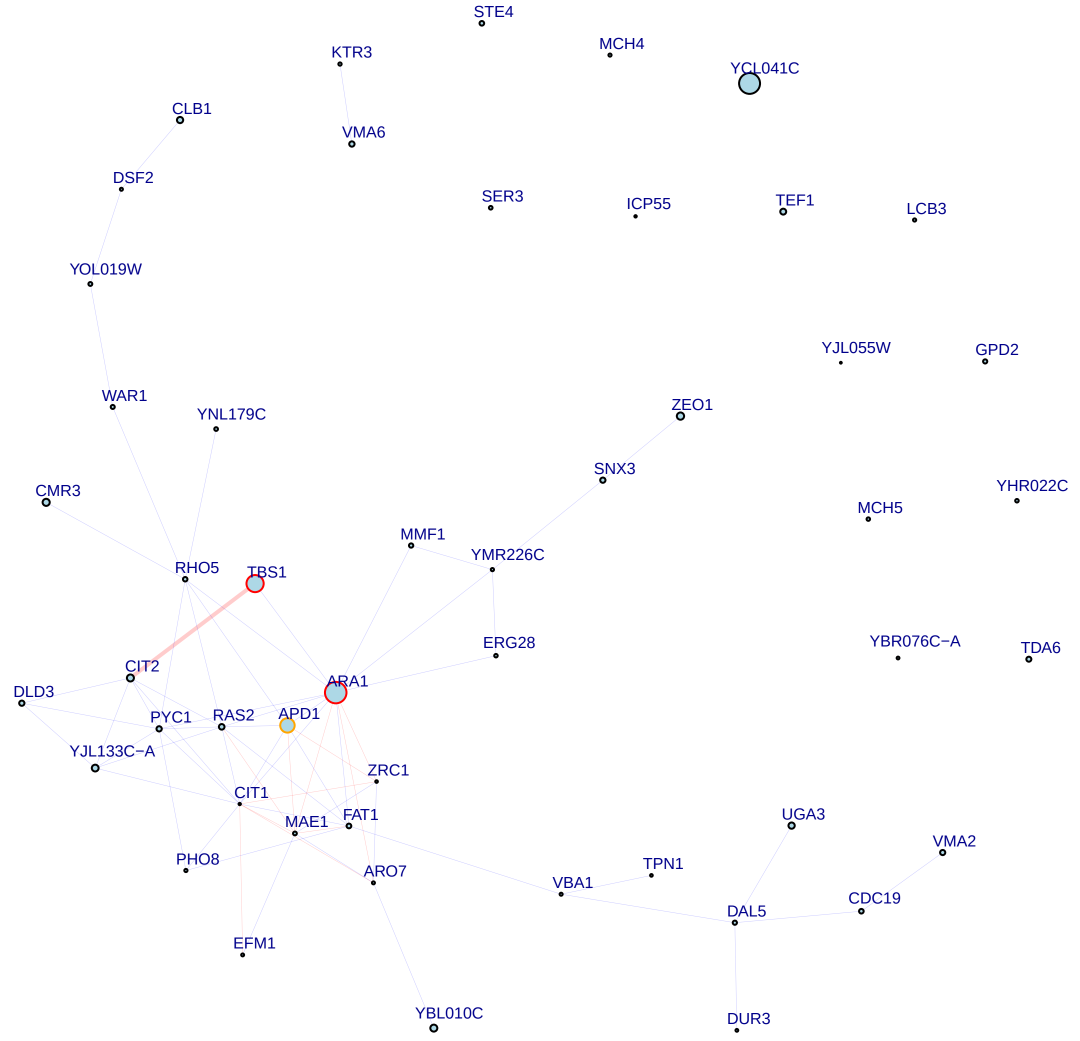

chrII:541139_G/A top 50 down genes

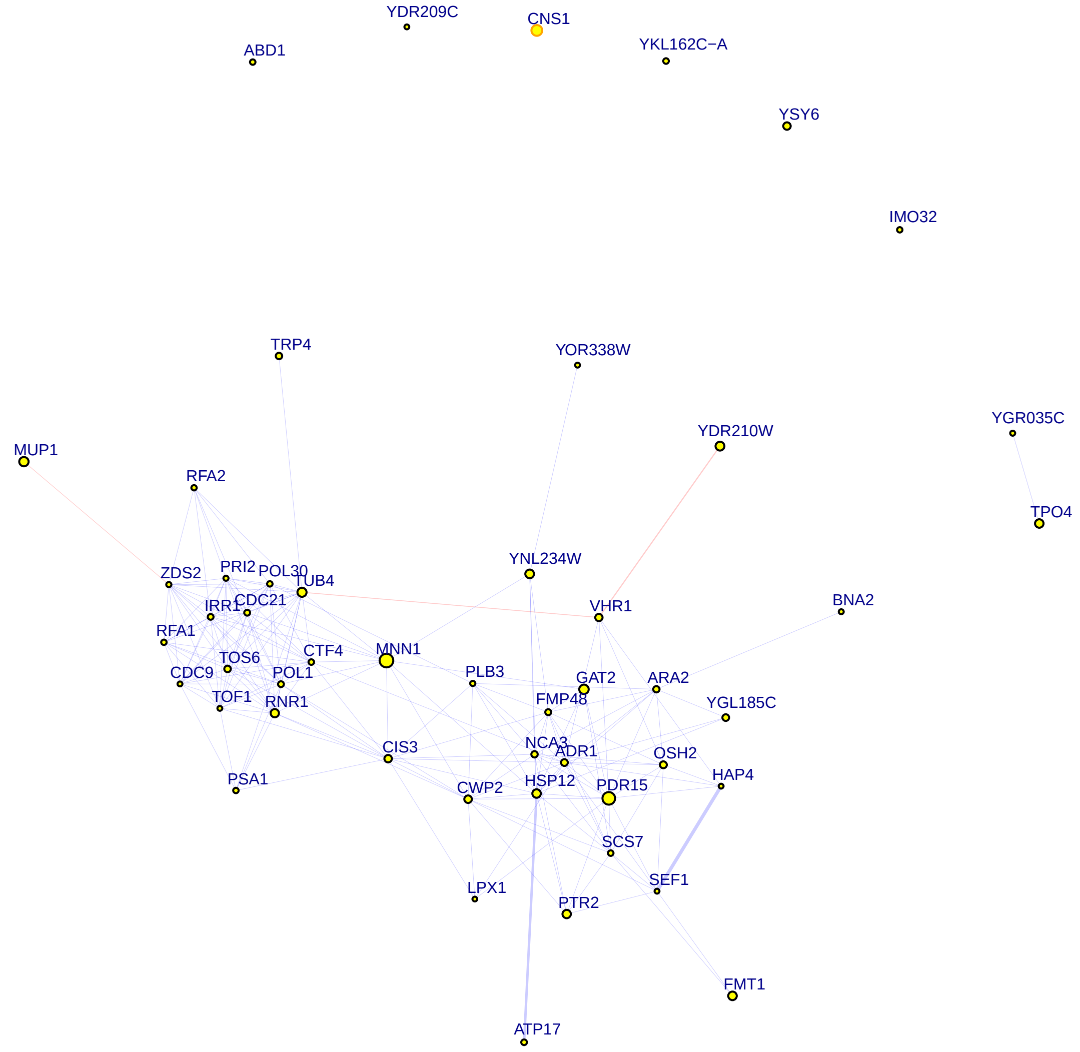

chrII:541139_G/A top 100 up and down genes

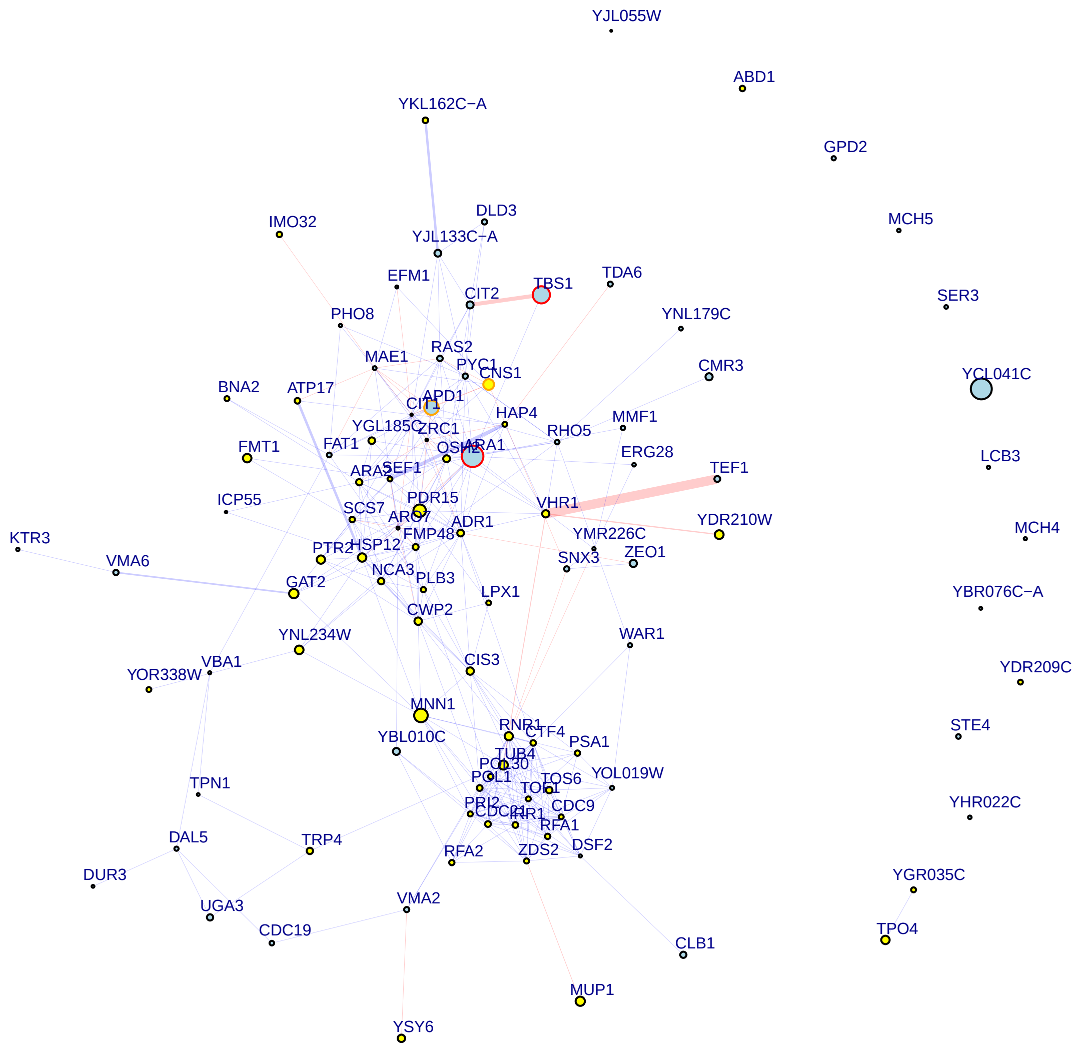

chrII:619028_C/A top 50 up genes

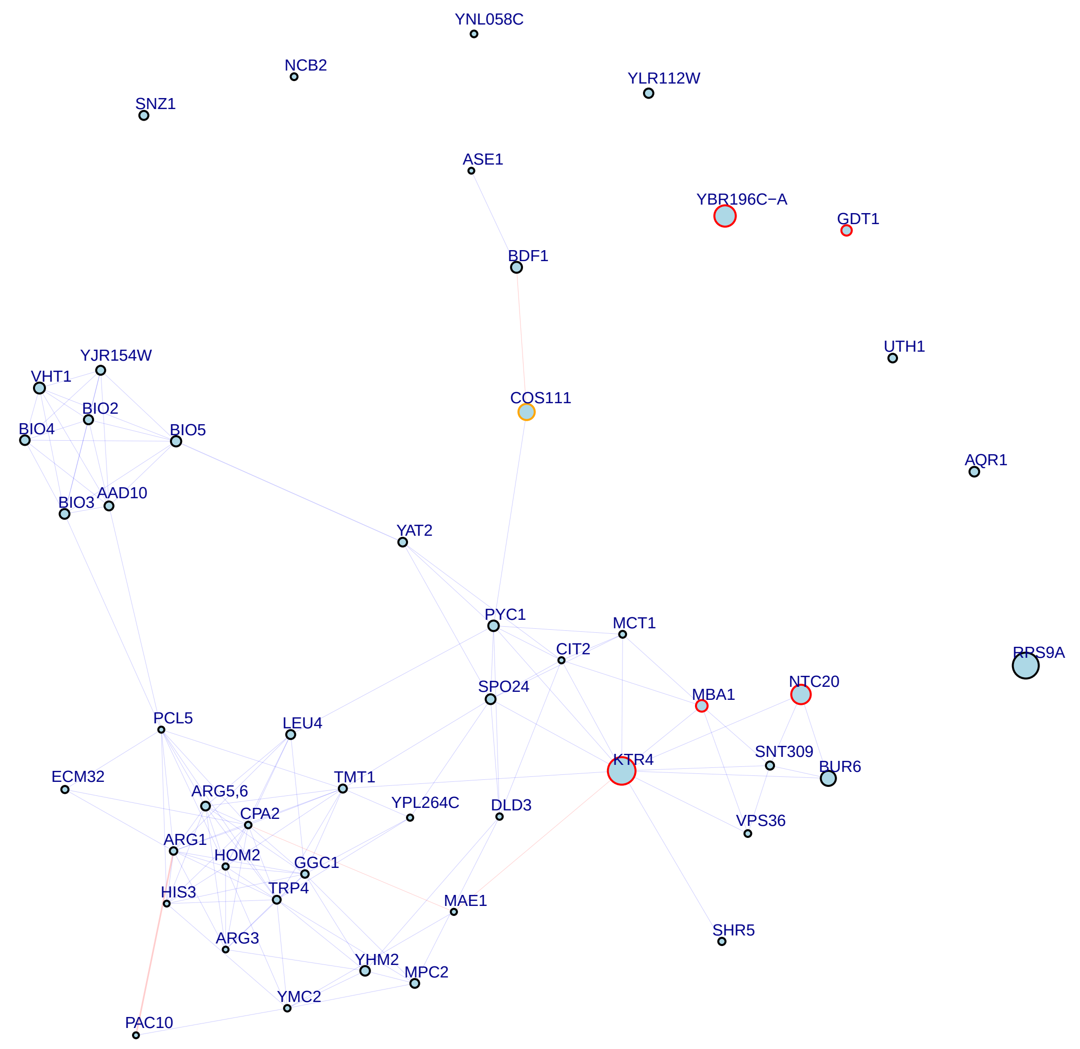

chrII:619028_C/A top 50 down genes

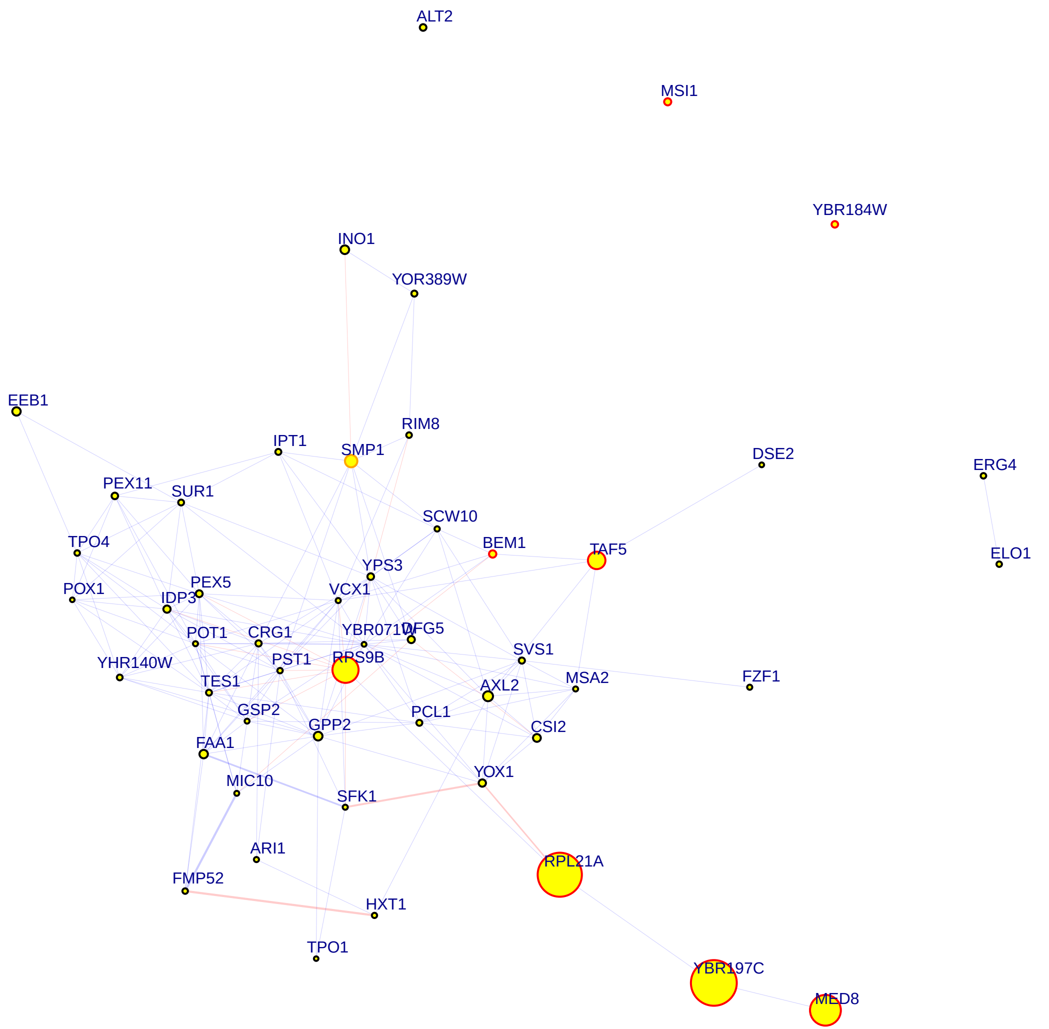

chrII:619028_C/A top 100 up and down genes

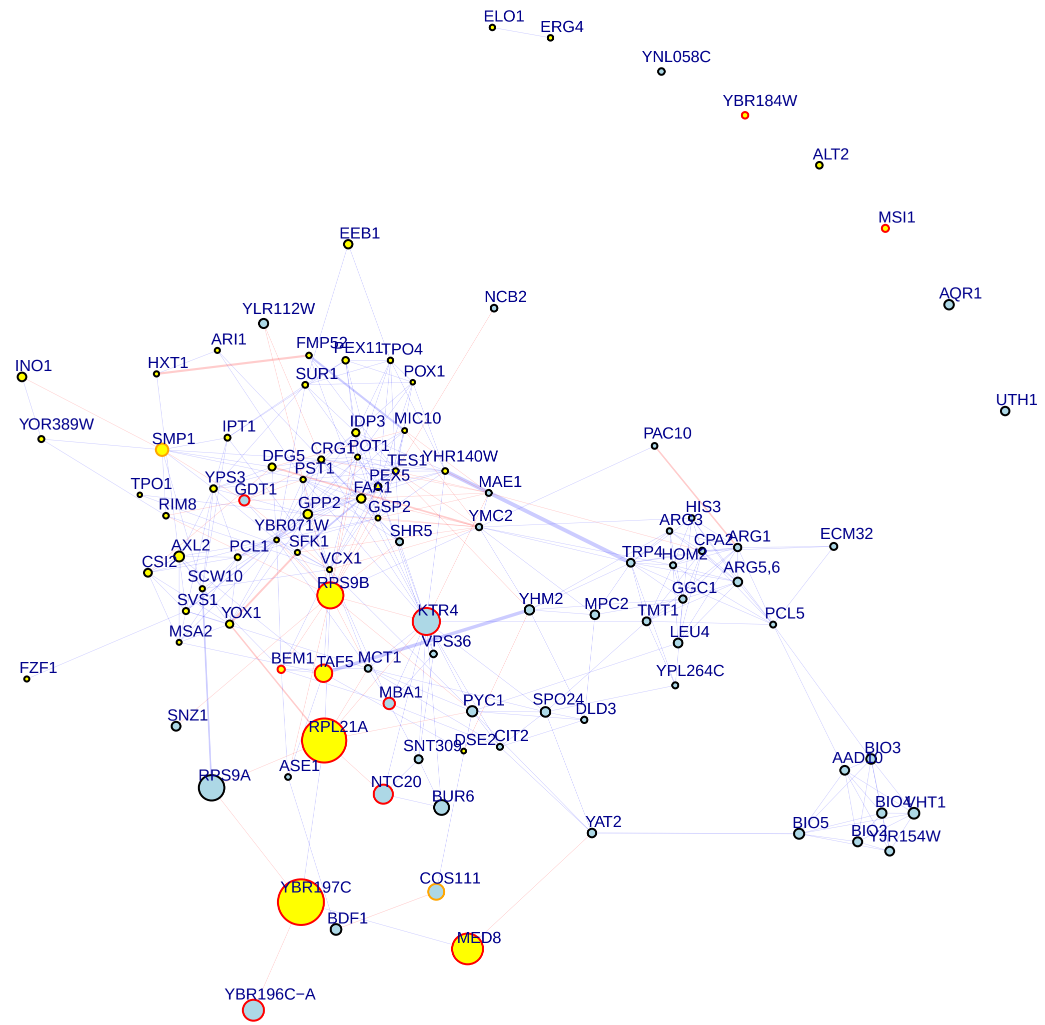

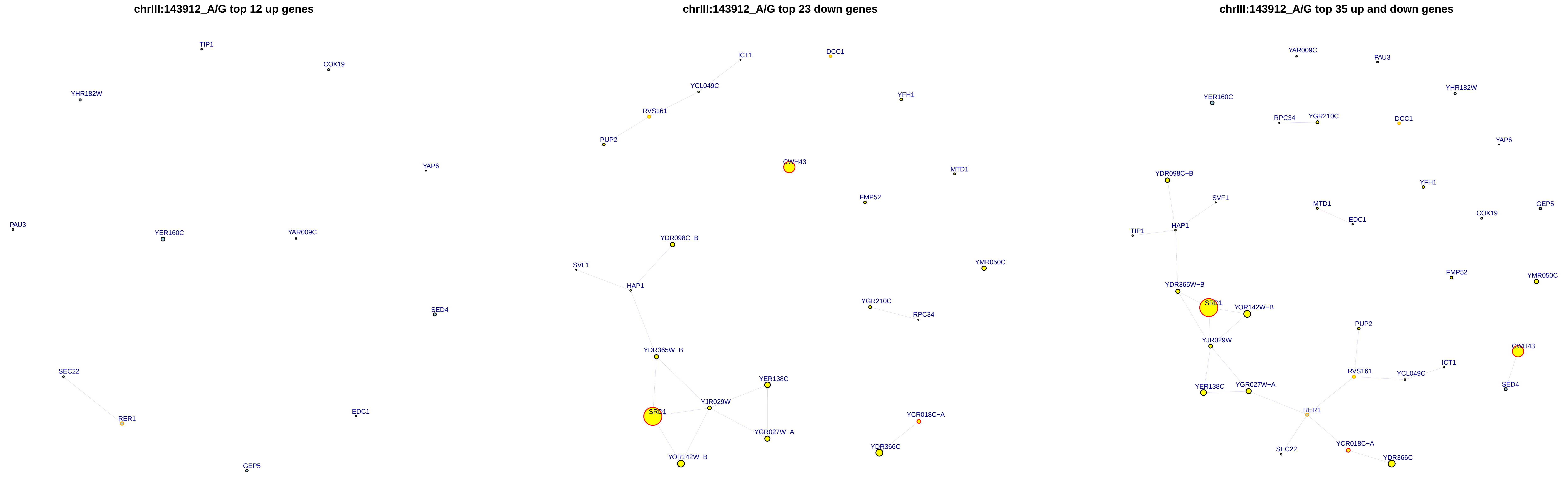
chrIII:143912_A/G top 12 up genes
TIP1
COX19
YHR182W
YAP6
PAU3
YER160C
YAR009C
SED4
SEC22
RER1
EDC1
GEP5
chrIII:143912_A/G top 23 down genes
ICT1
DCC1
YCL049C
YFH1
RVS161
PUP2
CWH43
MTD1
FMP52
YDR098C−B
SVF1
YMR050C
HAP1
YGR210C
RPC34
YDR365W−B
YER138C
YJR029W
SRO1
YCR018C−A
YGR027W−A
YDR366C
YOR142W−B
chrIII:143912_A/G top 35 up and down genes
YAR009C
PAU3
YER160C
YHR182W
RPC34
YGR210C
DCC1
YAP6
YDR098C−B
YFH1
SVF1
MTD1
GEP5
EDC1
COX19
TIP1
HAP1
FMP52
YMR050C
YDR365W−B
SRO1
YOR142W−B
PUP2
YJR029W
CWH43
ICT1
RVS161
YCL049C
SED4
YER138C
YGR027W−A
RER1
SEC22
YCR018C−A
YDR366C

chrIII:166390_G/C top 9 up genes

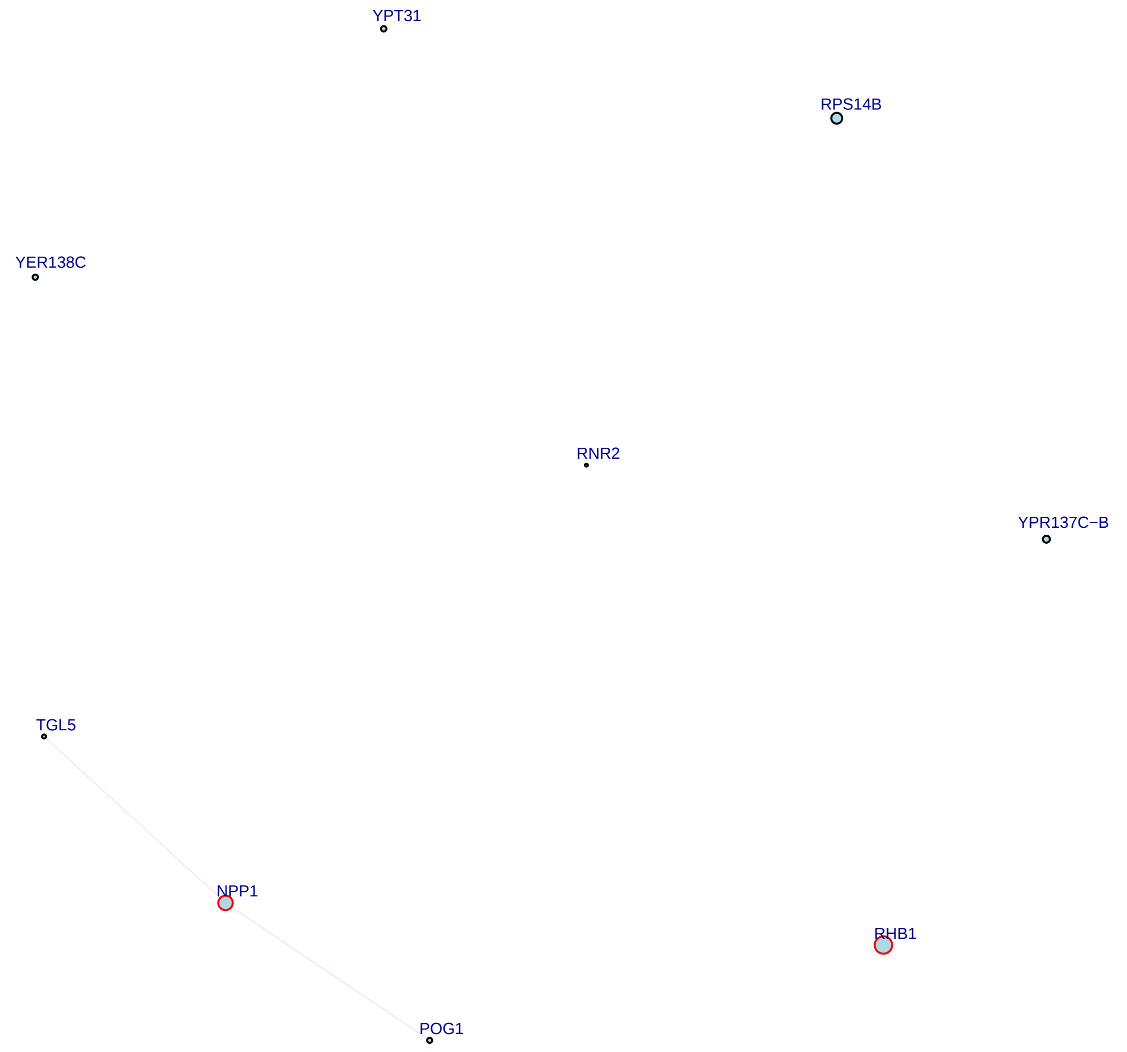

chrIII:166390_G/C top 17 down genes

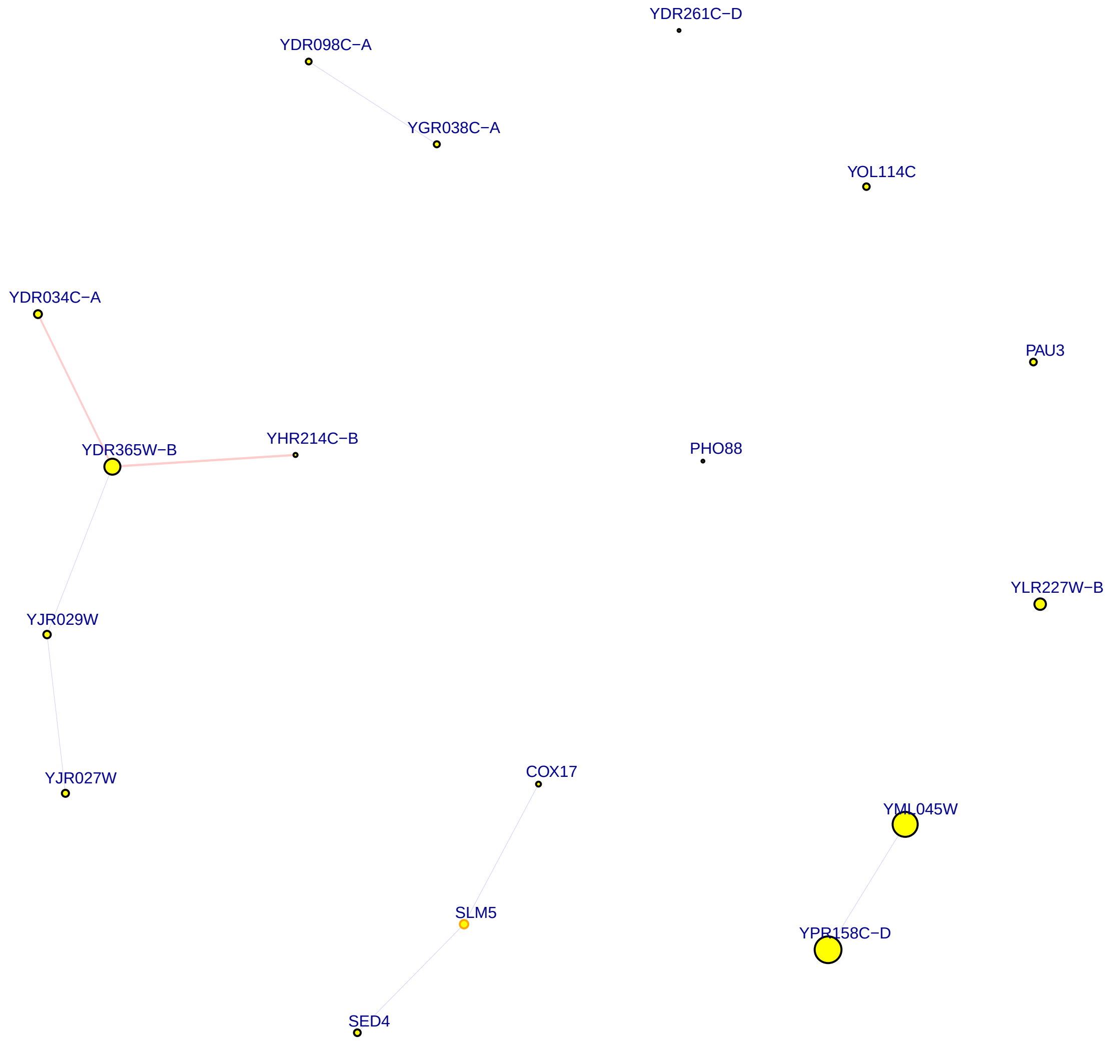

chrIII:166390_G/C top 26 up and down genes

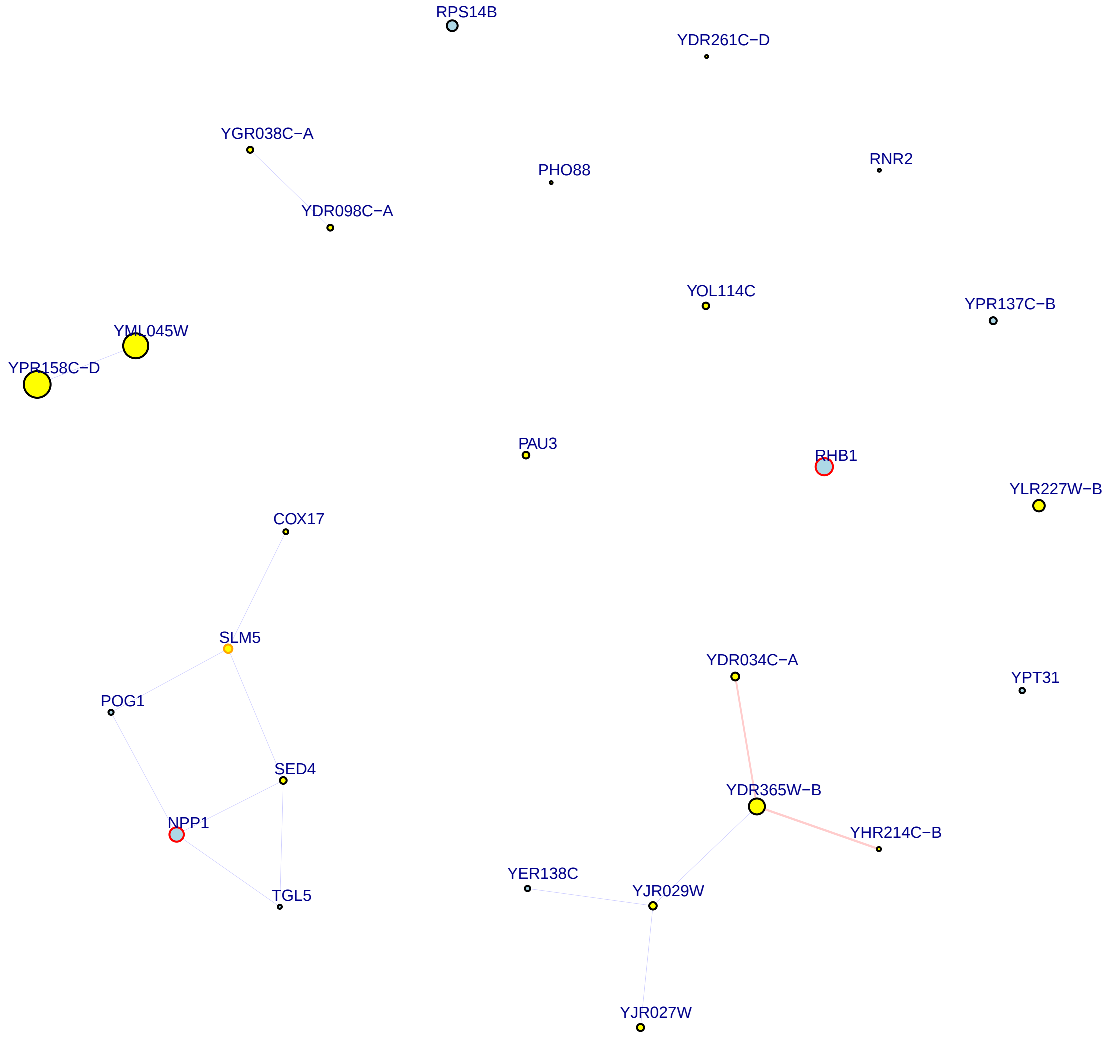

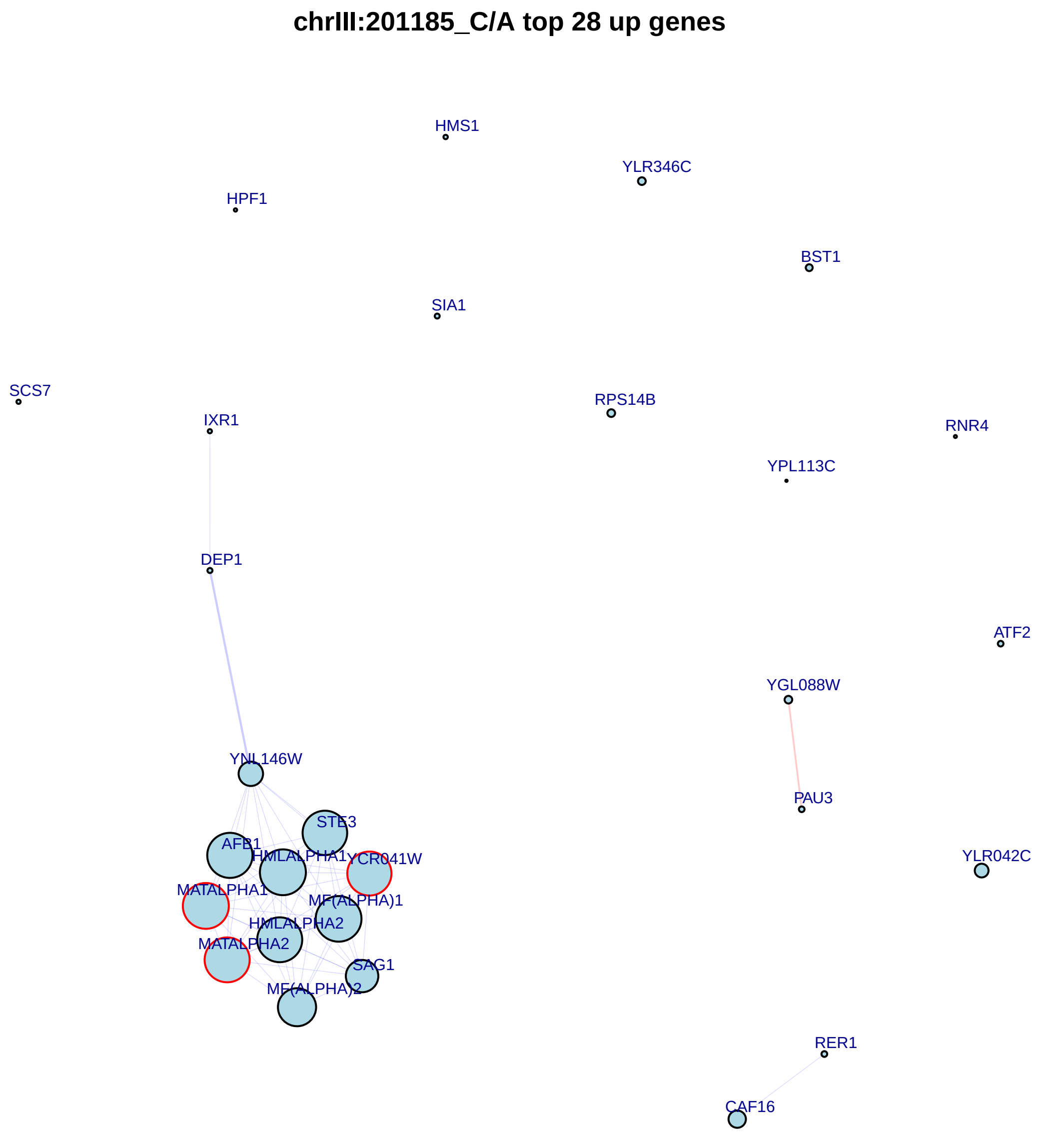
chrIII:201185_C/A top 28 up genes
HMS1
YLR346C
HPF1
BST1
SIA1
SCS7
RPS14B
IXR1
RNR4
YPL113C
DEP1
ATF2
YGL088W
YNL146W
PAU3
STE3
AFB1
HMLALPHA1
YCR041W
YLR042C
MATALPHA1
MF(ALPHA)1
HMLALPHA2
MATALPHA2
SAG1
MF(ALPHA)2
RER1
CAF16

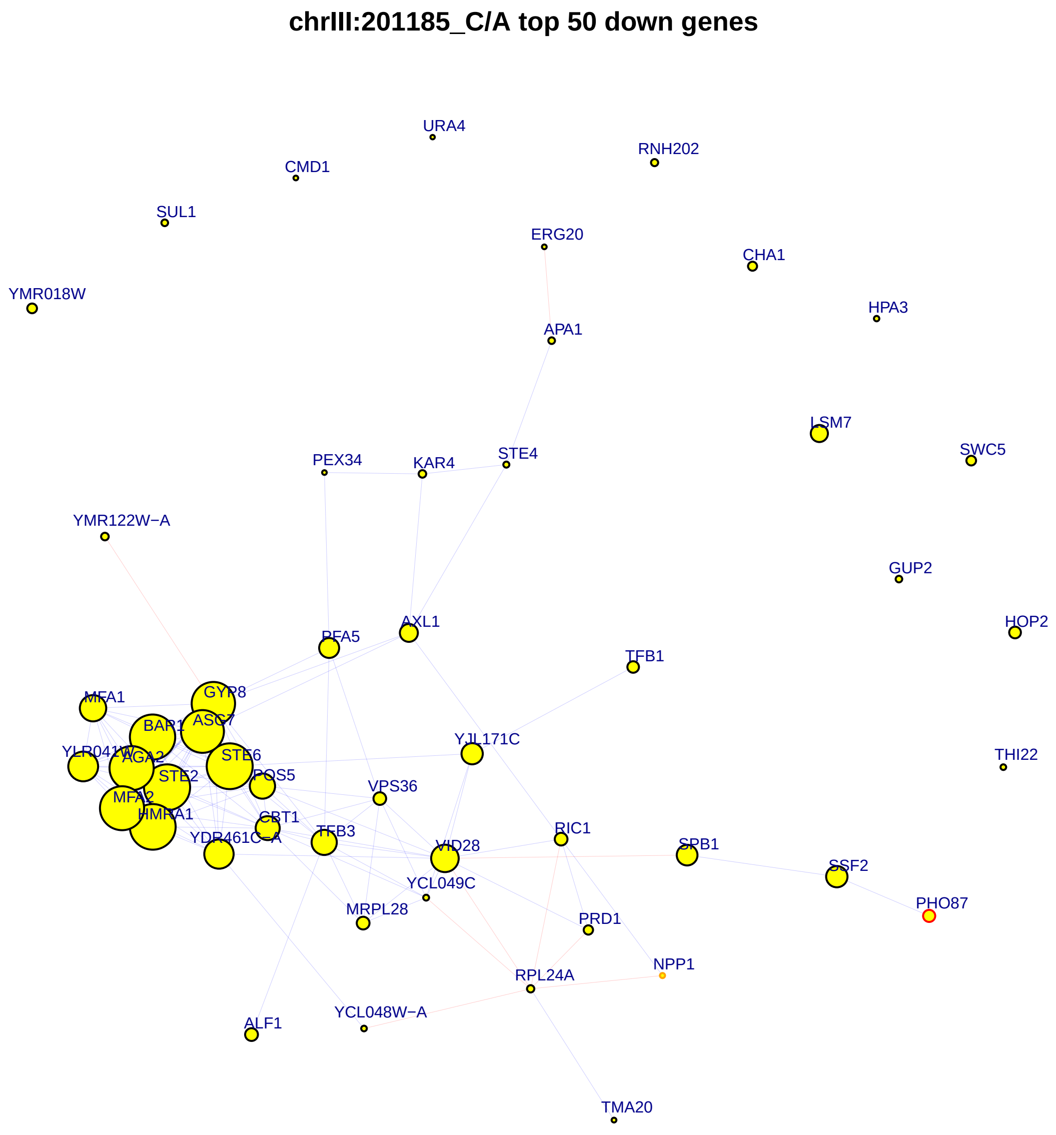
chrIII:201185_C/A top 50 down genes
URA4
CMD1
RNH202
SUL1
ERG20
CHA1
YMR018W
HPA3
APA1
LSM7
SWC5
PEX34
KAR4
STE4
YMR122W-A
GUP2
HOP2
PFA5
AXL1
TFB1
MFA1
GYP8
BAR1
ASG7
YJL171C
THI22
YLR041W
AGA2
STE6
POS5
STE2
VPS36
MFA2
CRT1
HMRA1
TFB3
RIC1
YDR461C-A
VID28
SPB1
YCL049C
SSF2
PHO87
MRPL28
PRD1
NPP1
RPL24A
ALF1
YCL048W-A
TMA20

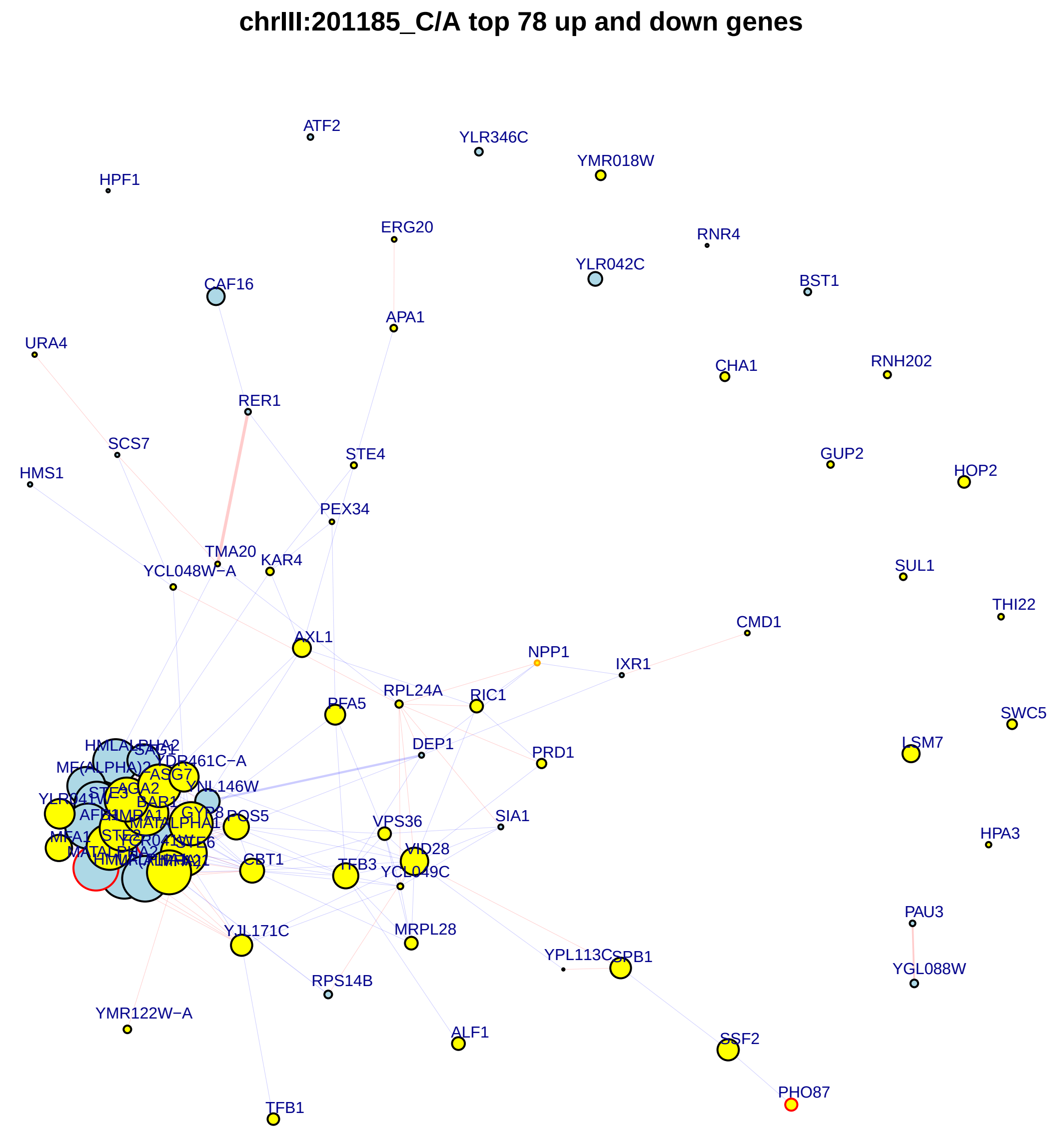
chrIII:201185_C/A top 78 up and down genes
ATF2
YLR346C
YMR018W
HPF1
ERG20
RNR4
YLR042C
BST1
CAF16
APA1
URA4
CHA1
RNH202
RER1
SCS7
STE4
GUP2
HOP2
HMS1
PEX34
TMA20
KAR4
YCL048W-A
SUL1
THI22
CMD1
AXL1
NPP1
IXR1
RPL24A
PFA5
RIC1
SWC5
DEP1
LSM7
PRD1
YDR461C-A
YNL146W
POS5
VPS36
SIA1
VID28
CRT1
TFB3
YCL049C
HPA3
YJL171C
MRPL28
YPL113C
SPB1
PAU3
YGL088W
RPS14B
YMR122W-A
ALF1
SSF2
PHO87
TFB1

chrIII:262105_C/T top 19 up genes

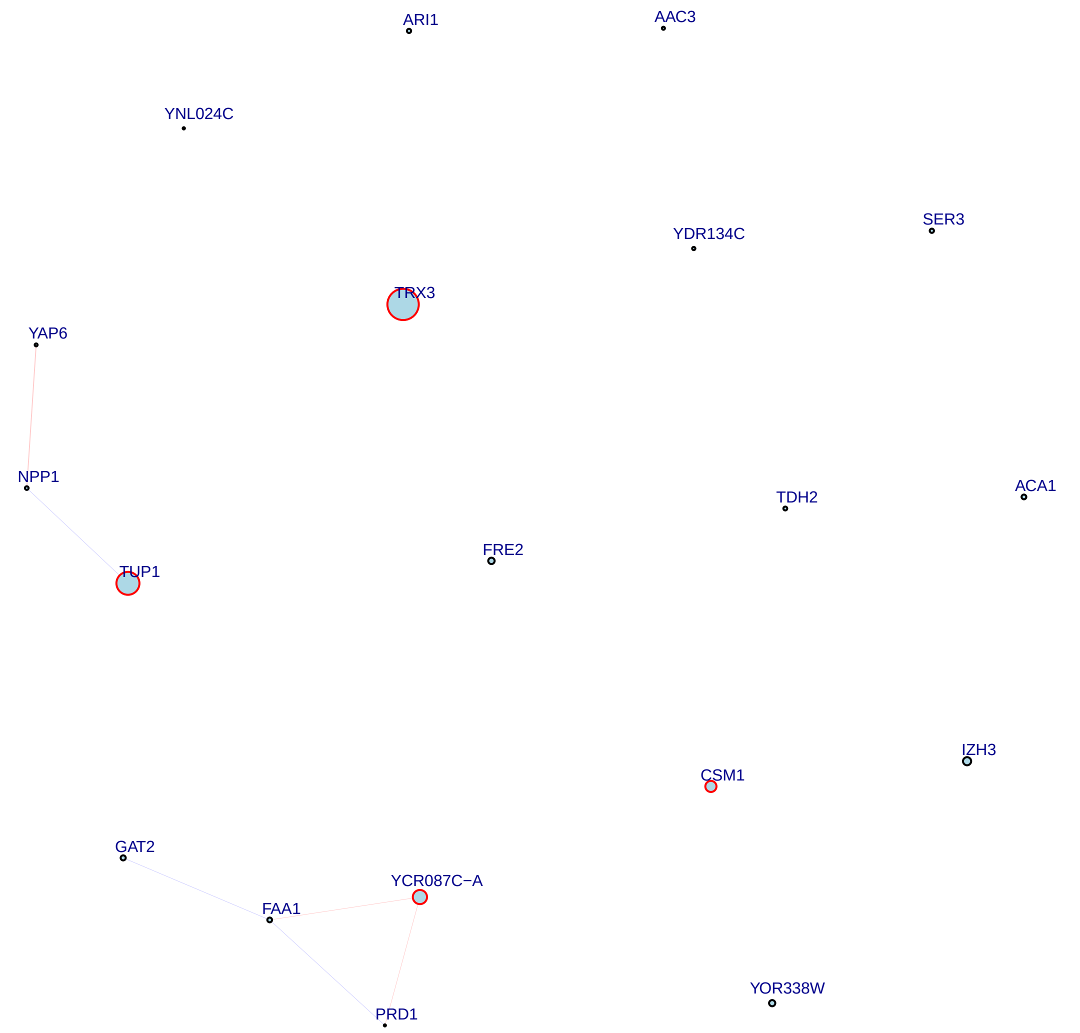

chrIII:262105_C/T top 28 down genes

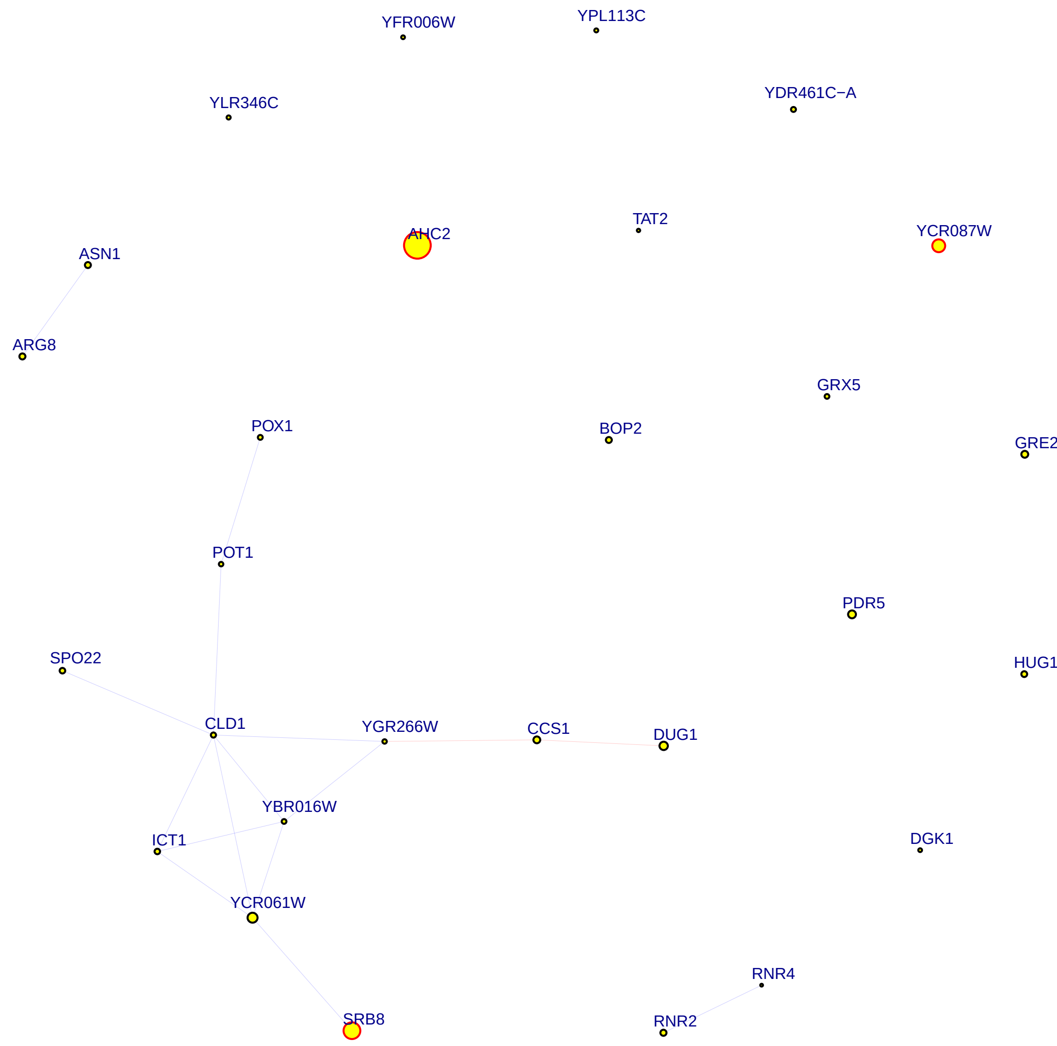

chrIII:262105_C/T top 47 up and down genes

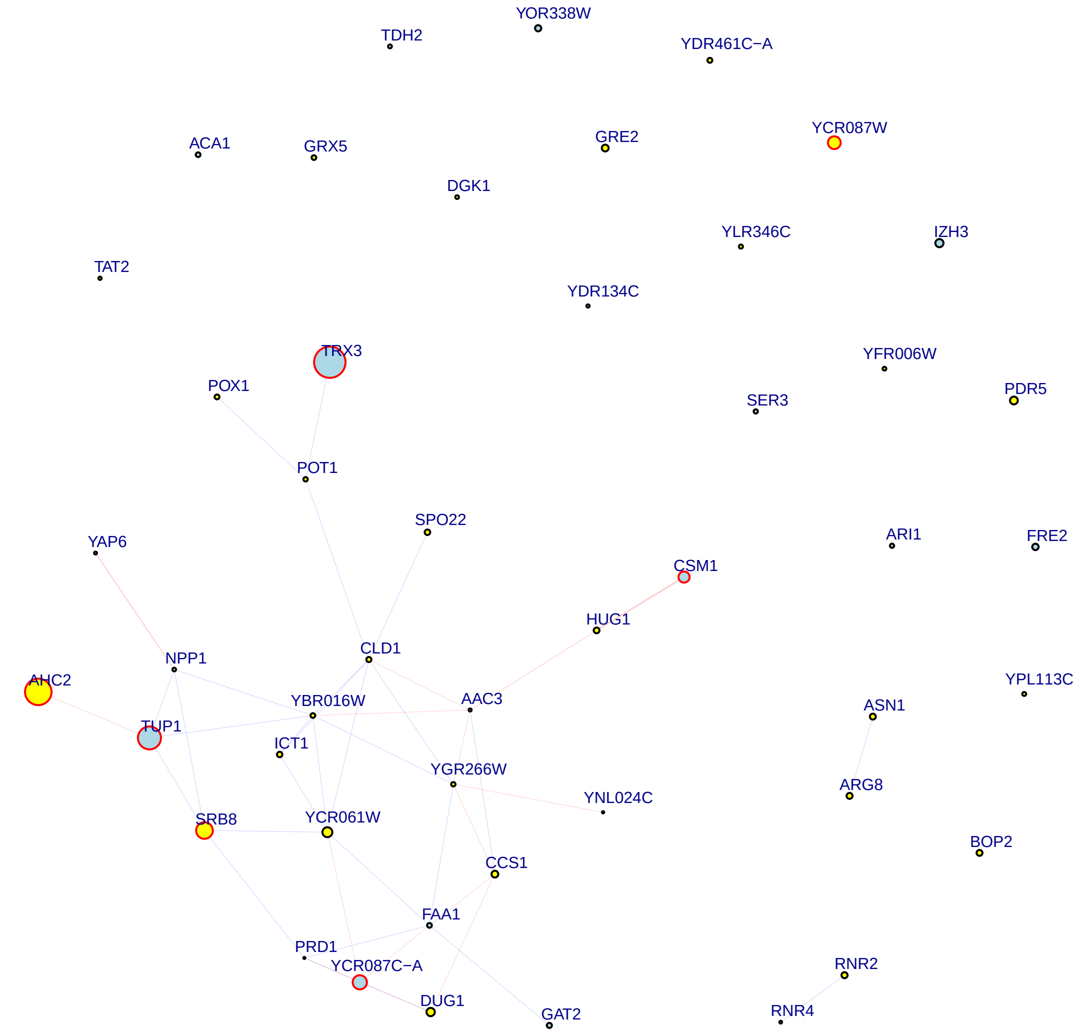

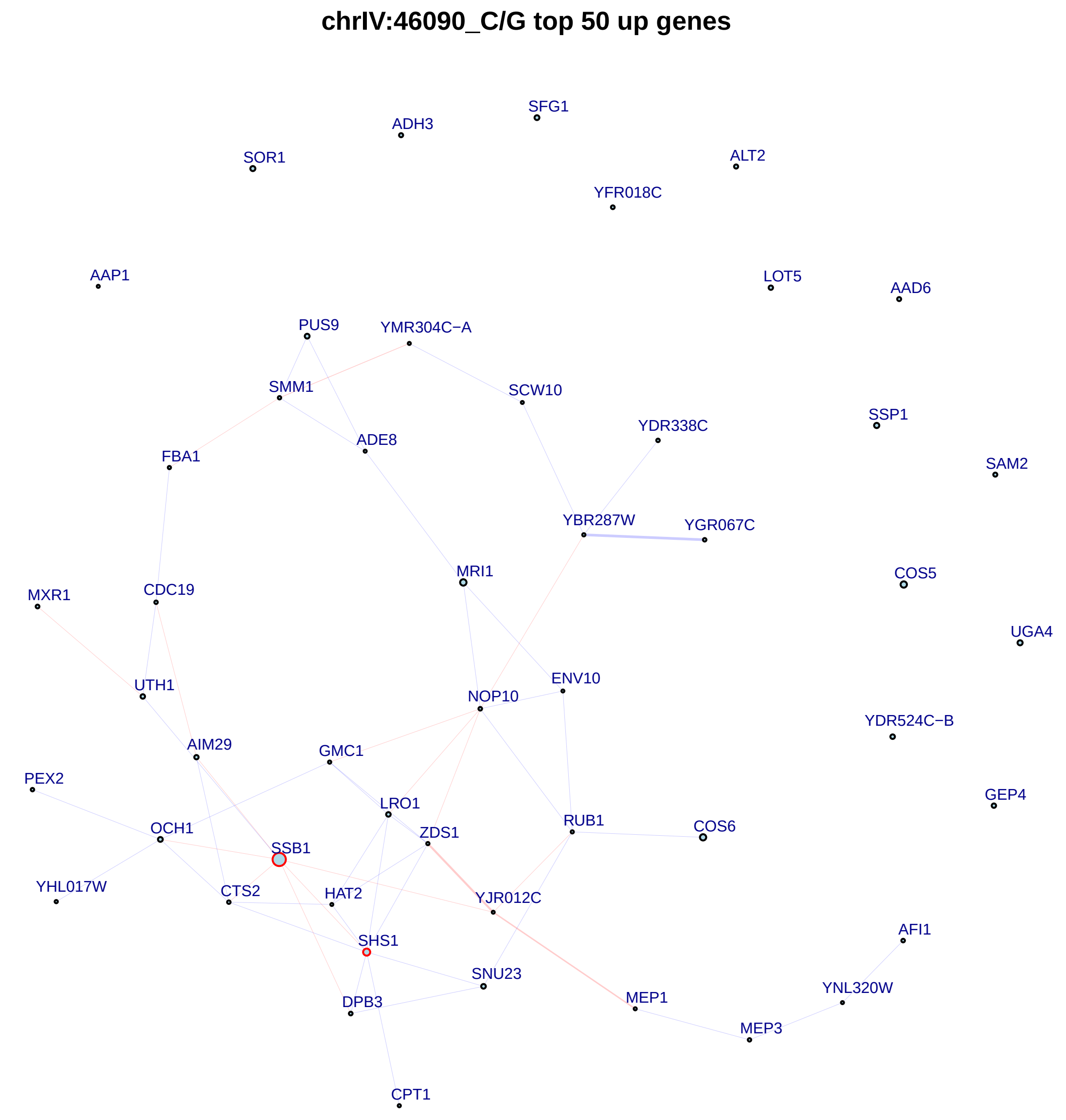
chrIV:46090_C/G top 50 up genes
SFG1
ADH3
SOR1
ALT2
YFR018C
AAP1
LOT5
AAD6
PUS9
YMR304C−A
SMM1
SCW10
YDR338C
SSP1
ADE8
FBA1
SAM2
YBR287W
YGR067C
MRI1
COS5
MXR1
CDC19
UGA4
ENV10
UTH1
NOP10
YDR524C−B
AIM29
GMC1
PEX2
LRO1
GEP4
ZDS1
RUB1
COS6
OCH1
SSB1
YHL017W
CTS2
HAT2
YJR012C
SHS1
AFI1
SNU23
YNL320W
MEP1
DPB3
MEP3
CPT1

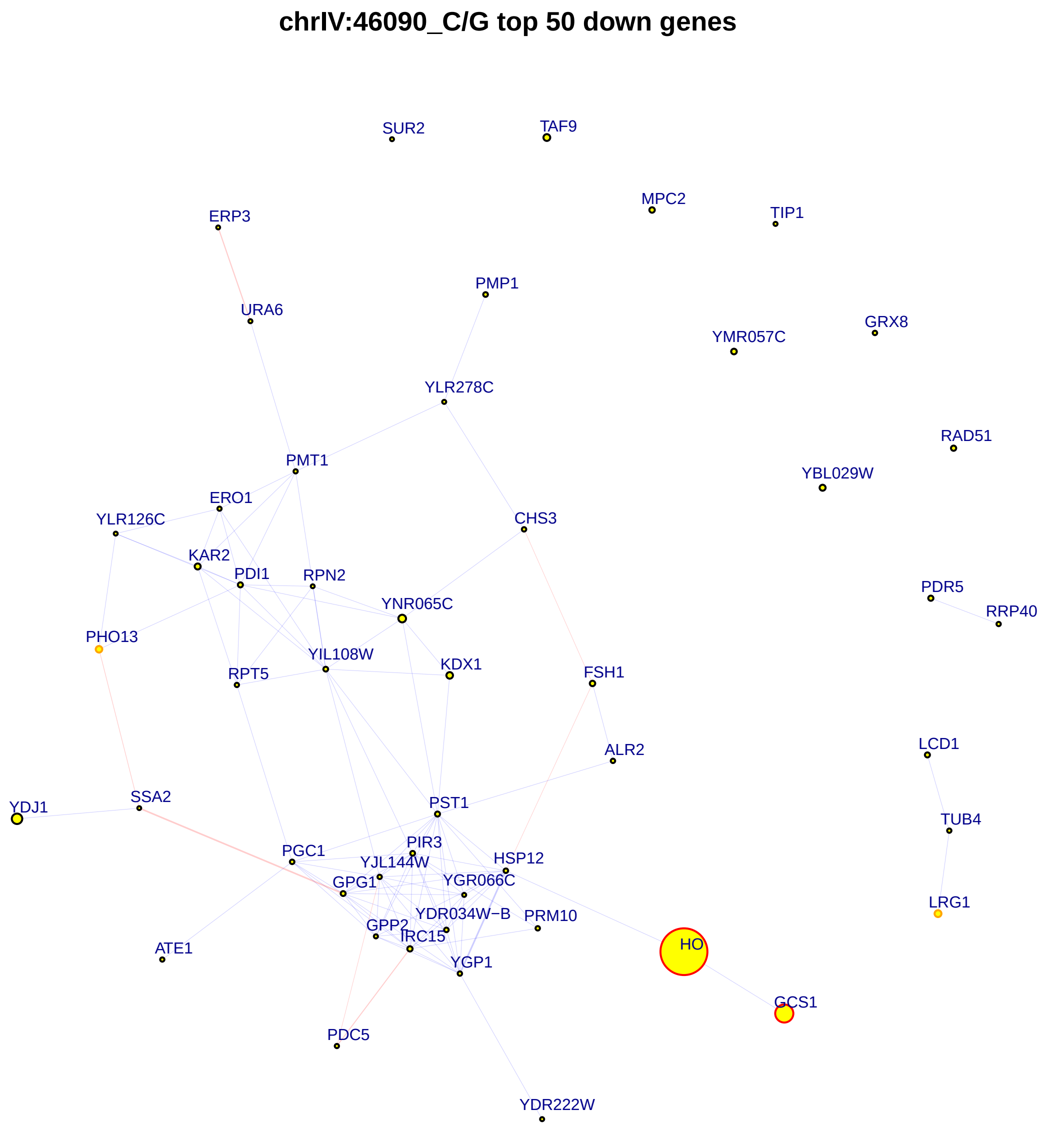
chrIV:46090_C/G top 50 down genes
SUR2
TAF9
MPC2
TIP1
ERP3
URA6
PMP1
YMR057C
GRX8
YLR278C
RAD51
YBL029W
PMT1
ERO1
YLR126C
CHS3
KAR2
PDI1
RPN2
YNR065C
PDR5
RRP40
PHO13
YIL108W
KDX1
RPT5
FSH1
LCD1
ALR2
PST1
TUB4
YDJ1
SSA2
PIR3
PGC1
YJL144W
HSP12
GPG1
YGR066C
LRG1
YDR034W−B
PRM10
GPP2
IRC15
ATE1
HO
YGP1
GCS1
PDC5
YDR222W

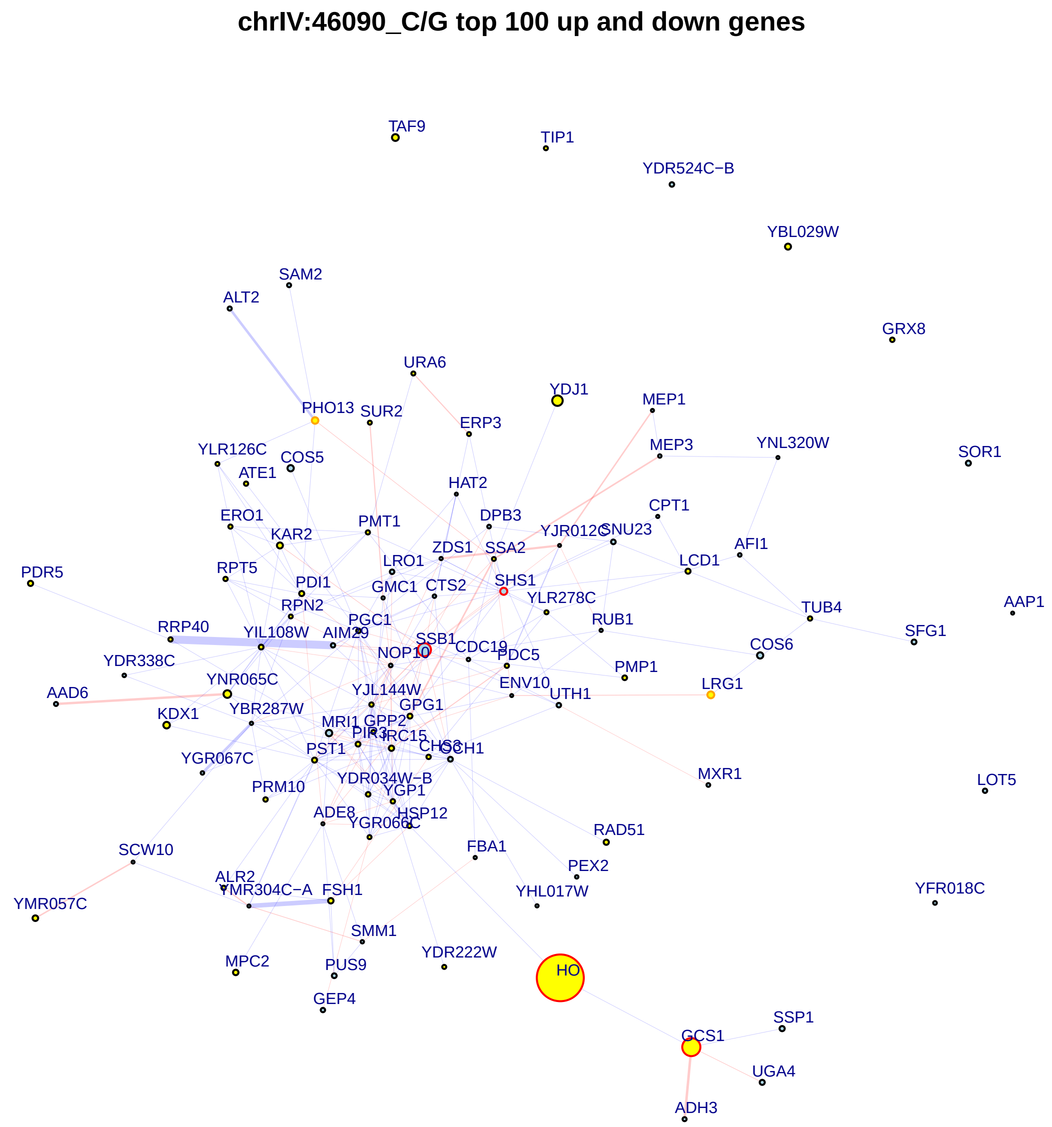
chrIV:46090_C/G top 100 up and down genes
TAF9
TIP1
YDR524C−B
YBL029W
SAM2
ALT2
GRX8
URA6
YDJ1
MEP1
PHO13
SUR2
ERP3
MEP3
YNL320W
SOR1
YLR126C
COS5
ATE1
HAT2
CPT1
ERO1
PMT1
DPB3
KAR2
SNU23
AFI1
PDR5
LCD1
AAP1
TUB4
SFG1
COS6
PMP1
LRG1
YDR338C
AAD6
KDX1
MXR1
LOT5
RAD51
PEX2
YHL017W
YFR018C
SCW10
YMR057C
FBA1
MPC2
PUS9
GEP4
YDR222W
HO
GCS1
SSP1
UGA4
ADH3

chrIV:156943_A/G top 50 up genes

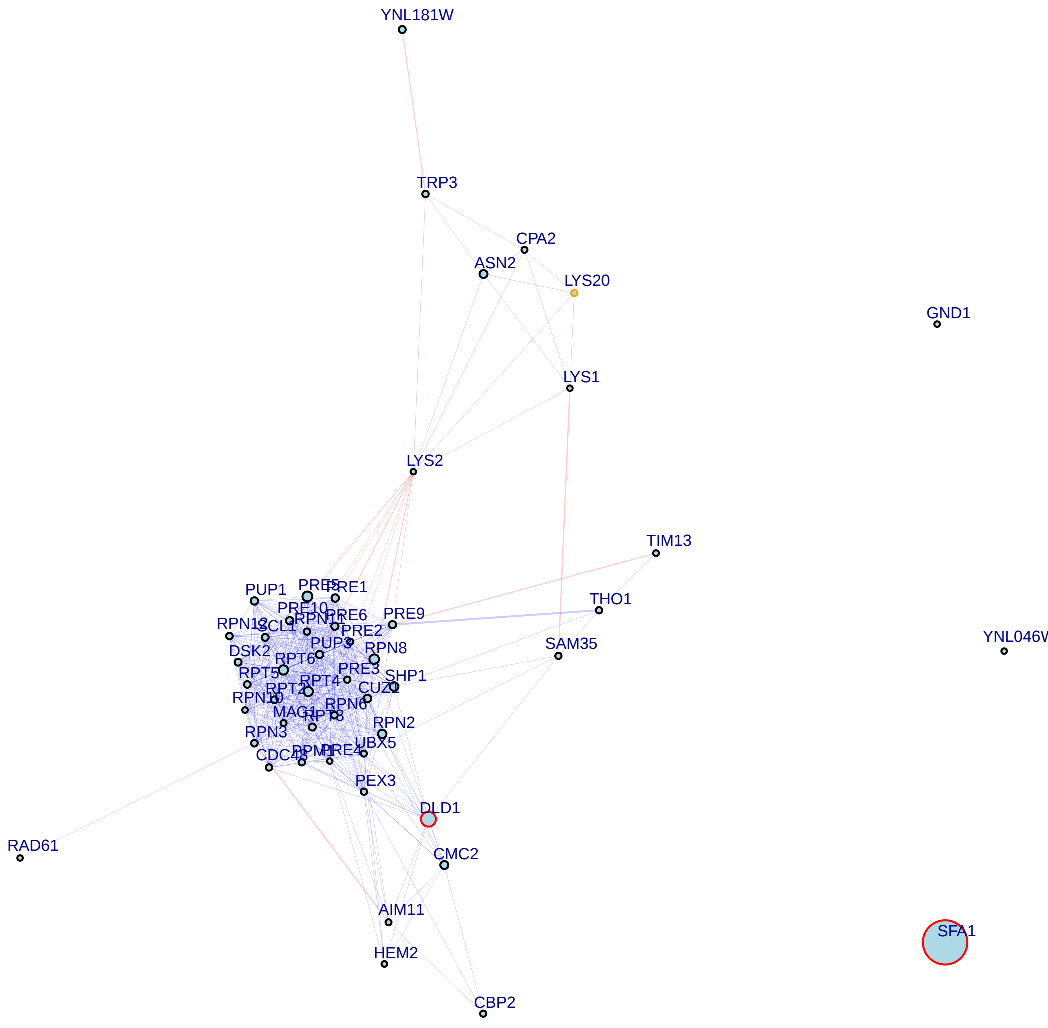

chrIV:156943_A/G top 50 down genes

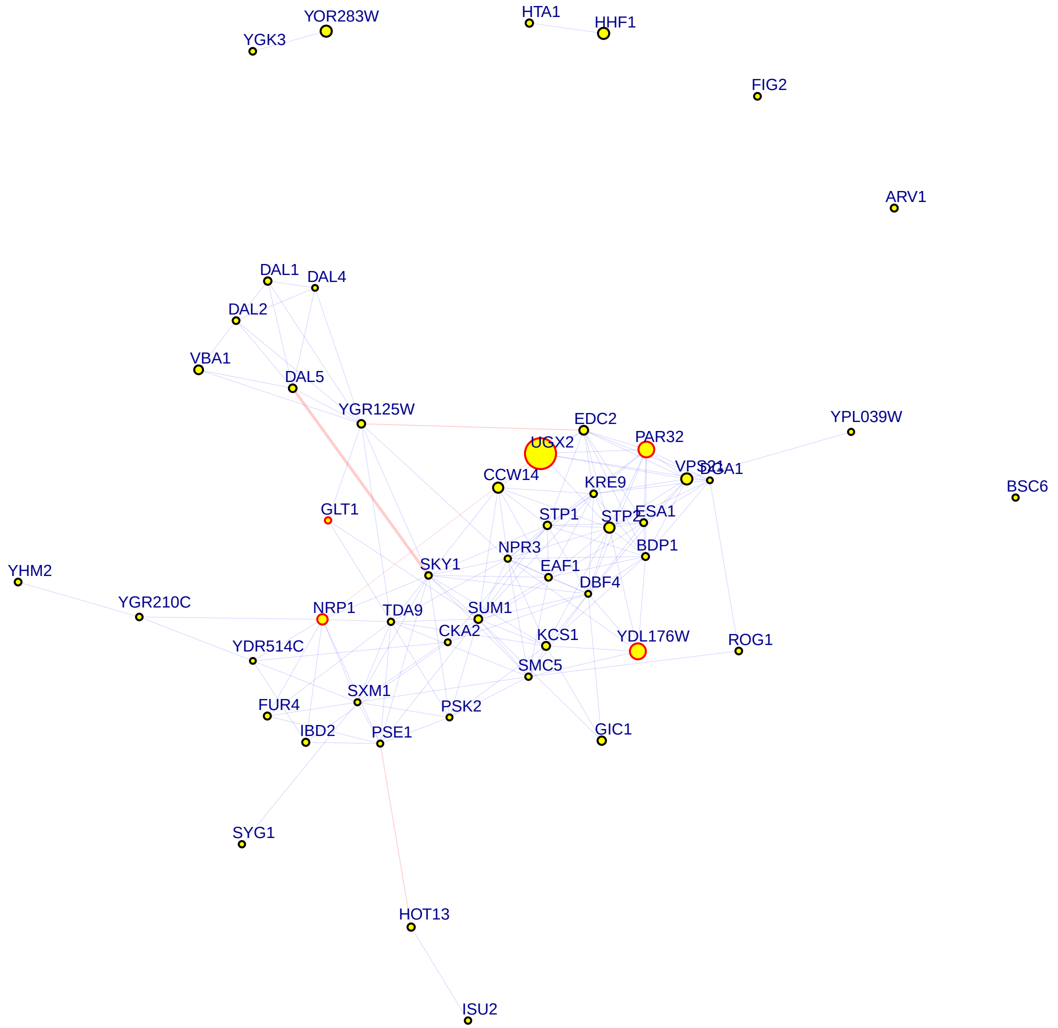

chrIV:156943_A/G top 100 up and down genes

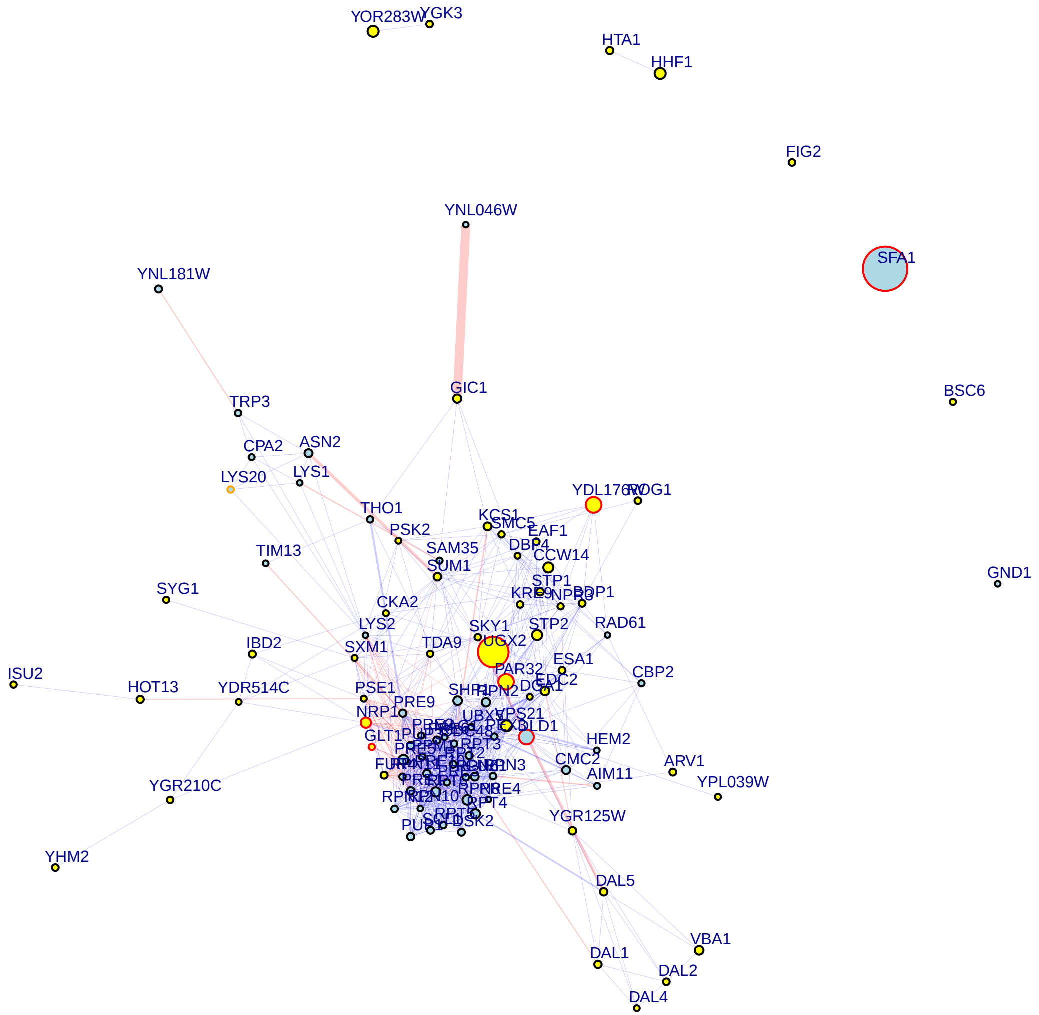

chrIV:215523_T/C top 50 up genes

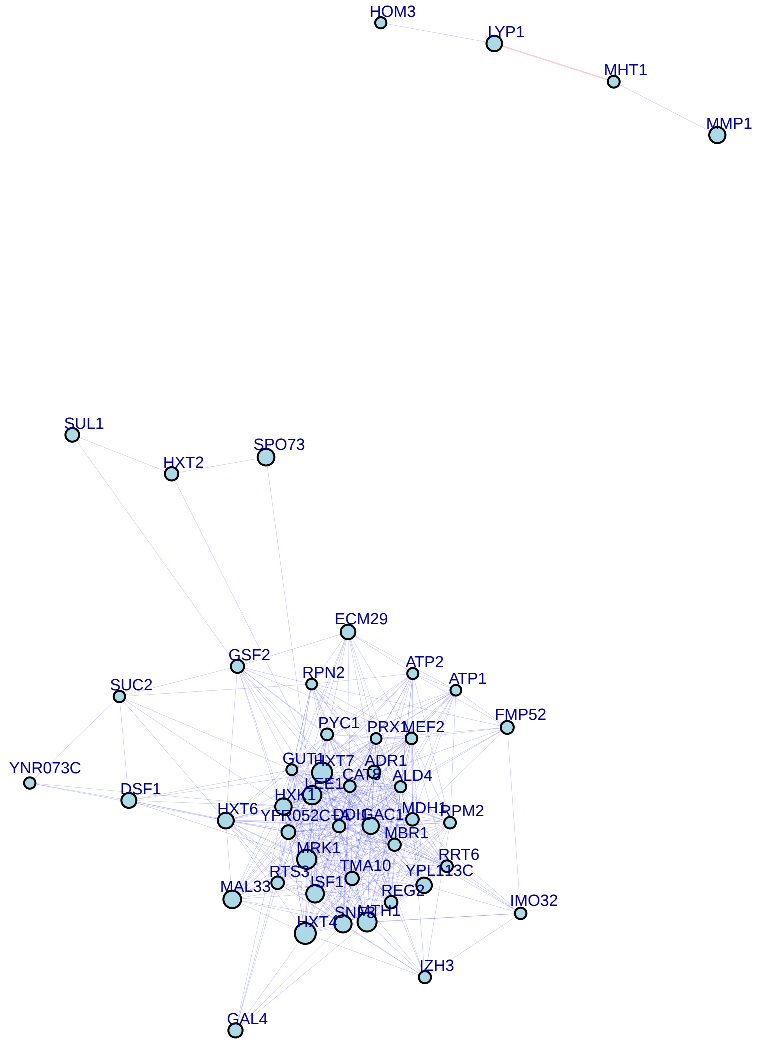

chrIV:215523_T/C top 50 down genes

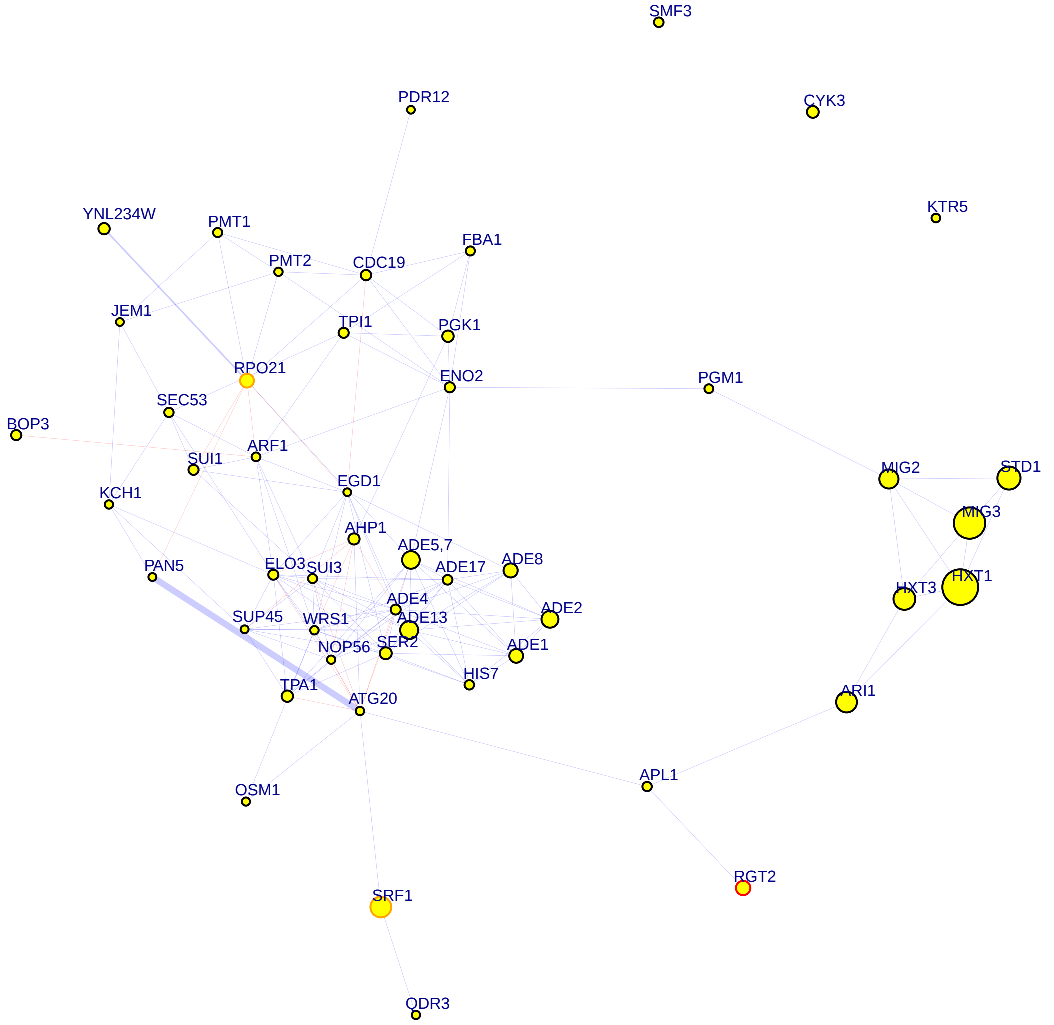

chrIV:215523_T/C top 100 up and down genes

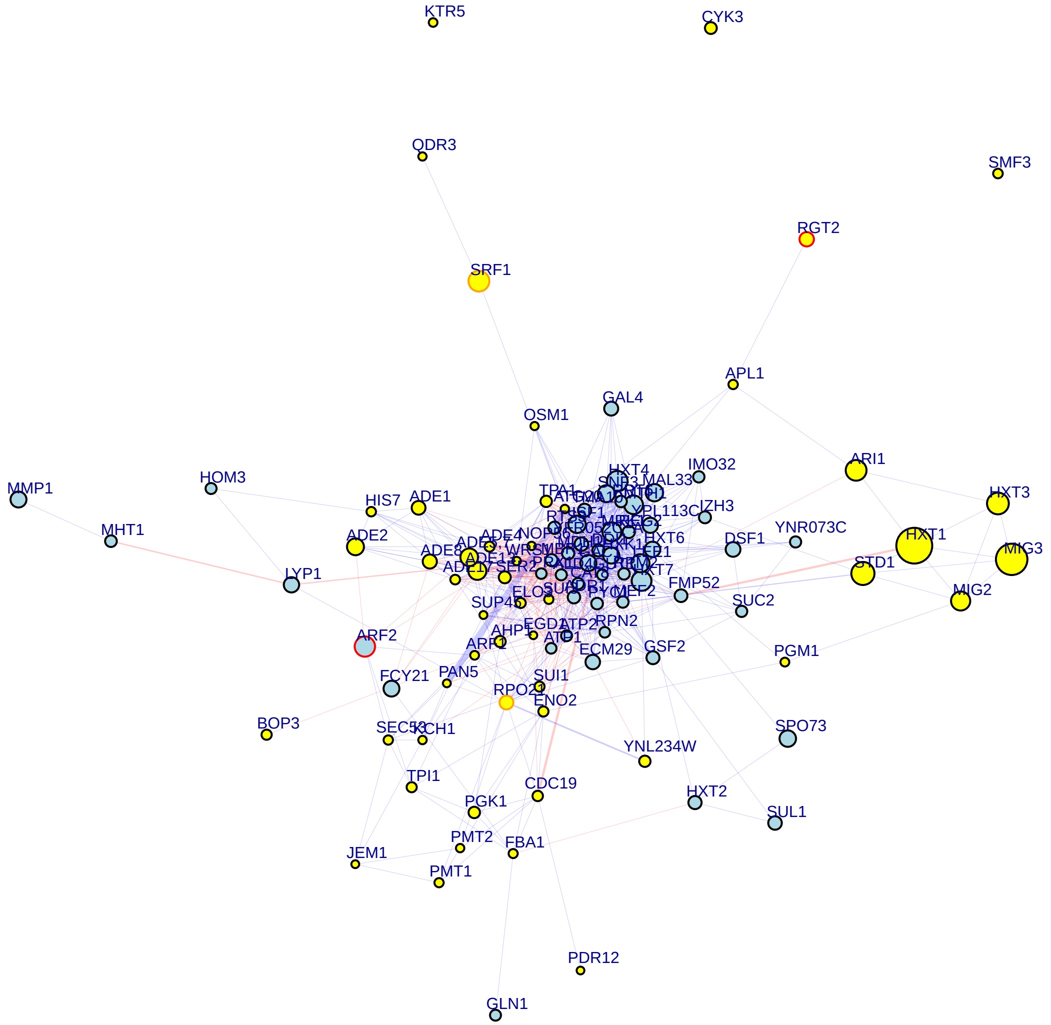

chrIV:461656_A/G top 50 up genes

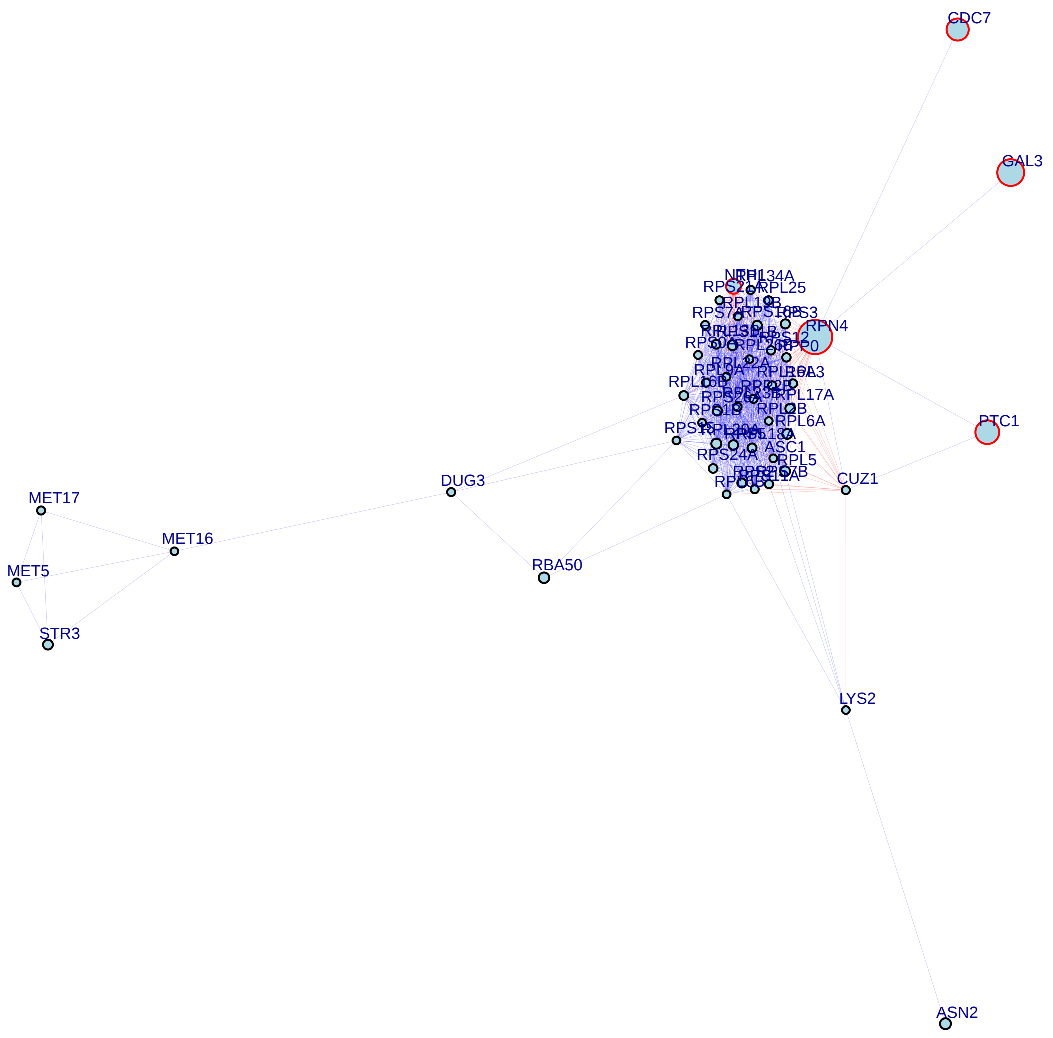

chrIV:461656_A/G top 50 down genes

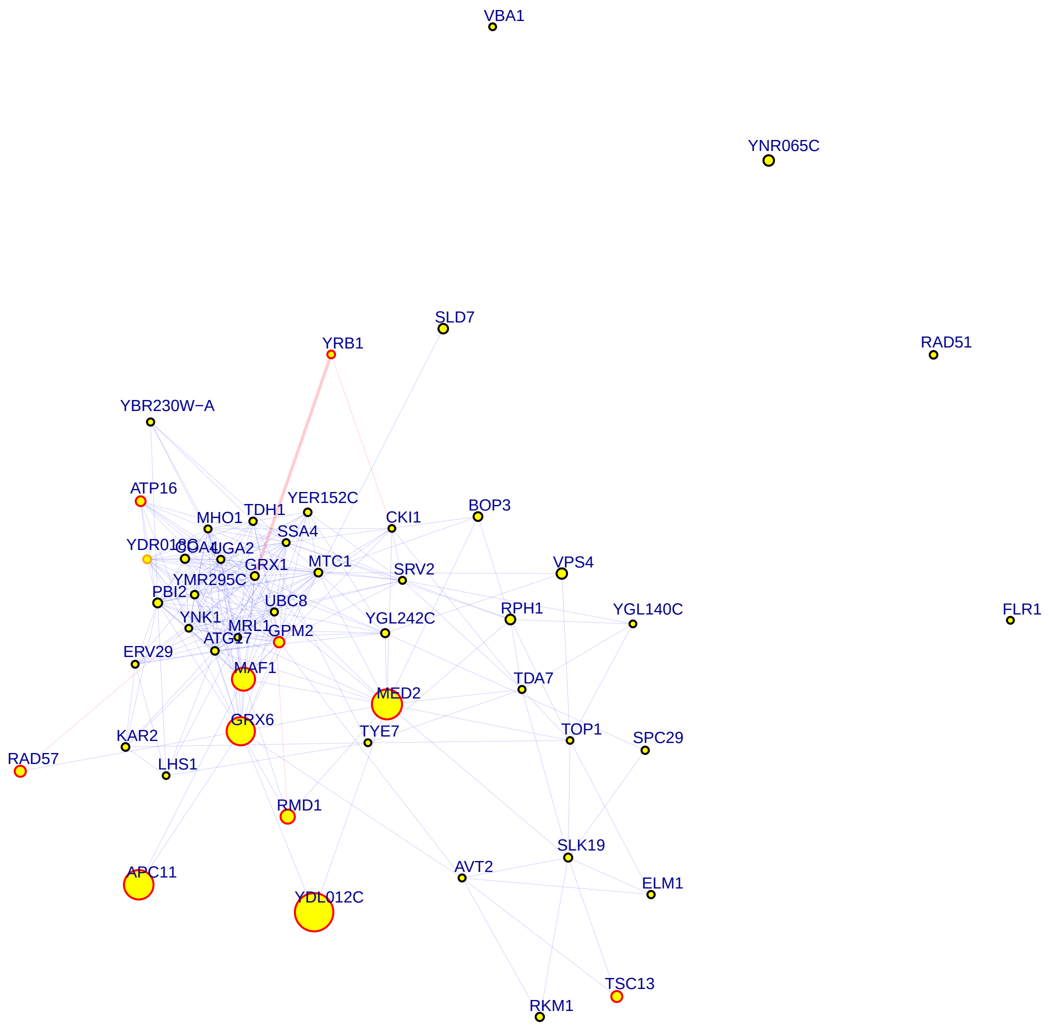

chrIV:461656_A/G top 100 up and down genes

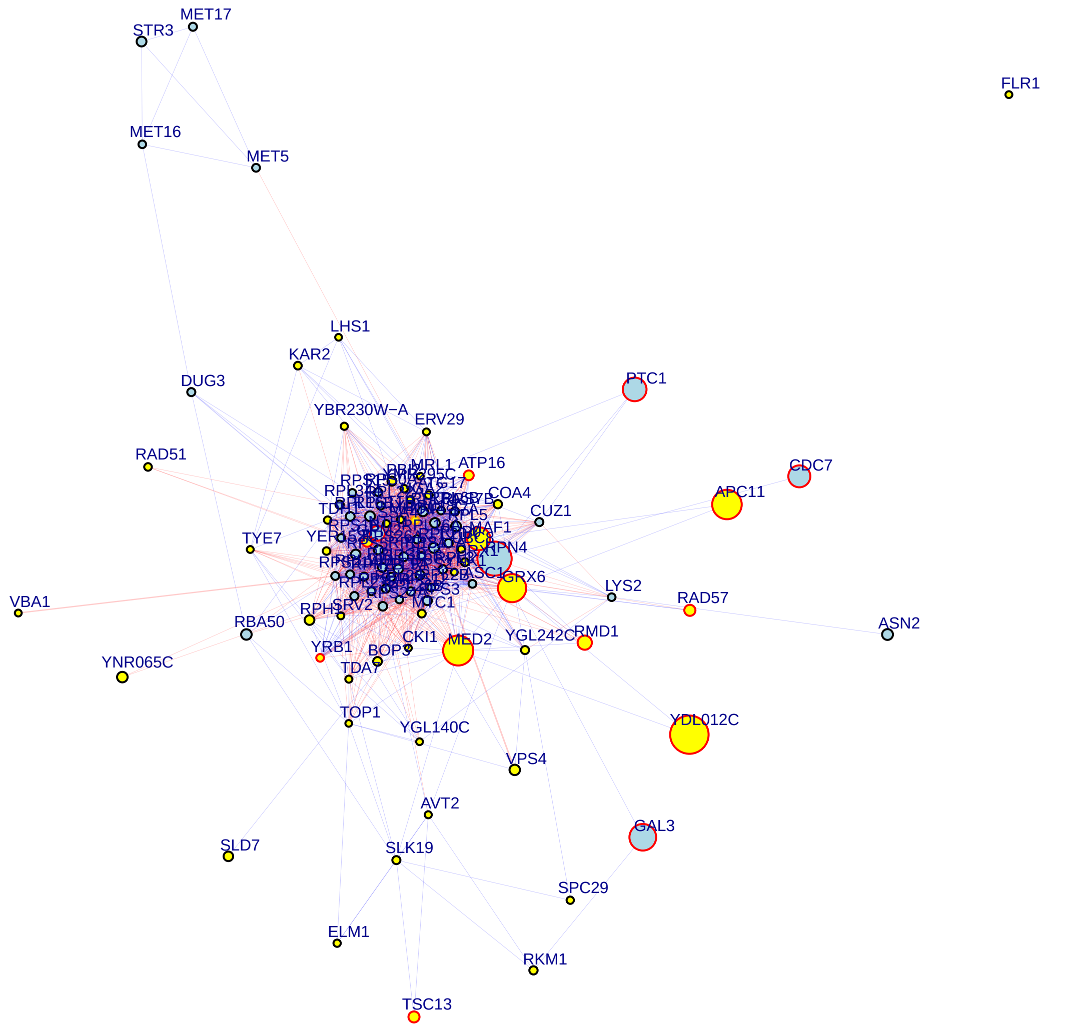

chrIV:509407_G/T top 50 up genes

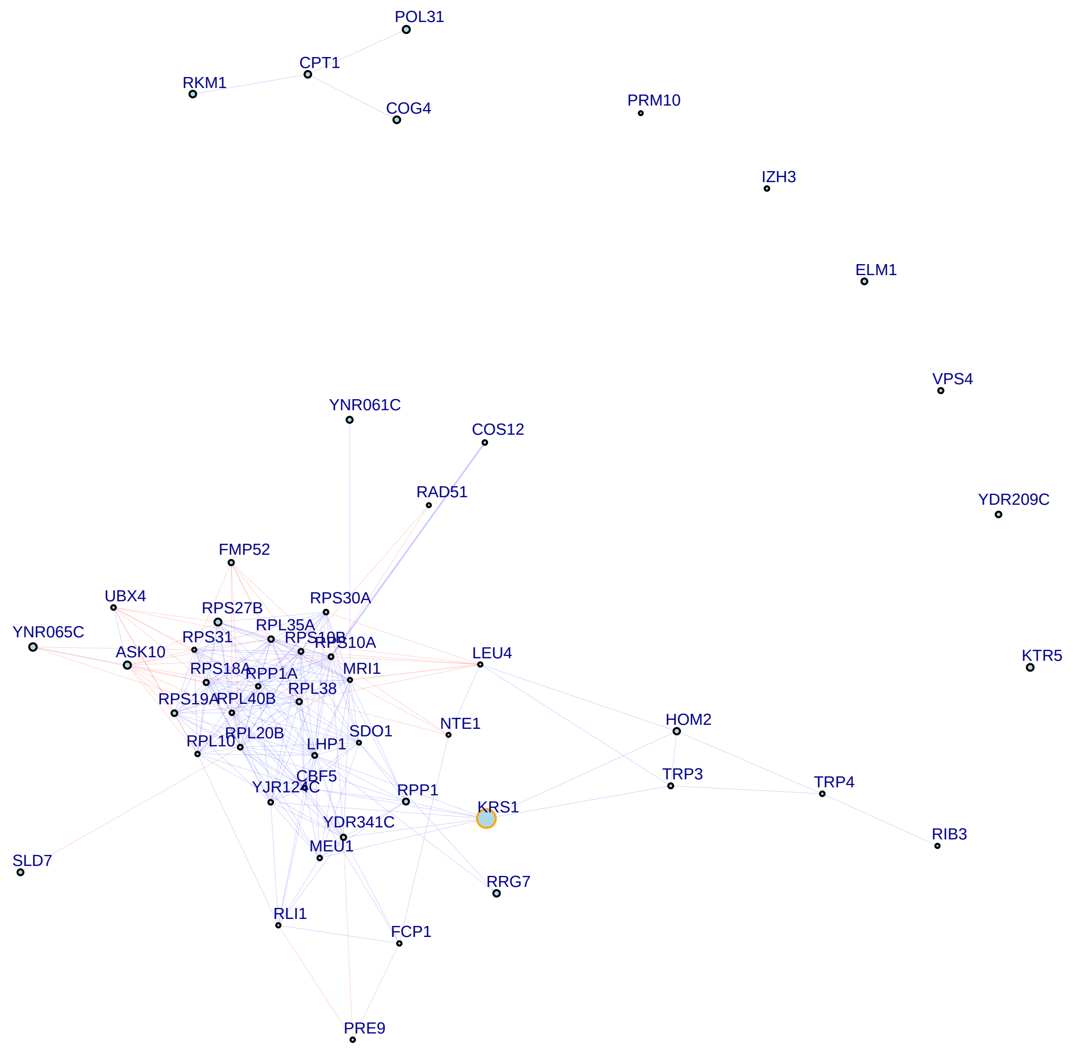

chrIV:509407_G/T top 50 down genes

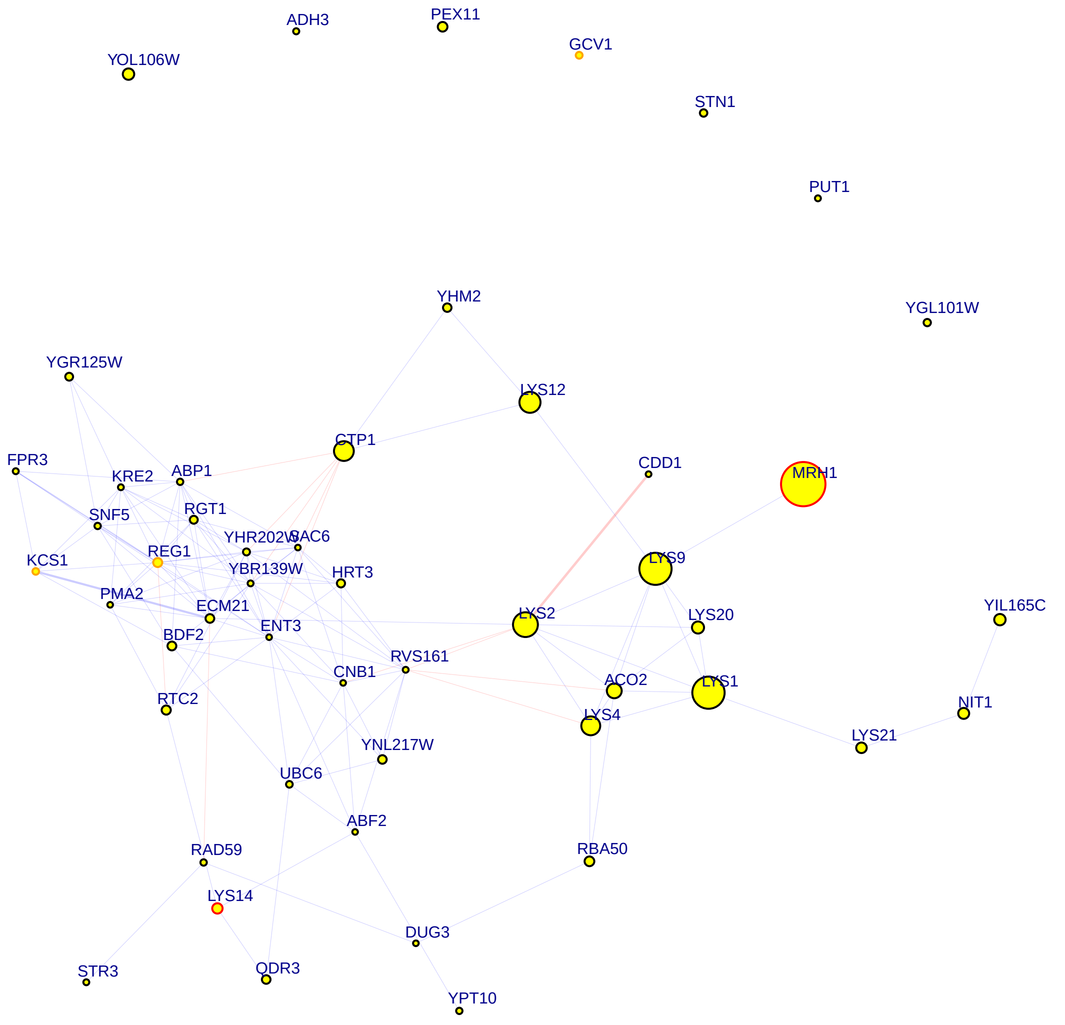

chrIV:509407_G/T top 100 up and down genes

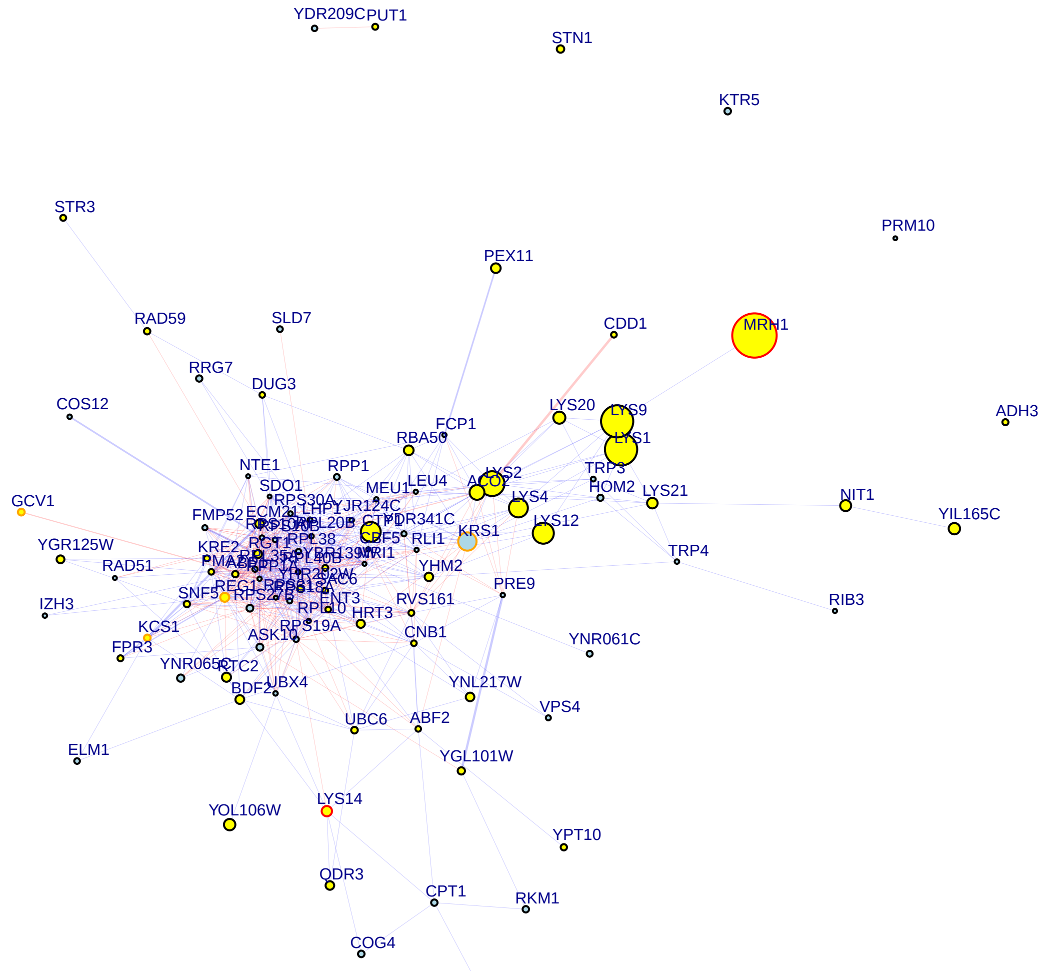

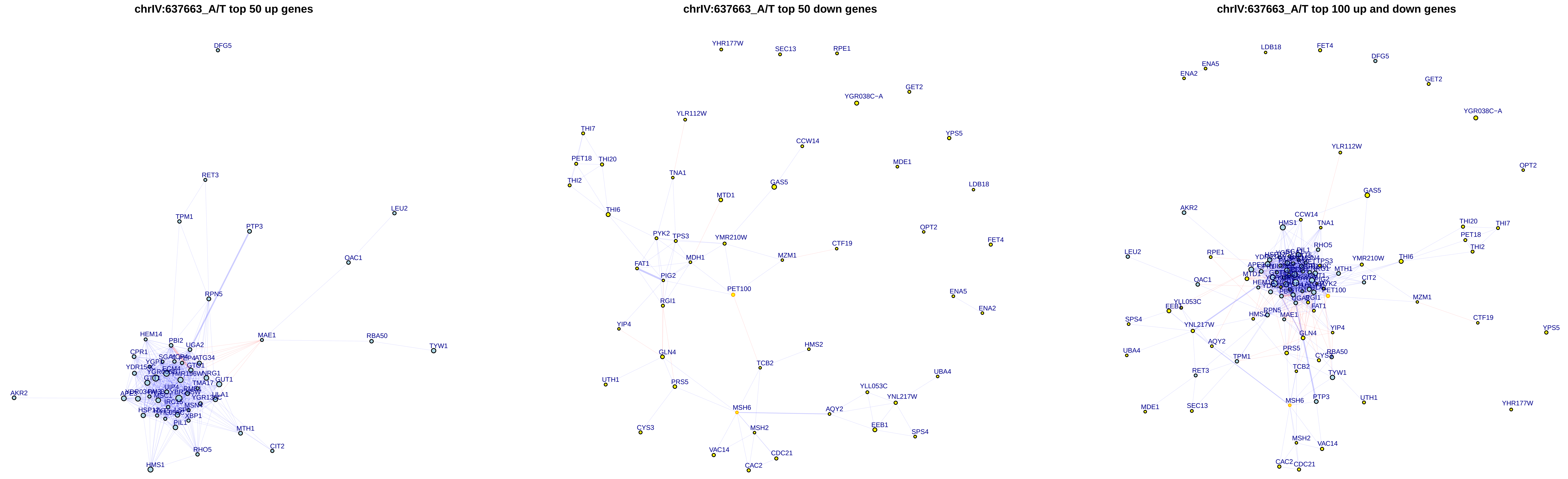
chrIV:637663_A/T top 50 up genes
DFG5
RET3
TPM1
PTP3
LEU2
QAC1
RPN5
MAE1
RBA50
TYW1
HEM14
PBI2
UGA2
CPR1
ATG34
YDR15
GUT1
NRG1
TMA17
AKR2
APE3
YGR130C
ULA1
MSN4
XBP1
PIL1
MTH1
CIT2
RHO5
HMS1
chrIV:637663_A/T top 50 down genes
YHR177W
SEC13
RPE1
GET2
YGR038C−A
YLR112W
THI7
PET18
THI20
THI2
CCW14
YPS5
MDE1
TNA1
GAS5
LDB18
MTD1
THI6
OPT2
PYK2
TPS3
YMR210W
FET4
CTF19
MDH1
MZM1
FAT1
PIG2
PET100
ENA5
RGI1
ENA2
YIP4
HMS2
GLN4
TCB2
UBA4
UTH1
PRS5
YLL053C
YNL217W
MSH6
AQY2
EEB1
SPS4
CYS3
MSH2
VAC14
CDC21
CAC2
chrIV:637663_A/T top 100 up and down genes
LDB18
FET4
ENA5
DFG5
ENA2
GET2
YGR038C−A
YLR112W
OPT2
GAS5
AKR2
CCW14
HMS1
TNA1
THI20
THI7
PET18
RHO5
LEU2
RPE1
THI2
THI6
YMR210W
MTH1
CIT2
QAC1
PET100
MZM1
YLL053C
EEB1
FAT1
SPS4
YNL217W
HMS2
RPN5
MAE1
CTF19
YPS5
YIP4
AQY2
GLN4
UBA4
PRS5
RBA50
TPM1
TCB2
RET3
TYW1
PTP3
UTH1
SEC13
MDE1
MSH6
YHR177W
MSH2
VAC14
CAC2
CDC21

chrIV:681304_G/A top 50 up genes

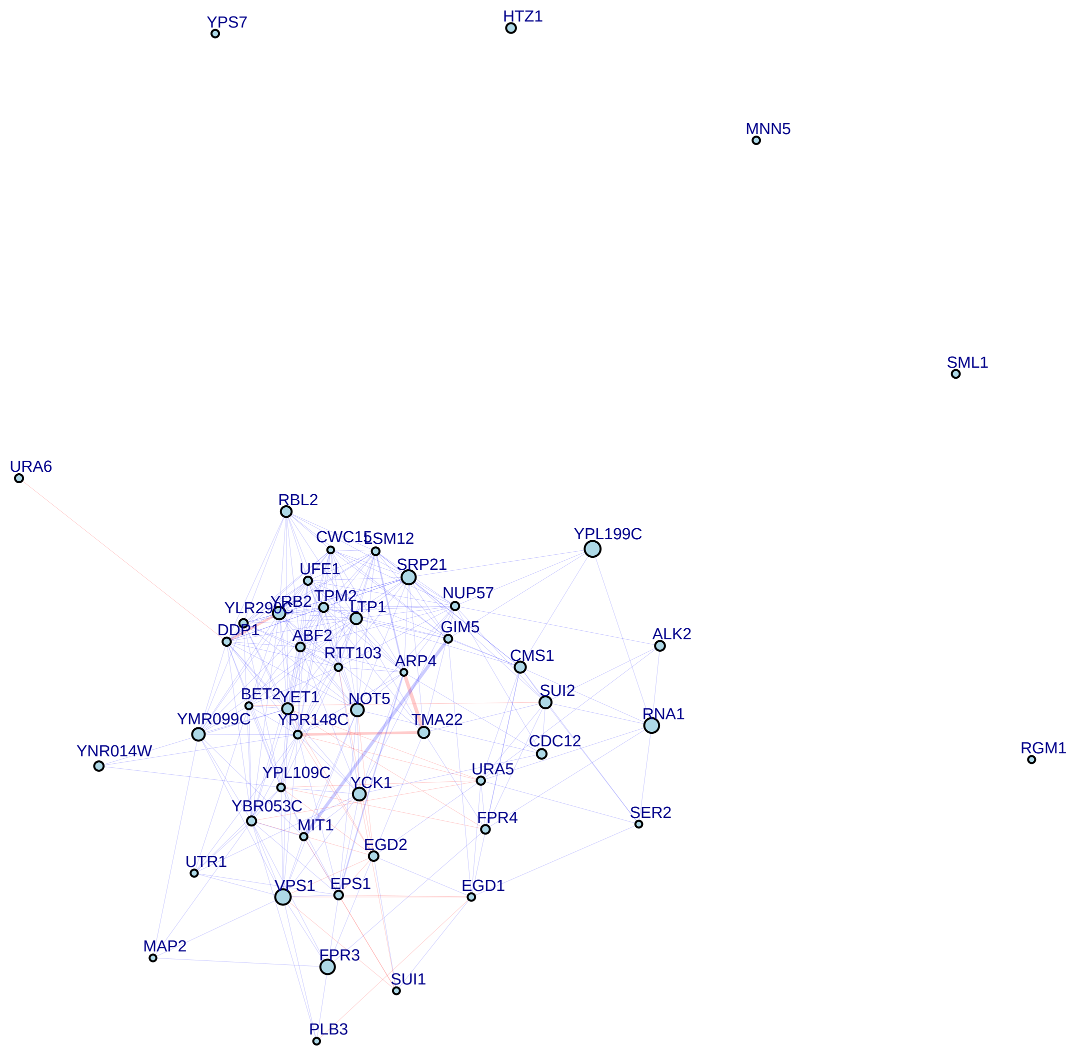

chrIV:681304_G/A top 50 down genes

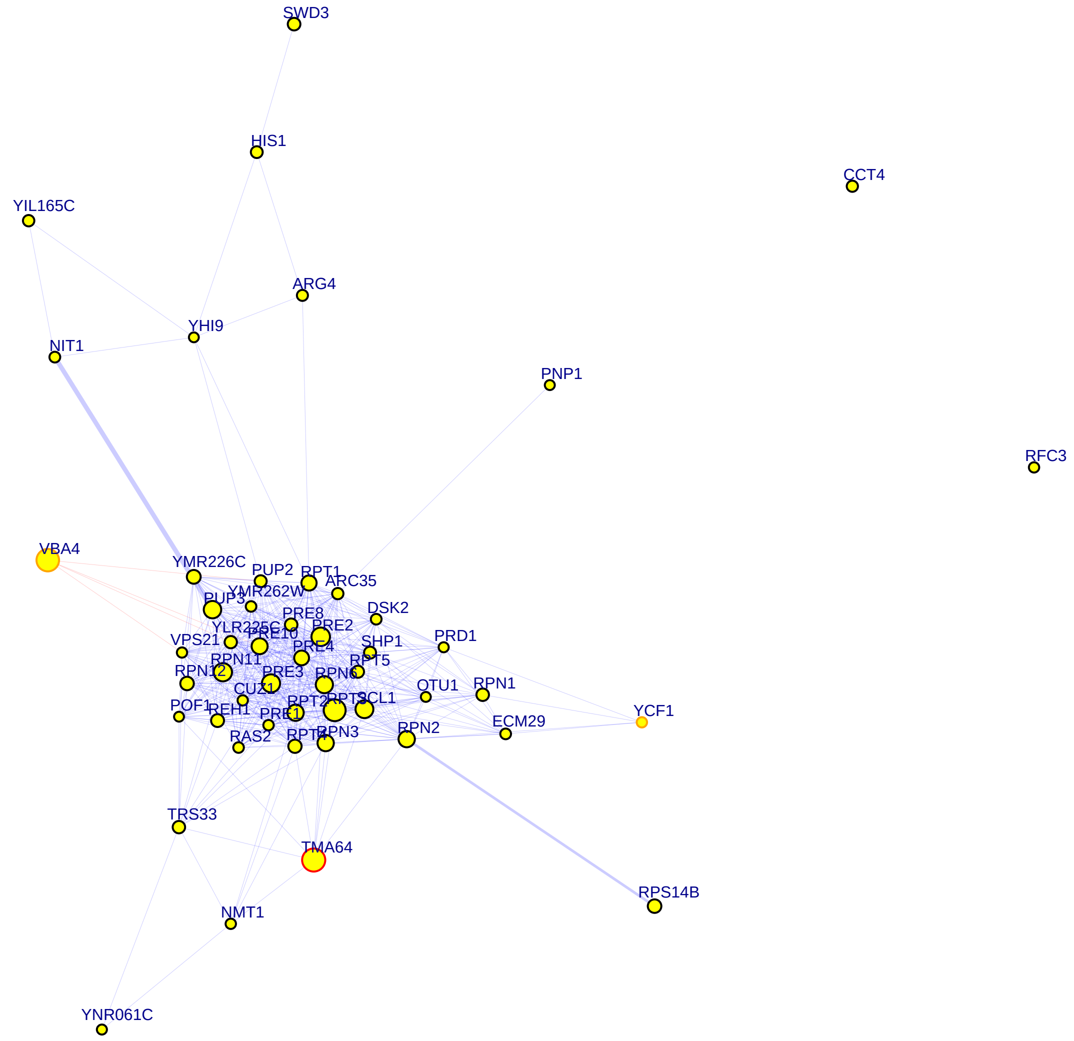

chrIV:681304_G/A top 100 up and down genes

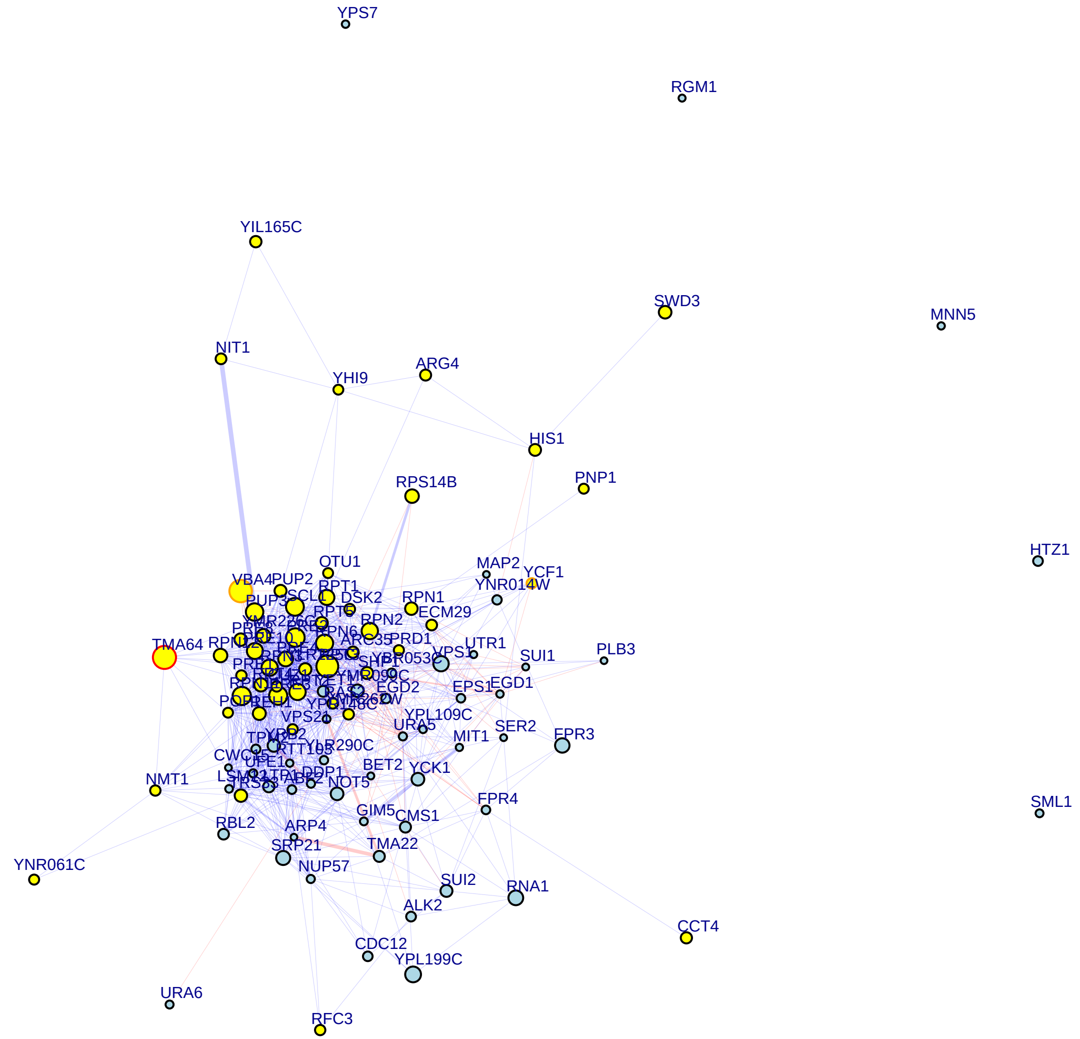

chrIV:745766_G/A top 50 up genes

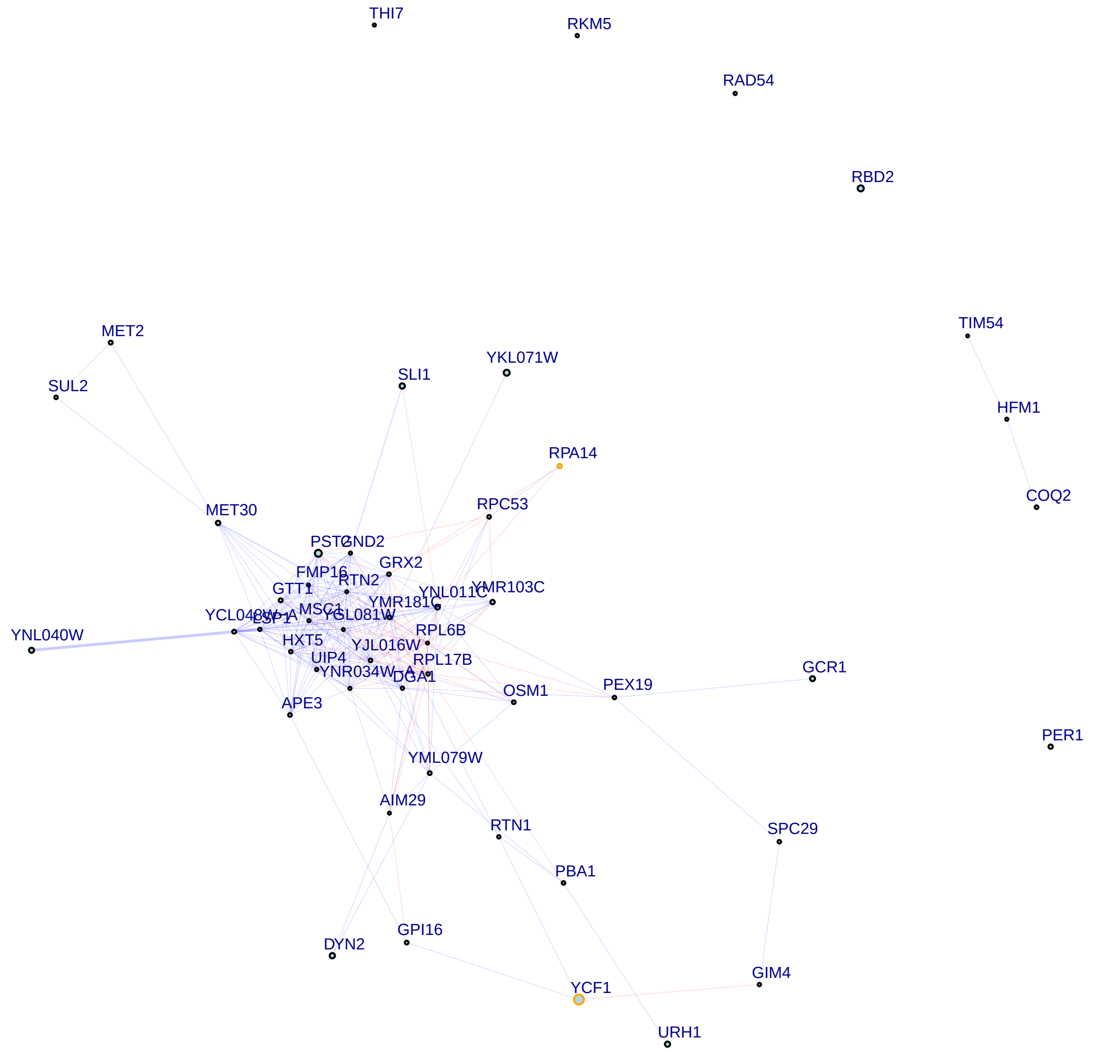

chrIV:745766_G/A top 50 down genes

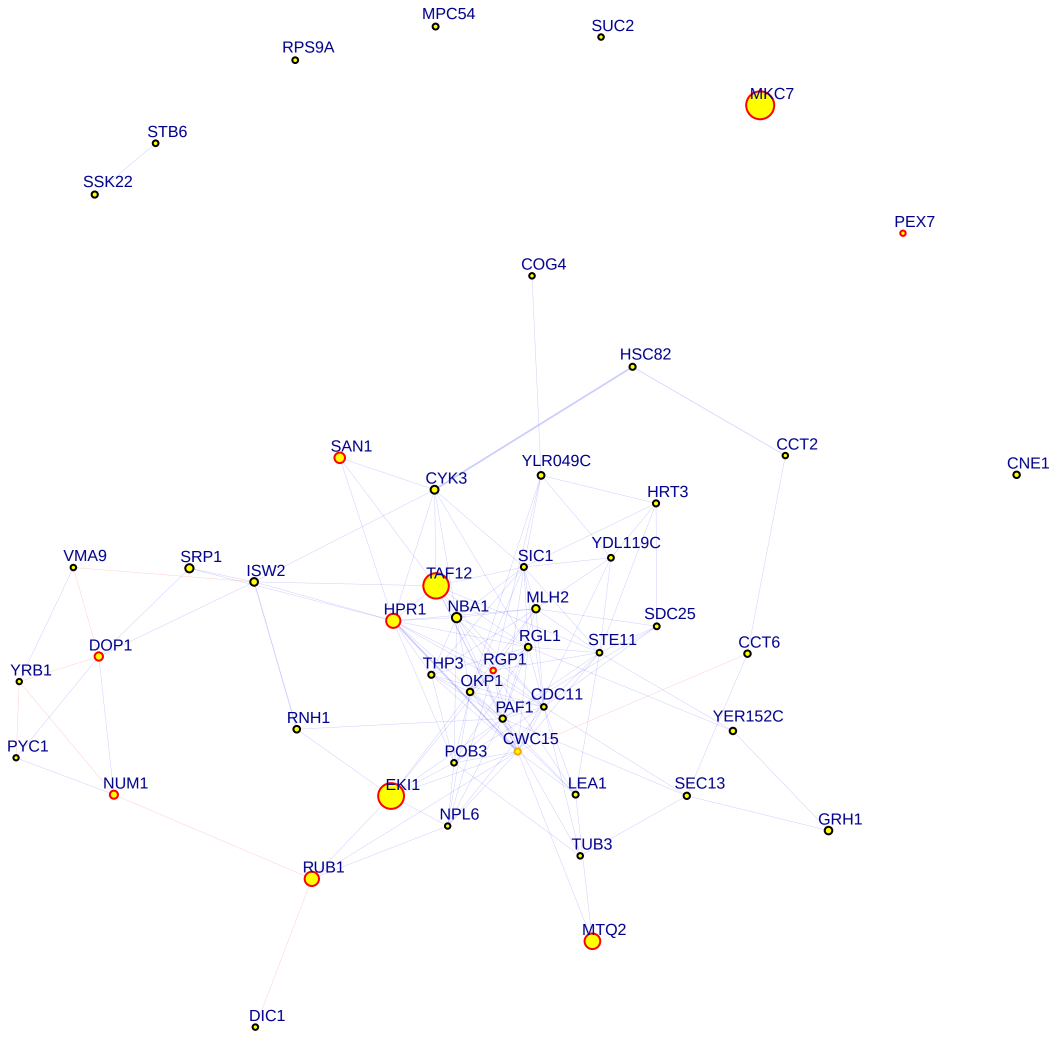

chrIV:745766_G/A top 100 up and down genes

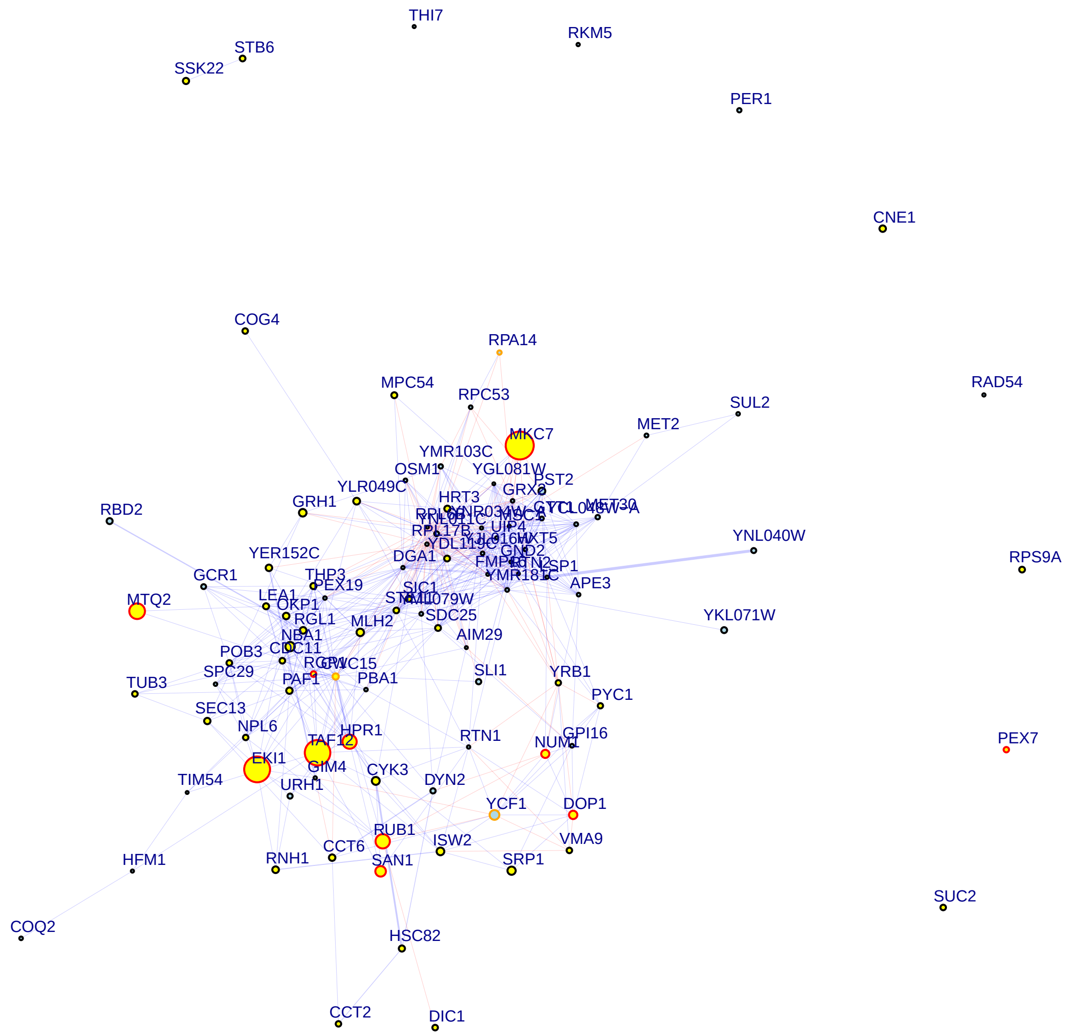

chrIV:975156_A/G top 50 up genes

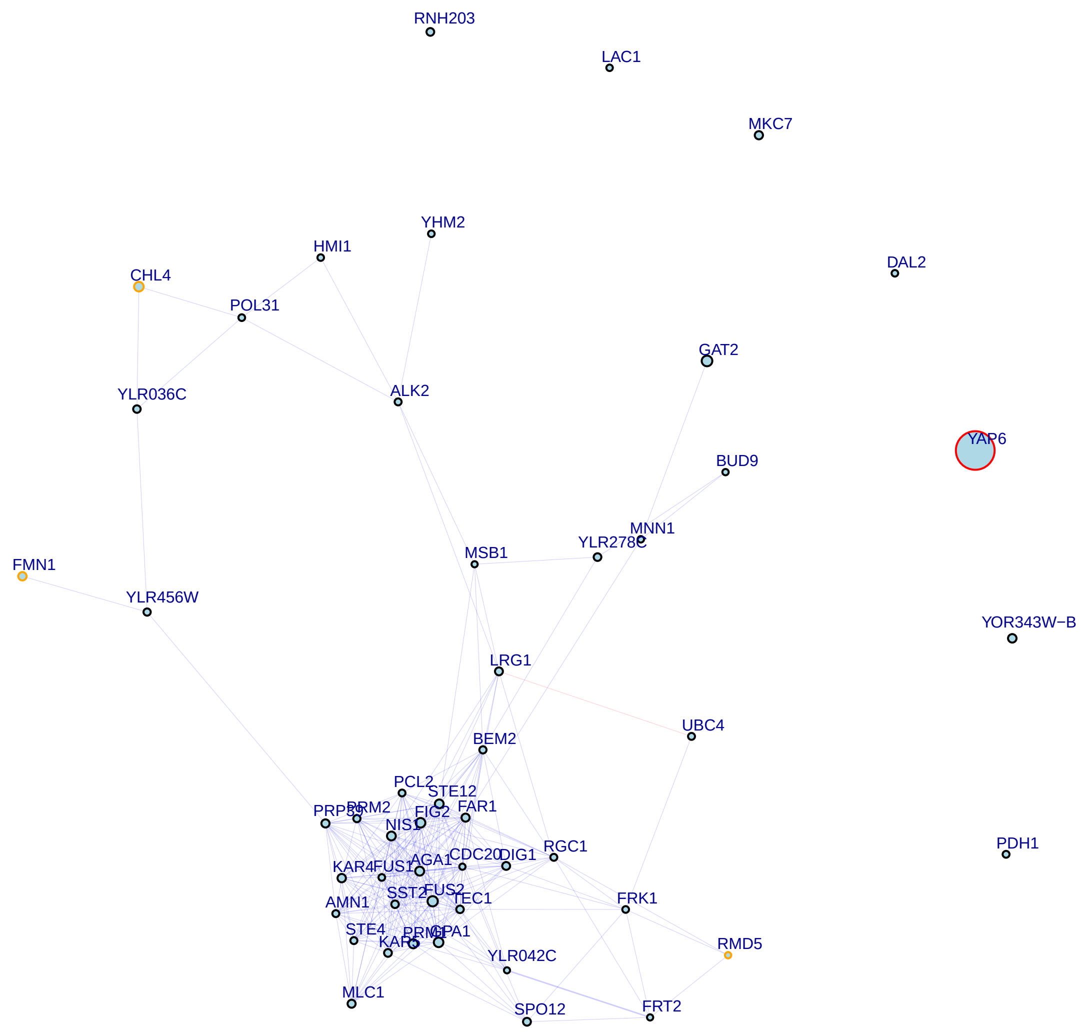

chrIV:975156_A/G top 50 down genes

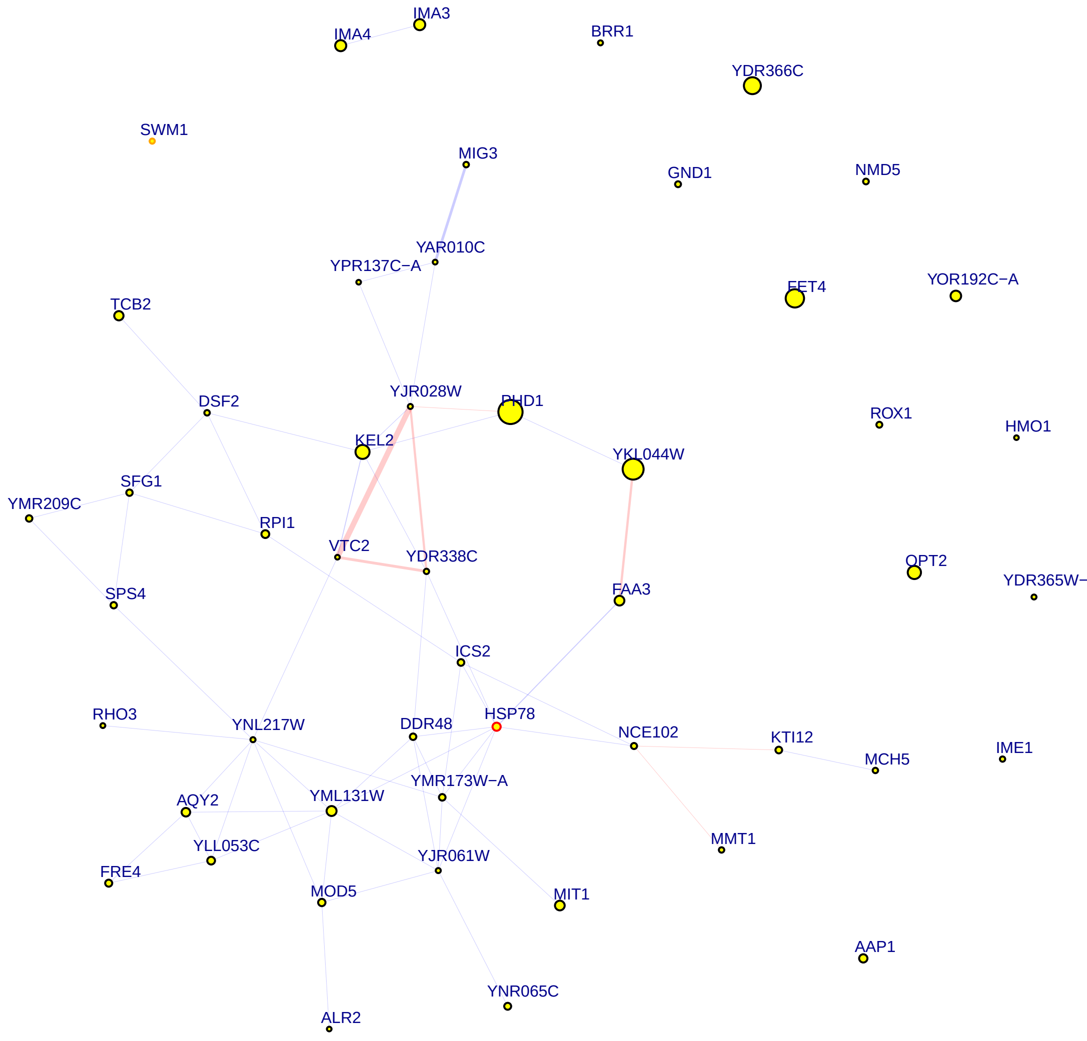

chrIV:975156_A/G top 100 up and down genes

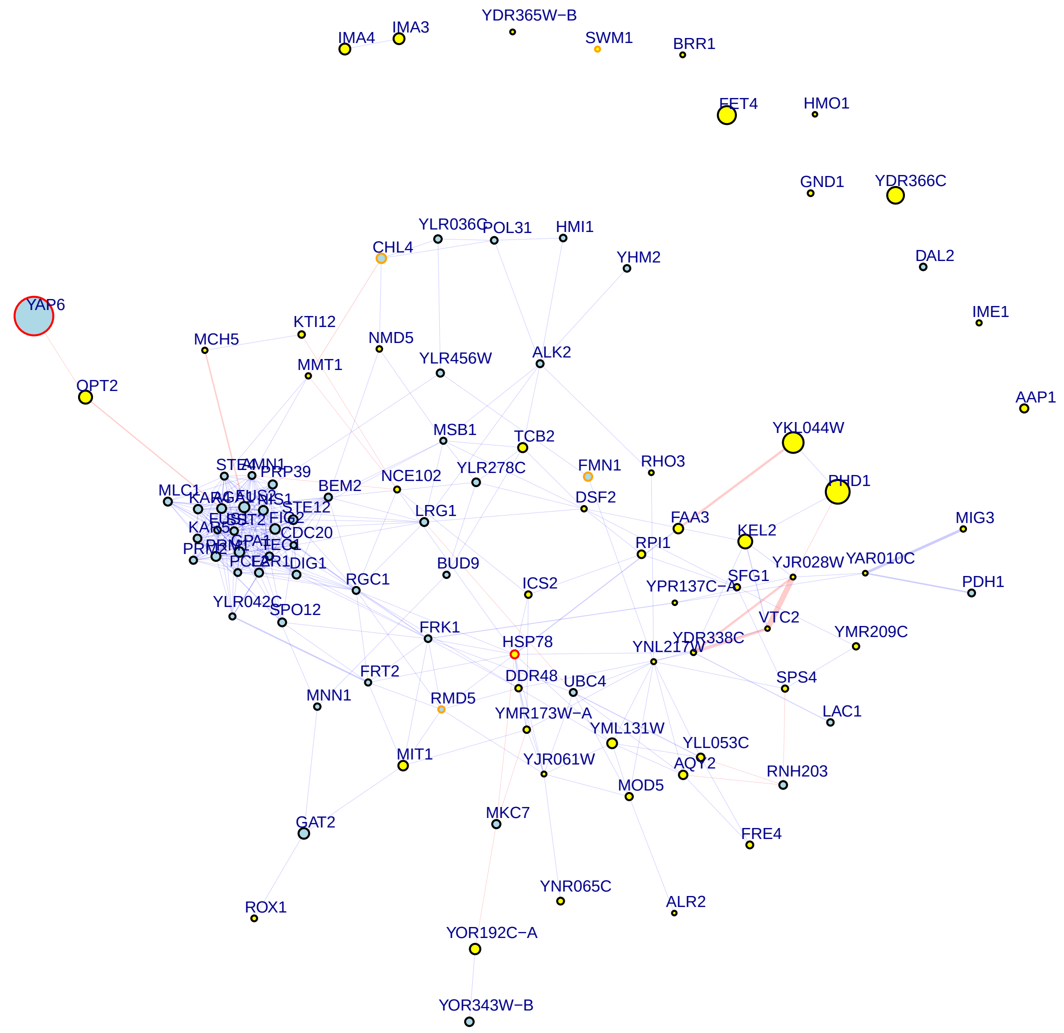

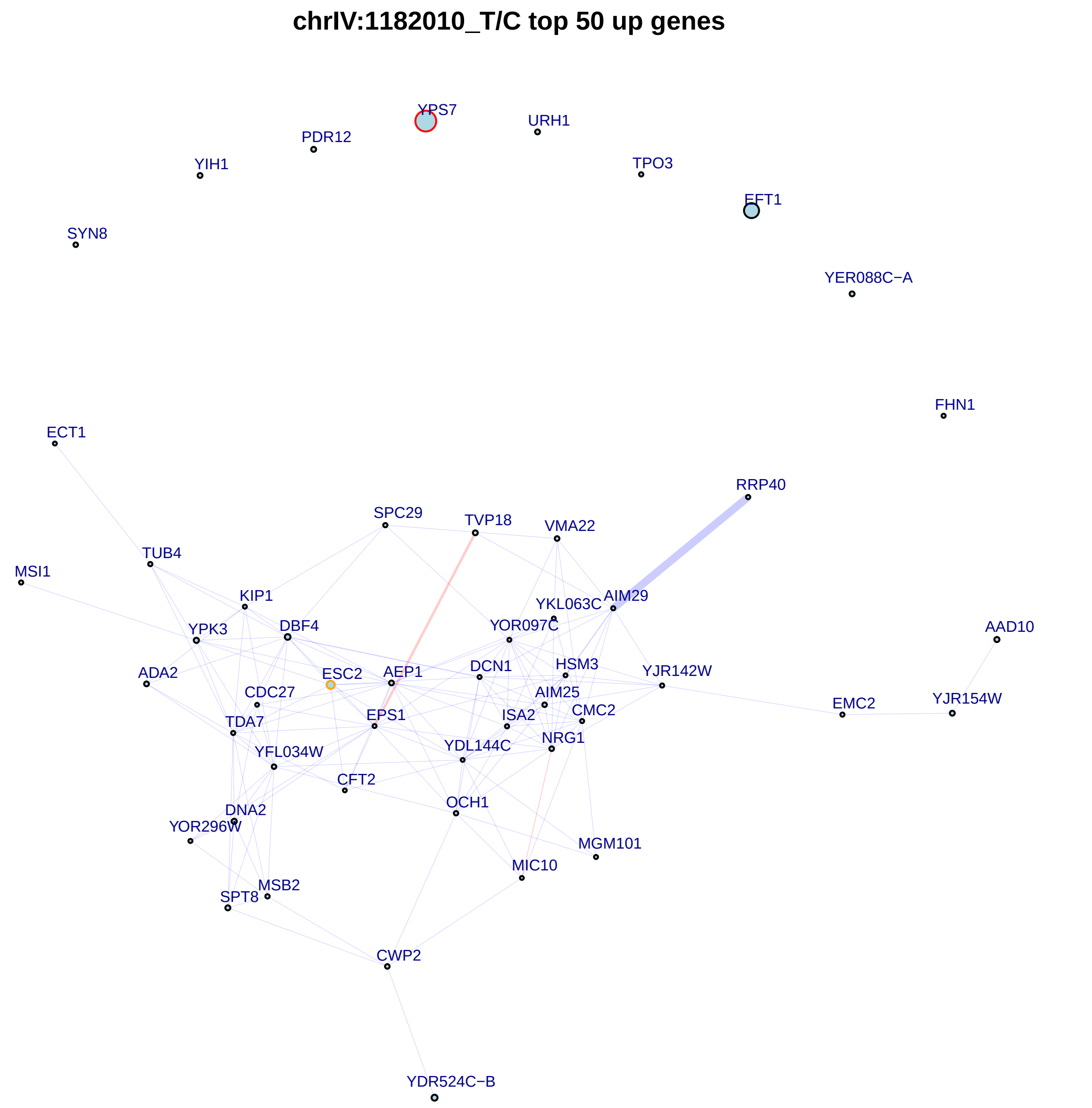
chrIV:1182010_T/C top 50 up genes
VPS7
URH1
PDR12
TPO3
YIH1
EFT1
SYN8
YER088C−A
FHN1
ECT1
RRP40
SPC29
TVP18
VMA22
TUB4
MSI1
KIP1
YKL063C
AIM29
YOR097C
AAD10
YPK3
DBF4
ADA2
ESC2
AEP1
DCN1
HSM3
YJR142W
CDC27
AIM25
EMC2
YJR154W
EPS1
ISA2
CMC2
TDA7
YDL144C
NRG1
YFL034W
CFT2
DNA2
OCH1
YOR296W
MGM101
MSB2
SPT8
MIC10
CWP2
YDR524C−B
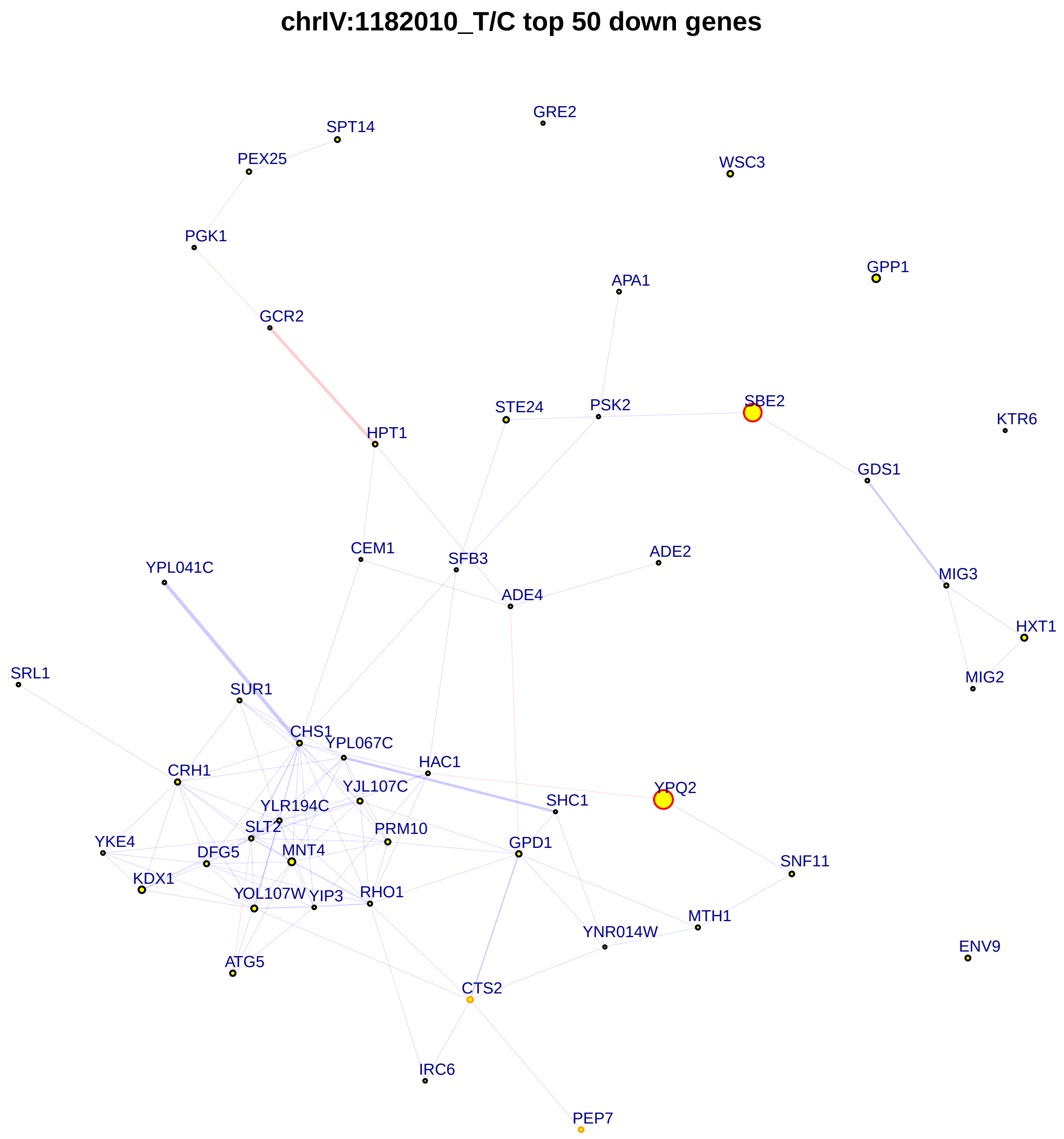
chrIV:1182010_T/C top 50 down genes
SPT14
GRE2
PEX25
WSC3
PGK1
GPP1
APA1
GCR2
STE24
PSK2
SBE2
KTR6
HPT1
GDS1
CEM1
SFB3
ADE2
YPL041C
ADE4
MIG3
HXT1
SRL1
MIG2
SUR1
CHS1
YPL067C
CRH1
HAC1
YJL107C
YPQ2
SHC1
YLR194C
SLT2
PRM10
YKE4
DFG5
MNT4
GPD1
SNF11
KDX1
YOL107W
YIP3
RHO1
MTH1
YNR014W
ENV9
ATG5
CTS2
IRC6
PEP7
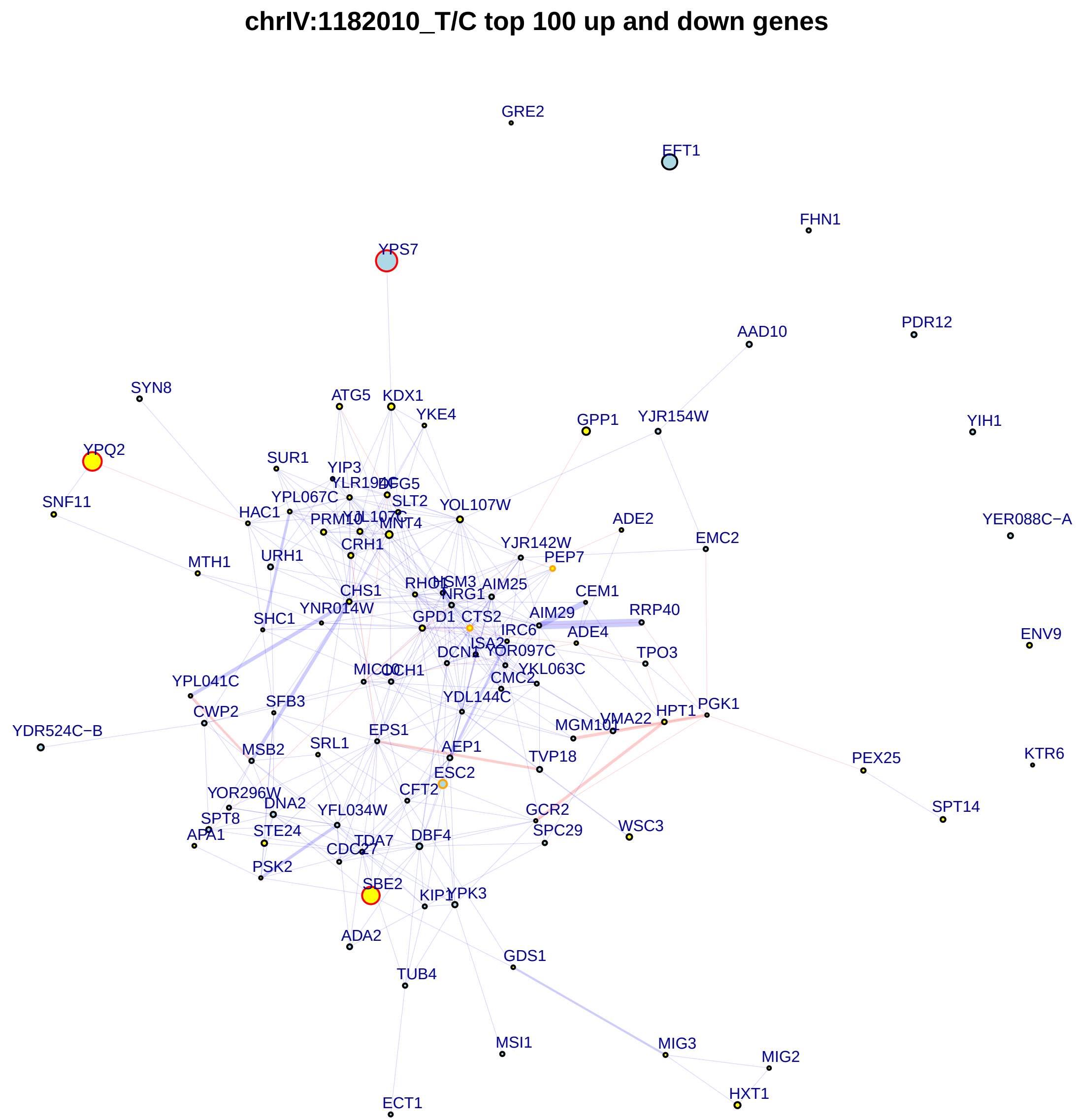
chrIV:1182010_T/C top 100 up and down genes
GRE2
EFT1
FHN1
VPS7
AAD10
PDR12
SYN8
ATG5
KDX1
YKE4
GPP1
YJR154W
YIH1
YPQ2
SUR1
YIP3
DFG5
SNF11
YPL067C
SLT2
YOL107W
YER088C−A
HAC1
MNT4
ADE2
EMC2
MTH1
URH1
CRH1
YJR142W
PEP7
RHO1
HSM3
AIM25
CEM1
CHS1
NRG1
YNR014W
AIM29
RRP40
SHC1
GPD1
CTS2
IRC6
ADE4
ENV9
DCN1
YOR097C
TPO3
YPL041C
MIC10
OCH1
YKL063C
CMC2
YDL144C
SFB3
CWP2
MGM101
VMA22
HPT1
PGK1
YDR524C−B
EPS1
AEP1
SRL1
MSB2
TVP18
PEX25
KTR6
ESC2
YOR296W
CFT2
DNA2
SPT14
SPT8
YFL034W
GCR2
APA1
STE24
SPC29
WSC3
TDA7
DBF4
PSK2
SBE2
KIP1
YPK3
ADA2
GDS1
TUB4
MSI1
MIG3
MIG2
ECT1
HXT1

chrIV:1357789_T/G top 50 up genes

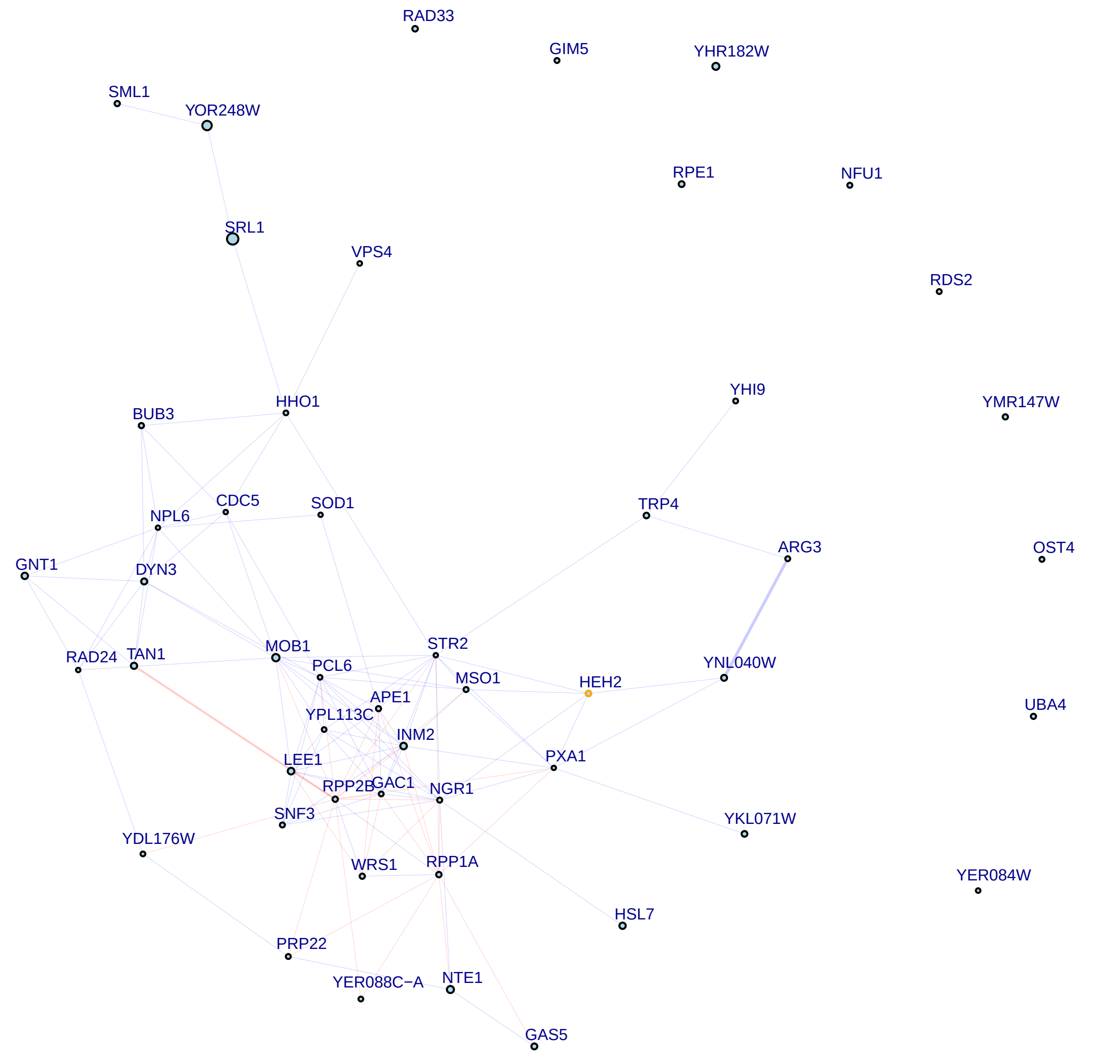

chrIV:1357789_T/G top 50 down genes

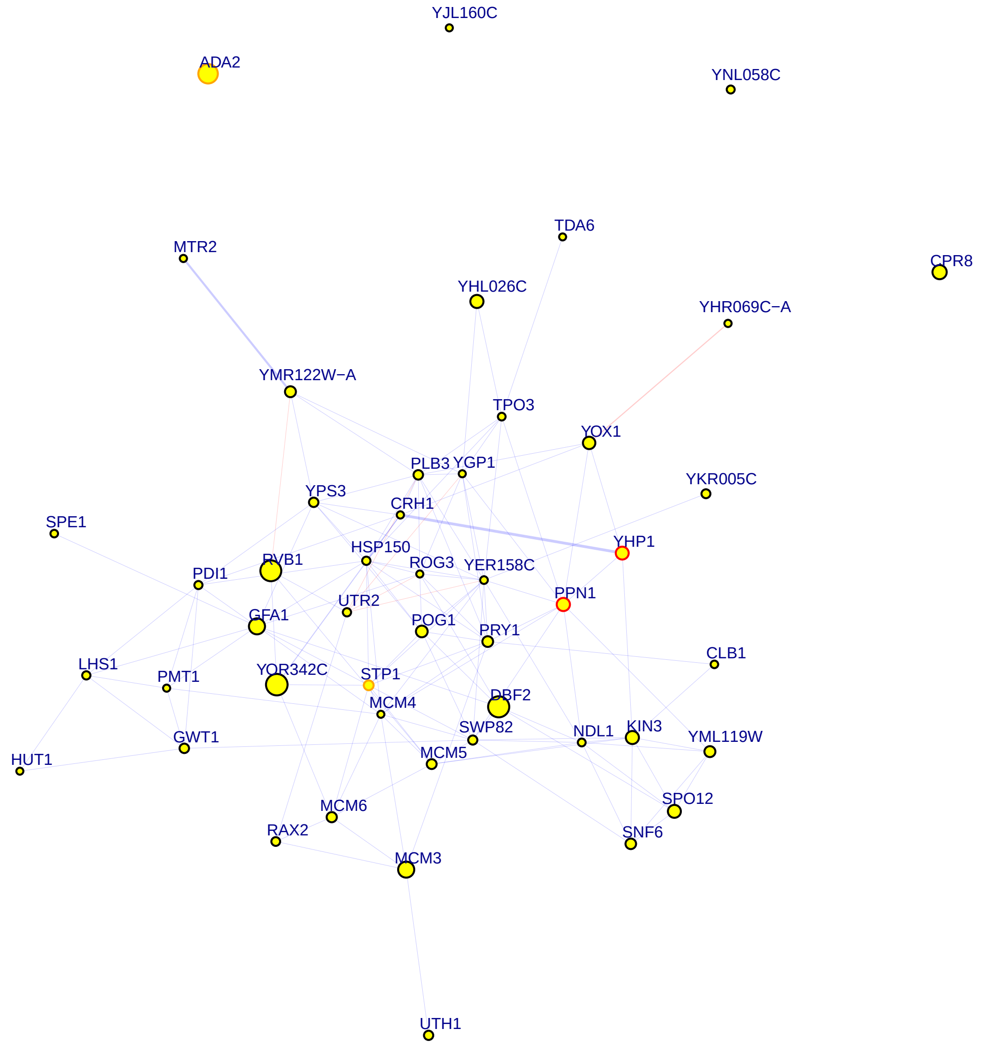

chrIV:1357789_T/G top 100 up and down genes

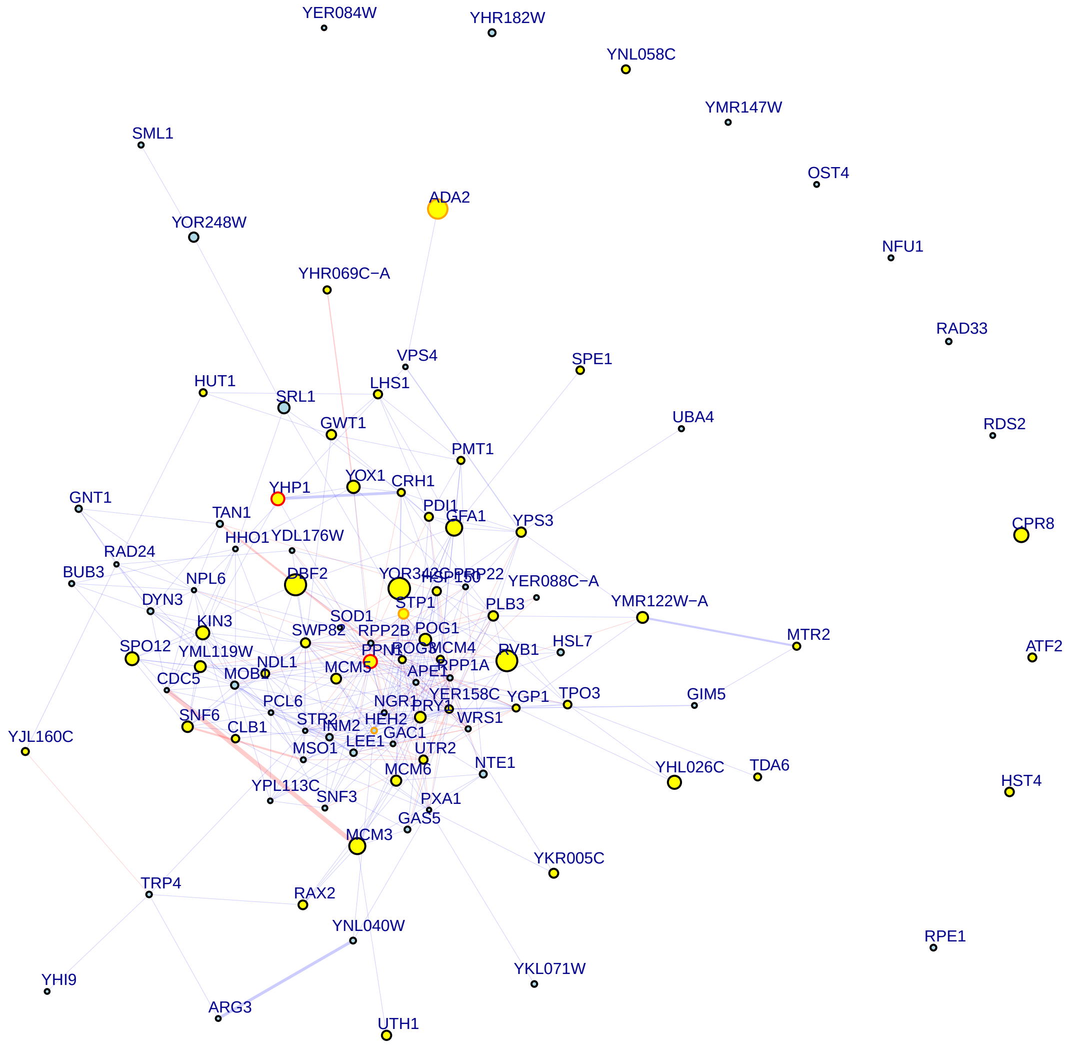

chrIV:1521369_A/C top 50 up genes

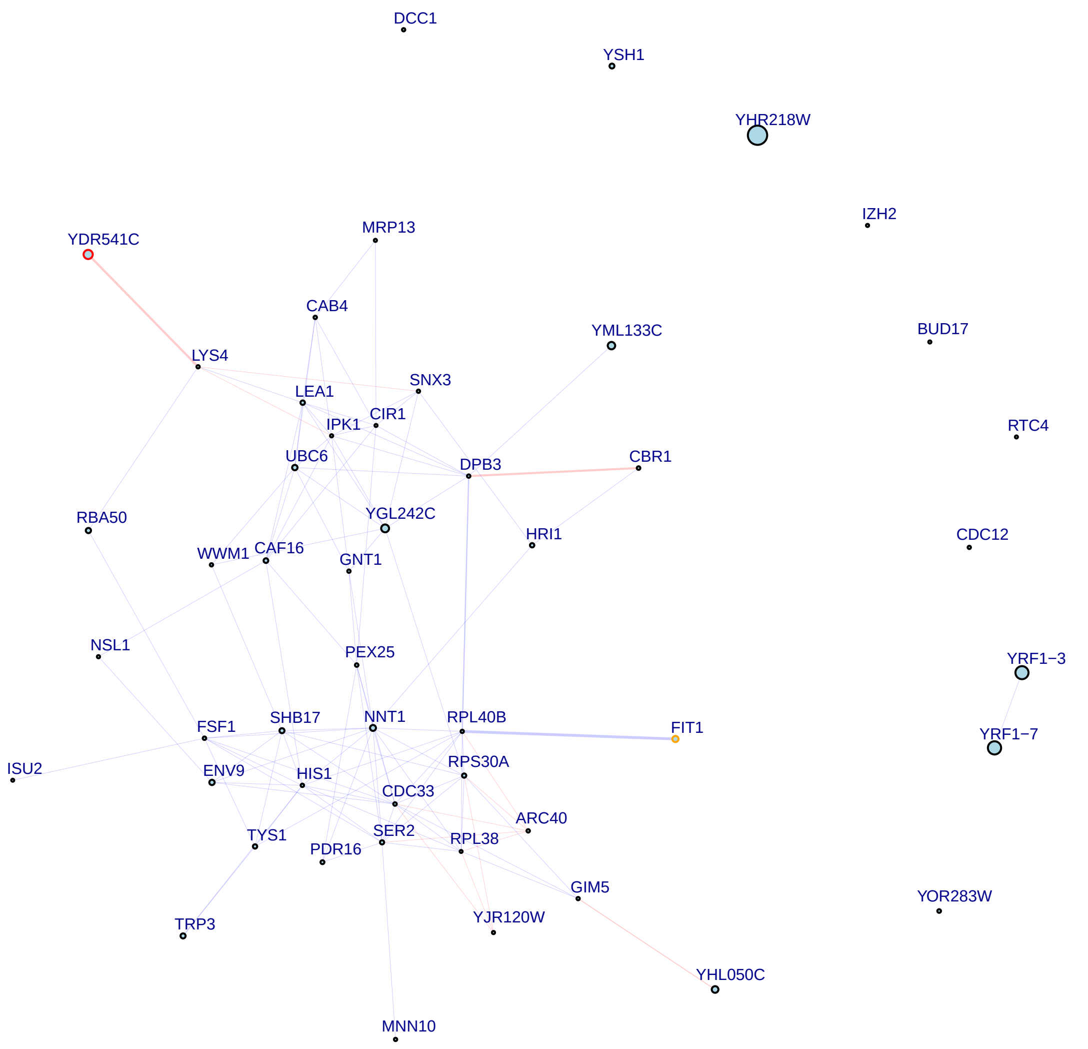

chrIV:1521369_A/C top 50 down genes

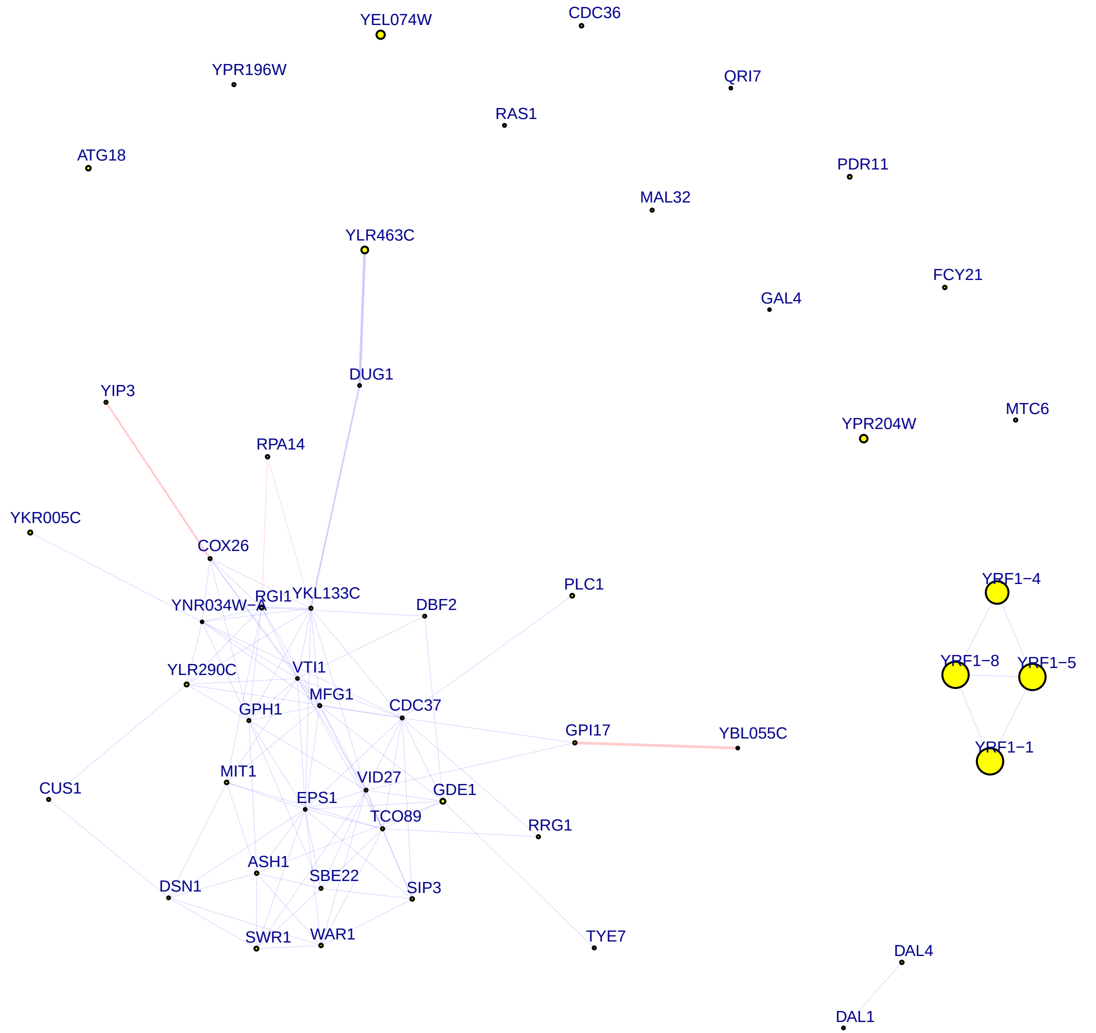

chrIV:1521369_A/C top 100 up and down genes

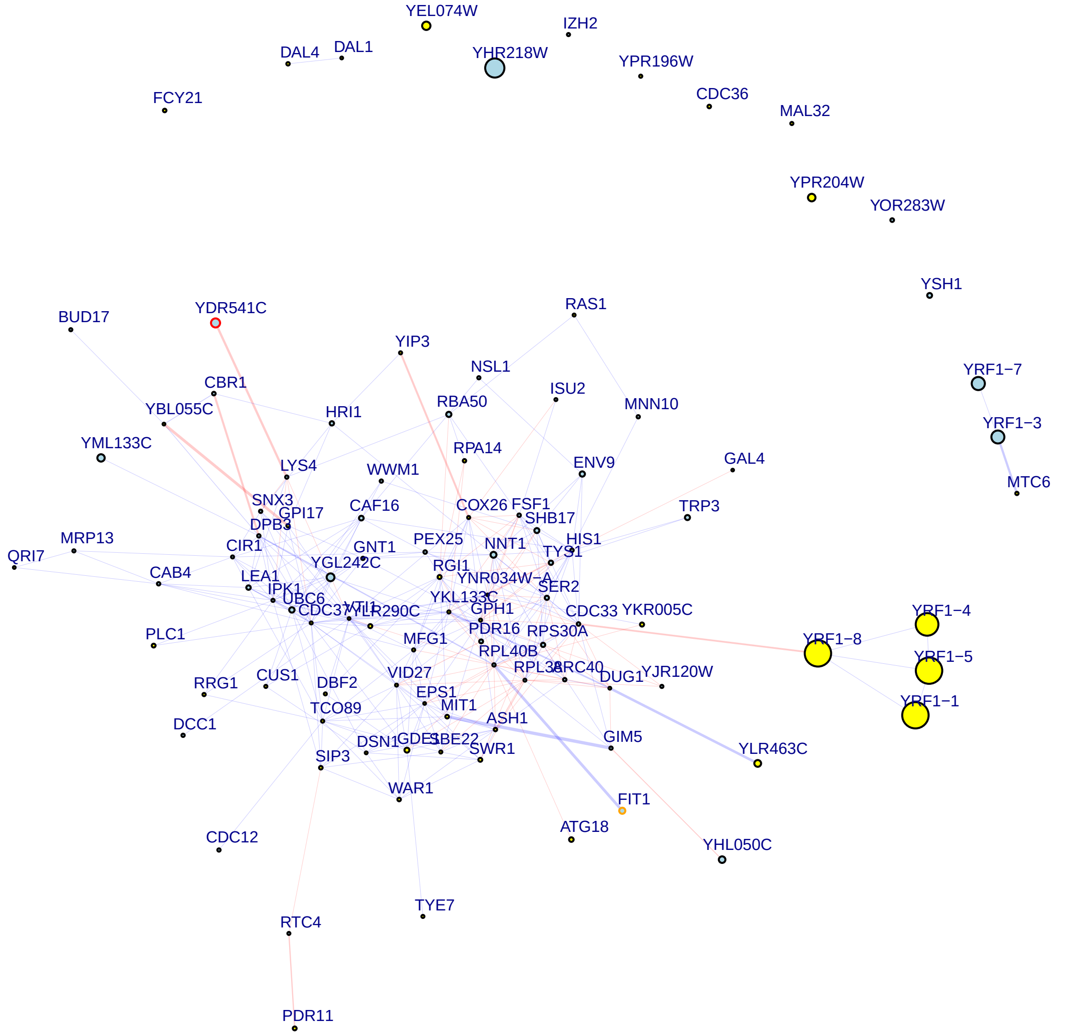

chrV:11186_T/C top 46 up genes

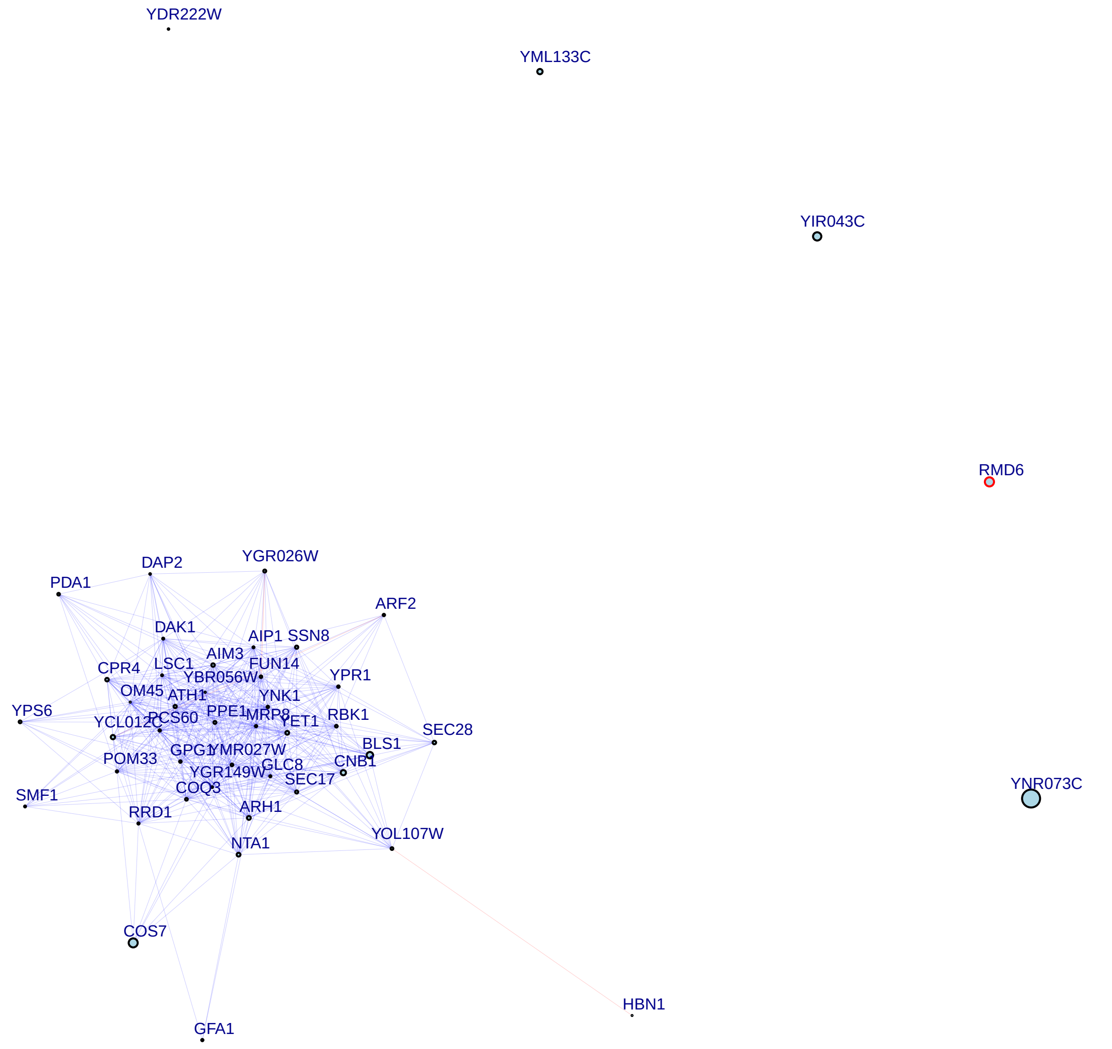

chrV:11186_T/C top 24 down genes

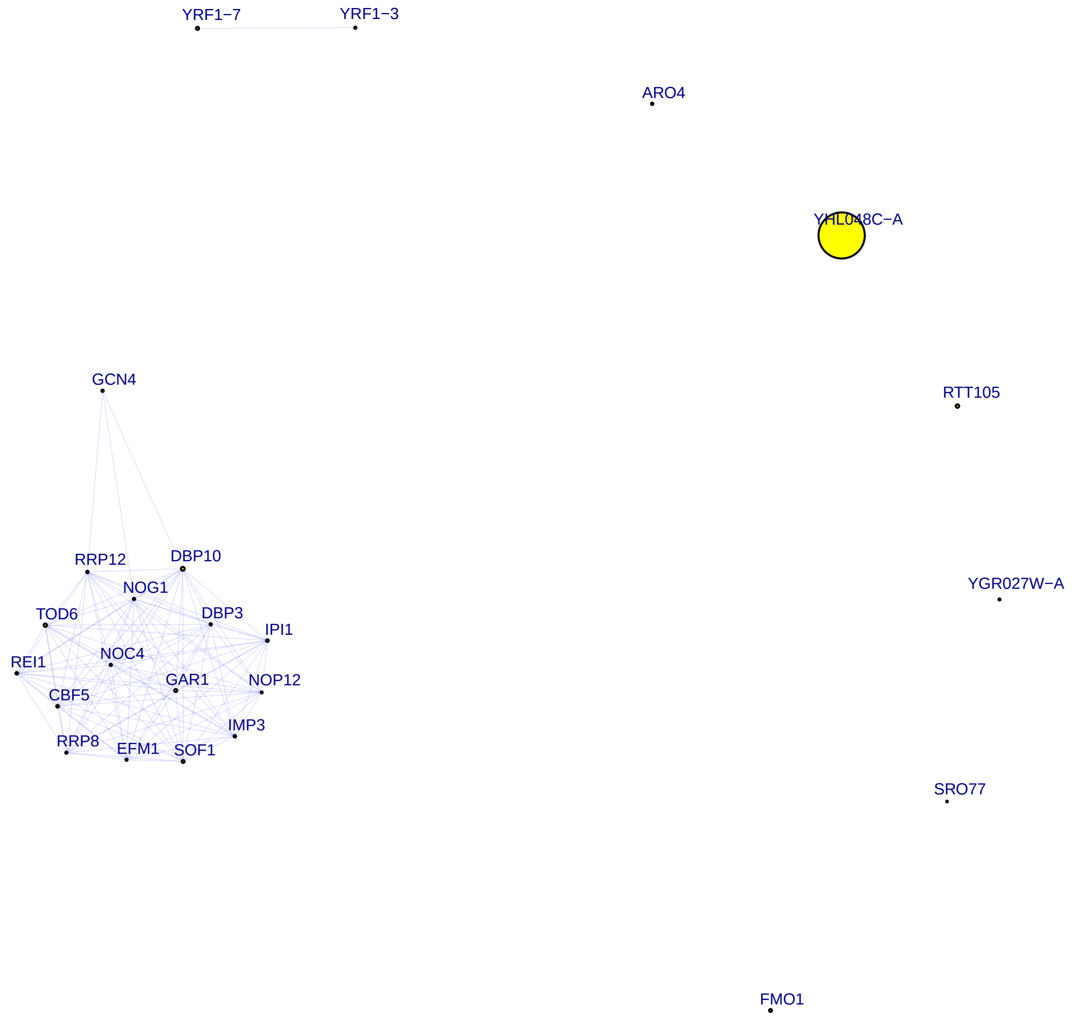

chrV:11186_T/C top 70 up and down genes

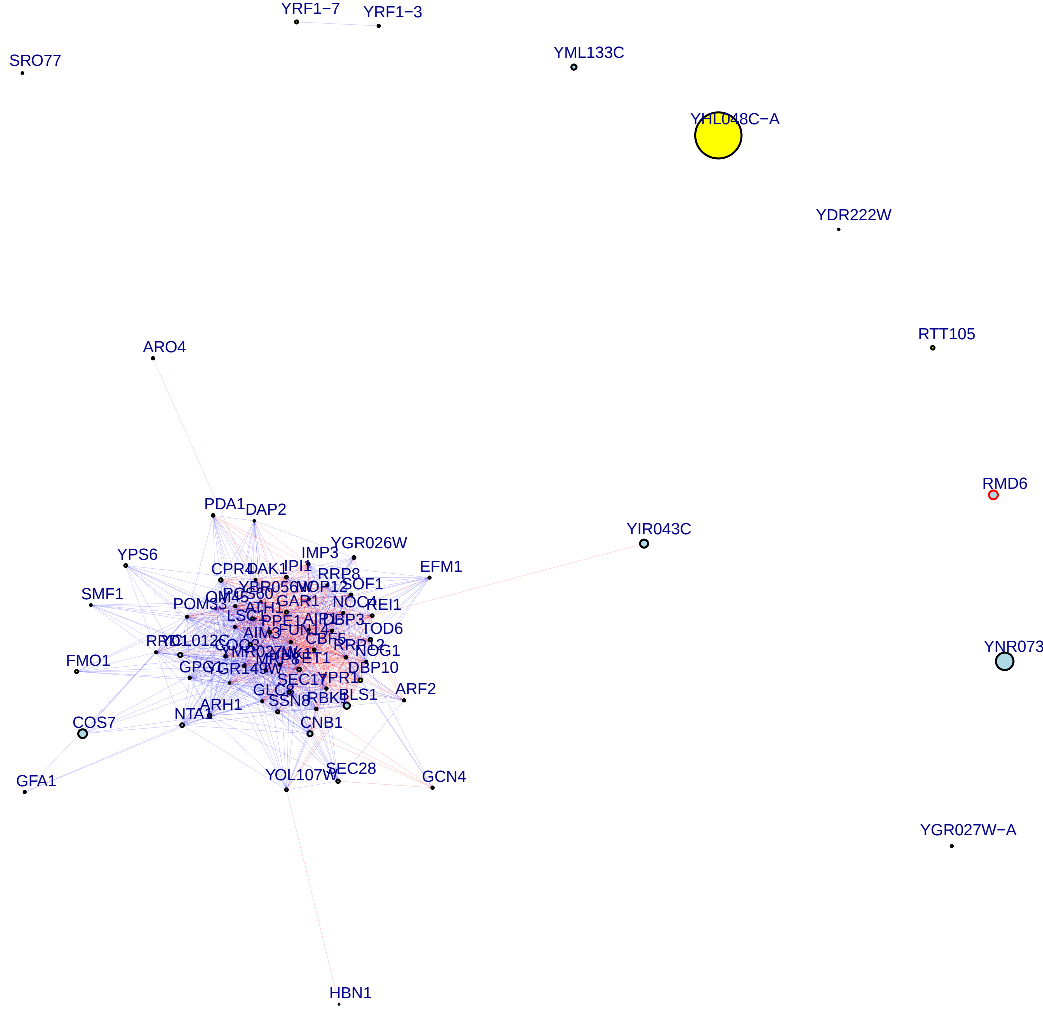

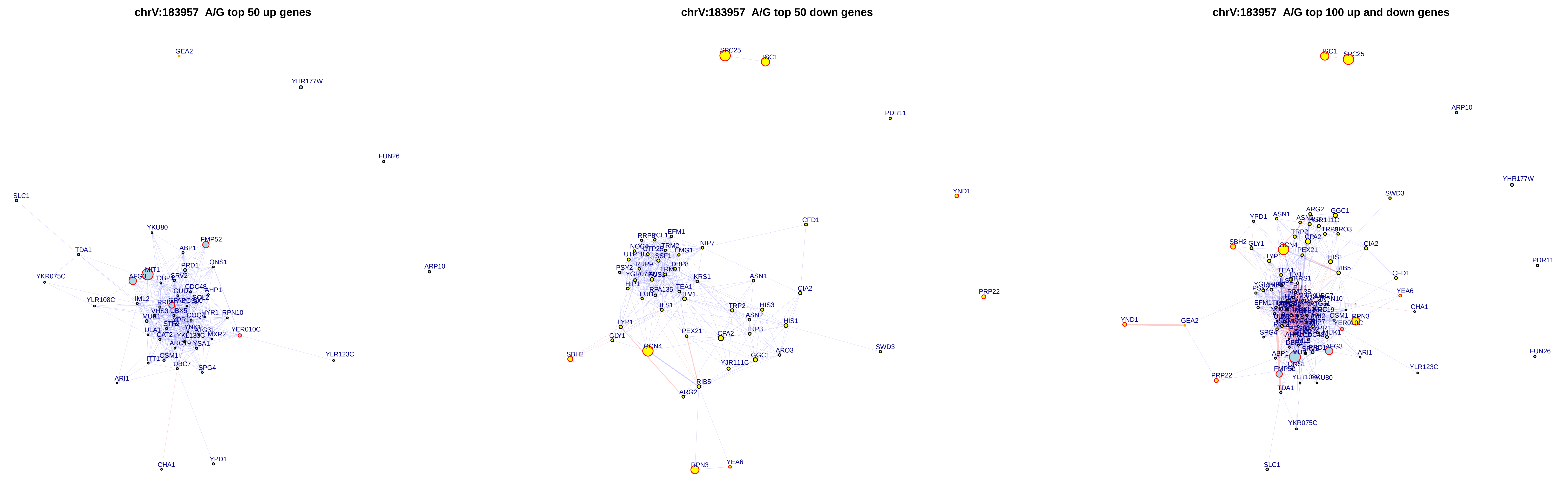
chrV:183957_A/G top 50 up genes
GEA2
YHR177W
FUN26
SLC1
ARP10
YKU80
FMP52
TDA1
ABP1
ONS1
YKR075C
MIT1
AFG3
PRD1
SRV2
CDC48
AHP1
YLR108C
IML2
RPN10
YER010C
ULA1
CAT2
YSA1
MXR2
ITT1
OSM1
UBC7
SPG4
YLR123C
ARI1
CHA1
YPD1
chrV:183957_A/G top 50 down genes
SPC25
ISC1
PDR11
YND1
CFD1
EFM1
NIP7
CIA2
KRS1
ASN1
TEA1
ILV1
ILS1
TRP2
HIS3
PRP22
LYP1
ASN2
HIS1
SWD3
GLY1
PEX21
CPA2
TRP3
SBH2
GCN4
ARO3
YJR111C
GGC1
RIB5
ARG2
RPN3
YEA6
chrV:183957_A/G top 100 up and down genes
ISC1
SPC25
ARP10
YHR177W
SWD3
PDR11
ARG2
GGC1
YPD1
ASN1
SBH2
GLY1
GCN4
CPA2
CIA2
PEX21
HIS1
RIB5
CFD1
YEA6
CHA1
ITT1
RPN3
YND1
GEA2
SPG4
OSM1
ARI1
AFG3
ABP1
ONS1
FMP52
YLR108C
YKU80
TDA1
PRP22
YLR123C
FUN26
YKR075C
SLC1

chrV:418504_A/G top 36 up genes

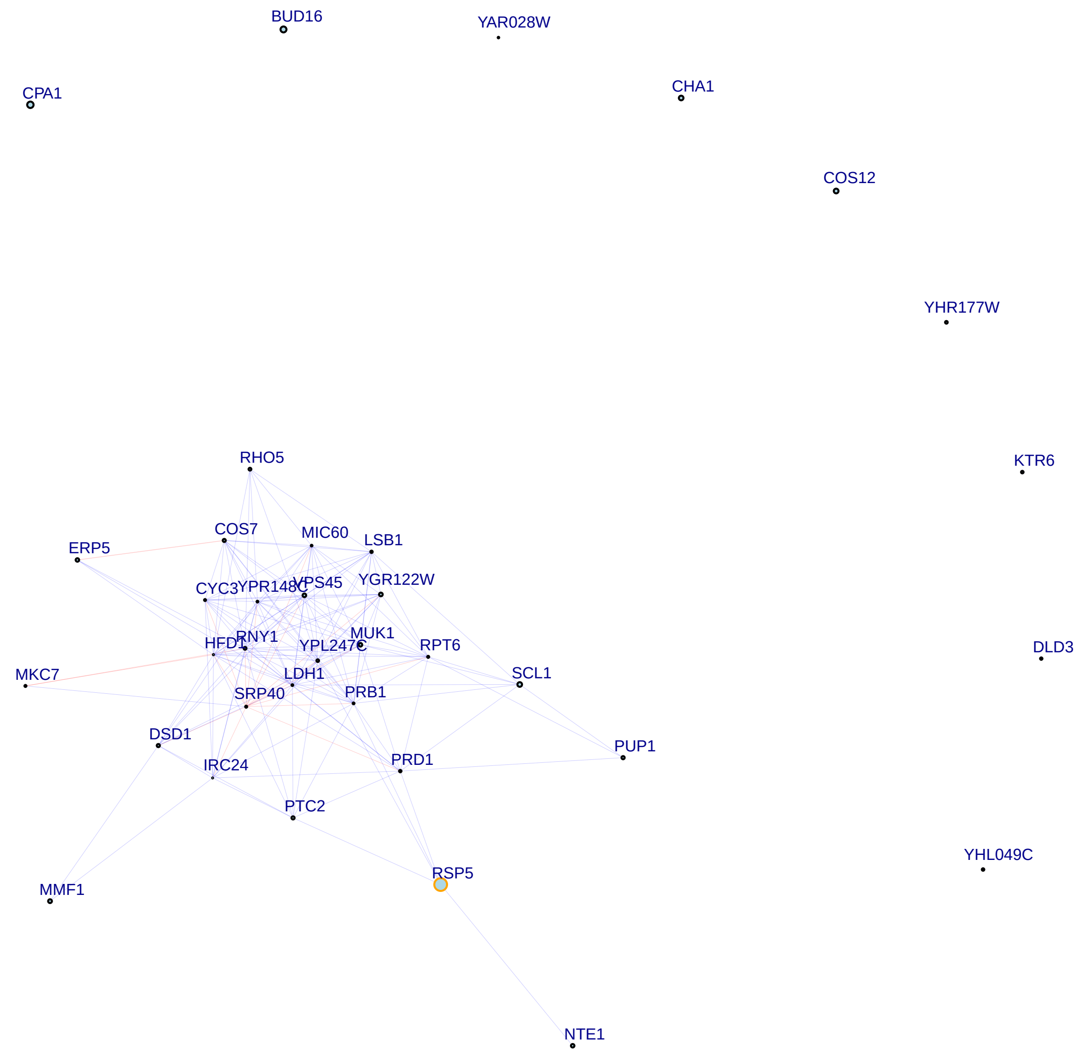

chrV:418504_A/G top 50 down genes

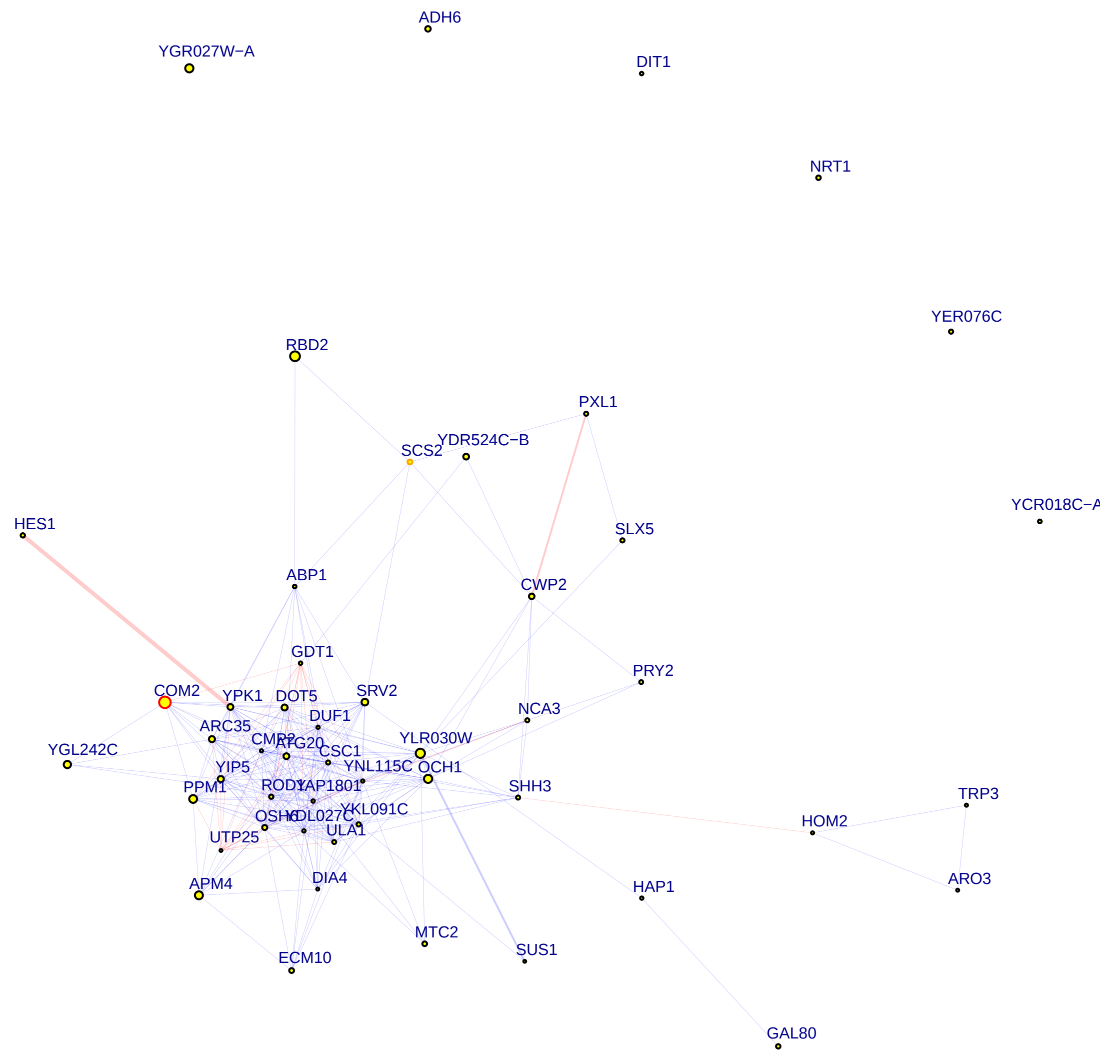

chrV:418504_A/G top 86 up and down genes

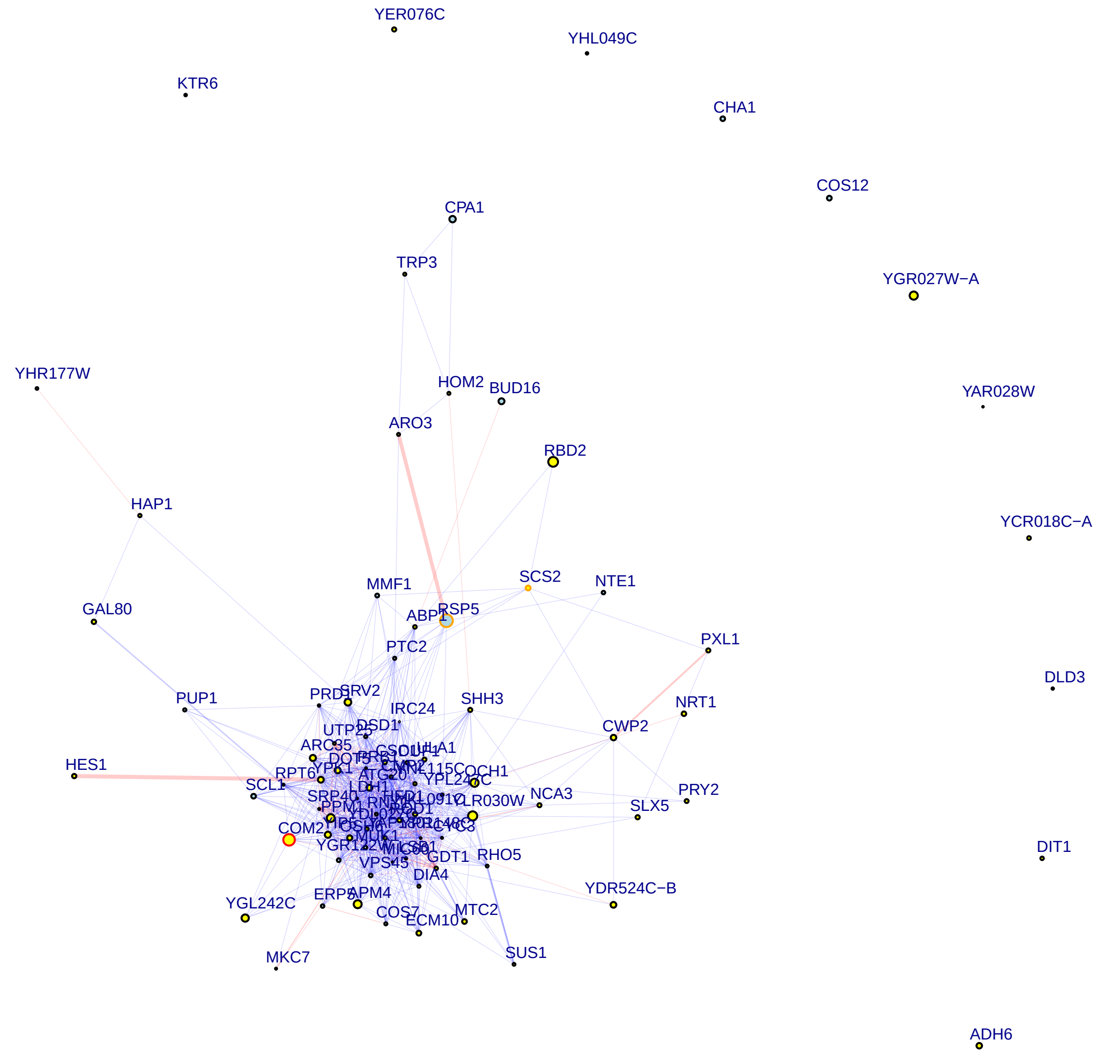

chrV:524625_A/T top 50 up genes

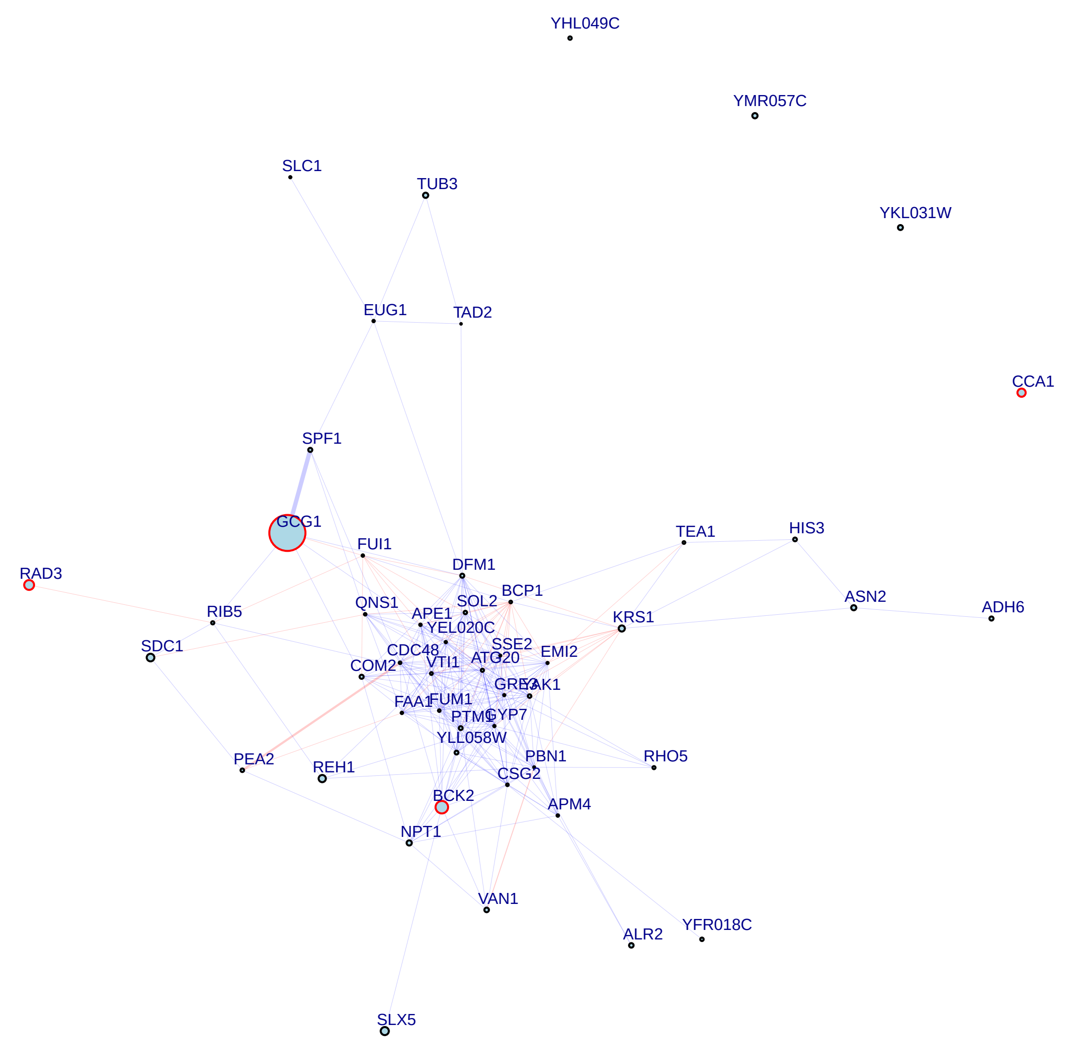

chrV:524625_A/T top 50 down genes

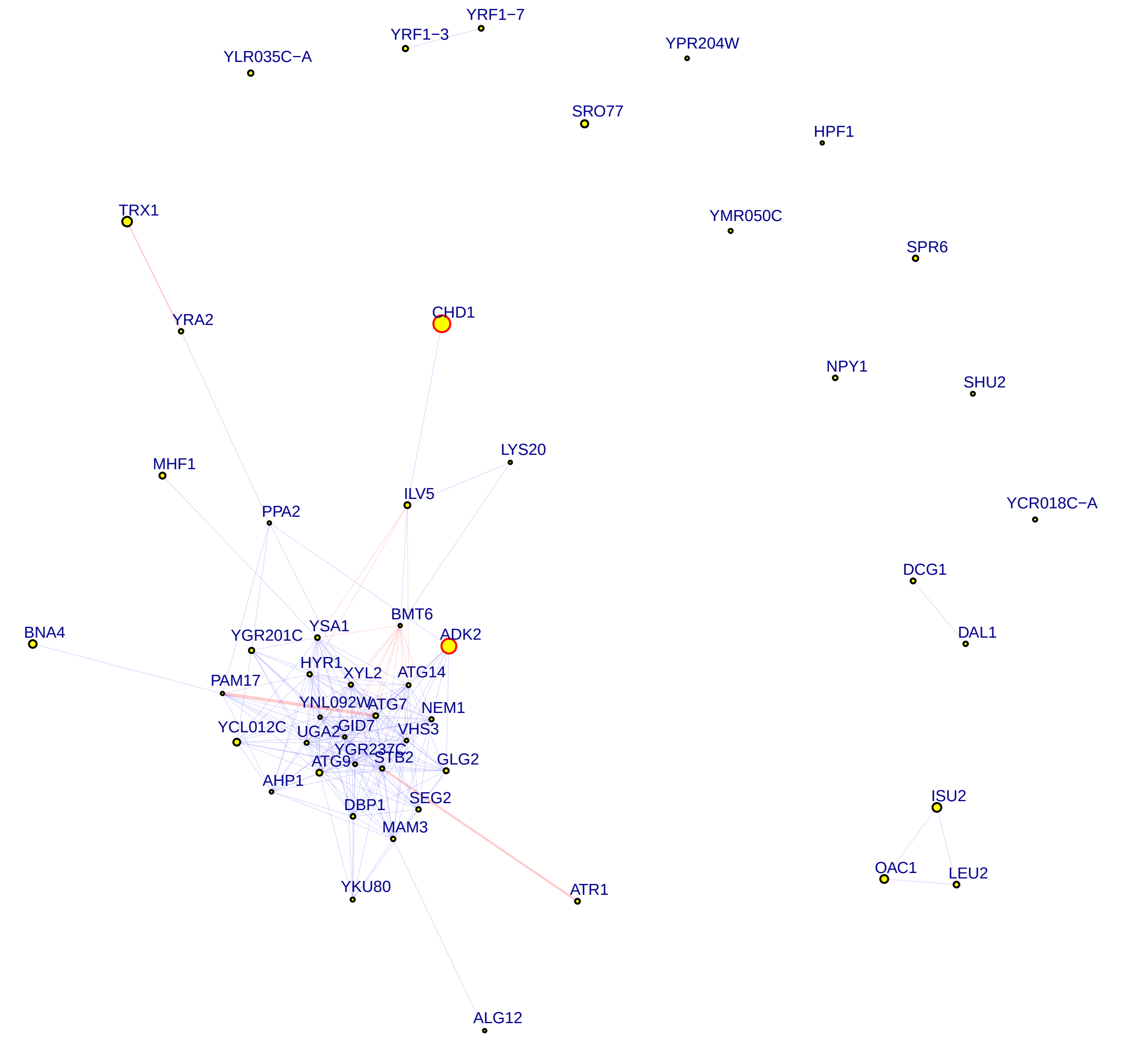

chrV:524625_A/T top 100 up and down genes

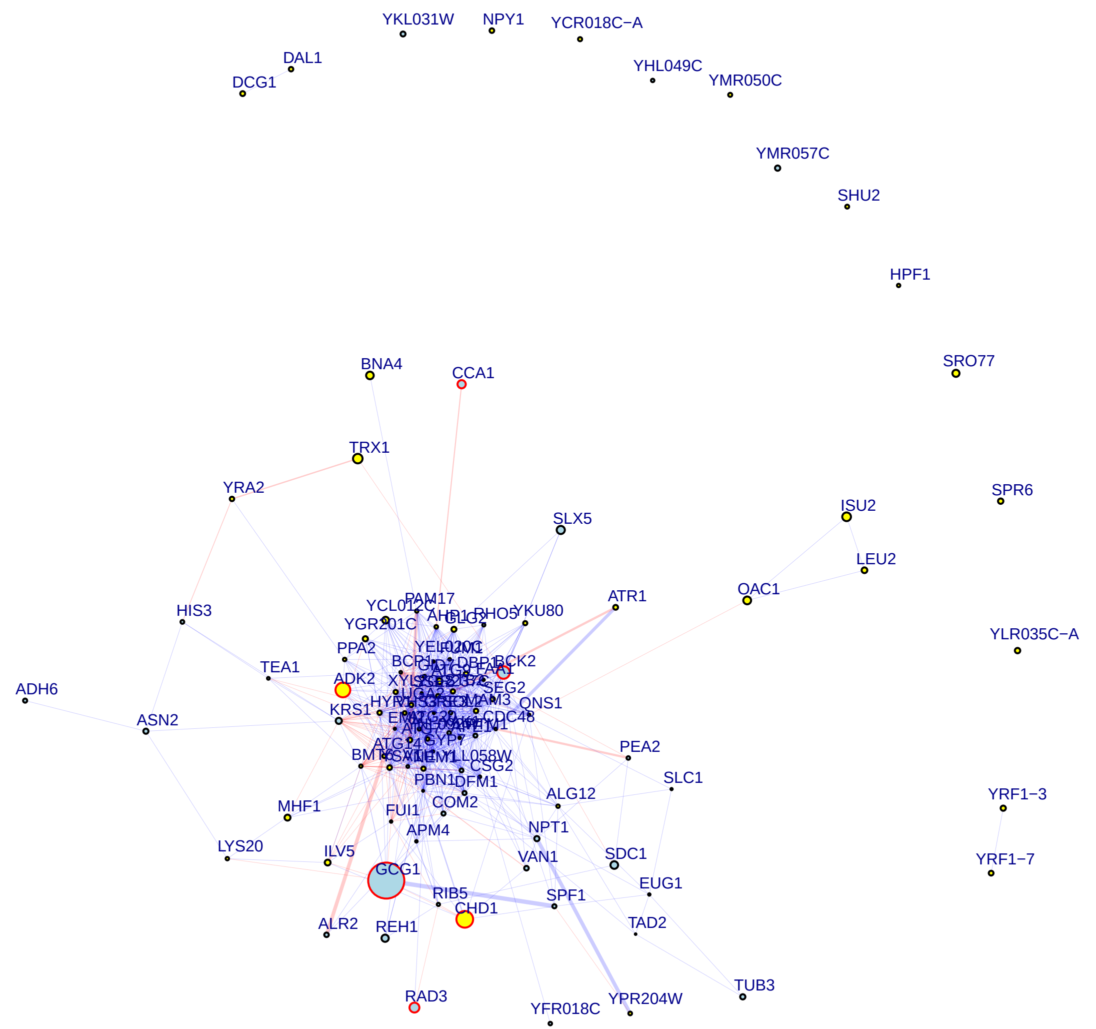

chrVI:71959_C/T top 50 up genes

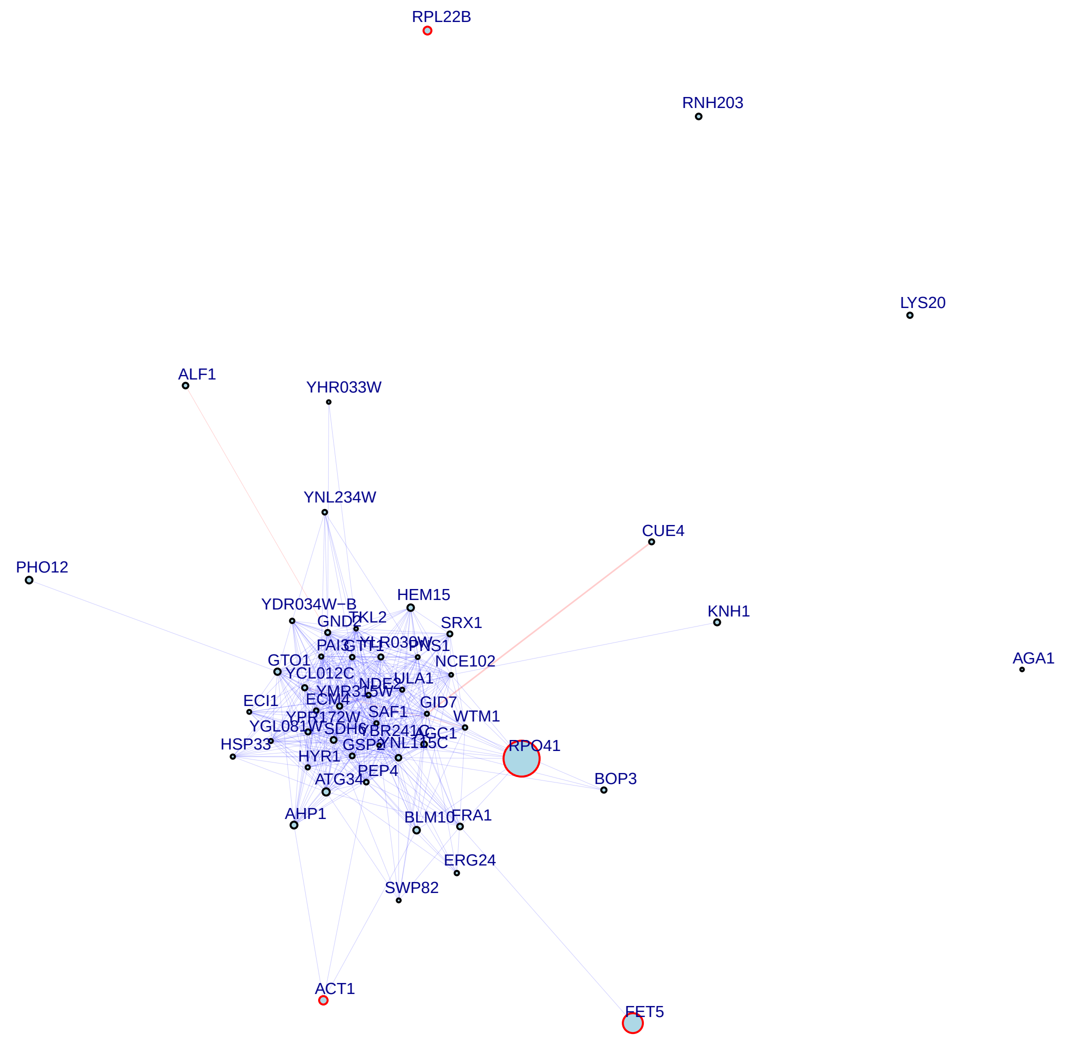

chrVI:71959_C/T top 50 down genes

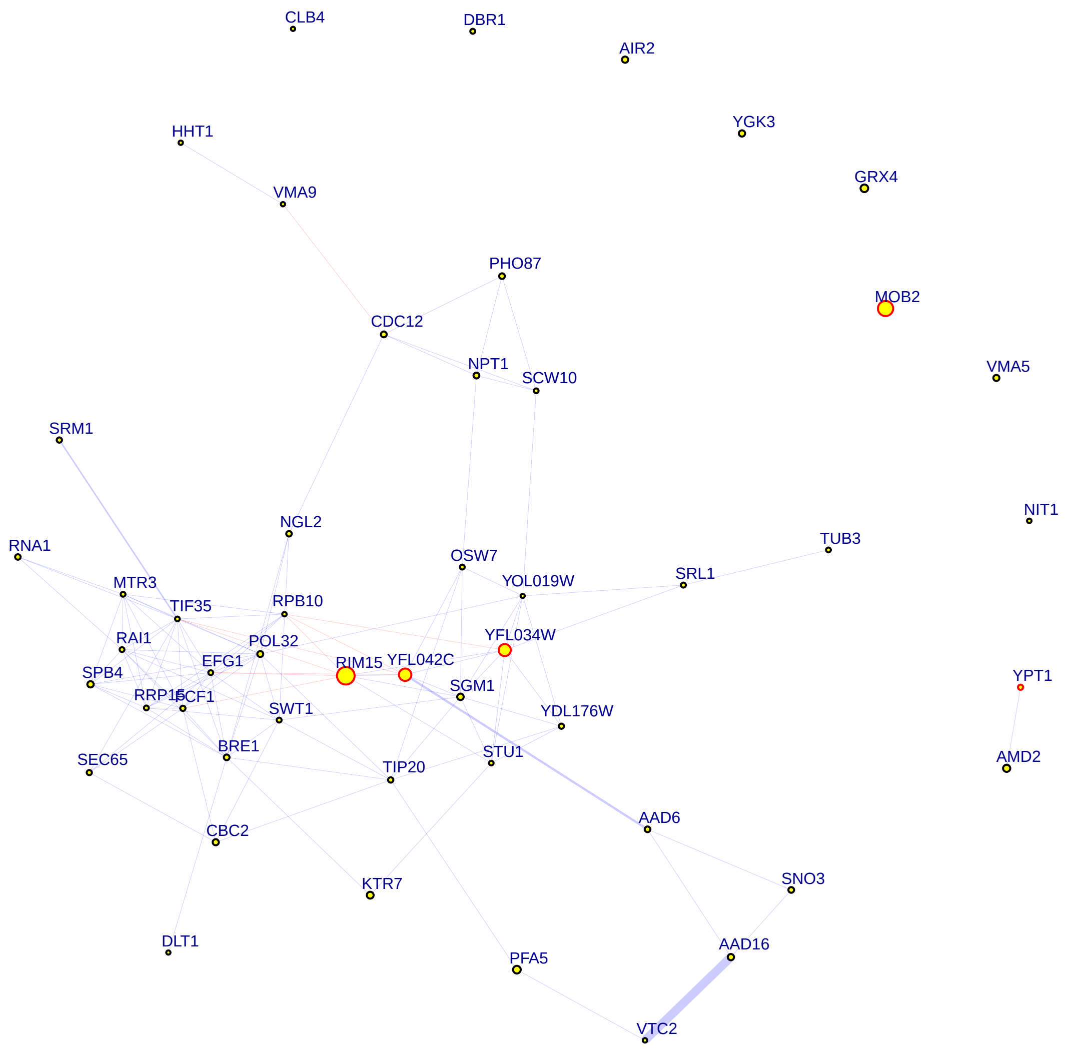

chrVI:71959_C/T top 100 up and down genes

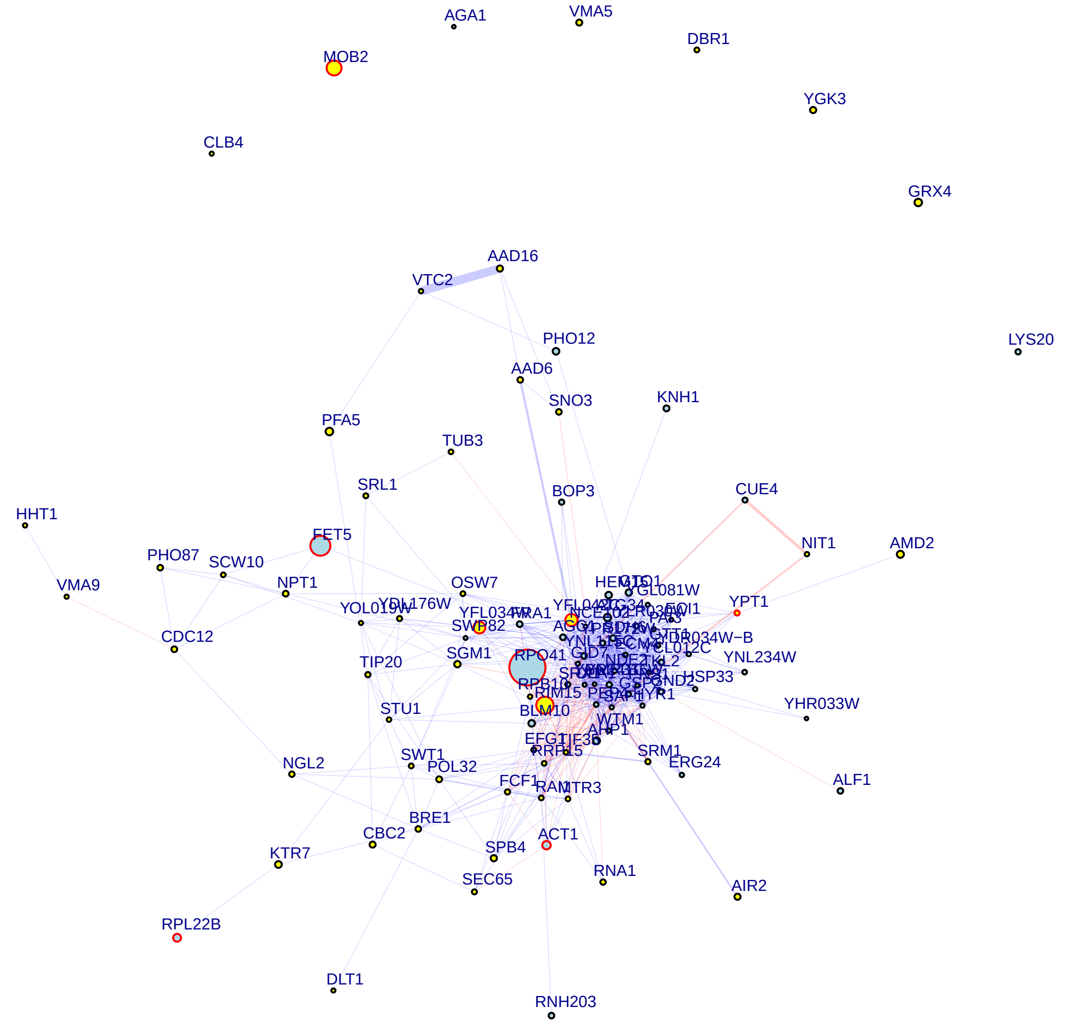

chrVI:95849_T/C top 49 up genes

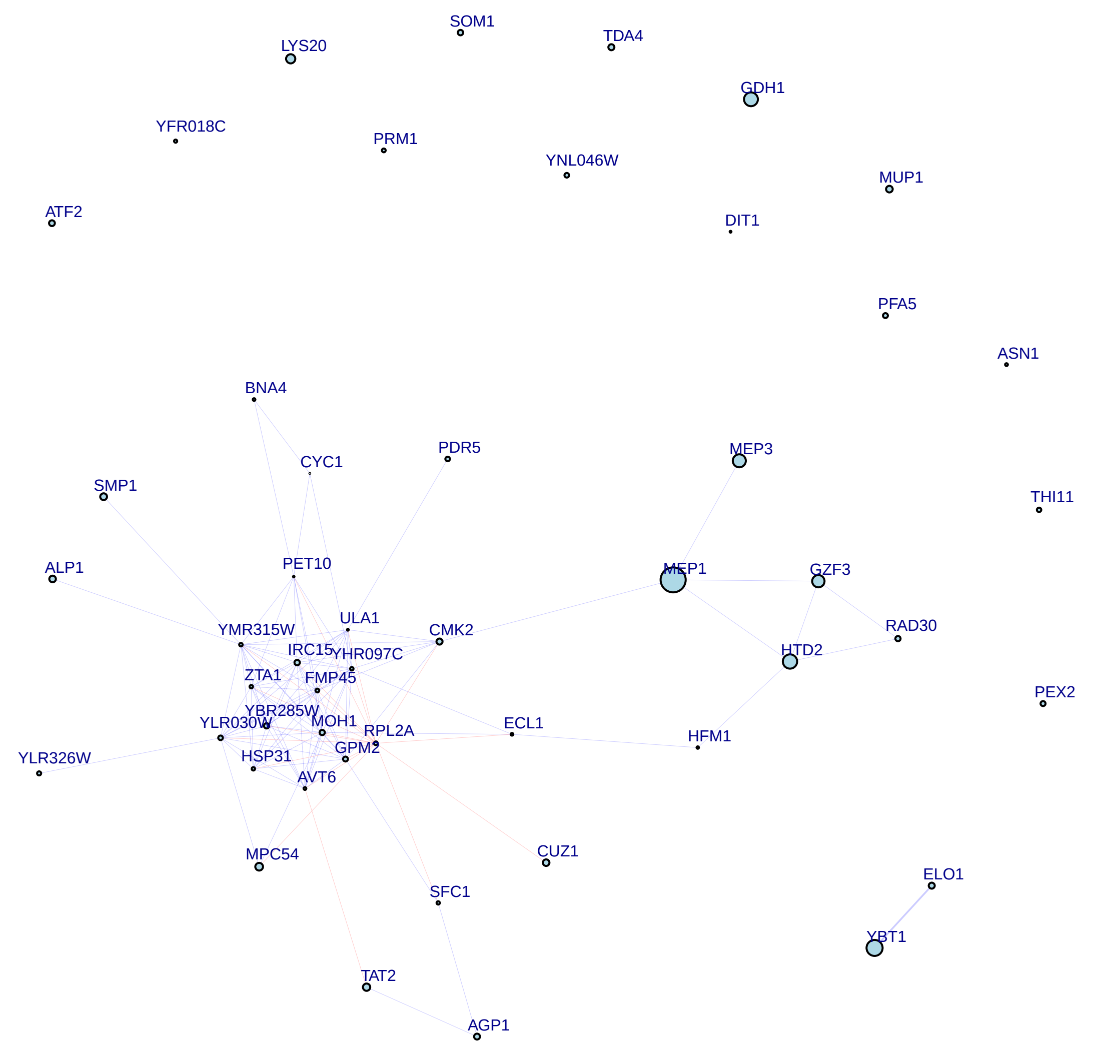

chrVI:95849_T/C top 50 down genes

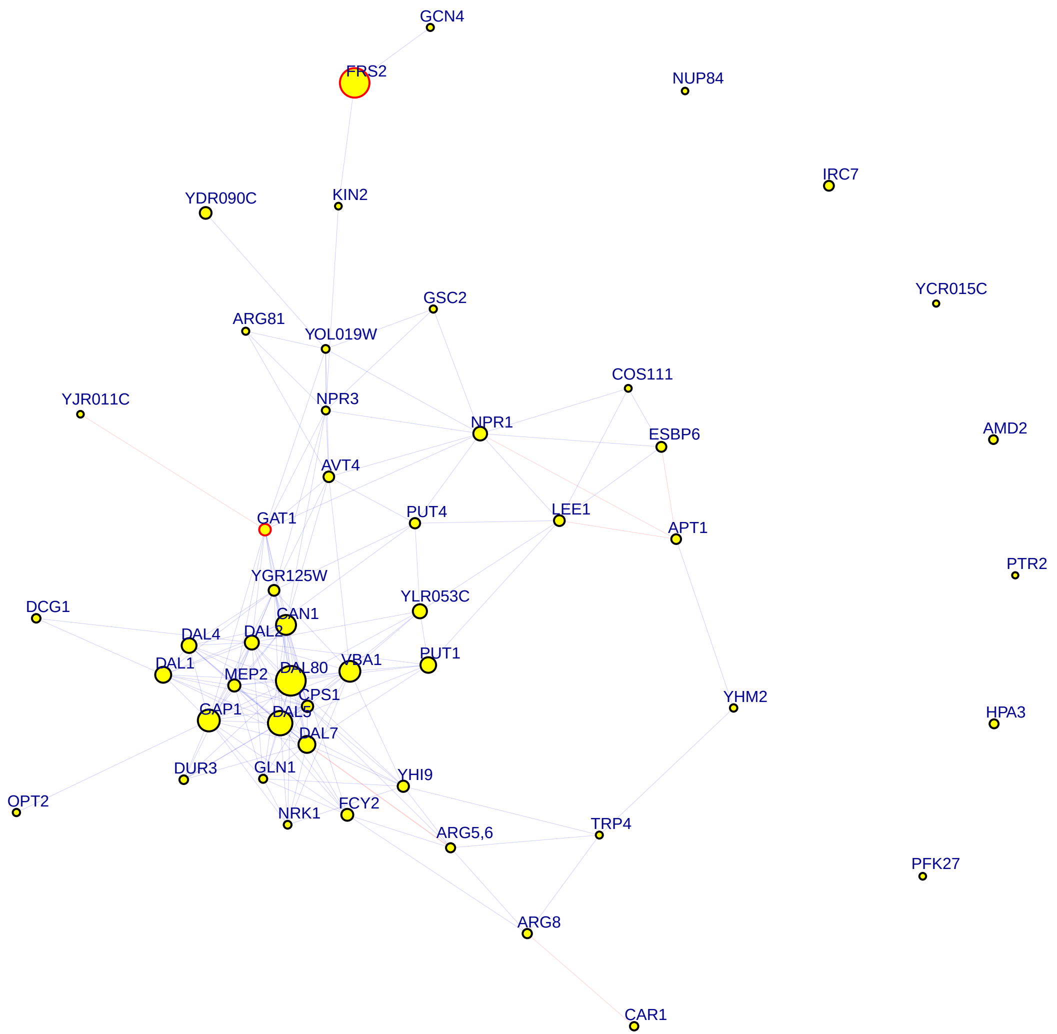

chrVI:95849_T/C top 99 up and down genes

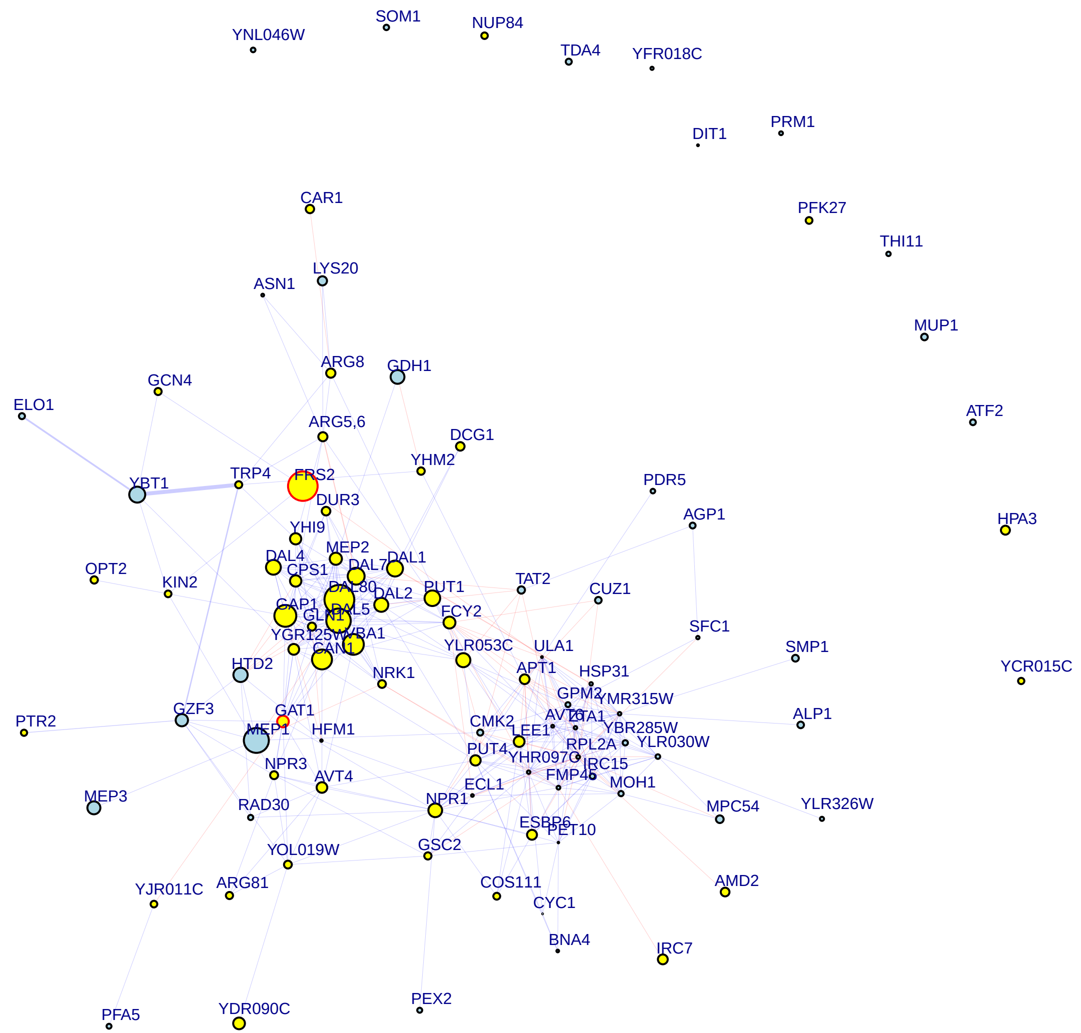

chrVI:156375_T/C top 44 up genes

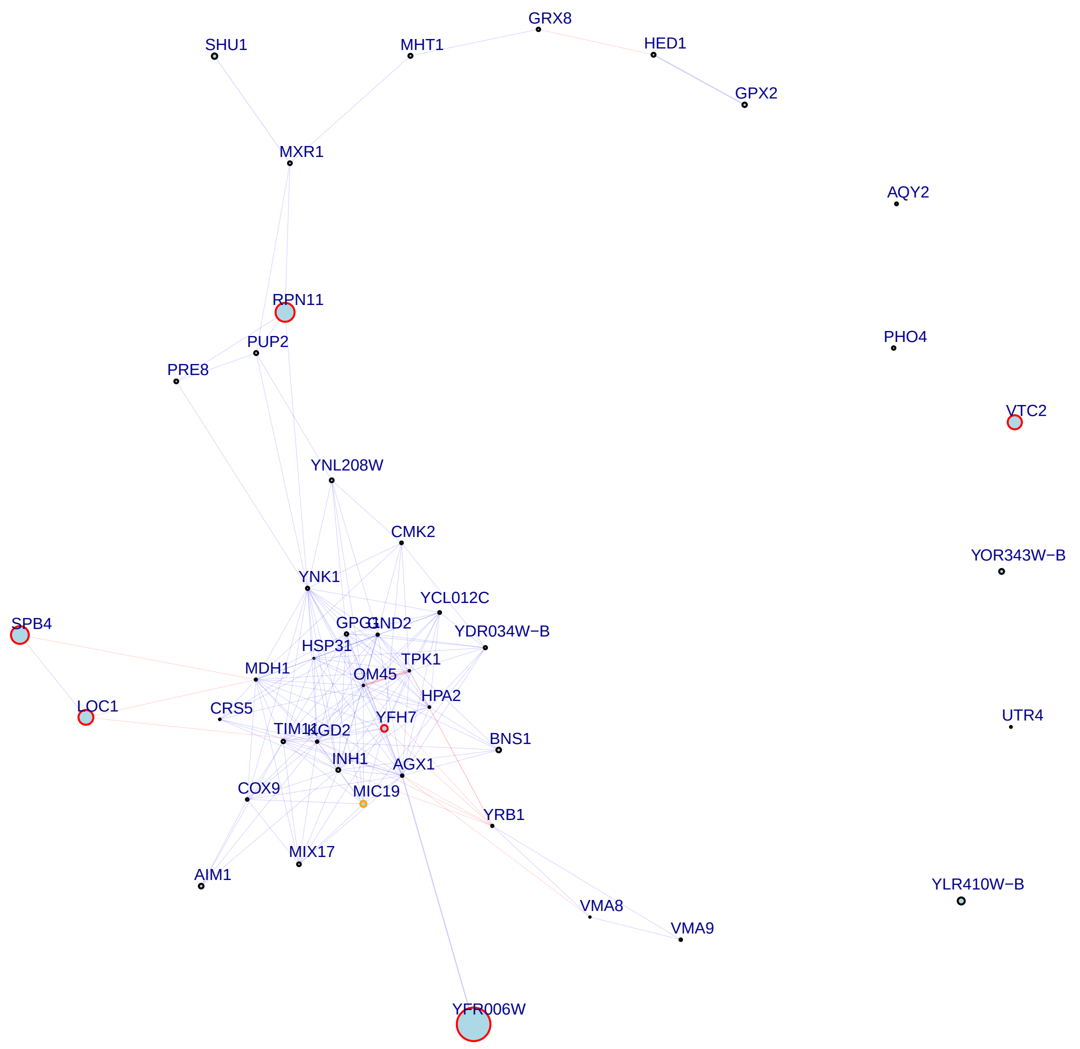

chrVI:156375_T/C top 50 down genes

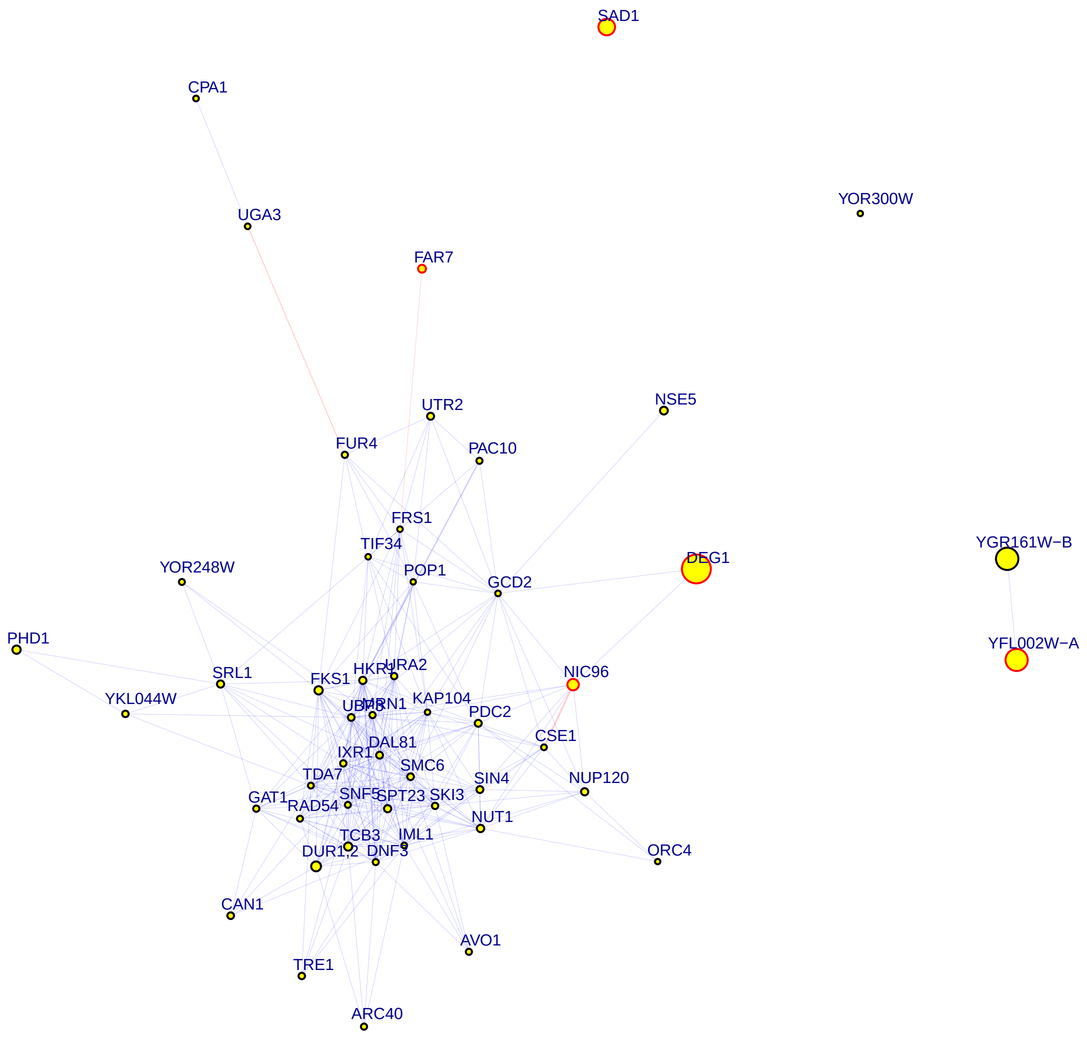

chrVI:156375_T/C top 94 up and down genes

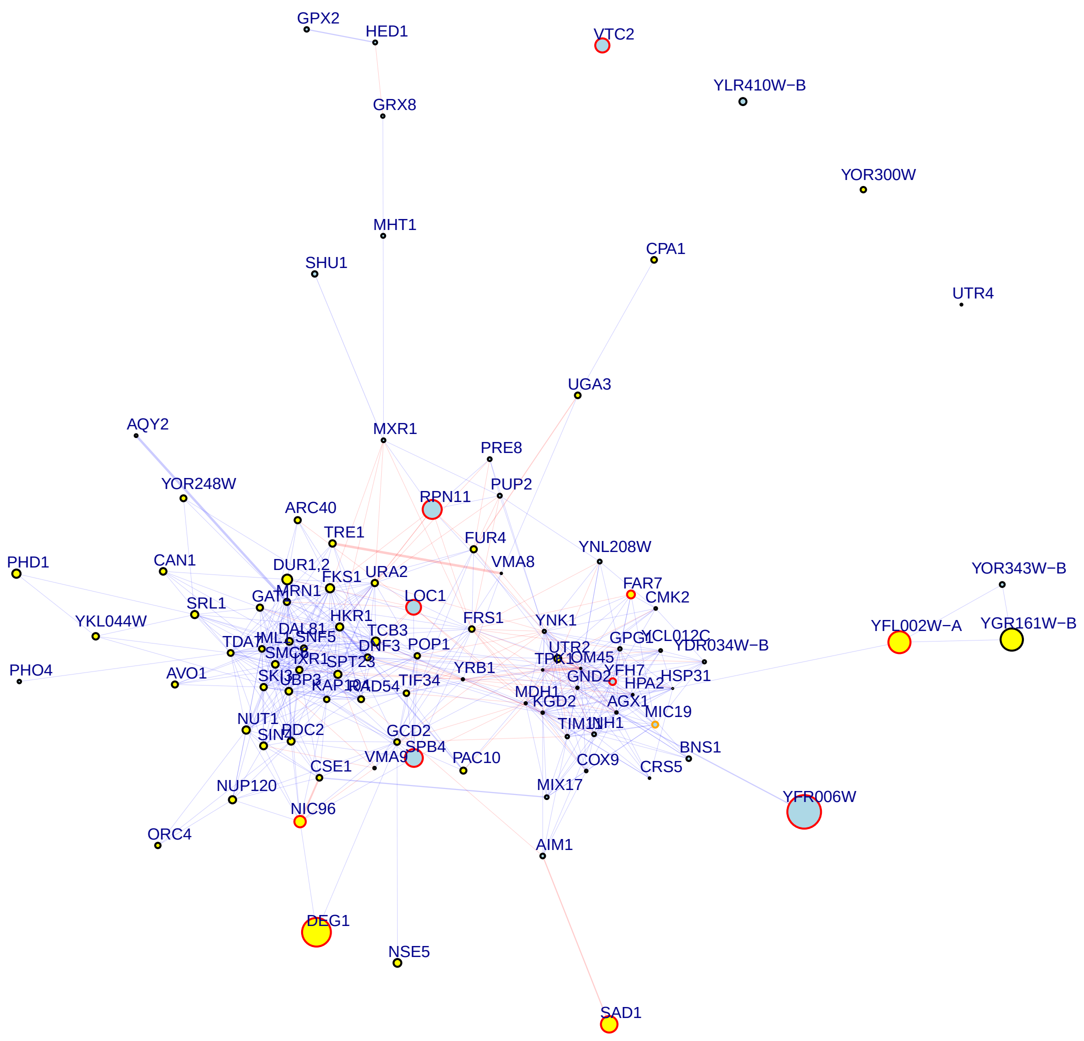

chrVI:229337_G/A top 16 up genes

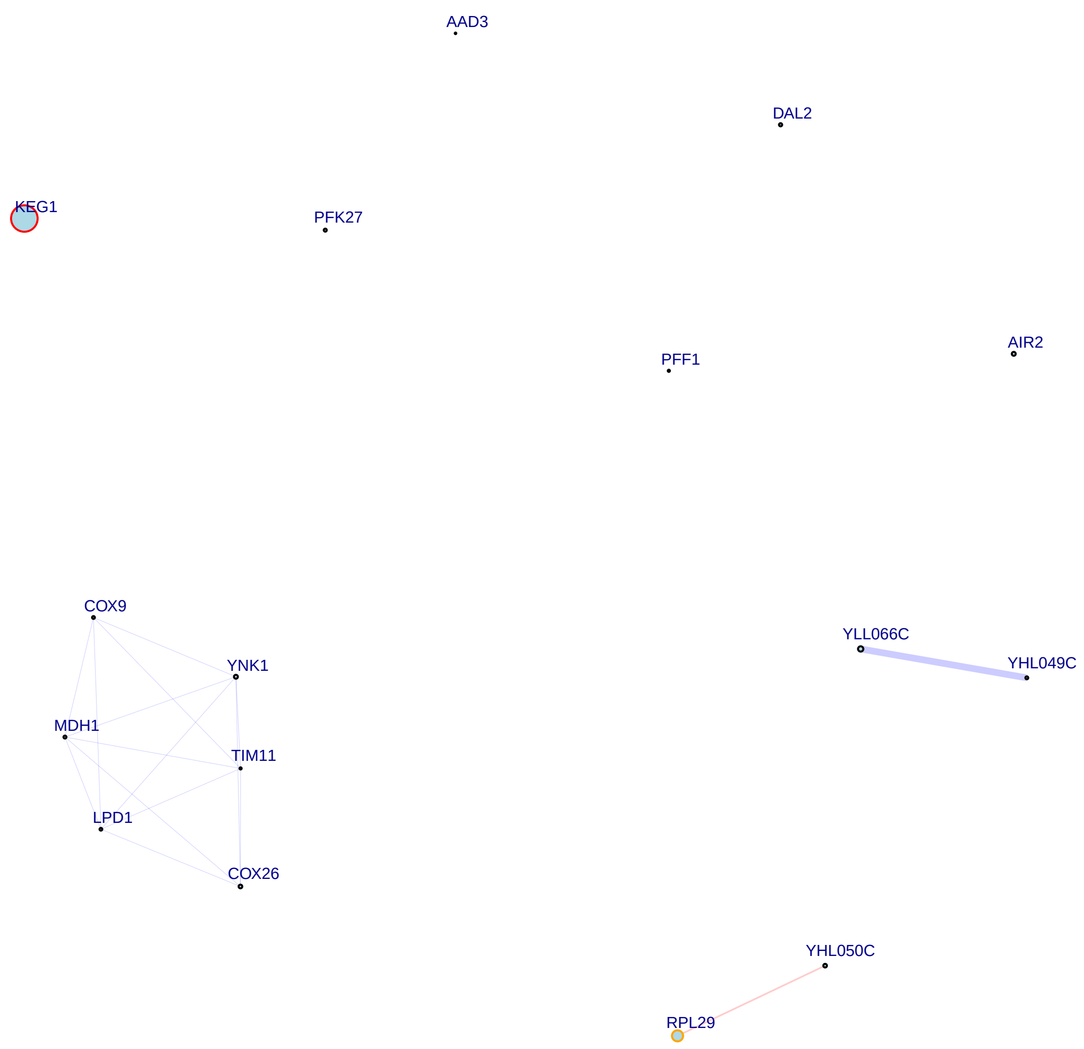

chrVI:229337_G/A top 50 down genes

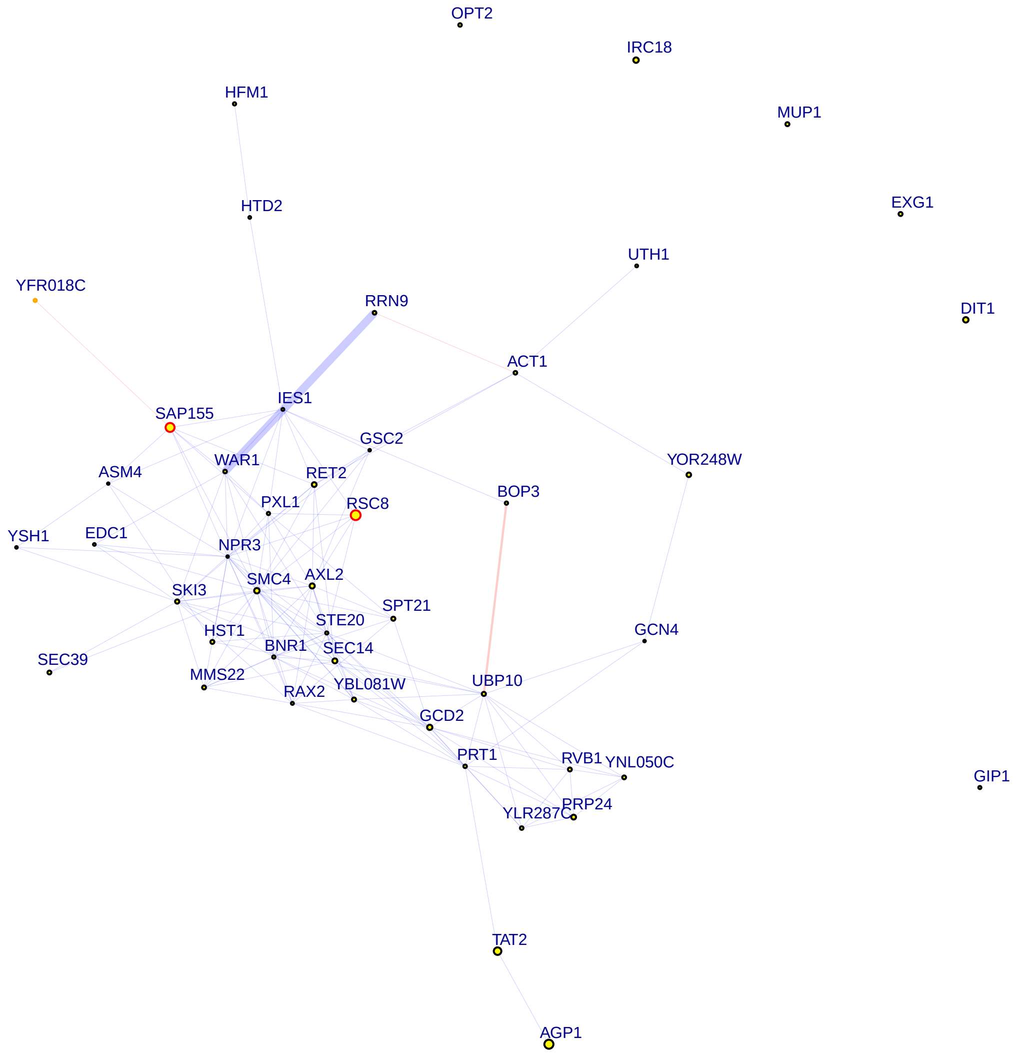

chrVI:229337_G/A top 66 up and down genes

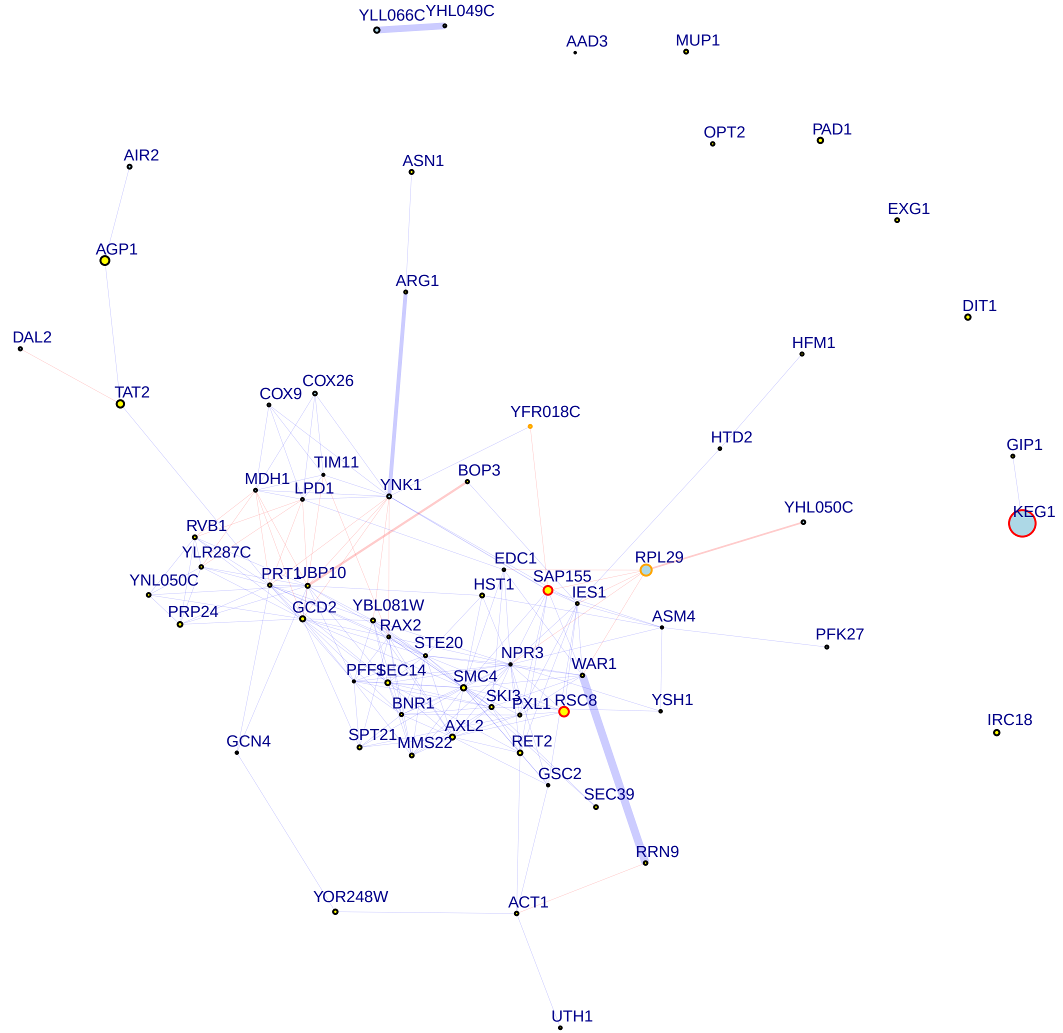

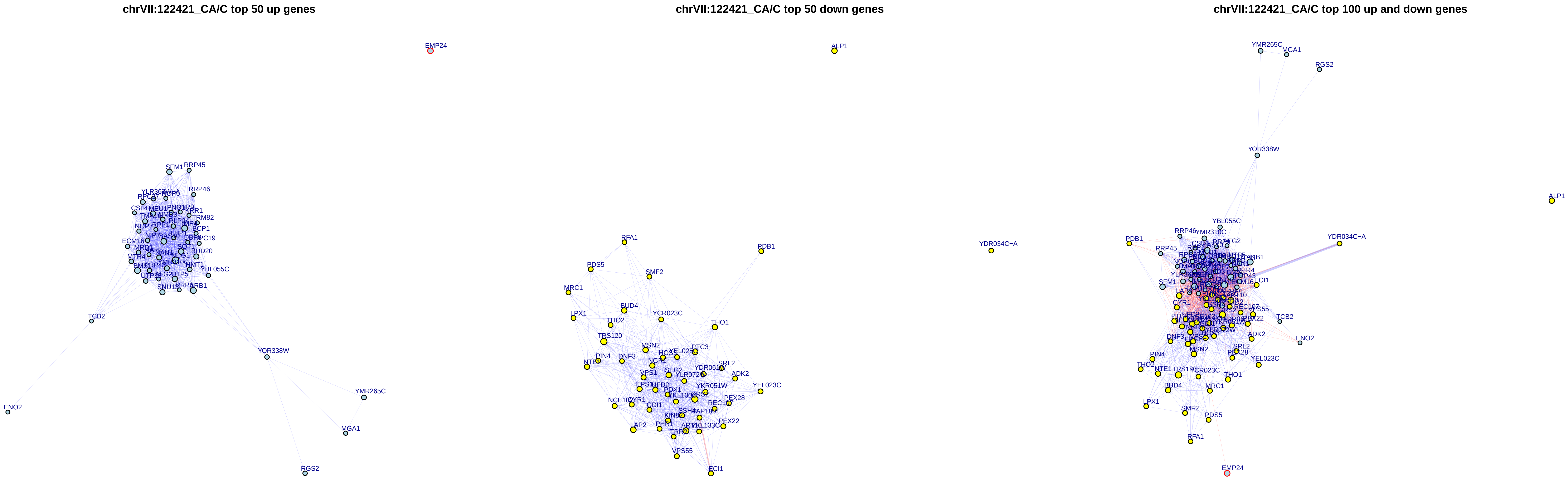
chrVII:122421_CA/C top 50 up genes
chrVII:122421_CA/C top 50 down genes
chrVII:122421_CA/C top 100 up and down genes
EMP24
TCB2
ENO2
YOR338W
YMR265C
MGA1
RGS2
ALP1
PDB1
ECI1
YDR034C−A
YBL055C

chrVII:141743_C/T top 50 up genes

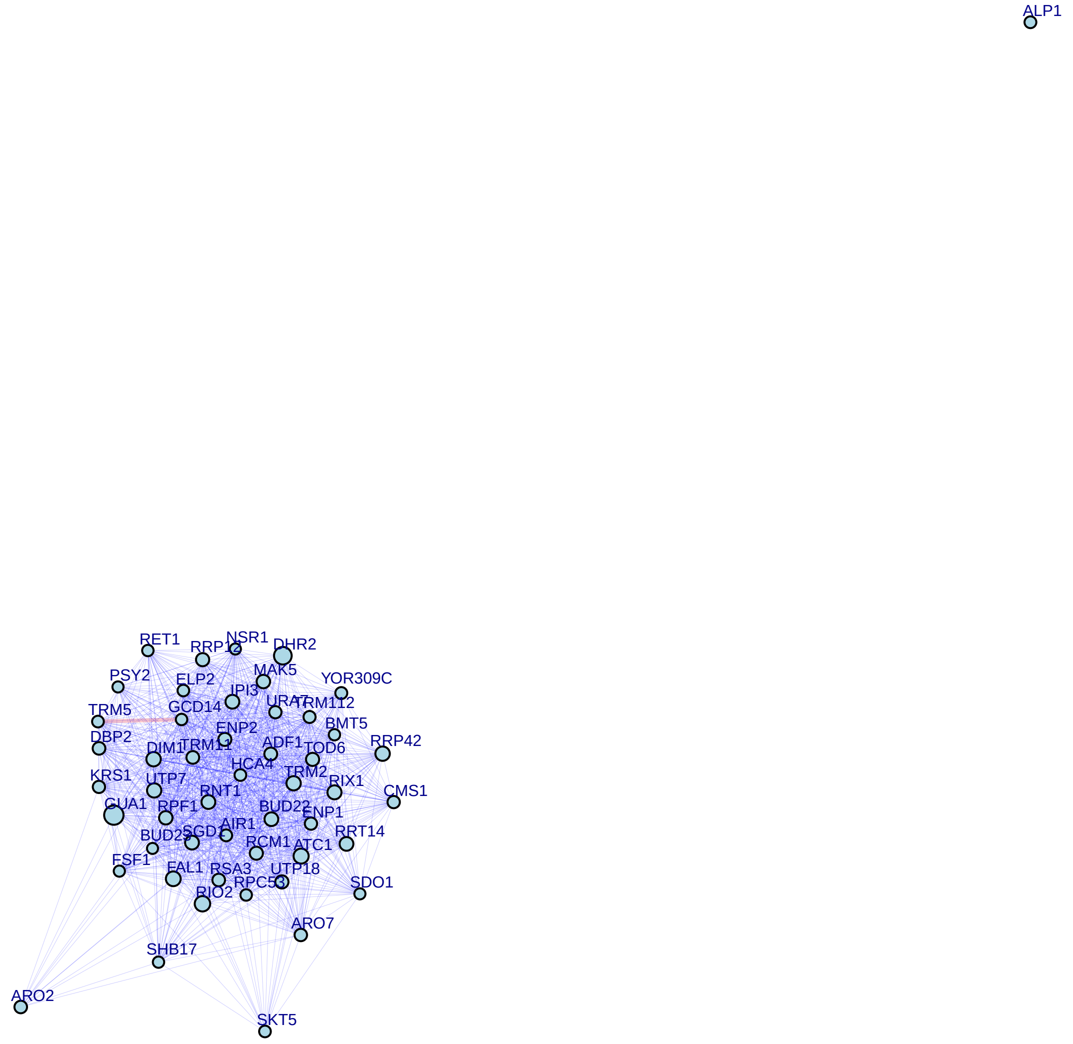

chrVII:141743_C/T top 50 down genes

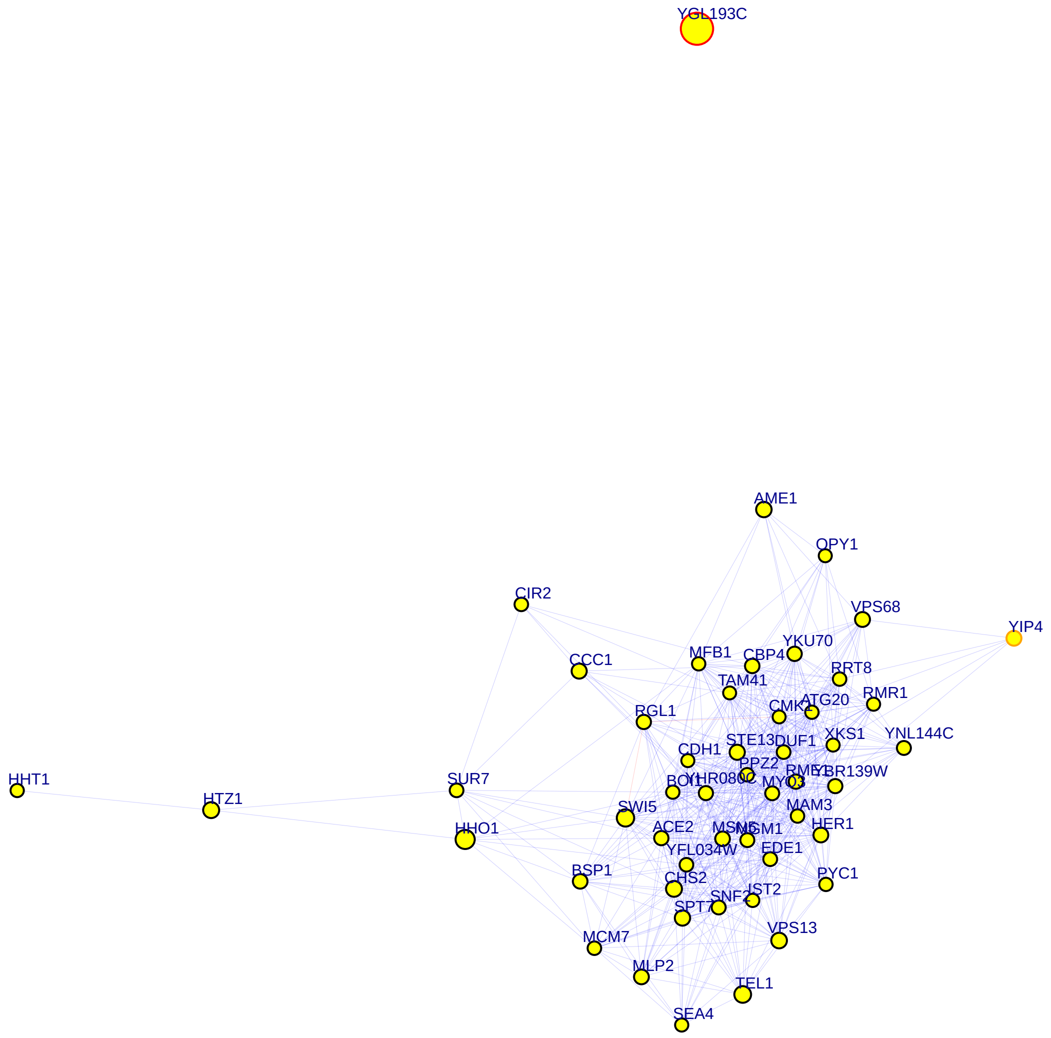

chrVII:141743_C/T top 100 up and down genes

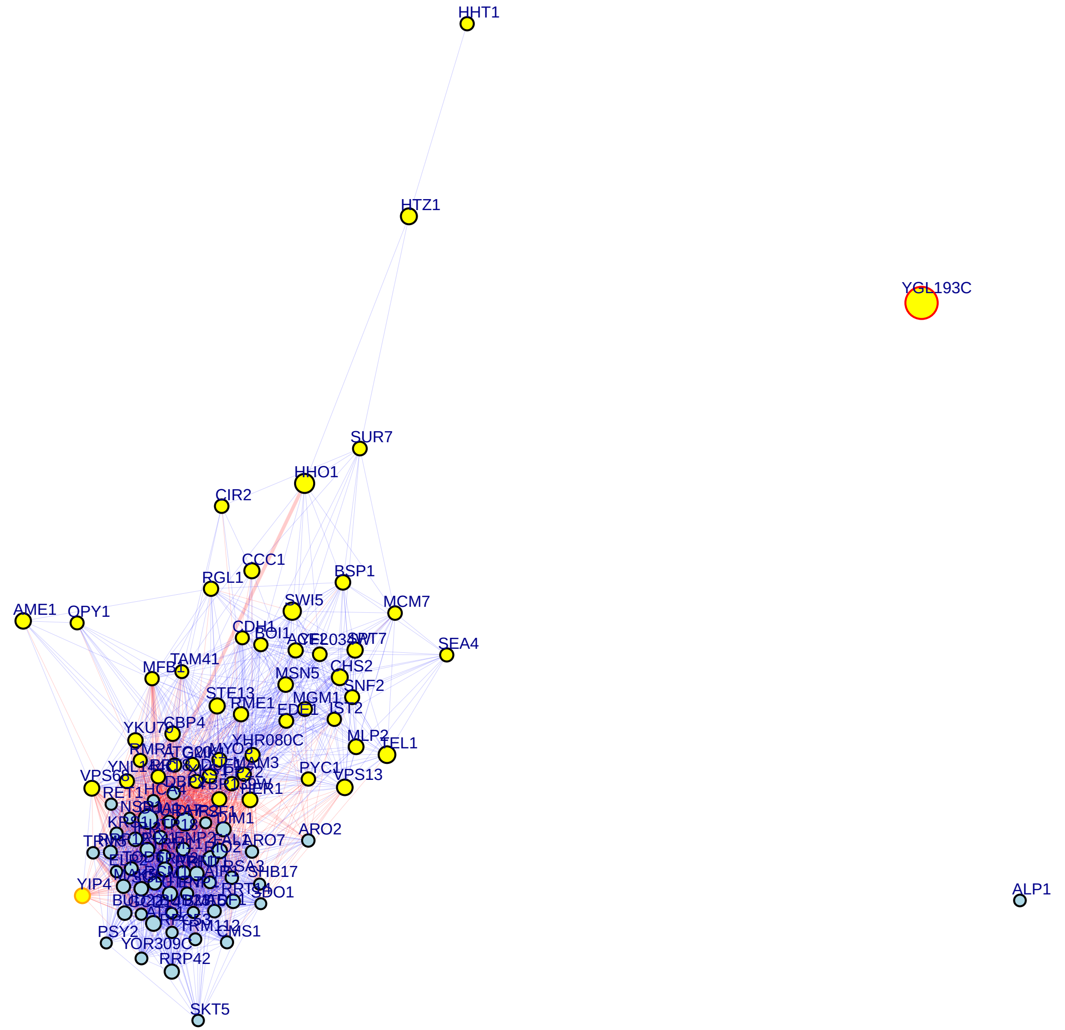

chrVII:178509_T/C top 50 up genes

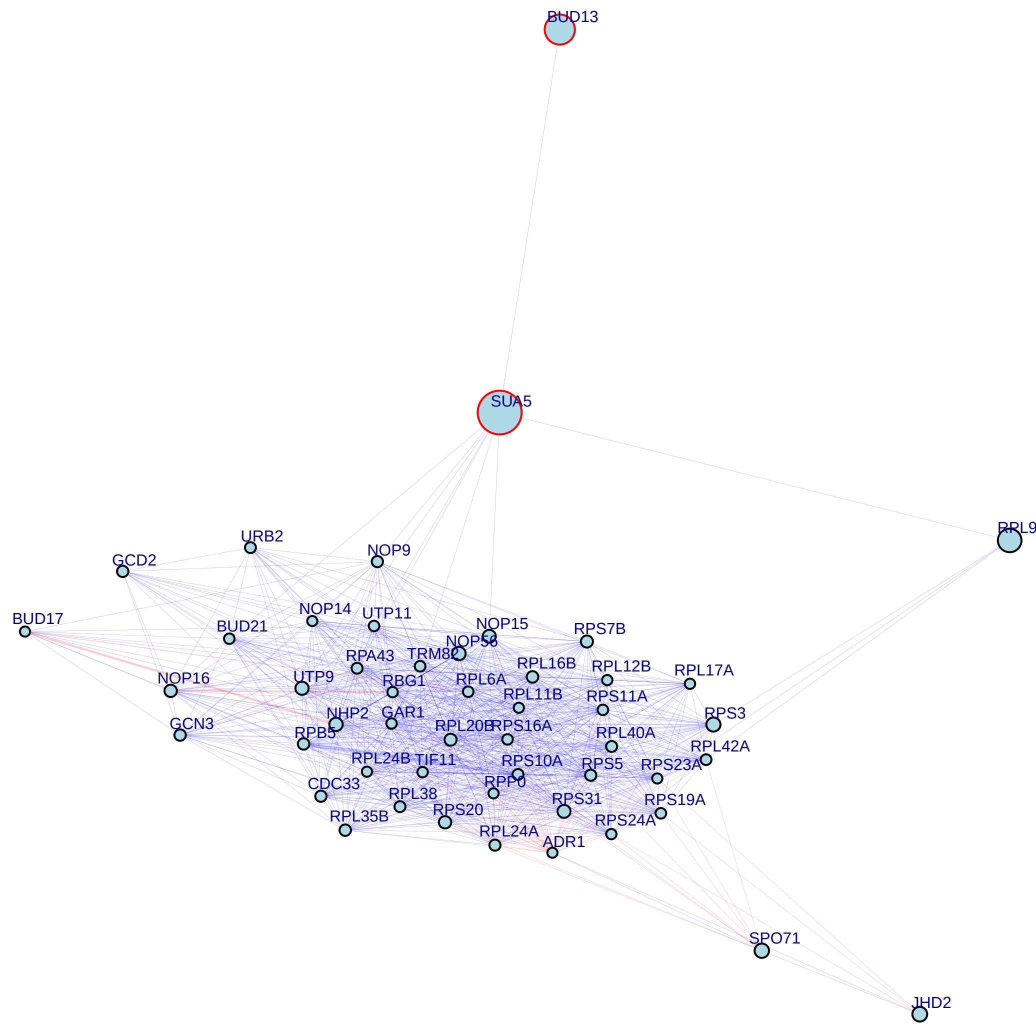

chrVII:178509_T/C top 50 down genes

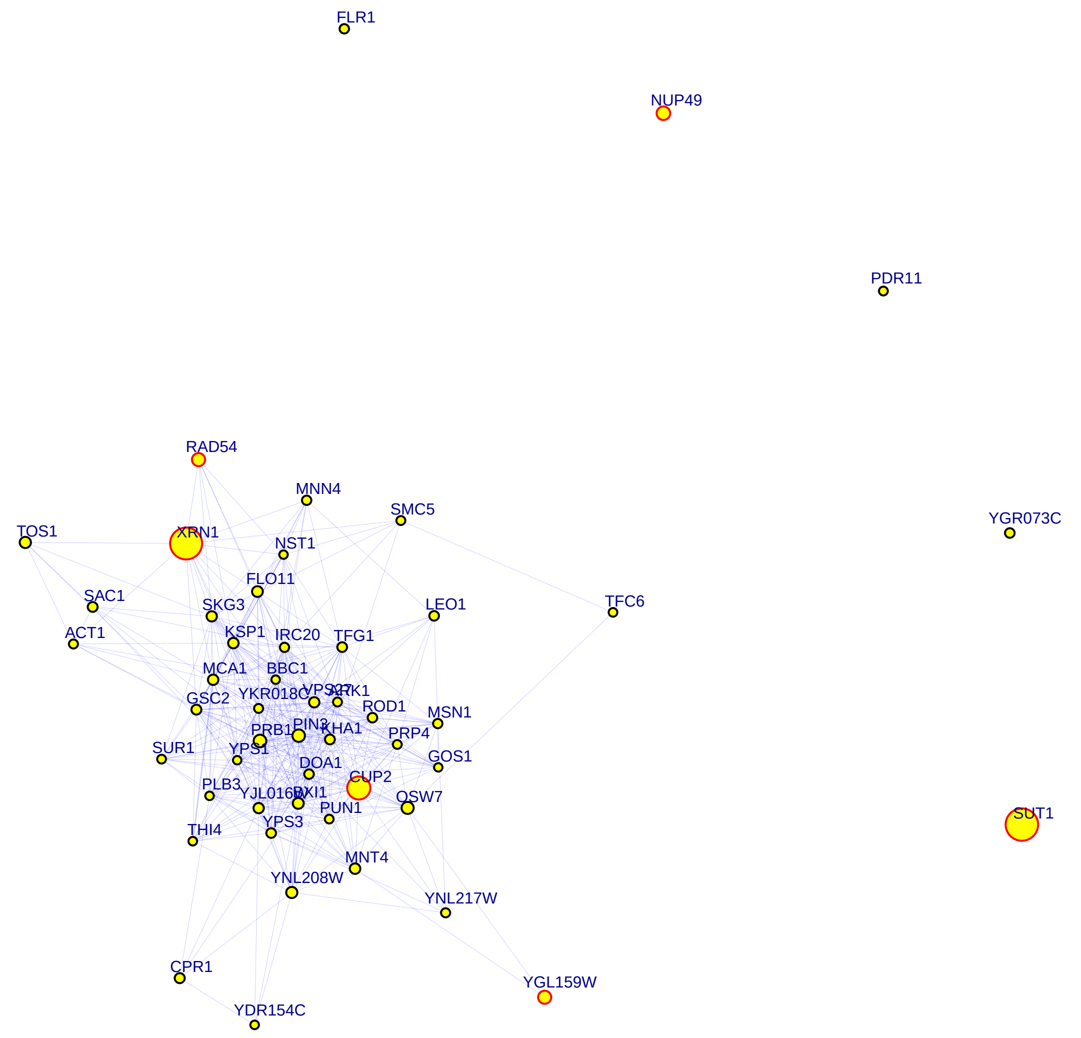

chrVII:178509_T/C top 100 up and down genes

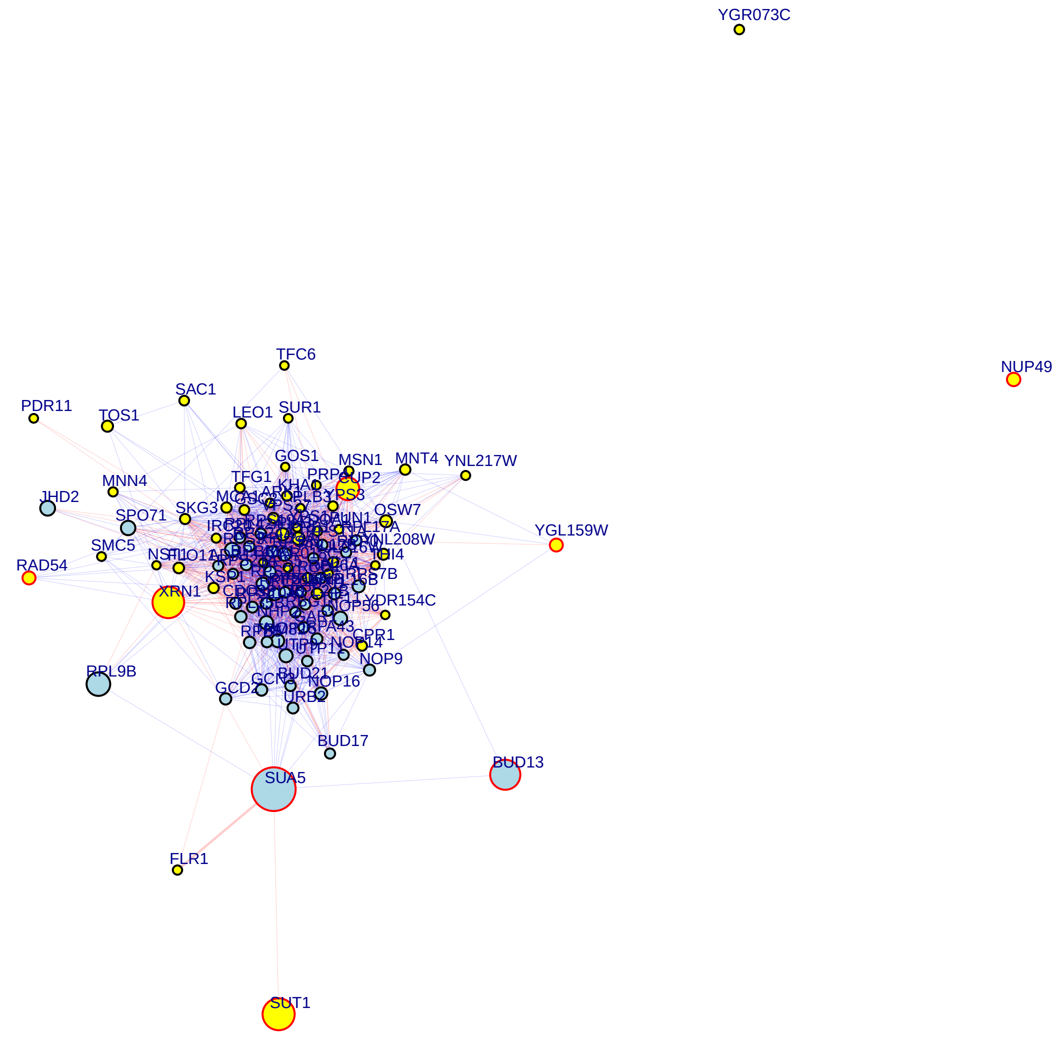

chrVII:372033_G/C top 50 up genes

chrVII:372033_G/C top 50 down genes

chrVII:372033_G/C top 100 up and down genes

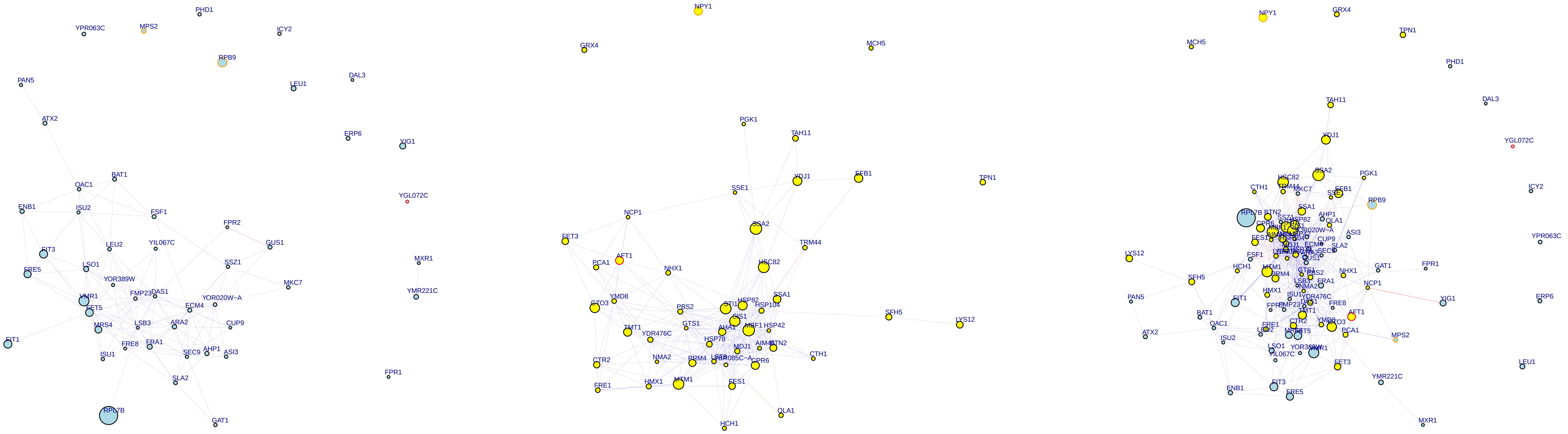

chrVII:397449_G/A top 50 up genes

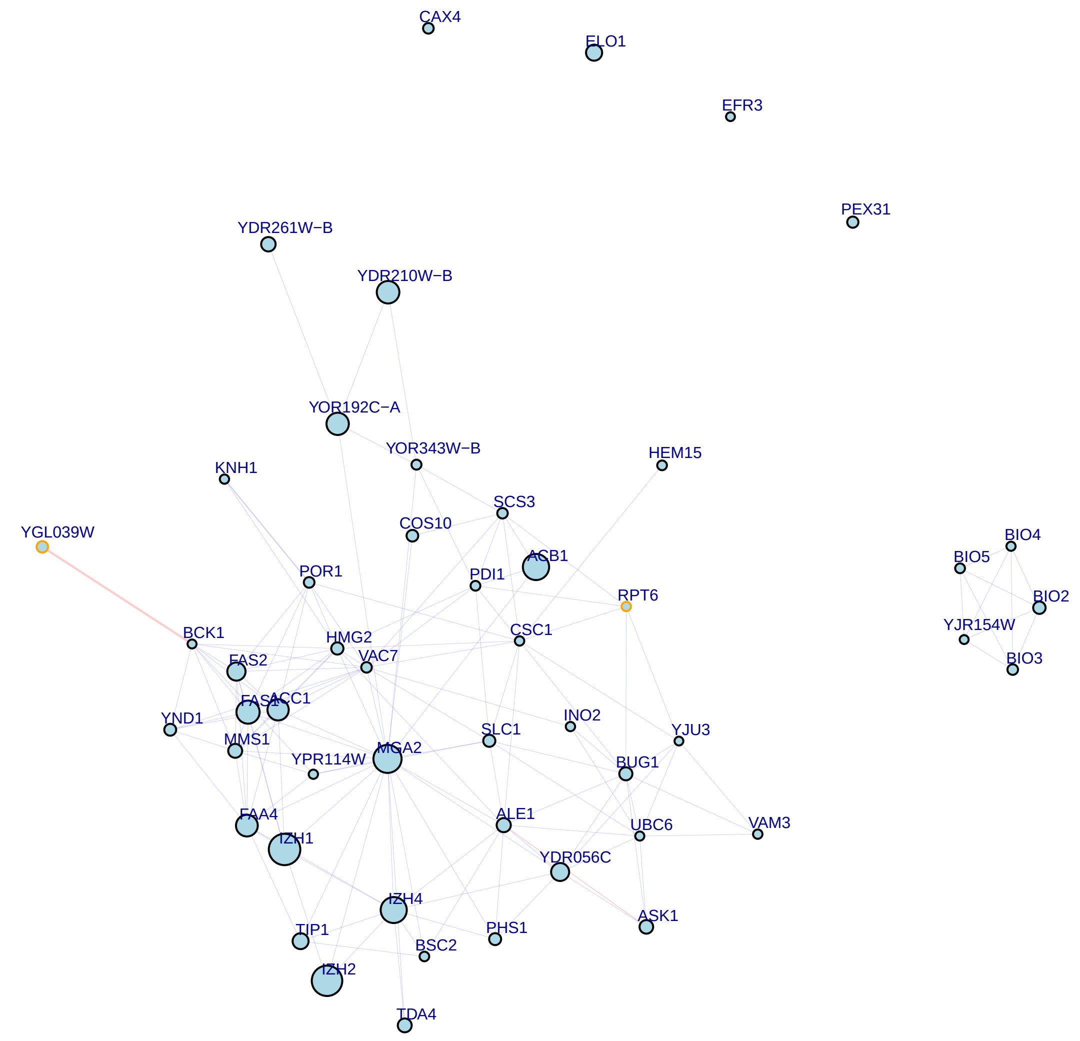

chrVII:397449_G/A top 50 down genes

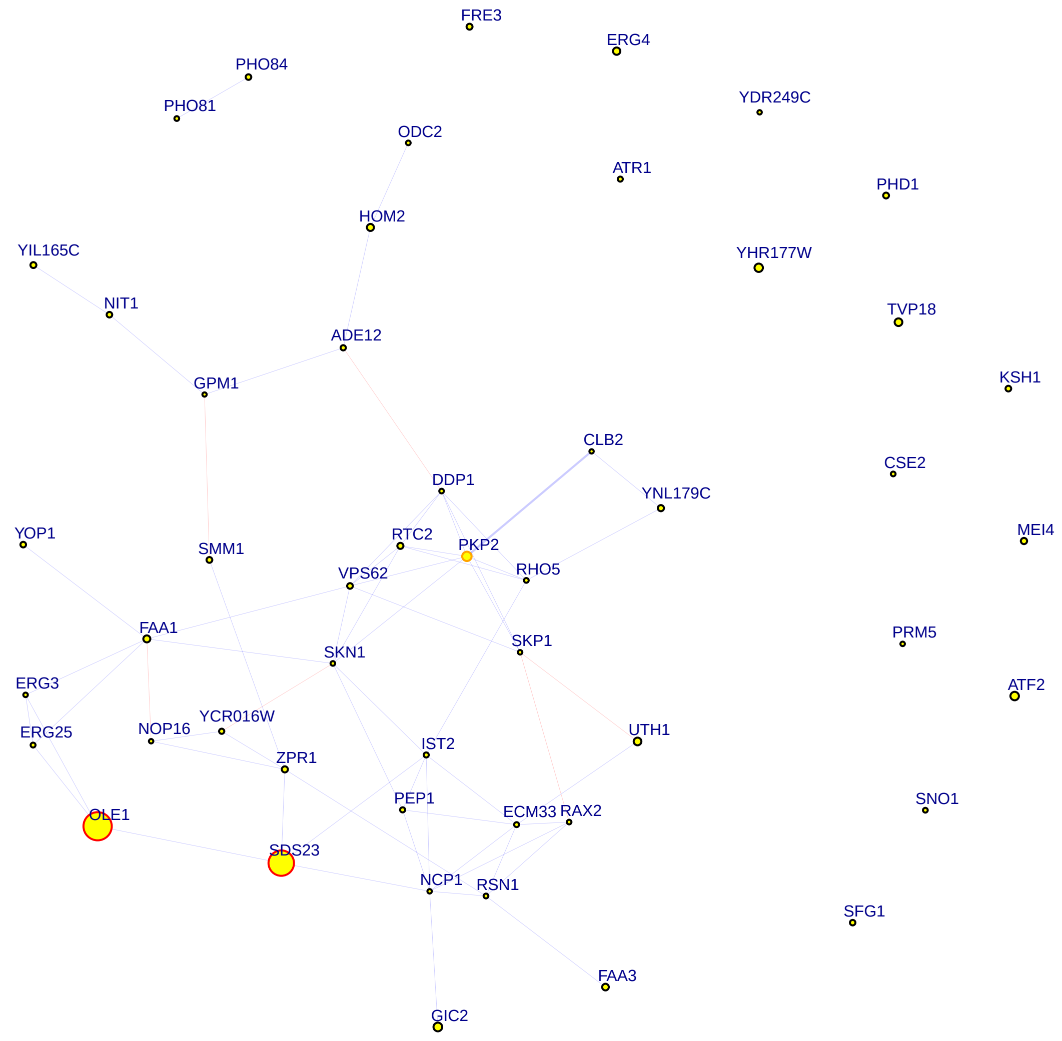

chrVII:397449_G/A top 100 up and down genes

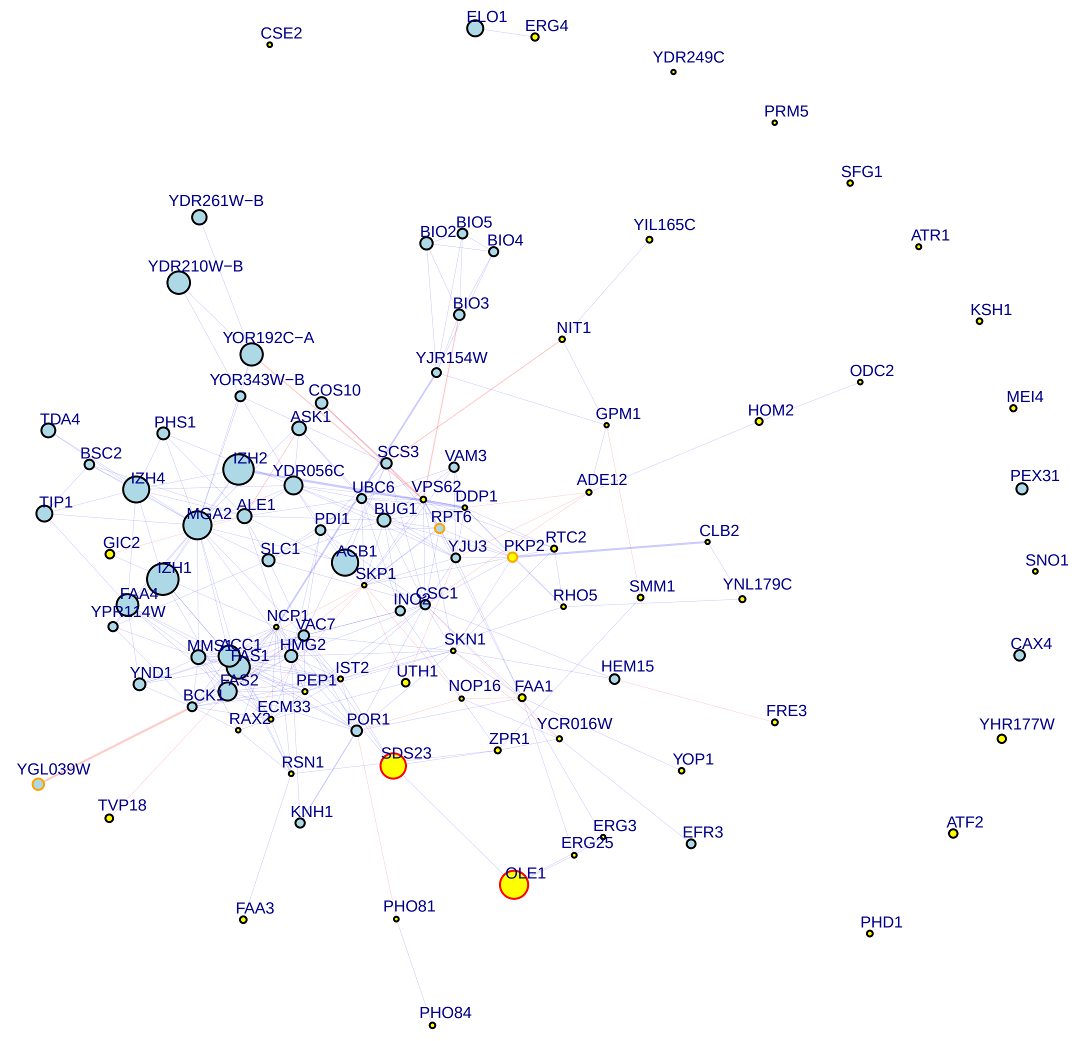

chrVII:470028_A/G top 50 up genes

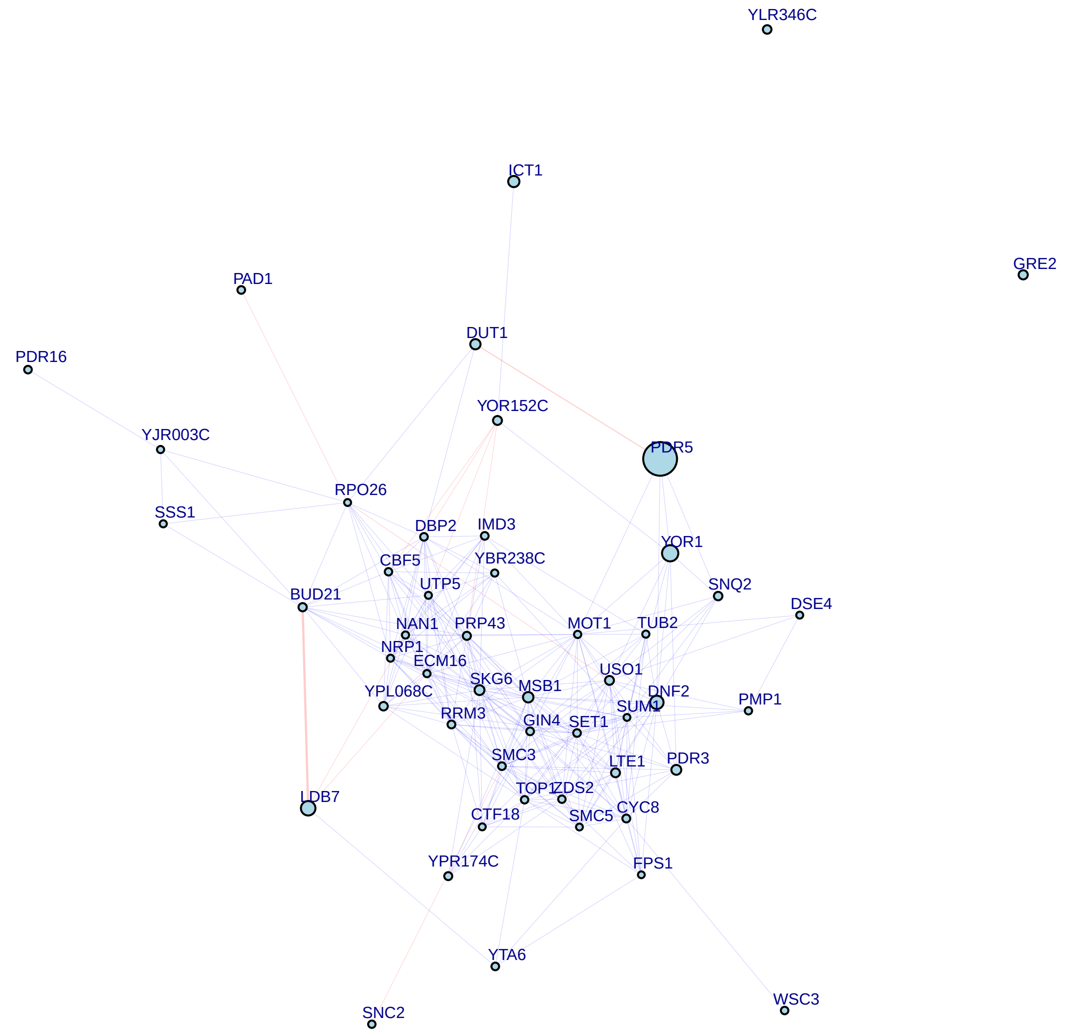

chrVII:470028_A/G top 50 down genes

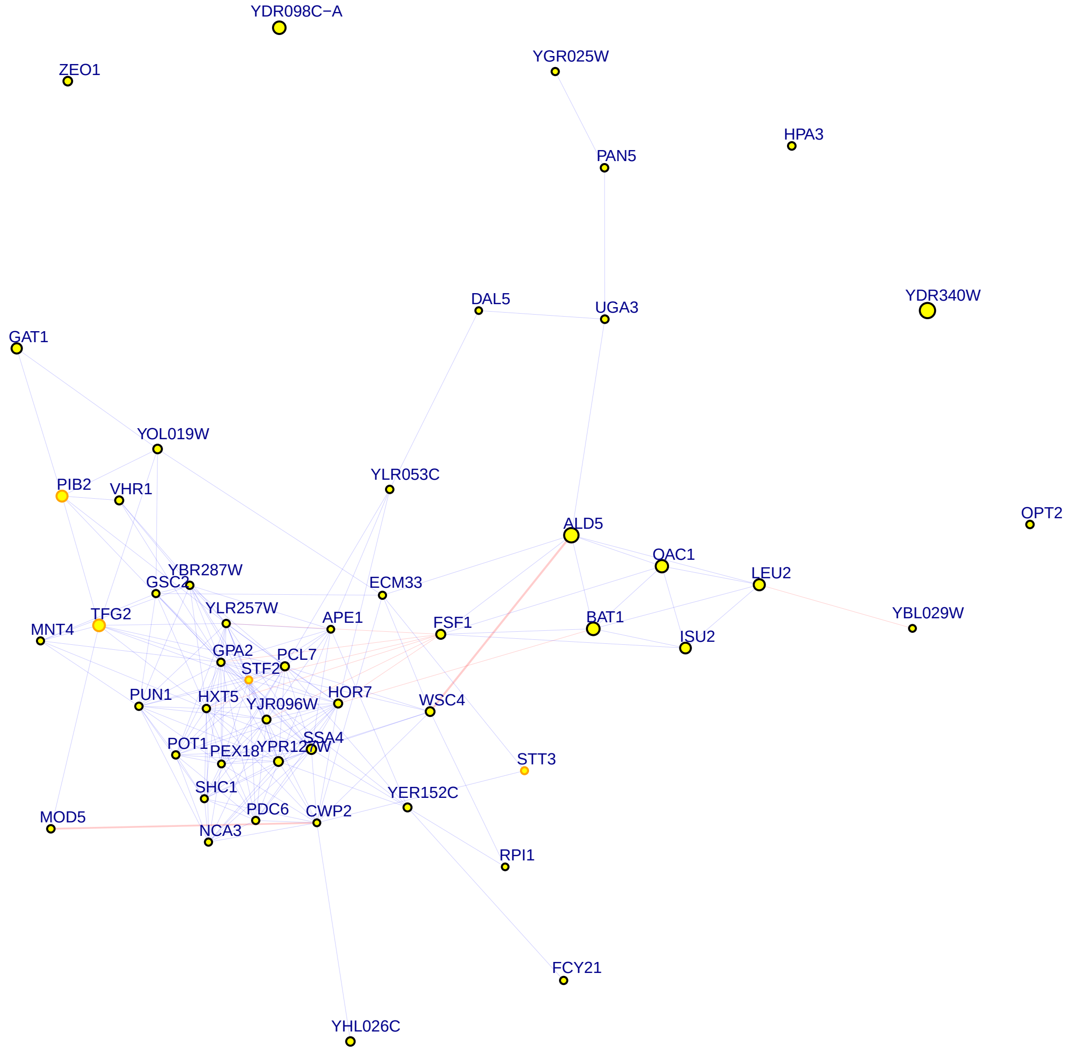

chrVII:470028_A/G top 100 up and down genes

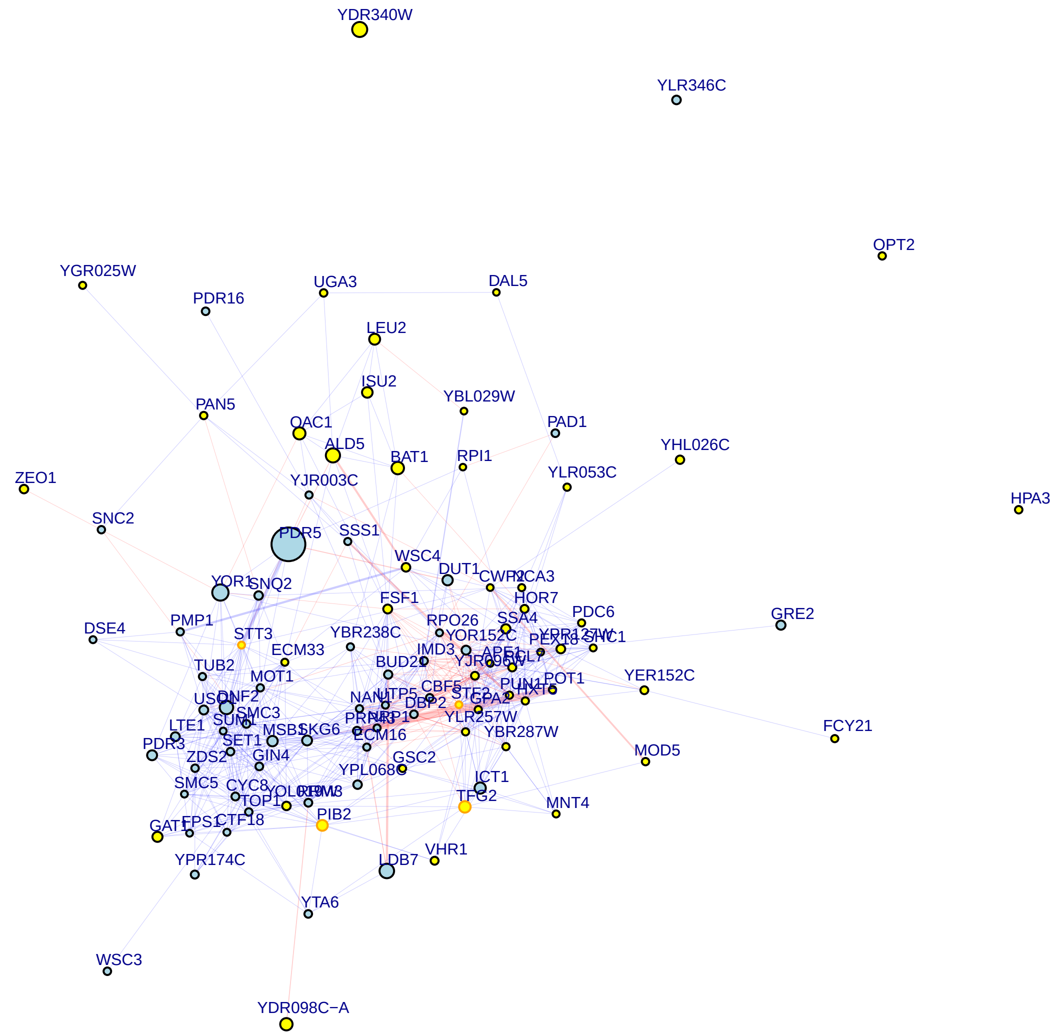

chrVII:656248_T/G top 50 up genes

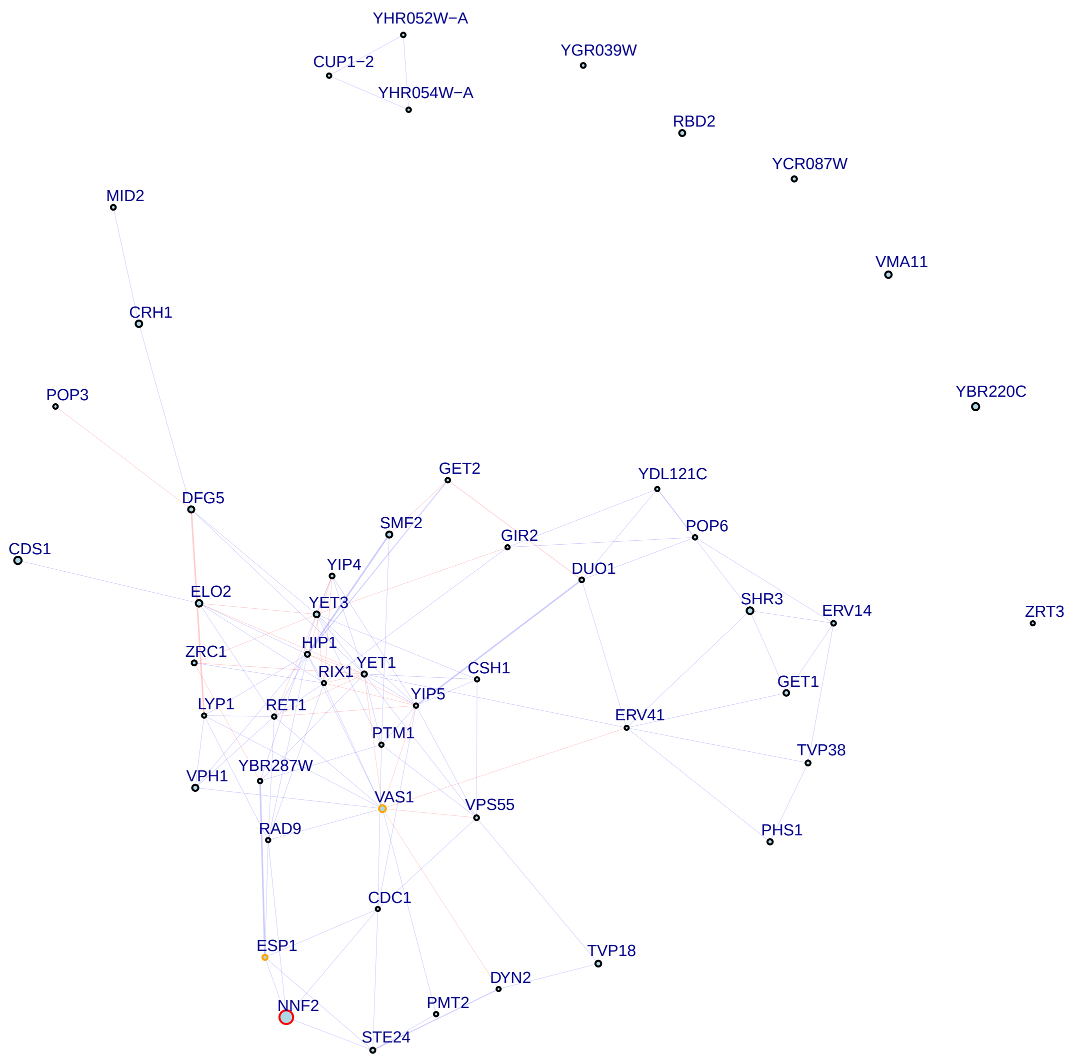

chrVII:656248_T/G top 50 down genes

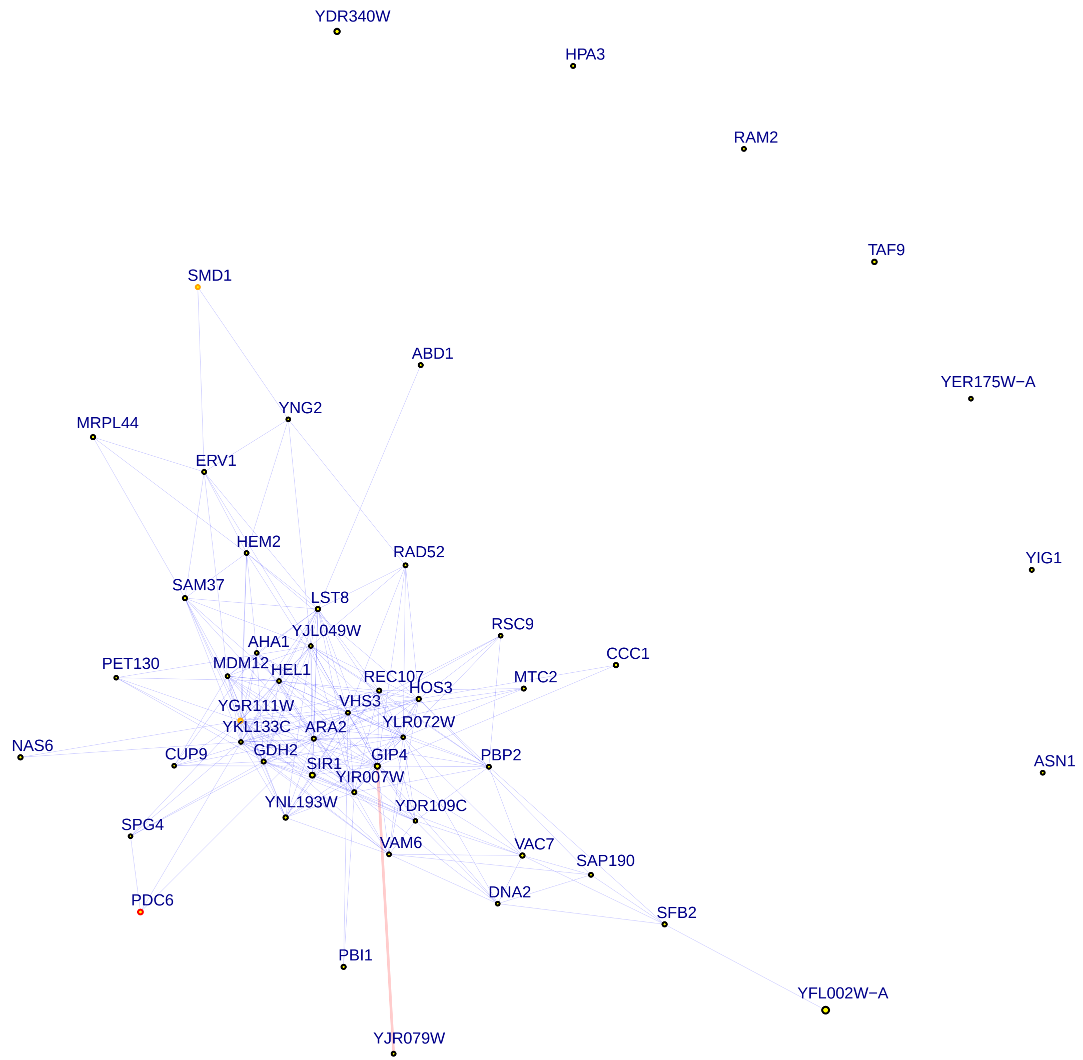

chrVII:656248_T/G top 100 up and down genes

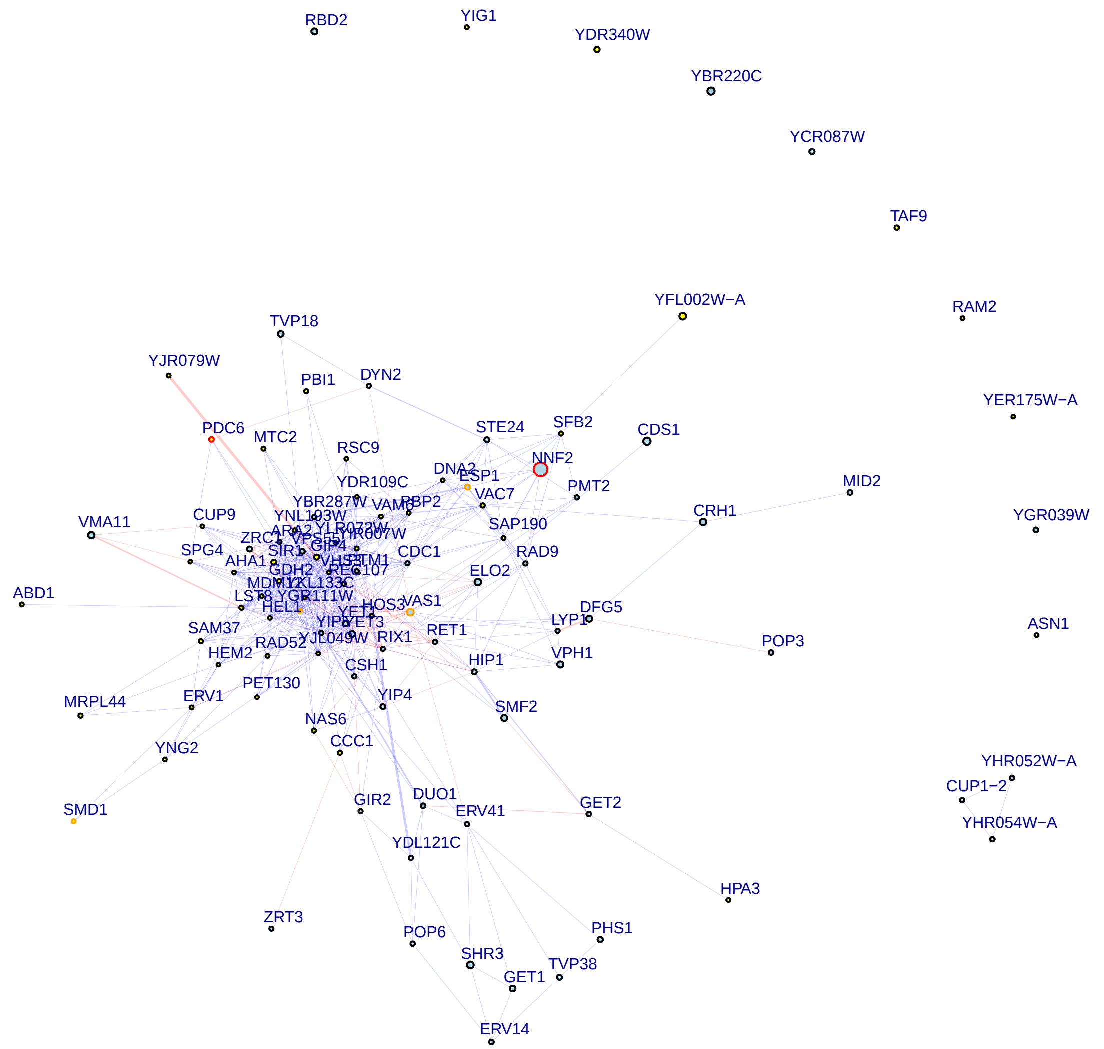

chrVII:850923_T/C top 50 up genes

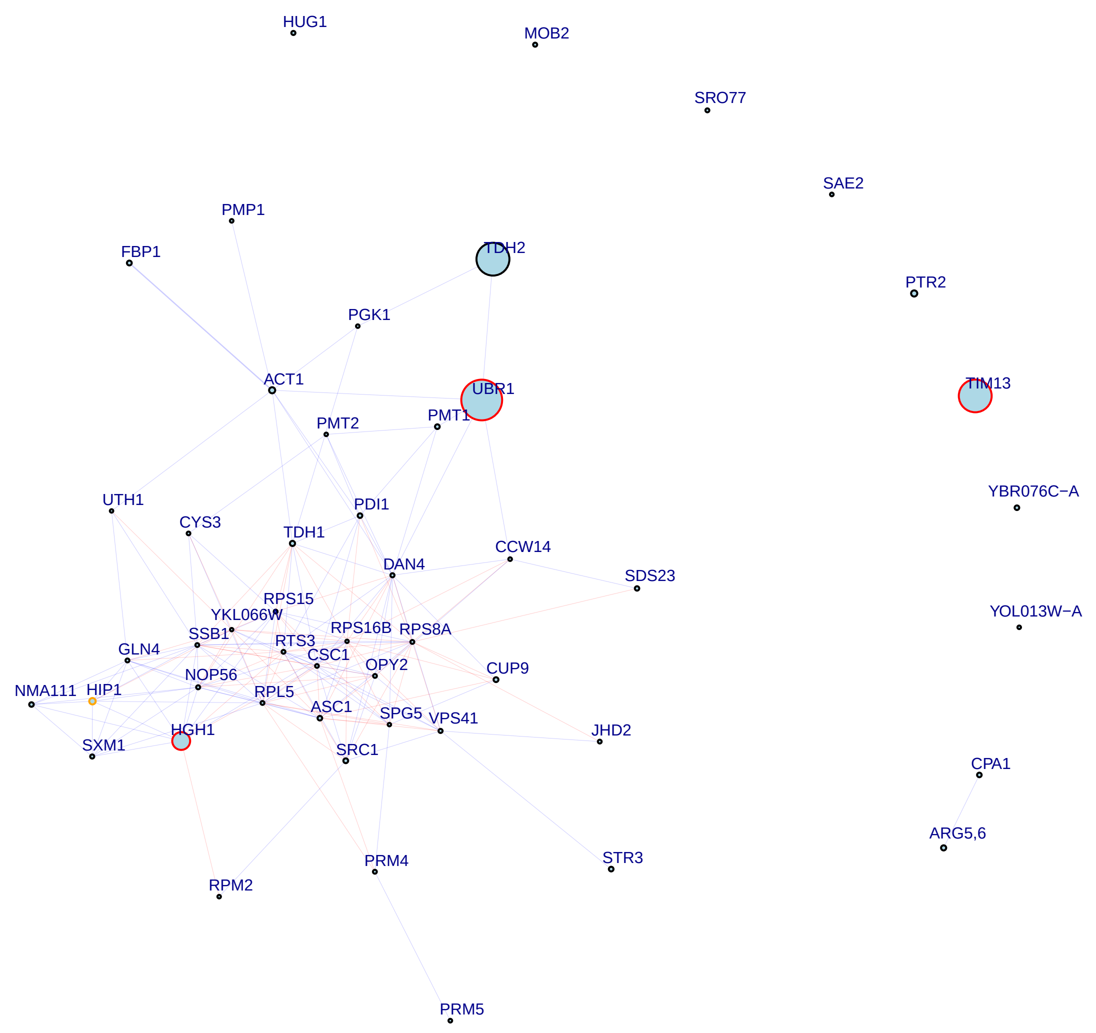

chrVII:850923_T/C top 50 down genes

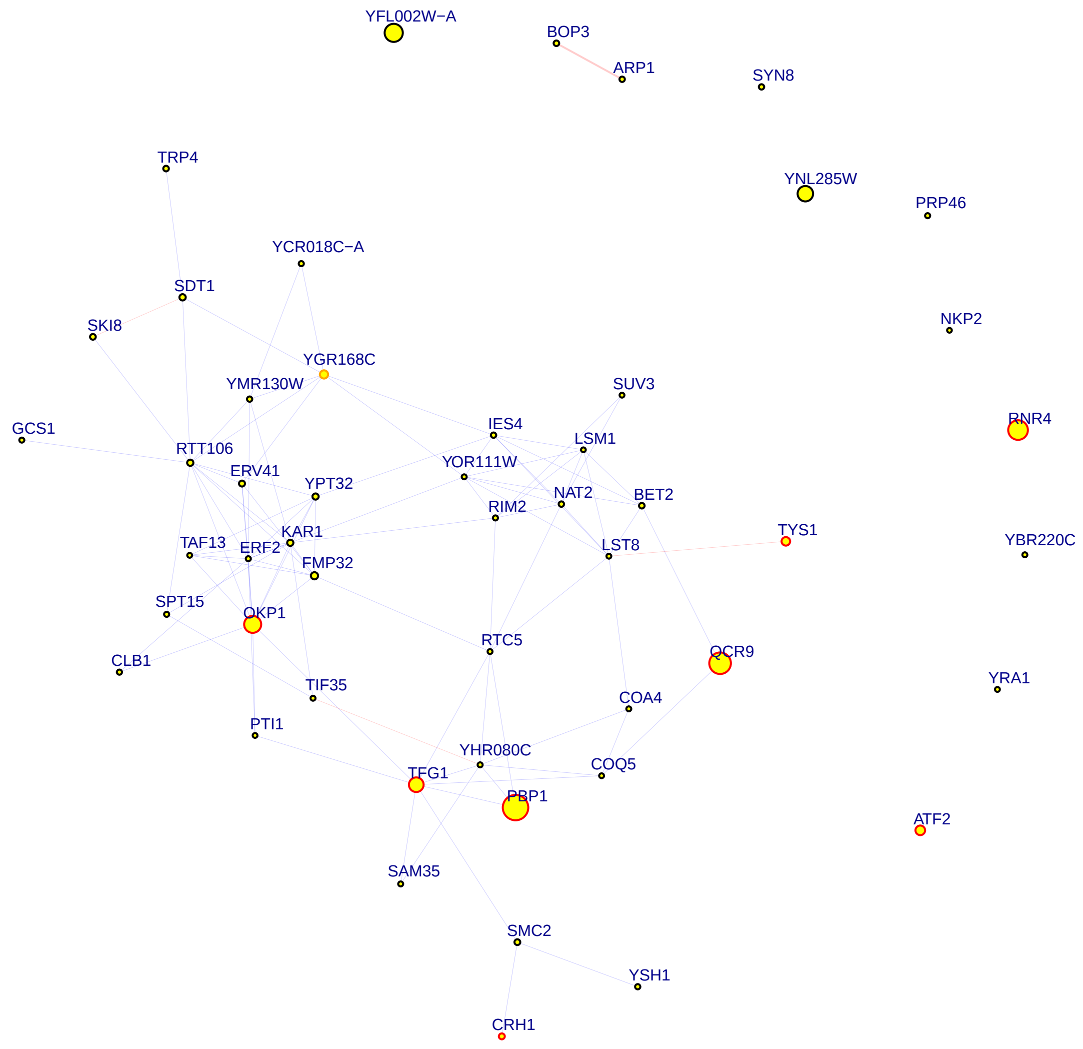

chrVII:850923_T/C top 100 up and down genes

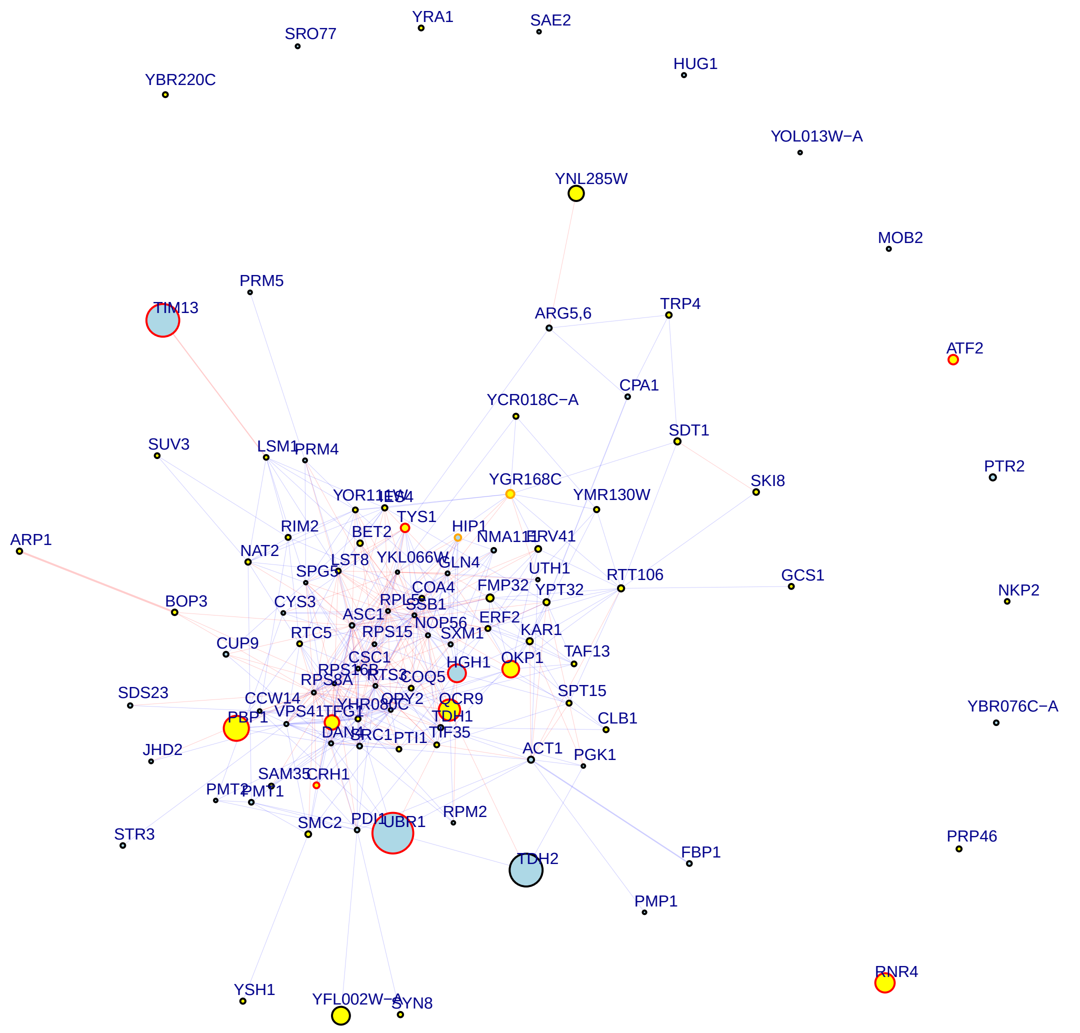

chrVII:984362_C/T top 50 up genes

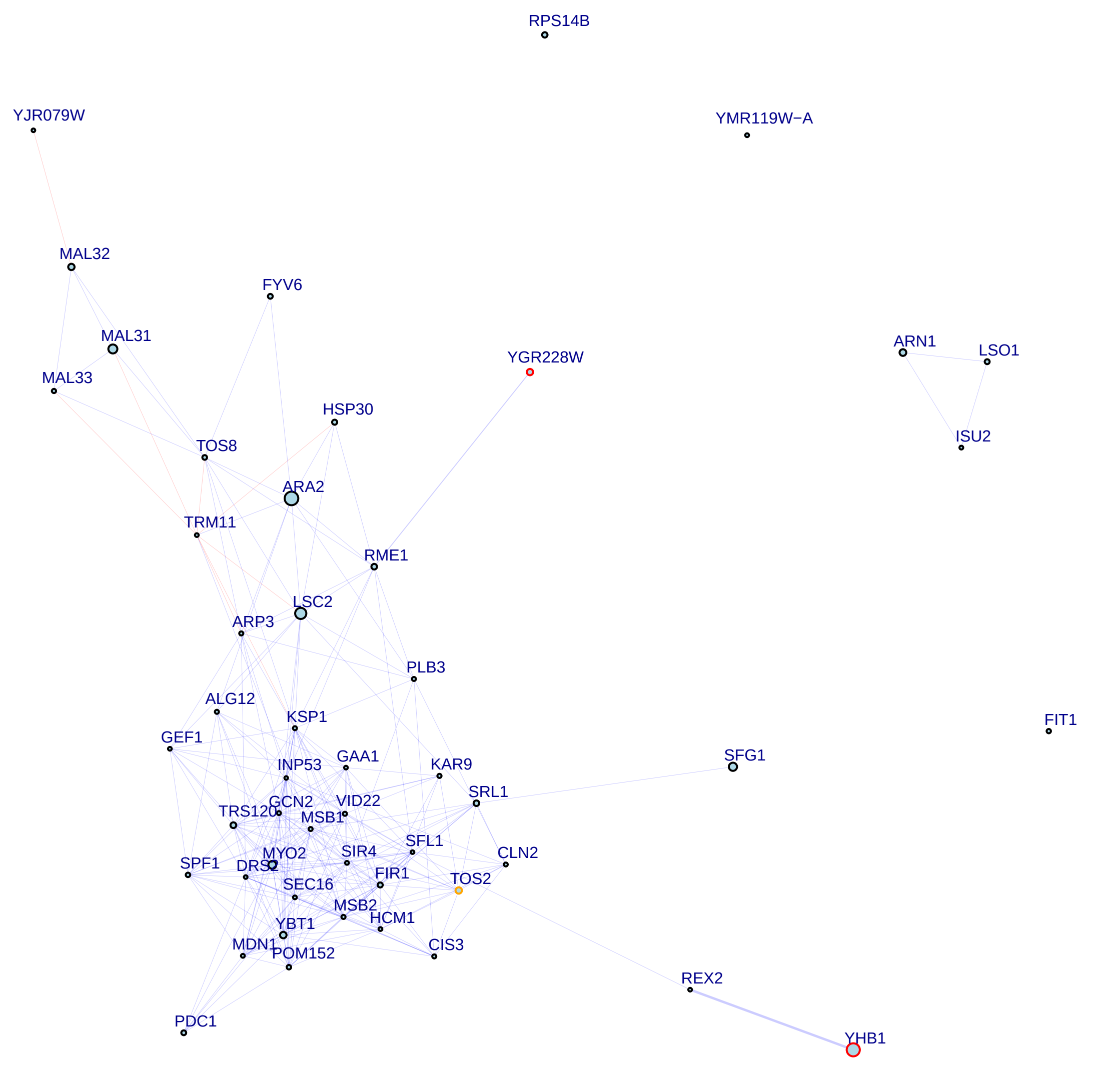

chrVII:984362_C/T top 50 down genes

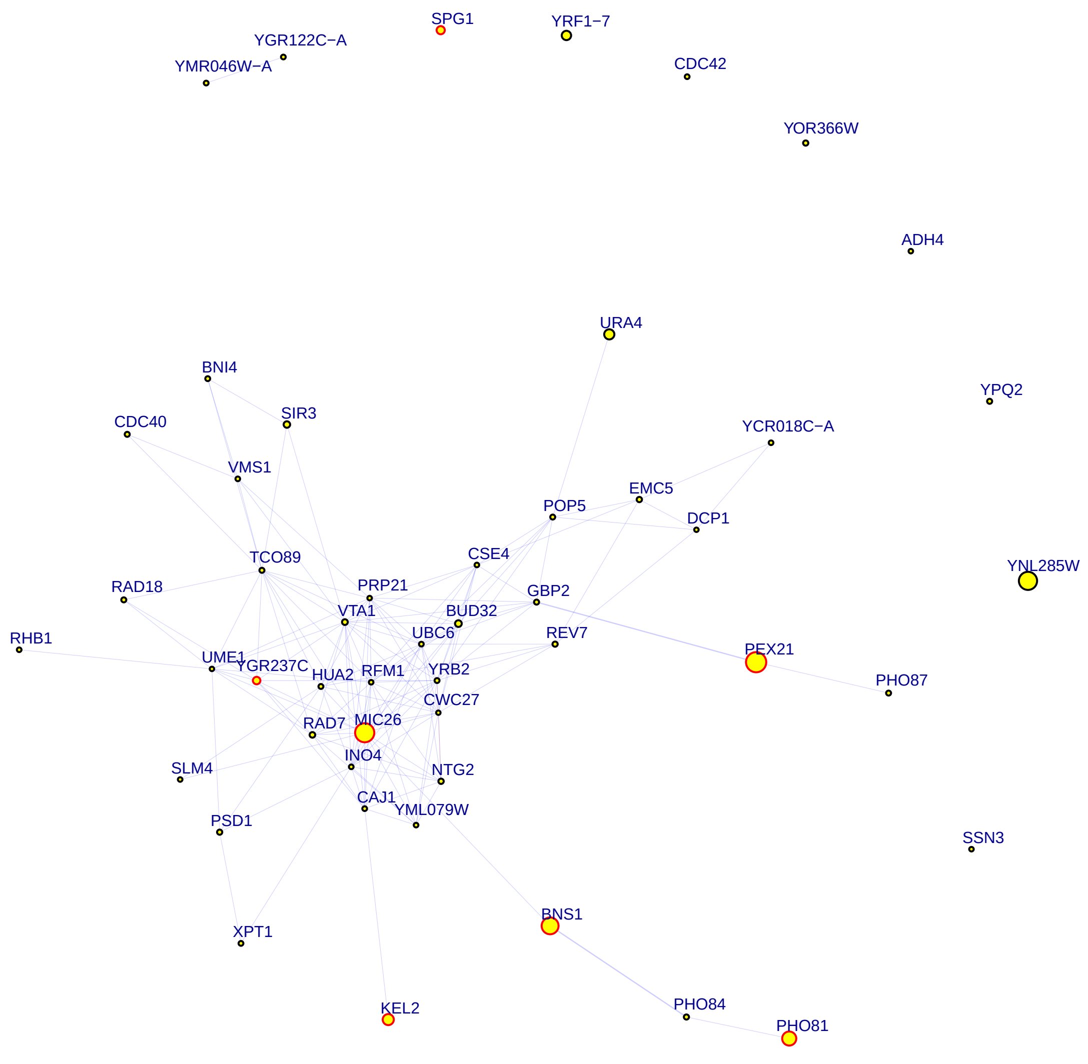

chrVII:984362_C/T top 100 up and down genes

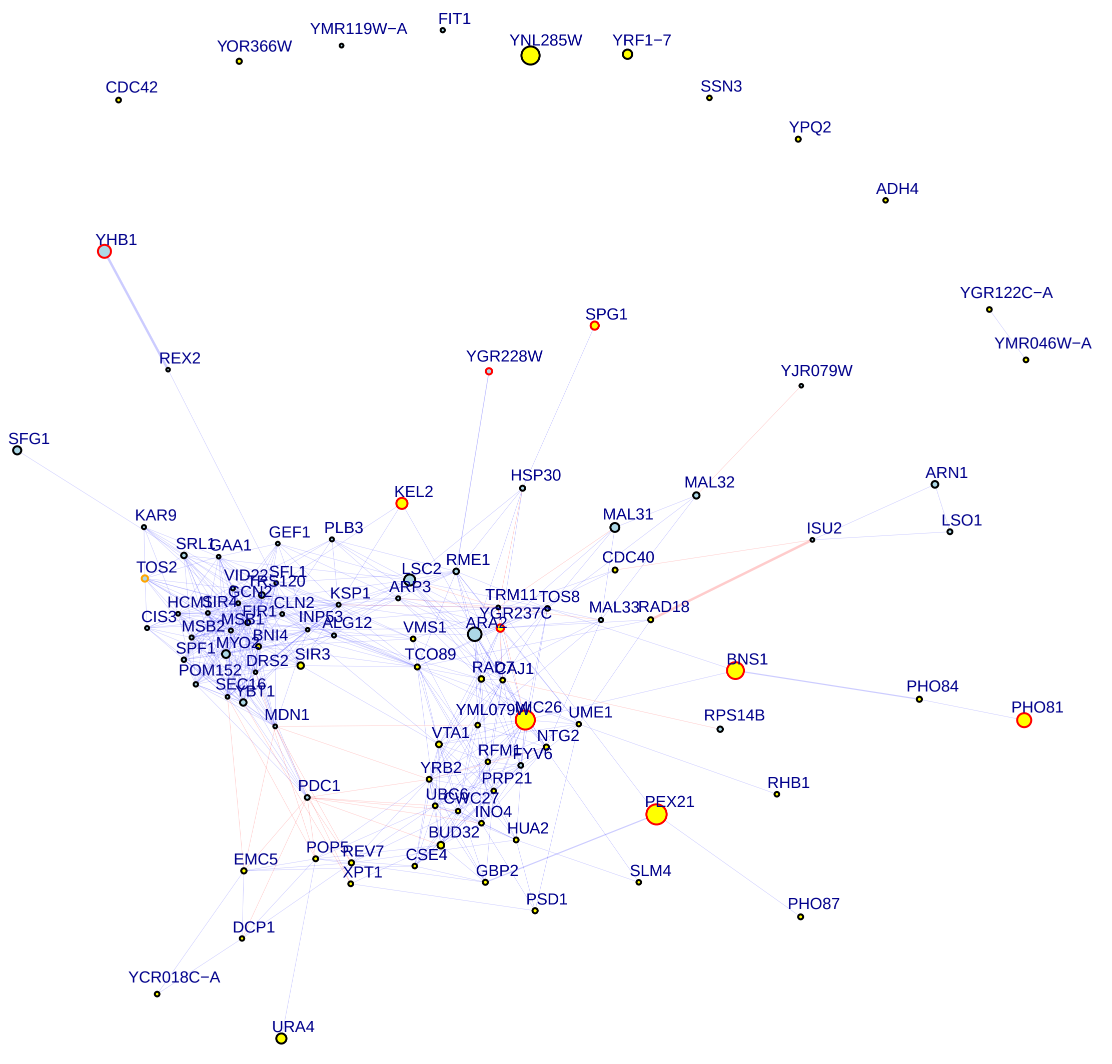

chrVIII:104423_C/A top 50 up genes

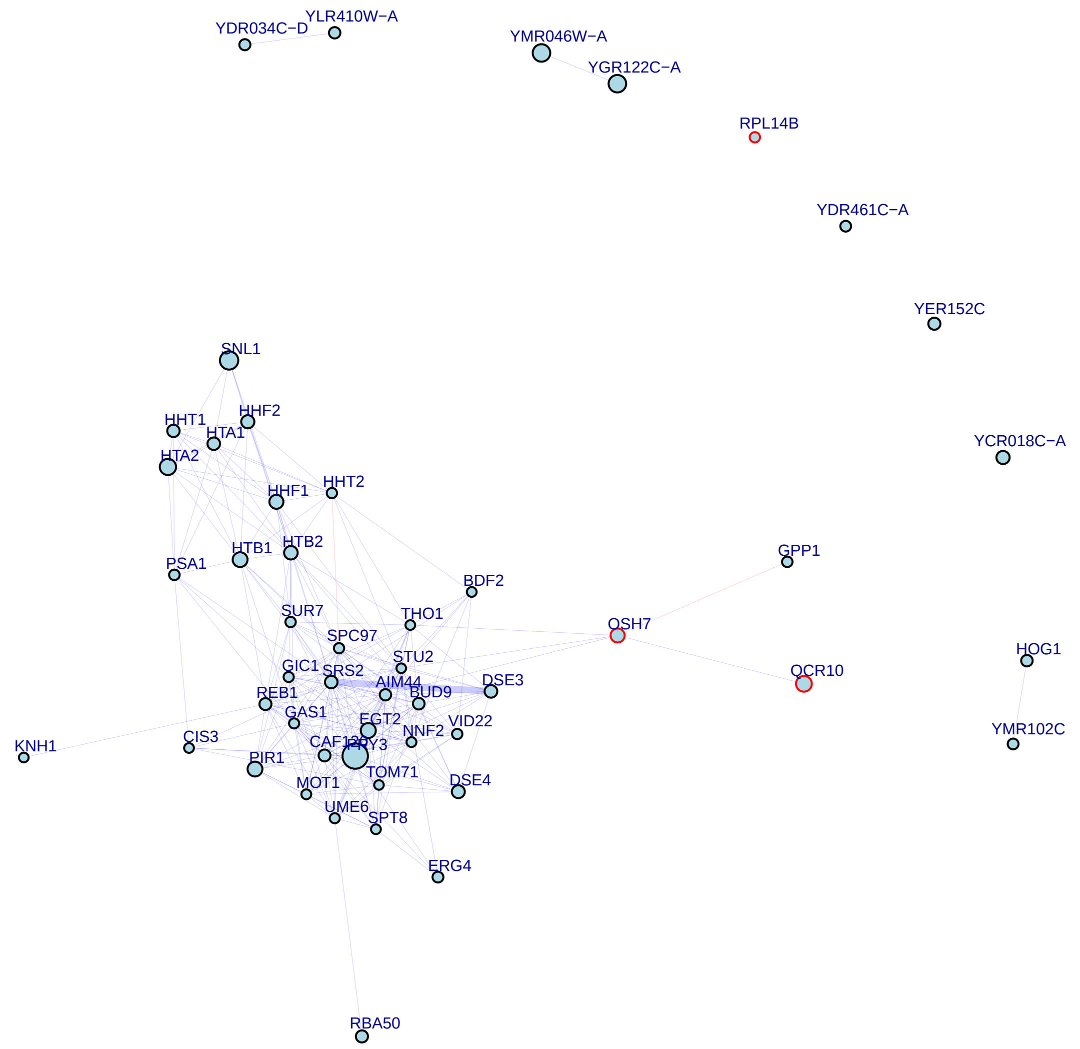

chrVIII:104423_C/A top 50 down genes

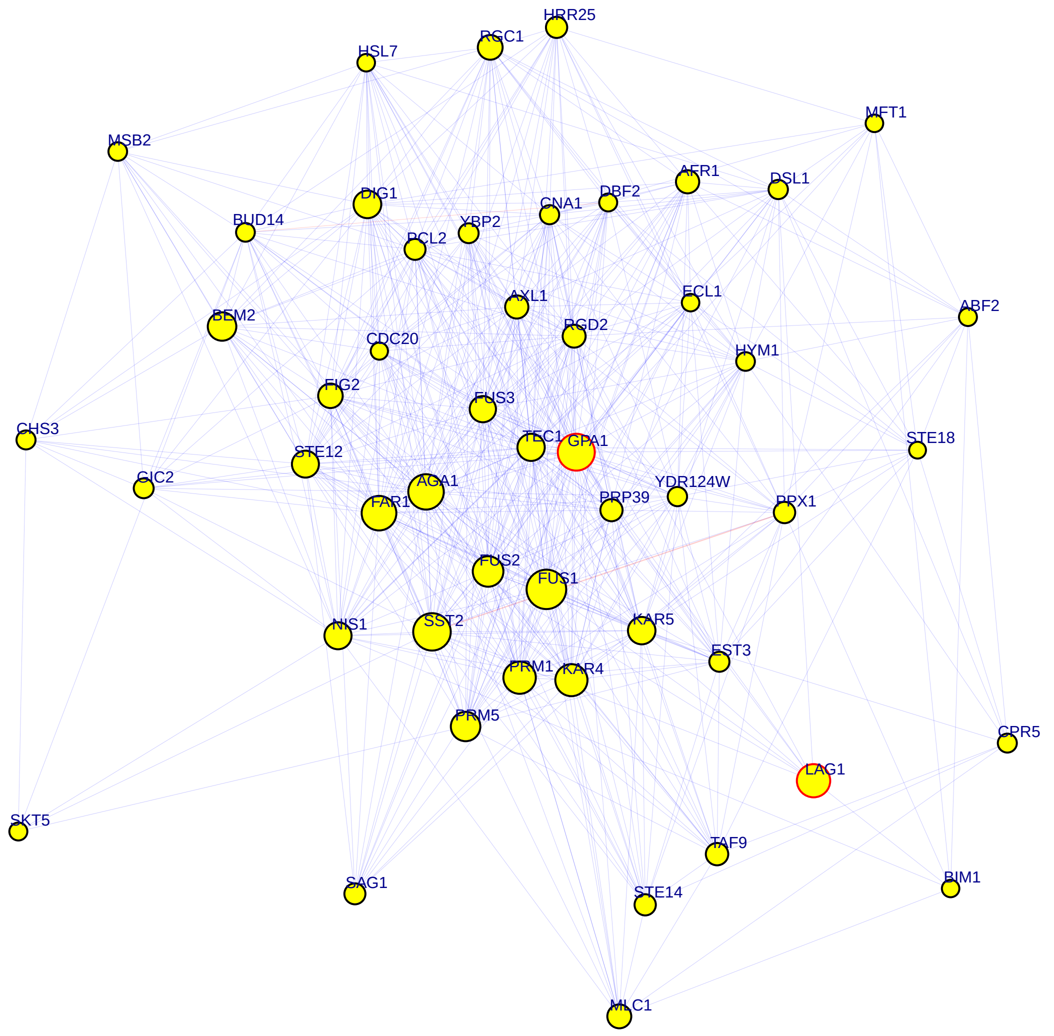

chrVIII:104423_C/A top 100 up and down genes

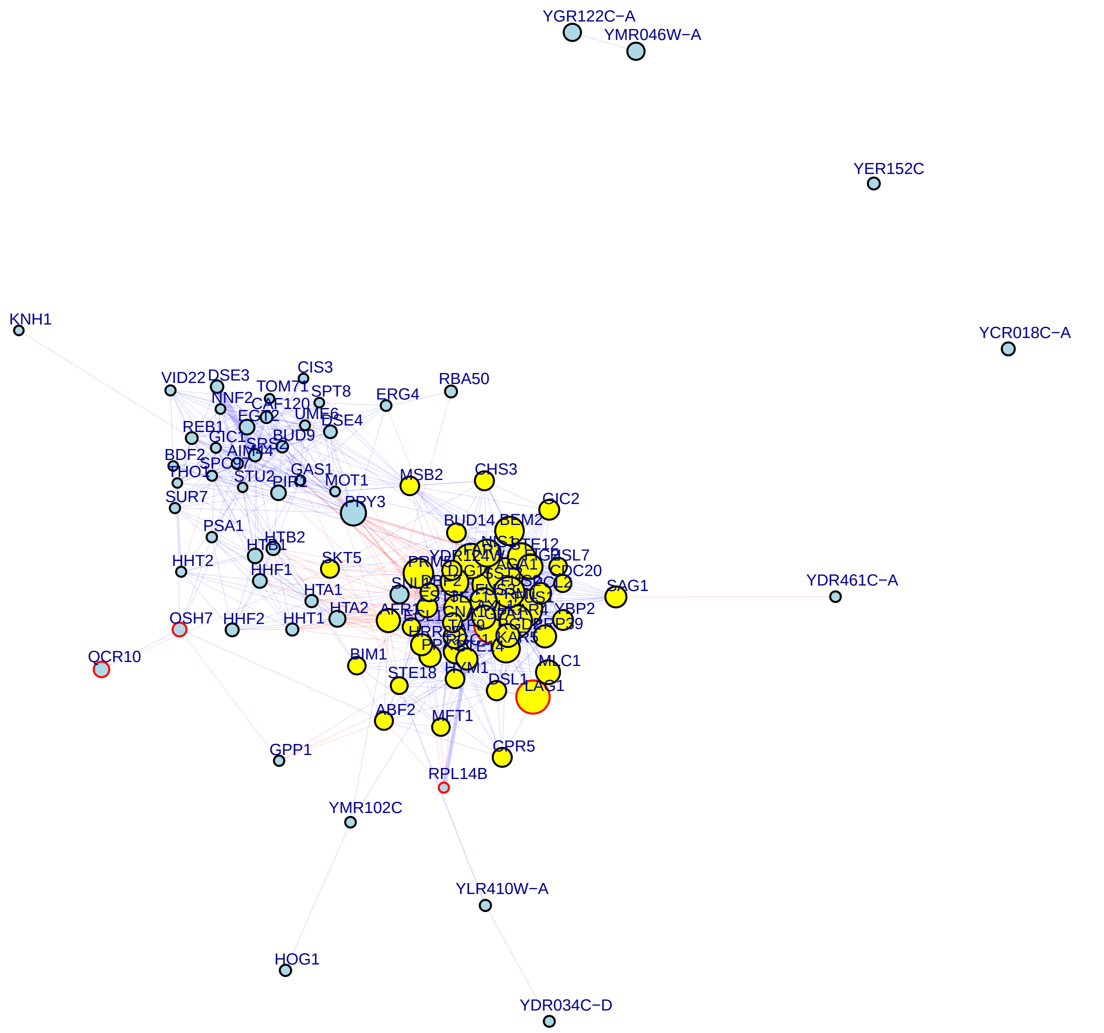

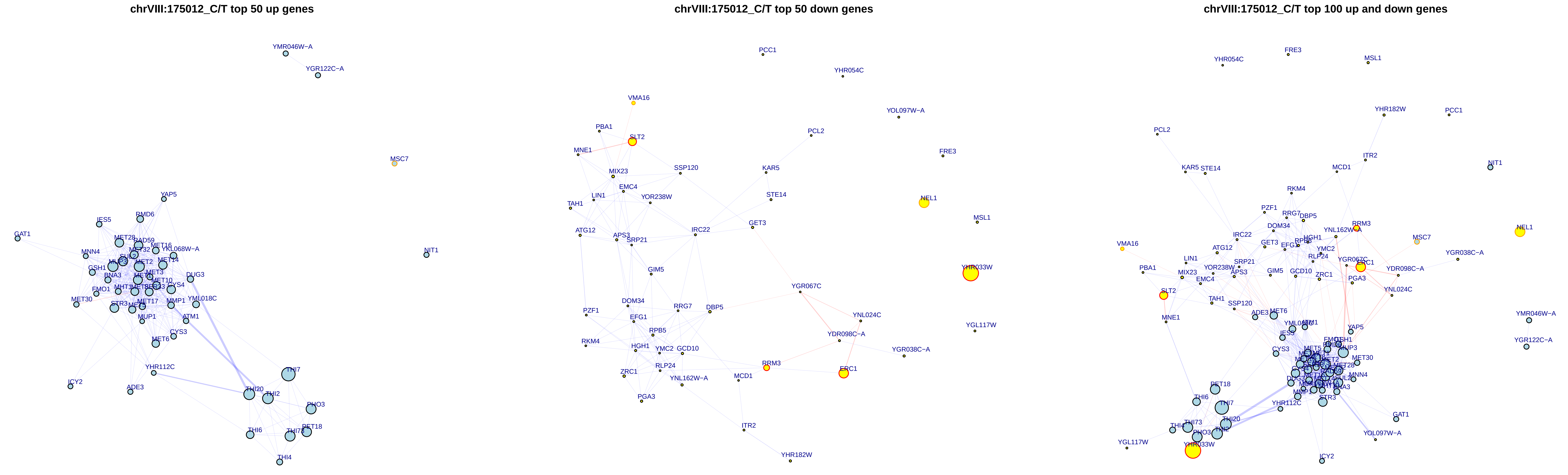
chrVIII:175012_C/T top 50 up genes
YMR046W−A
YGR122C−A
MSC7
NIT1
YAP5
IES5
RMD6
GAT1
MET28
RAD59
MET16
YKL068W−A
MNN4
MET32
MET14
GSH1
MUP3
MET2
MET3
DUG3
BNA3
MET10
CYS4
FMO1
MHT1
MET30
YML018C
STR3
MET17
MMP1
MUP1
ATM1
CYS3
MET6
YHR112C
THI7
ICY2
ADE3
THI20
THI2
PHO3
THI6
THI73
PET18
THI4
chrVIII:175012_C/T top 50 down genes
PCC1
YHR054C
VMA16
YOL097W−A
PBA1
SLT2
PCL2
MNE1
FRE3
SSP120
KAR5
MIX23
EMC4
STE14
LIN1
YOR238W
NEL1
TAH1
MSL1
ATG12
IRC22
GET3
APS3
SRP21
YHR033W
GIM5
YGR067C
DOM34
RRG7
DBP5
YNL024C
PZF1
EFG1
YGL117W
RPB5
YDR098C−A
RKM4
HGH1
YMC2
GCD10
YGR038C−A
RRM3
ERC1
ZRC1
RLP24
MCD1
YNL162W−A
PGA3
ITR2
YHR182W
chrVIII:175012_C/T top 100 up and down genes
FRE3
YHR054C
MSL1
YHR182W
PCC1
PCL2
ITR2
NIT1
KAR5
STE14
MCD1
RKM4
PZF1
RRG7
DBP5
DOM34
RRM3
YNL162W−A
NEL1
IRC22
GET3
HGH1
MSC7
VMA16
ATG12
YMC2
YGR038C−A
RLP24
LIN1
YOR238W
SRP21
YGR067C
ERC1
YDR098C−A
PBA1
MIX23
APS3
GIM5
GCD10
ZRC1
EMC4
PGA3
YNL024C
SLT2
TAH1
SSP120
ADE3
MET6
MNE1
YAP5
IES5
CYS3
MET30
MNN4
PET18
THI6
THI7
YHR112C
STR3
THI20
THI73
THI4
PHO3
GAT1
YGL117W
YHR033W
YOL097W−A
ICY2
YMR046W−A
YGR122C−A

chrVIII:459310_C/G top 50 up genes

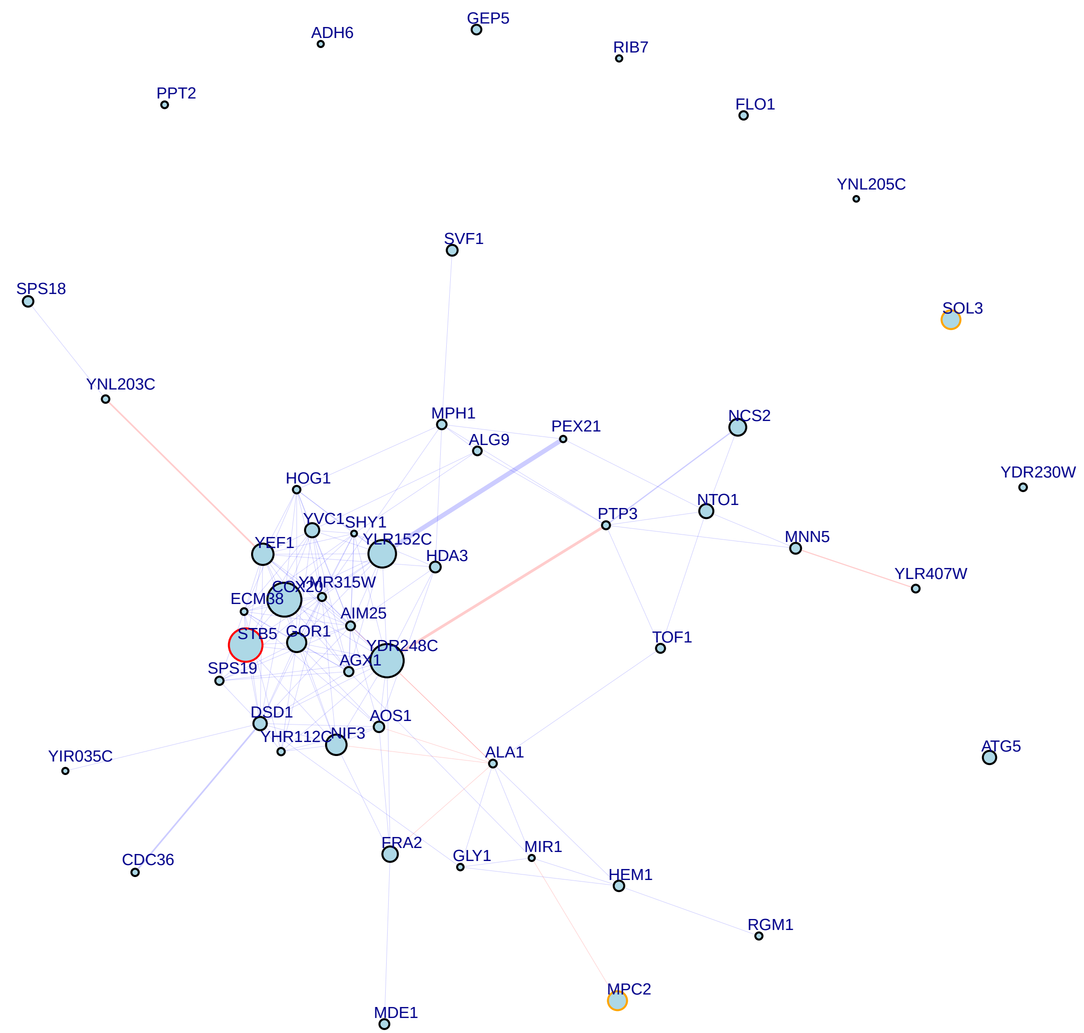

chrVIII:459310_C/G top 50 down genes

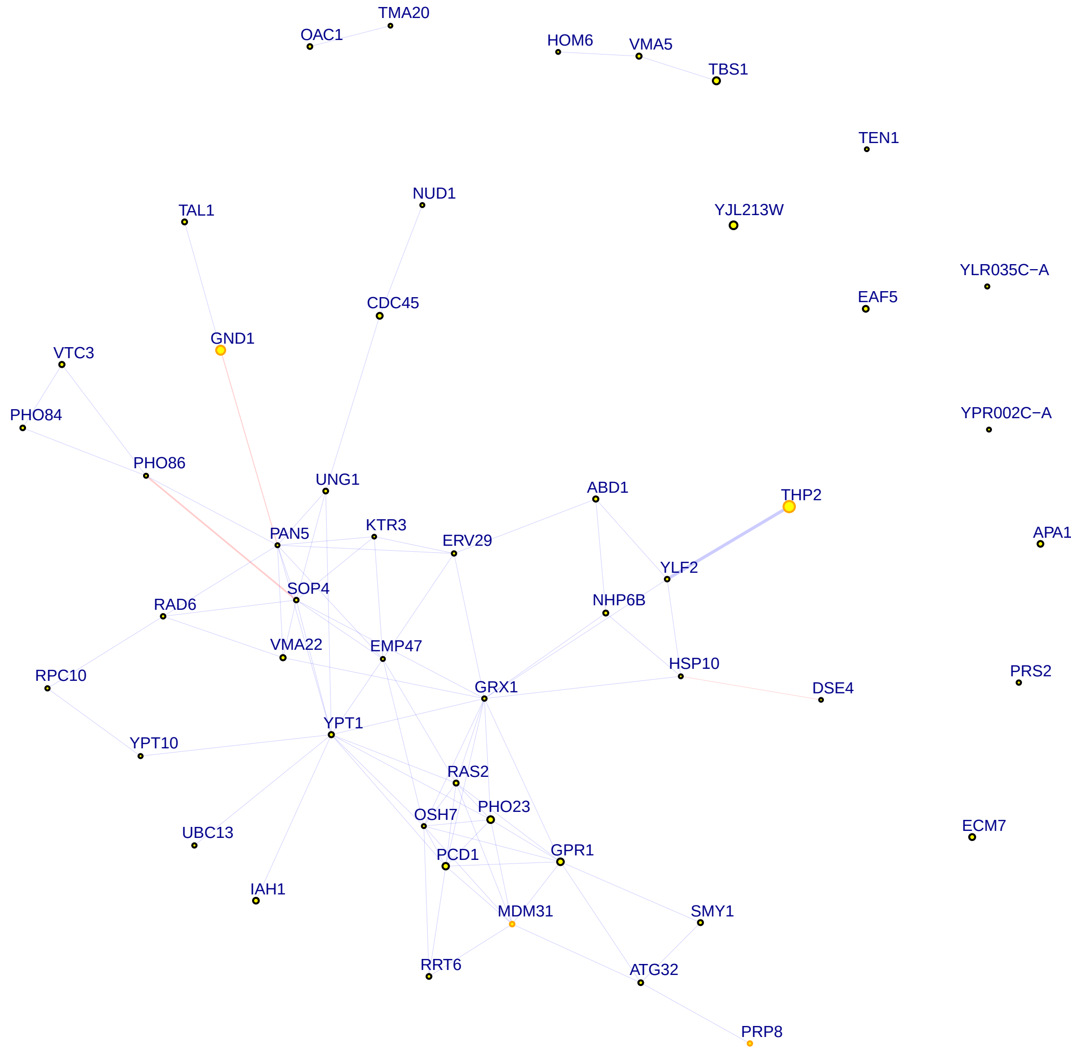

chrVIII:459310_C/G top 100 up and down genes

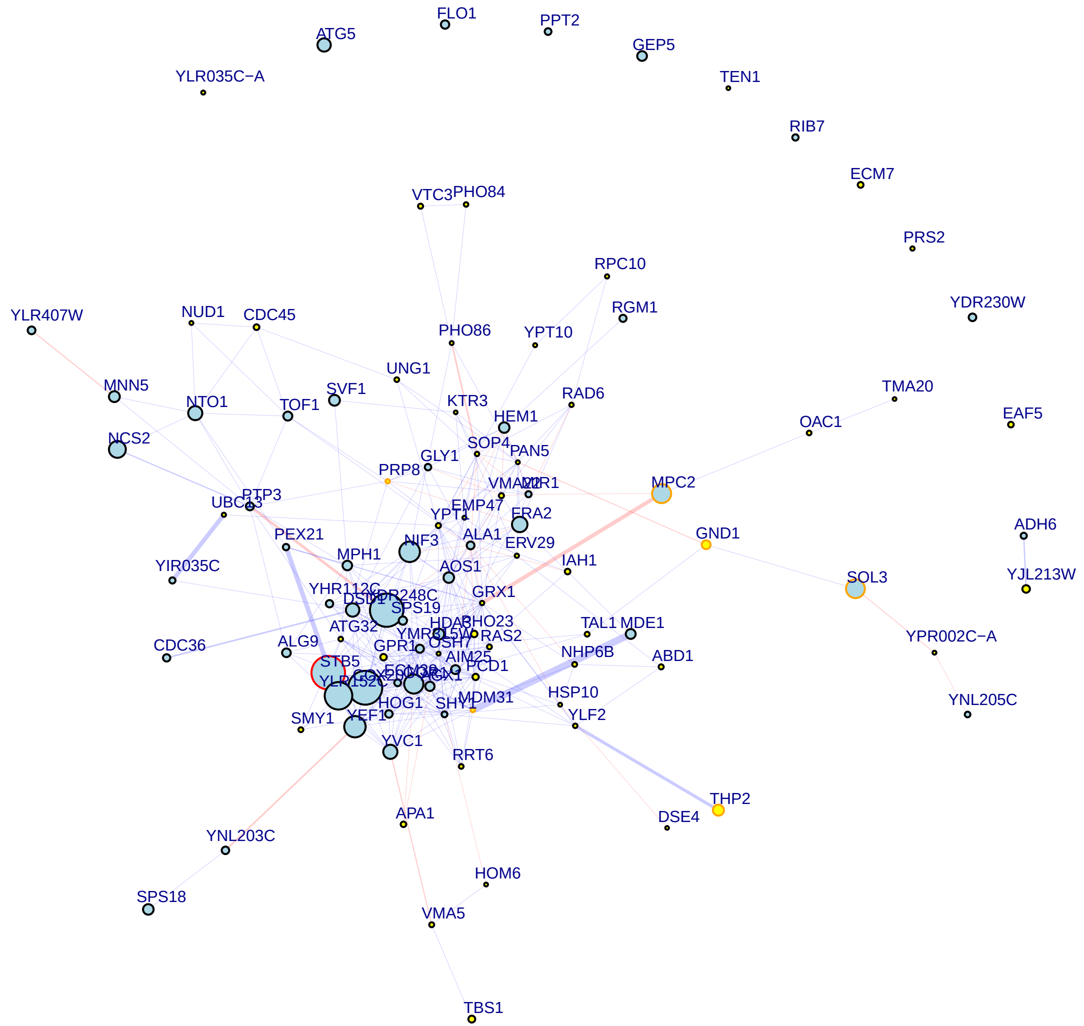

chrIX:28874_G/C top 35 up genes

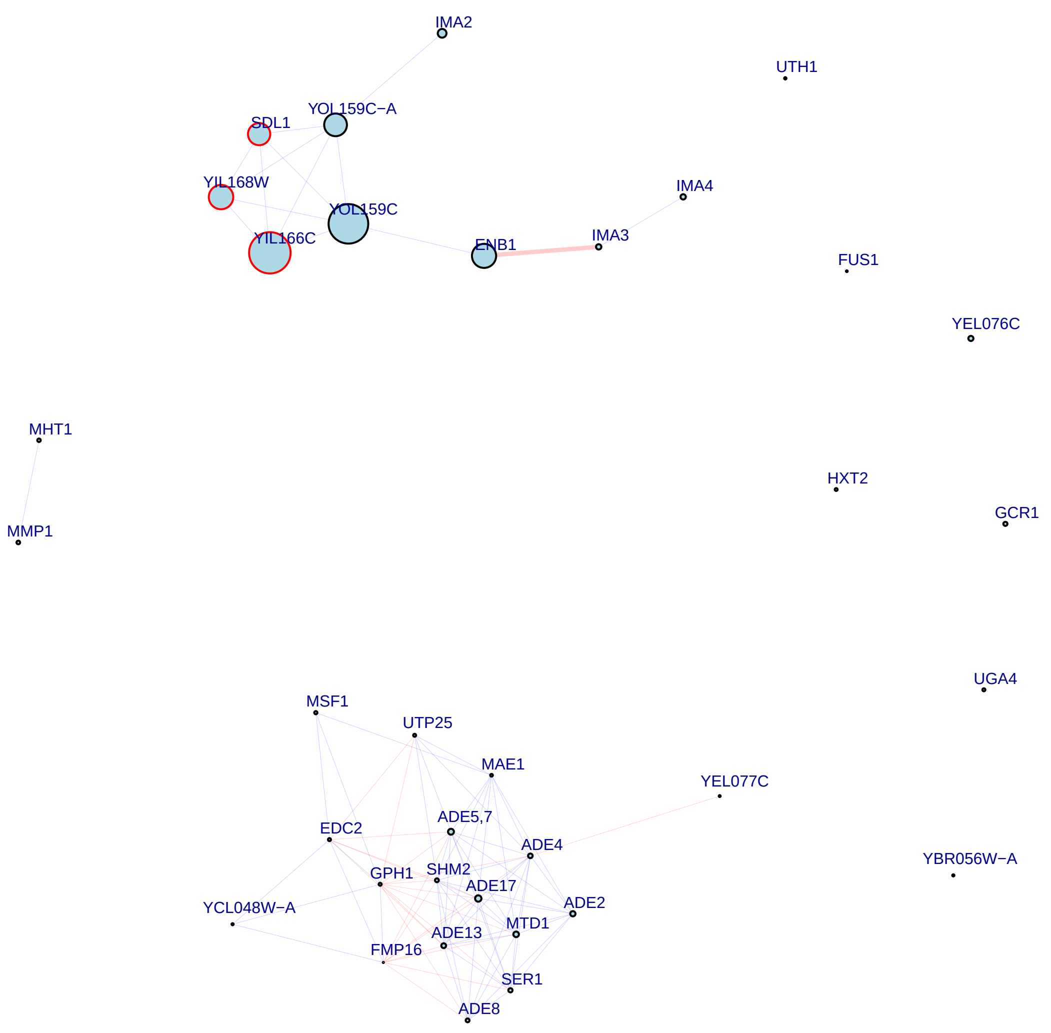

chrIX:28874_G/C top 31 down genes

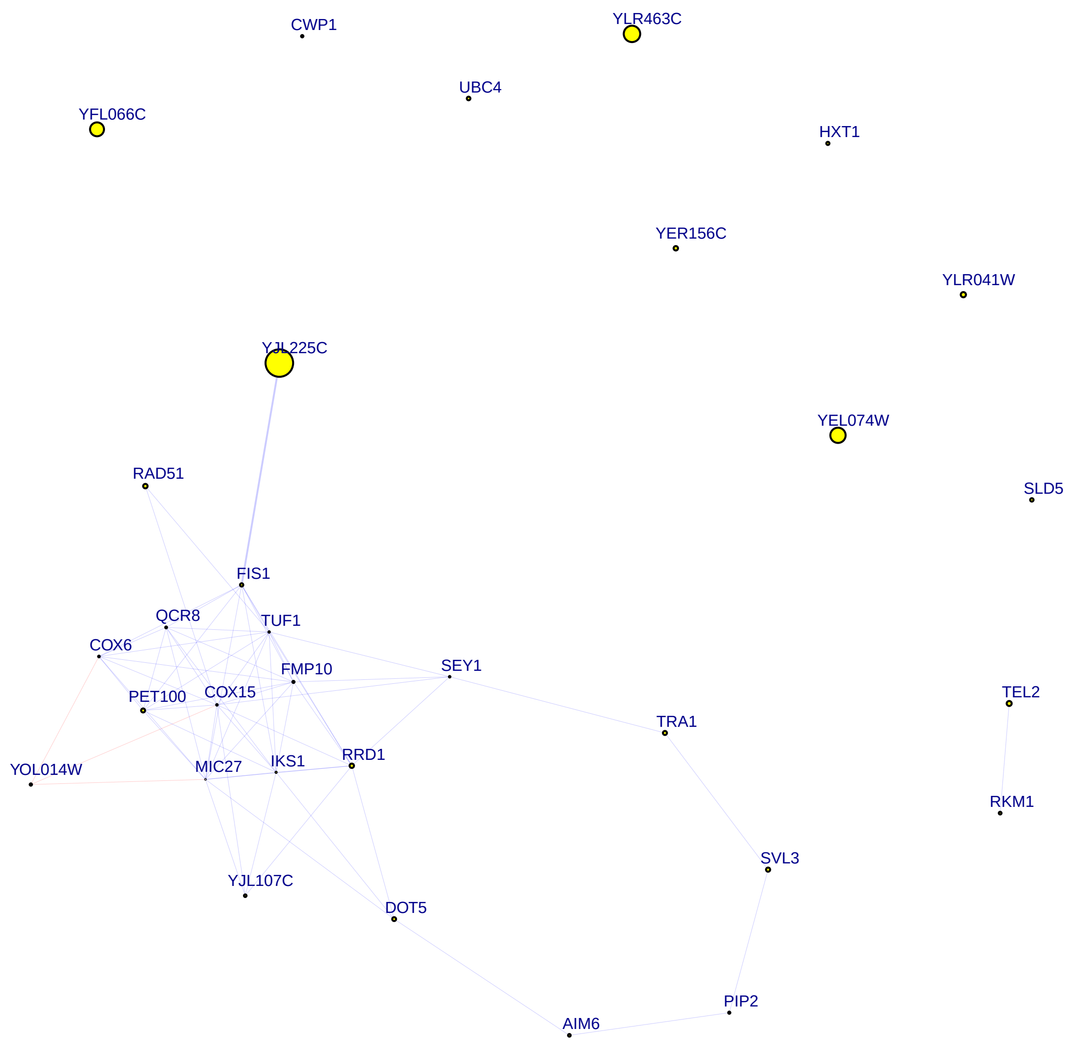

chrIX:28874_G/C top 66 up and down genes

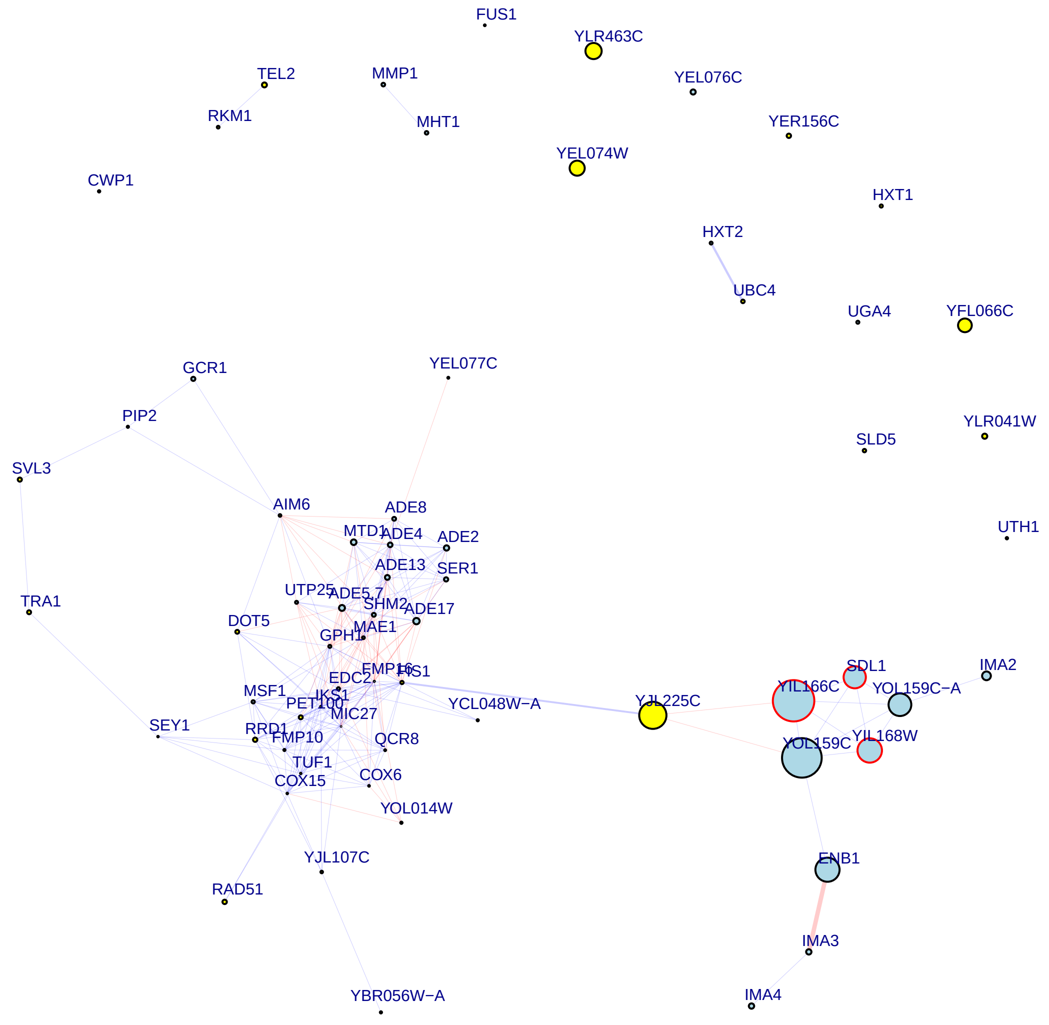

chrIX:105090_T/C top 50 up genes

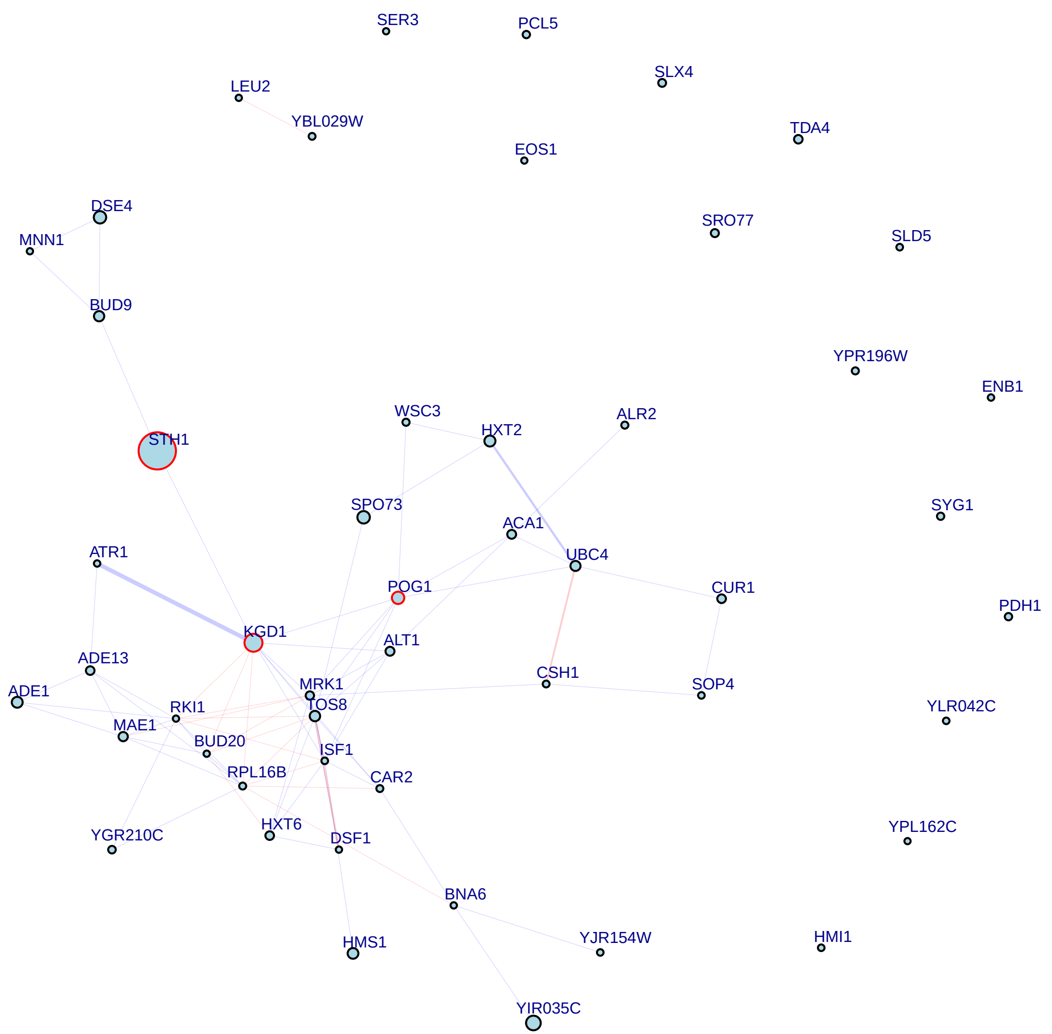

chrIX:105090_T/C top 50 down genes

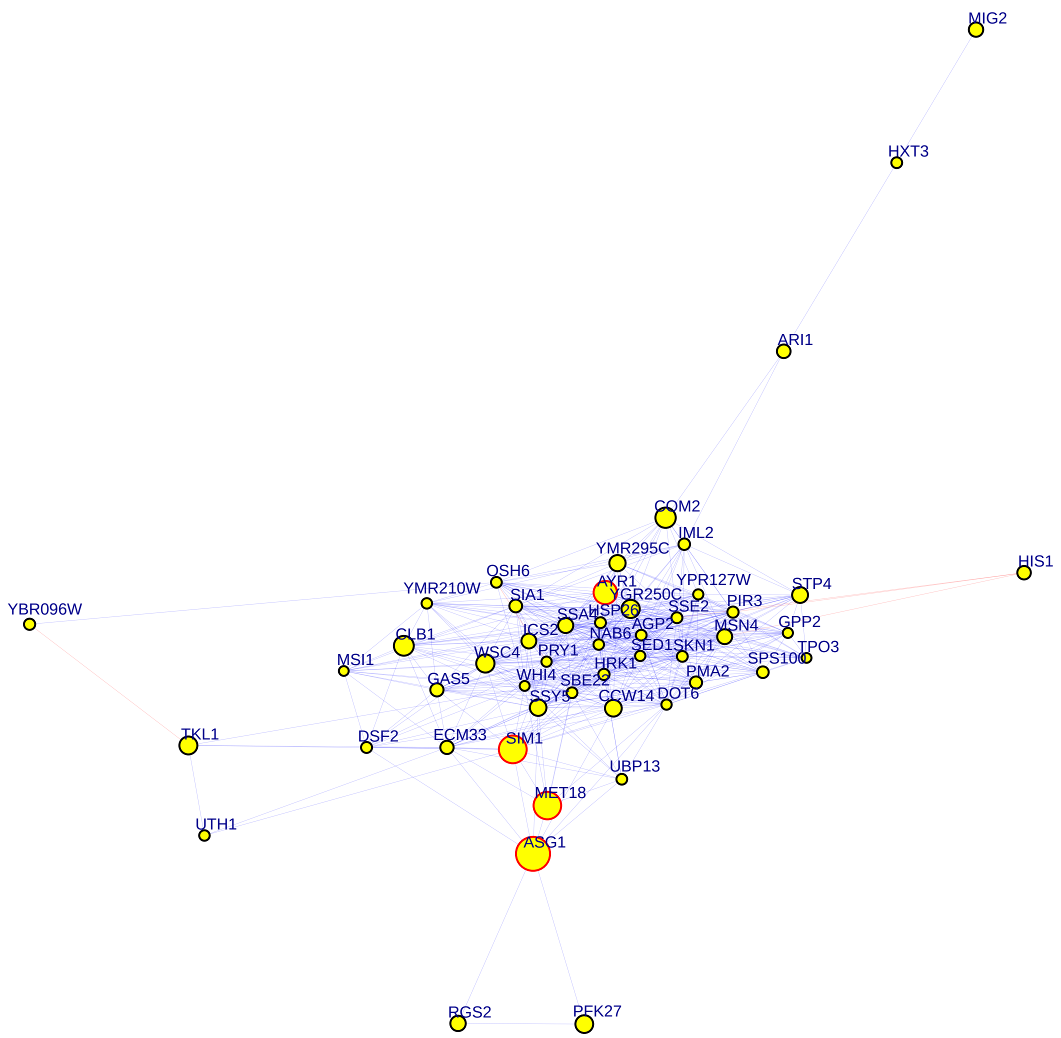

chrIX:105090_T/C top 100 up and down genes

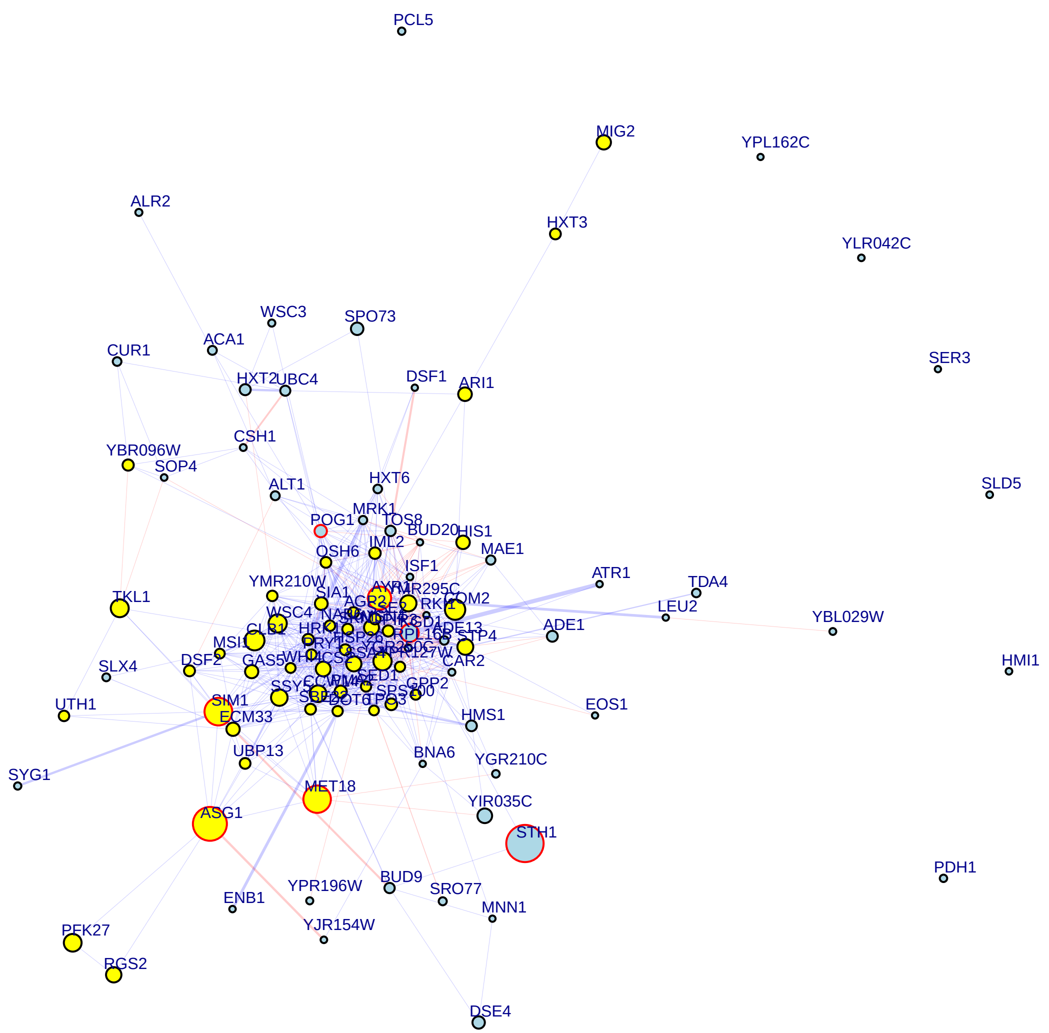

chrIX:178160_C/T top 50 up genes

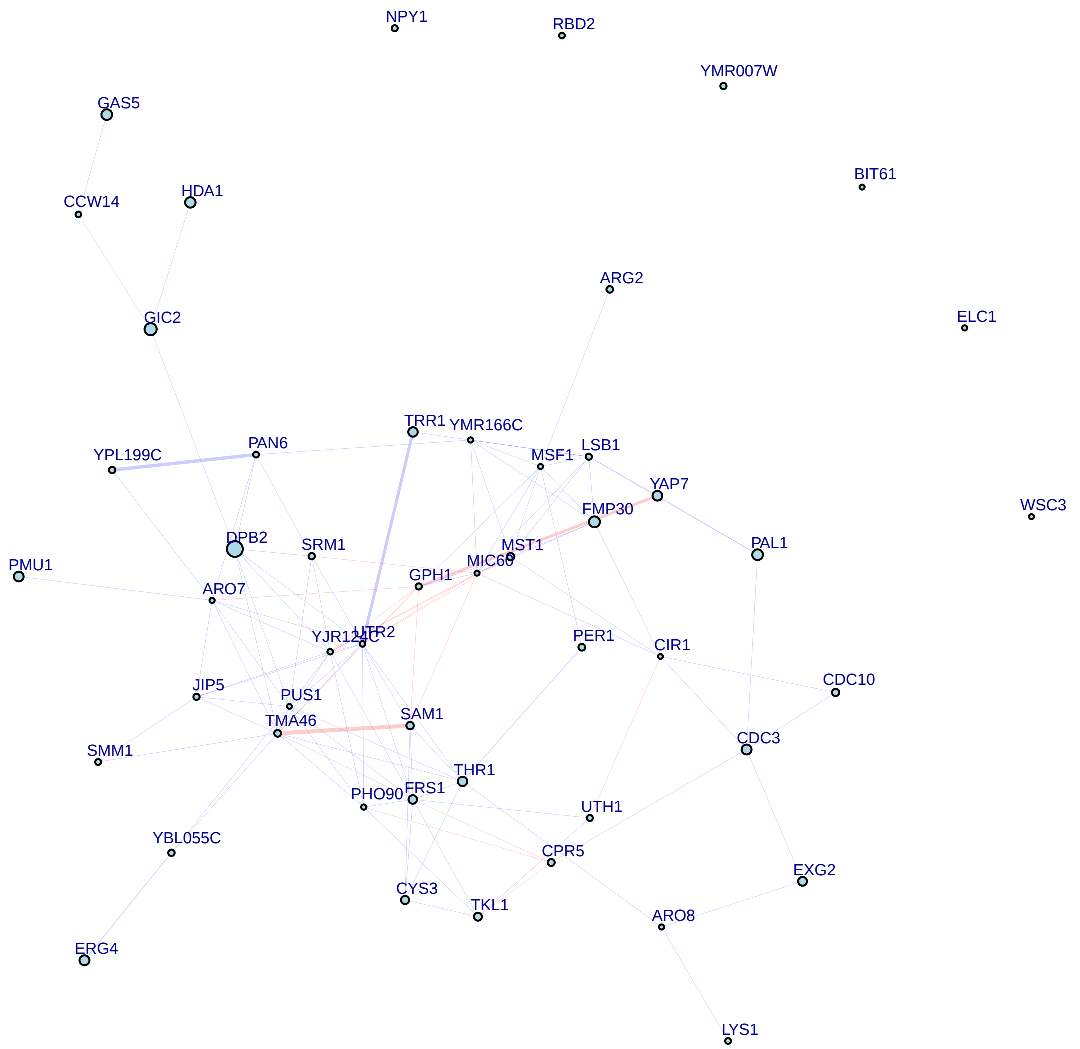

chrIX:178160_C/T top 50 down genes

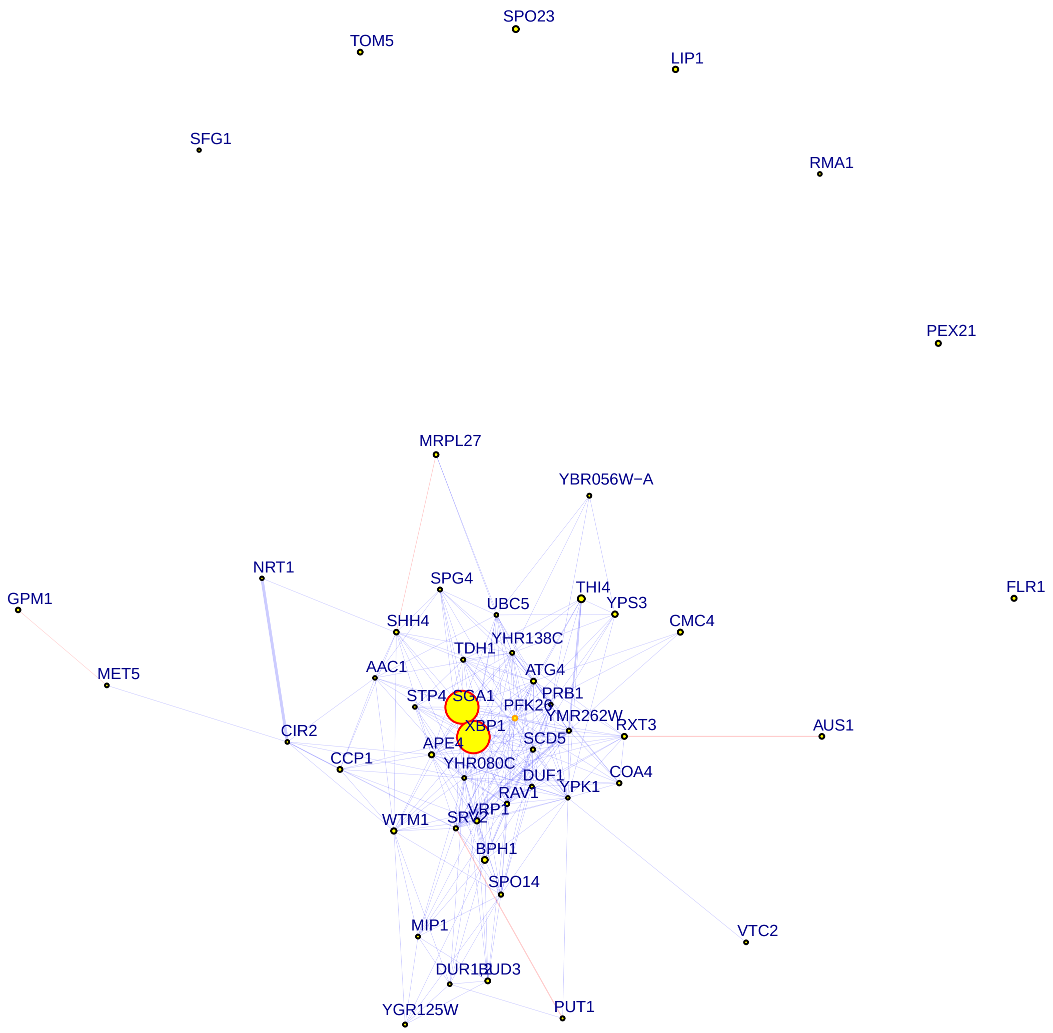

chrIX:178160_C/T top 100 up and down genes

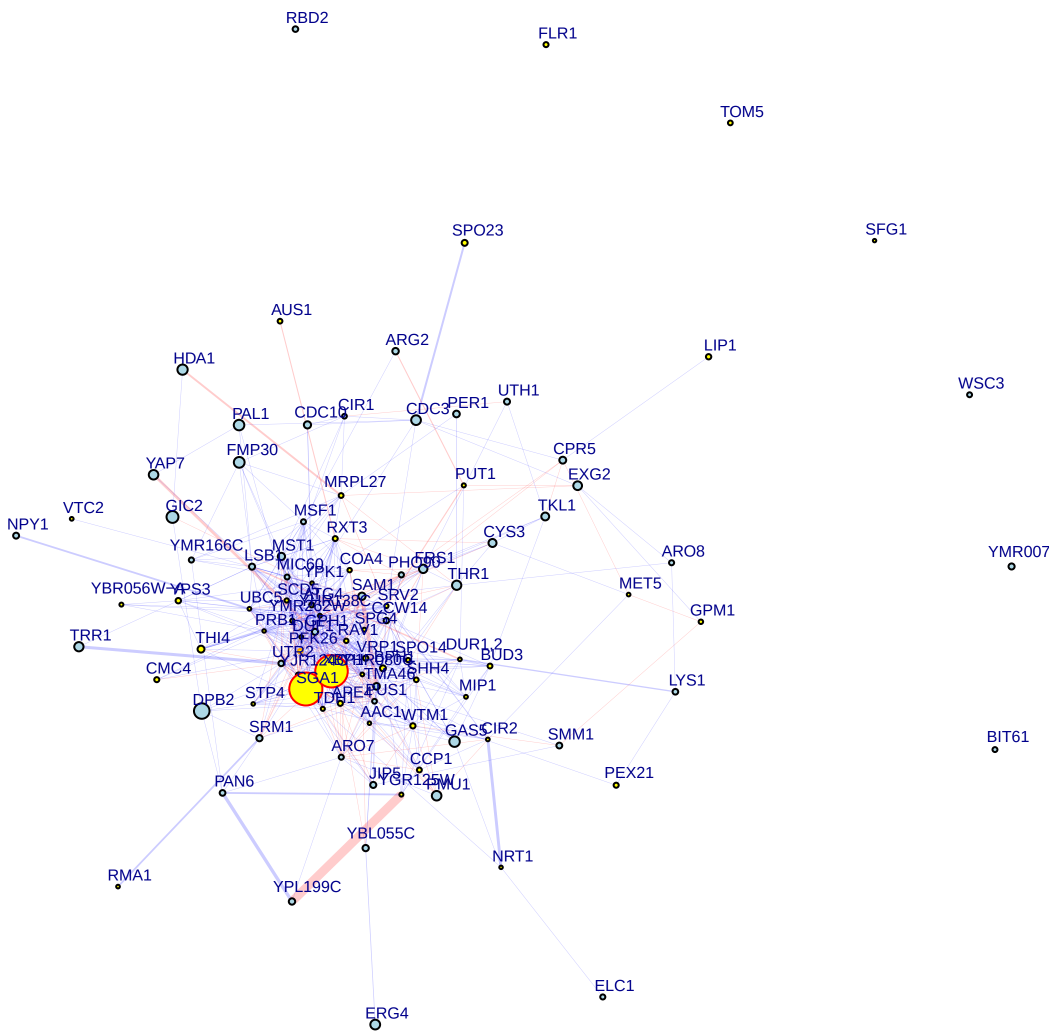

chrIX:227301_A/T top 37 up genes

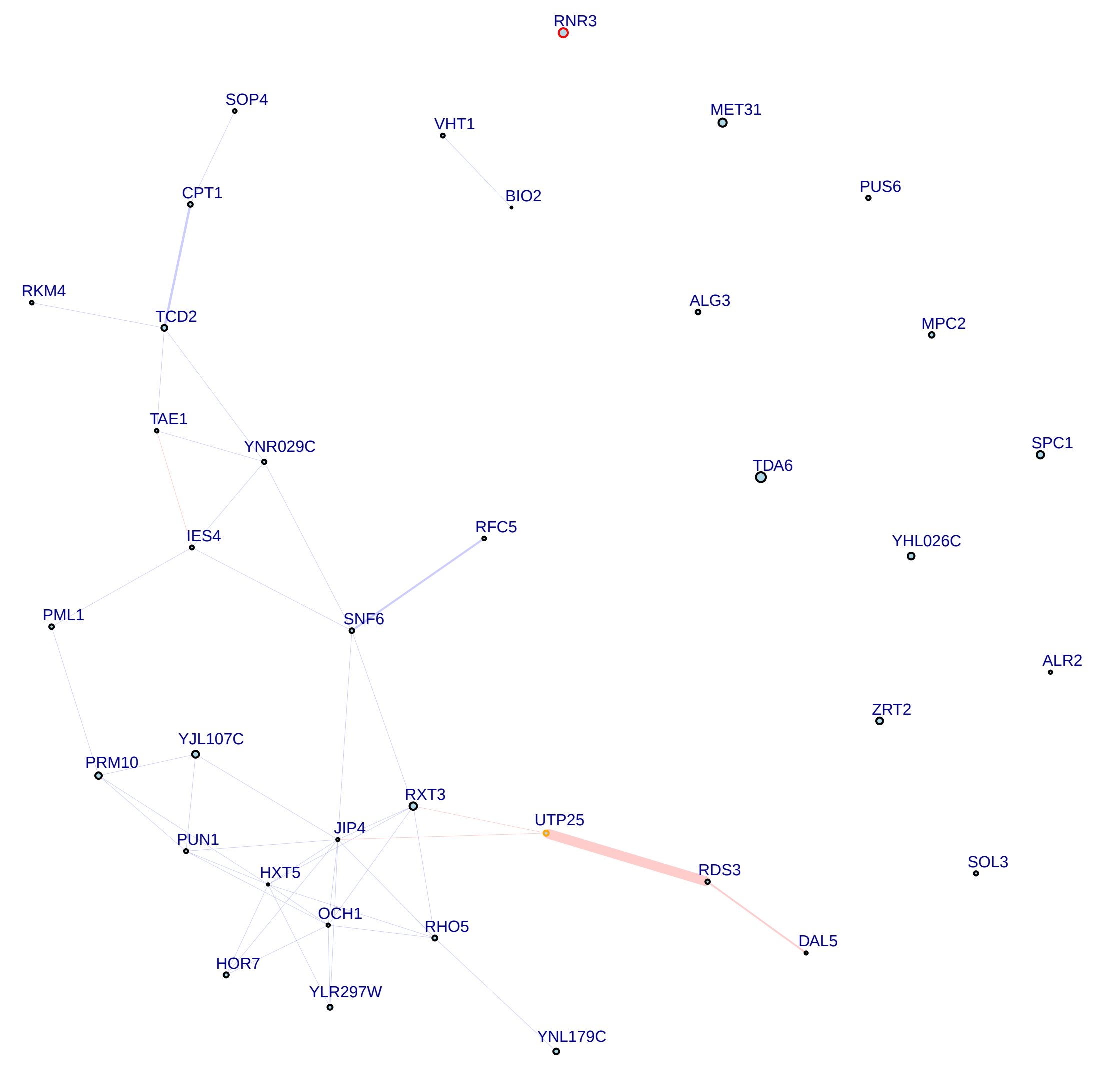

chrIX:227301_A/T top 50 down genes

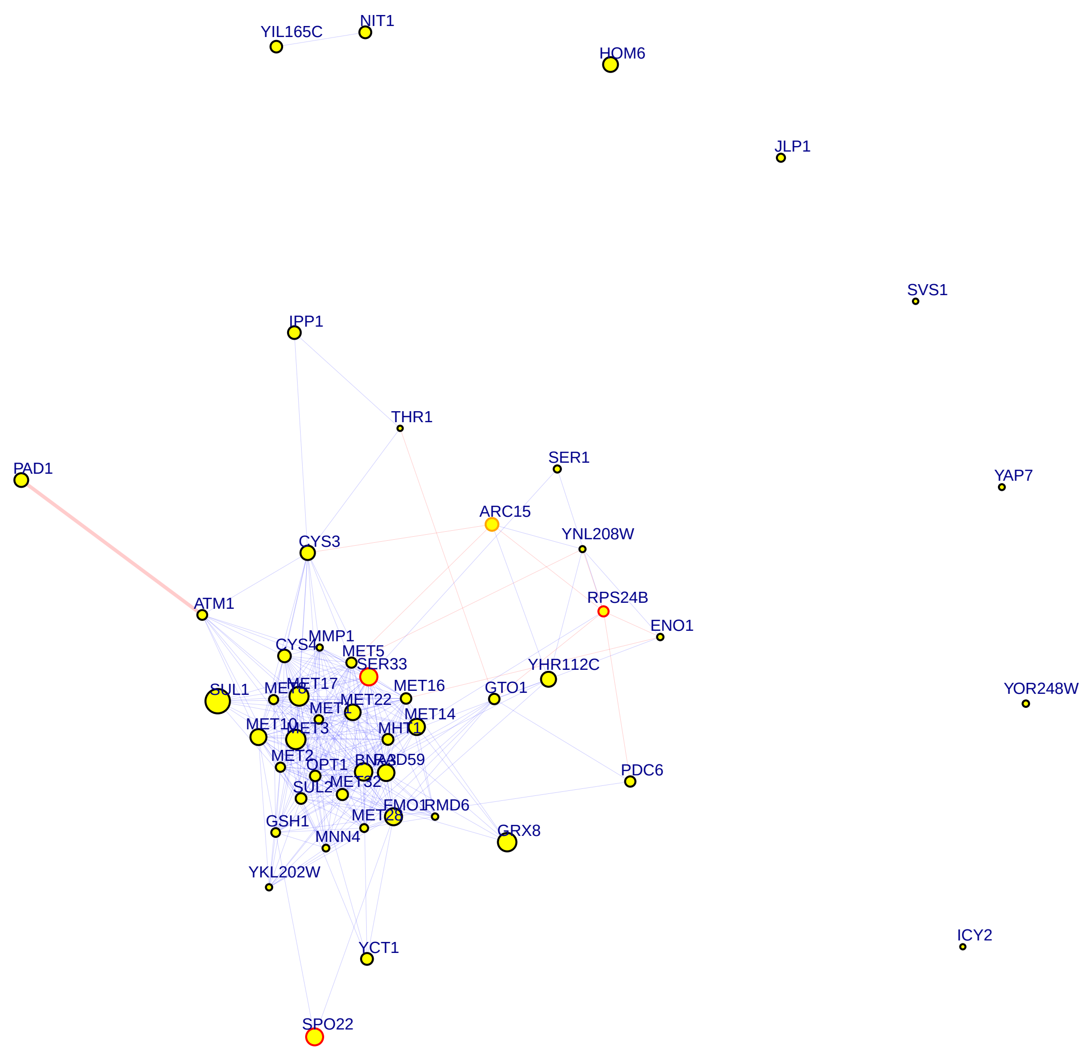

chrIX:227301_A/T top 87 up and down genes

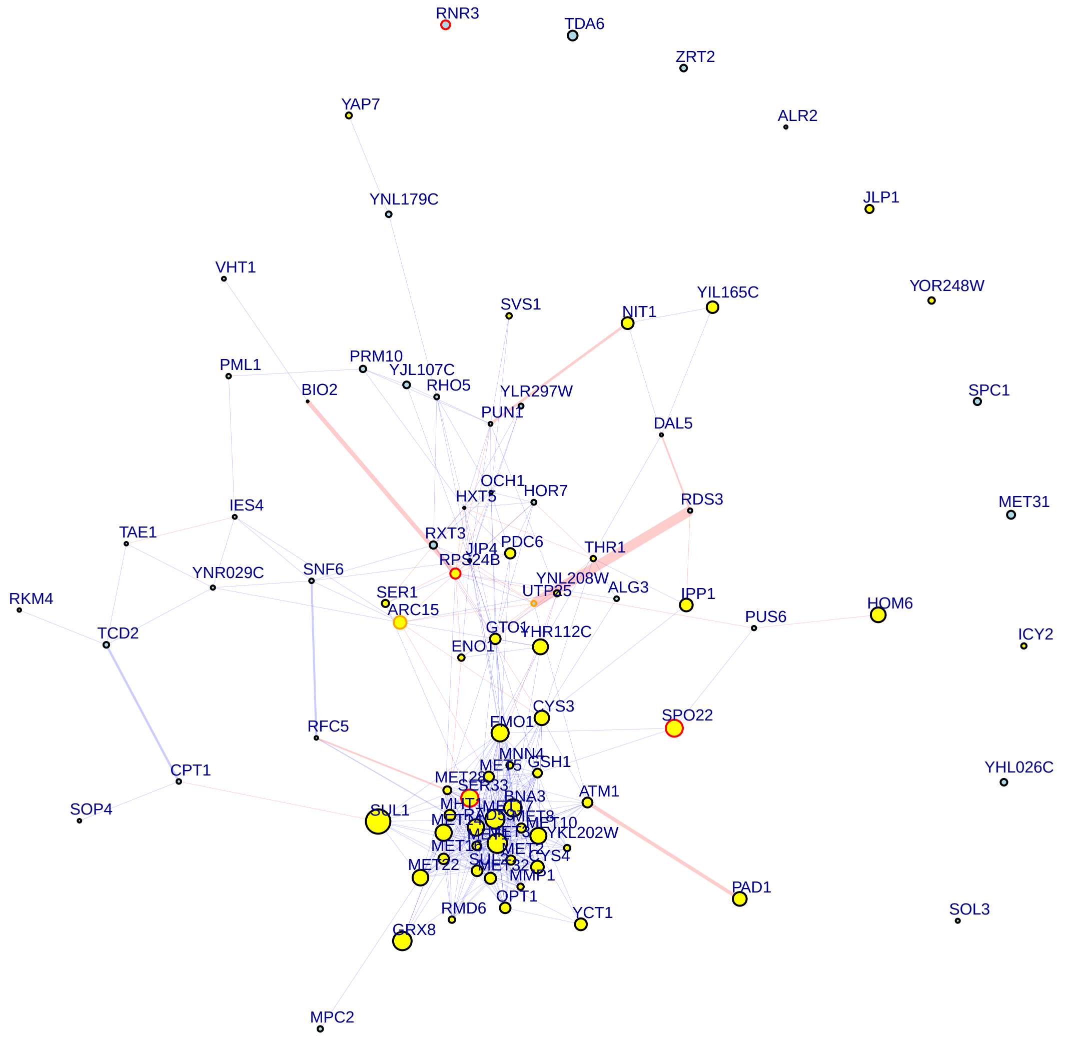

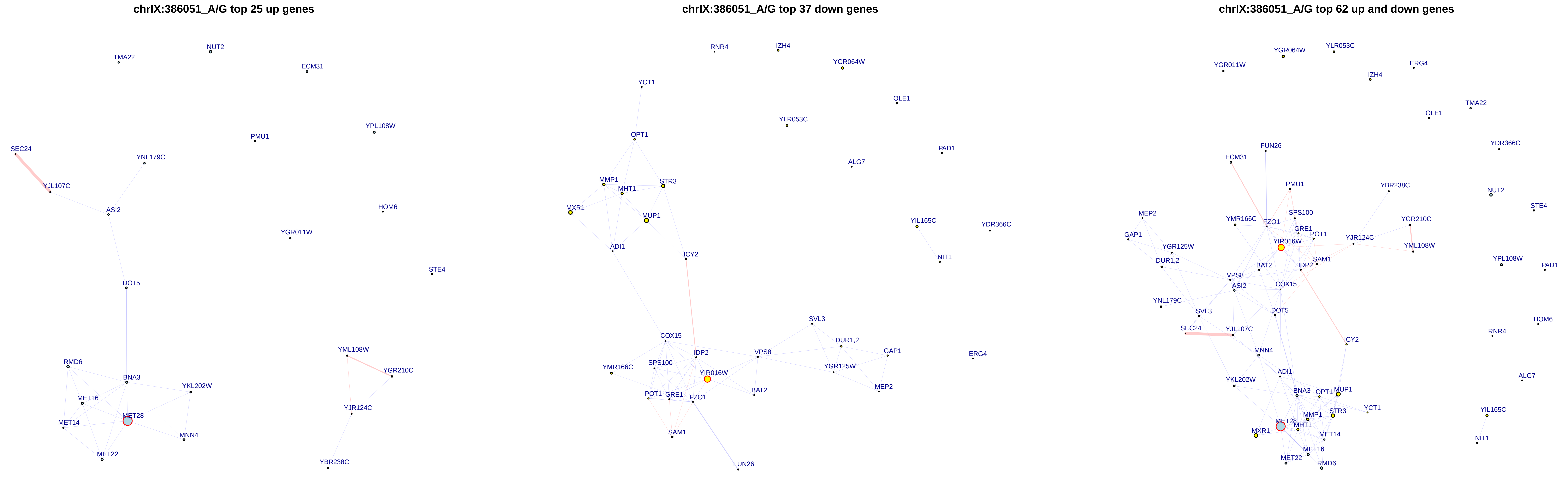
chrIX:386051_A/G top 25 up genes
TMA22
NUT2
ECM31
PMU1
YPL108W
SEC24
YNL179C
YJL107C
ASI2
HOM6
YGR011W
STE4
DOT5
YML108W
YGR210C
RMD6
BNA3
YKL202W
MET16
YJR124C
MET28
MET14
MNN4
MET22
YBR238C
chrIX:386051_A/G top 37 down genes
RNR4
IZH4
YGR064W
YCT1
QLE1
YLR053C
OPT1
PAD1
ALG7
MMP1
STR3
MHT1
MXR1
MUP1
YIL165C
YDR366C
ADI1
ICY2
NIT1
SVL3
COX15
DUR1,2
IDP2
VPS8
GAP1
ERG4
YMR166C
SPS100
YIR016W
YGR125W
POT1
GRE1
FZO1
BAT2
MEP2
SAM1
FUN26
chrIX:386051_A/G top 62 up and down genes
YGR064W
YLR053C
YGR011W
ERG4
IZH4
TMA22
QLE1
YDR366C
FUN26
ECM31
PMU1
YBR238C
NUT2
STE4
MEP2
SPS100
YMR166C
FZO1
YGR210C
GAP1
GRE1
POT1
YJR124C
YIR016W
YGR125W
YML108W
YPL108W
PAD1
DUR1,2
SAM1
BAT2
IDP2
VPS8
COX15
ASI2
YNL179C
DOT5
SVL3
HOM6
SEC24
YJL107C
ICY2
RNR4
MNN4
ADI1
ALG7
YKL202W
BNA3
OPT1
MUP1
YCT1
YIL165C
MMP1
STR3
MET28
MHT1
MXR1
MET14
NIT1
MET16
MET22
RMD6

chrX:26108_C/T top 50 up genes

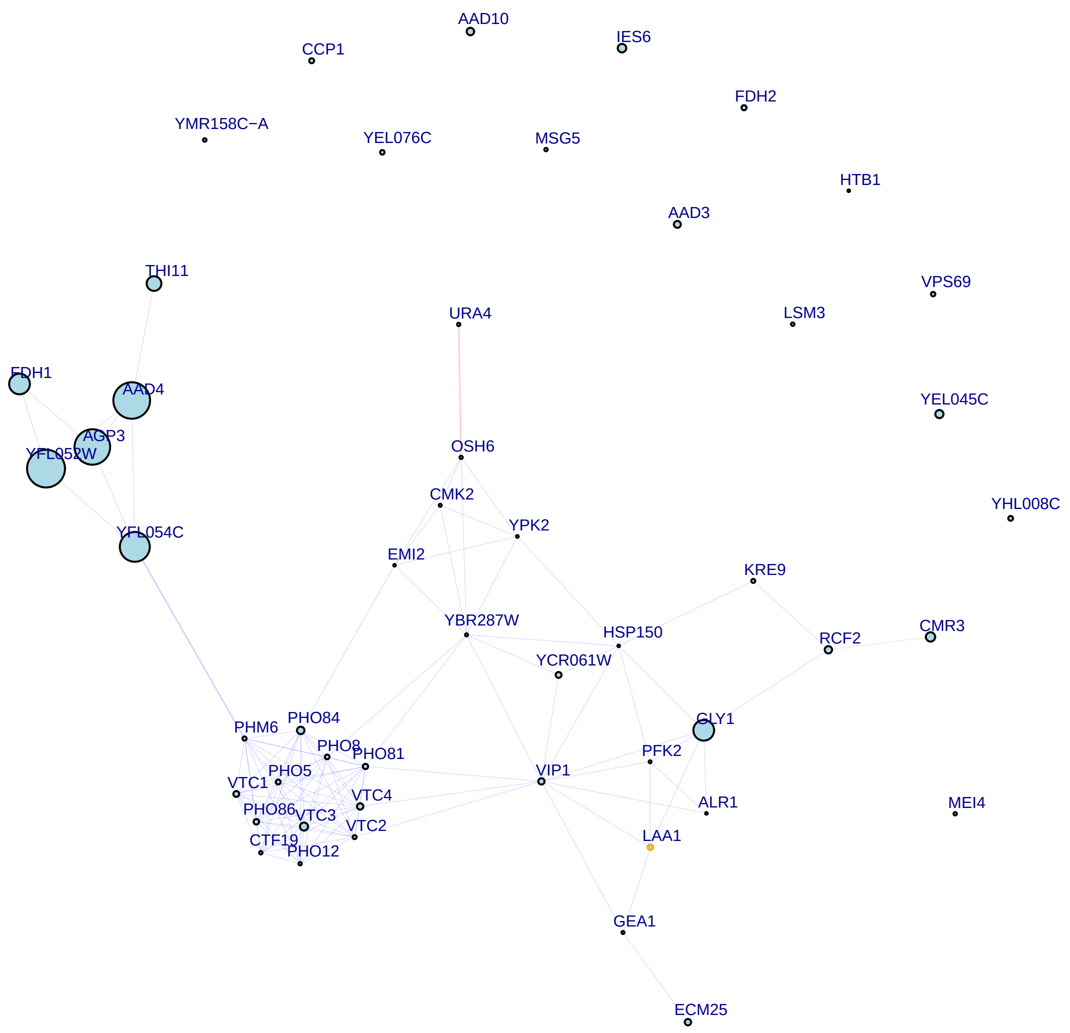

chrX:26108_C/T top 49 down genes

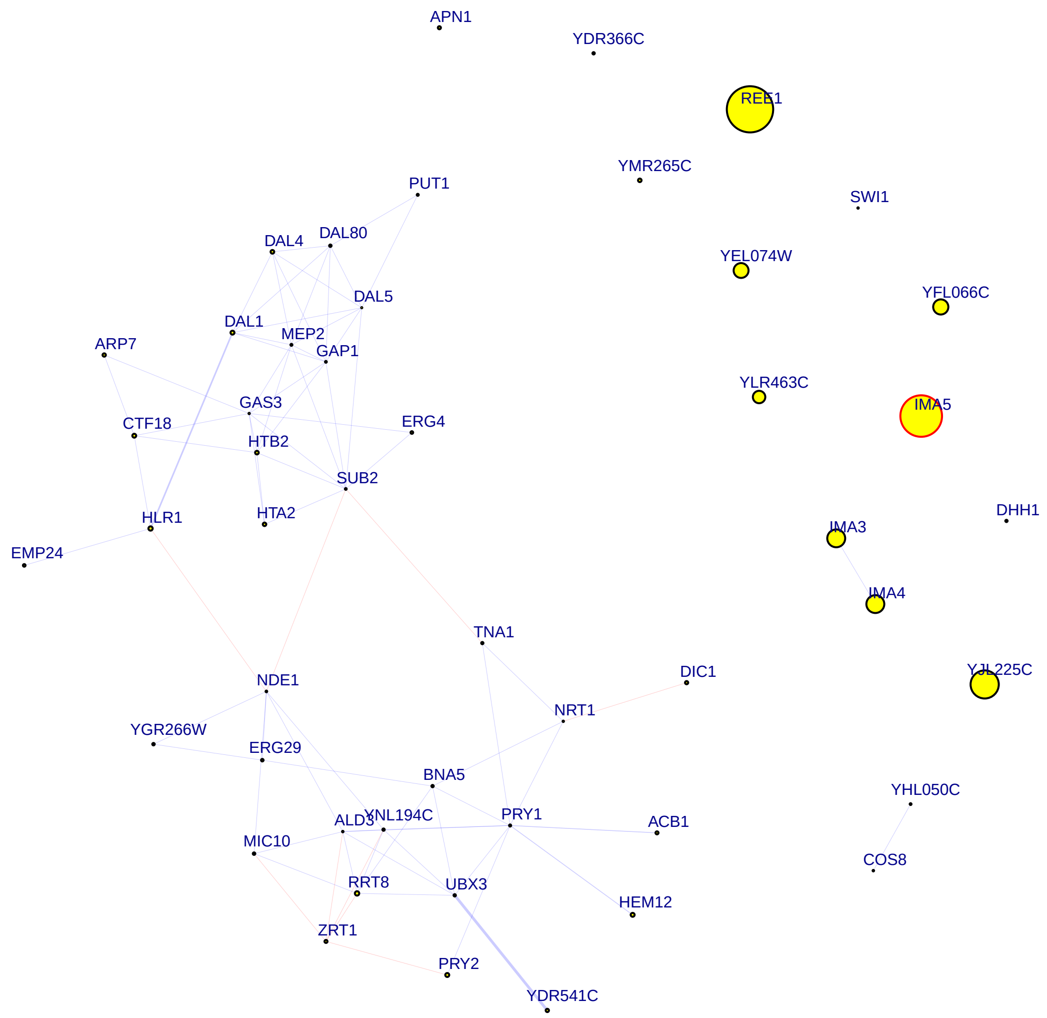

chrX:26108_C/T top 99 up and down genes

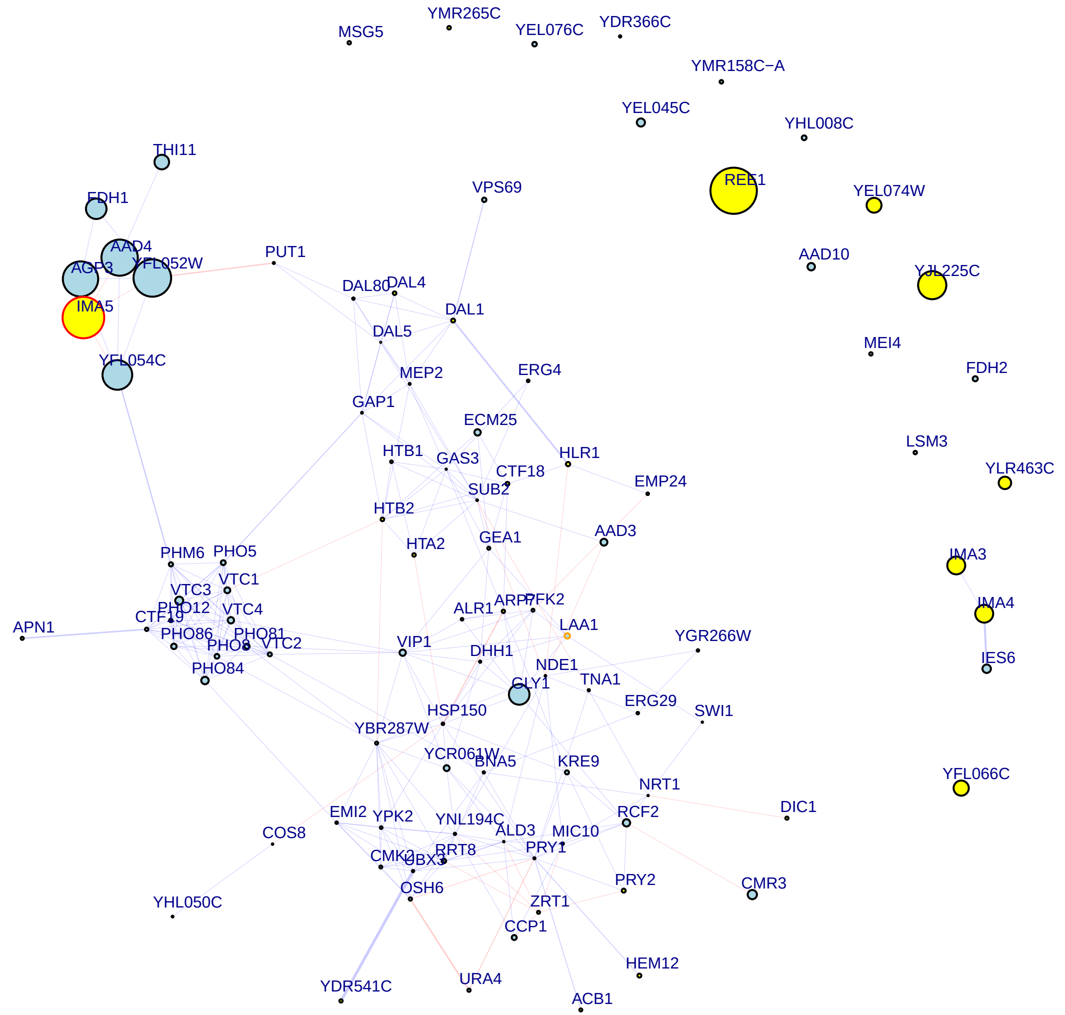

chrX:108904_A/G top 50 up genes

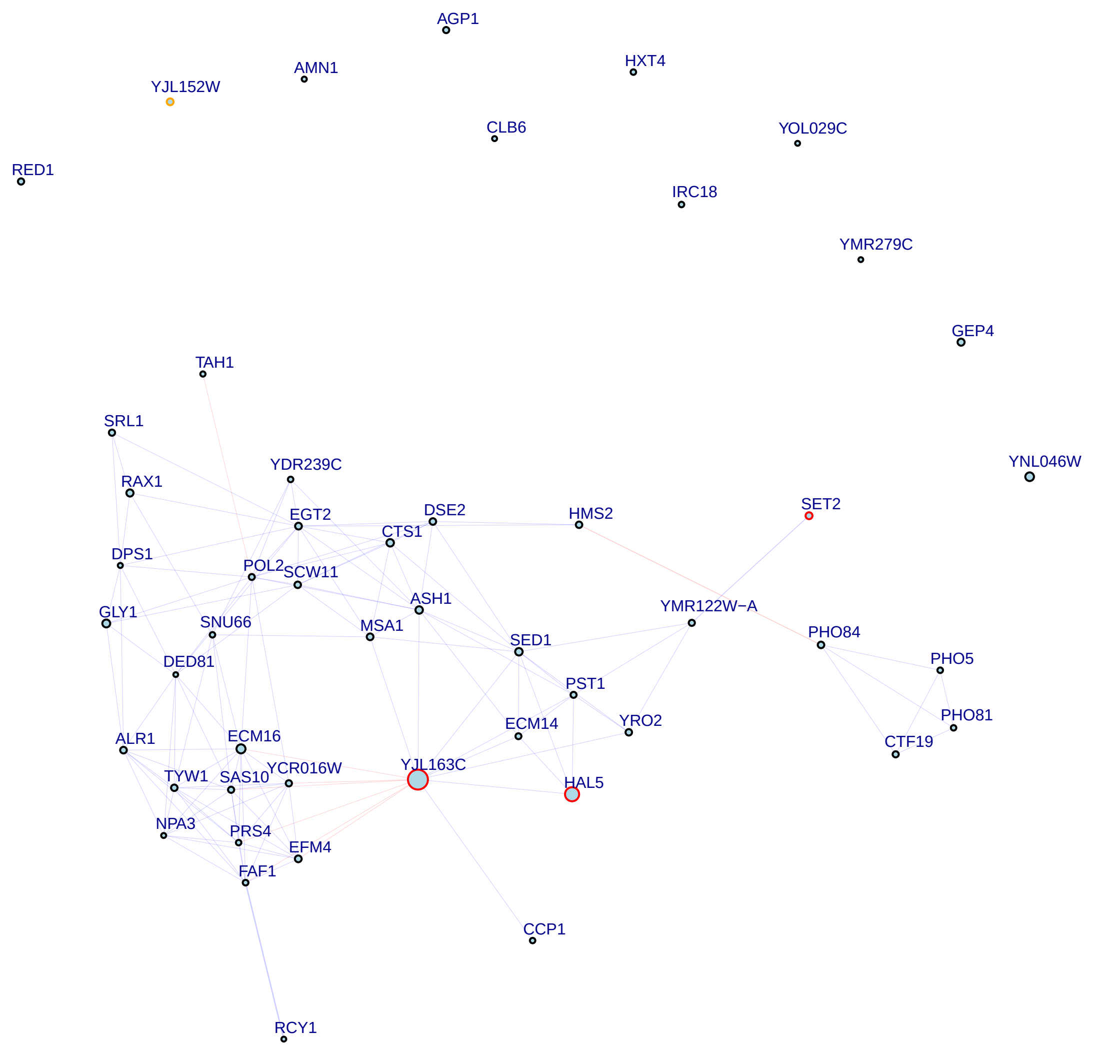

chrX:108904_A/G top 50 down genes

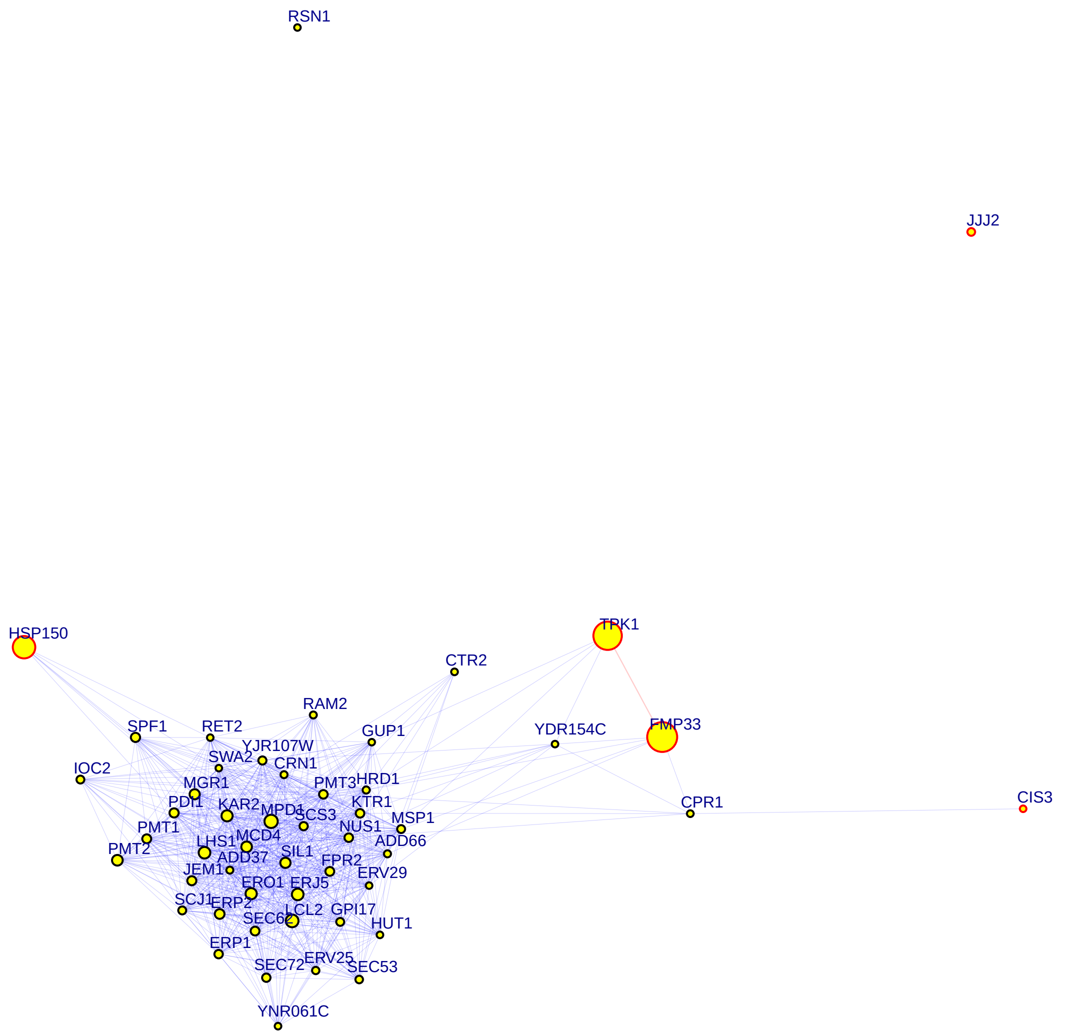

chrX:108904_A/G top 100 up and down genes

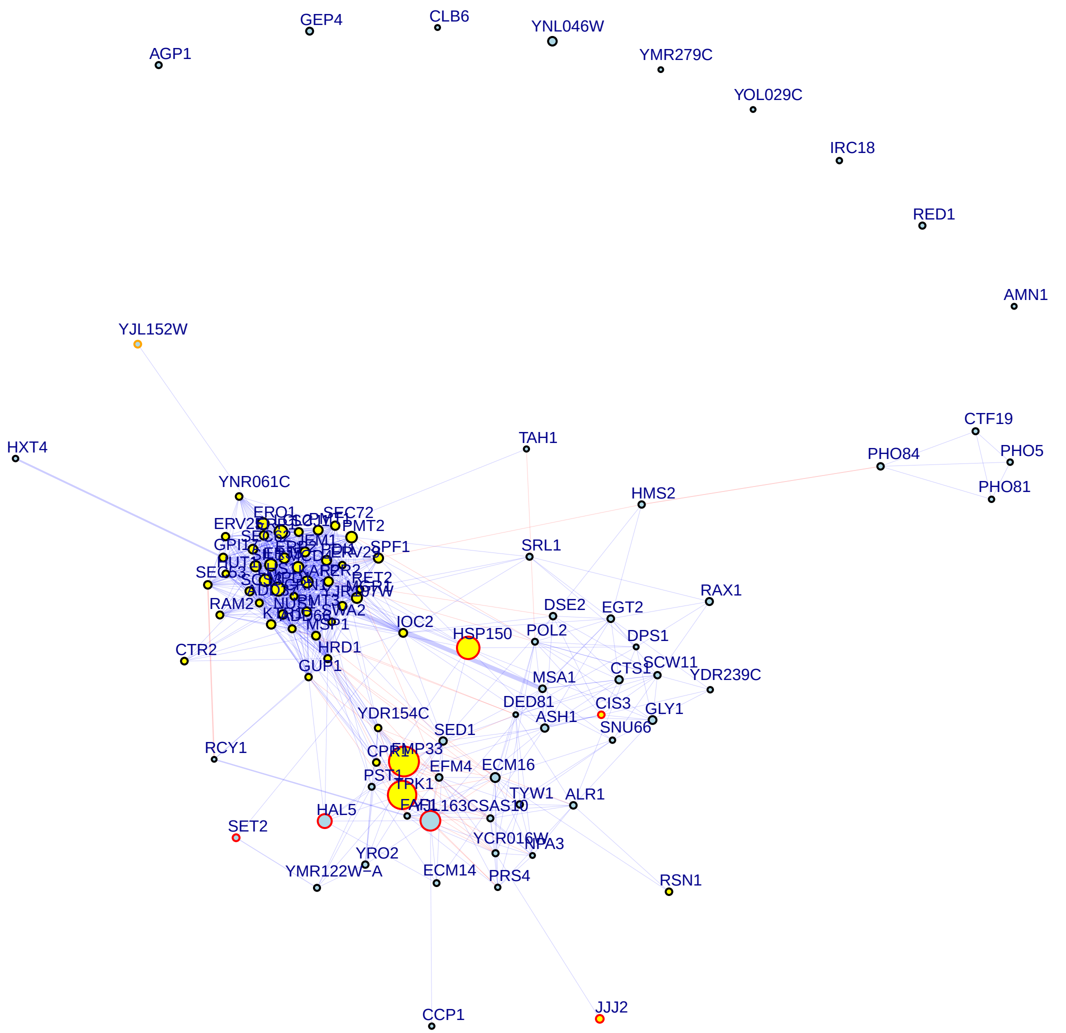

chrX:181214_T/C top 50 up genes

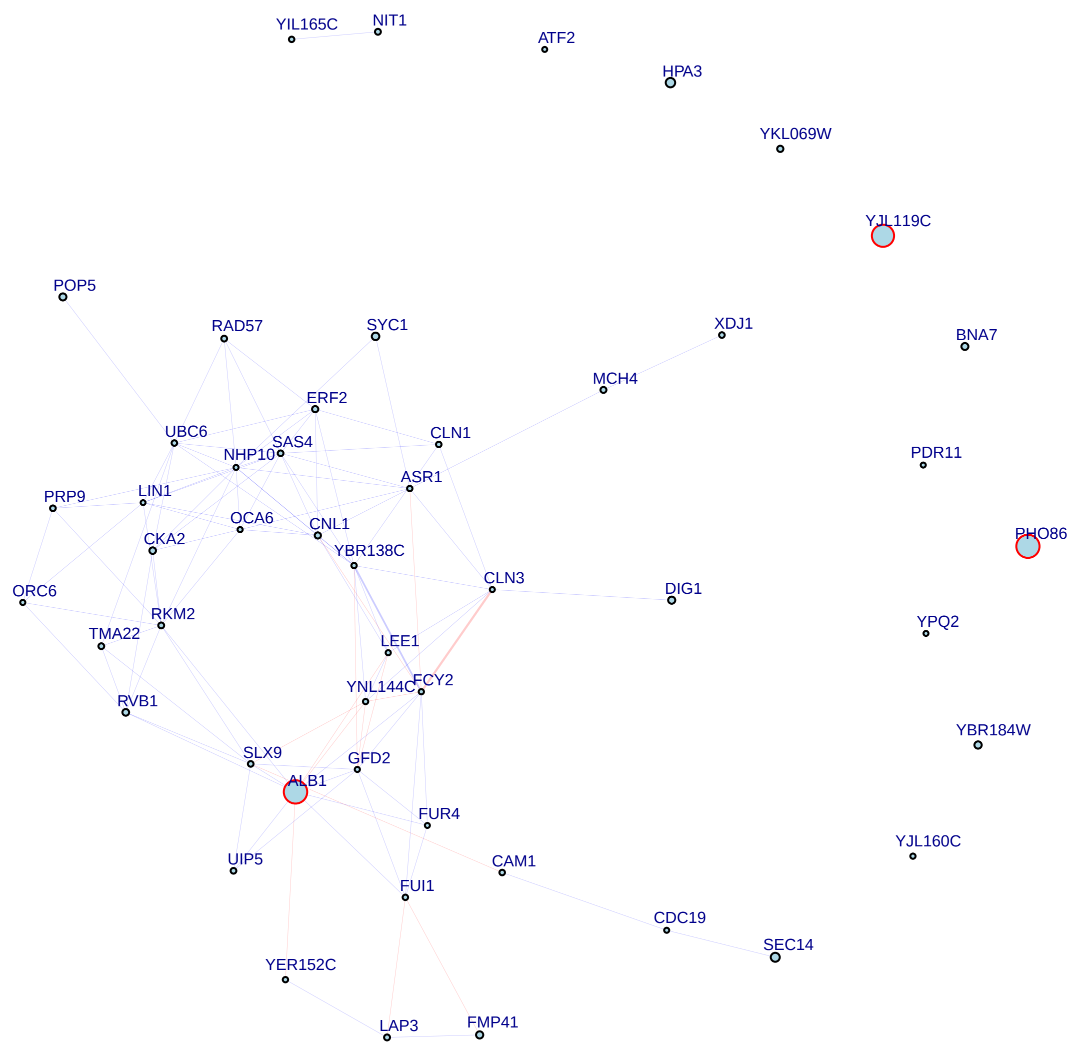

chrX:181214_T/C top 50 down genes

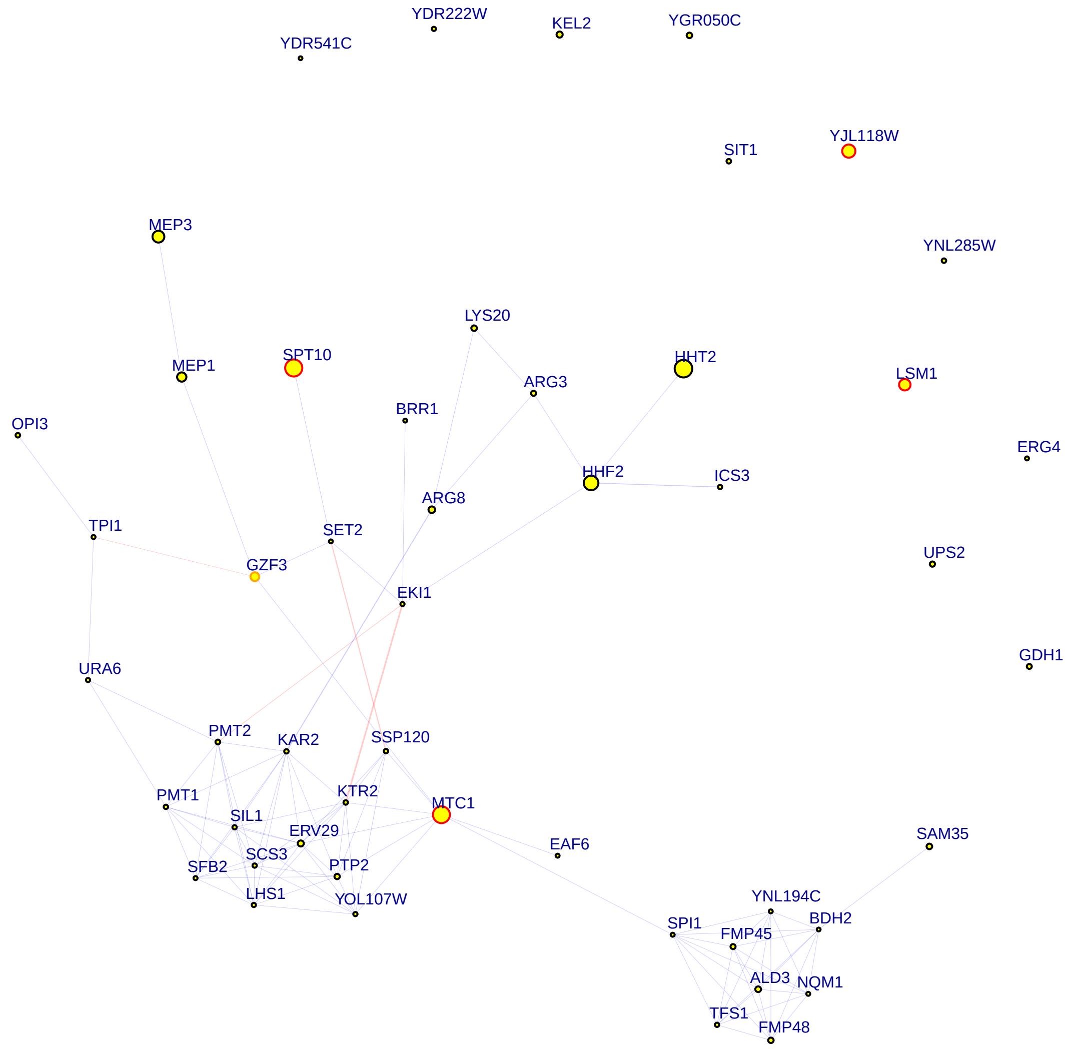

chrX:181214_T/C top 100 up and down genes

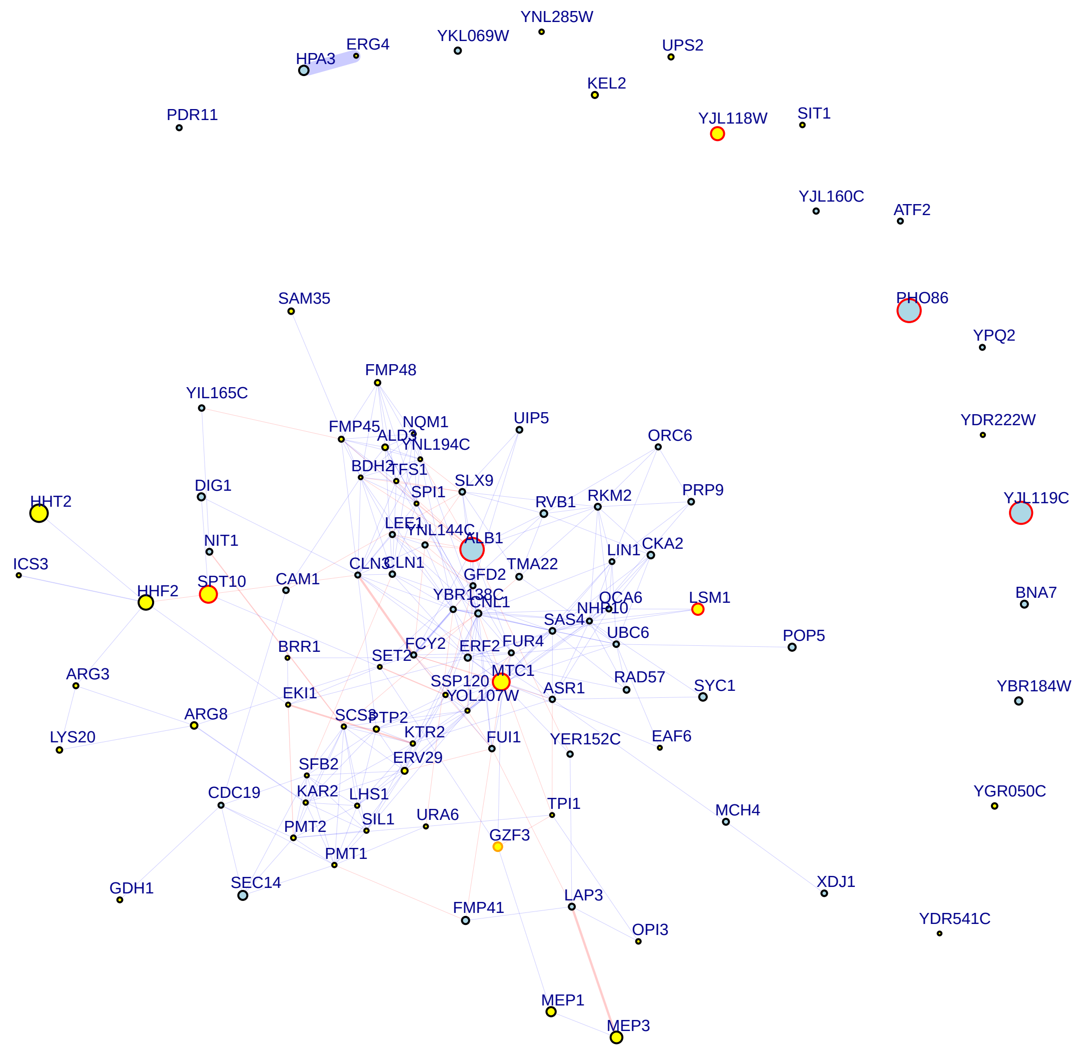

chrX:330144_C/T top 50 up genes

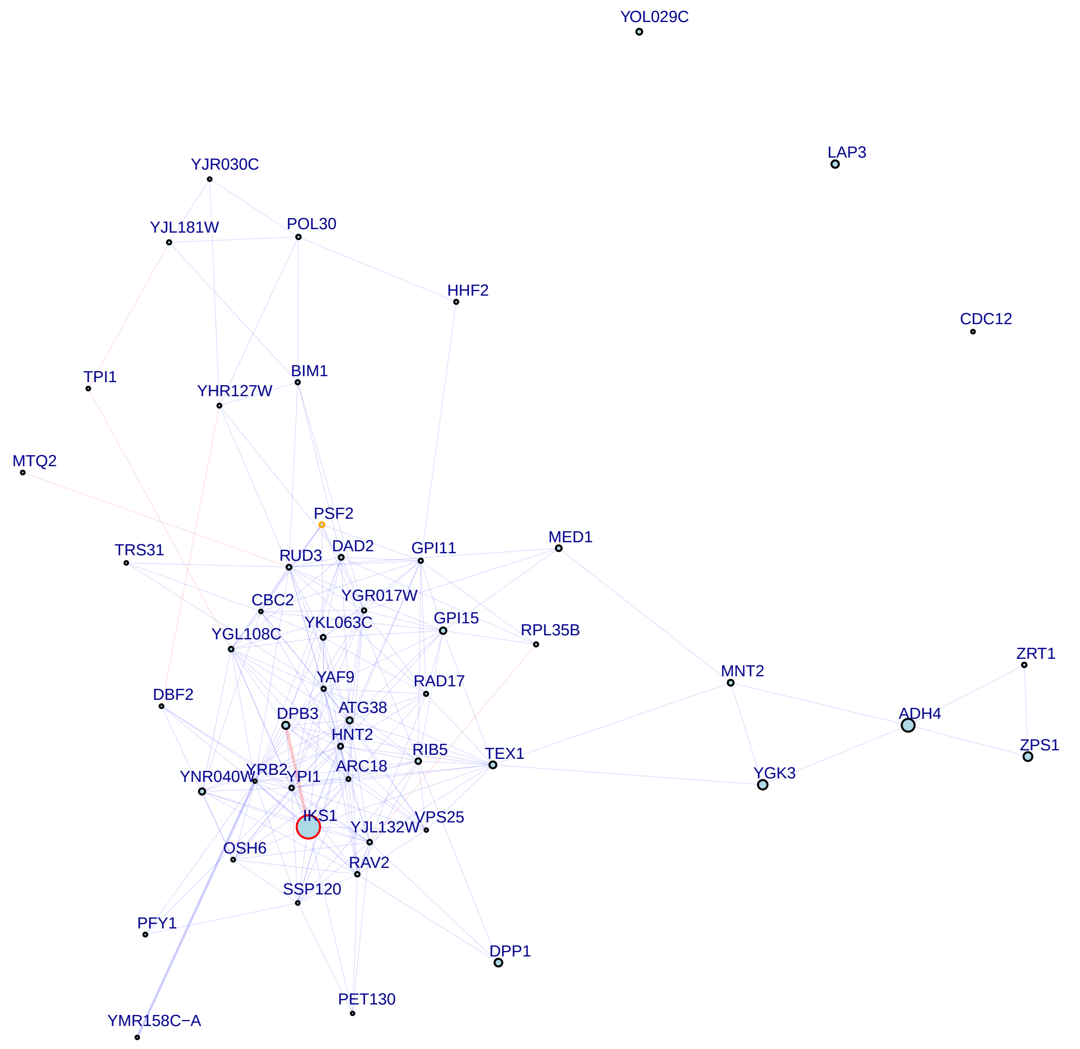

chrX:330144_C/T top 50 down genes

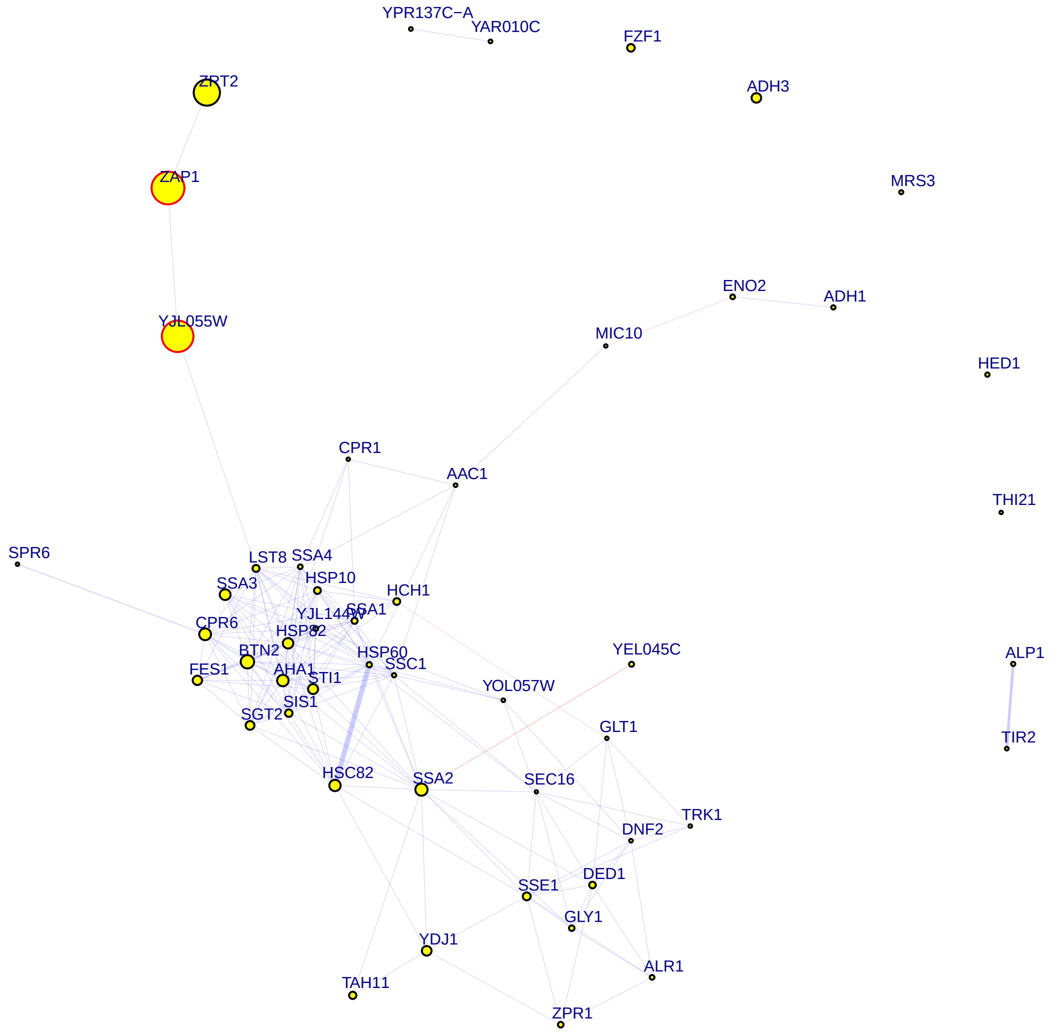

chrX:330144_C/T top 100 up and down genes

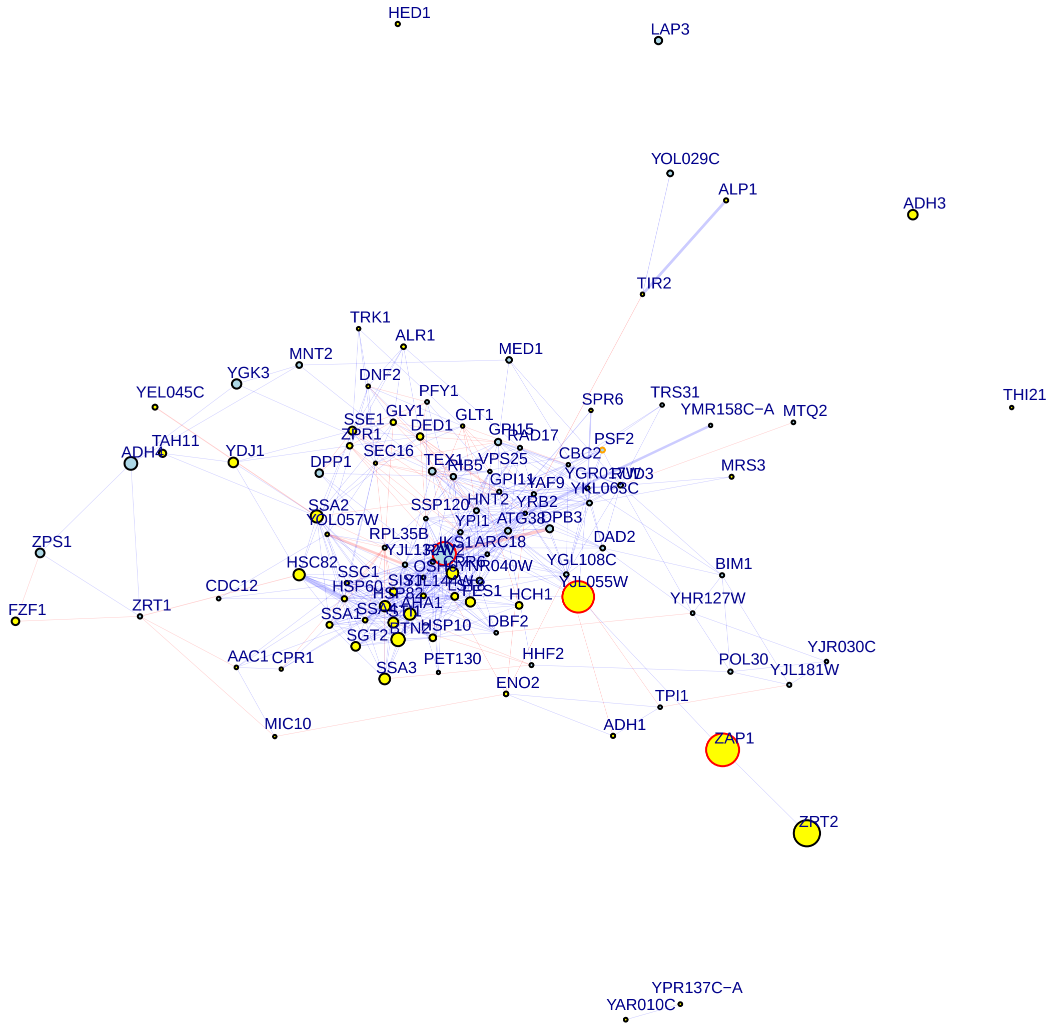

chrX:426287_C/A top 50 up genes

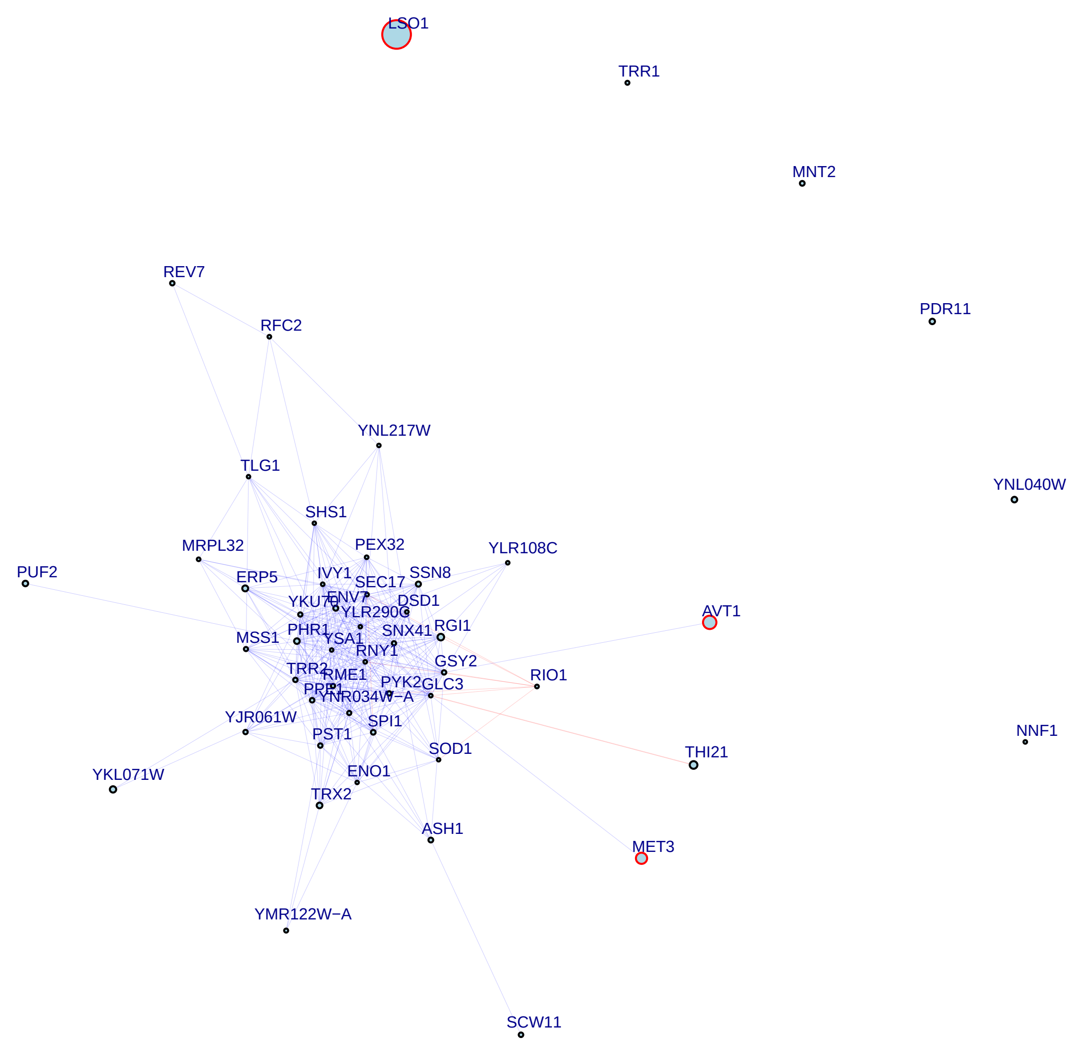

chrX:426287_C/A top 50 down genes

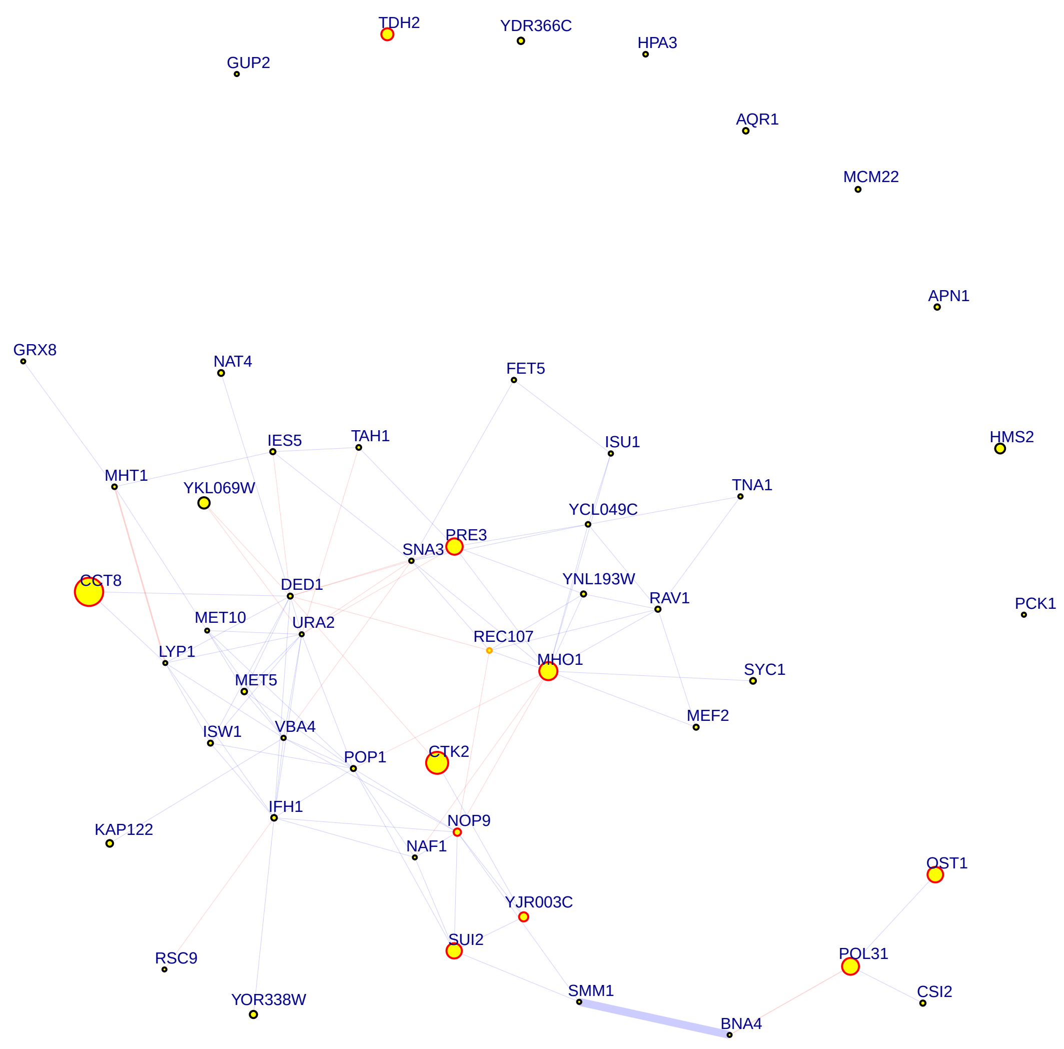

chrX:426287_C/A top 100 up and down genes

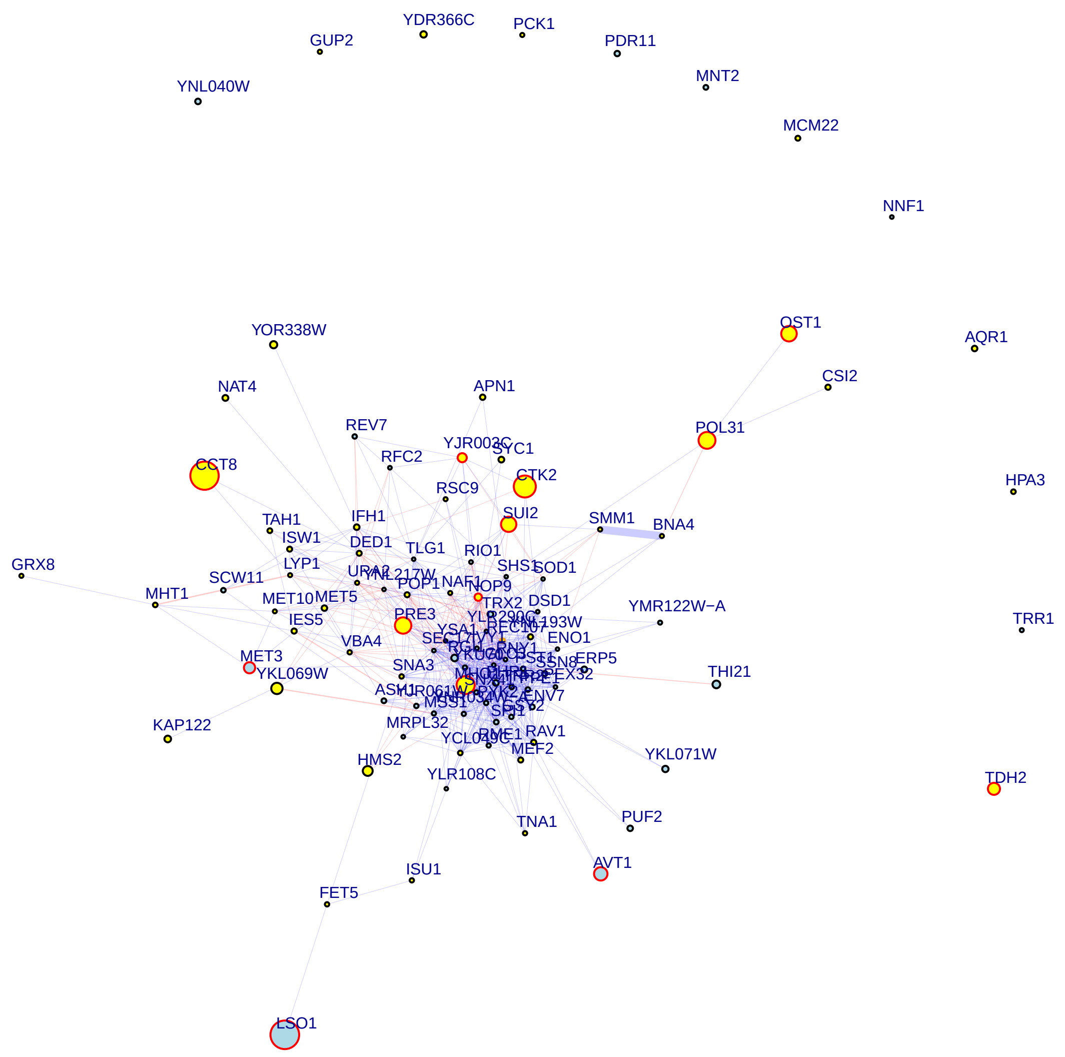

chrX:548174_T/C top 50 up genes

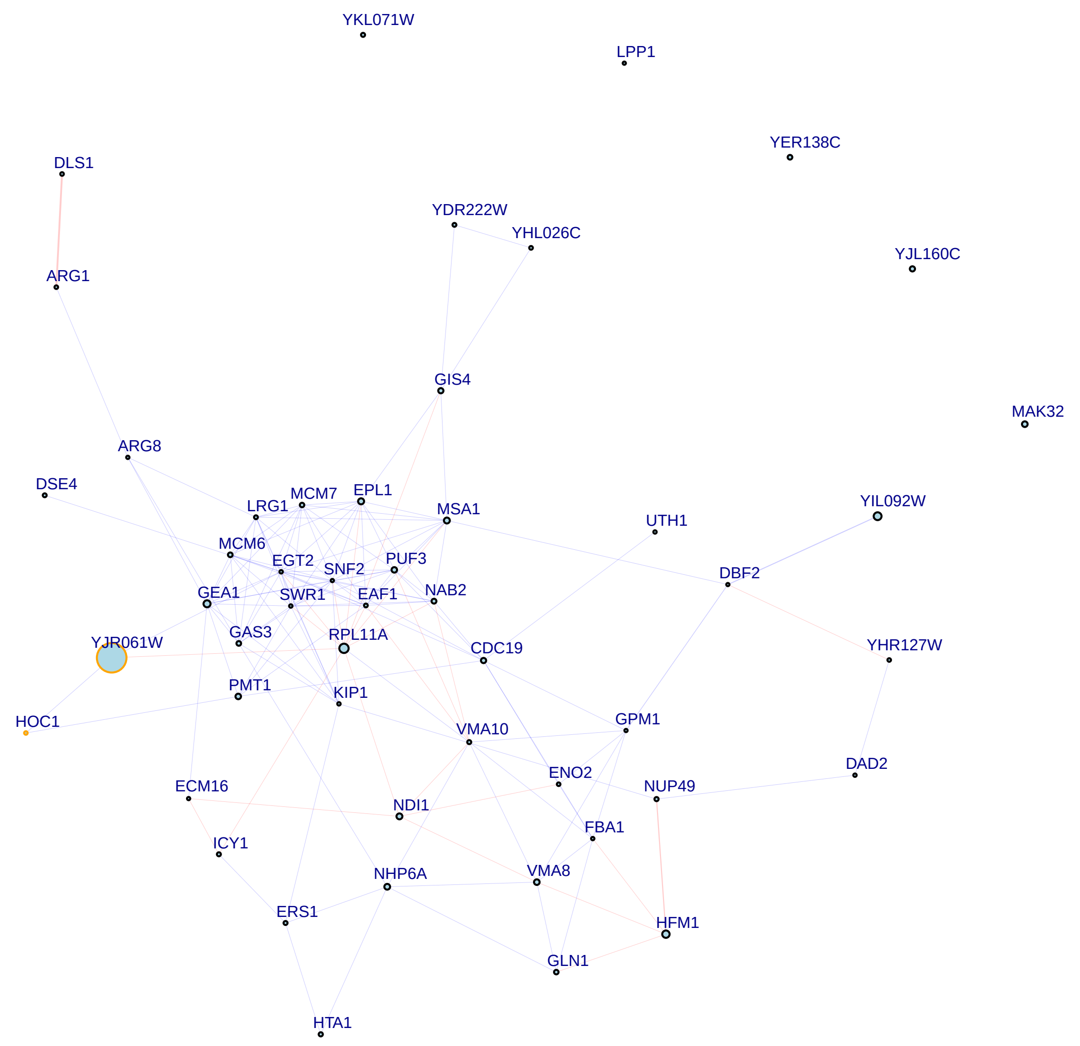

chrX:548174_T/C top 50 down genes

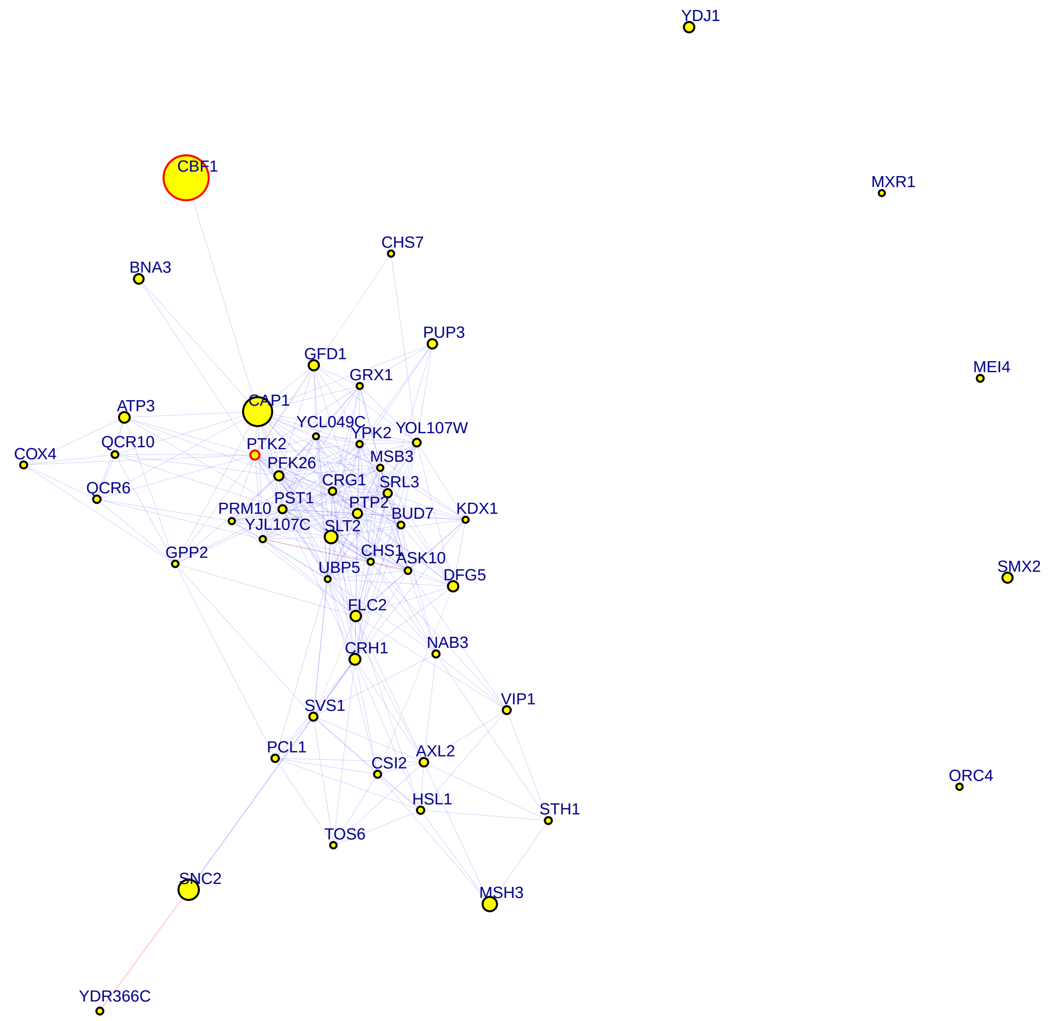

chrX:548174_T/C top 100 up and down genes

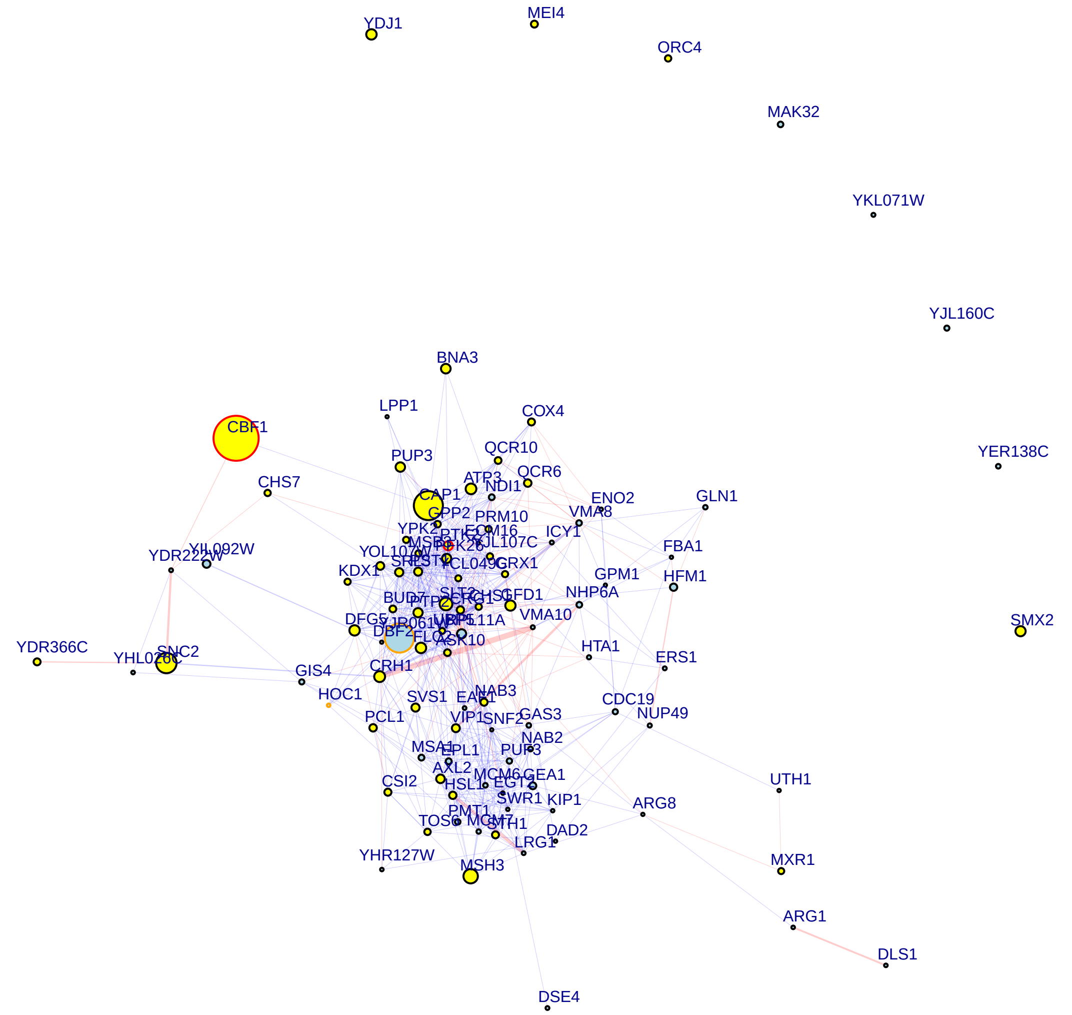

chrX:659425_T/C top 50 up genes

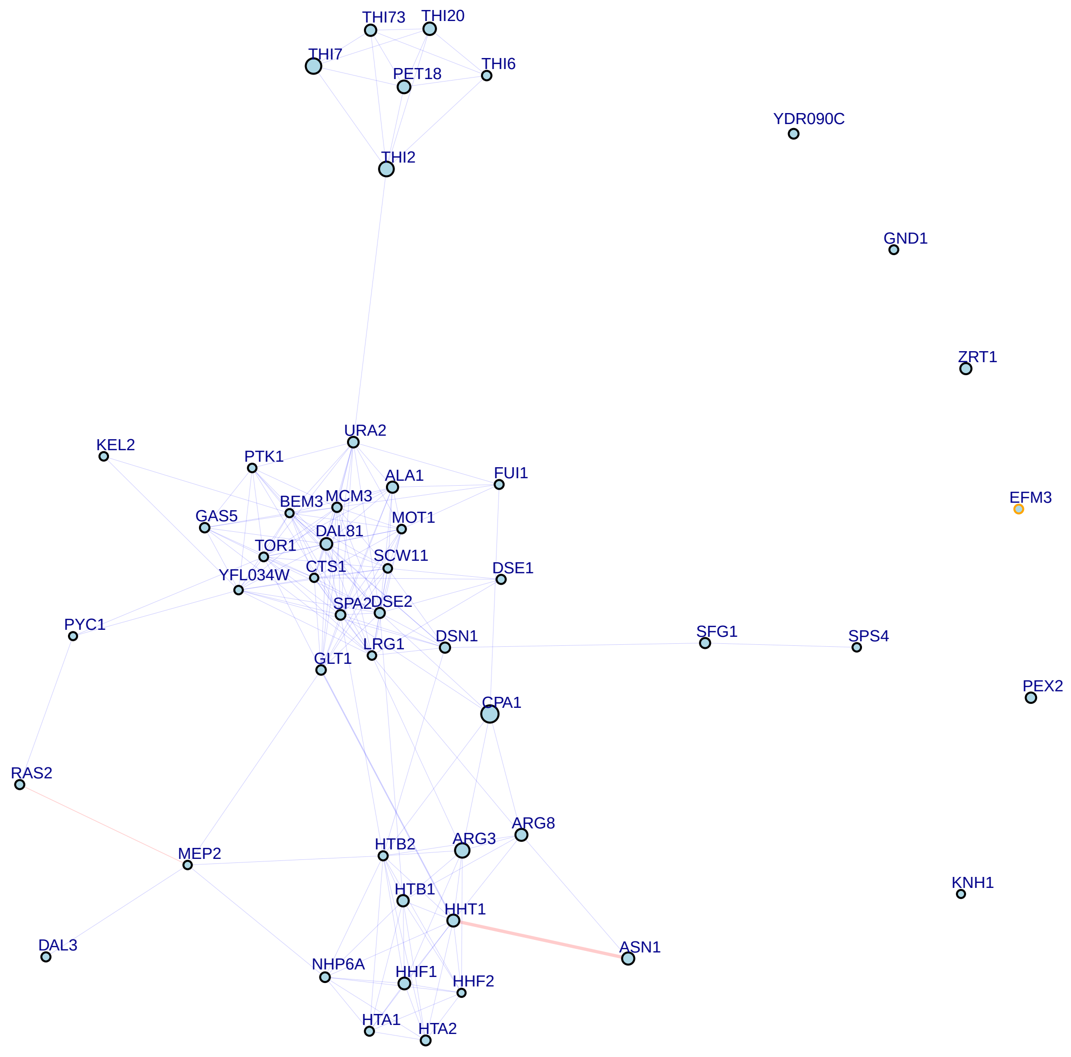

chrX:659425_T/C top 50 down genes

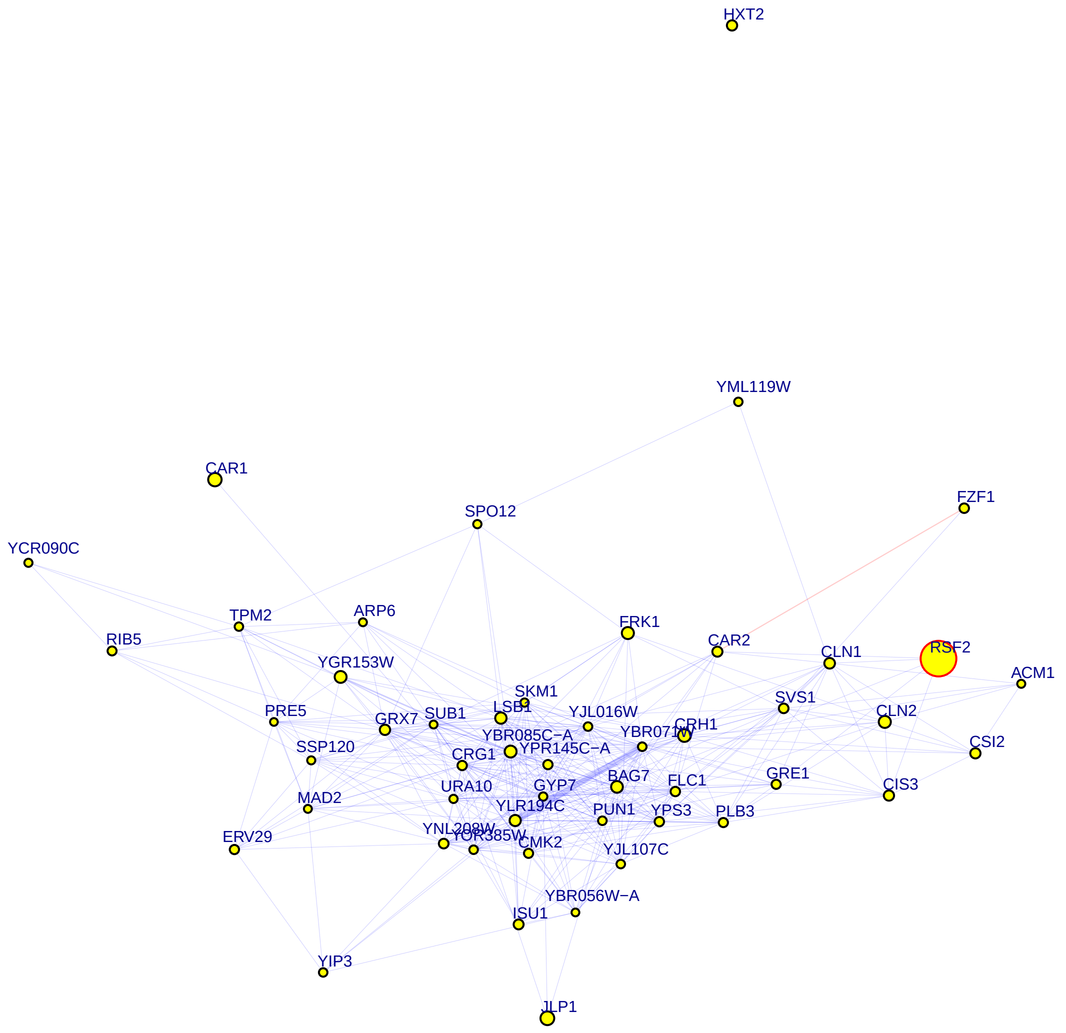

chrX:659425_T/C top 100 up and down genes

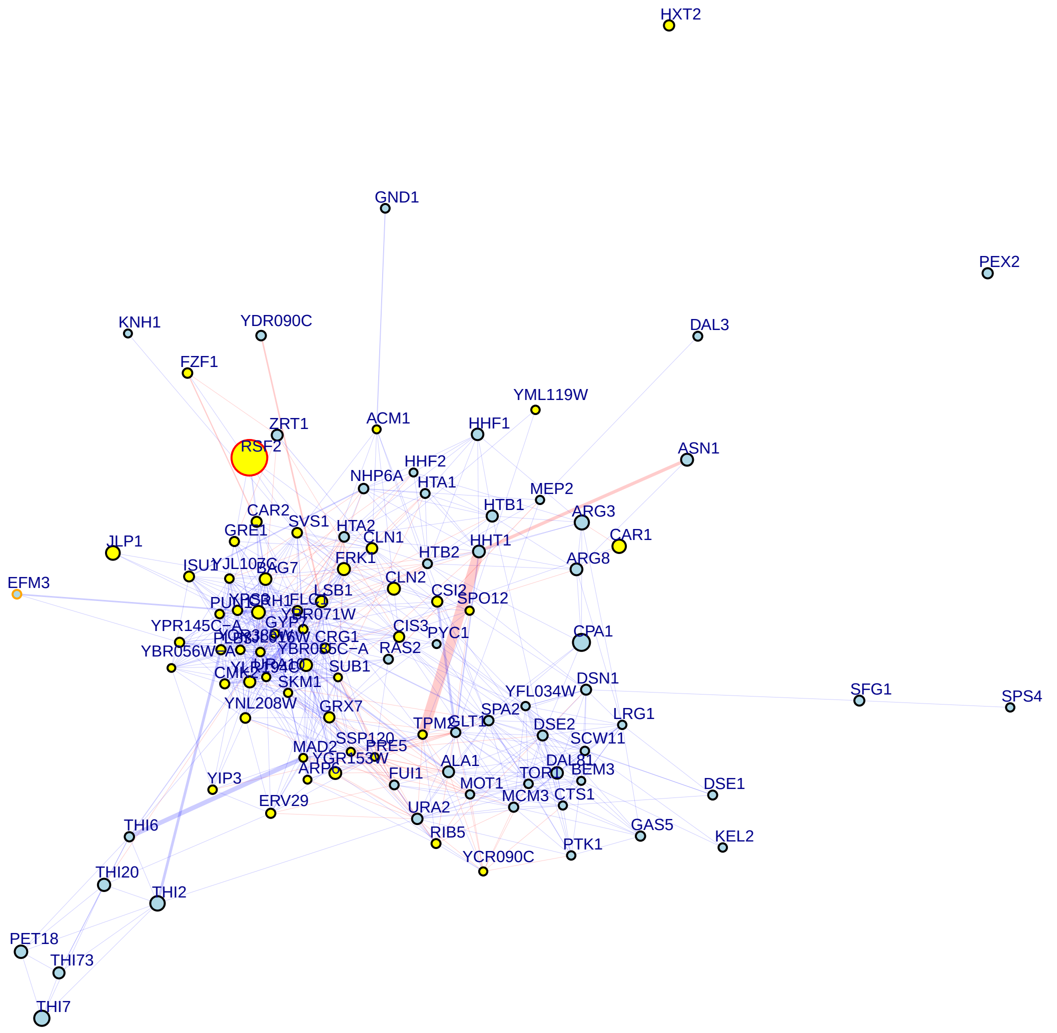

chrX:722130_T/C top 50 up genes

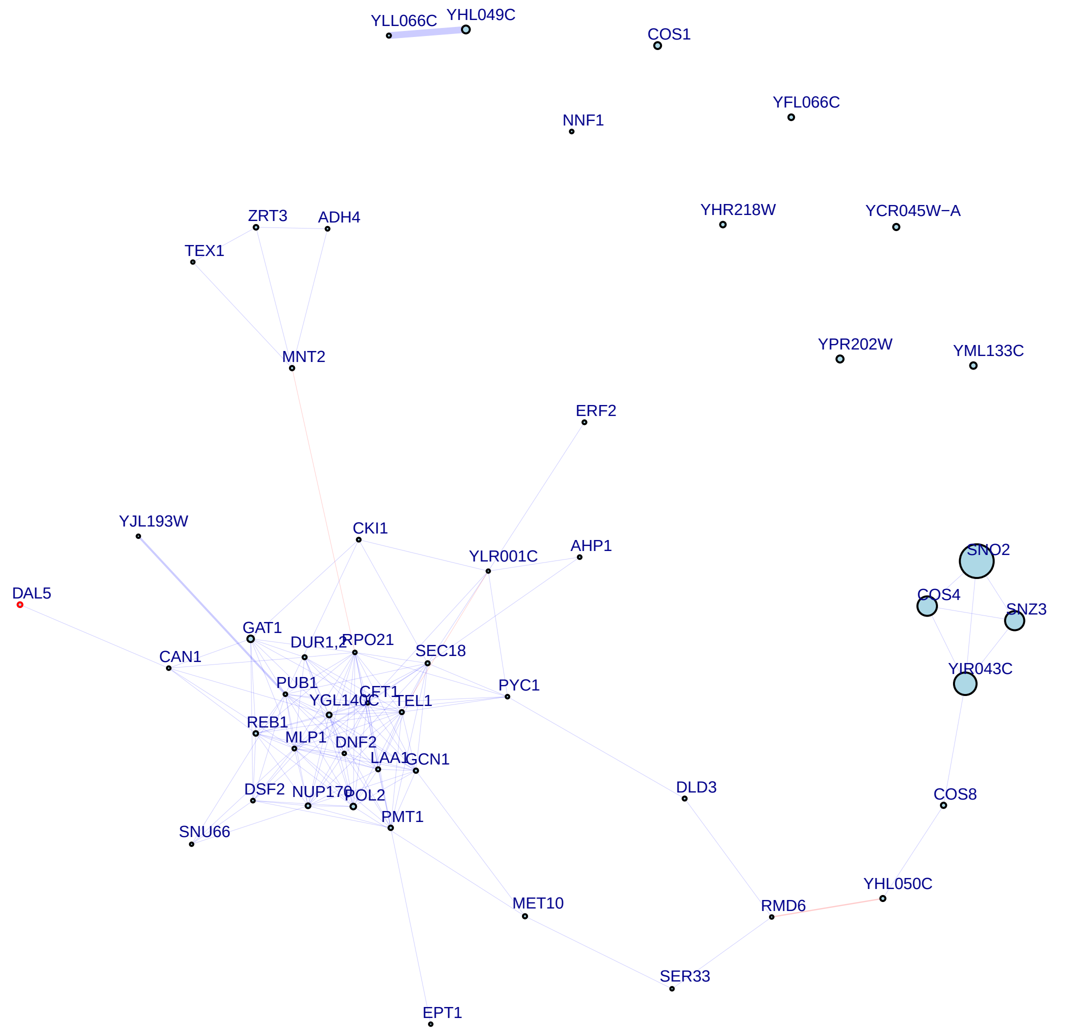

chrX:722130_T/C top 35 down genes

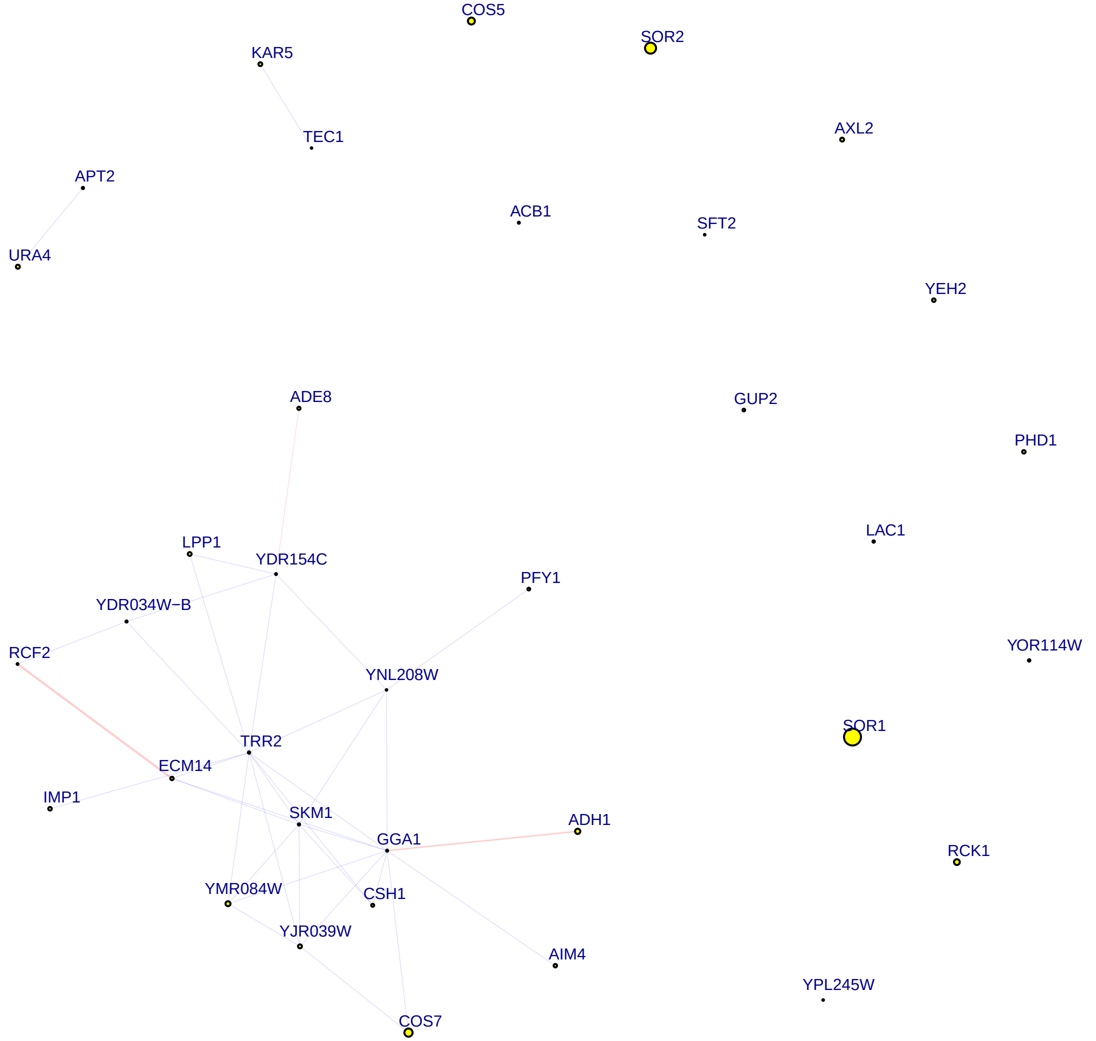

chrX:722130_T/C top 85 up and down genes

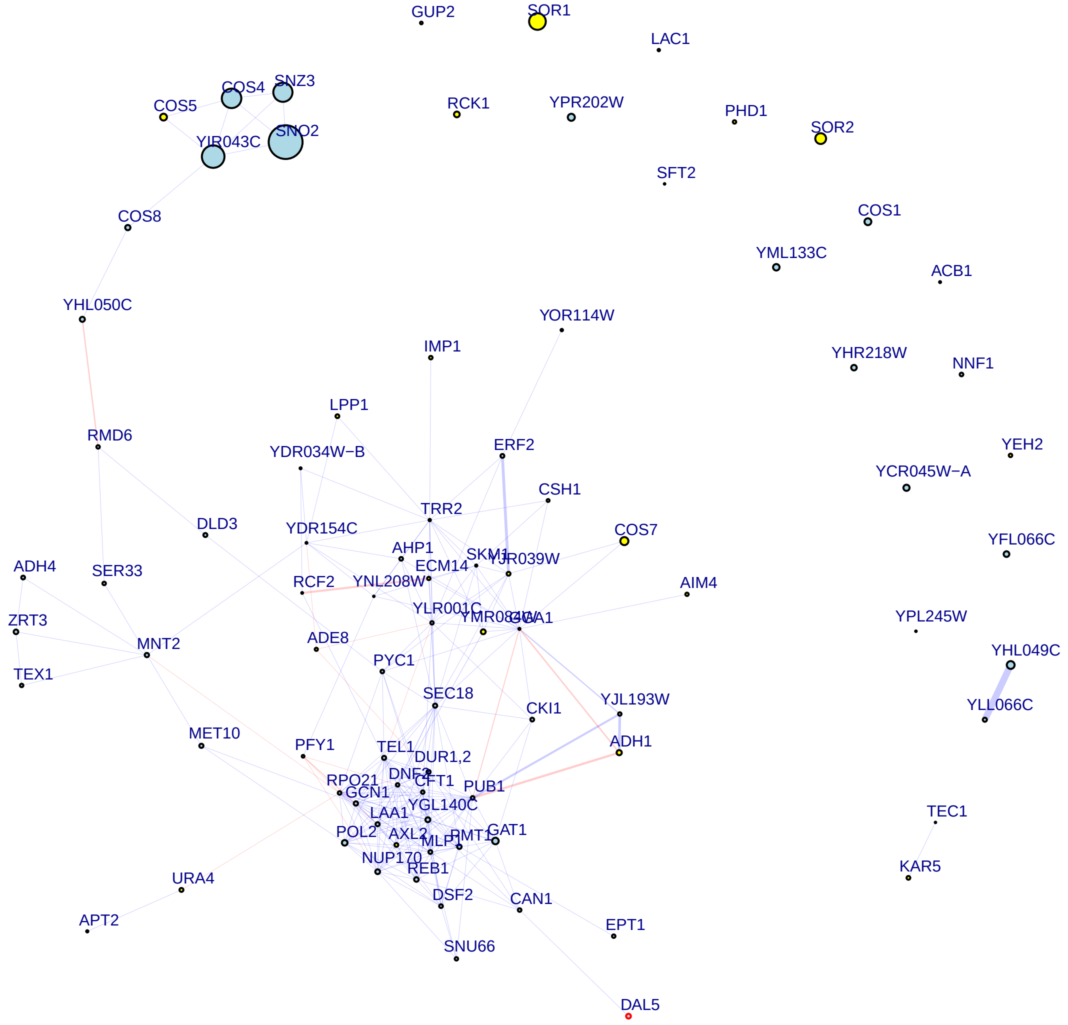

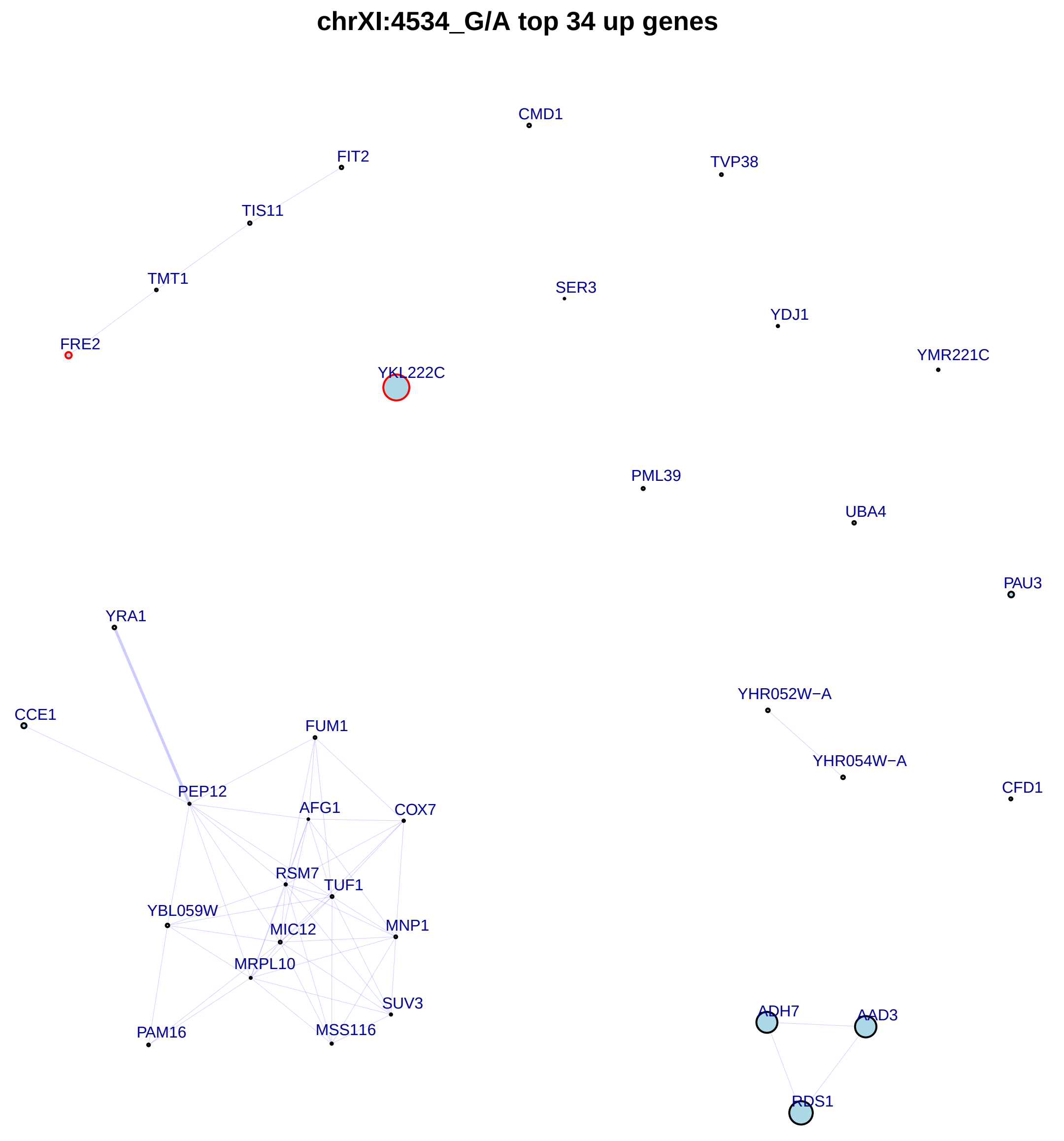
chrXI:4534_G/A top 34 up genes
CMD1
FIT2
TVP38
TIS11
TMT1
SER3
YDJ1
FRE2
YMR221C
YKL222C
PML39
UBA4
PAU3
YRA1
YHR052W−A
CCE1
FUM1
YHR054W−A
PEP12
AFG1
COX7
CFD1
RSM7
TUF1
YBL059W
MIC12
MNP1
MRPL10
SUV3
PAM16
MSS116
ADH7
AAD3
RDS1

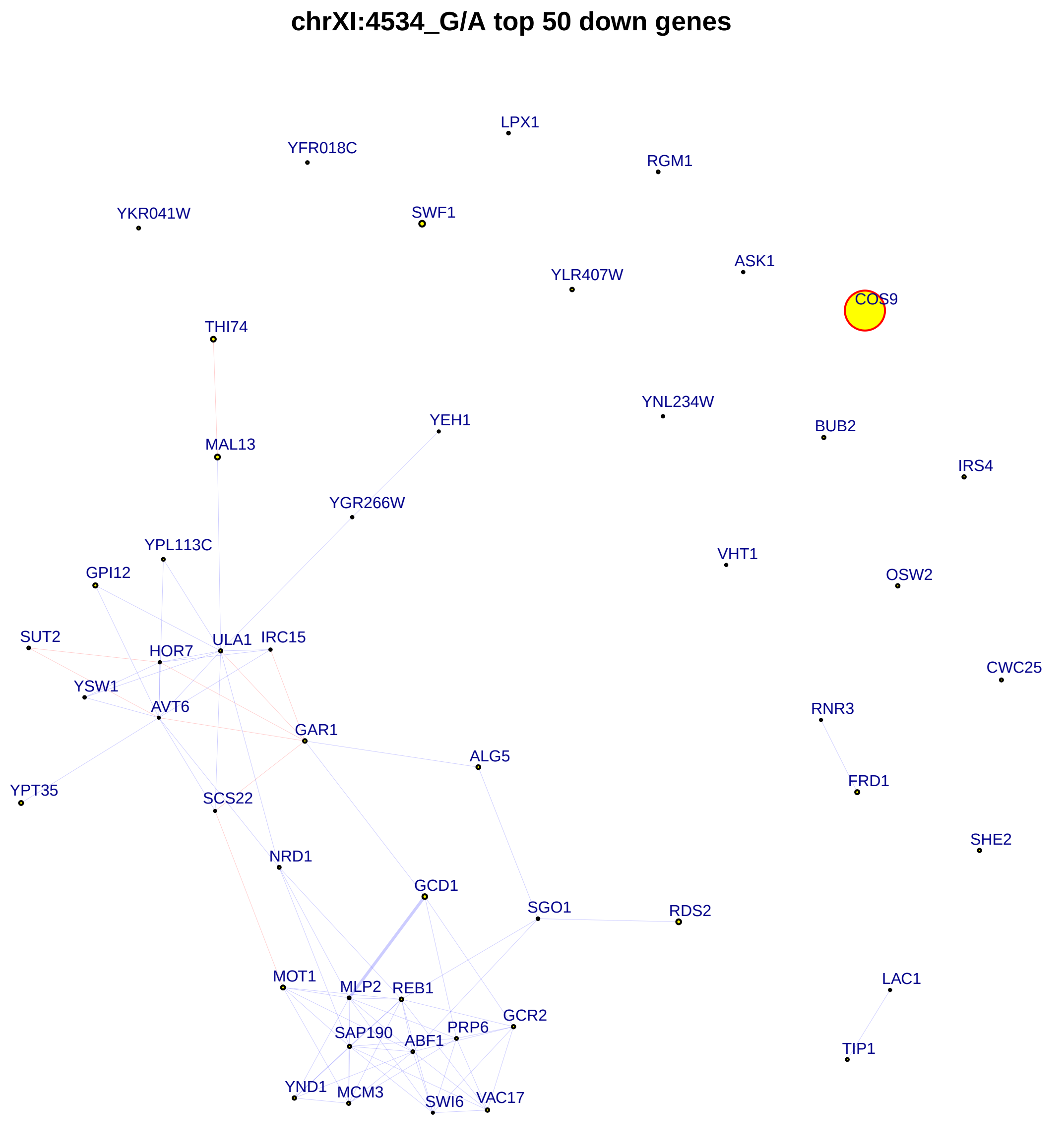
chrXI:4534_G/A top 50 down genes
LPX1
YFR018C
RGM1
YKR041W
SWF1
ASK1
COS9
YLR407W
THI74
YNL234W
YEH1
BUB2
IRS4
MAL13
YGR266W
YPL113C
VHT1
OSW2
GPI12
SUT2
ULA1
IRC15
HOR7
YSW1
CWC25
AVT6
RNR3
GAR1
ALG5
FRD1
YPT35
SCS22
SHE2
NRD1
GCD1
SGO1
RDS2
MOT1
MLP2
REB1
LAC1
GCR2
PRP6
SAP190
ABF1
TIP1
YND1
MCM3
SWI6
VAC17

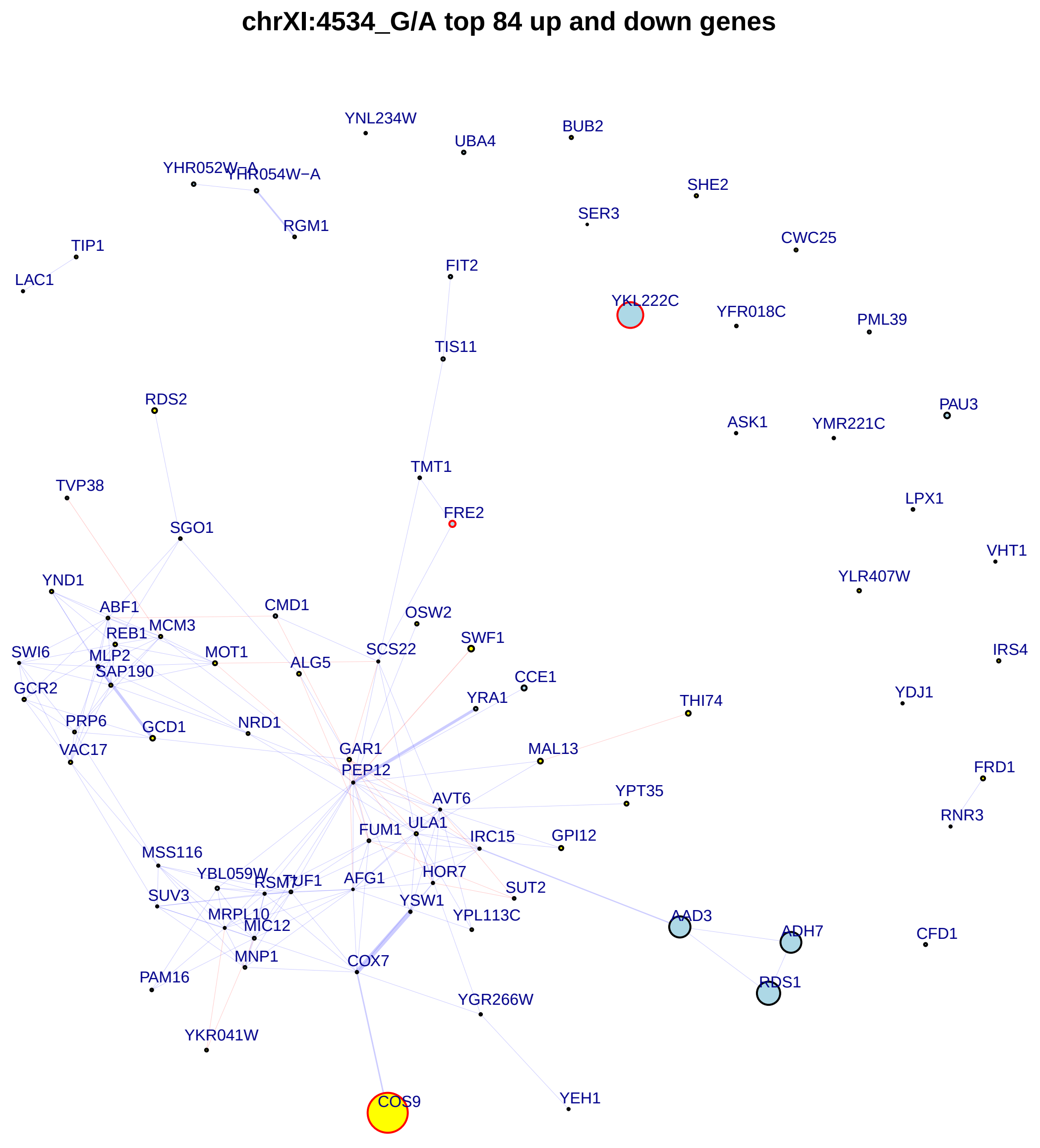
chrXI:4534_G/A top 84 up and down genes
YNL234W
UBA4
BUB2
YHR052W−A
YHR054W−A
SHE2
SER3
RGM1
CWC25
TIP1
FIT2
LAC1
YKL222C
YFR018C
PML39
TIS11
RDS2
PAU3
ASK1
YMR221C
TVP38
TMT1
LPX1
FRE2
SGO1
VHT1
YND1
YLR407W
CMD1
OSW2
ABF1
MCM3
REB1
SWF1
SWI6
MOT1
SCS22
MLP2
ALG5
IRS4
SAP190
YRA1
CCE1
GCR2
YDJ1
THI74
PRP6
GCD1
NRD1
VAC17
GAR1
MAL13
FRD1
PEP12
AVT6
YPT35
RNR3
FUM1
ULA1
IRC15
GPI12
MSS116
YBL059W
RSM7
TUF1
AFG1
HOR7
SUT2
SUV3
YSW1
AAD3
MRPL10
YPL113C
ADH7
CFD1
MIC12
MNP1
COX7
RDS1
PAM16
YGR266W
YKR041W
COS9
YEH1

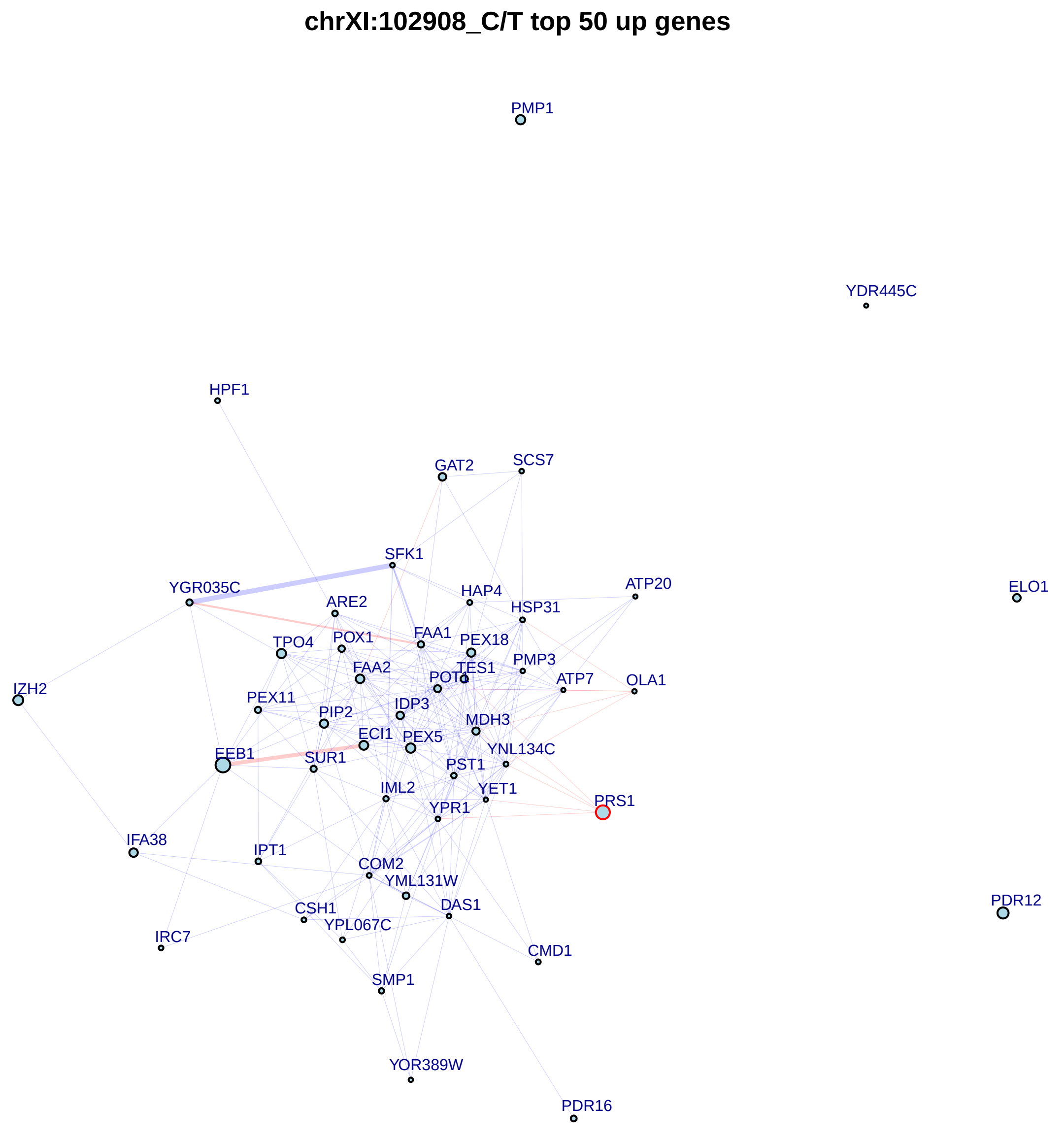
chrXI:102908_C/T top 50 up genes
PMP1
YDR445C
HPF1
GAT2
SCS7
SFK1
YGR035C
ARE2
HAP4
HSP31
ATP20
ELO1
TPO4
POX1
FAA1
PEX18
PMP3
IZH2
FAA2
POT1
TES1
ATP7
OLA1
PEX11
IDP3
PIP2
MDH3
ECI1
PEX5
YNL134C
FEB1
SUR1
PST1
PRS1
IML2
YET1
YPR1
IFA38
IPT1
COM2
YML131W
CSH1
DAS1
IRC7
YPL067C
CMD1
SMP1
PDR12
YOR389W
PDR16

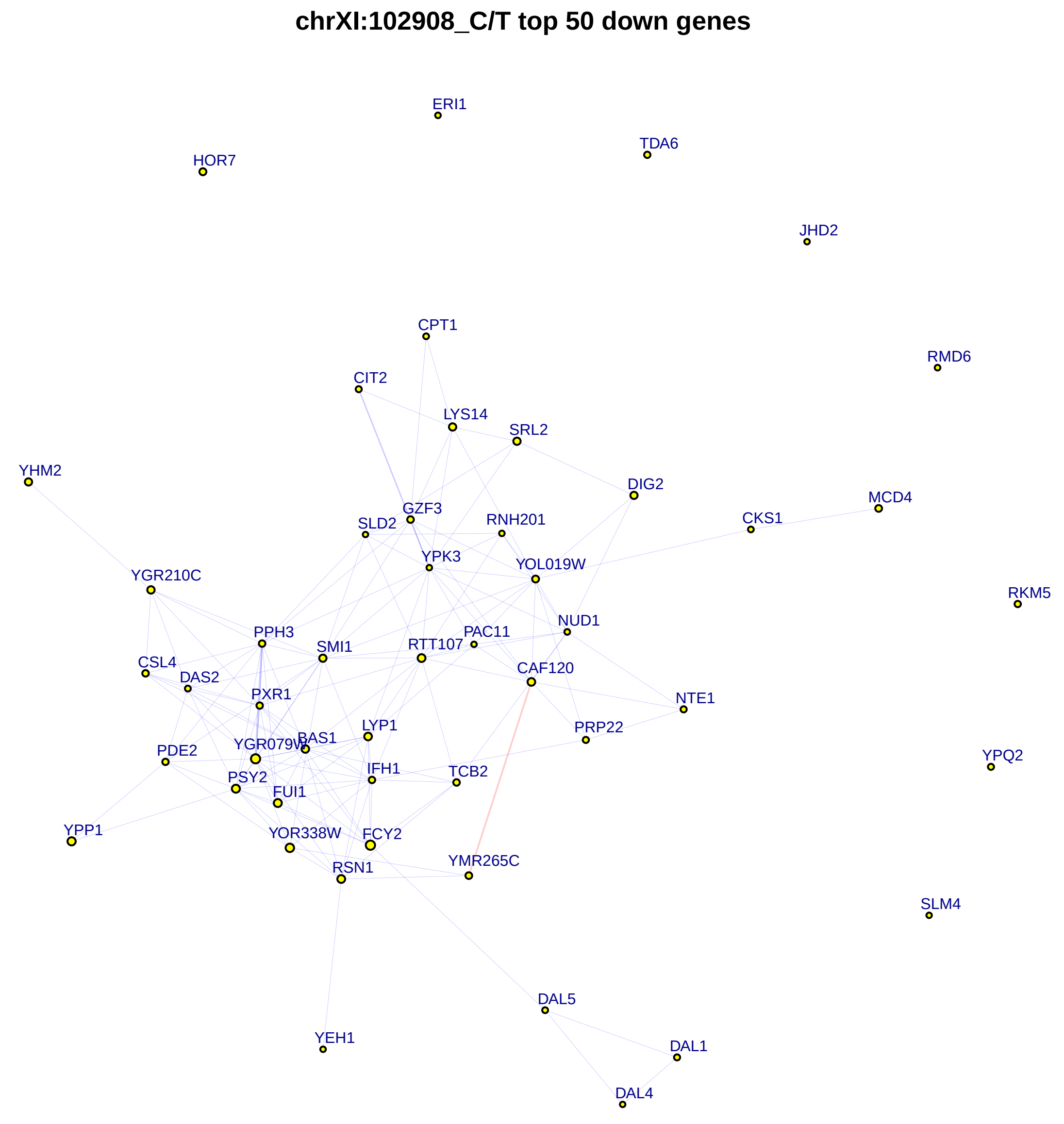
chrXI:102908_C/T top 50 down genes
ERI1
HOR7
TDA6
JHD2
CPT1
CIT2
LYS14
SRL2
RMD6
YHM2
DIG2
GZF3
RNH201
SLD2
CKS1
MCD4
YPK3
YOL019W
YGR210C
RKM5
NUD1
PPH3
PAC11
SMI1
RTT107
CSL4
DAS2
CAF120
NTE1
PXR1
LYP1
PRP22
PDE2
YGR079W
BAS1
IFH1
TCB2
YPQ2
PSY2
FUI1
YPP1
YOR338W
FCY2
RSN1
YMR265C
SLM4
DAL5
YEH1
DAL1
DAL4

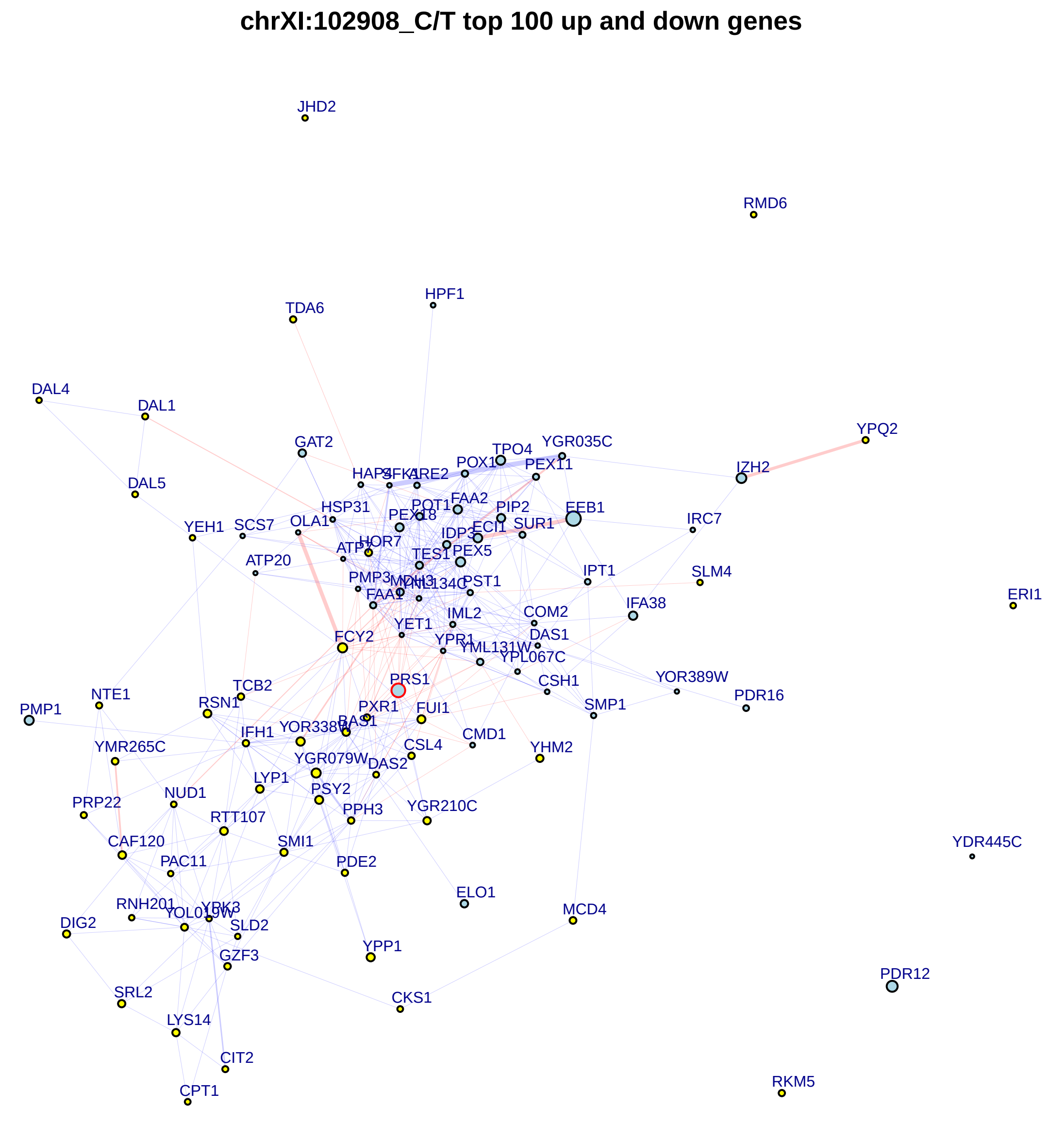
chrXI:102908_C/T top 100 up and down genes
JHD2
RMD6
HPF1
TDA6
DAL4
DAL1
YPQ2
GAT2
IZH2
DAL5
FEB1
IRC7
YEH1
SCS7
ERI1
IPT1
SLM4
ATP20
IFA38
FCY2
YOR389W
PDR16
PRS1
SMP1
NTE1
TCB2
PMP1
RSN1
PXR1
FUI1
YMR265C
CMD1
YHM2
PRP22
NUD1
LYP1
PSY2
YGR210C
CAF120
RTT107
PAC11
SMI1
PDE2
ELO1
RNH201
DIG2
SLD2
MCD4
GZF3
YPP1
SRL2
CKS1
LYS14
PDR12
CIT2
CPT1
RKM5
YDR445C

chrXI:232667_A/T top 50 up genes

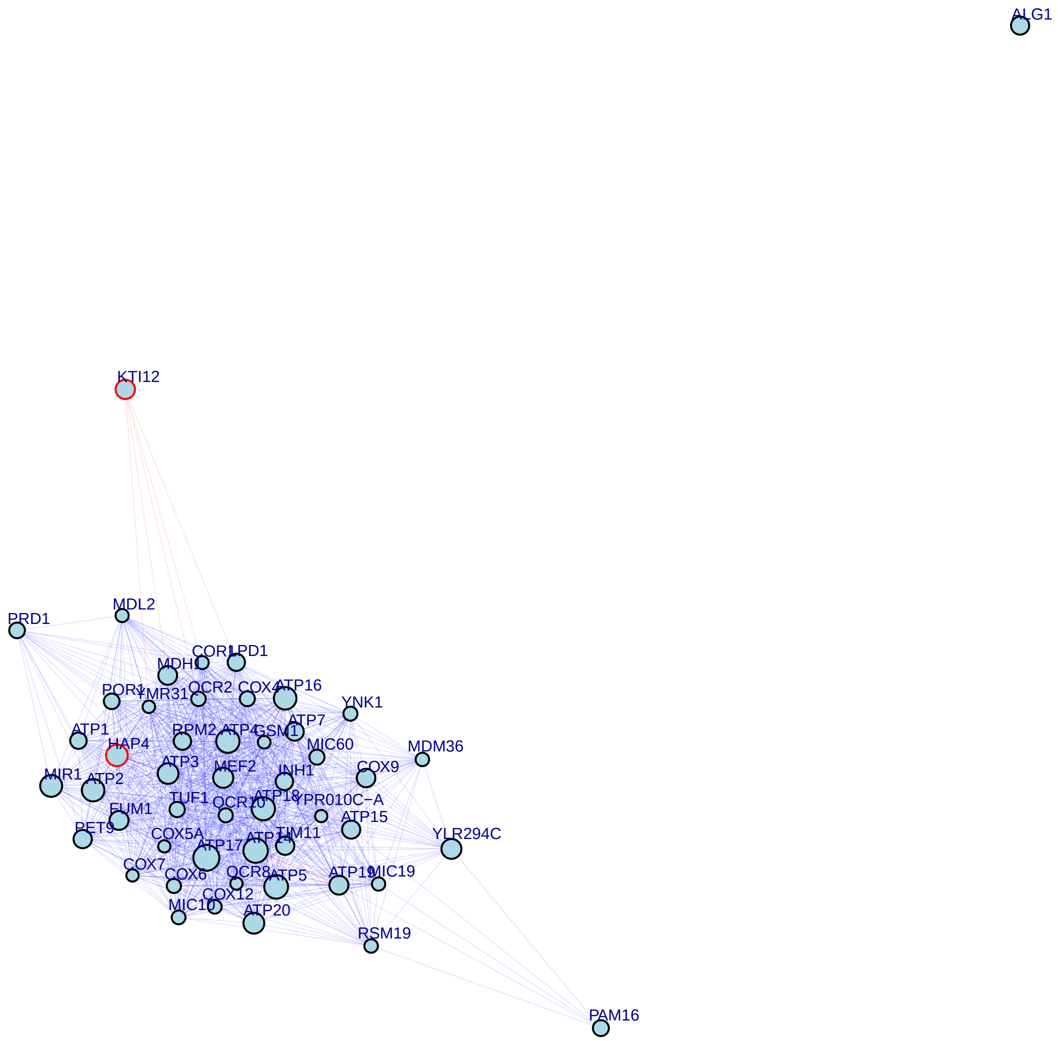

chrXI:232667_A/T top 50 down genes

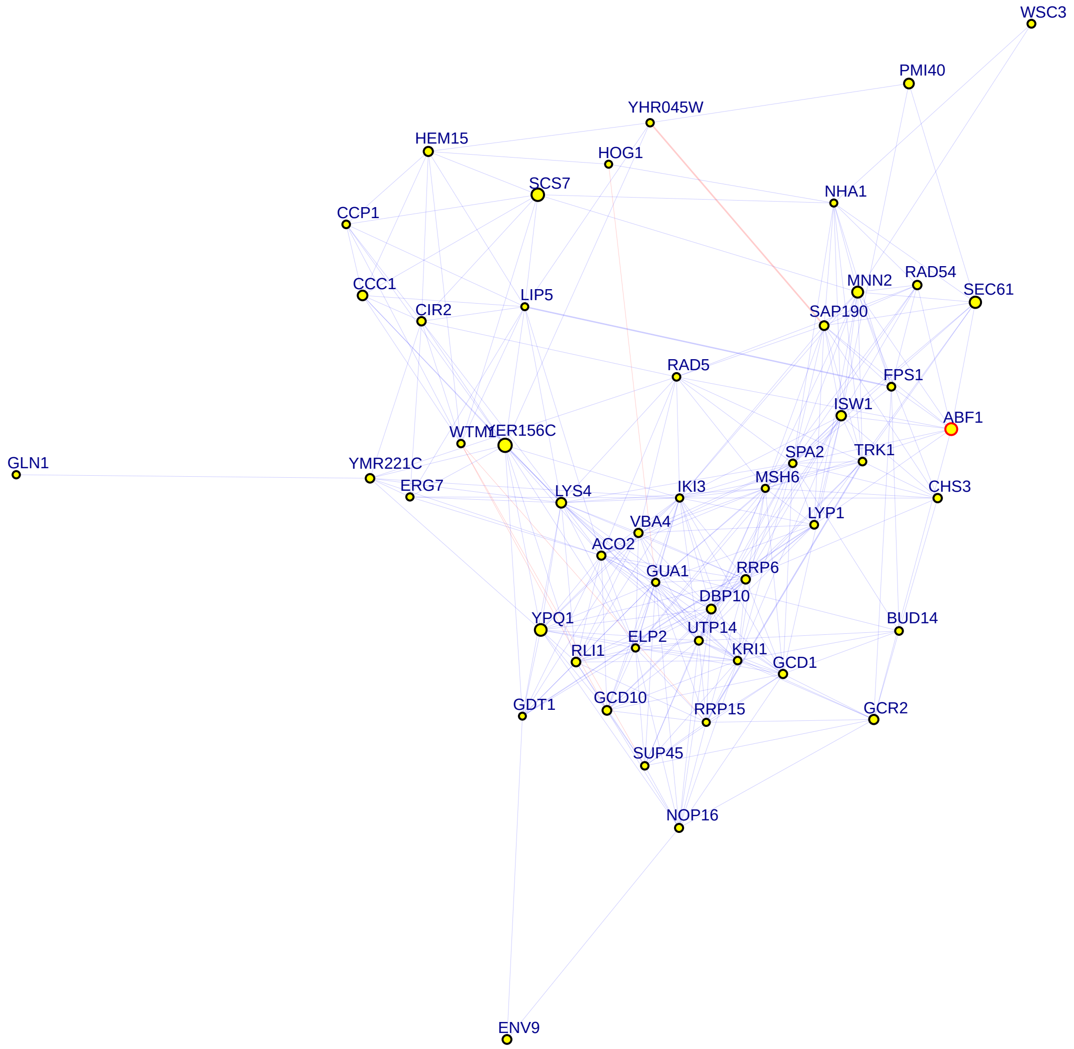

chrXI:232667_A/T top 100 up and down genes

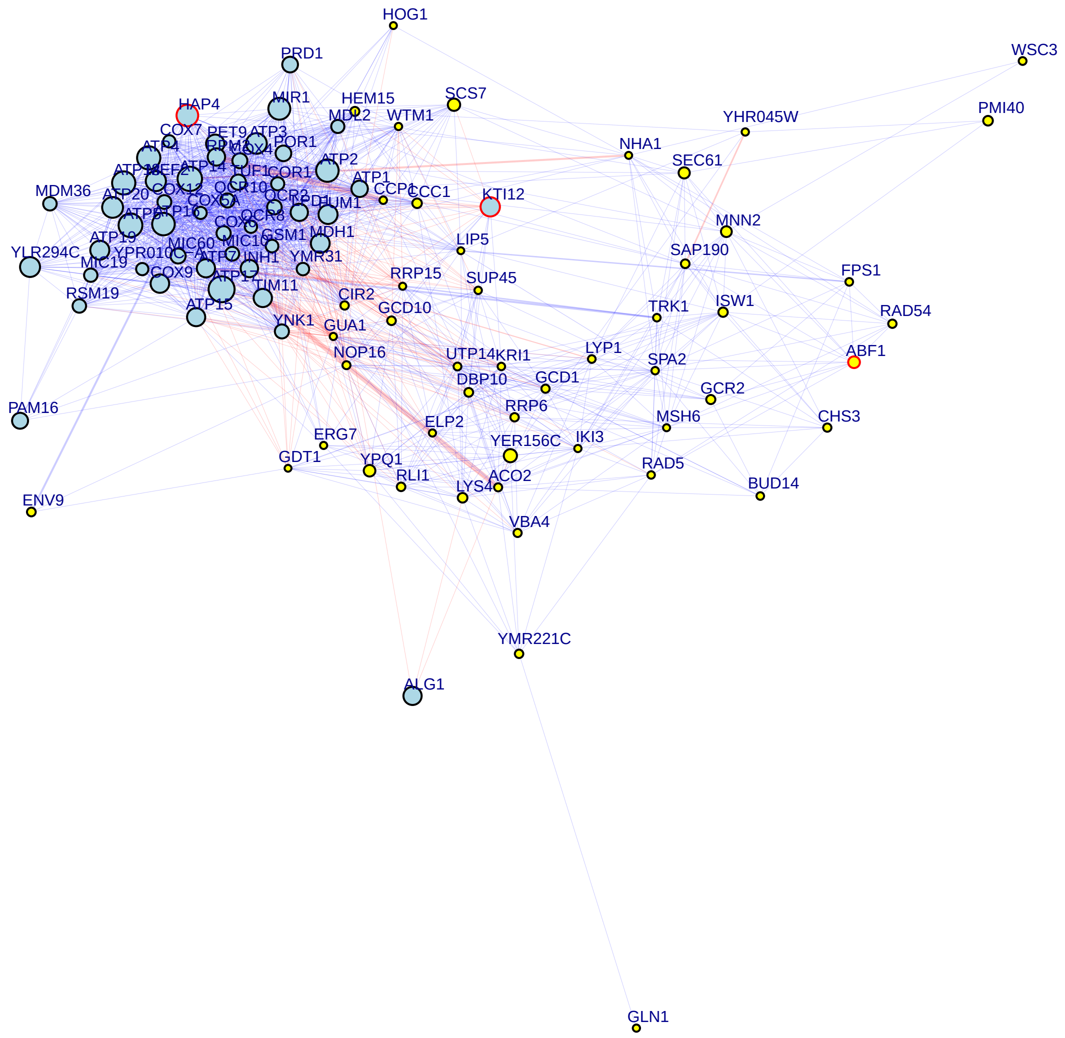

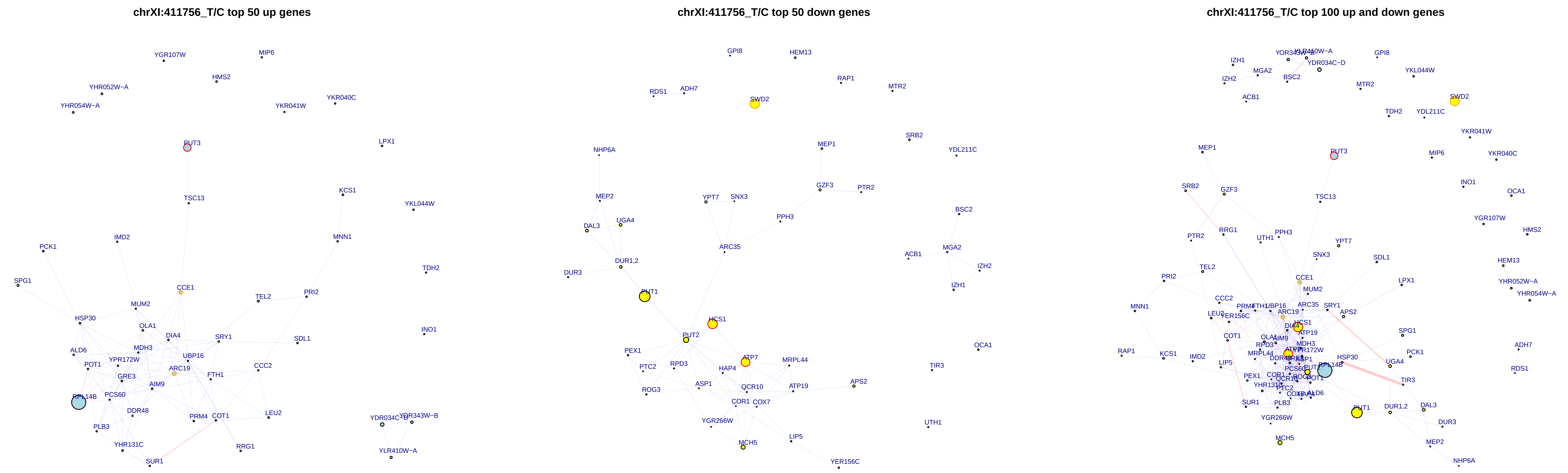

chrXI:411756_T/C top 50 up genes
YGR107W
MIP6
HMS2
YHR052W−A
YHR054W−A
YKR041W
YKR040C
PUT3
LPX1
TSC13
KCS1
YKL044W
MNN1
PCK1
IMD2
TDH2
SPG1
CCE1
PRI2
TEL2
MUM2
HSP30
OLA1
INO1
DIA4
SRY1
SDL1
MDH3
ALD6
YPR172W
UBP16
POT1
ARC19
CCC2
GRE3
FTH1
AIM9
RPL14B
PCS60
DDR48
PRM4
COT1
LEU2
PLB3
YHR131C
RRG1
SUR1
YLR410W−A
chrXI:411756_T/C top 50 down genes
GPI8
HEM13
RAP1
MTR2
RDS1
ADH7
SWD2
SRB2
YDL211C
NHP6A
MEP1
GZF3
PTR2
MEP2
YPT7
SNX3
BSC2
UGA4
PPH3
DAL3
ARC35
ACB1
MGA2
DUR1,2
IZH2
DUR3
IZH1
PUT1
HCS1
PUT2
QCA1
PEX1
MRPL44
TIR3
PTC2
RPD3
HAP4
APS2
ATP7
ASP1
QCR10
ATP19
ROG3
COR1
COX7
YGR266W
UTH1
MCH5
LIP5
YER156C
chrXI:411756_T/C top 100 up and down genes

chrXI:533229_G/C top 50 up genes

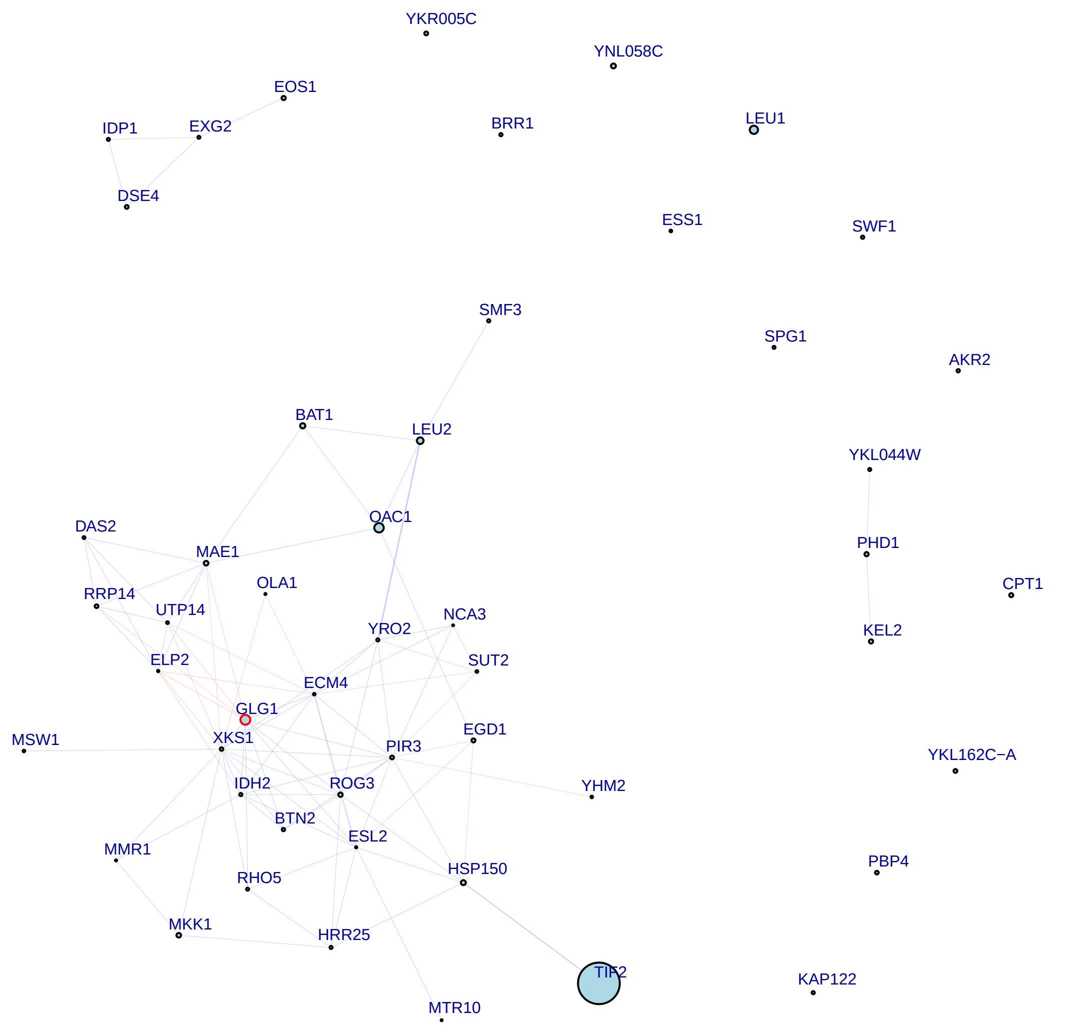

chrXI:533229_G/C top 50 down genes

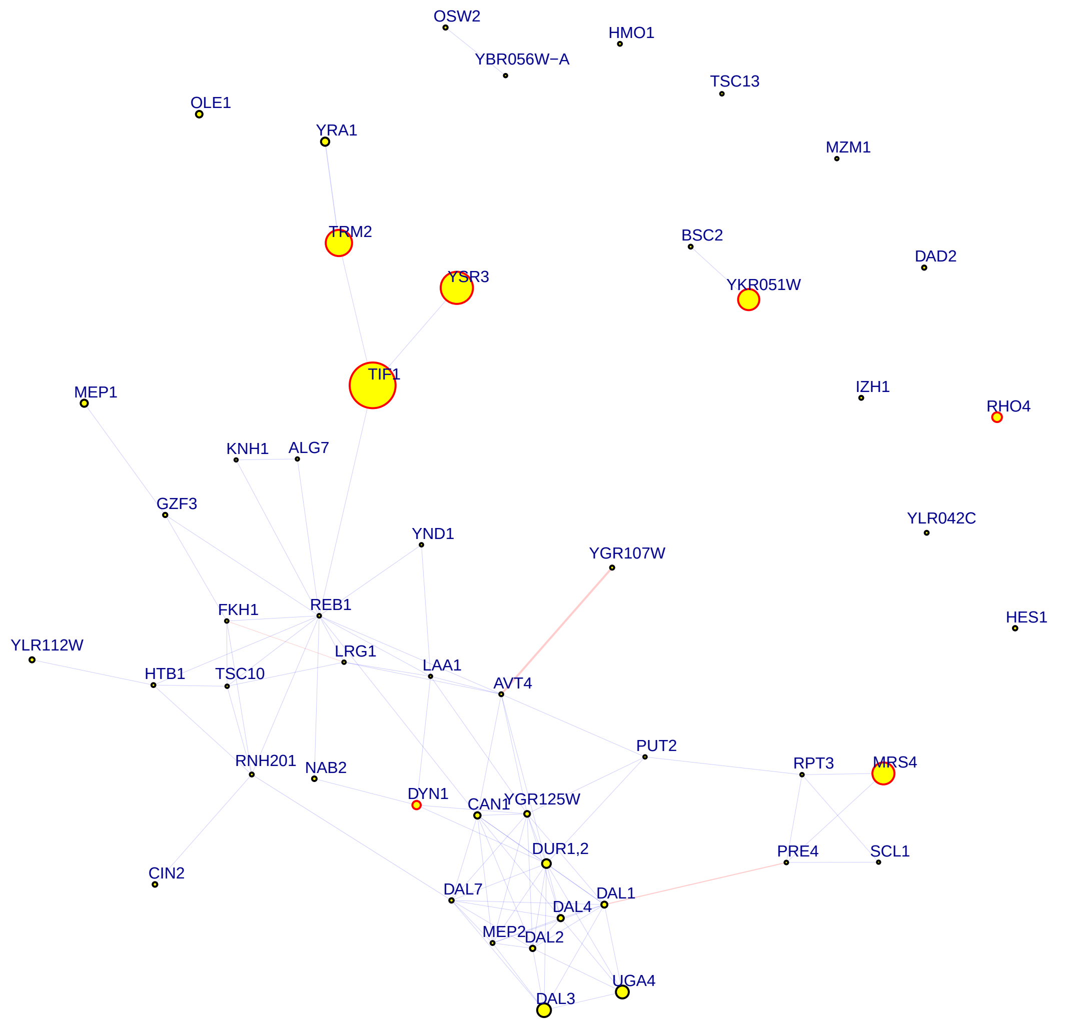

chrXI:533229_G/C top 100 up and down genes

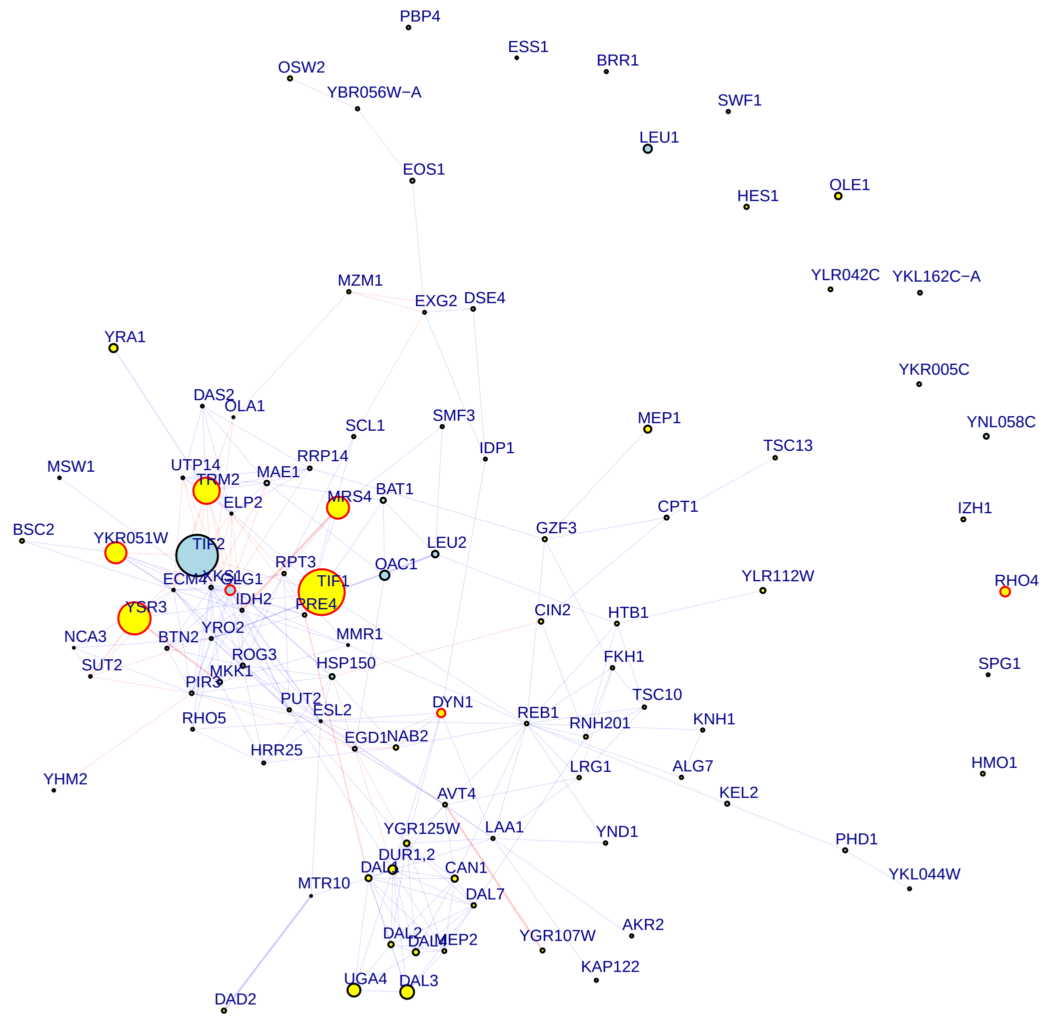

chrXI:641098_A/G top 50 up genes

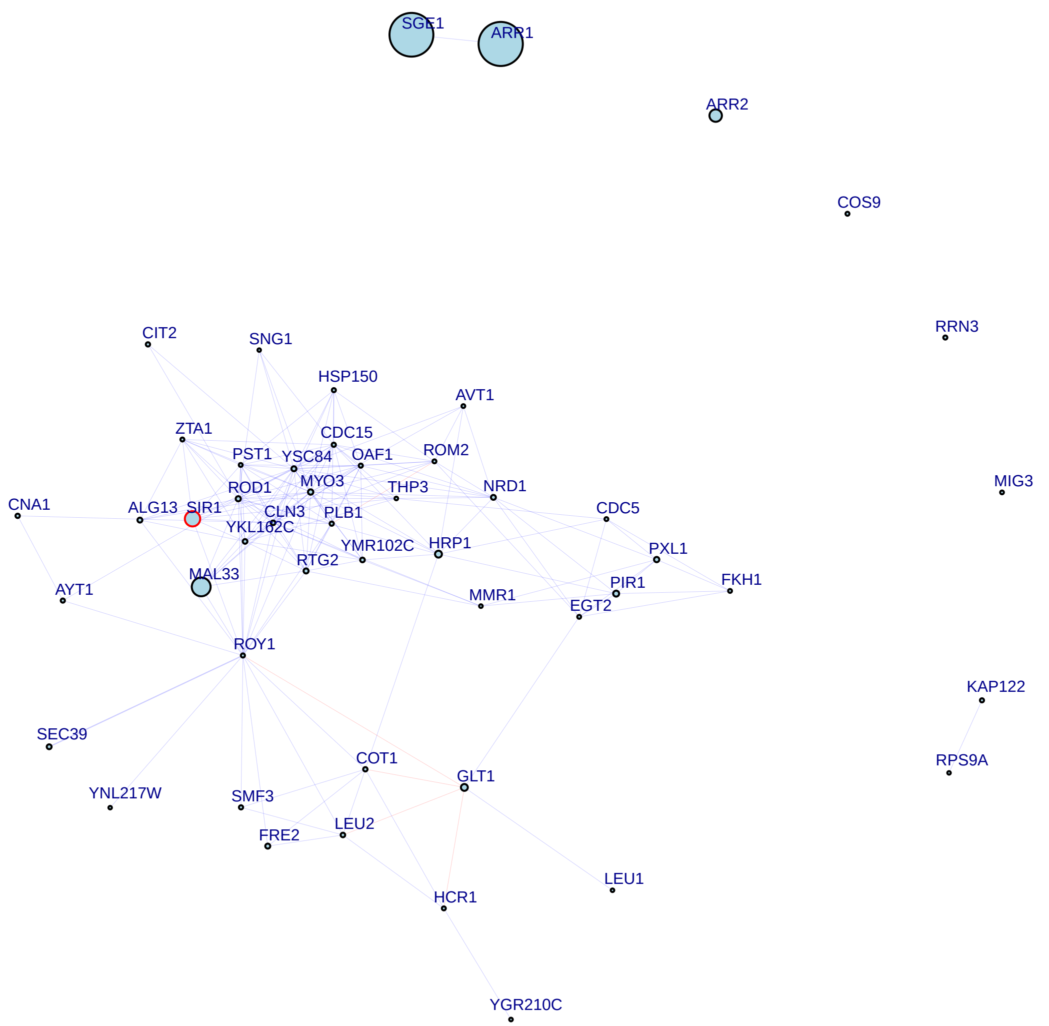

chrXI:641098_A/G top 45 down genes

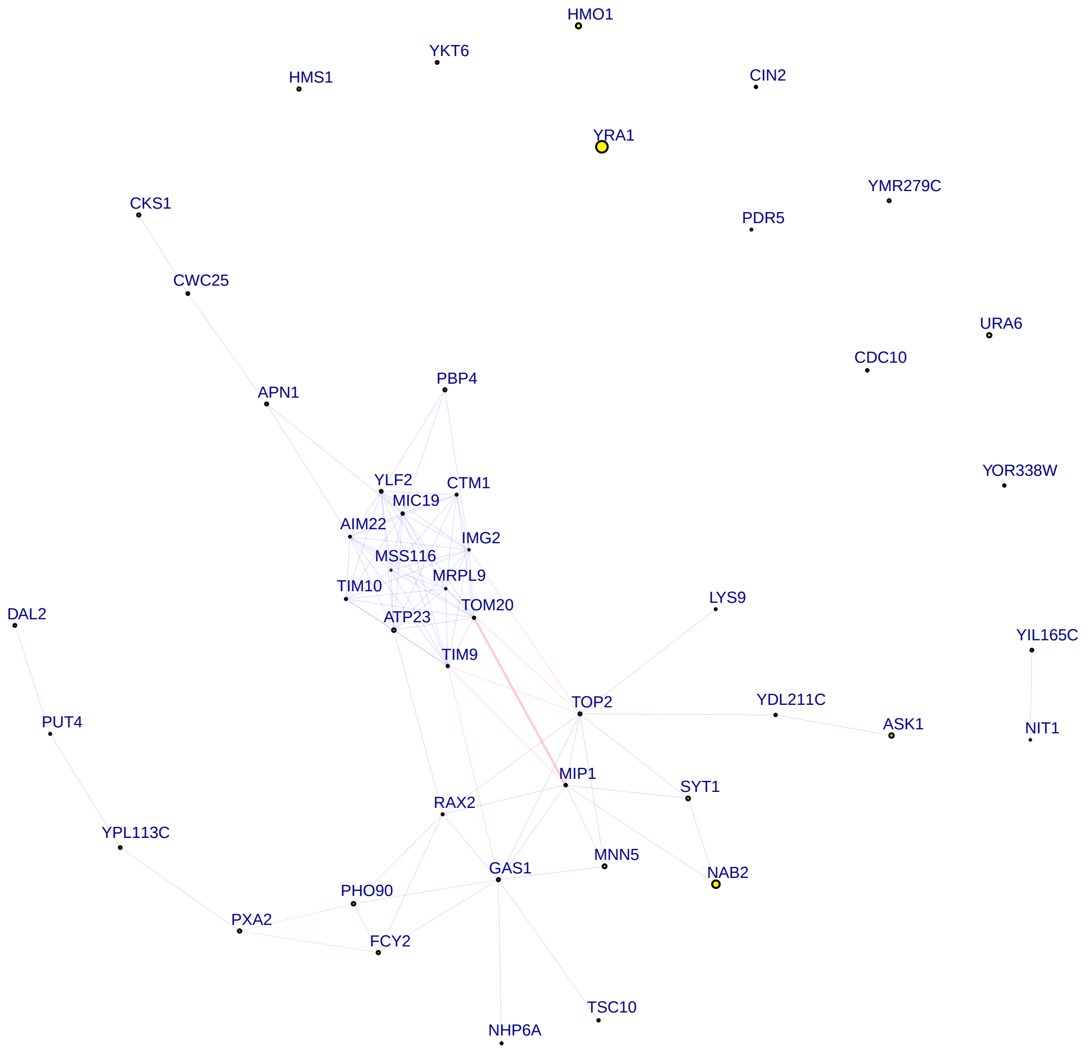

chrXI:641098_A/G top 95 up and down genes

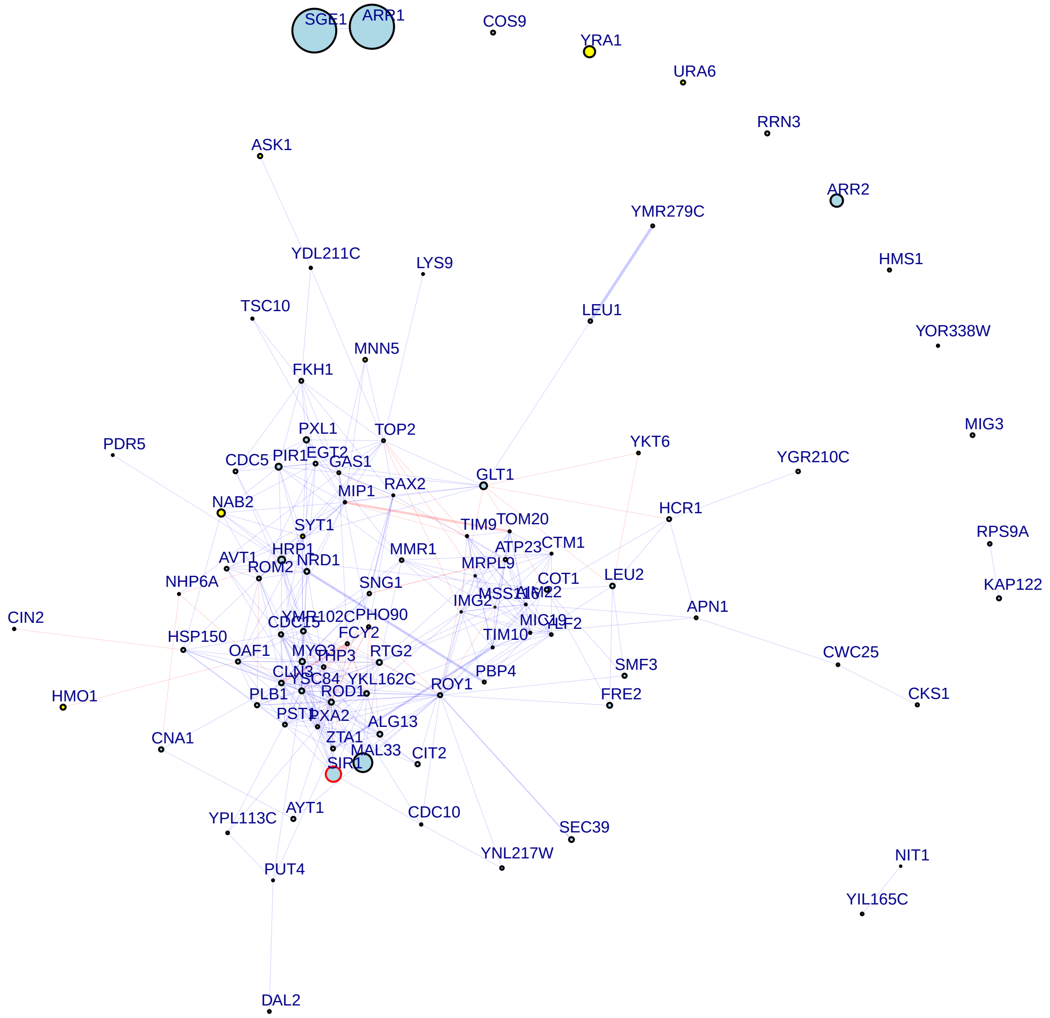

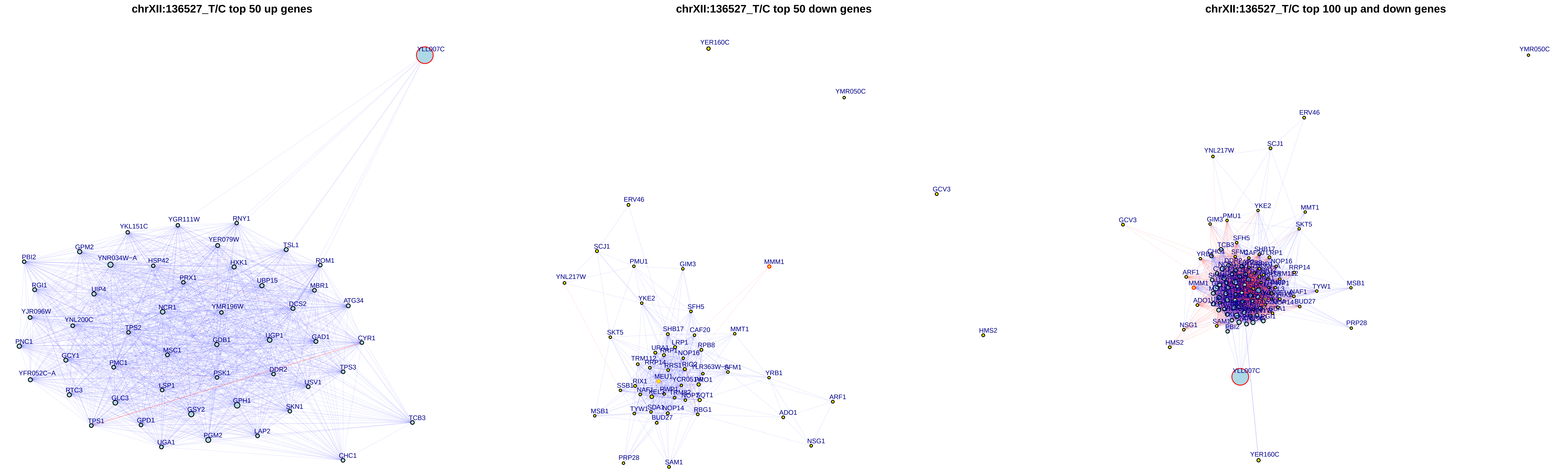
chrXII:136527_T/C top 50 up genes
YLL007C
YKL151C
YGR111W
RNY1
YER079W
TSL1
ROM1
PBI2
GPM2
YNR034W−A
HSP42
HXK1
PRX1
UBP15
MBR1
RGI1
UIP4
ATG34
NCR1
YMR196W
DCS2
YJR096W
YNL200C
TPS2
CDB1
UGP1
GAD1
CYR1
PNC1
GCY1
MSC1
PMC1
PSK1
DDR2
TPS3
YFR052C−A
USV1
RTC3
LSP1
GLC3
GPH1
GSY2
SKN1
TPS1
GPD1
LAP2
TCB3
UGA1
PGM2
CHC1
chrXII:136527_T/C top 50 down genes
YER160C
YMR050C
GCV3
ERV46
SCJ1
PMU1
GIM3
MMM1
YNL217W
YKE2
SFH5
SKT5
SHB17
CAF20
MMT1
HMS2
LRP1
RPB8
NOP16
TRM112
RRP14
YLR363W−A
SFM1
YRB1
RIX1
MEU1
SSB1
NAF1
TRM82
NOP14
SDA1
TYW1
MSB1
BUD27
RBG1
ADO1
ARF1
NSG1
PRP28
SAM1
chrXII:136527_T/C top 100 up and down genes
YMR050C
ERV46
SCJ1
YNL217W
YKE2
MMT1
GCV3
GIM3
PMU1
SKT5
SFH5
TCB3
SHB17
LRP1
NOP16
RRP14
ARF1
MMM1
TYW1
MSB1
NAF1
BUD27
ADO1
NSG1
PBI2
PRP28
HMS2
YLL007C
YER160C

chrXII:510559_C/T top 50 up genes

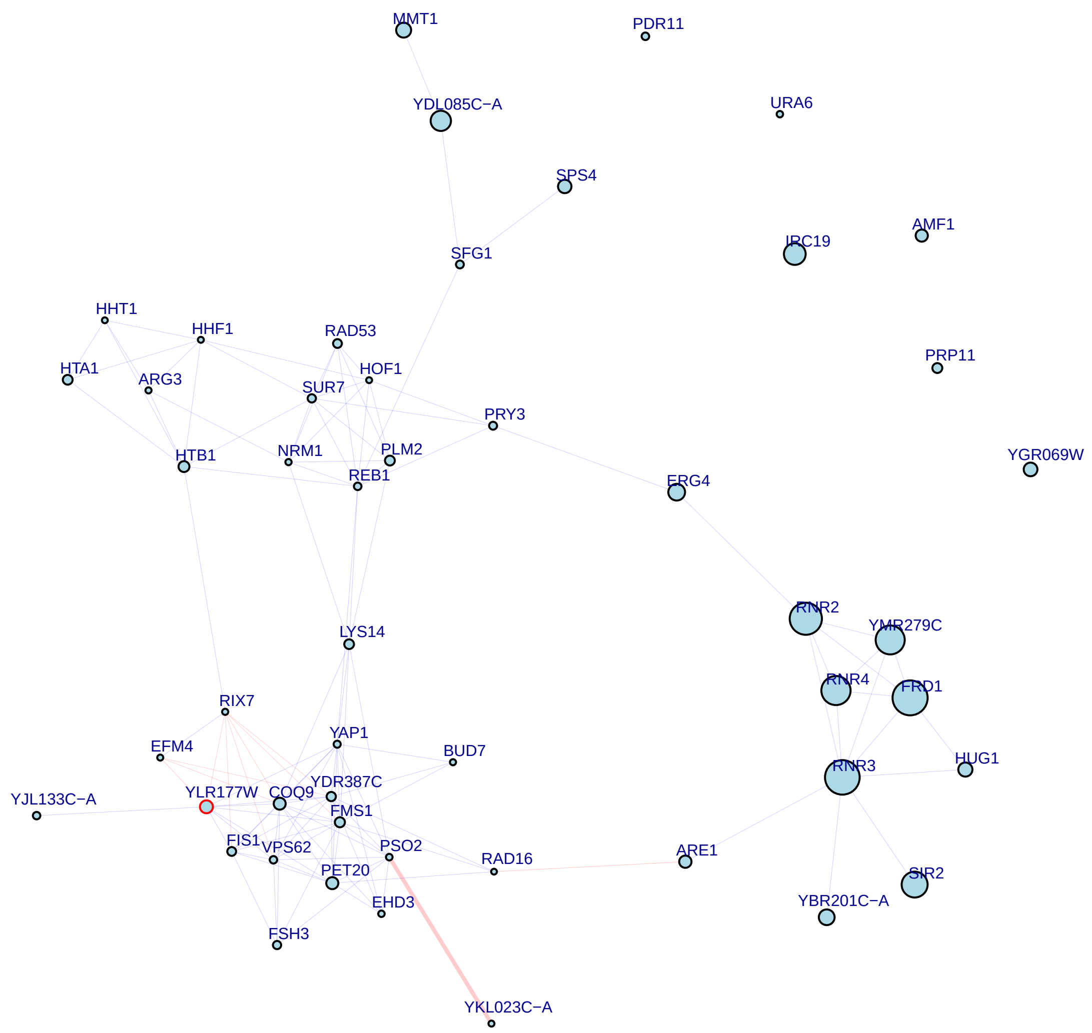

chrXII:510559_C/T top 50 down genes

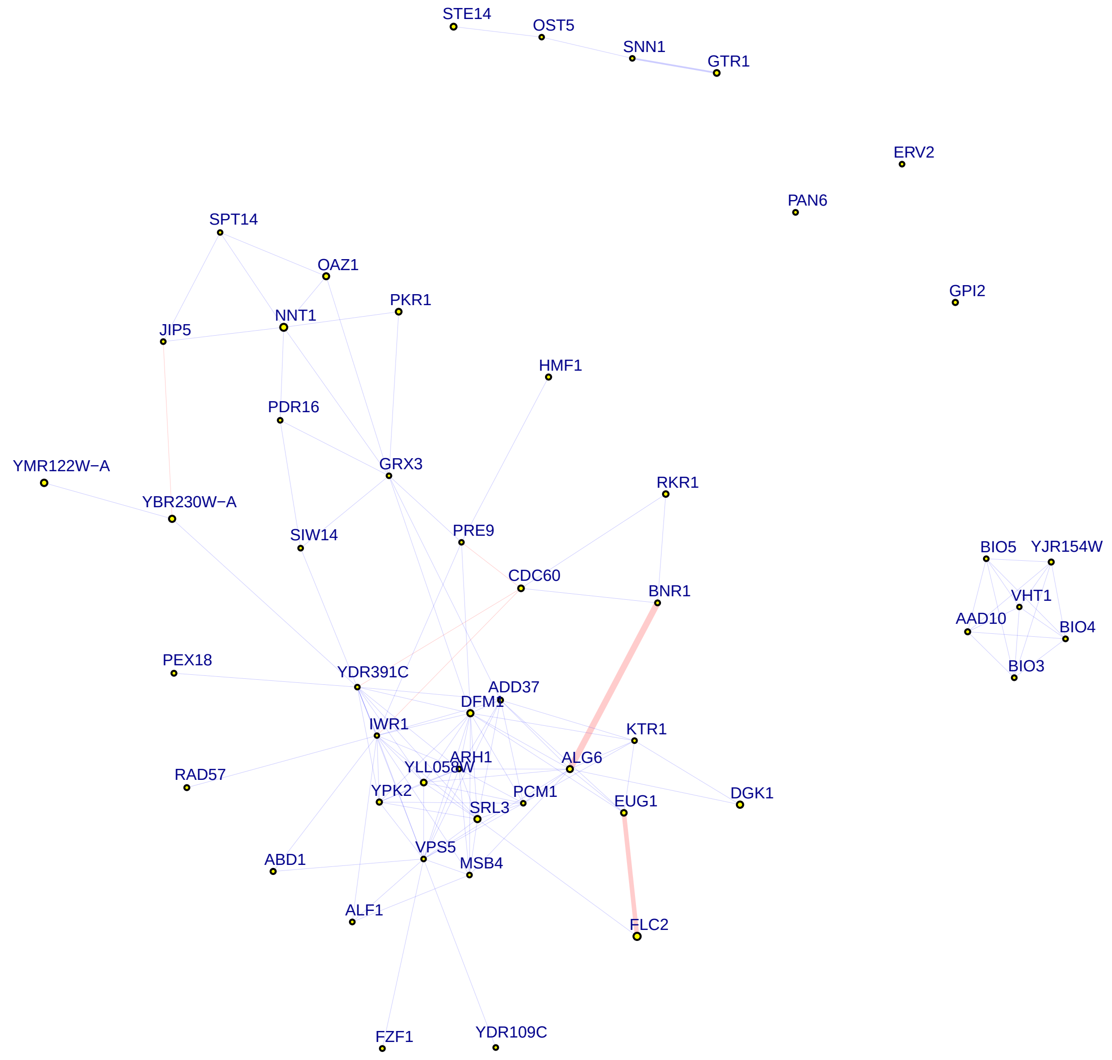

chrXII:510559_C/T top 100 up and down genes

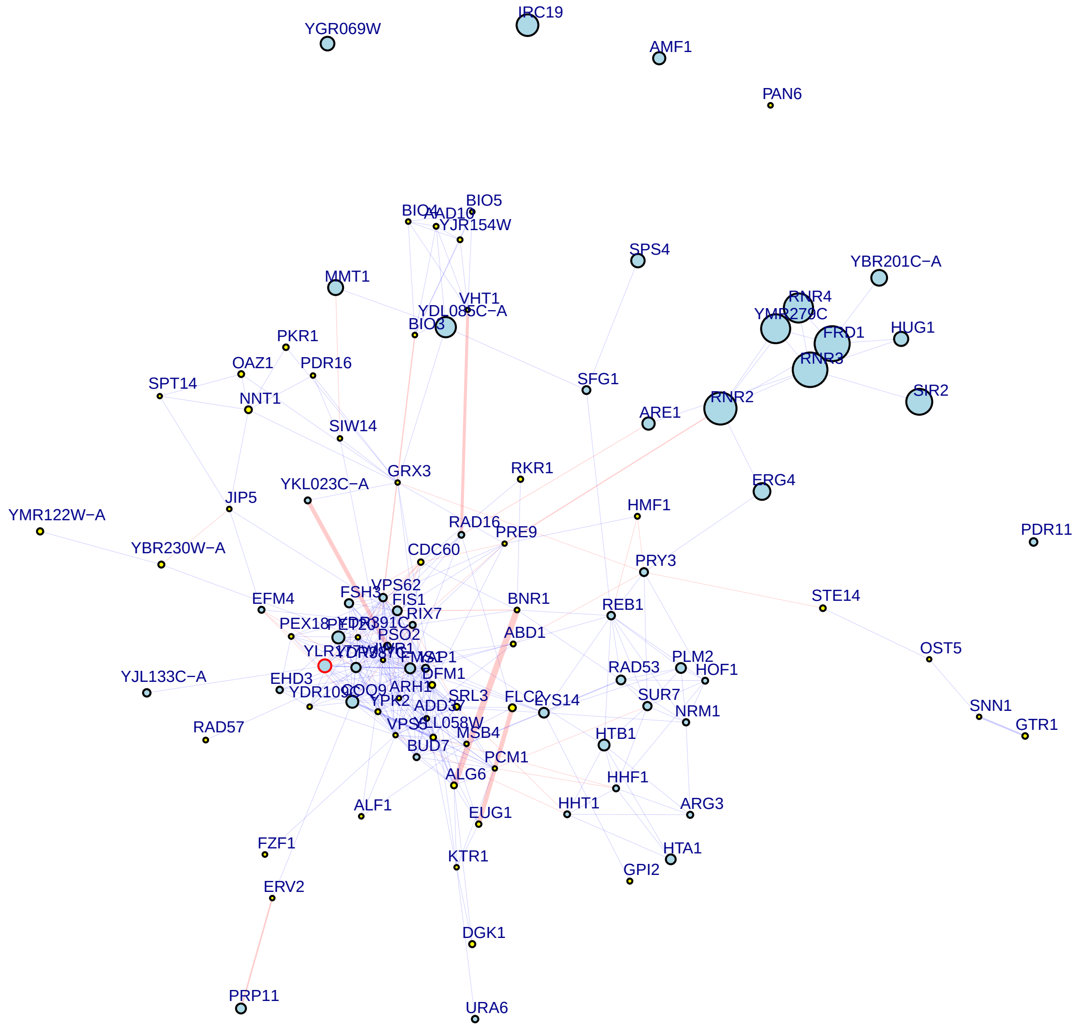

chrXII:657022_T/C top 50 up genes

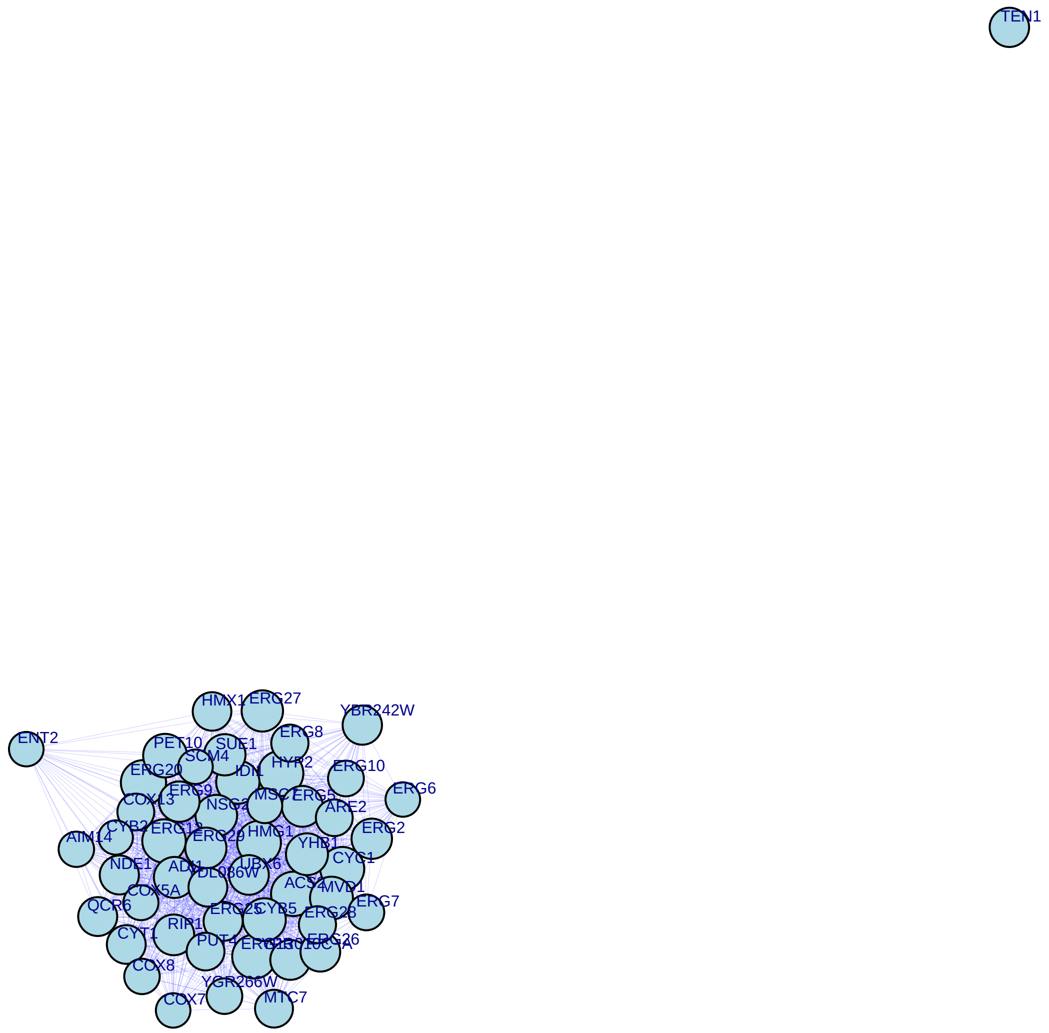

chrXII:657022_T/C top 50 down genes

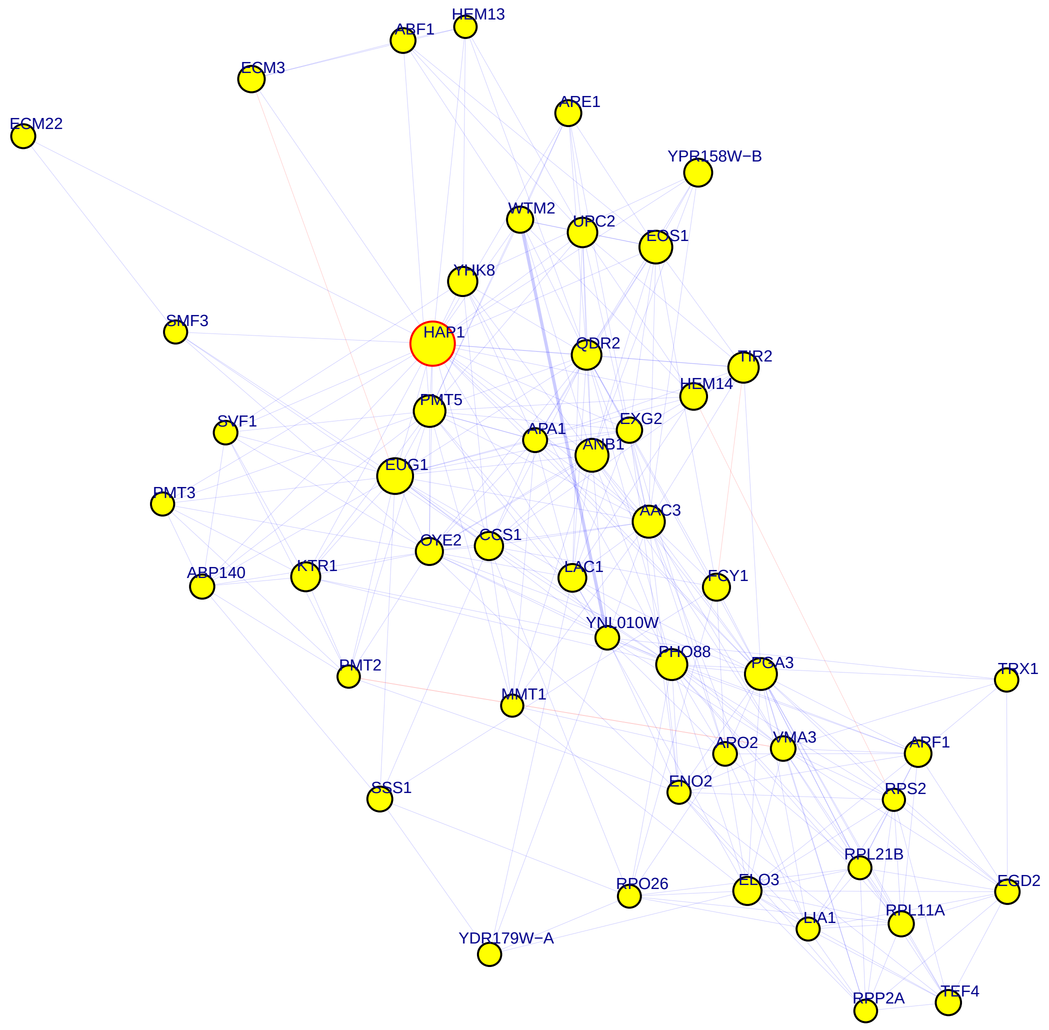

chrXII:657022_T/C top 100 up and down genes

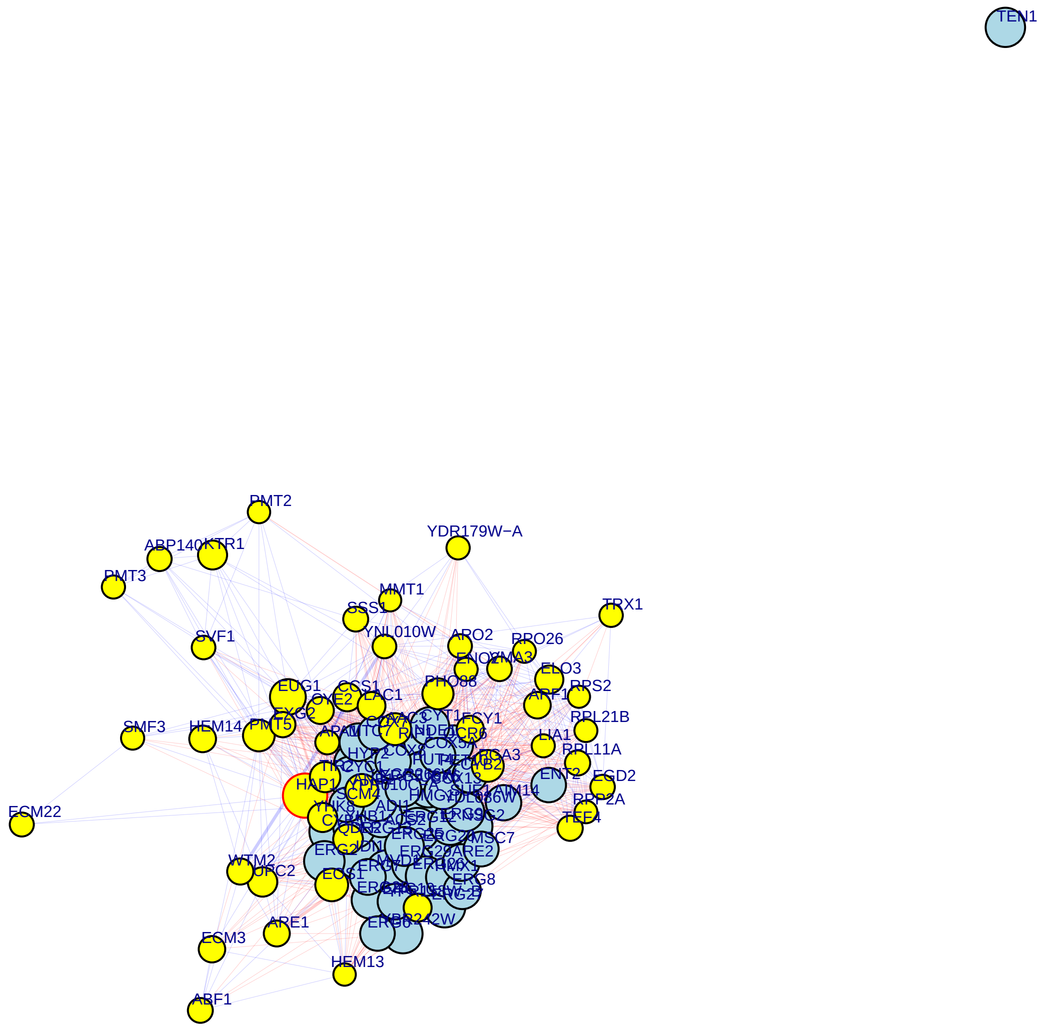

chrXII:694841_T/C top 50 up genes

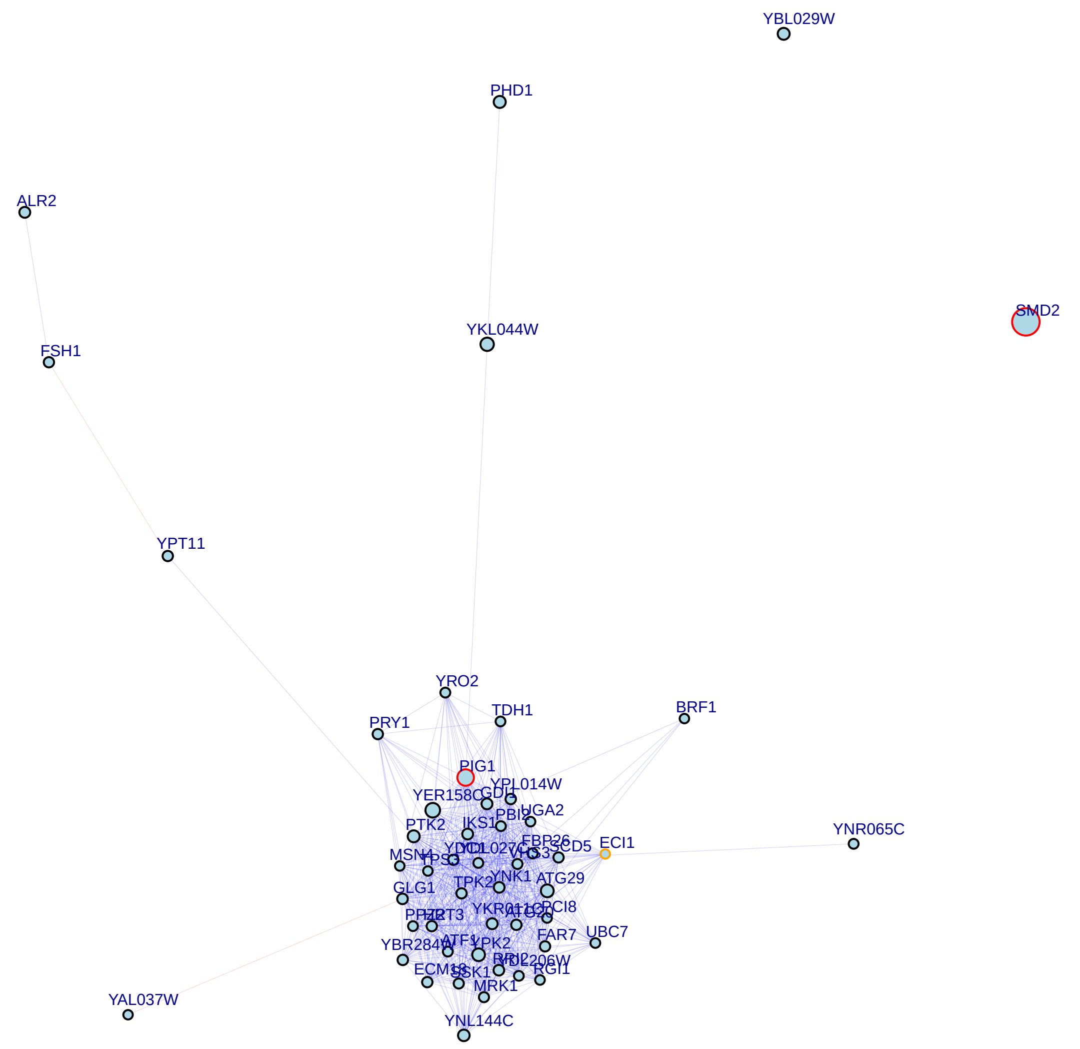

chrXII:694841_T/C top 50 down genes

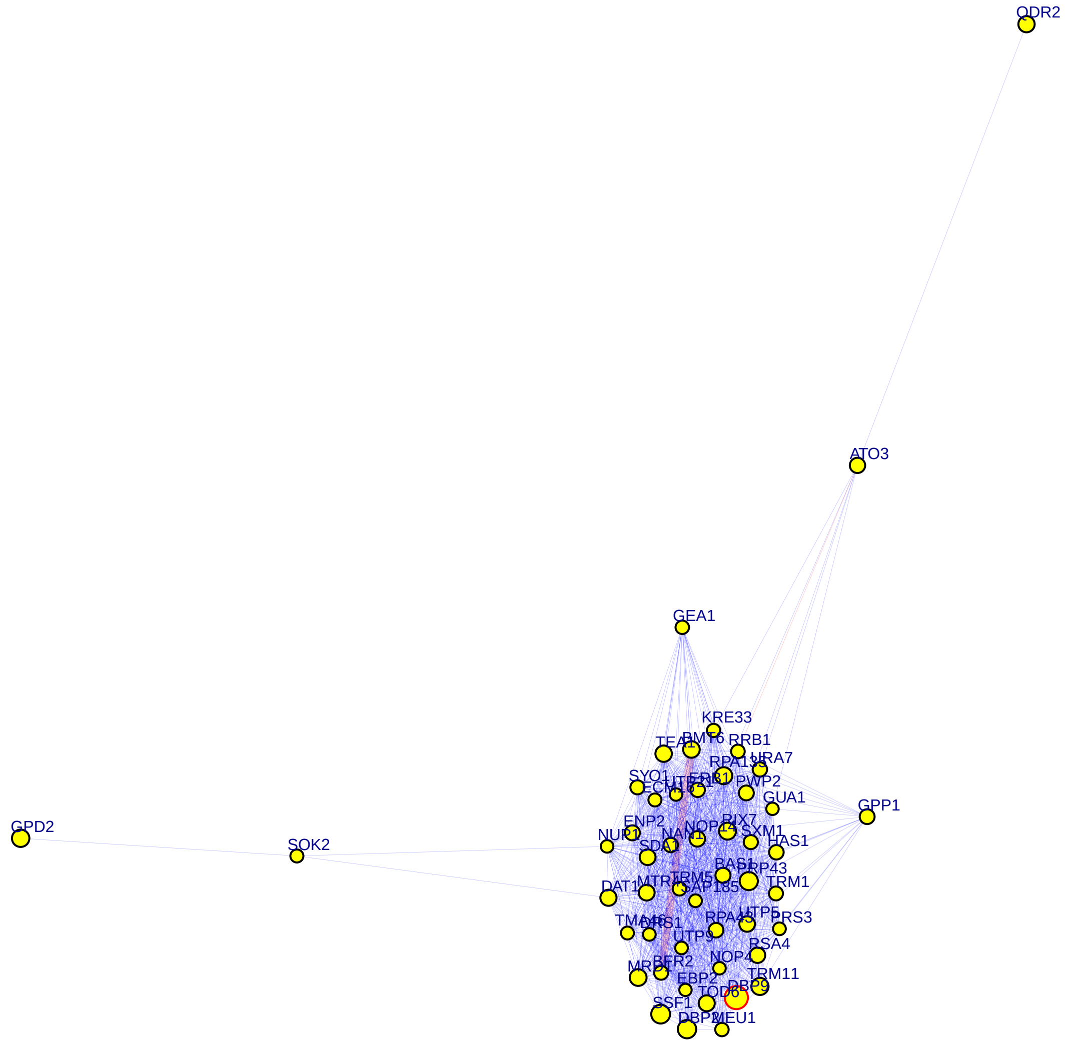

chrXII:694841_T/C top 100 up and down genes

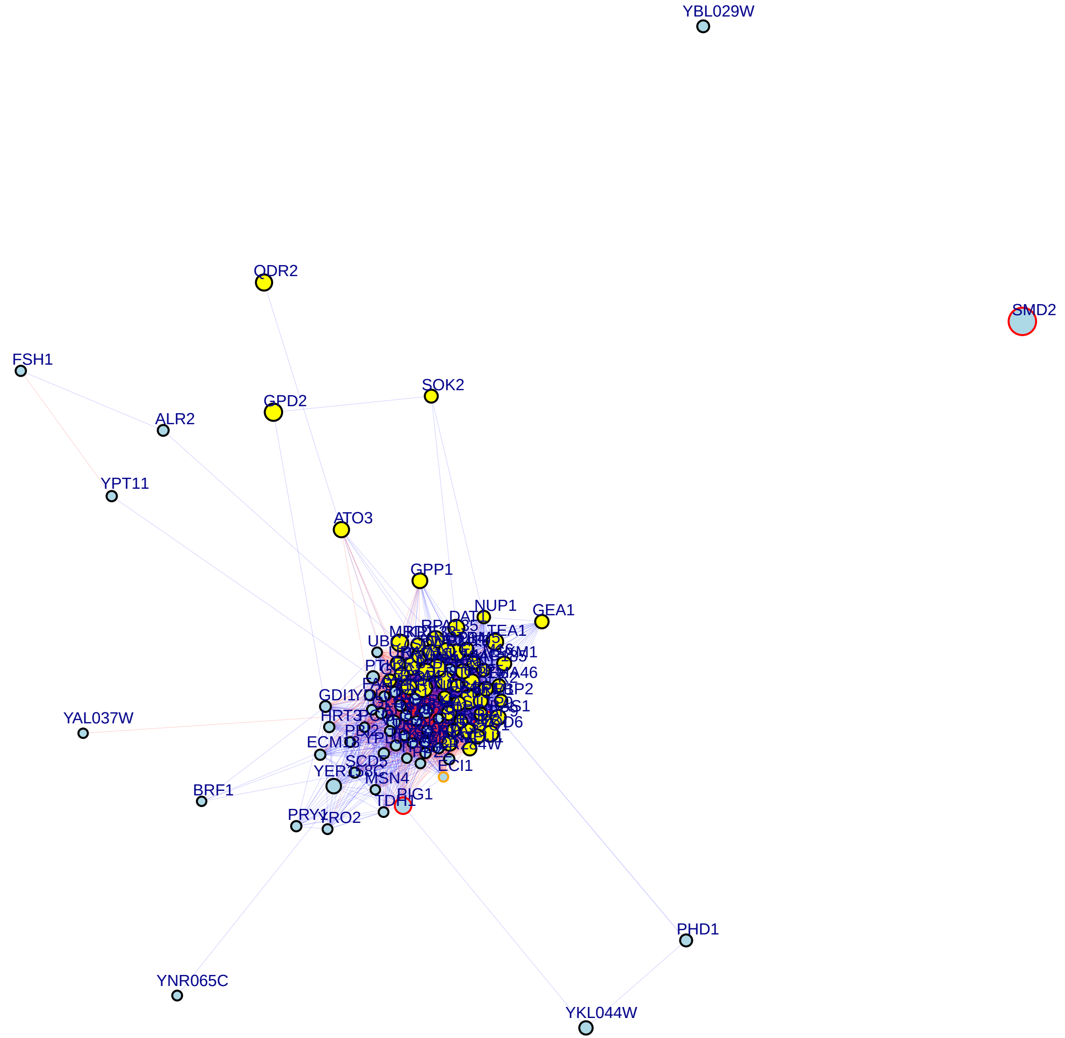

chrXII:848842_A/G top 50 up genes

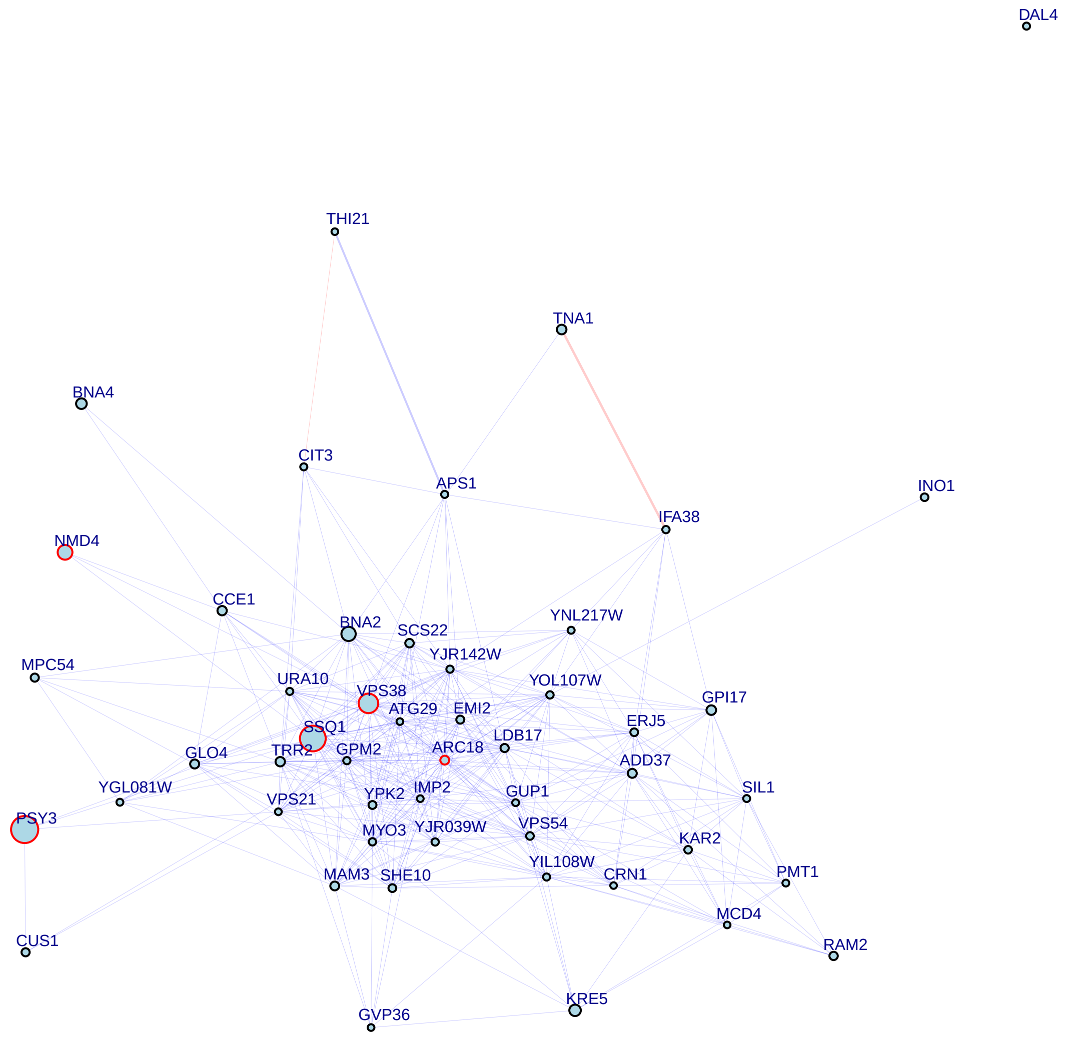

chrXII:848842_A/G top 50 down genes

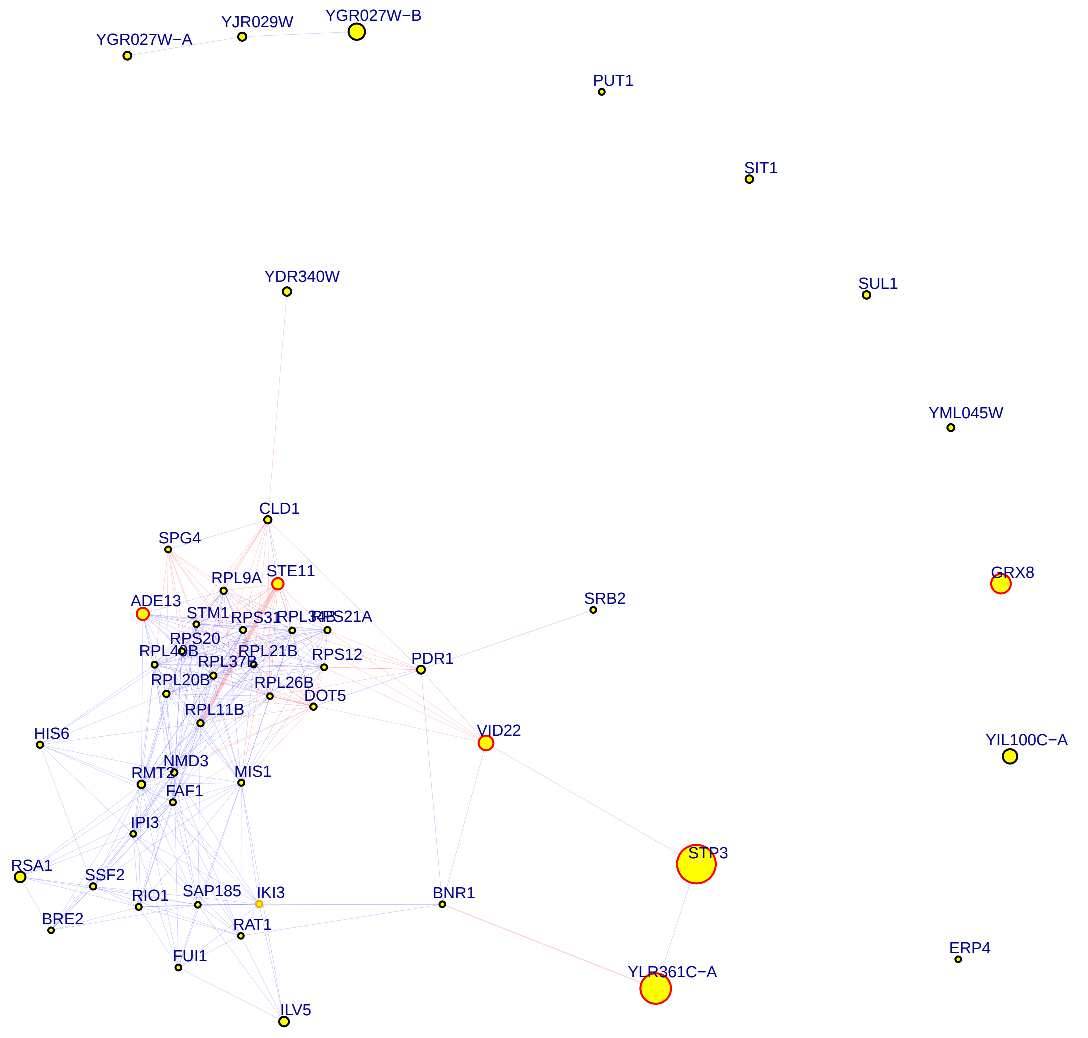

chrXII:848842_A/G top 100 up and down genes

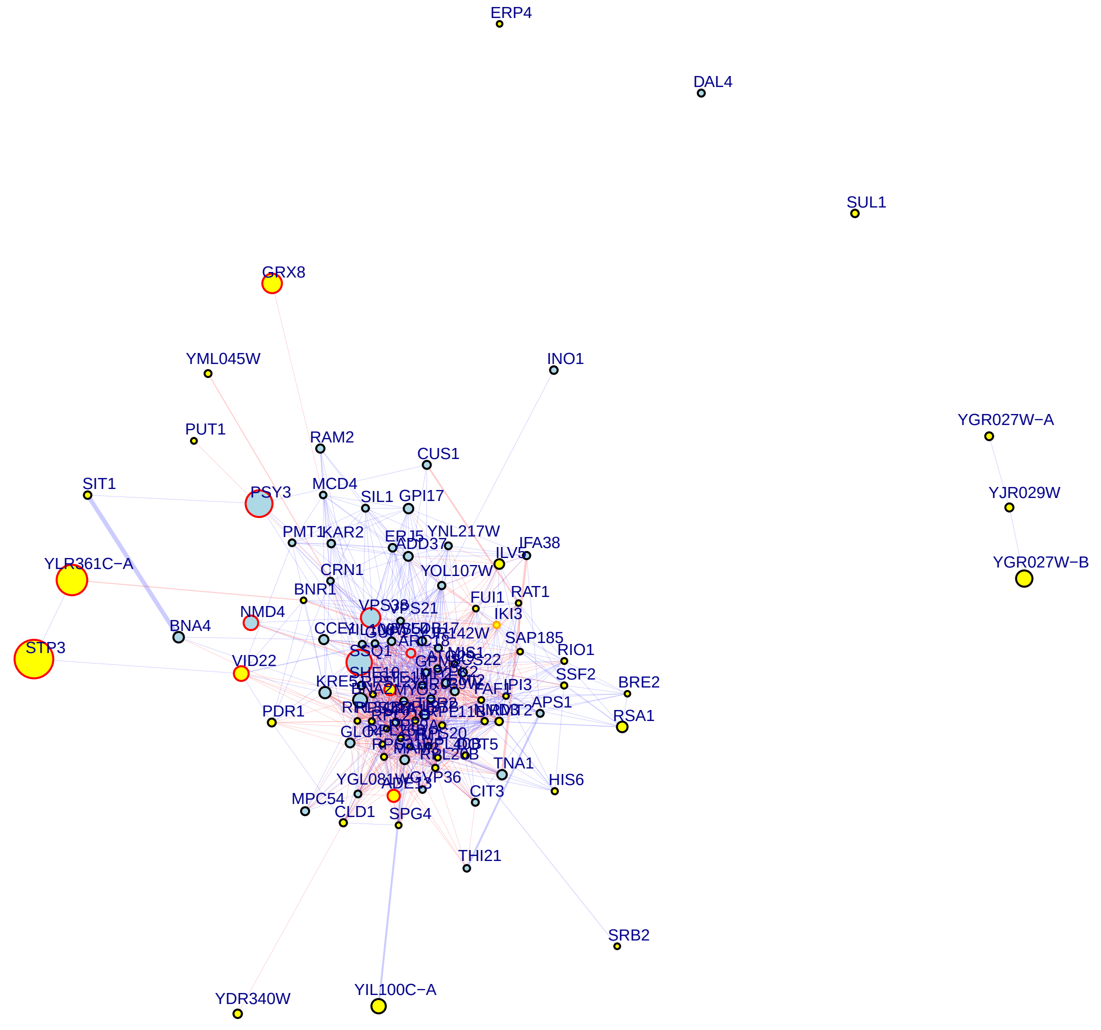

chrXII:1058607_C/T top 50 up genes

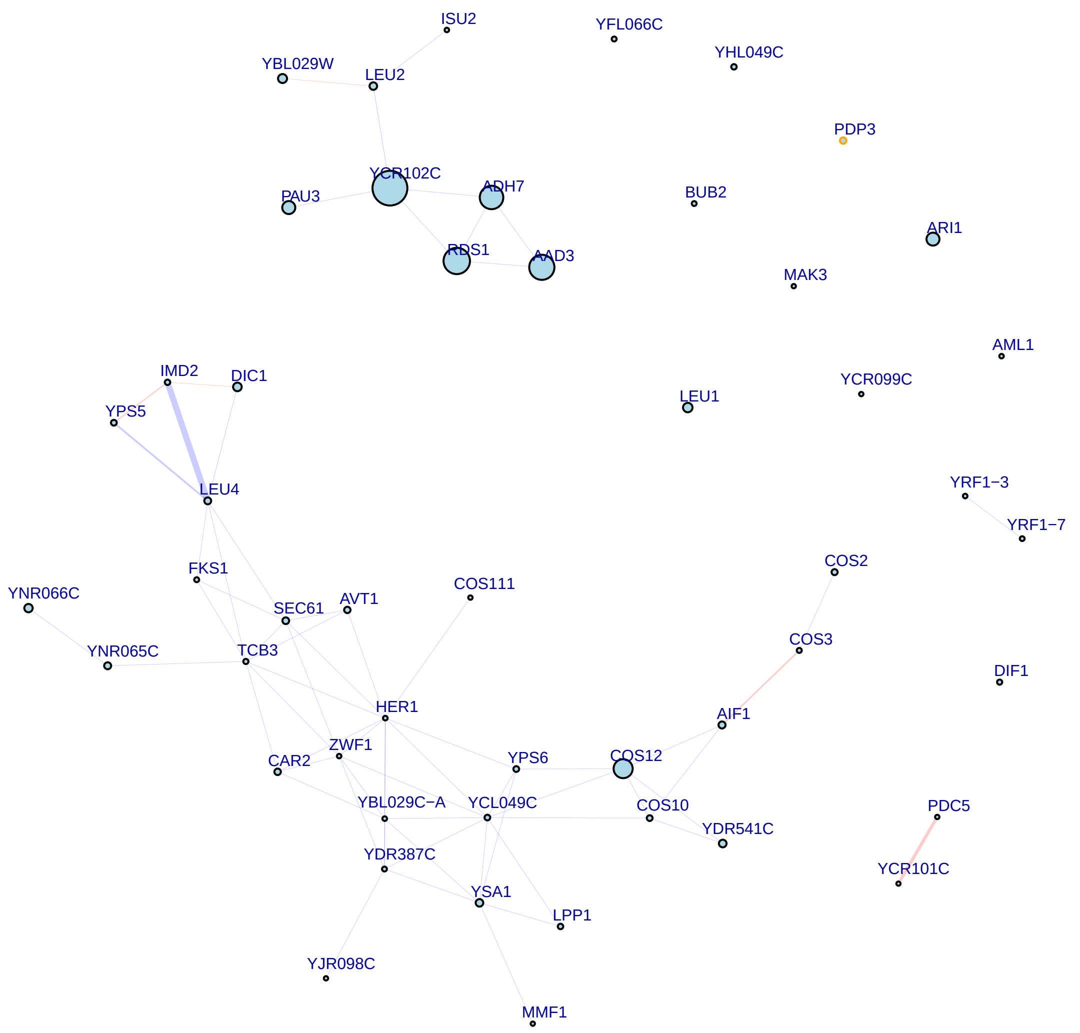

chrXII:1058607_C/T top 50 down genes

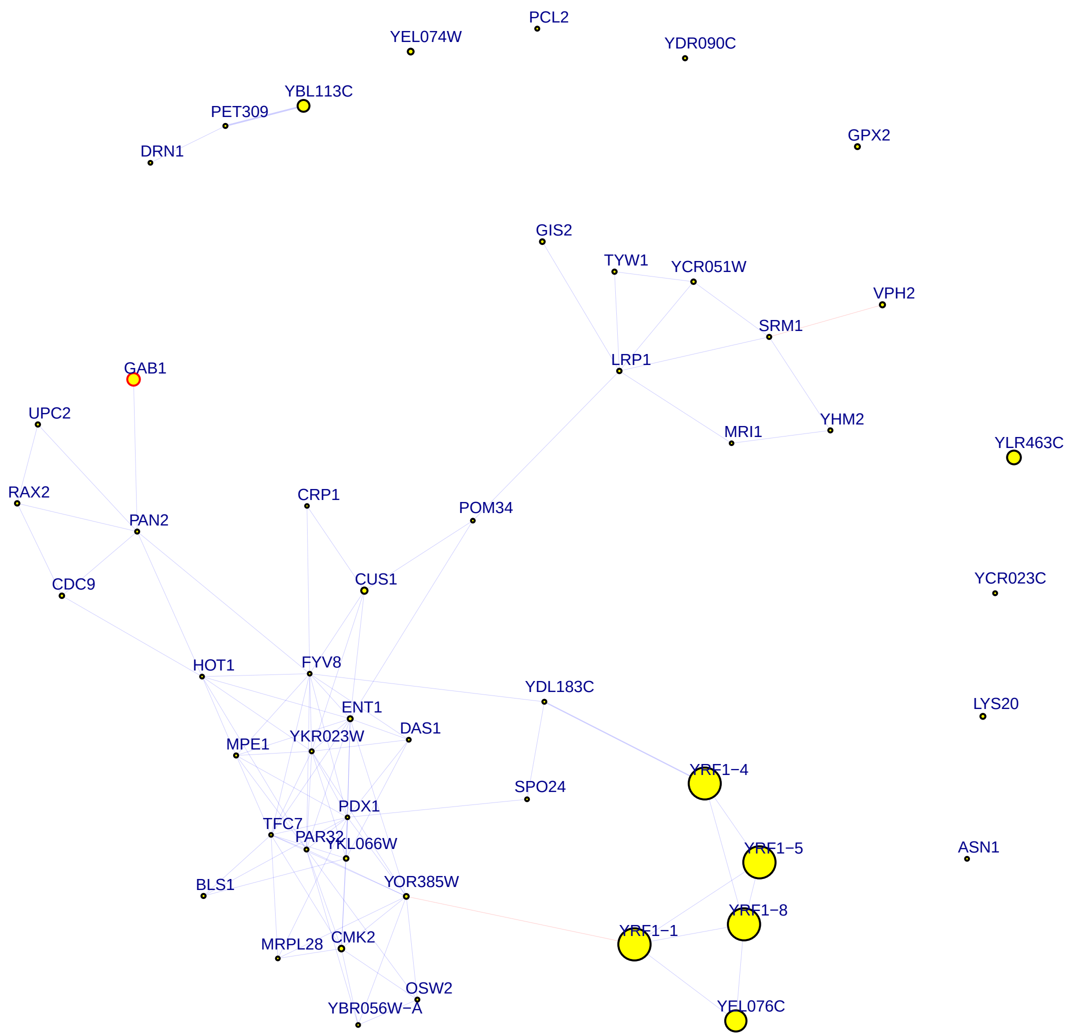

chrXII:1058607_C/T top 100 up and down genes

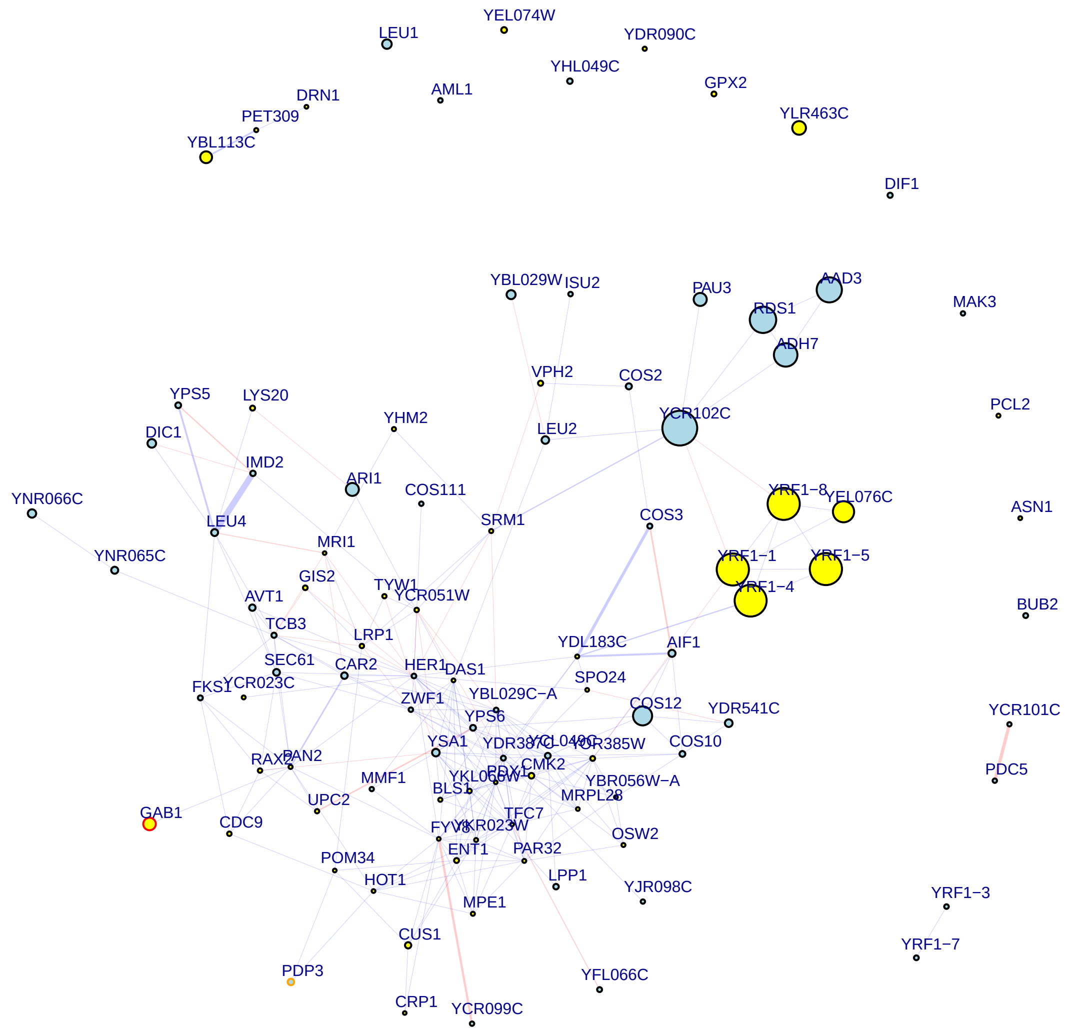

chrXIII:33836_G/A top 50 up genes

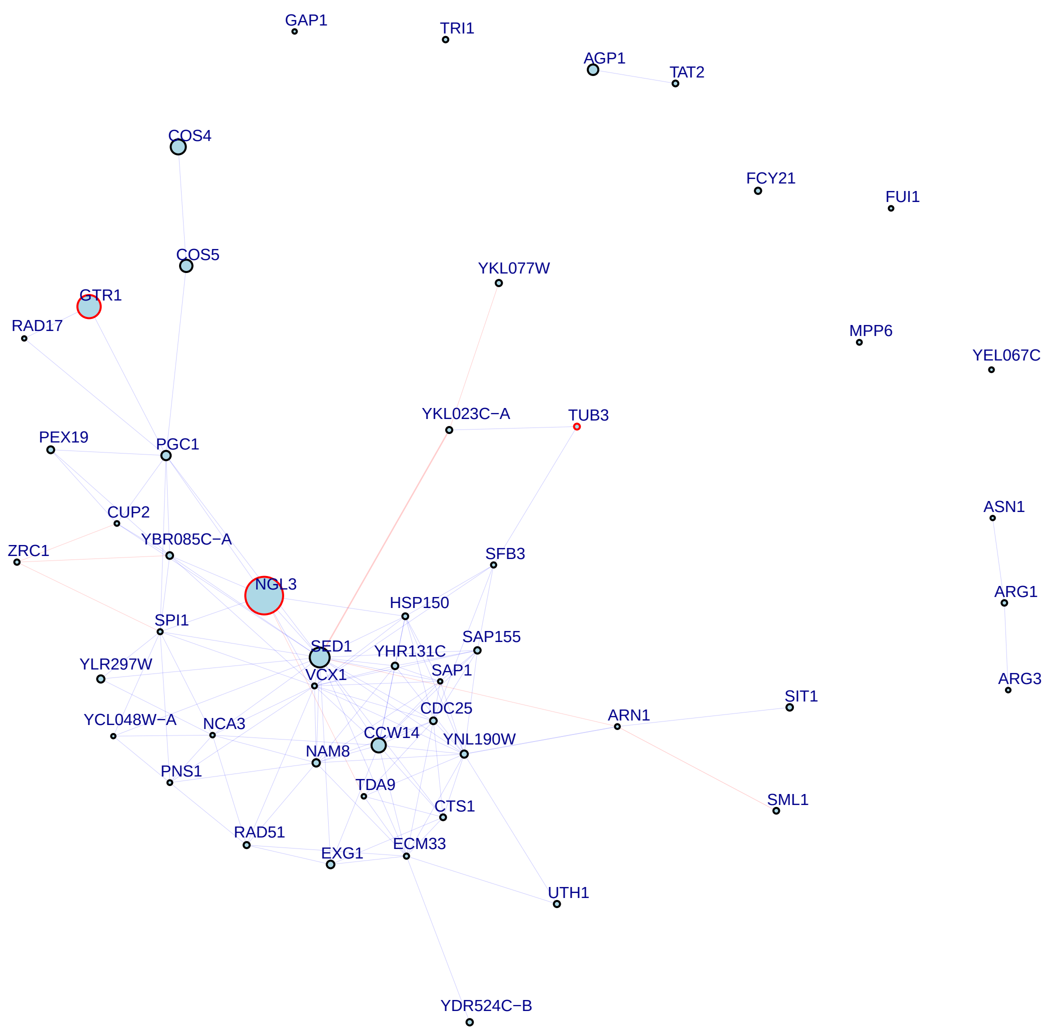

chrXIII:33836_G/A top 50 down genes

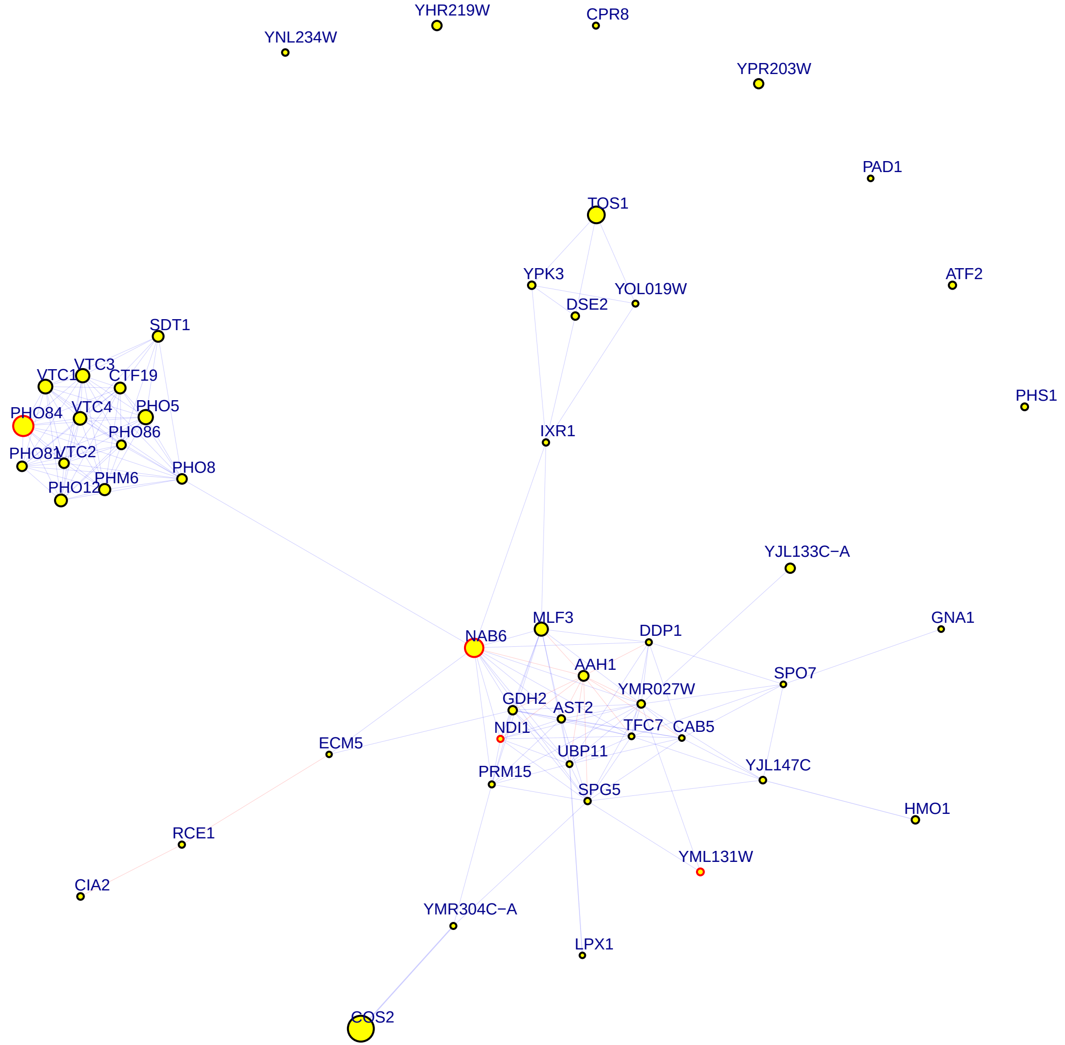

chrXIII:33836_G/A top 100 up and down genes

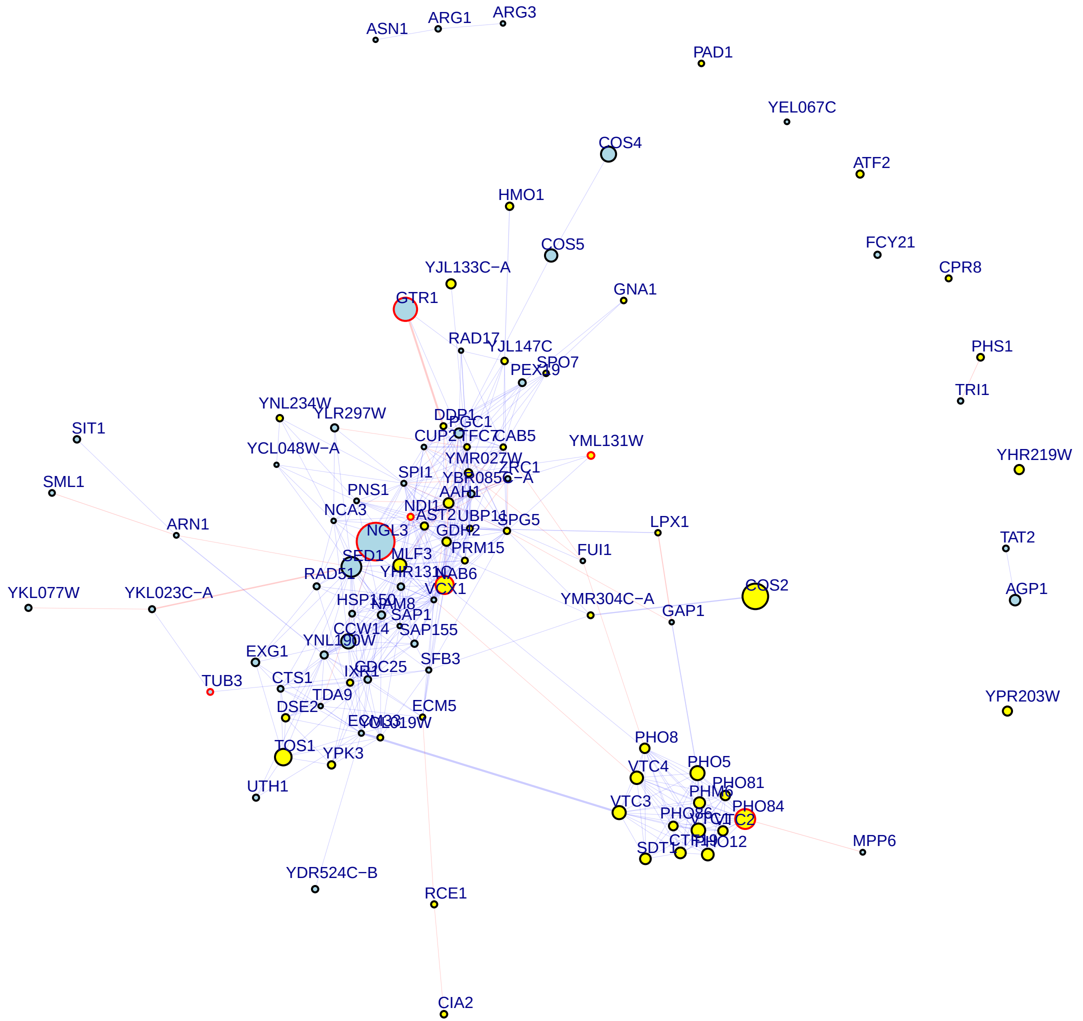

chrXIII:85889_GA/G top 50 up genes

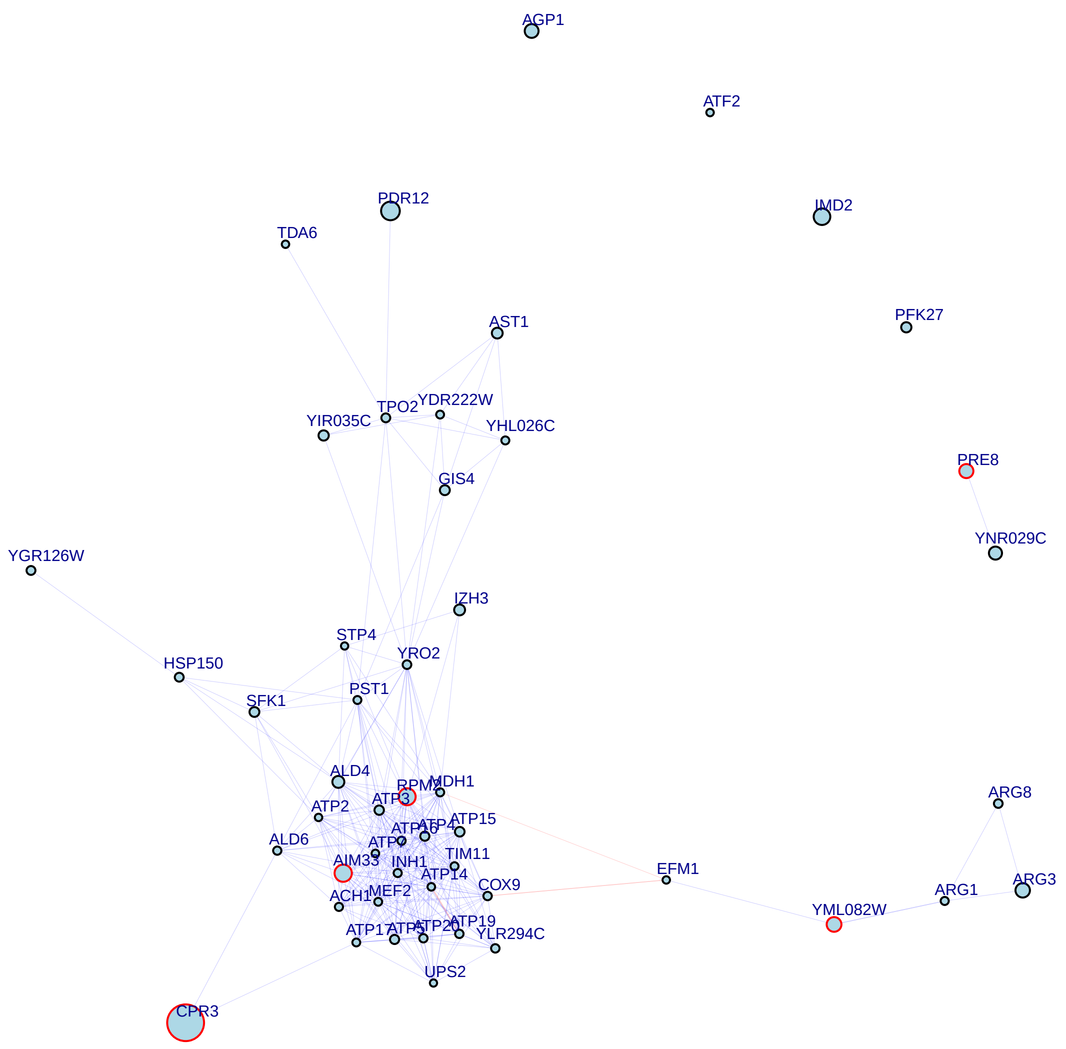

chrXIII:85889_GA/G top 50 down genes

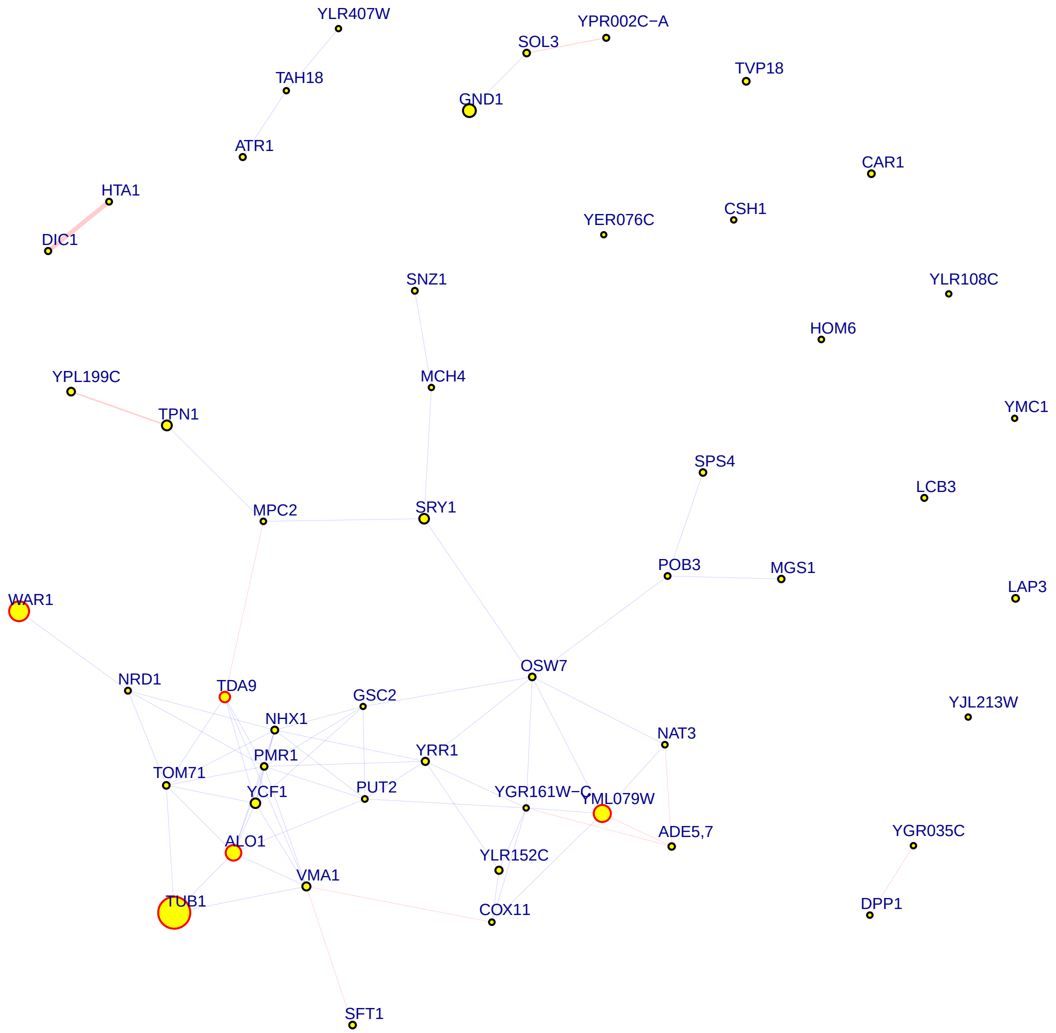

chrXIII:85889_GA/G top 100 up and down genes

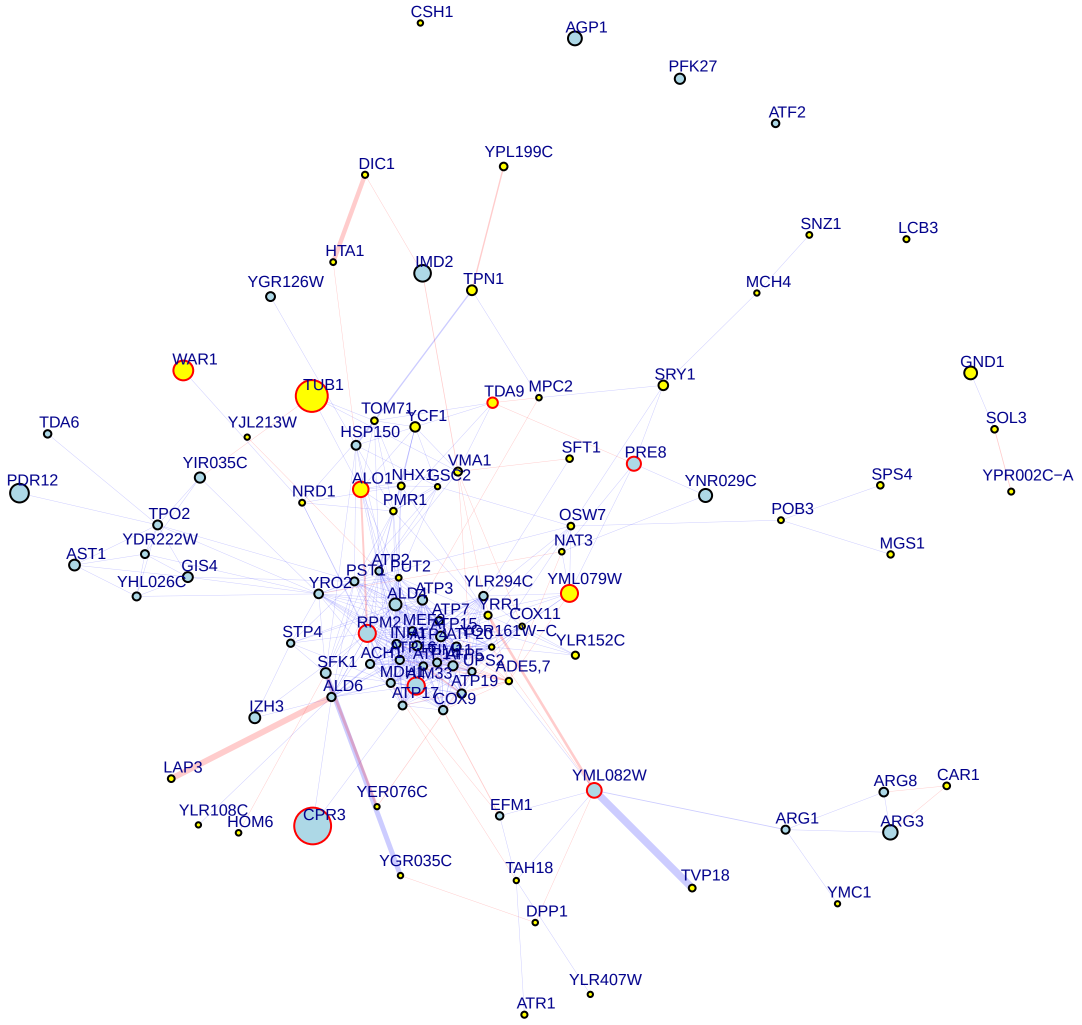

chrXIII:253667_T/C top 50 up genes

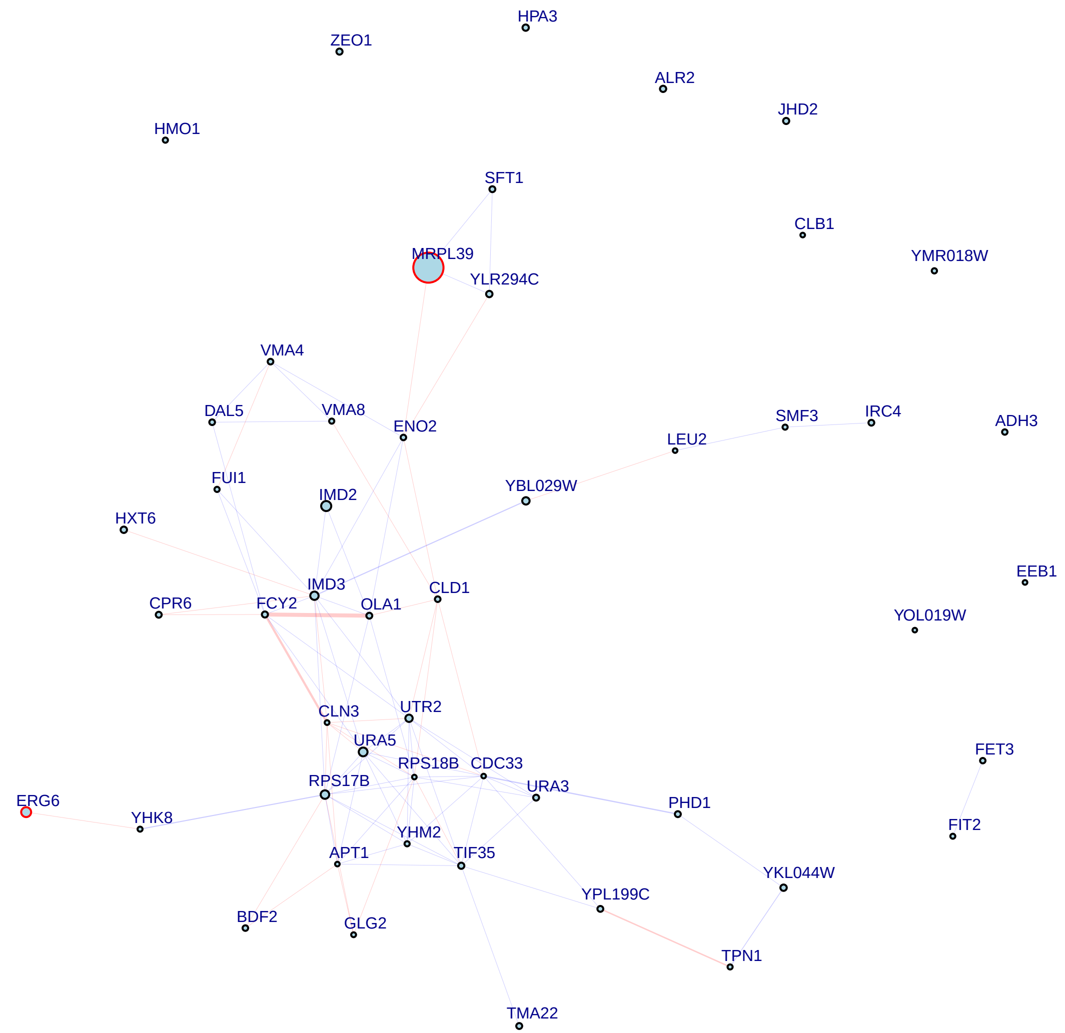

chrXIII:253667_T/C top 50 down genes

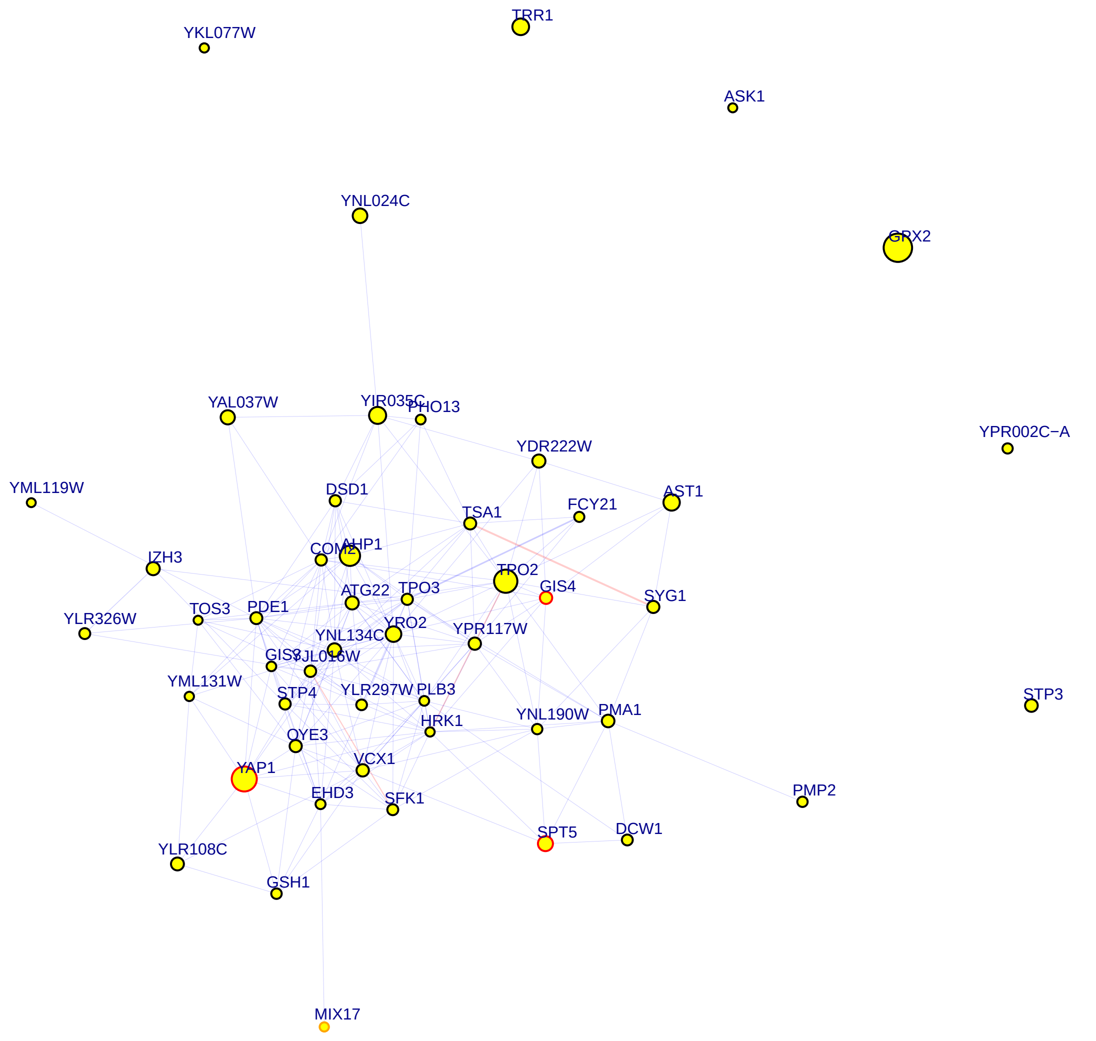

chrXIII:253667_T/C top 100 up and down genes

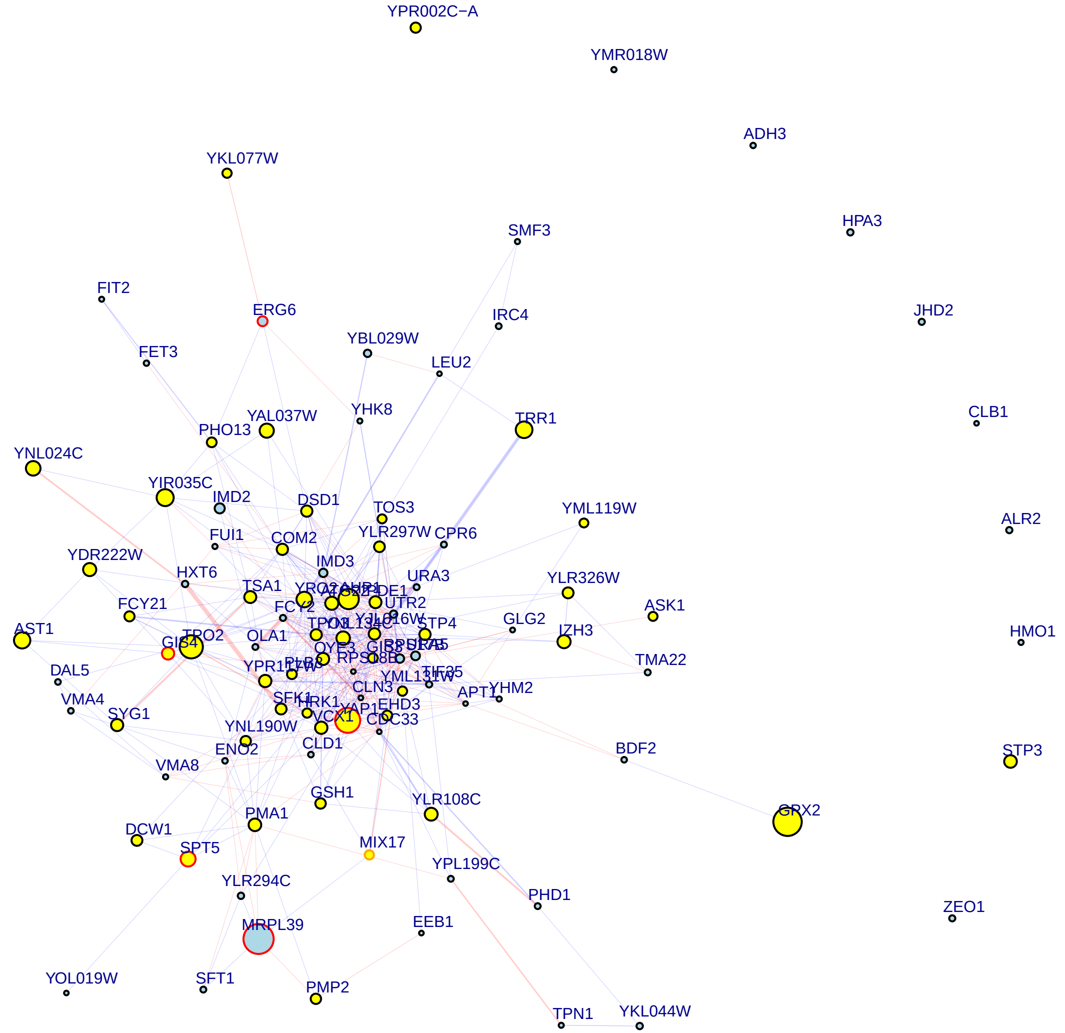

chrXIII:333449_G/A top 50 up genes

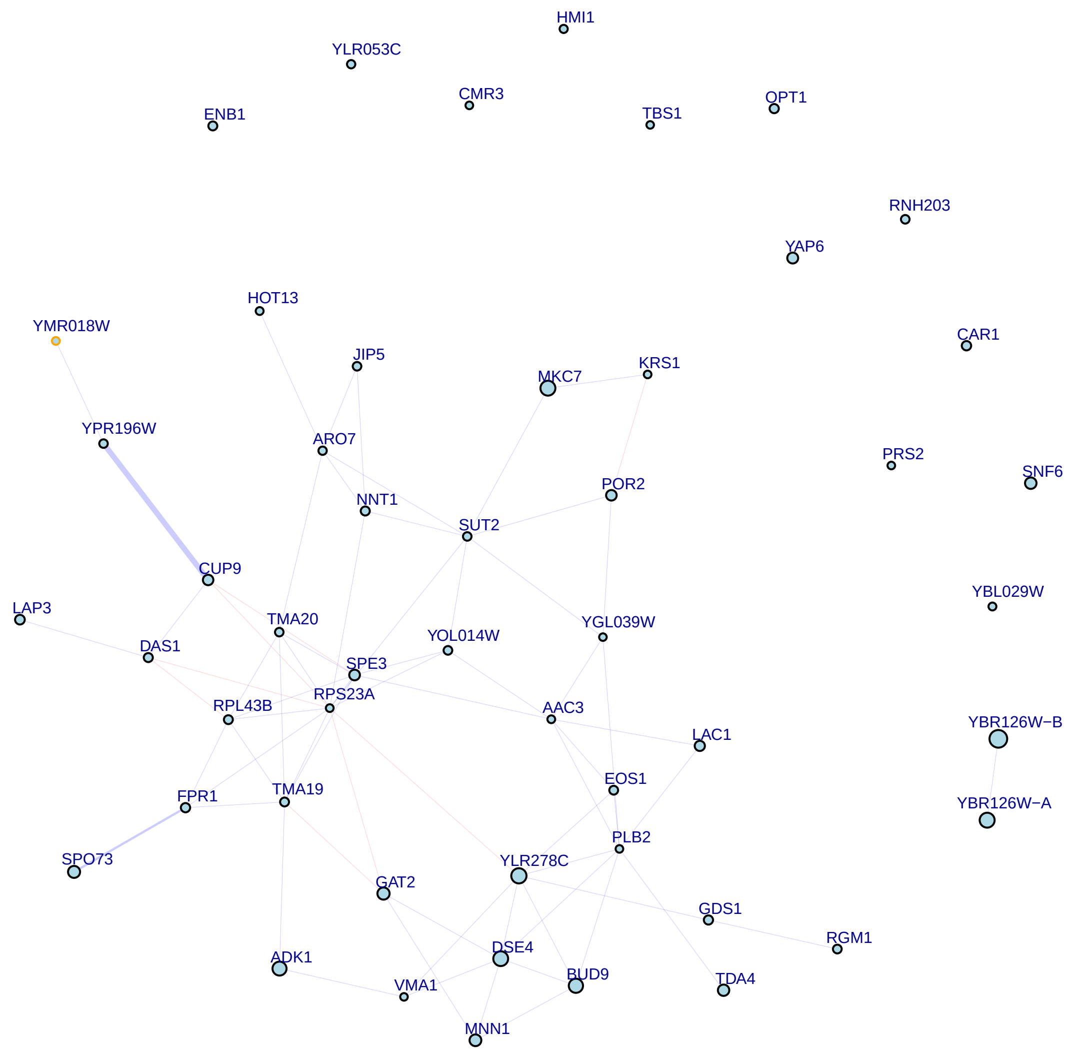

chrXIII:333449_G/A top 50 down genes

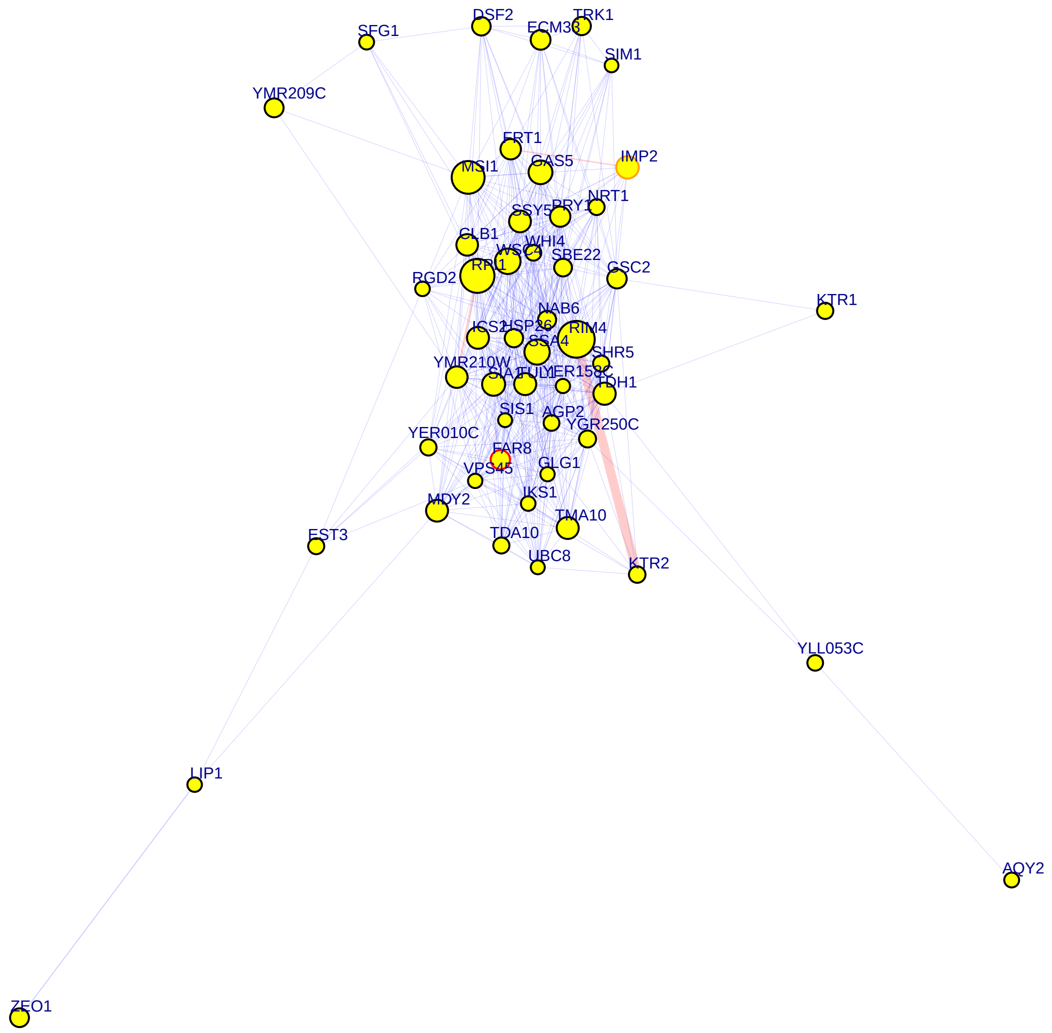

chrXIII:333449_G/A top 100 up and down genes

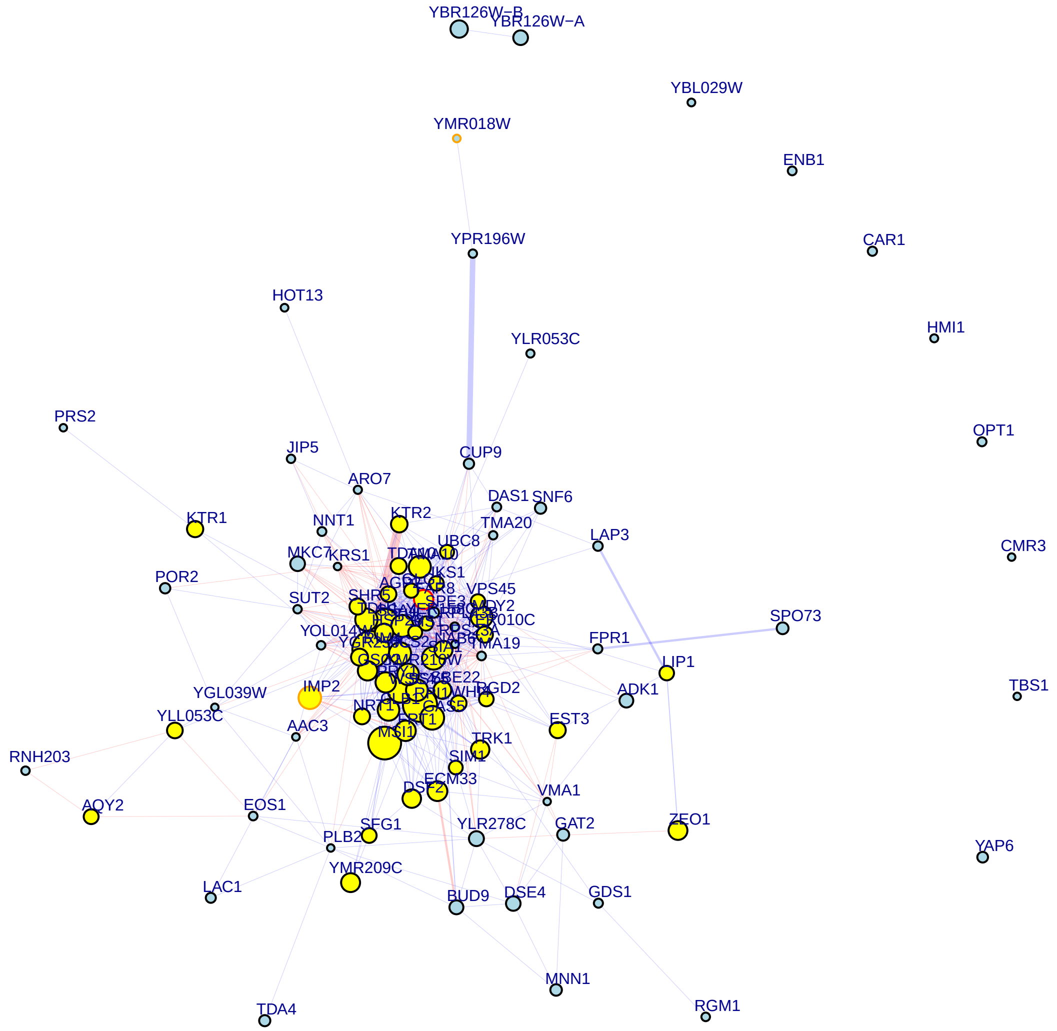

chrXIII:409778_G/T top 50 up genes

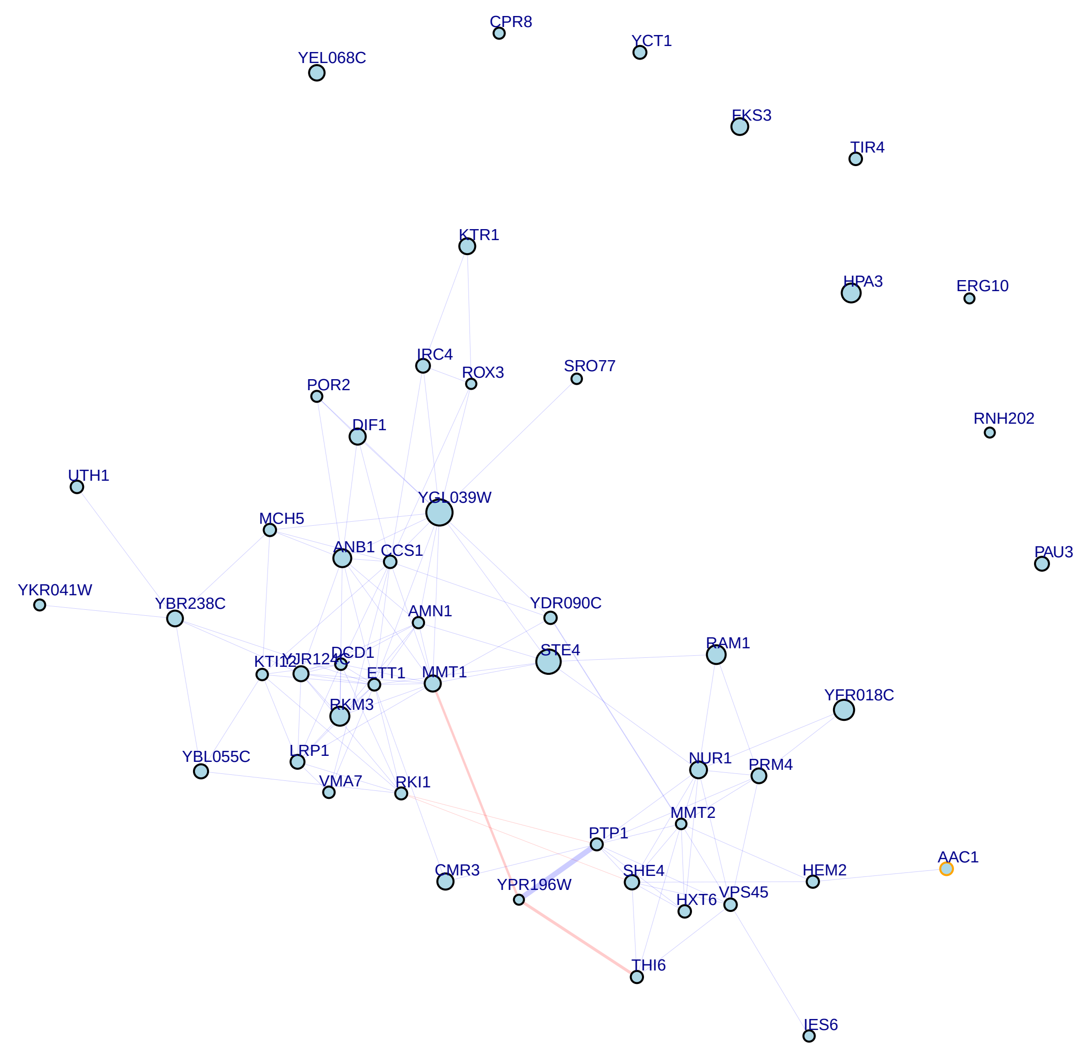

chrXIII:409778_G/T top 50 down genes

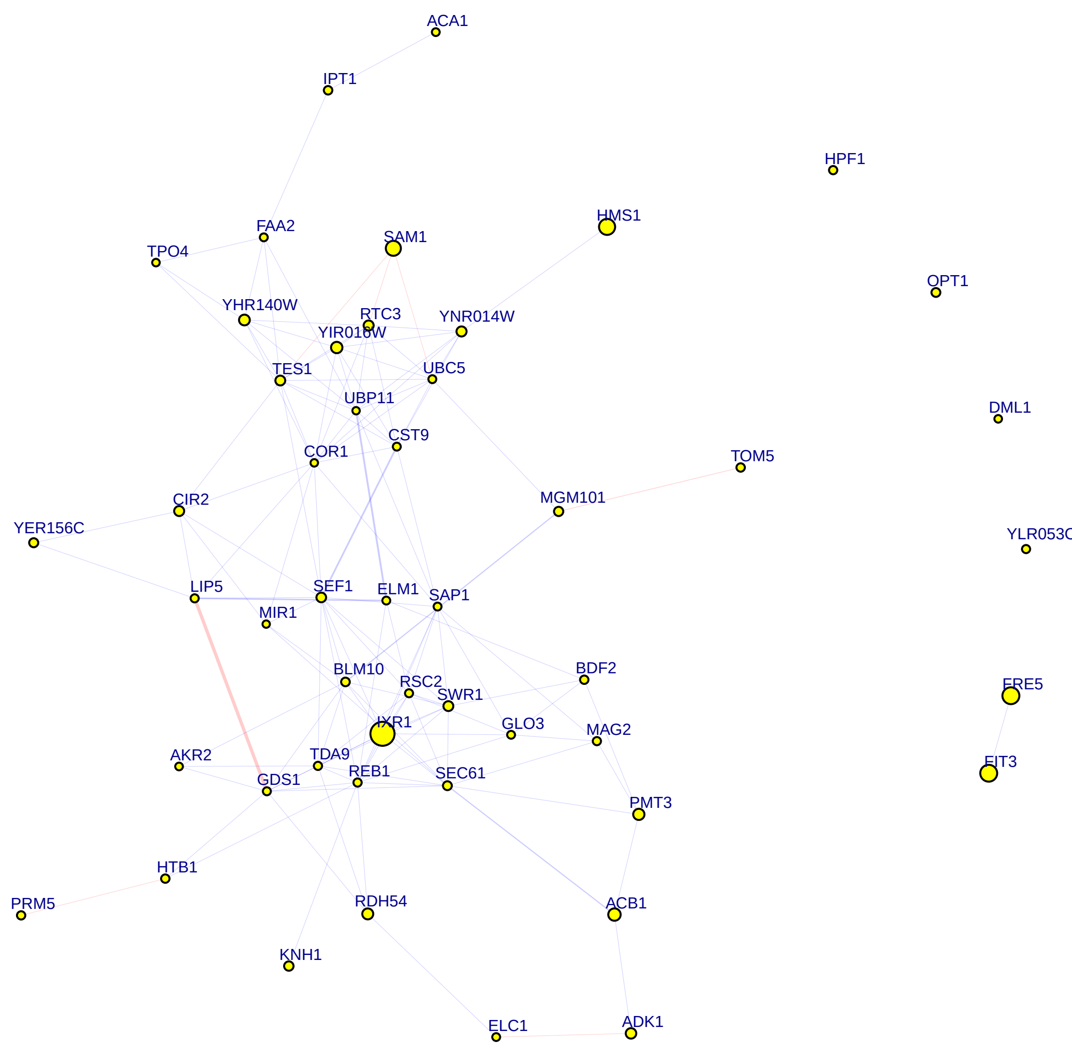

chrXIII:409778_G/T top 100 up and down genes

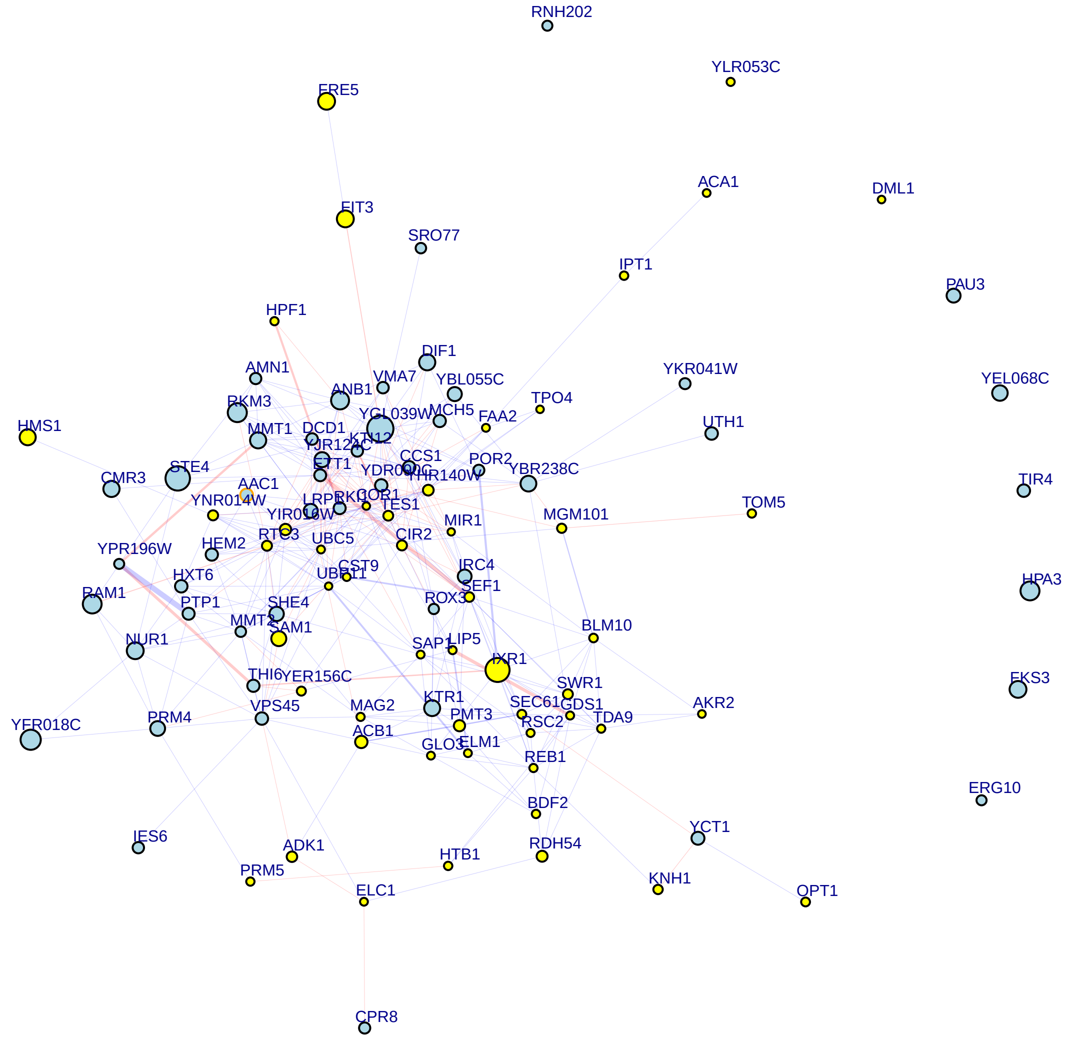

chrXIII:591723_A/G top 50 up genes

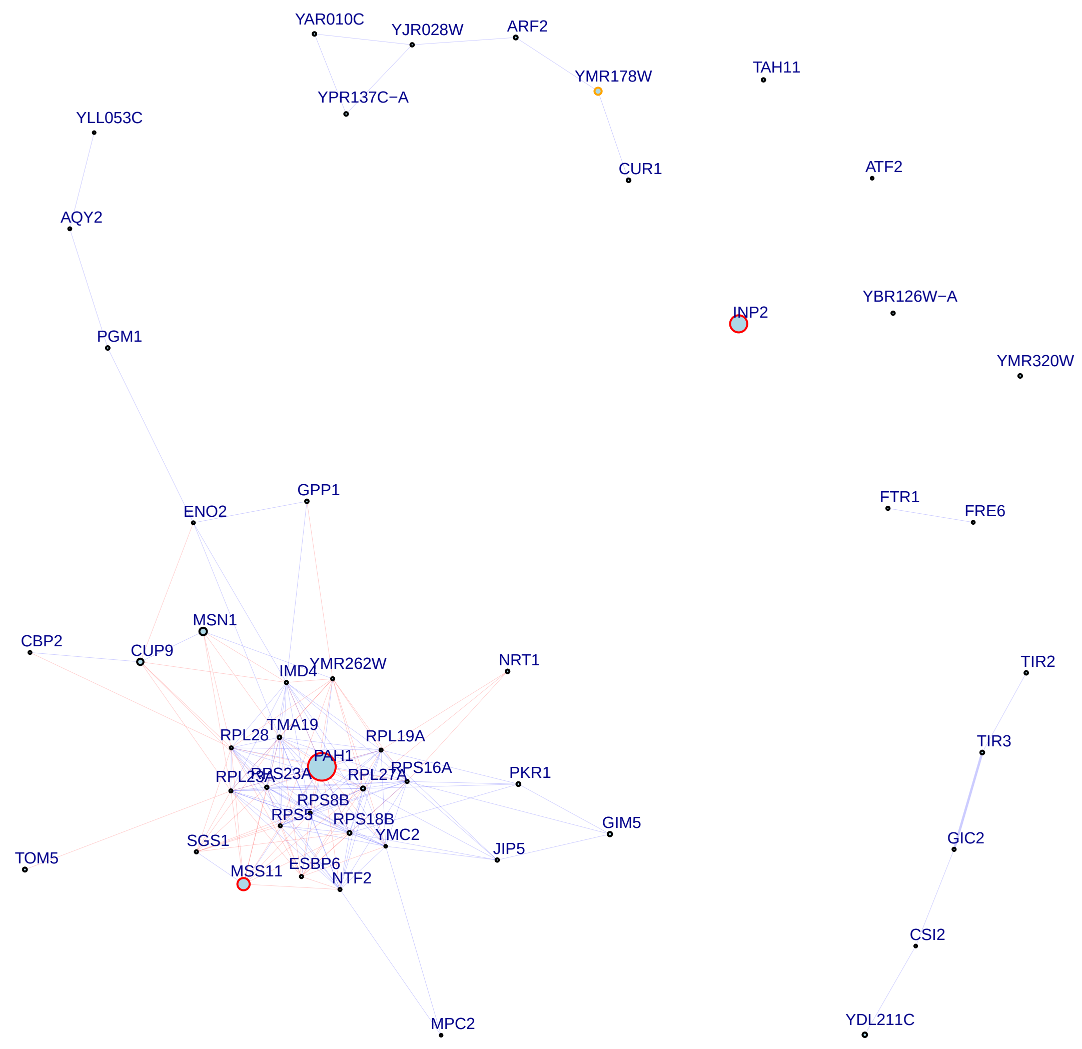

chrXIII:591723_A/G top 50 down genes

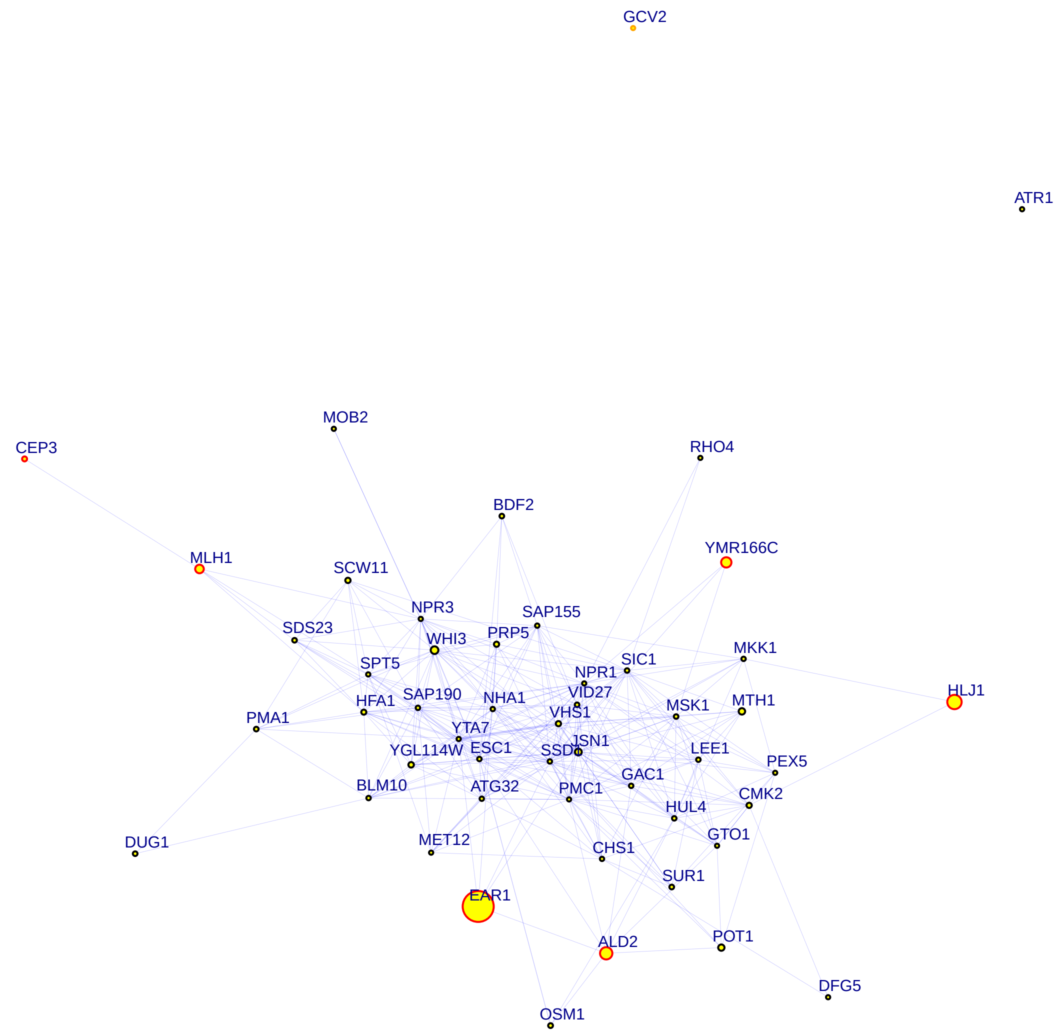

chrXIII:591723_A/G top 100 up and down genes

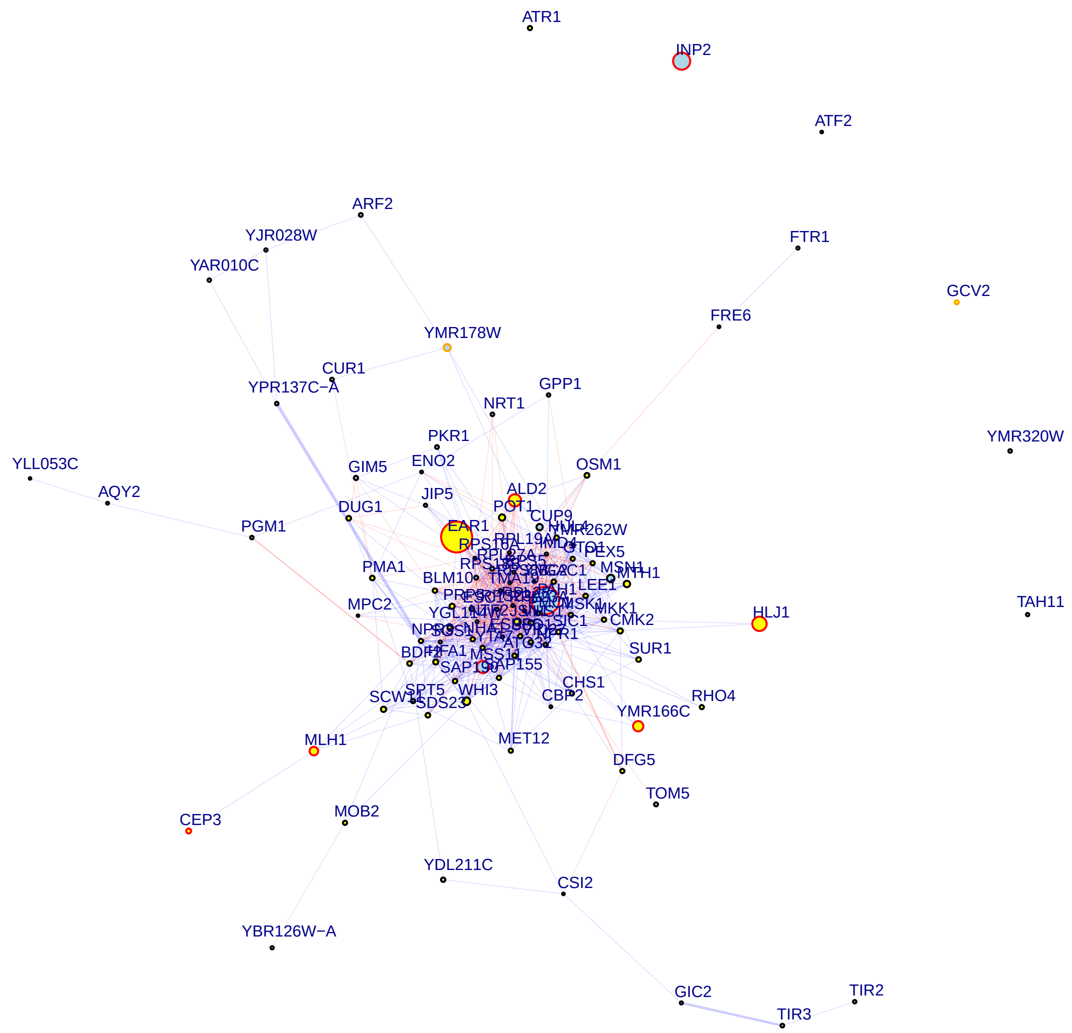

chrXIII:761318_T/A top 50 up genes

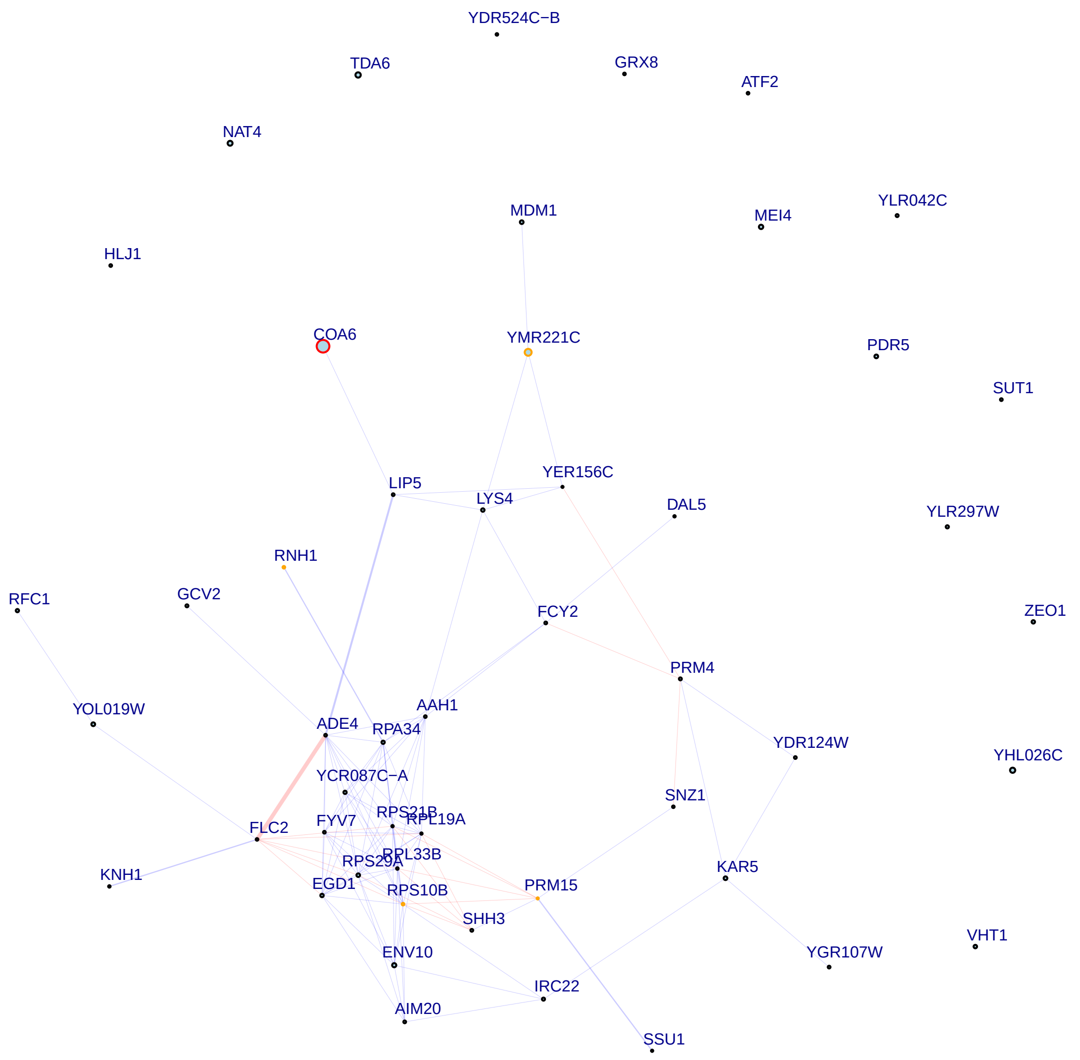

chrXIII:761318_T/A top 50 down genes

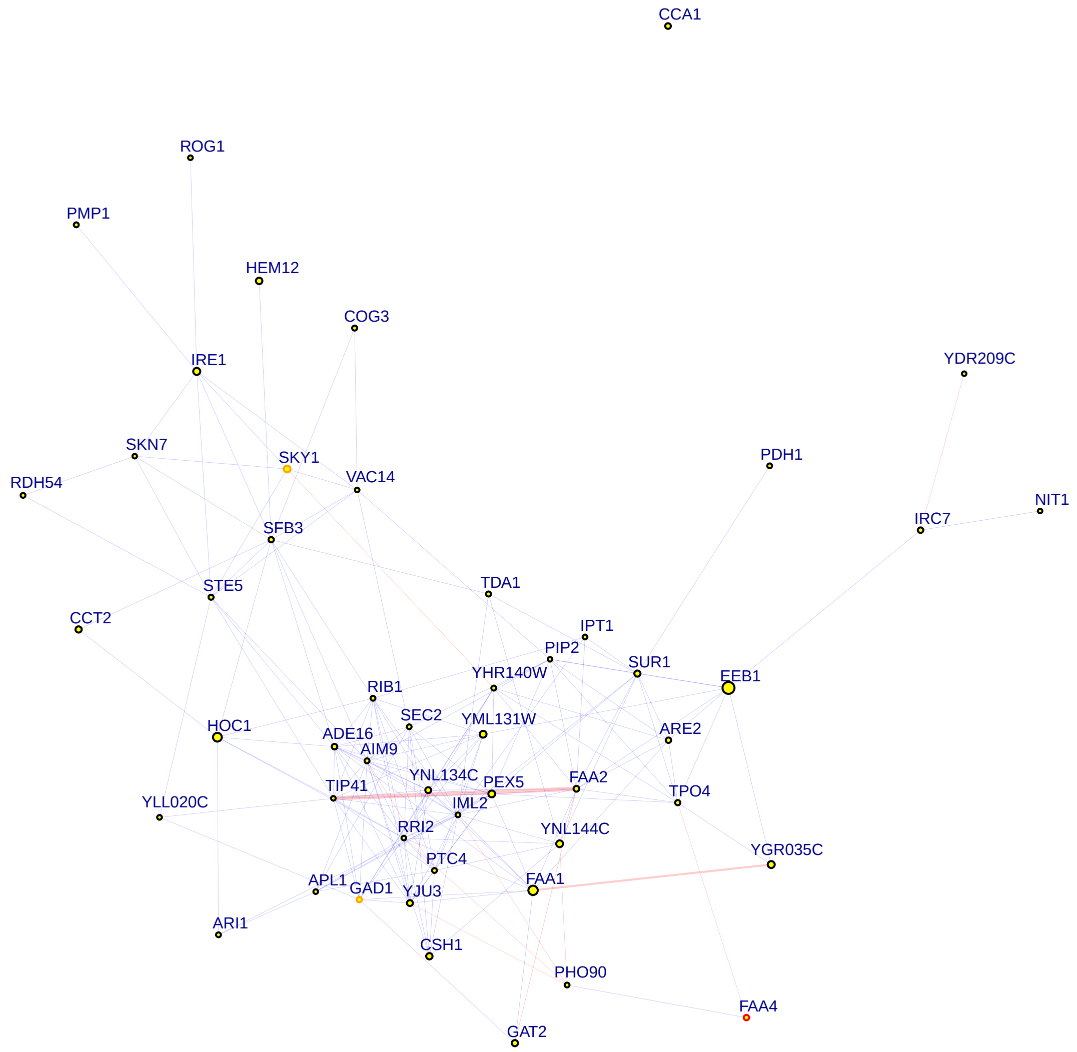

chrXIII:761318_T/A top 100 up and down genes

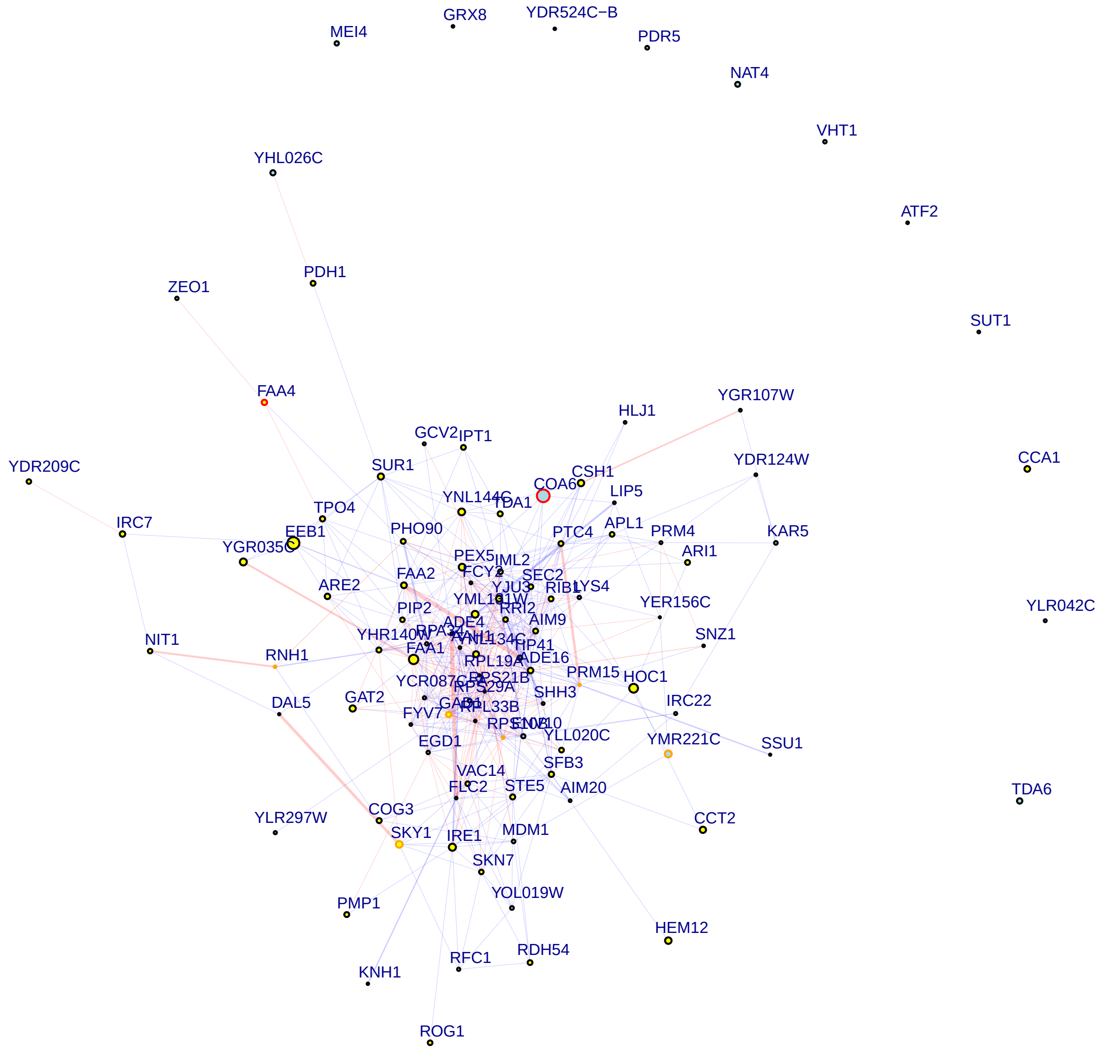

chrXIII:914575_T/C top 50 up genes

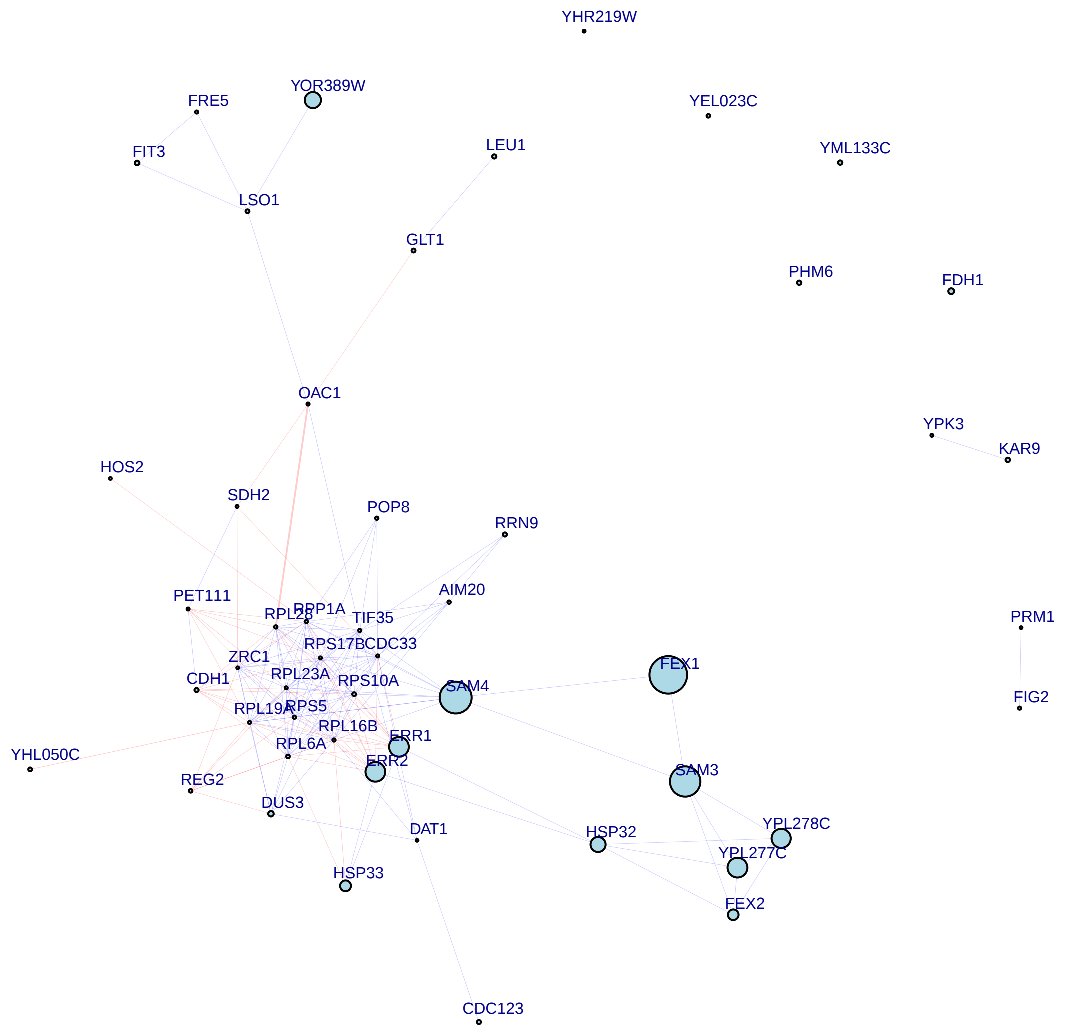

chrXIII:914575_T/C top 50 down genes

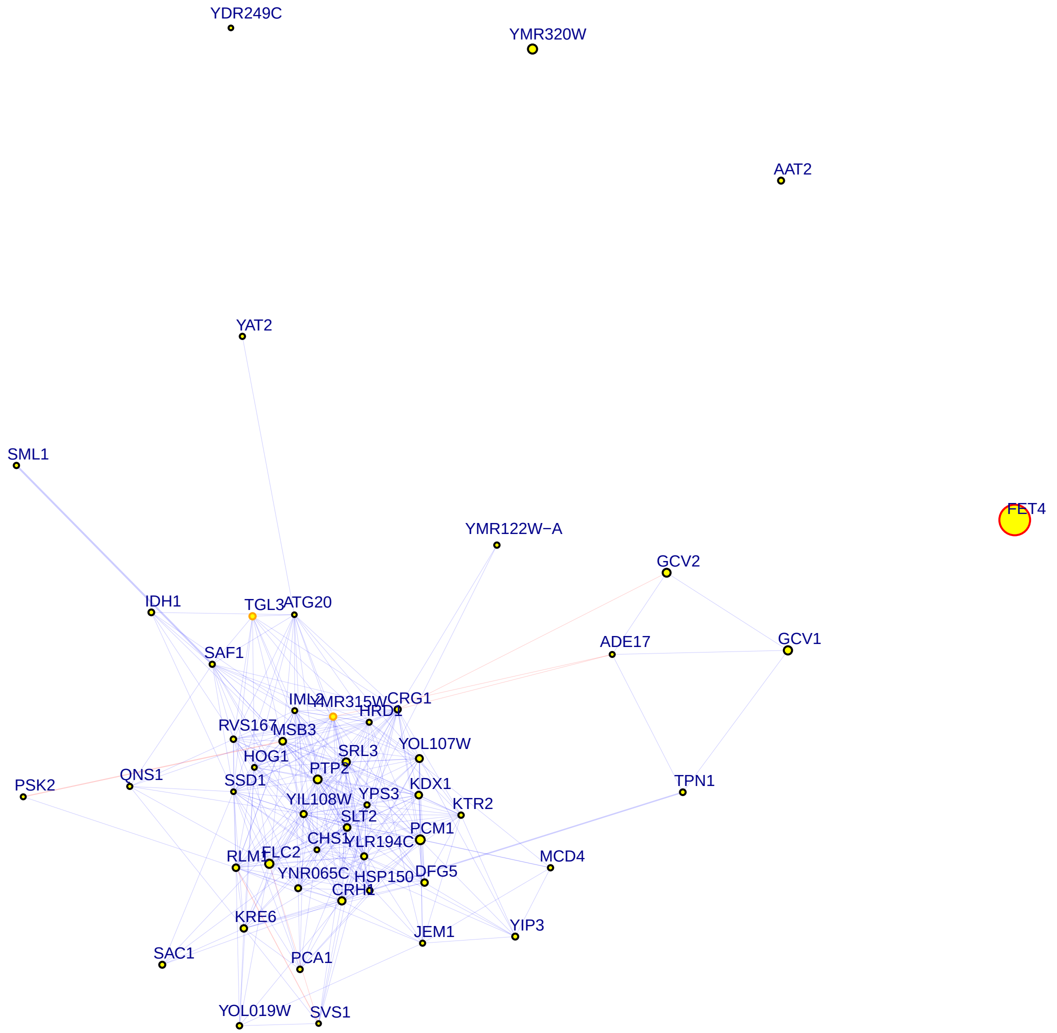

chrXIII:914575_T/C top 100 up and down genes

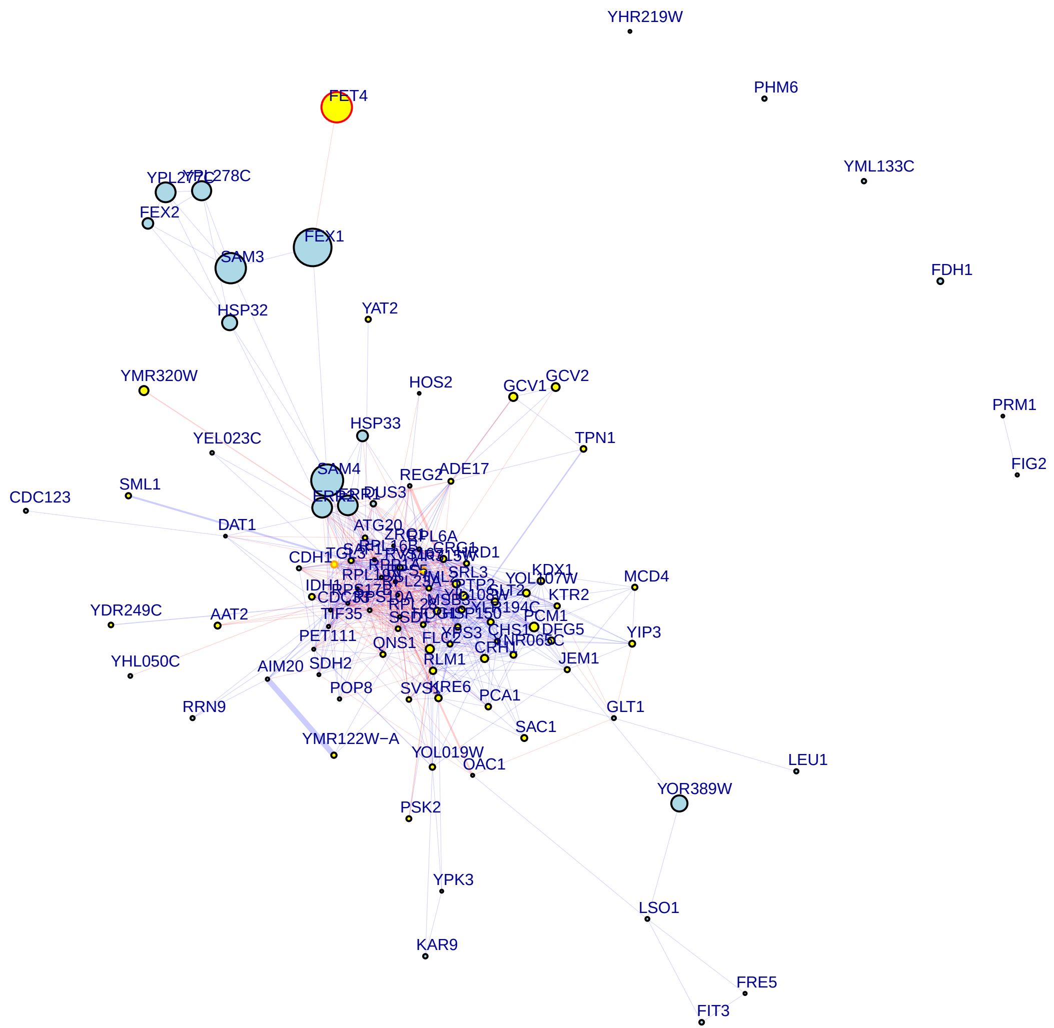

chrXIV:24003_C/CCCTTTTTTTA top 50 up genes

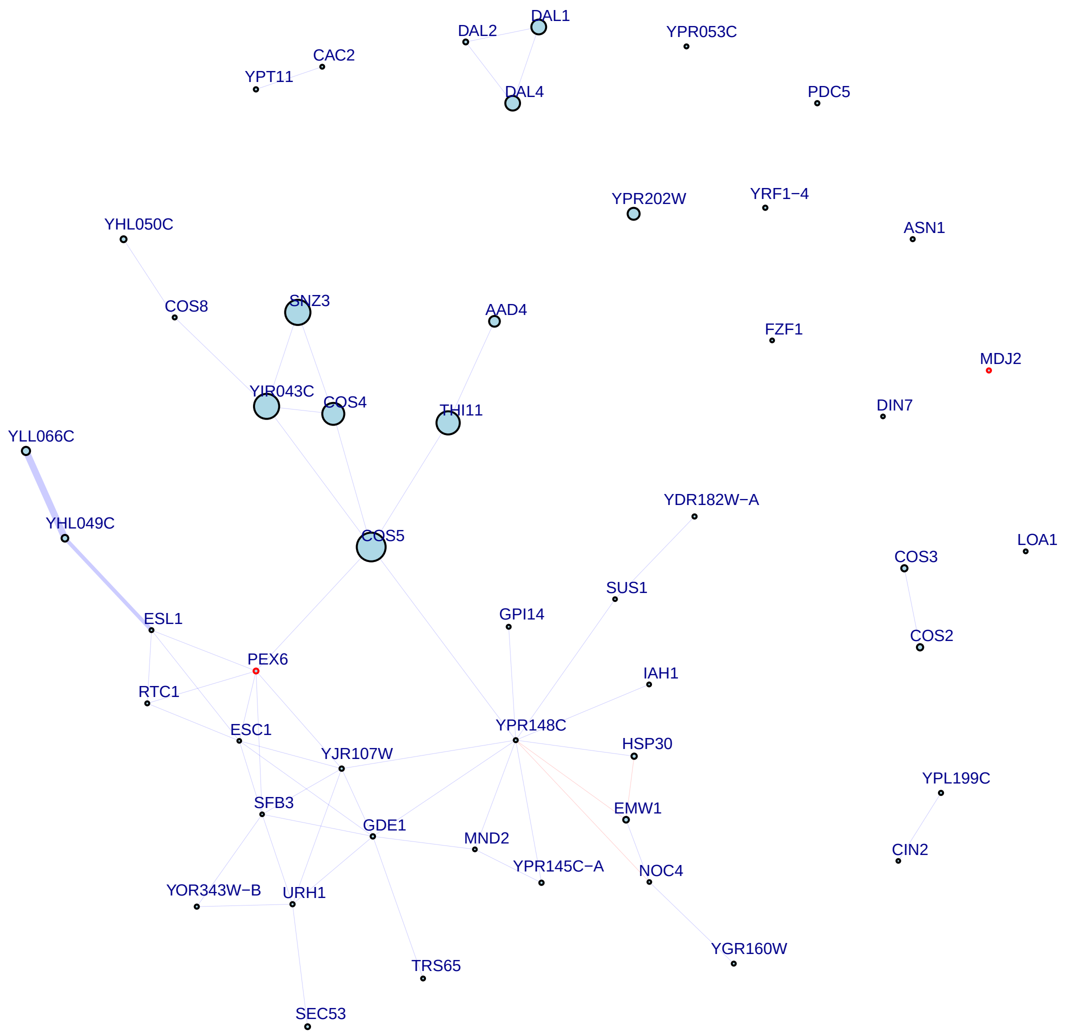

chrXIV:24003_C/CCCTTTTTTTA top 50 down genes

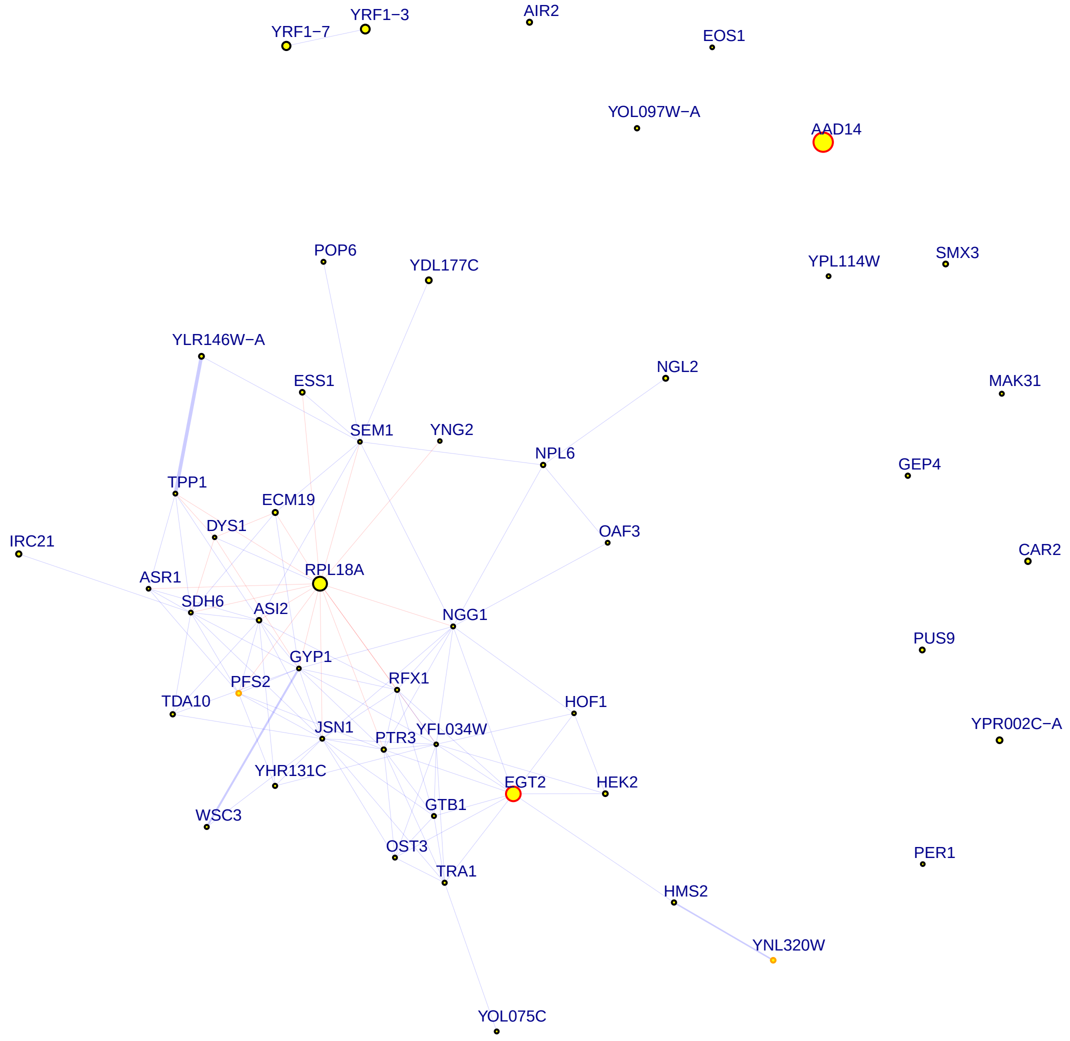

chrXIV:24003_C/CCCTTTTTTTA top 100 up and down genes

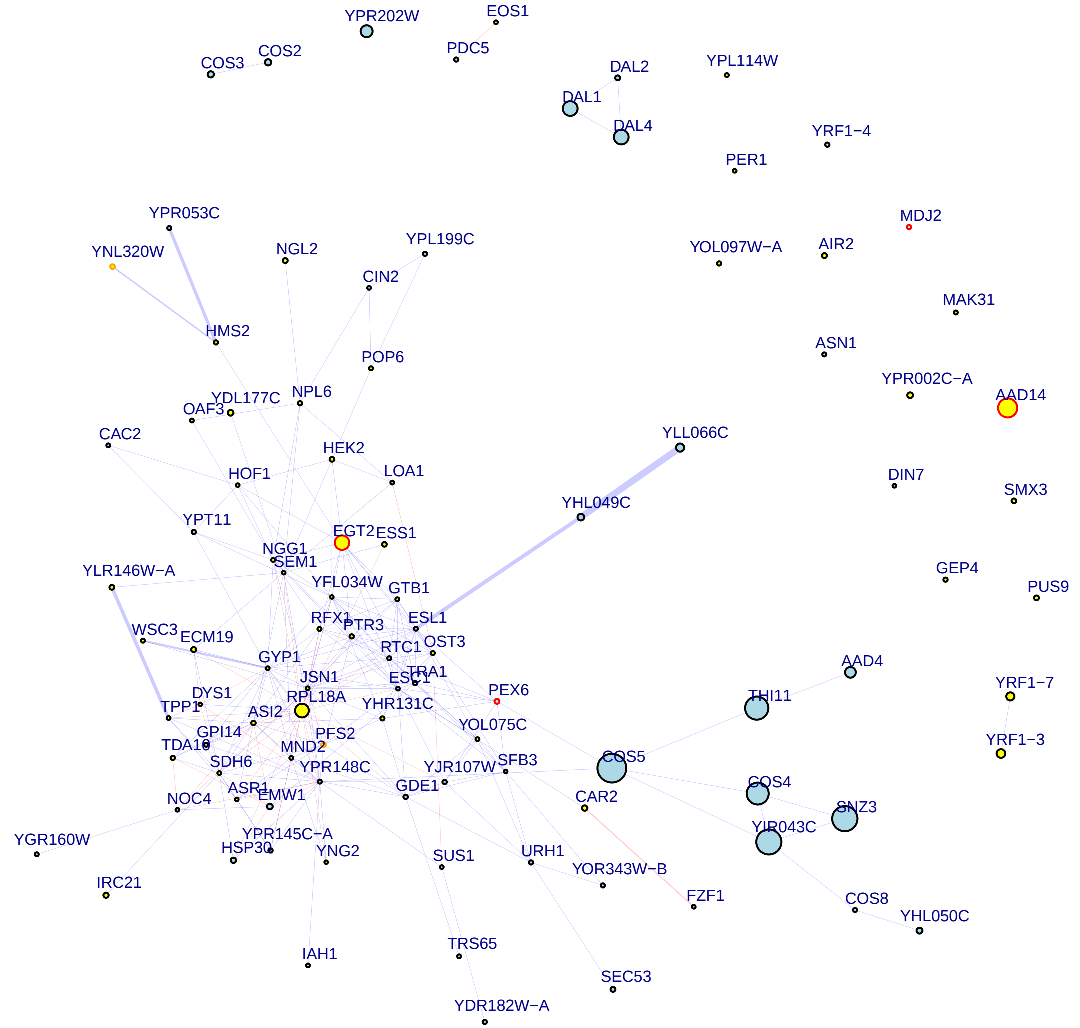

chrXIV:267161_G/A top 50 up genes

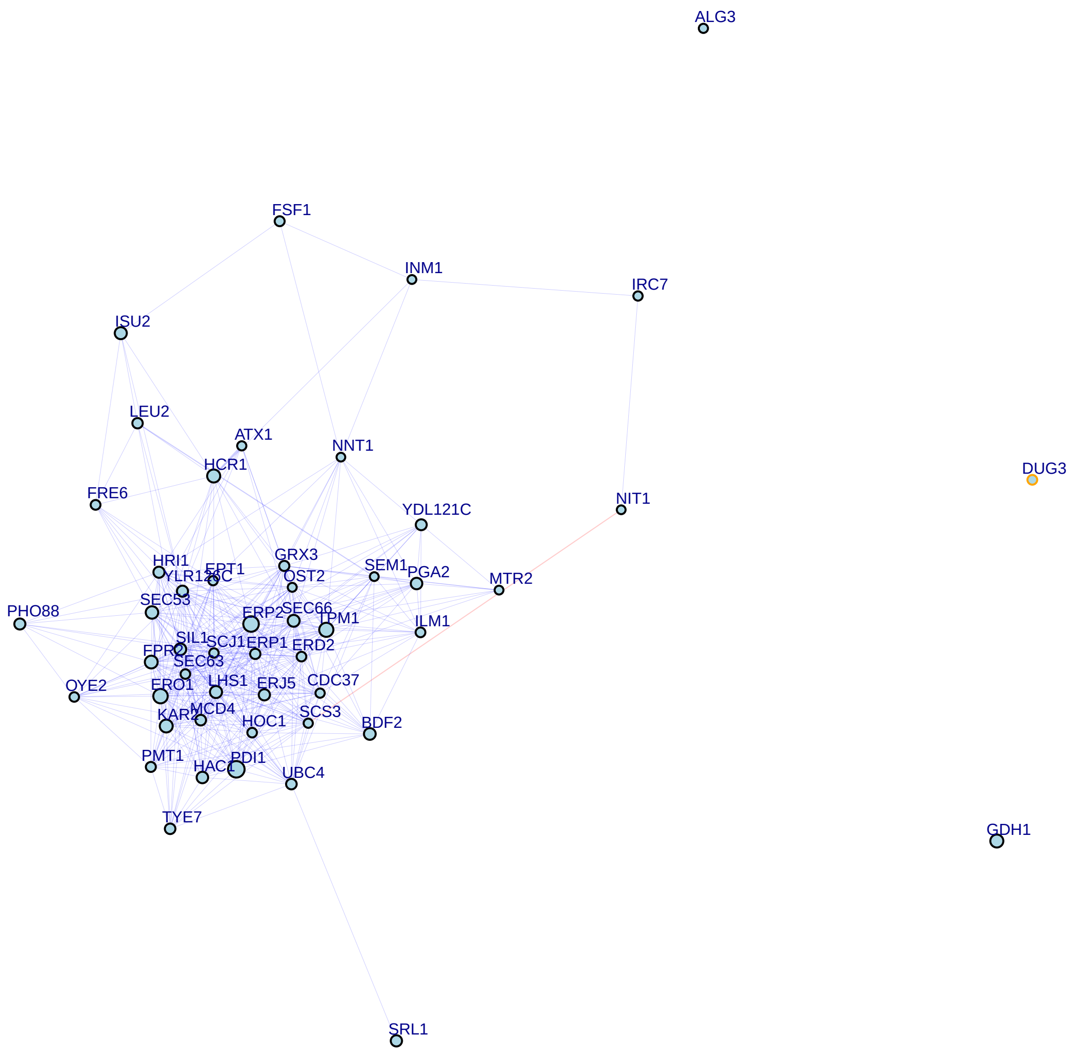

chrXIV:267161_G/A top 50 down genes

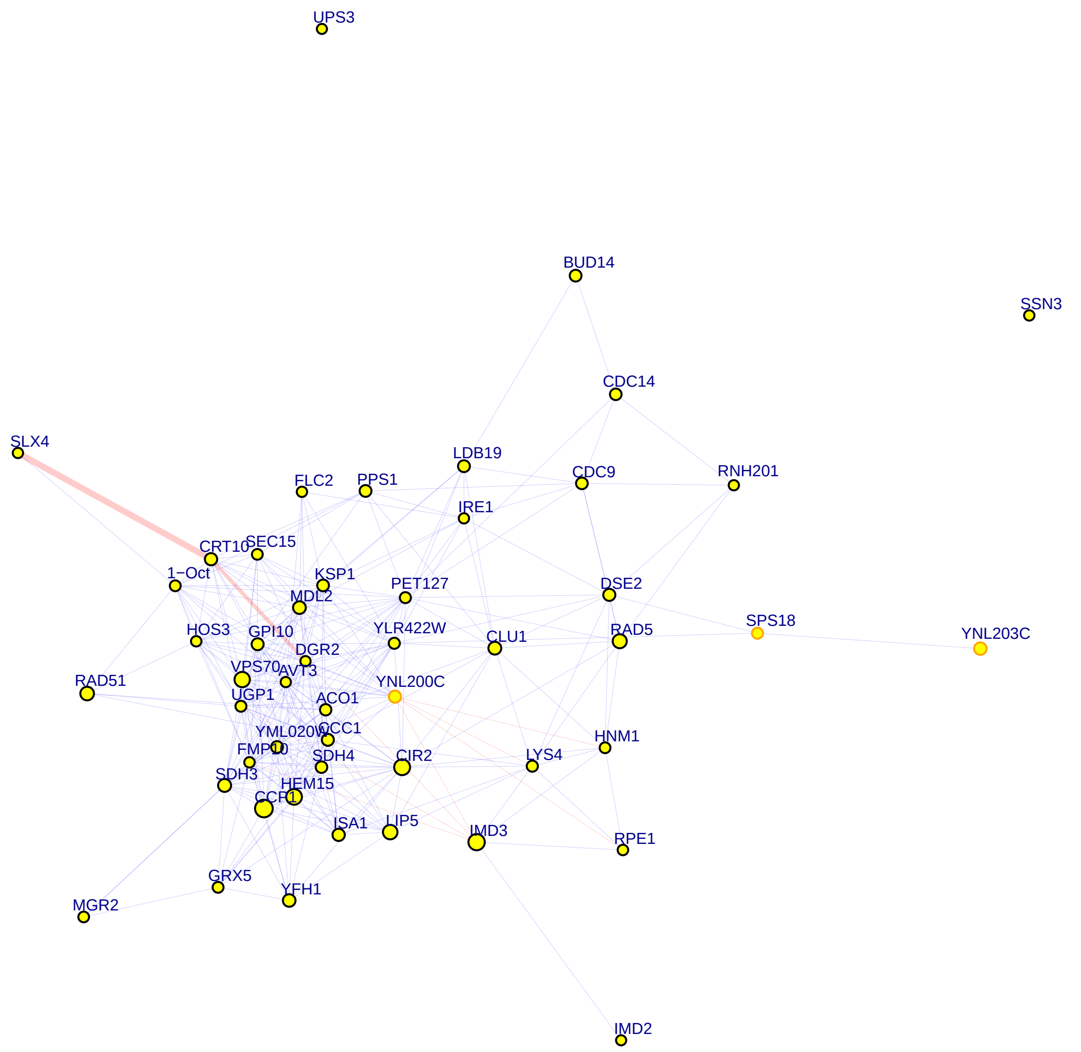

chrXIV:267161_G/A top 100 up and down genes

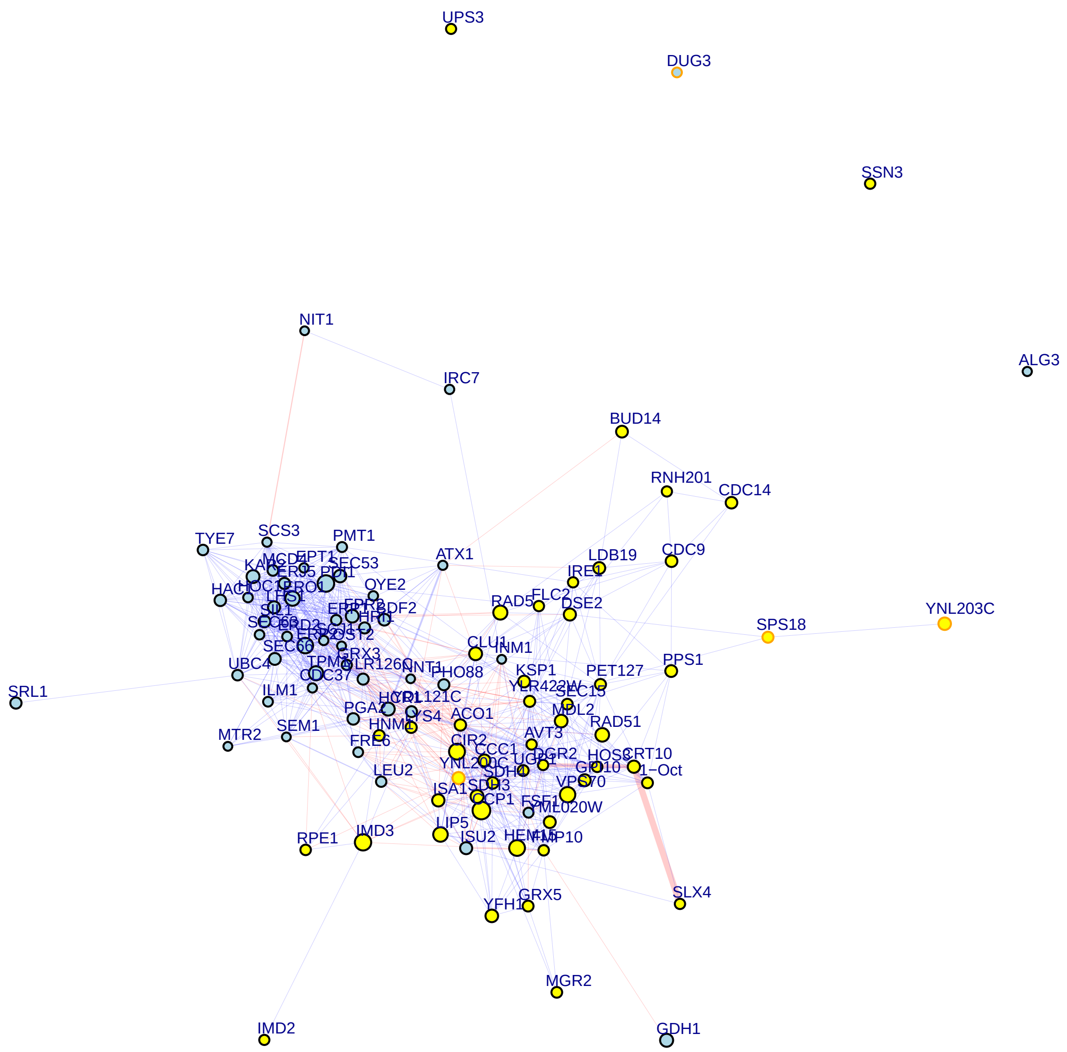

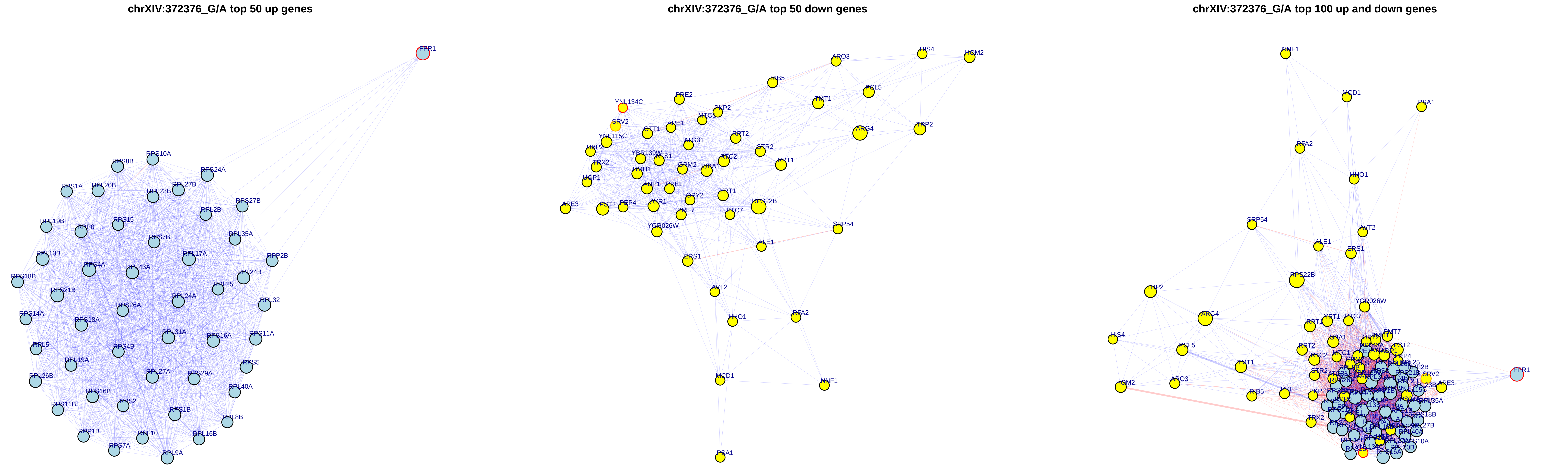
chrXIV:372376_G/A top 50 up genes
FPR1
RPS8B
RPS10A
RPS24A
RPS1A
RPL20B
RPL27B
RPL23B
RPS27B
RPL2B
RPL19B
RPP0
RPS15
RPL35A
RPS7B
RPL13B
RPL17A
RPP2B
RPS4A
RPL43A
RPS18B
RPL24B
RPL25
RPS21B
RPL32
RPS26A
RPL24A
RPS14A
RPS18A
RPS11A
RPL31A
RPS16A
RPL5
RPS4B
RPS5
RPL19A
RPL26B
RPL27A
RPS29A
RPL40A
RPS16B
RPS2
RPS11B
RPS1B
RPL8B
RPP1B
RPL10
RPL16B
RPS7A
RPL9A
chrXIV:372376_G/A top 50 down genes
HIS4
HOM2
ARO3
RIB5
PCL5
PRE2
TMT1
YNL134C
PKP2
SRV2
MTC1
ARG4
APE1
GTT1
TRP2
YNL115C
ATG31
RRT2
HRP2
STR2
YBR139W
RTC2
RRT1
TRX2
GRM2
SBA1
BMH1
UGP1
ADP1
PRE1
YRT1
ARE3
PST2
PEP4
AYR1
GPY2
RPS22B
PMT7
PTC7
YGR026W
SRP54
ALE1
ERS1
AVT2
HHO1
RFA2
MCD1
HNF1
PSA1
chrXIV:372376_G/A top 100 up and down genes
HNF1
MCD1
PSA1
RFA2
HHO1
SRP54
AVT2
ALE1
ERS1
TRP2
RPS22B
YGR026W
ARG4
HIS4
PCL5
TMT1
HOM2
ARO3
RIB5
PRE2
TRX2
FPR1
SRV2
ARE3

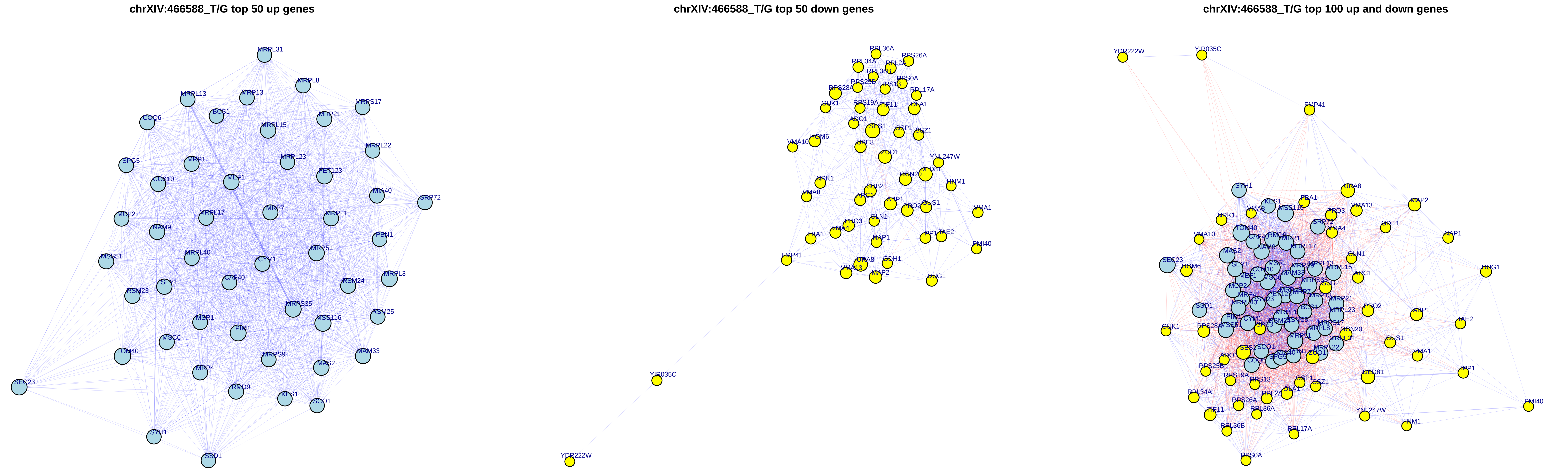
chrXIV:466588_T/G top 50 up genes
chrXIV:466588_T/G top 50 down genes
chrXIV:466588_T/G top 100 up and down genes
MRRL31
MRPL8
MRRL13
MRP13
MRRS17
COQ6
BCS1
MRP21
MRRL15
MRRL22
SPG5
MRP1
MRRL23
PET123
COX10
MEF1
MIA40
SRP72
MCP2
MRP7
MRPL1
MRRL17
NAM9
PBN1
MSS51
MRRL40
MRP51
CYM1
MRRL3
CAF40
SEY1
RSM24
RSM23
MRPS35
RSM25
MSR1
MSS116
PIM1
MSC6
MAM33
TOM40
MRPS9
MAS2
MRP4
RMD9
SEC23
KES1
SCO1
SYH1
SSD1
RPL36A
RPS26A
RPL34A
RPS0A
RPS28A
RPL17A
GUK1
RPS19A
TIF11
OLA1
ADO1
SES1
GSP1
BSZ1
VMA10
HOM6
SRE3
ZDO1
YNL247W
PED81
GSN20
HNM1
NRK1
SNB2
VMA8
ARC1
ABP1
VMA1
PRO2
GUS1
PRO3
GLN1
FBA1
VMA4
IRP1
TAE2
MAP1
PMI40
FMP41
URA8
GDH1
VMA13
MAP2
DUG1
YIR035C
YDR222W

chrXIV:579945_GTTA/G top 50 up genes

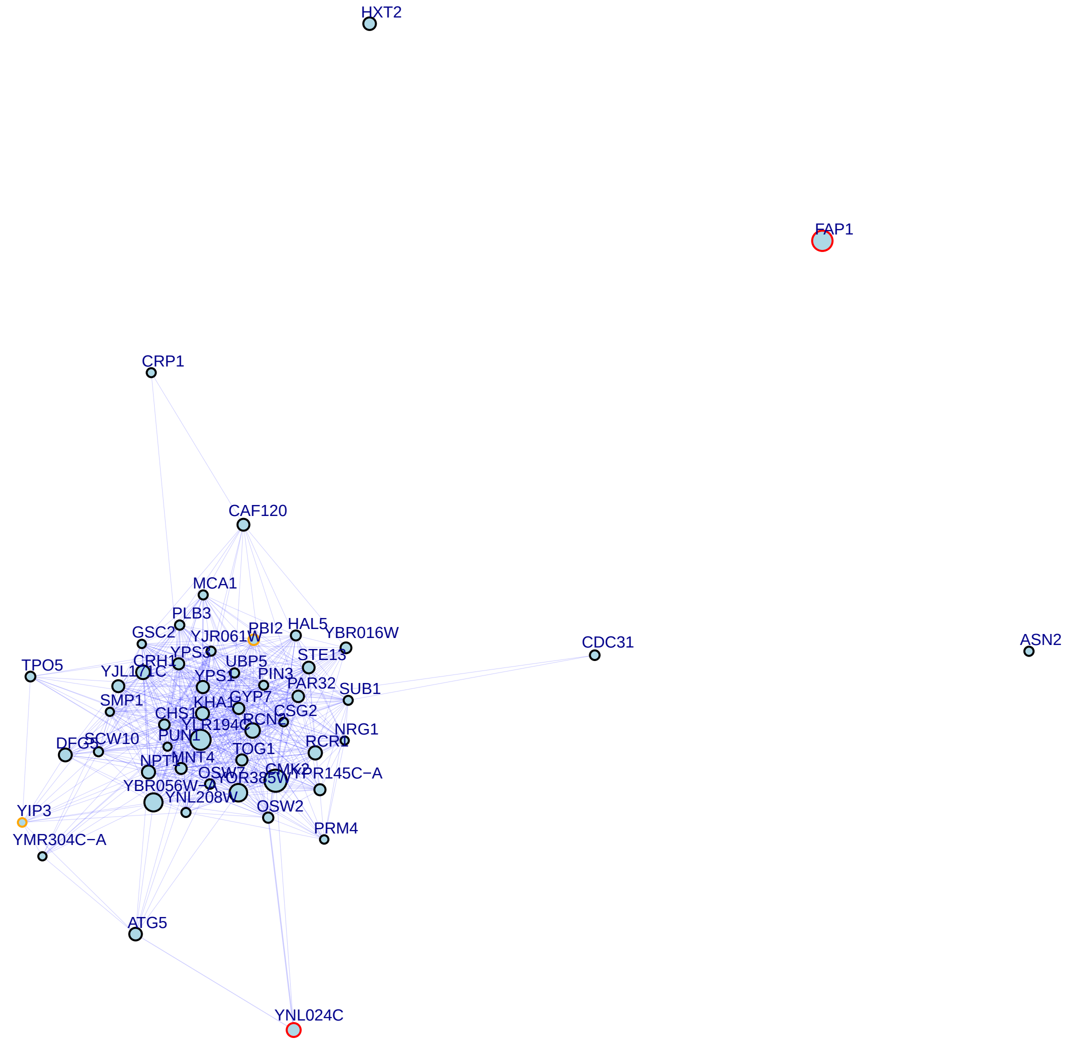

chrXIV:579945_GTTA/G top 50 down genes

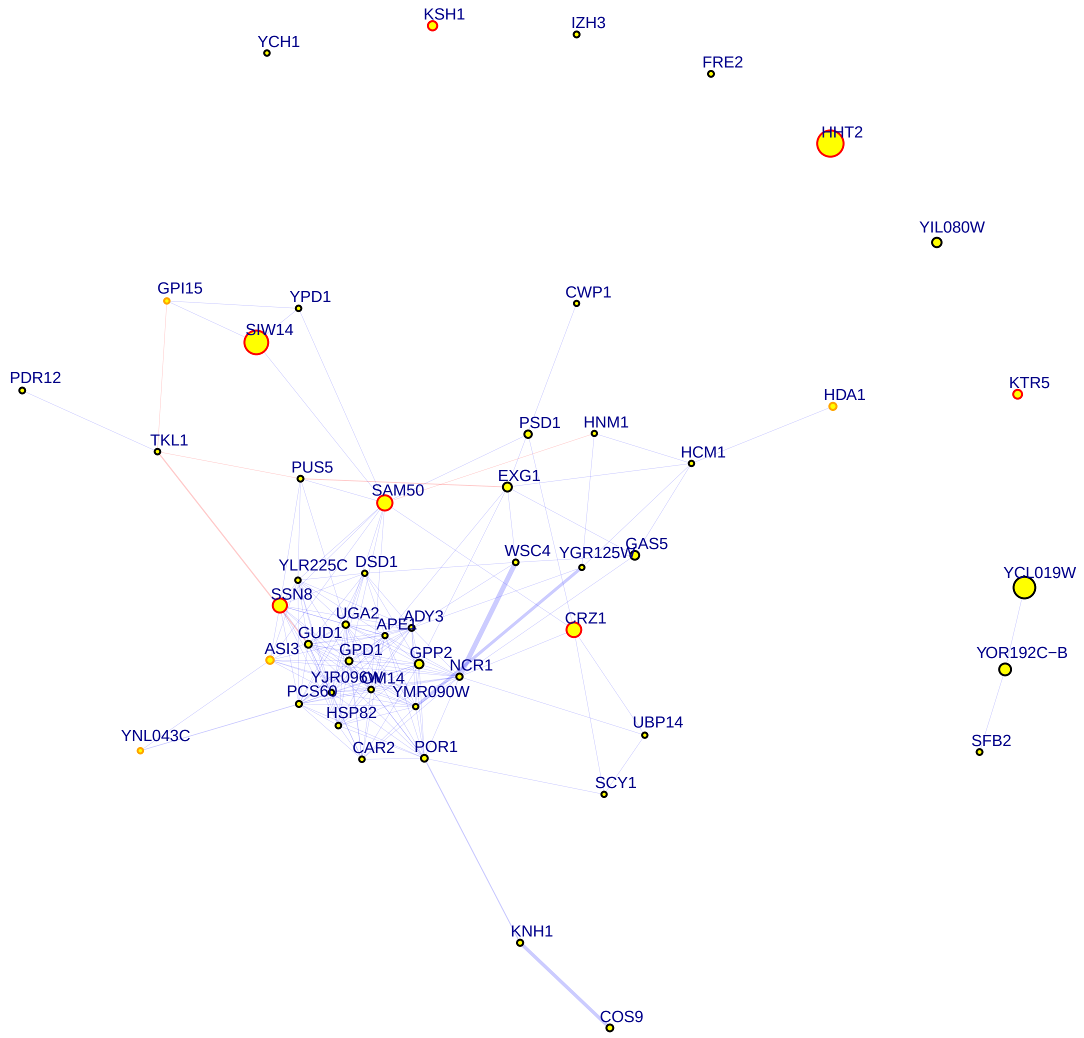

chrXIV:579945_GTTA/G top 100 up and down genes

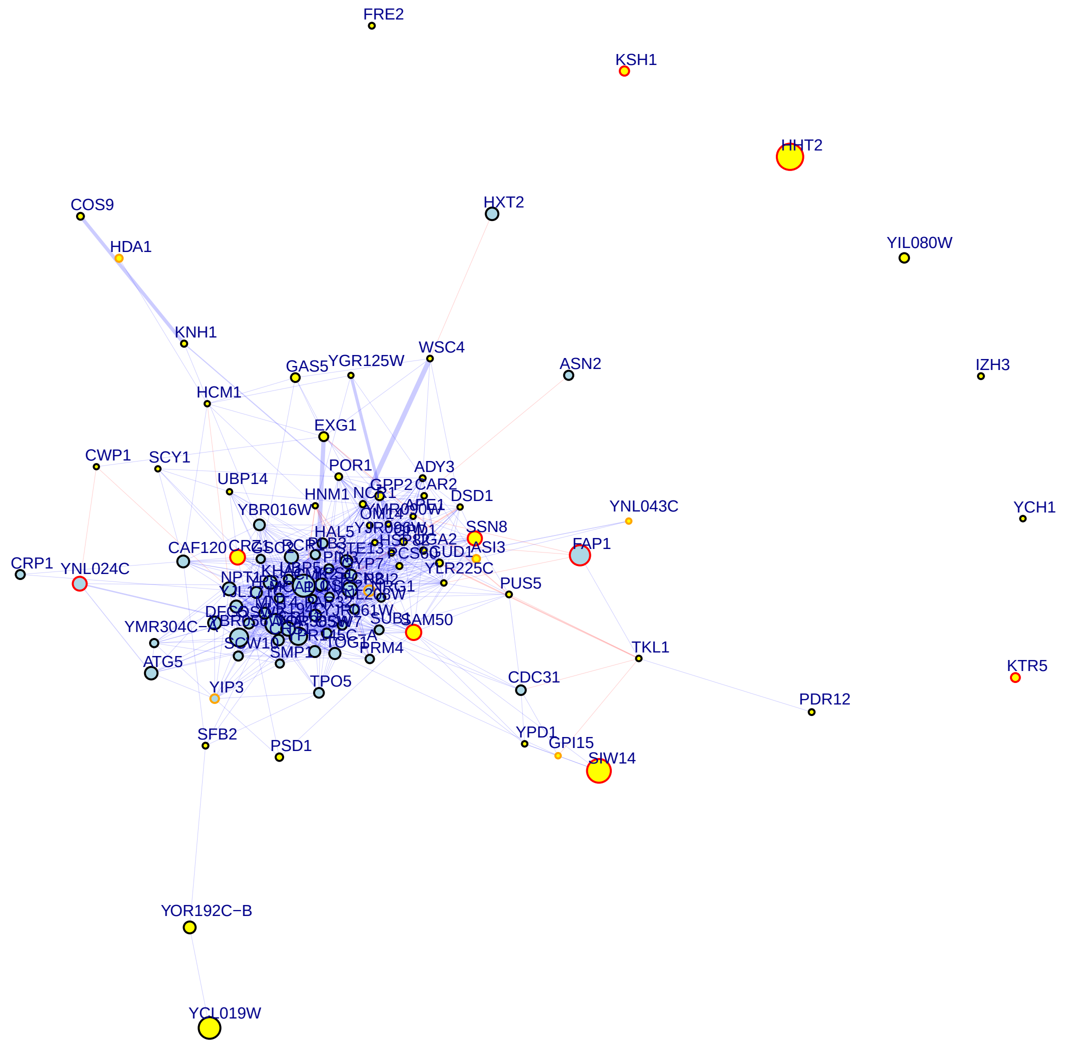

chrXV:56600_G/C top 50 up genes

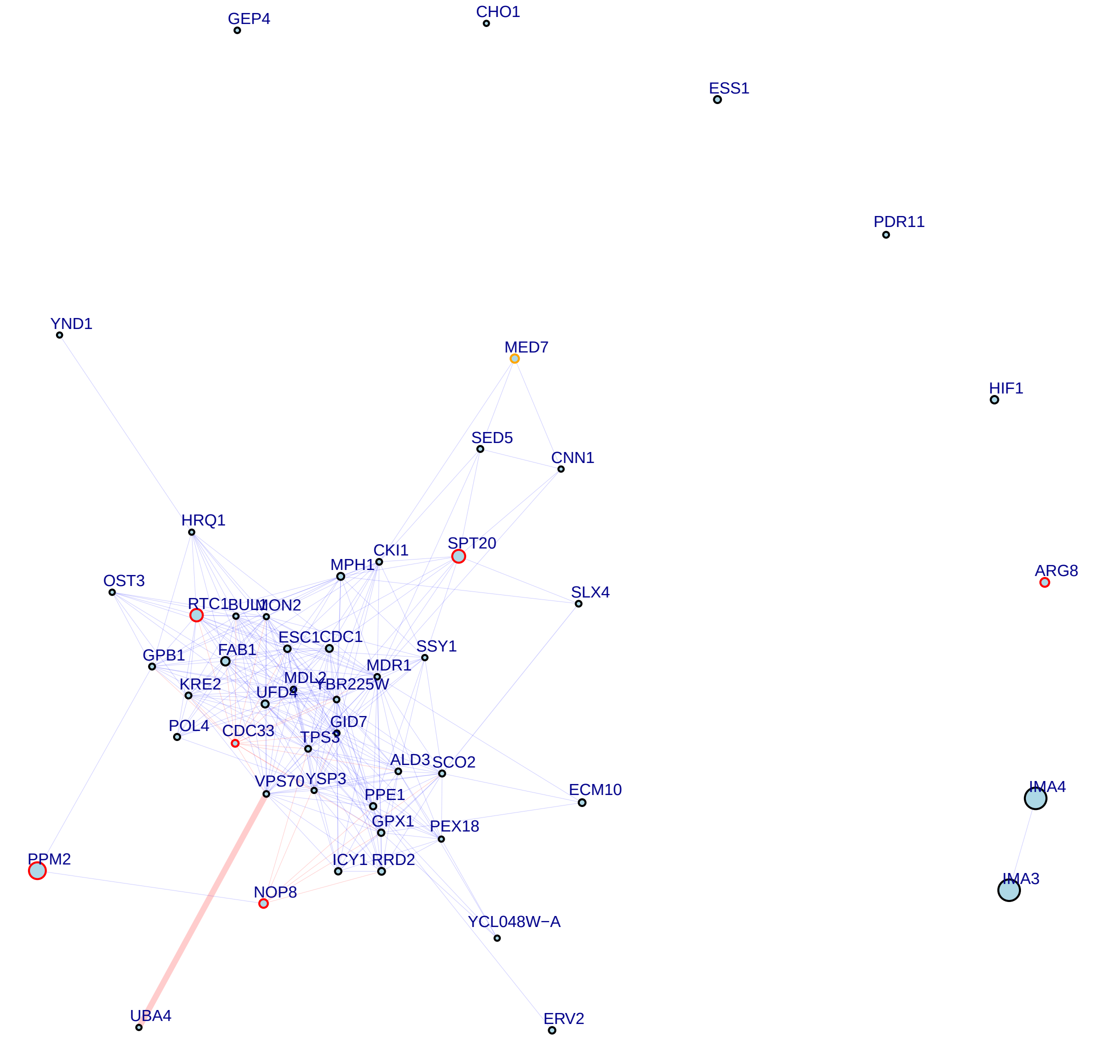

chrXV:56600_G/C top 50 down genes

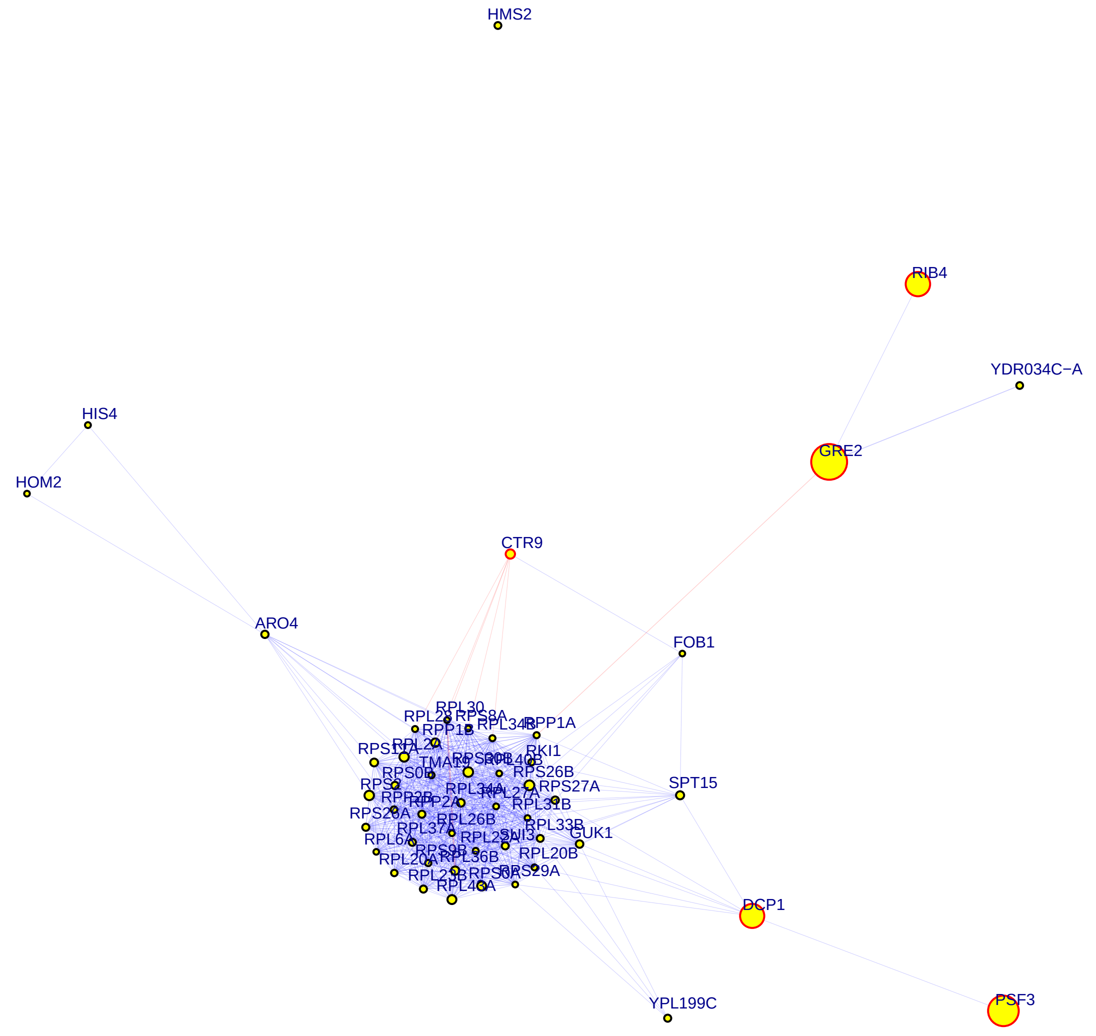

chrXV:56600_G/C top 100 up and down genes

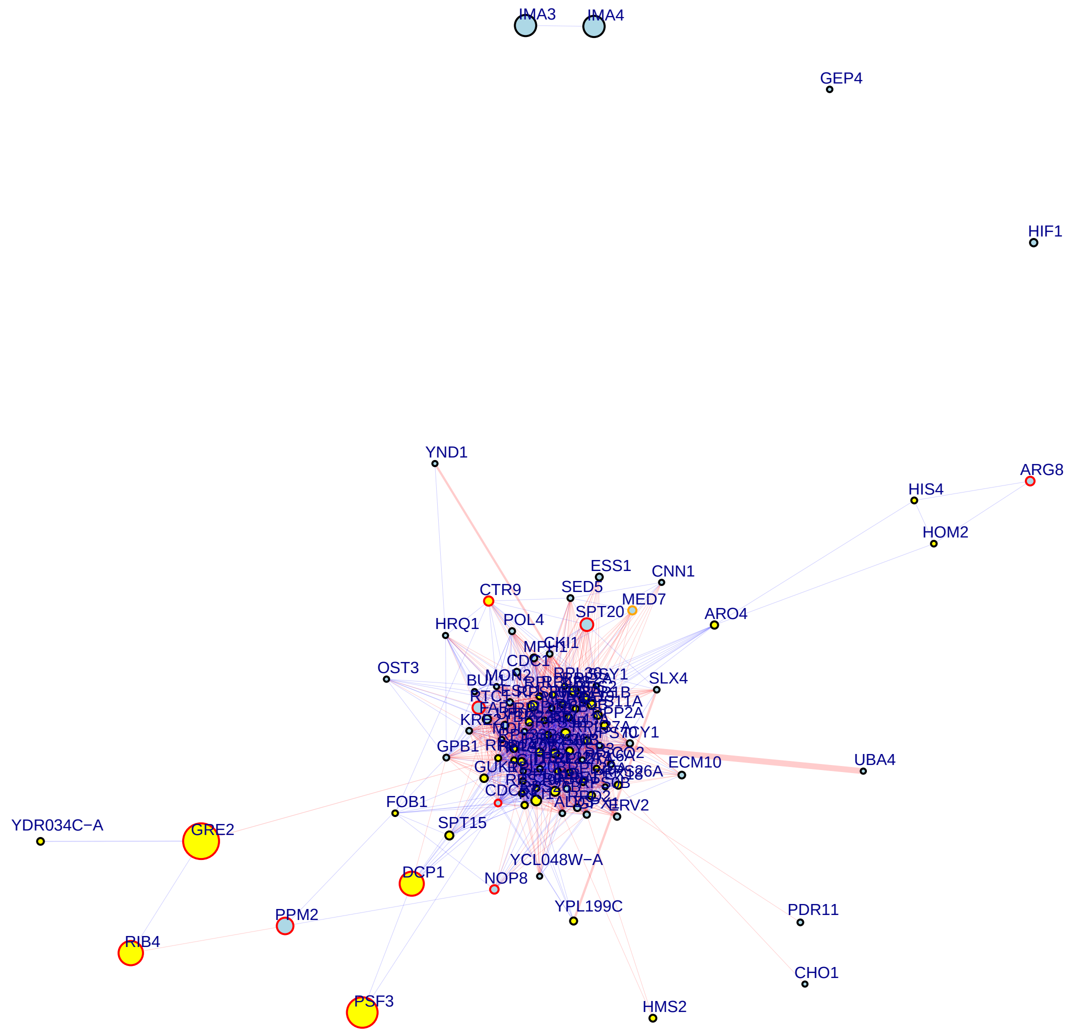

chrXV:150803_G/A top 50 up genes

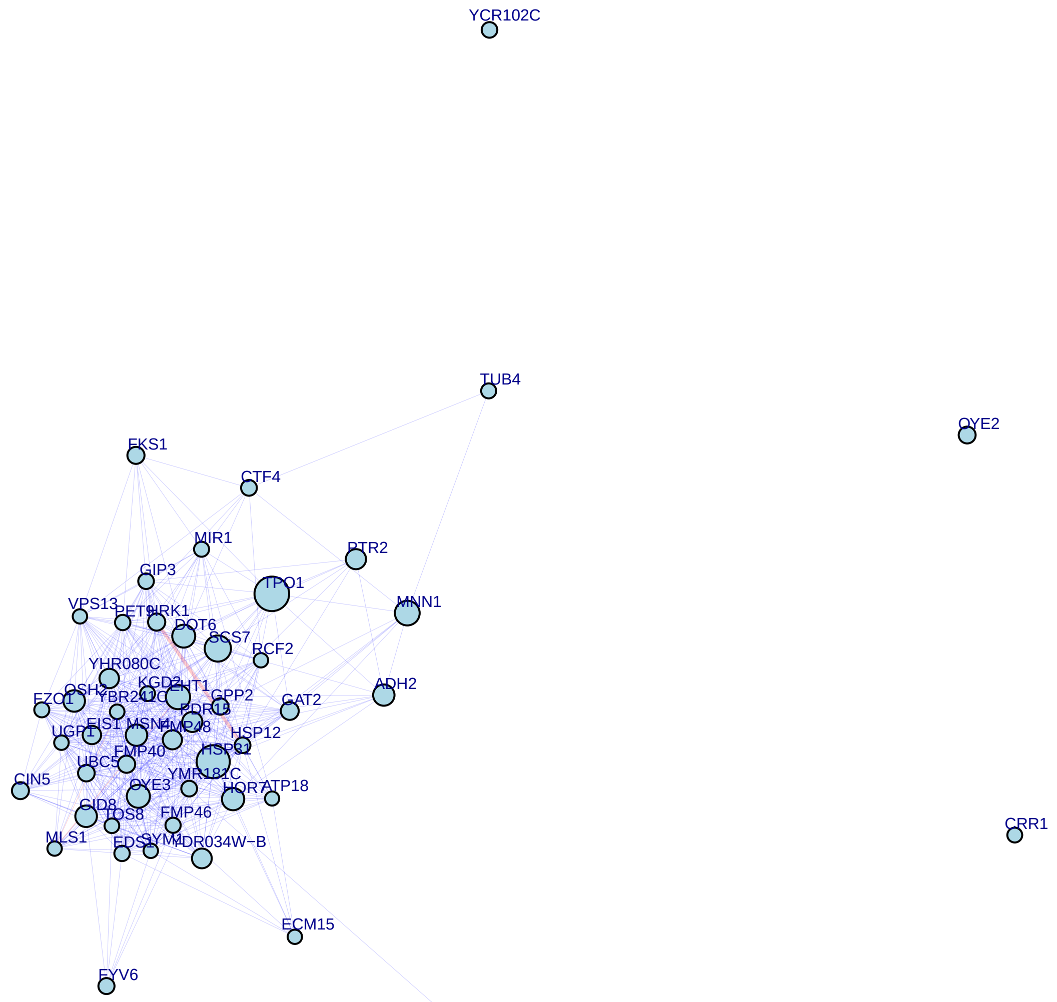

chrXV:150803_G/A top 50 down genes

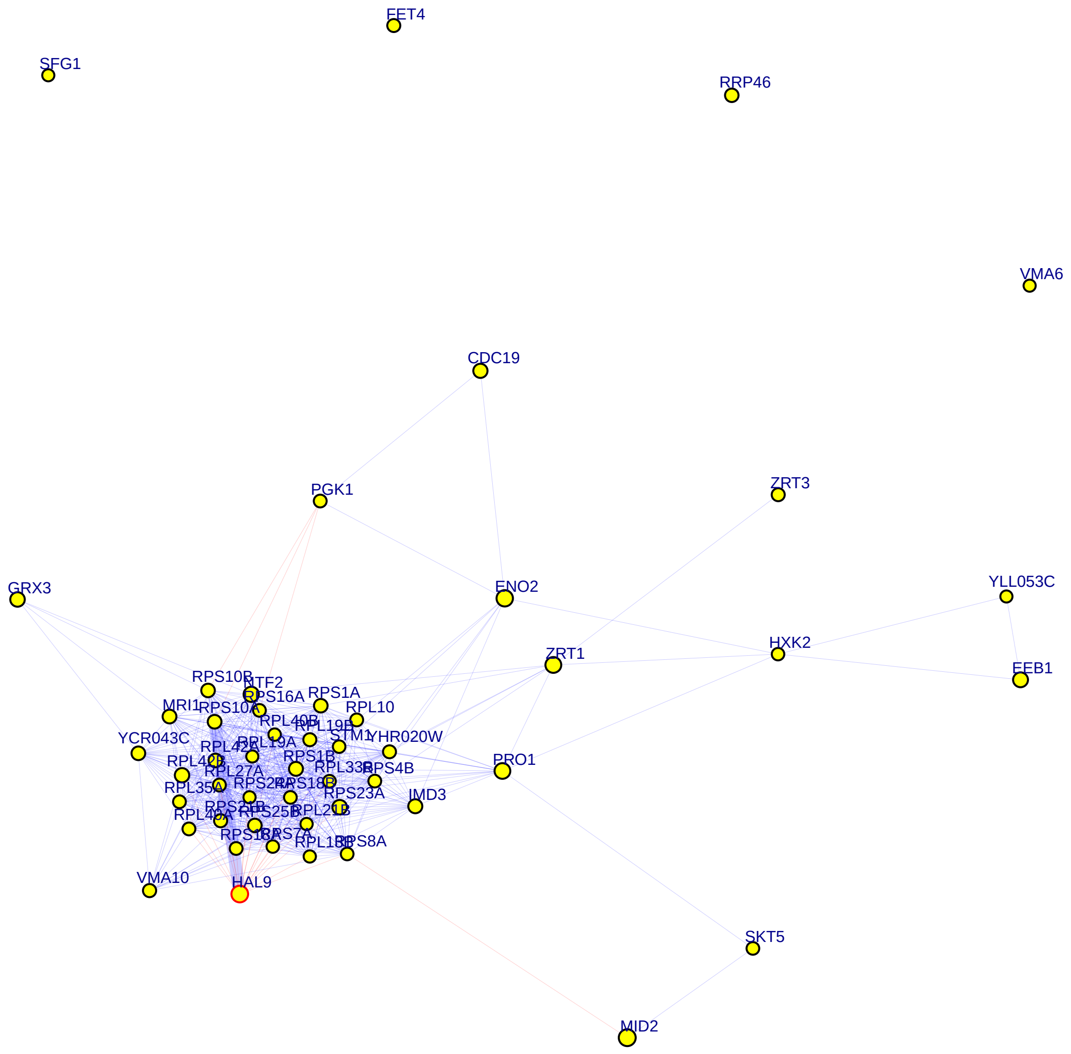

chrXV:150803_G/A top 100 up and down genes

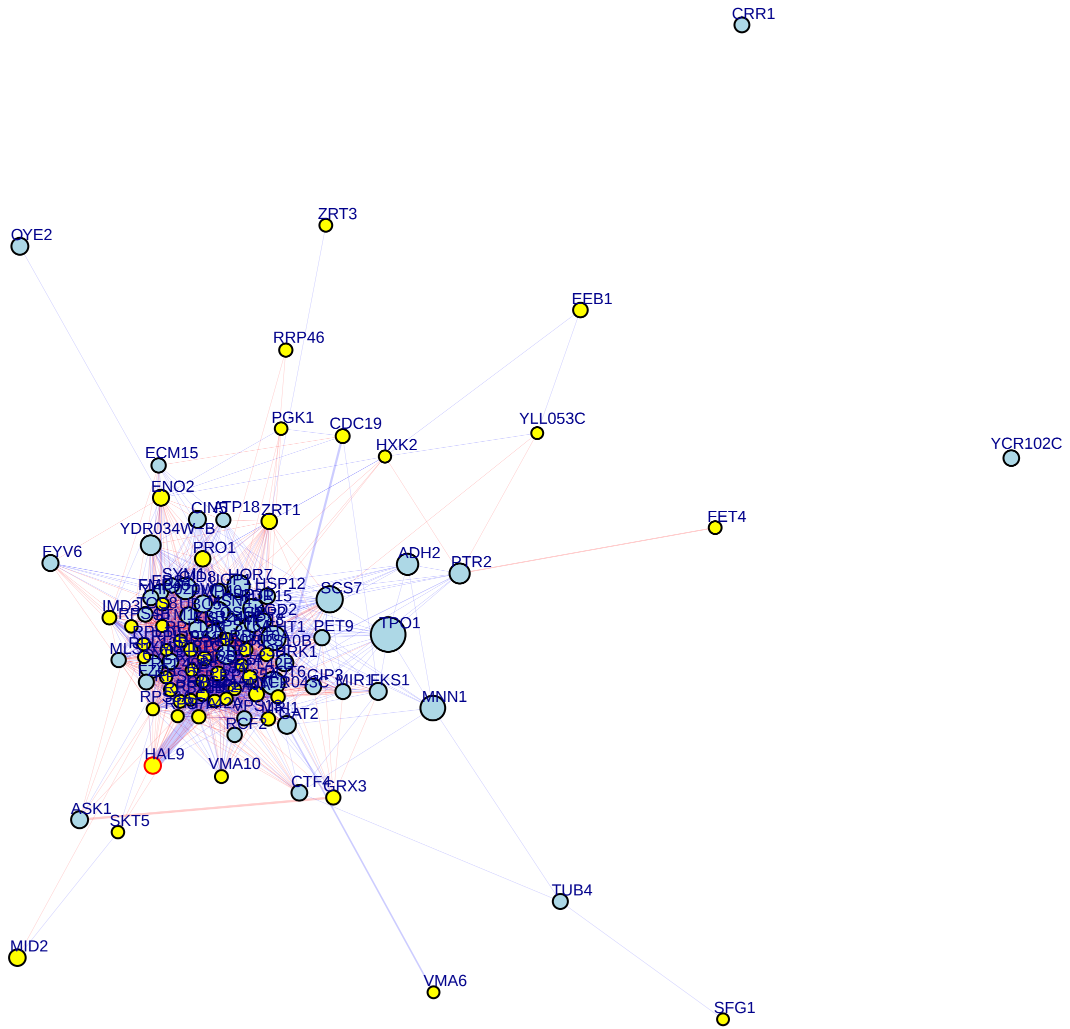

chrXV:171150_T/C top 50 up genes

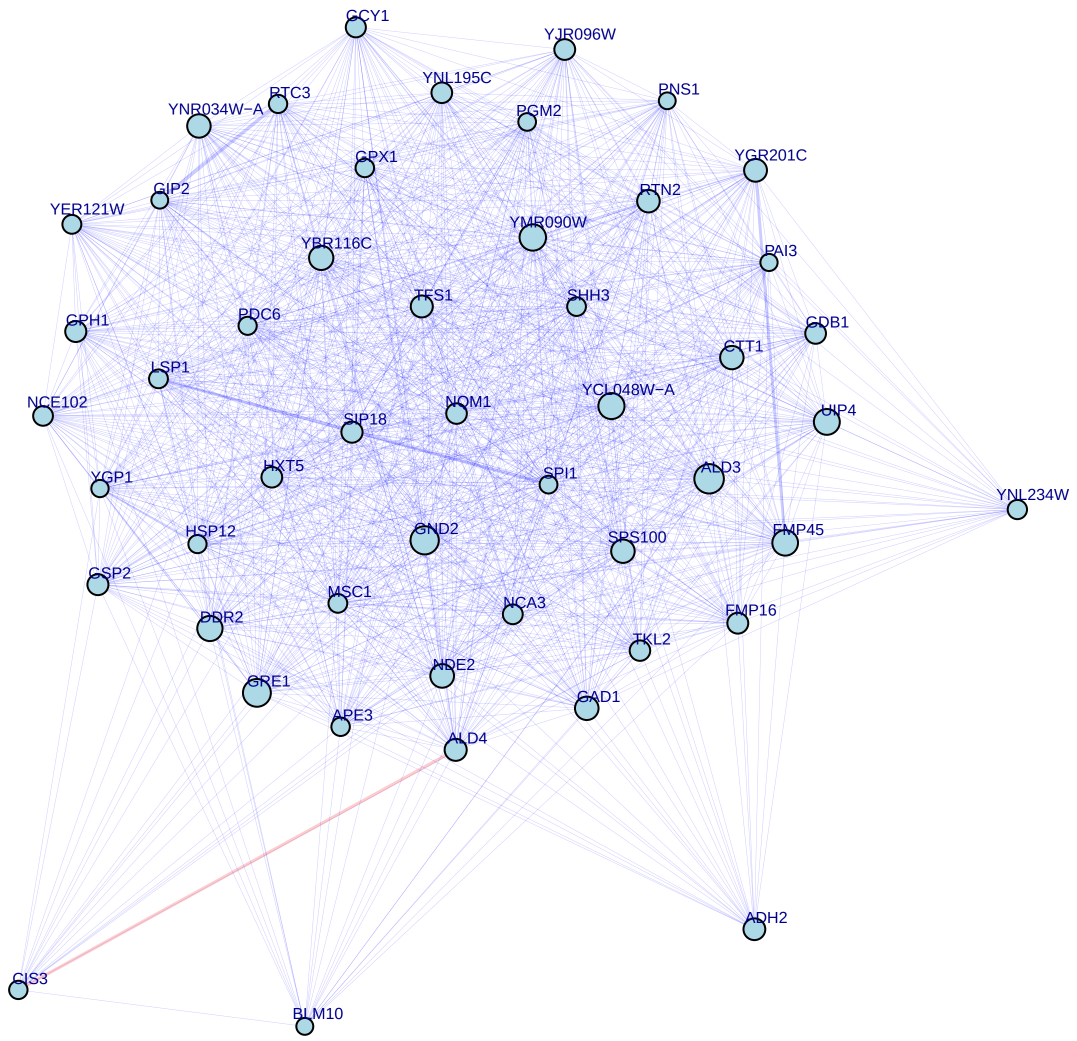

chrXV:171150_T/C top 50 down genes

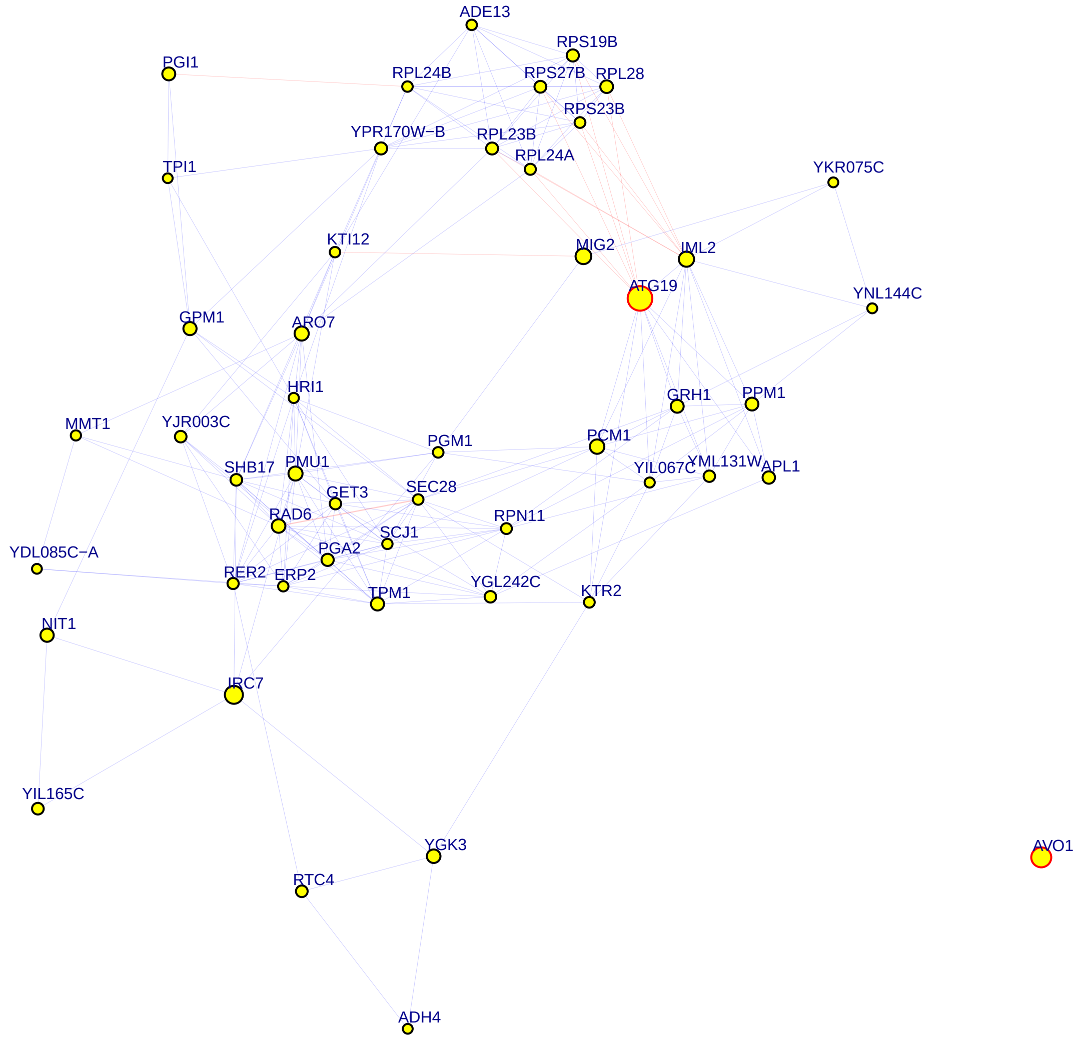

chrXV:171150_T/C top 100 up and down genes

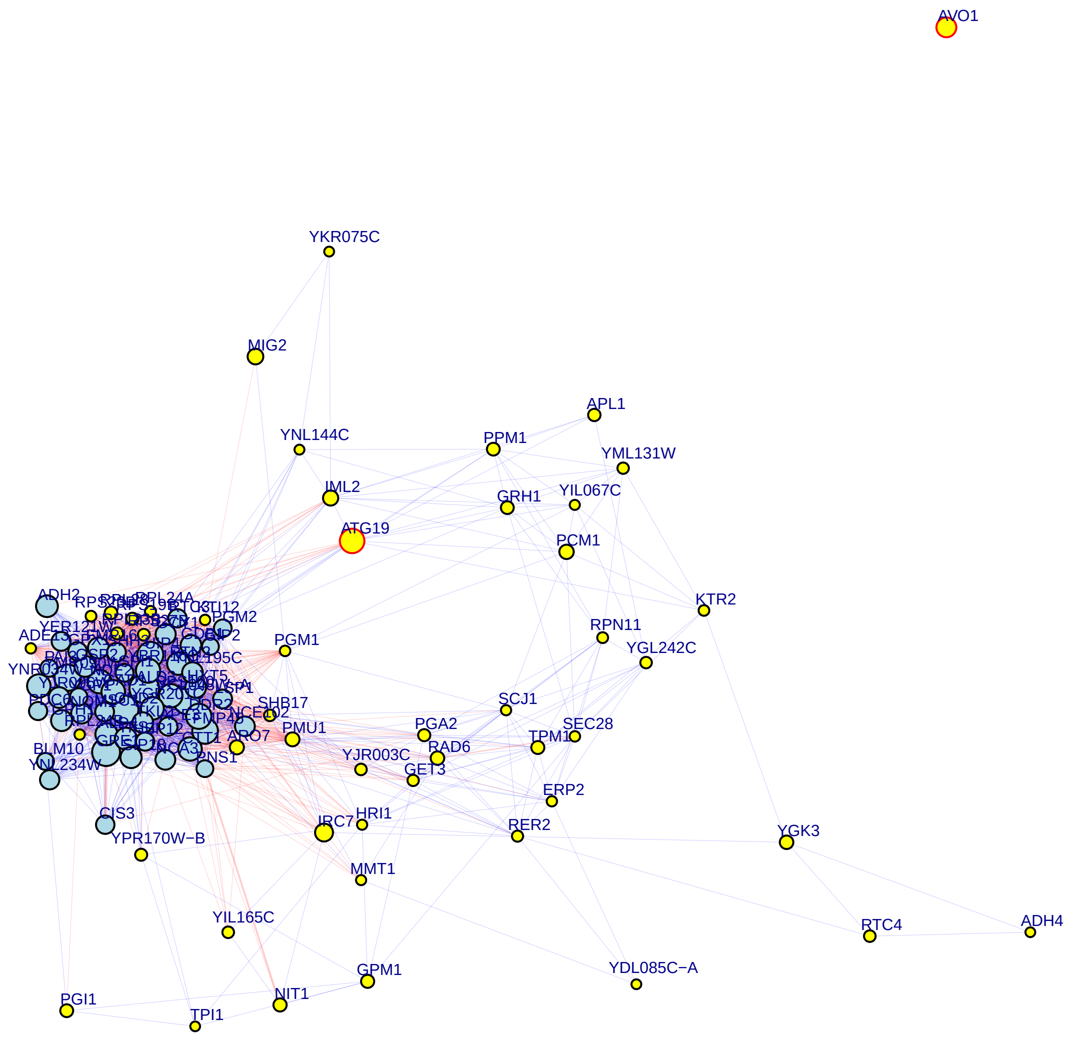

chrXV:377605_C/T top 50 up genes

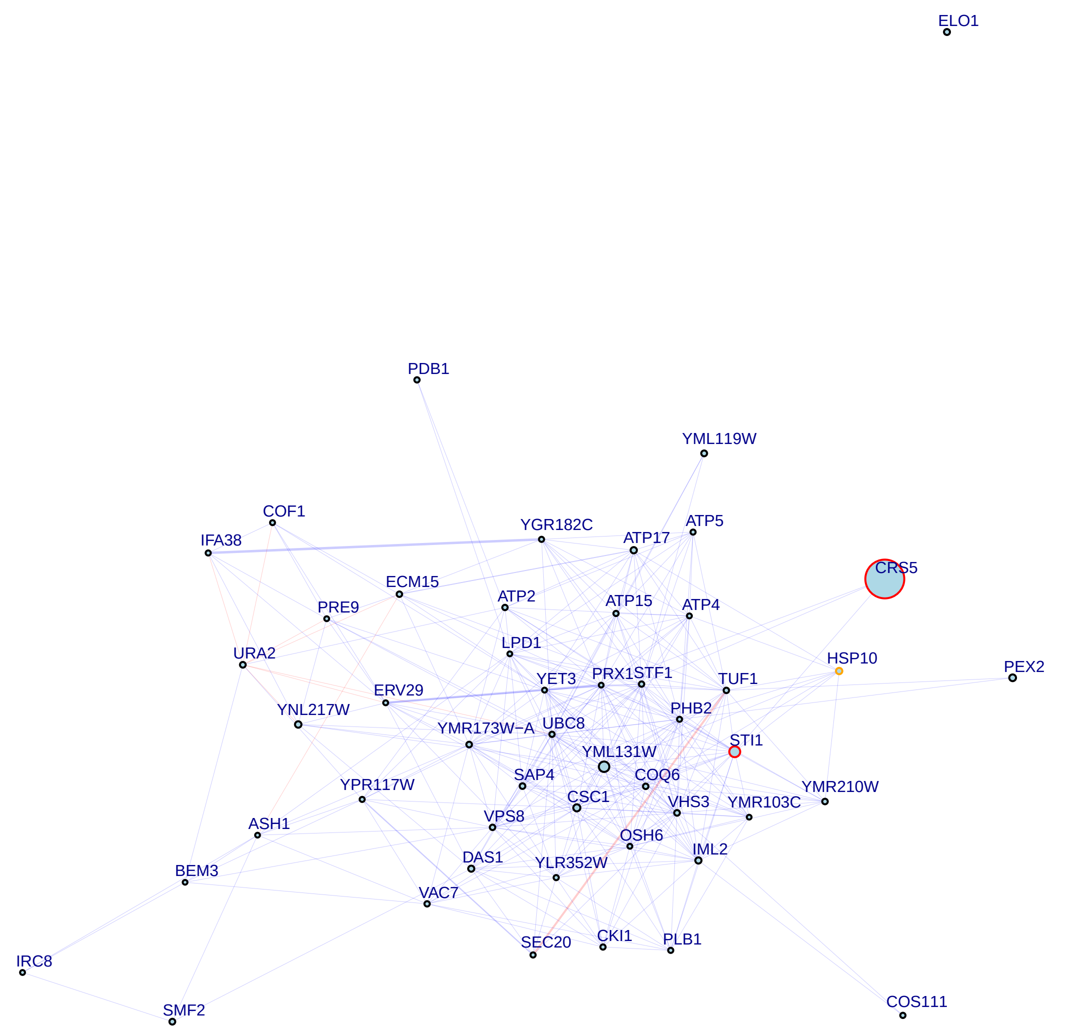

chrXV:377605_C/T top 50 down genes

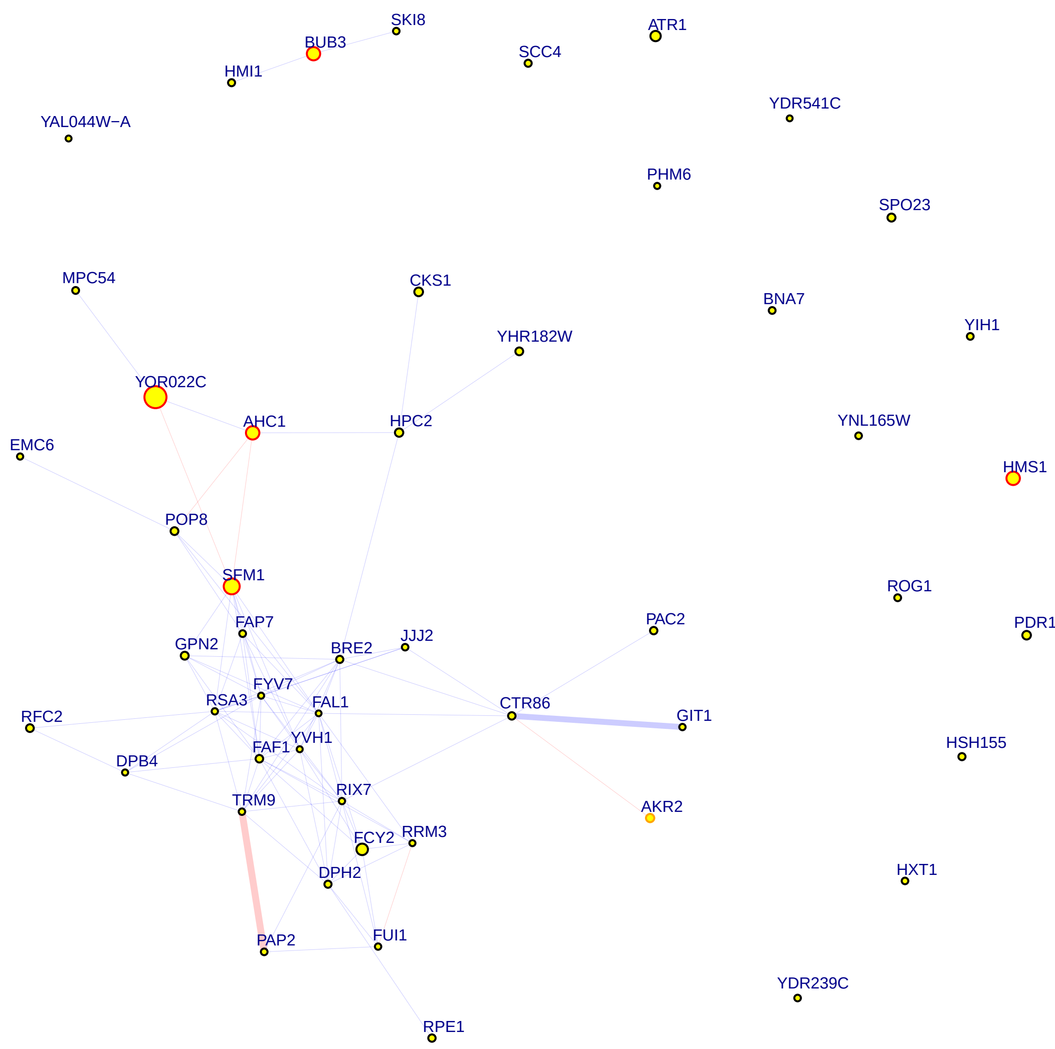

chrXV:377605_C/T top 100 up and down genes

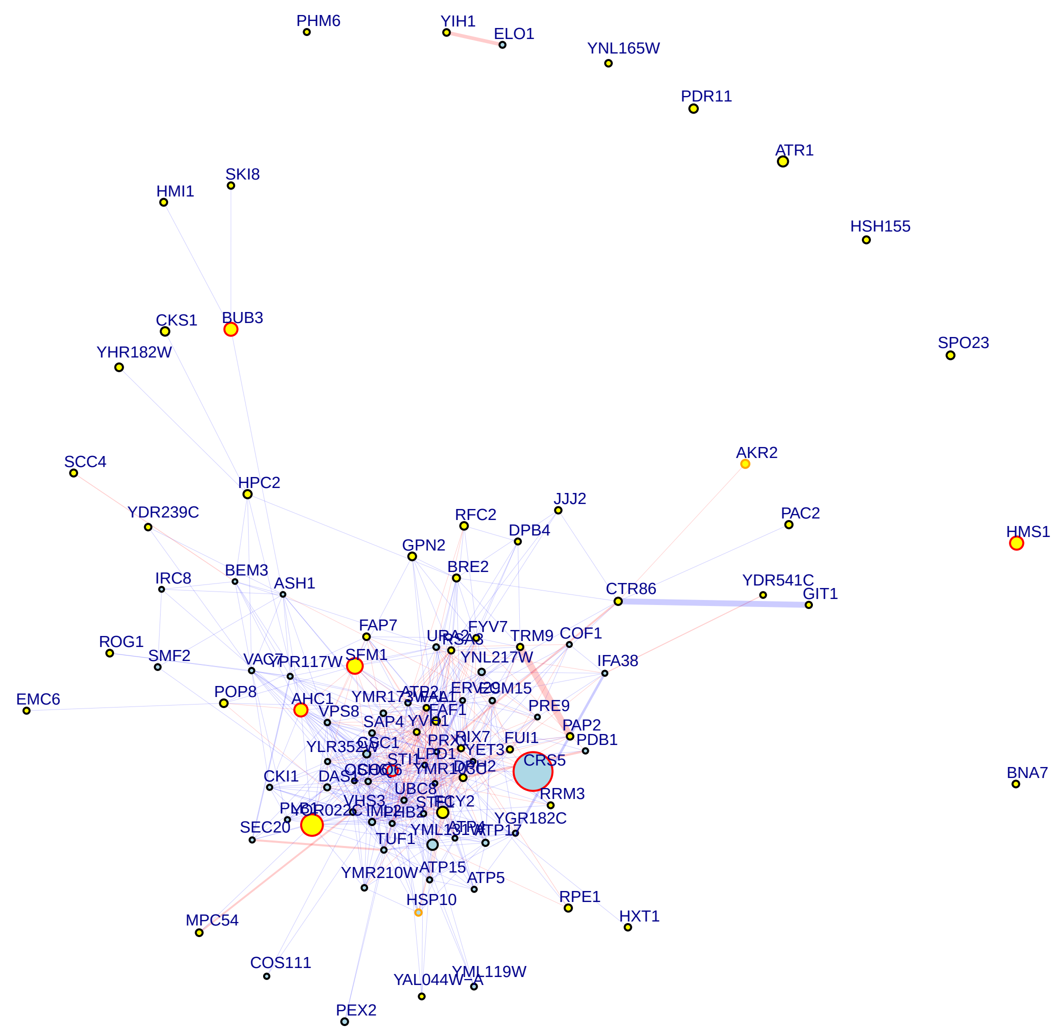

chrXV:521864_C/T top 50 up genes

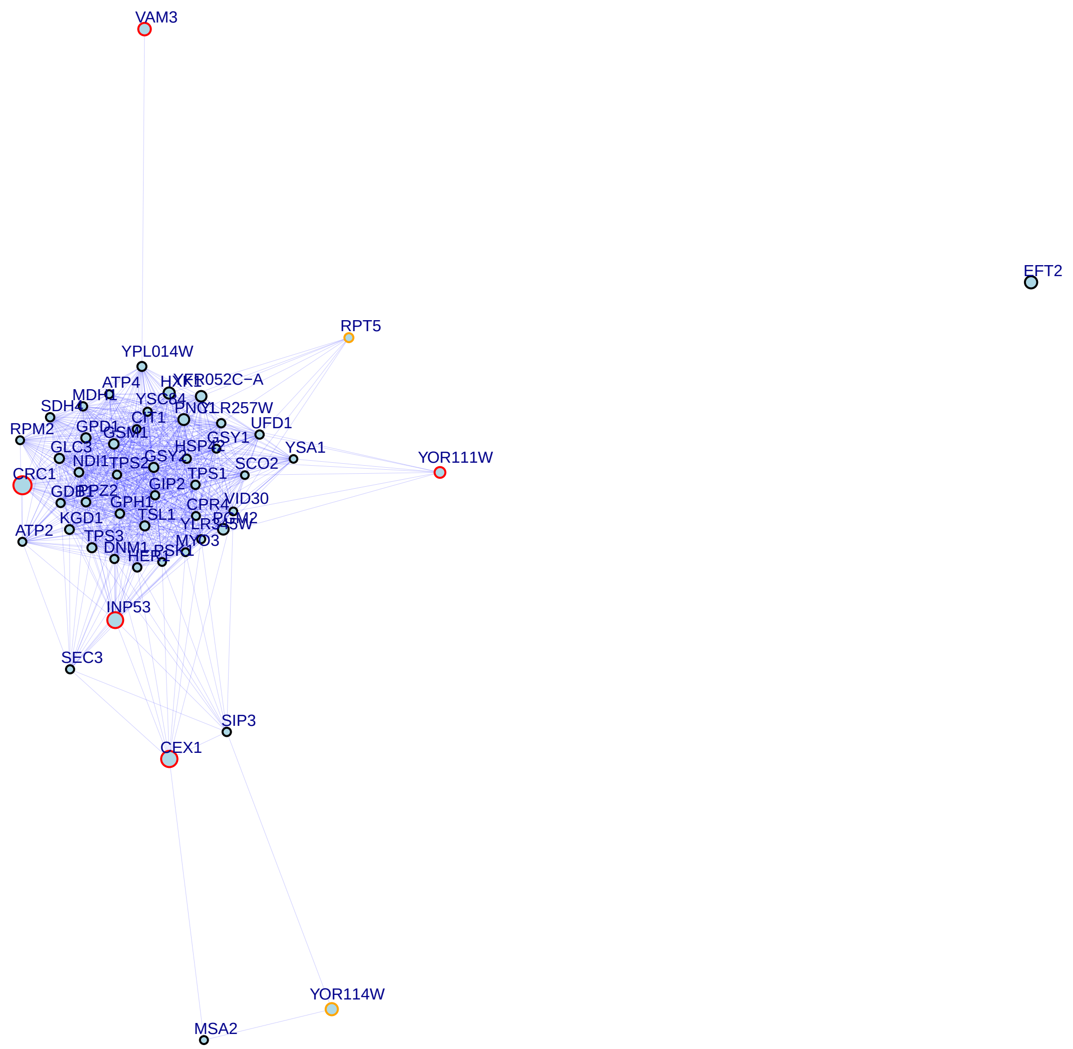

chrXV:521864_C/T top 50 down genes

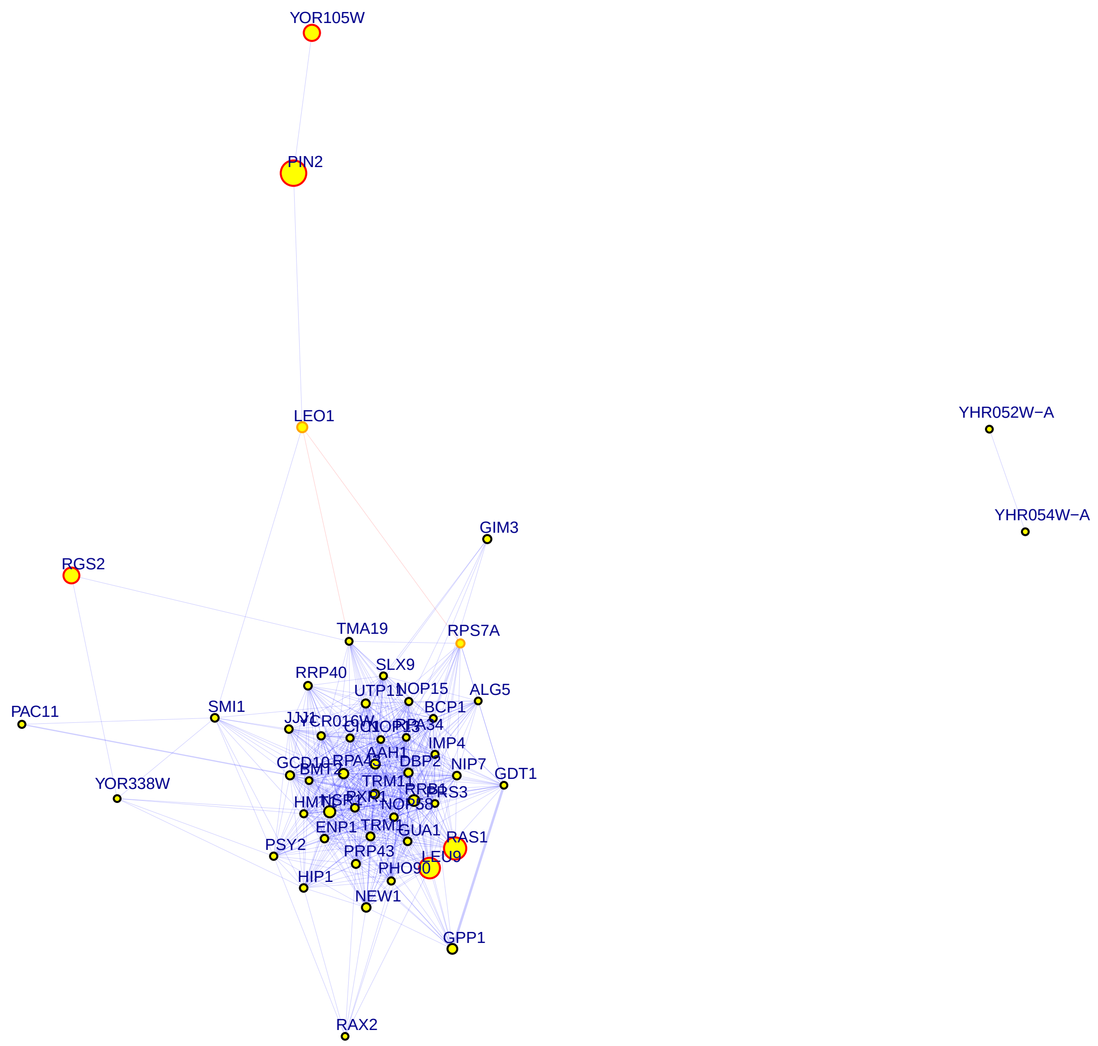

chrXV:521864_C/T top 100 up and down genes

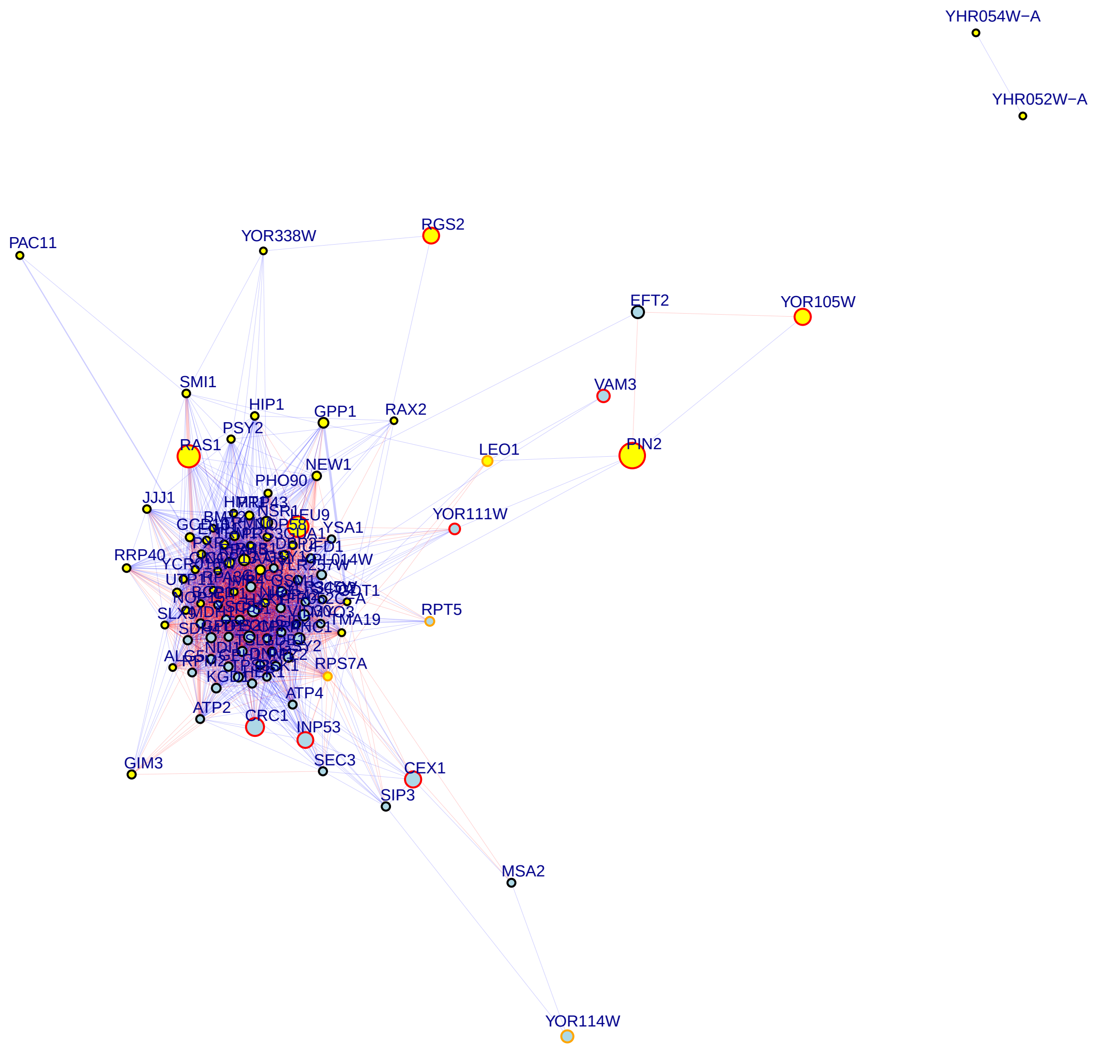

chrXV:589481_C/T top 50 up genes

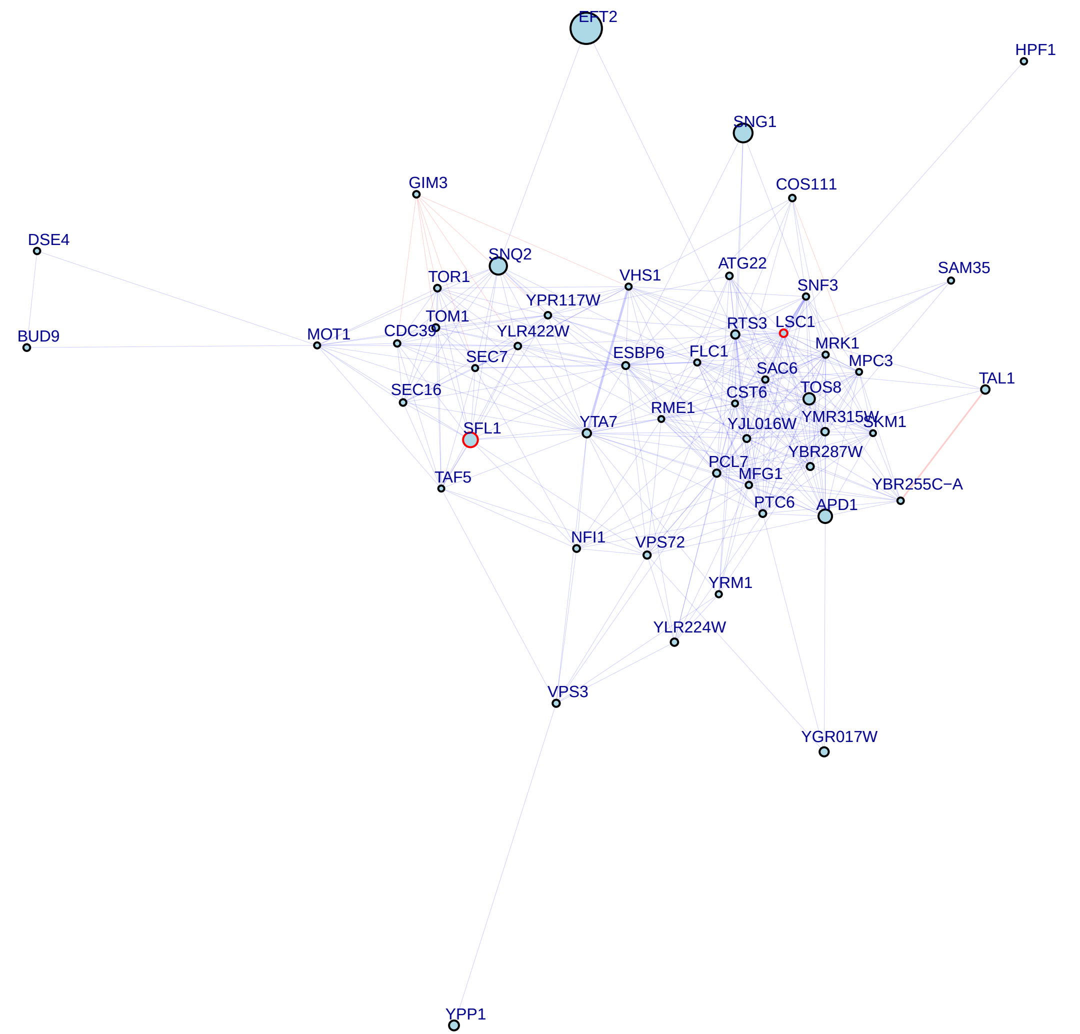

chrXV:589481_C/T top 50 down genes

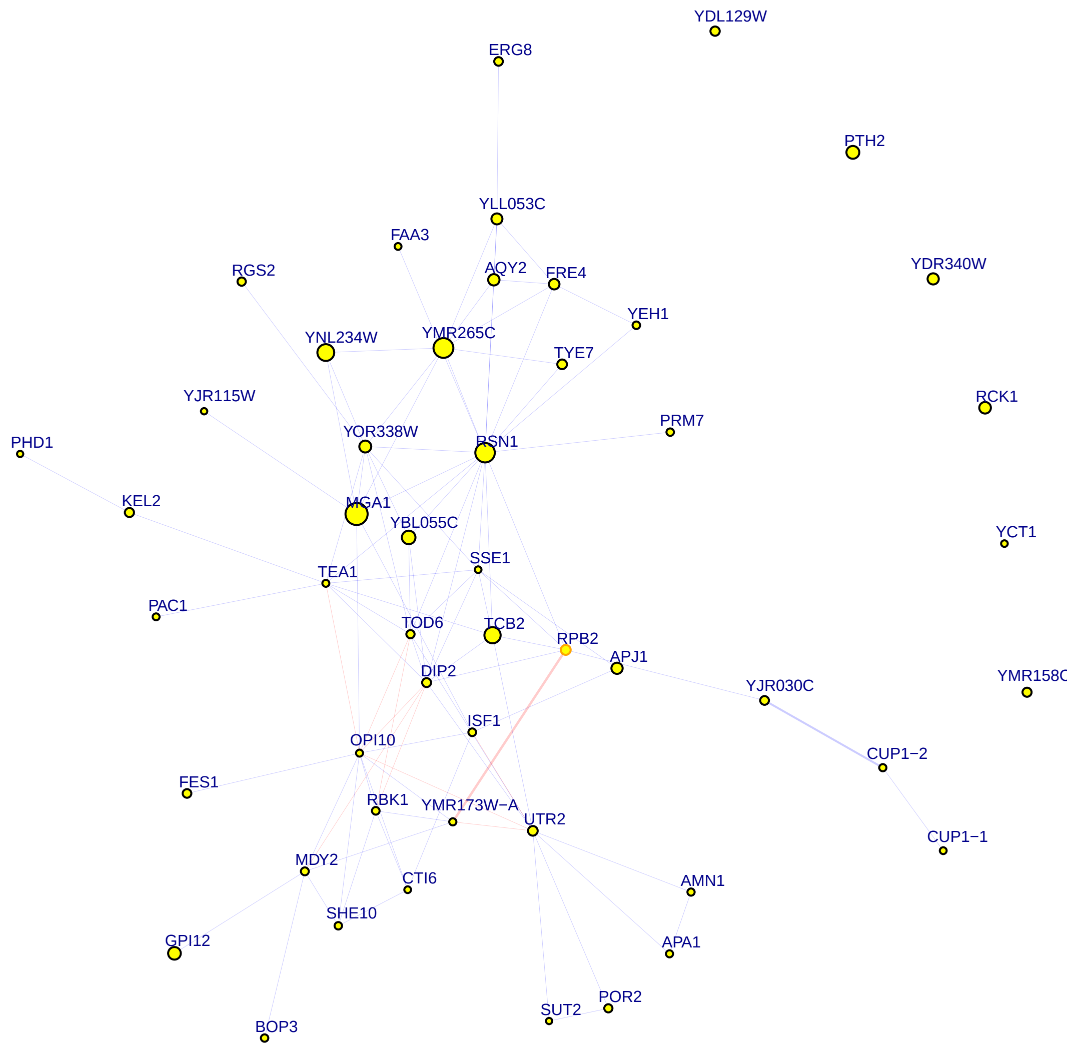

chrXV:589481_C/T top 100 up and down genes

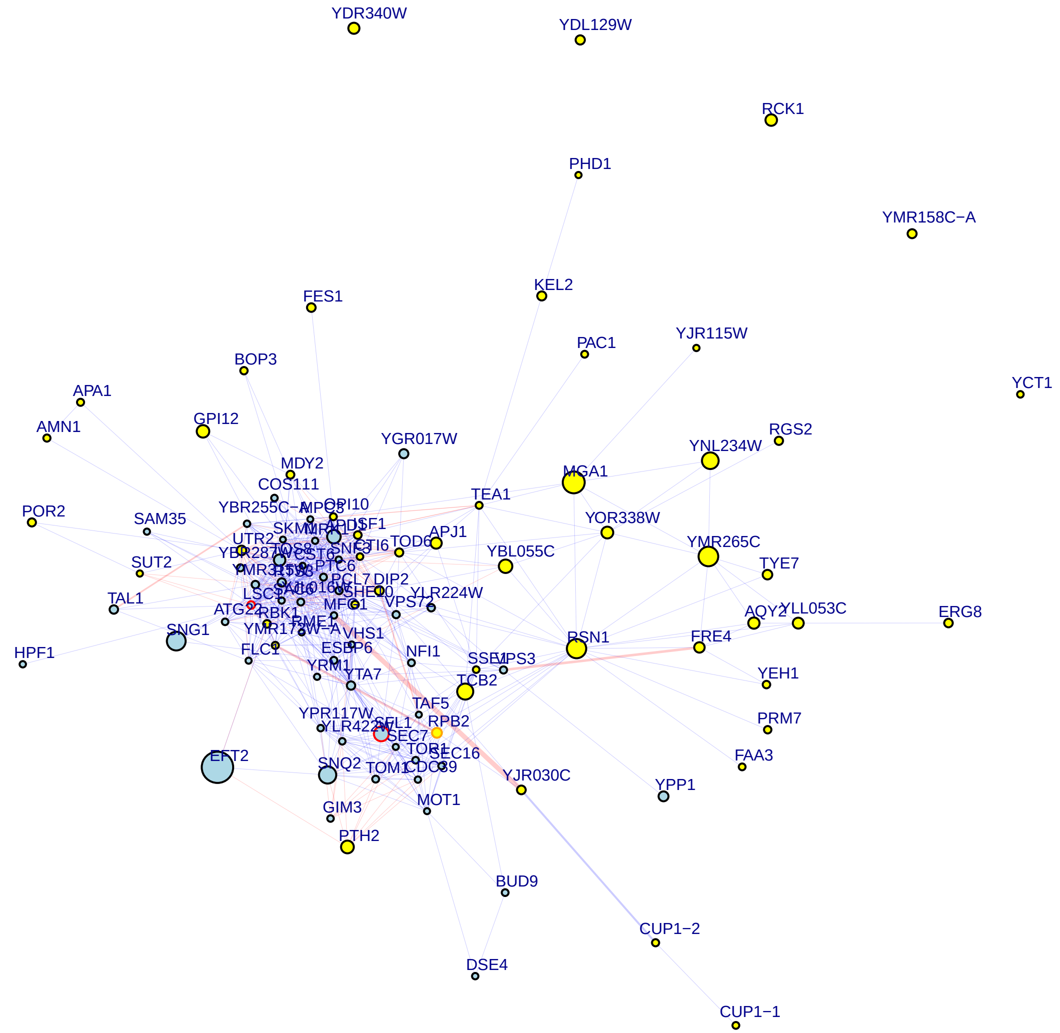

chrXV:725113_T/C top 50 up genes

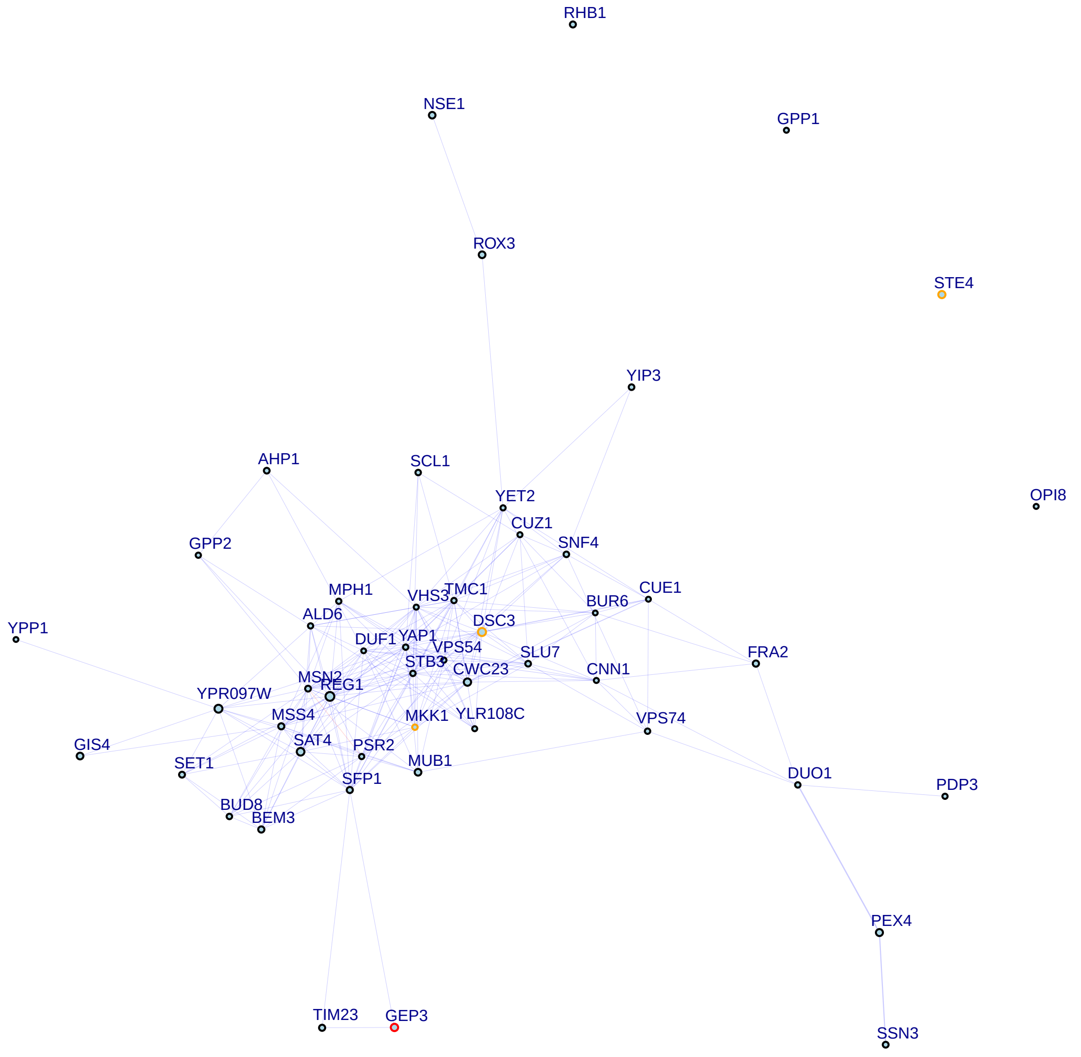

chrXV:725113_T/C top 50 down genes

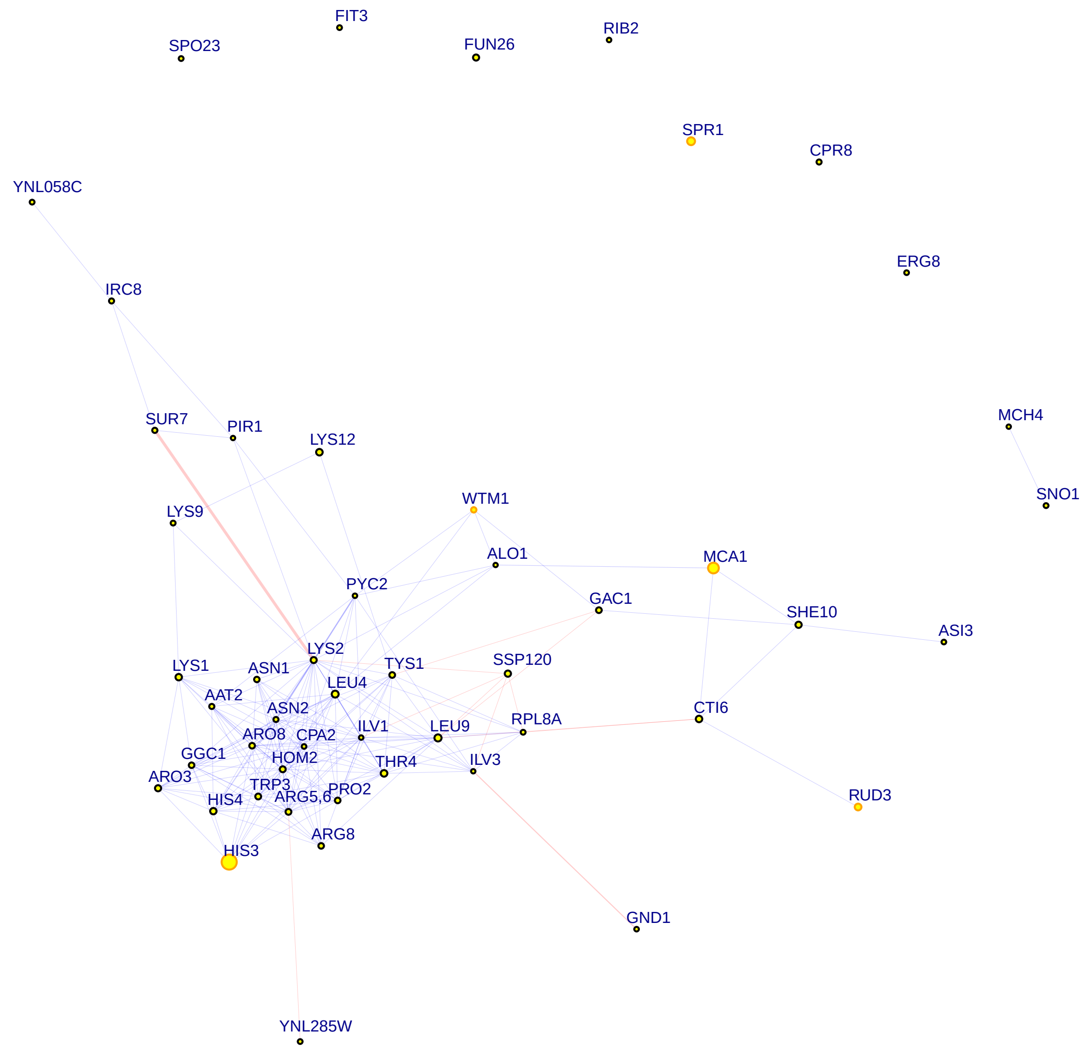

chrXV:725113_T/C top 100 up and down genes

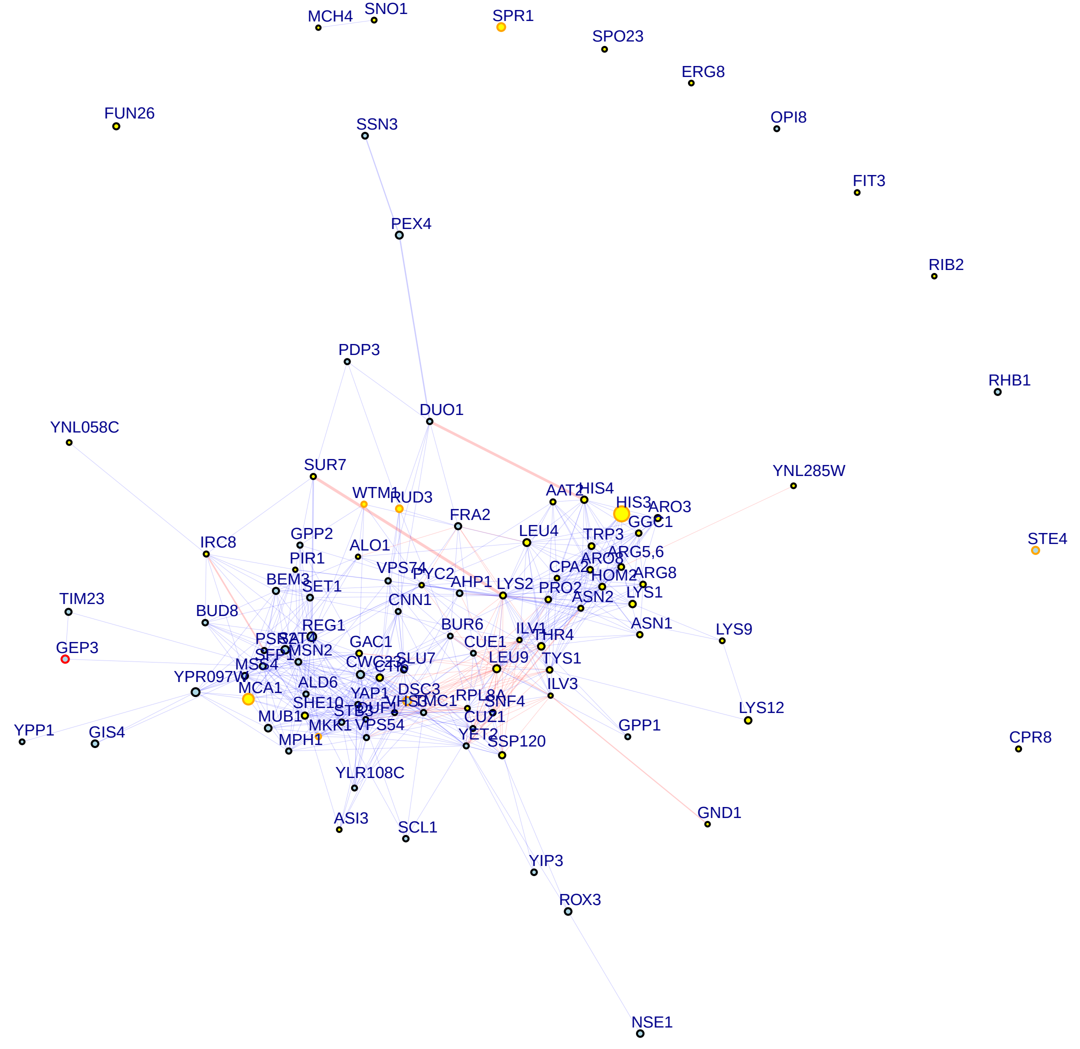

chrXV:845199_G/A top 50 up genes

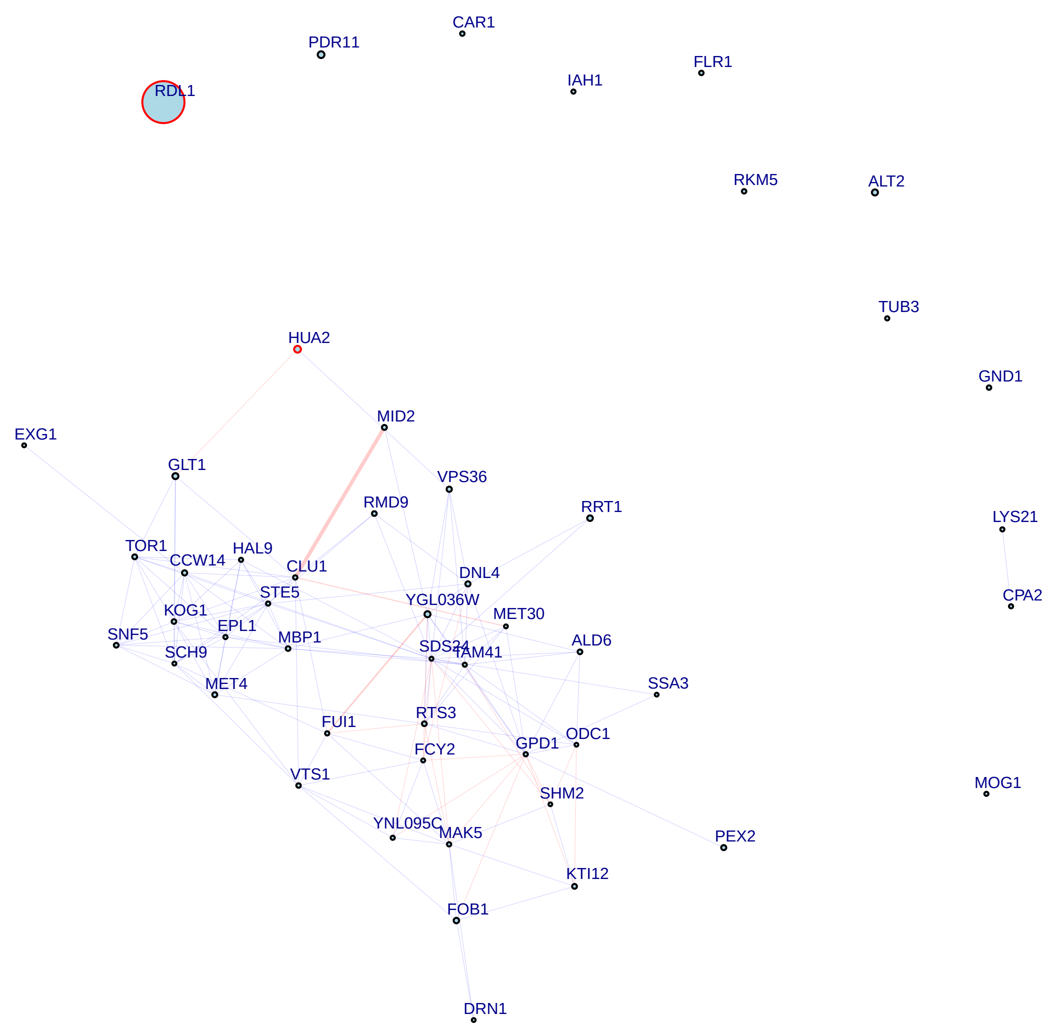

chrXV:845199_G/A top 50 down genes

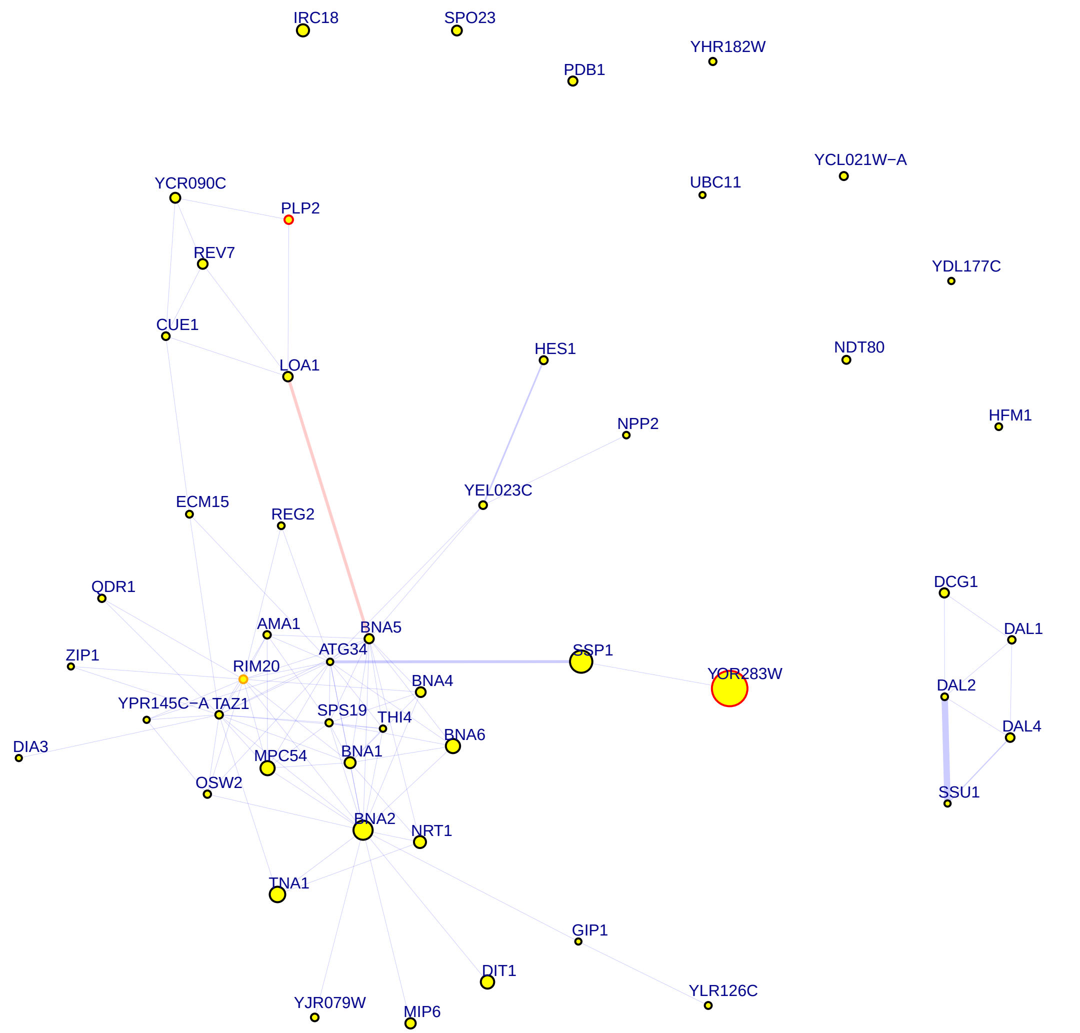

chrXV:845199_G/A top 100 up and down genes

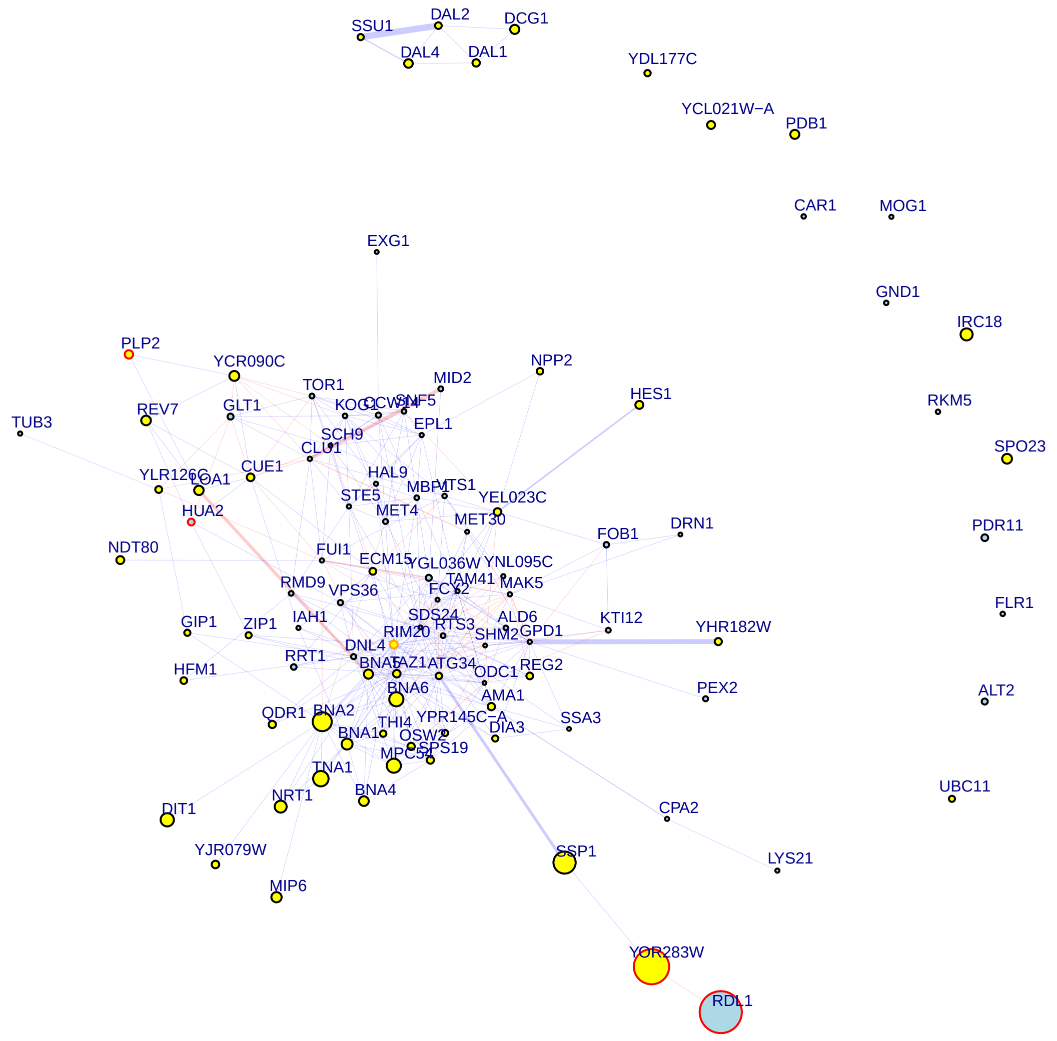

chrXV:958080_T/A top 50 up genes

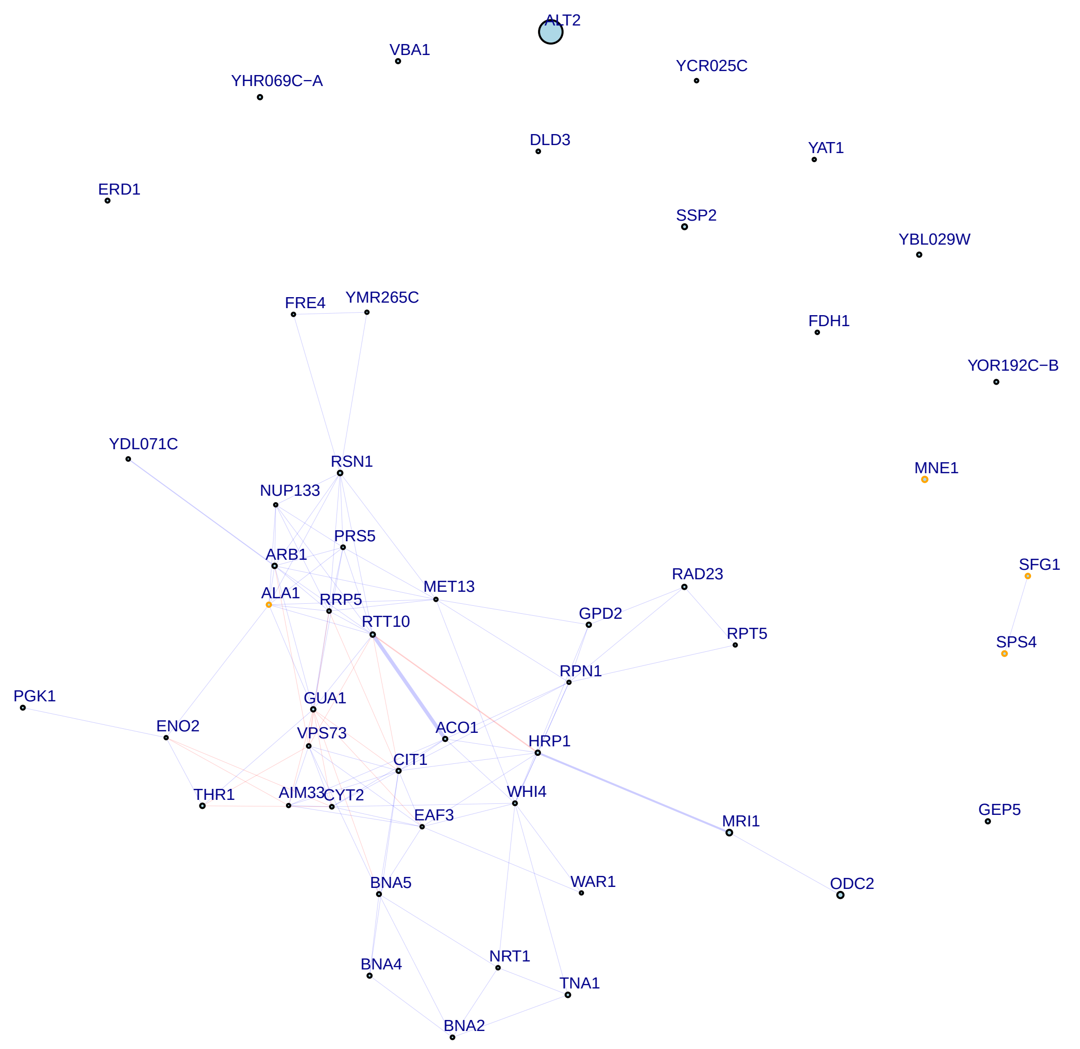

chrXV:958080_T/A top 50 down genes

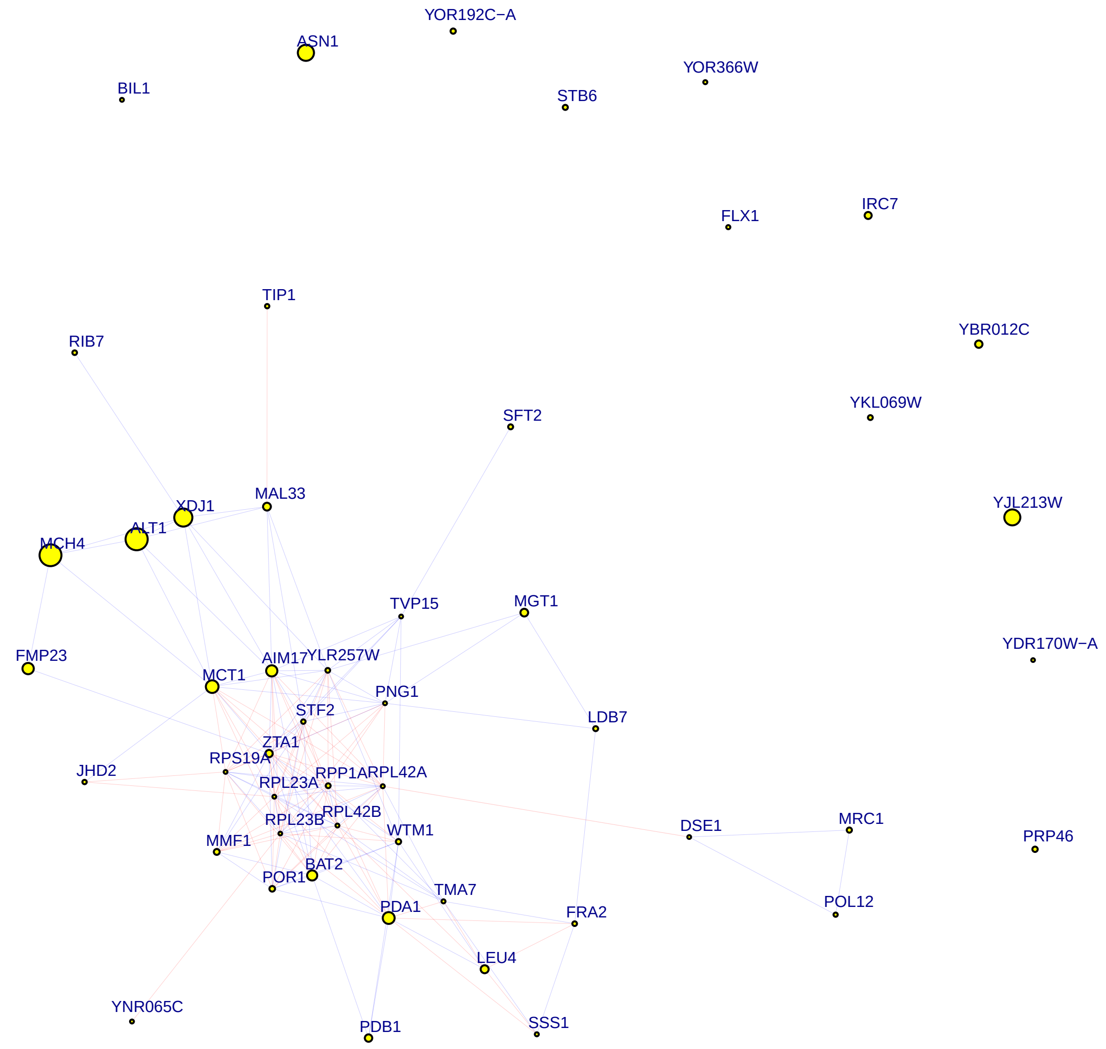

chrXV:958080_T/A top 100 up and down genes

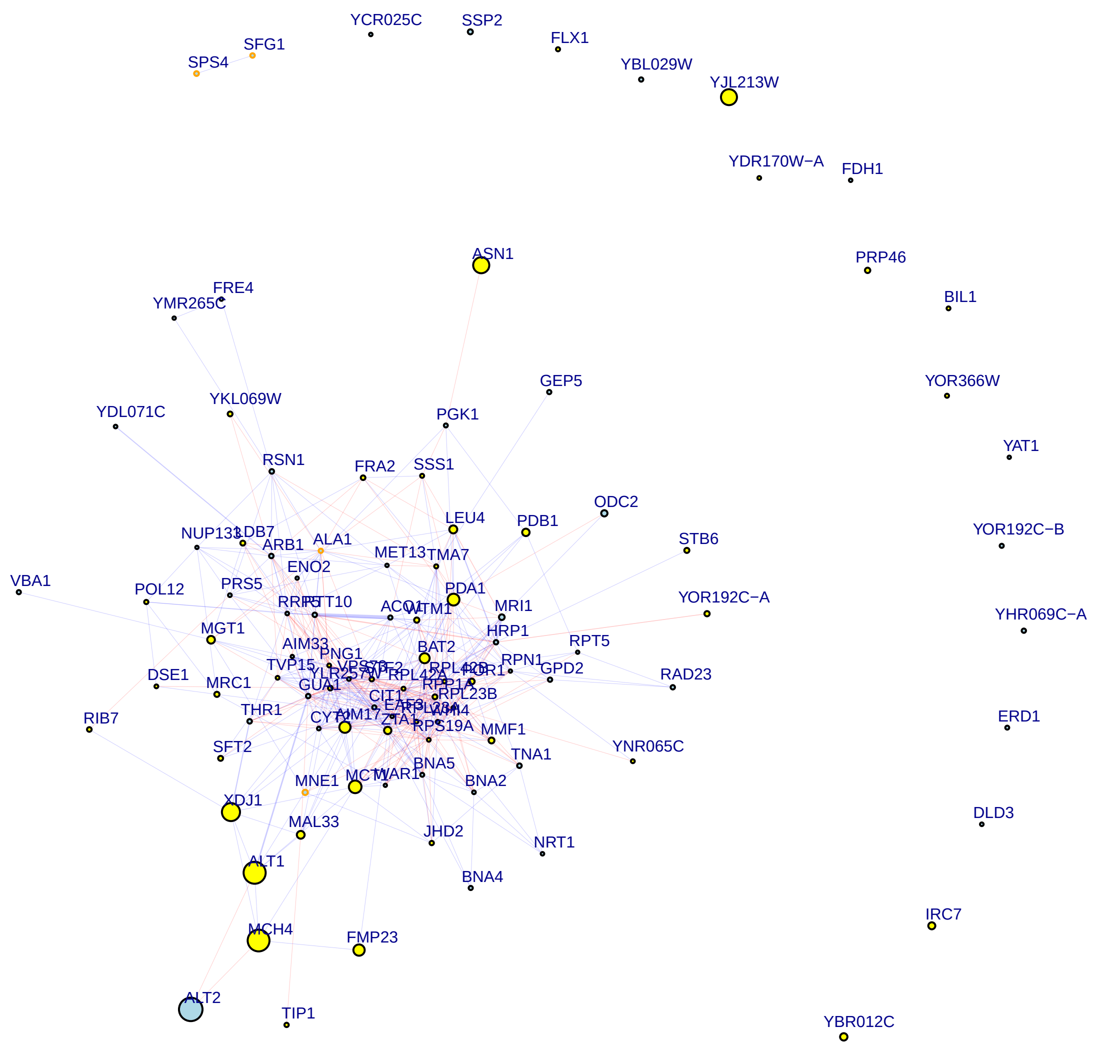

chrXV:1065815_A/G top 50 up genes

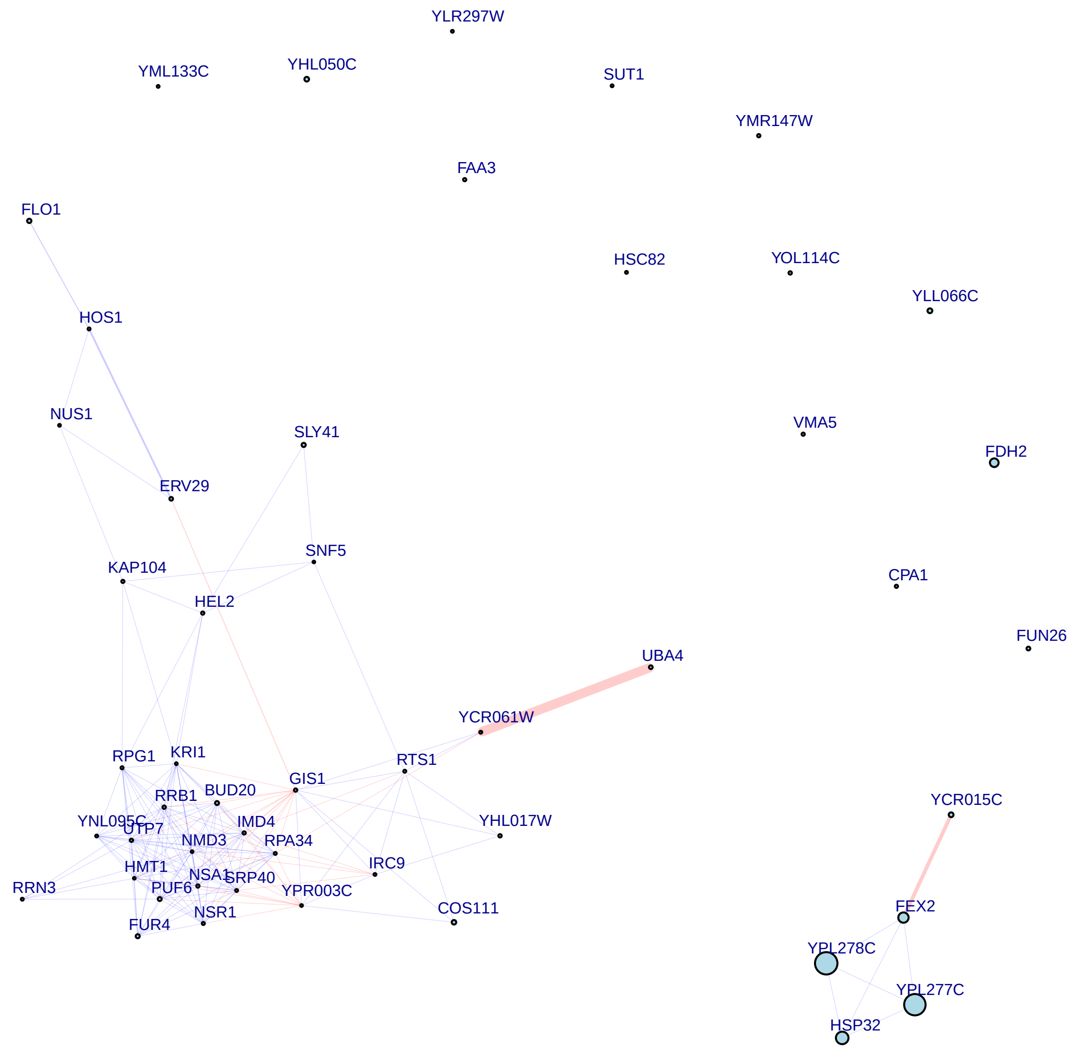

chrXV:1065815_A/G top 50 down genes

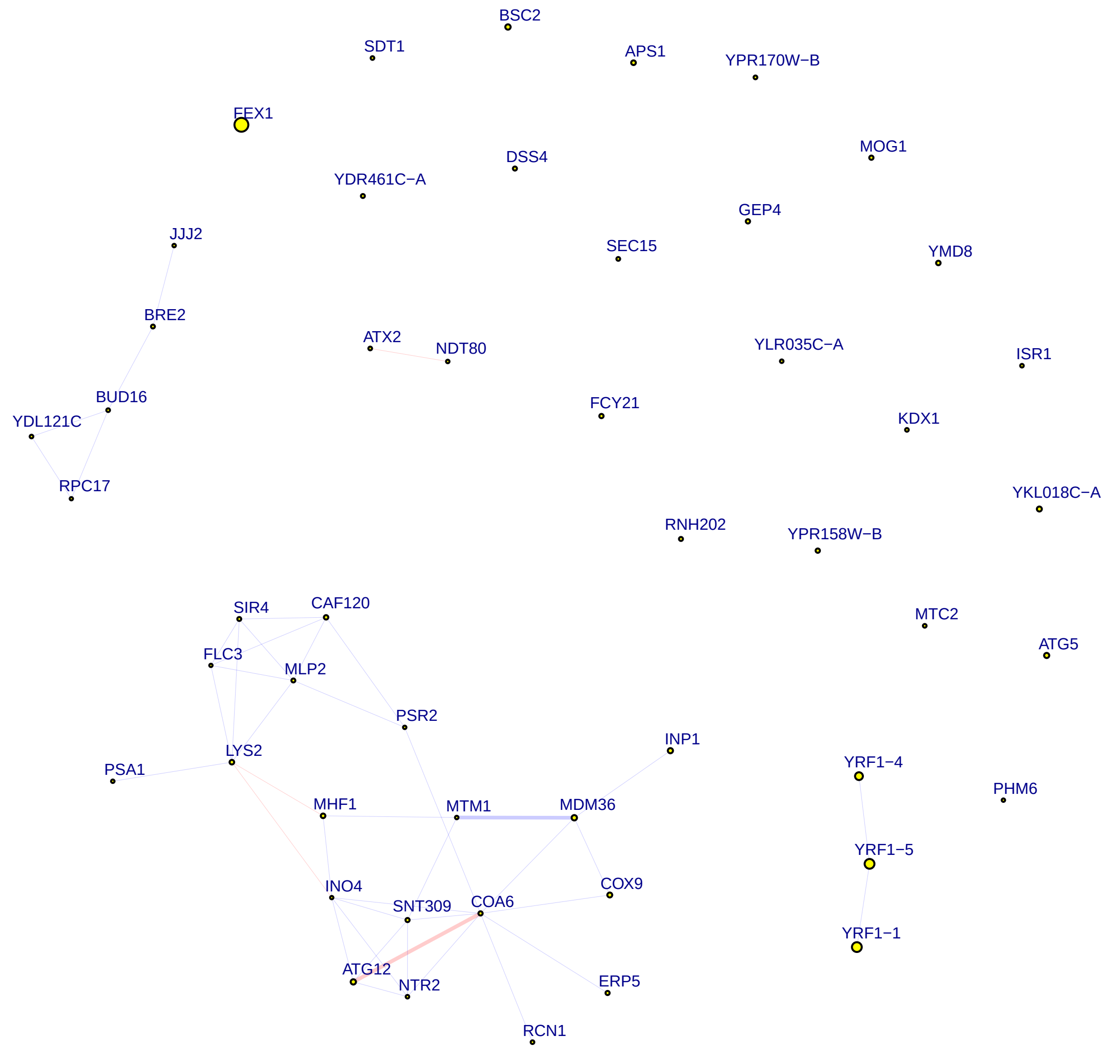

chrXV:1065815_A/G top 100 up and down genes

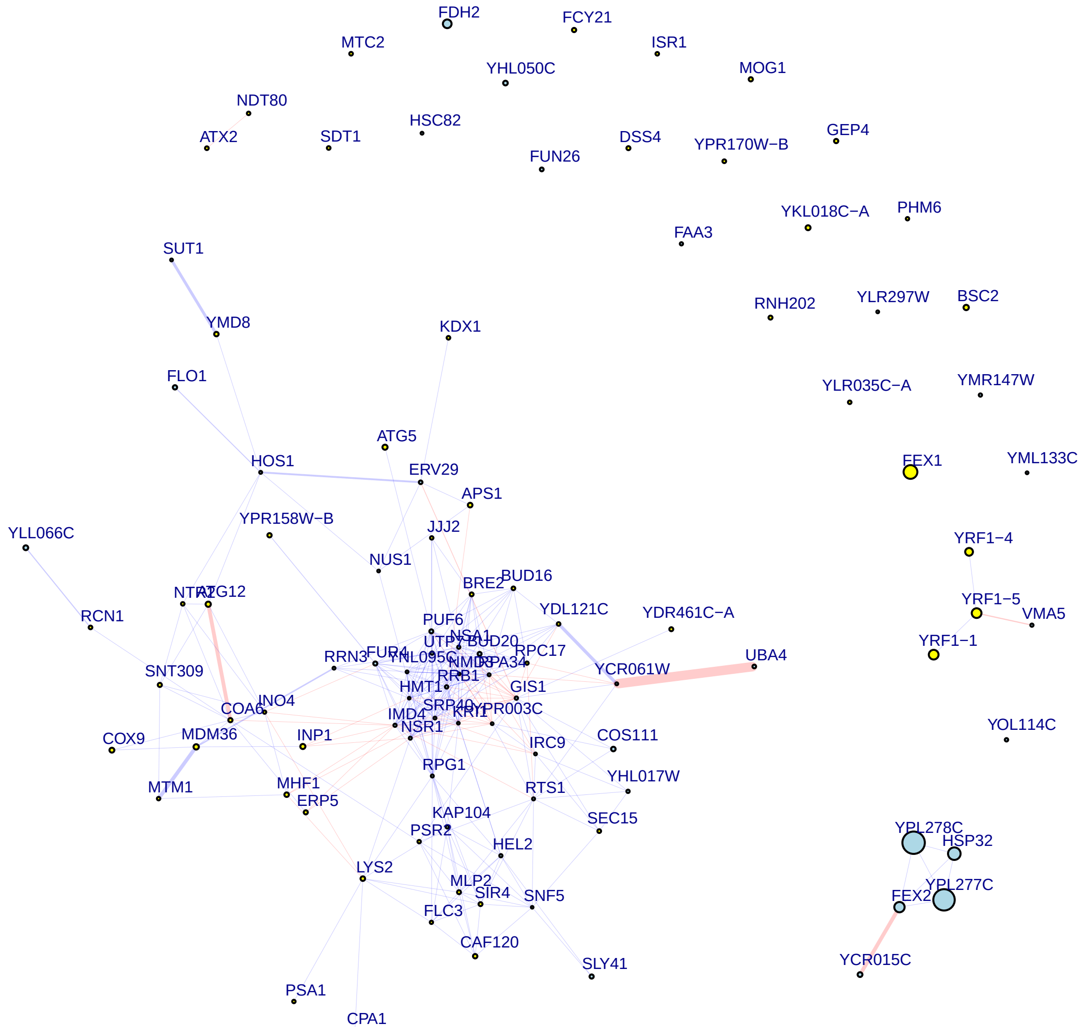

chrXVI:26510_T/C top 29 up genes

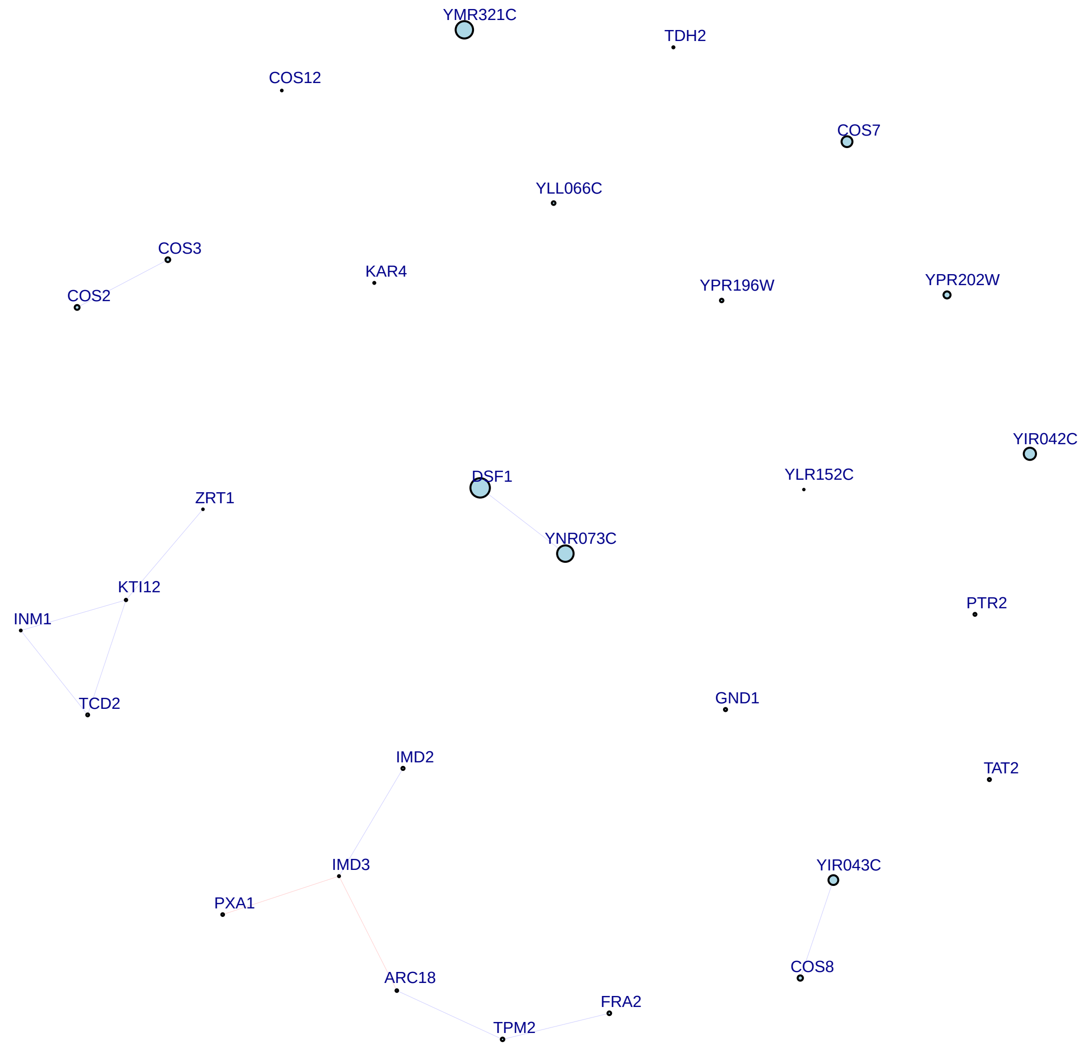

chrXVI:26510_T/C top 50 down genes

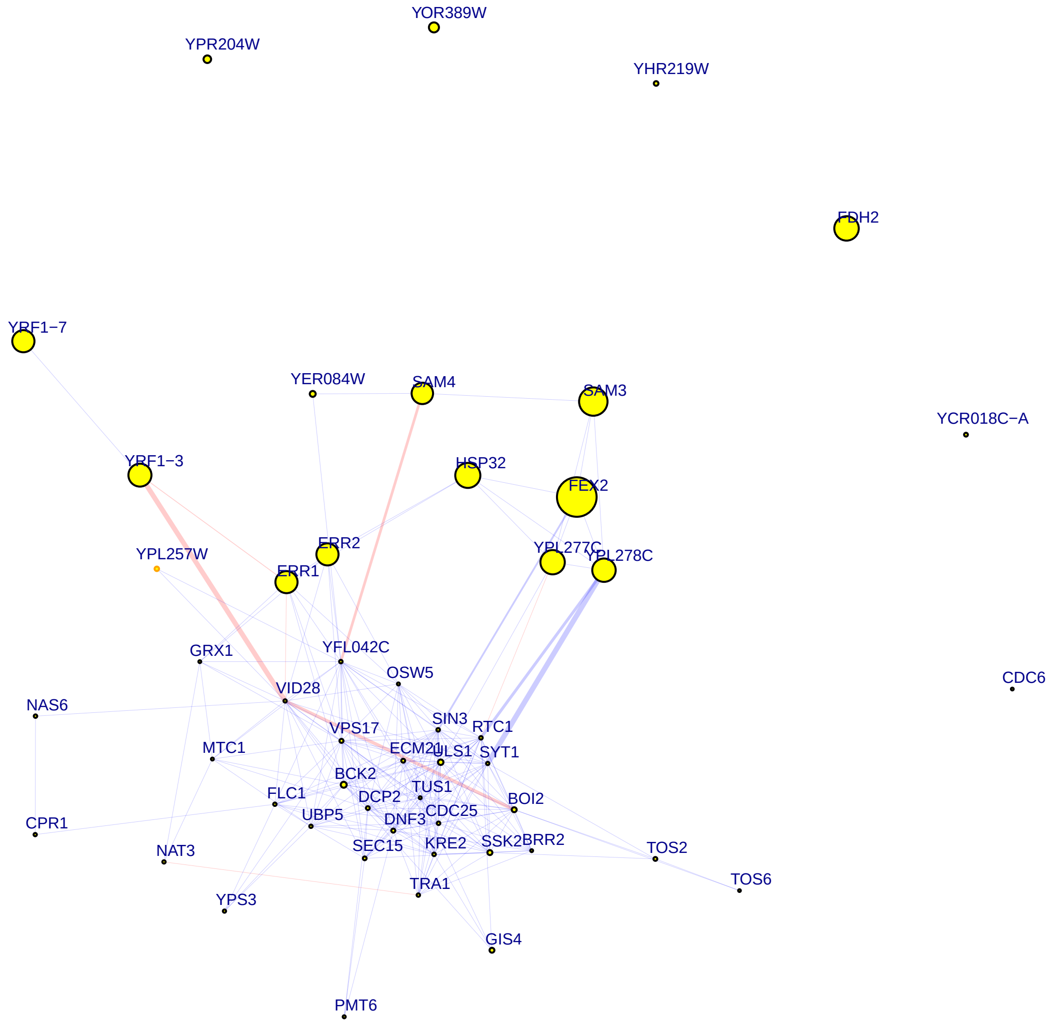

chrXVI:26510_T/C top 79 up and down genes

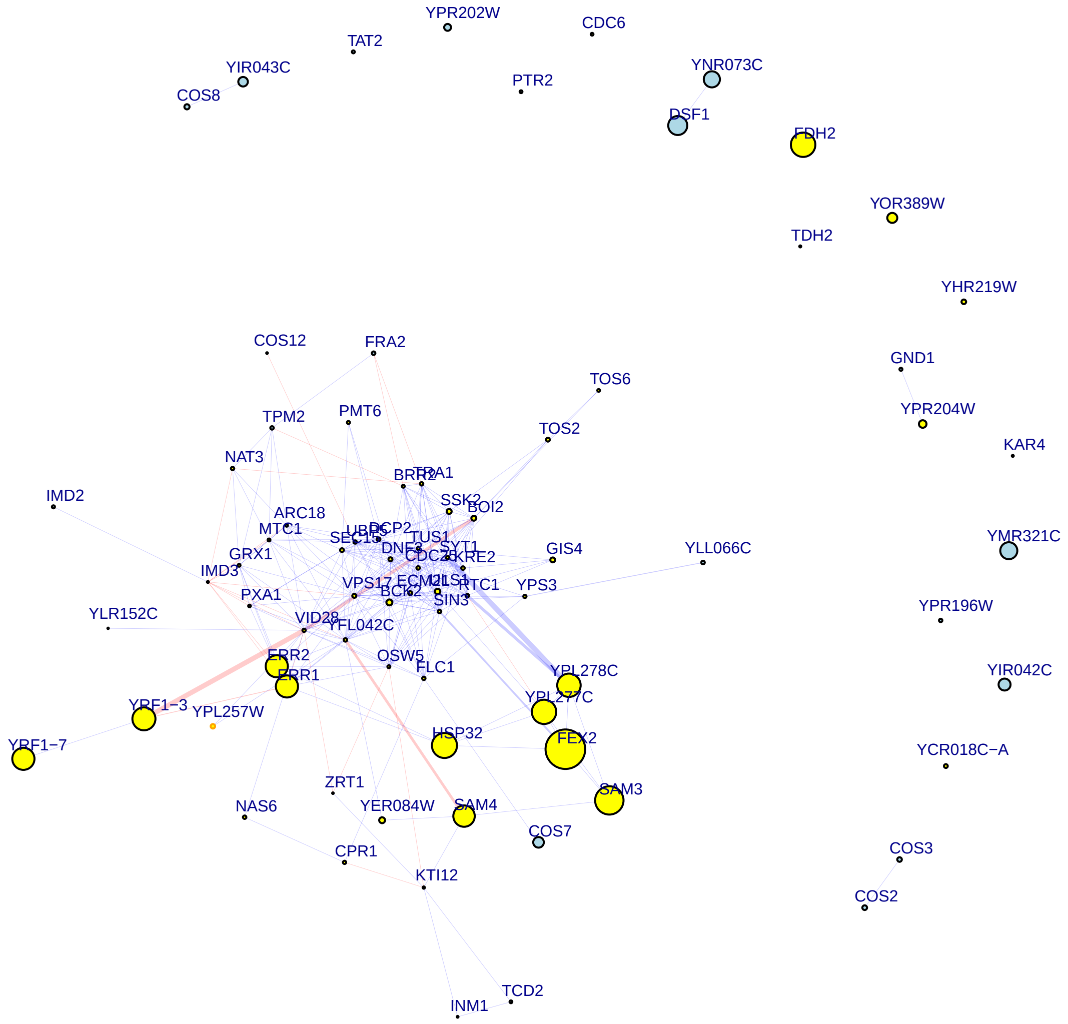

chrXVI:84945_G/A top 50 up genes

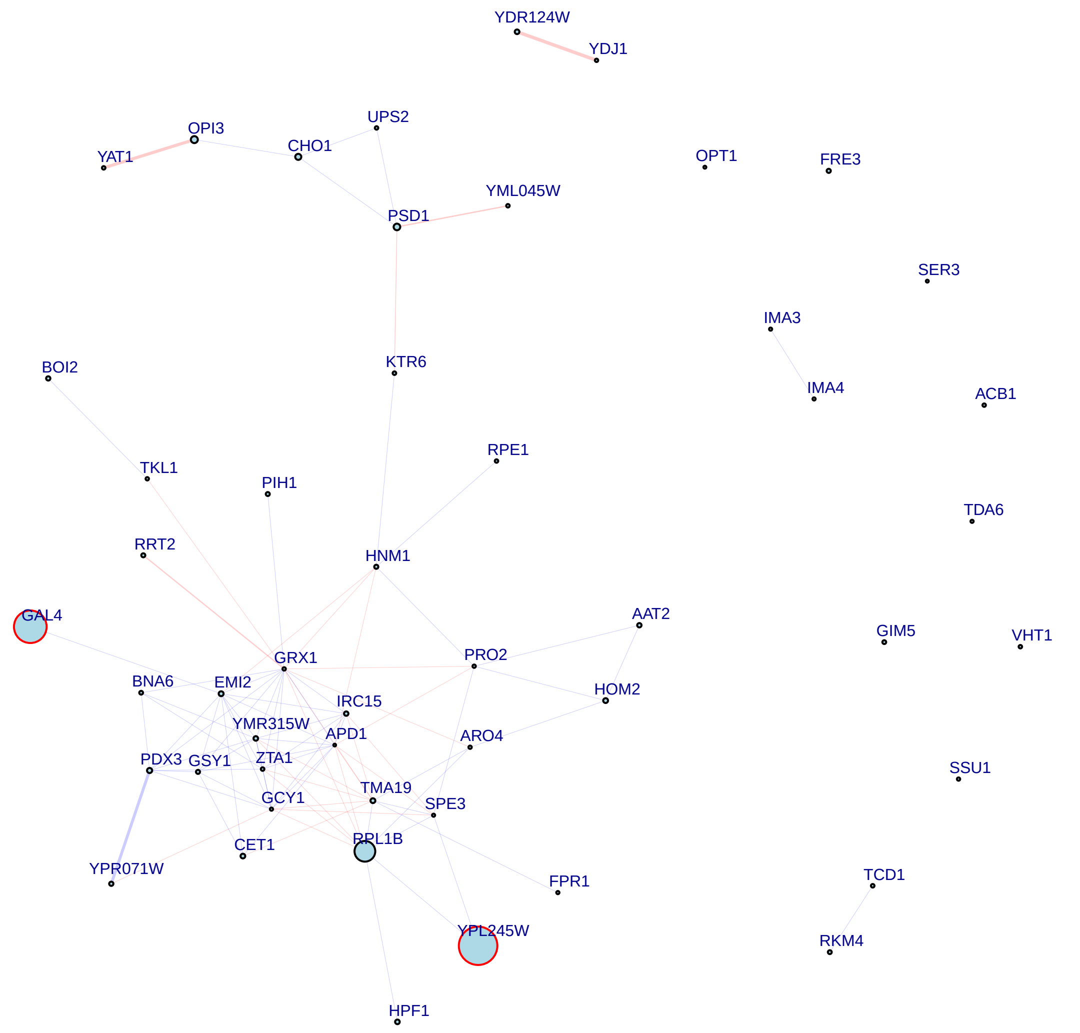

chrXVI:84945_G/A top 50 down genes

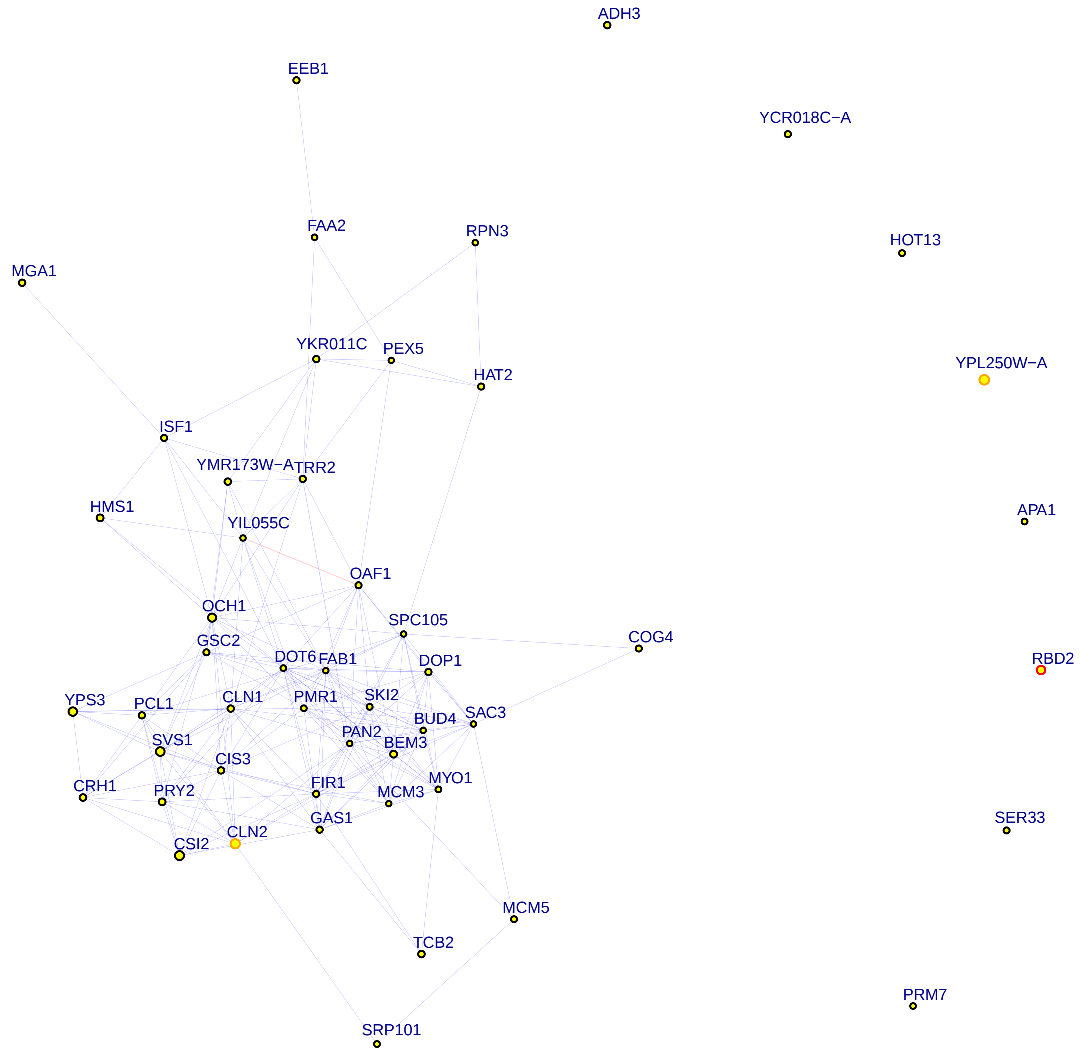

chrXVI:84945_G/A top 100 up and down genes

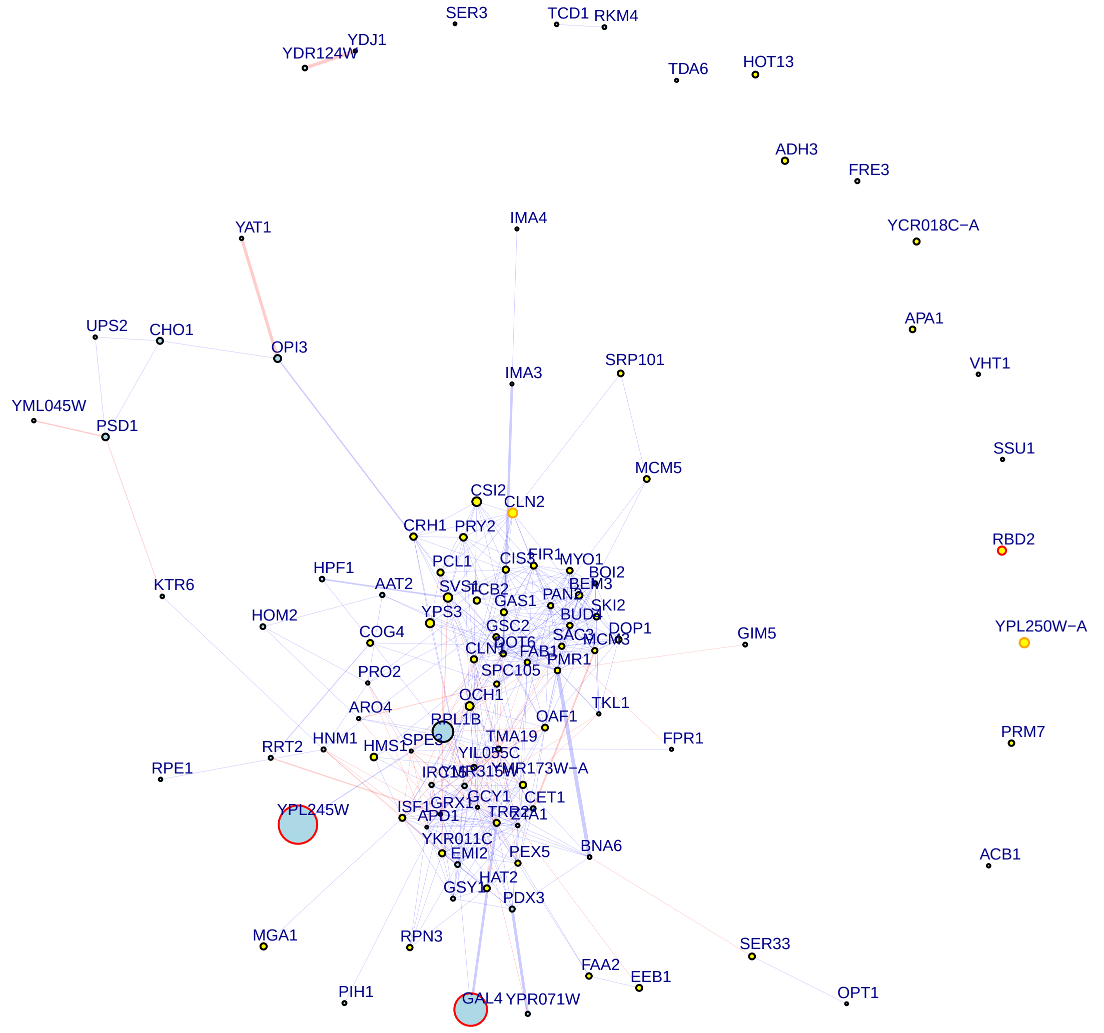

chrXVI:207364_C/T top 50 up genes

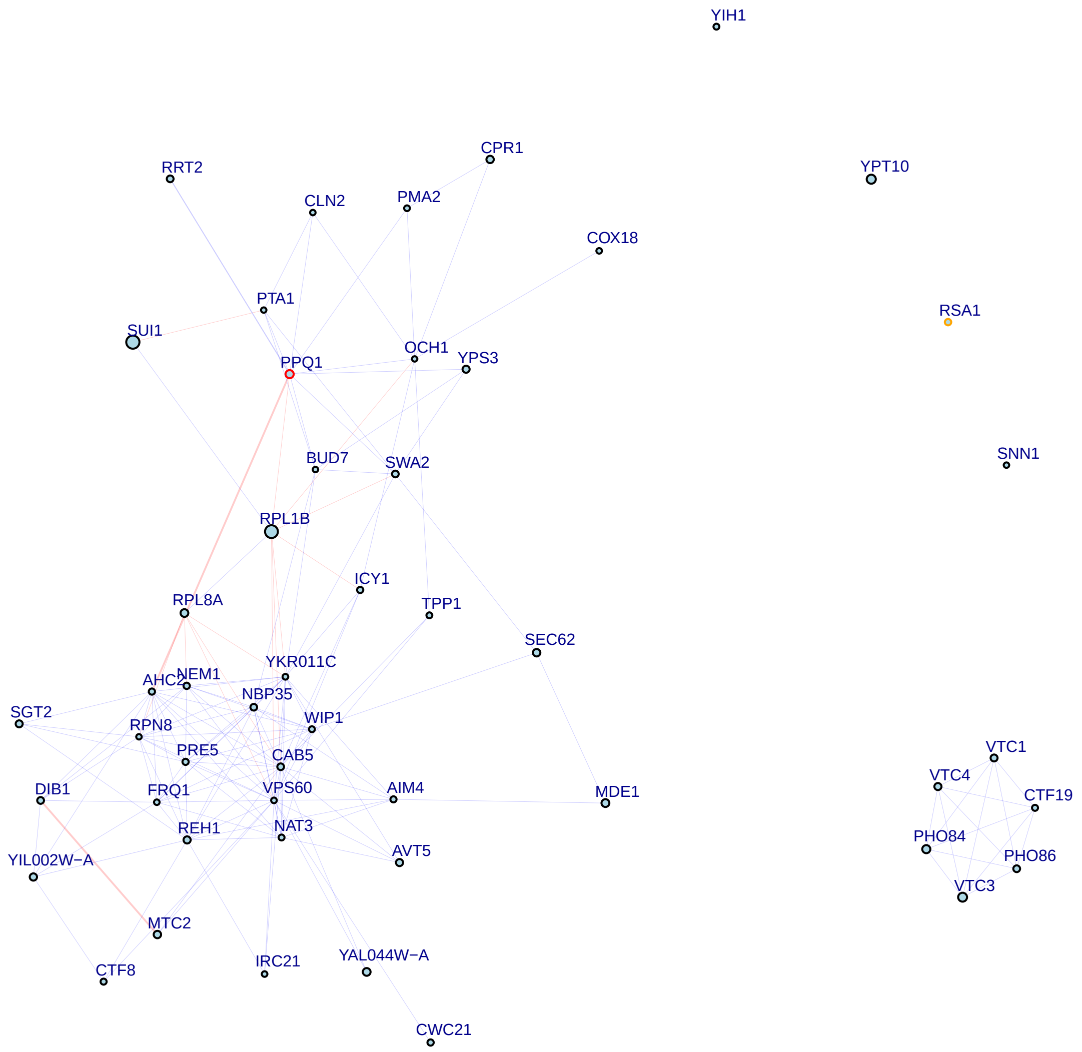

chrXVI:207364_C/T top 50 down genes

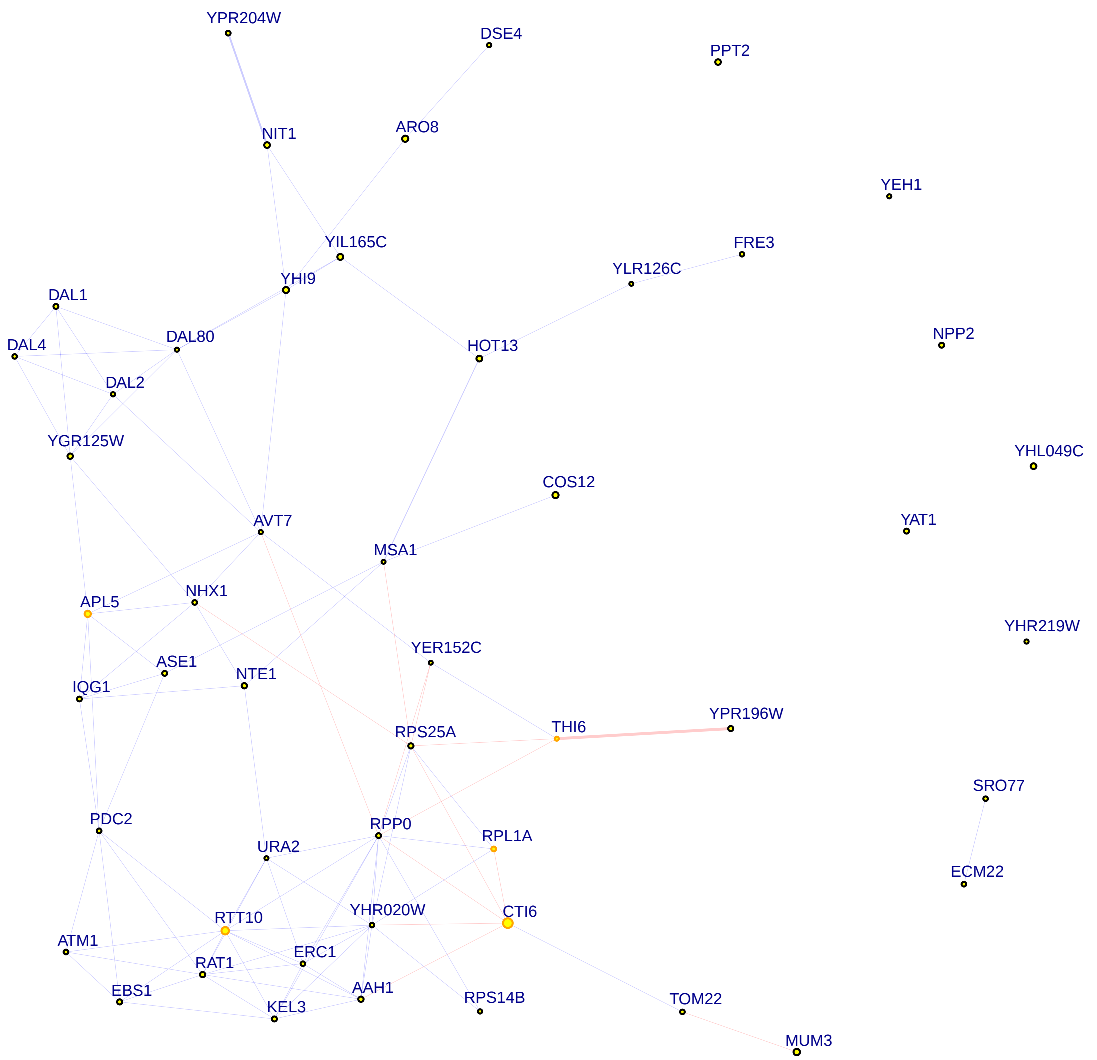

chrXVI:207364_C/T top 100 up and down genes

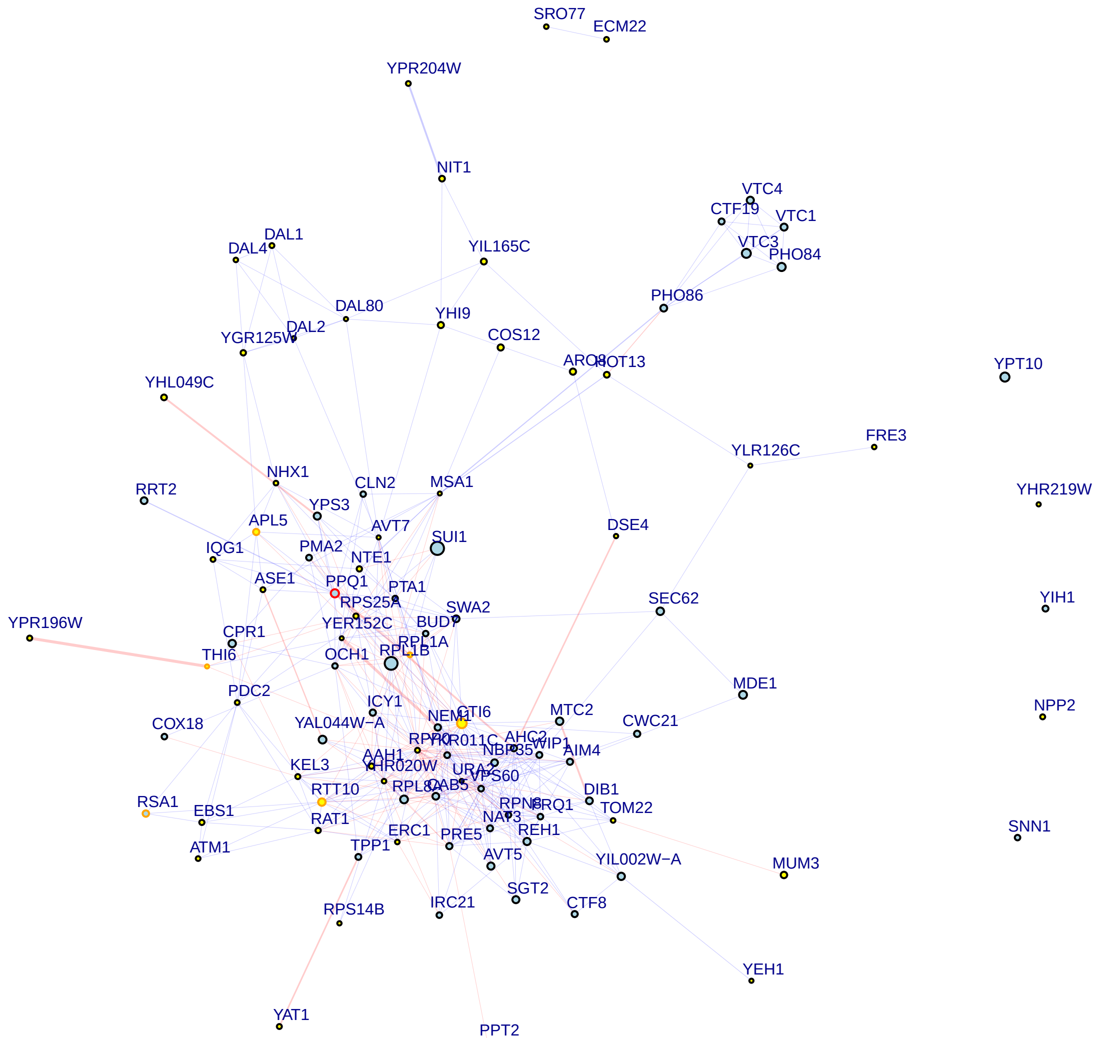

chrXVI:383928_G/A top 50 up genes

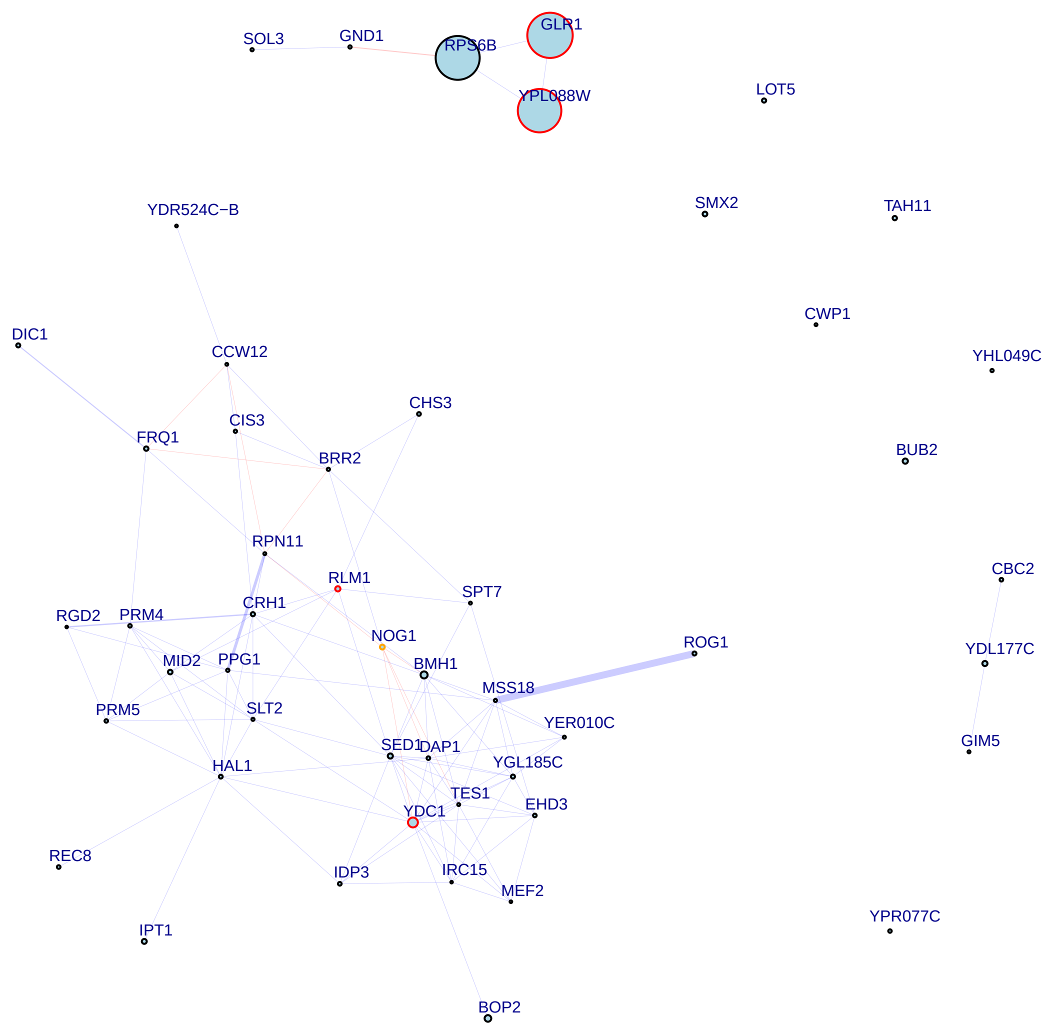

chrXVI:383928_G/A top 50 down genes

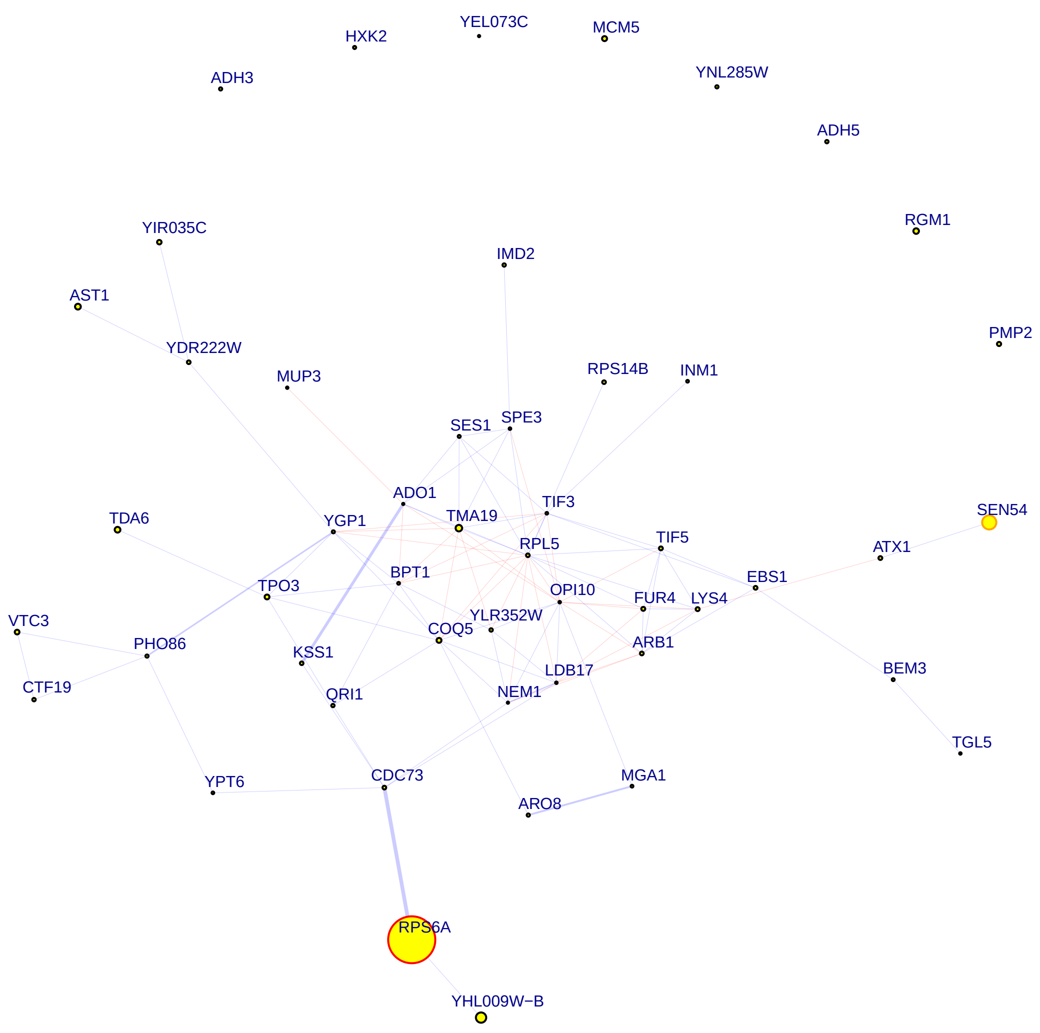

chrXVI:383928_G/A top 100 up and down genes

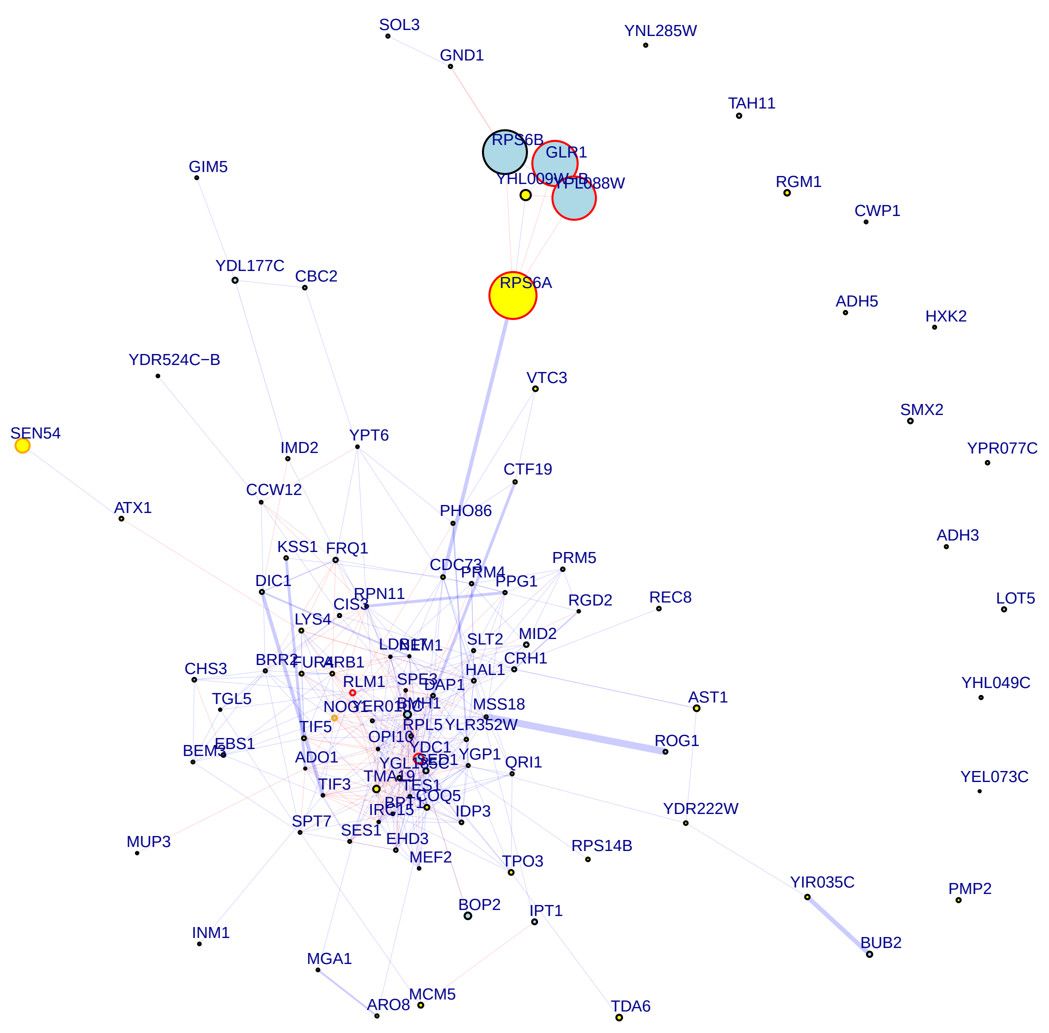

chrXVI:463543_G/A top 50 up genes

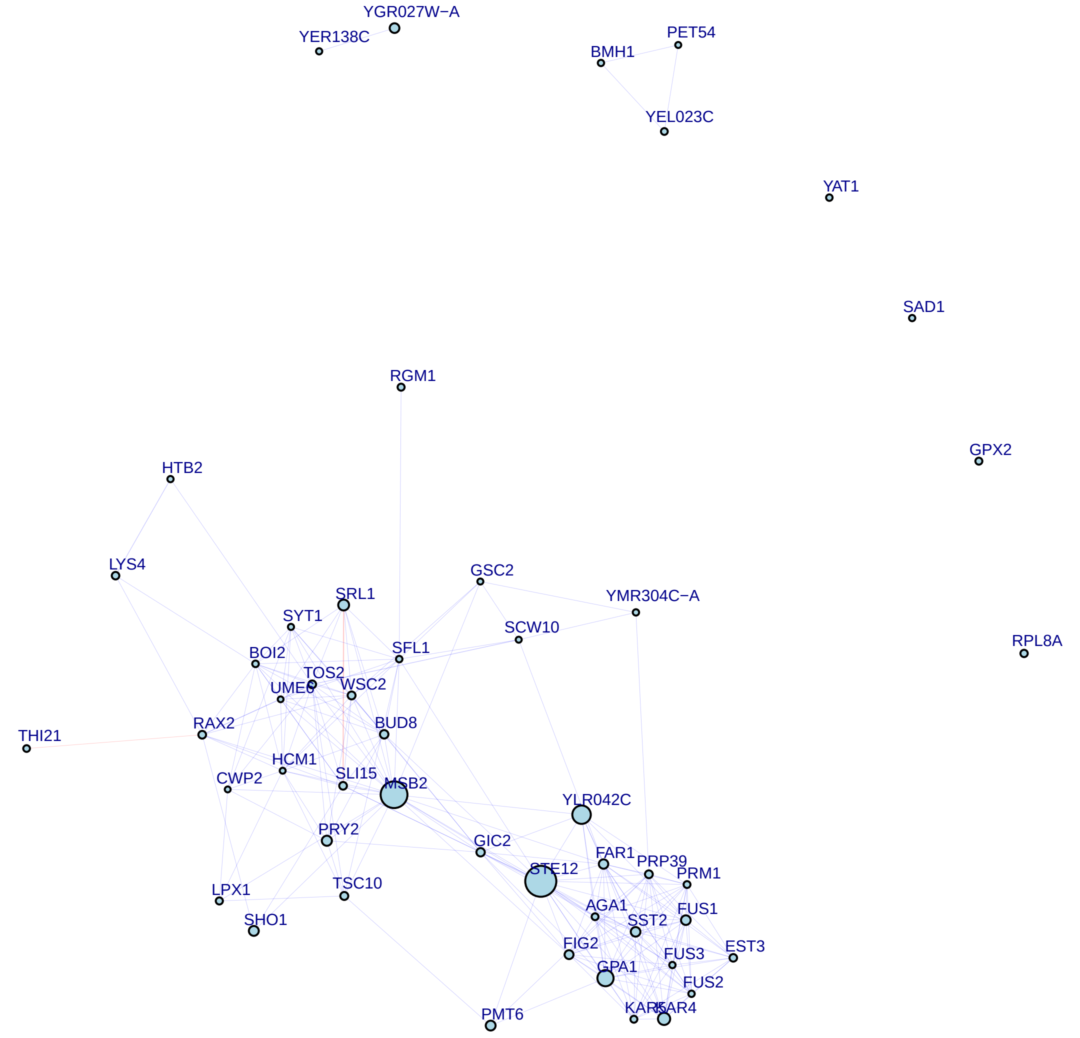

chrXVI:463543_G/A top 50 down genes

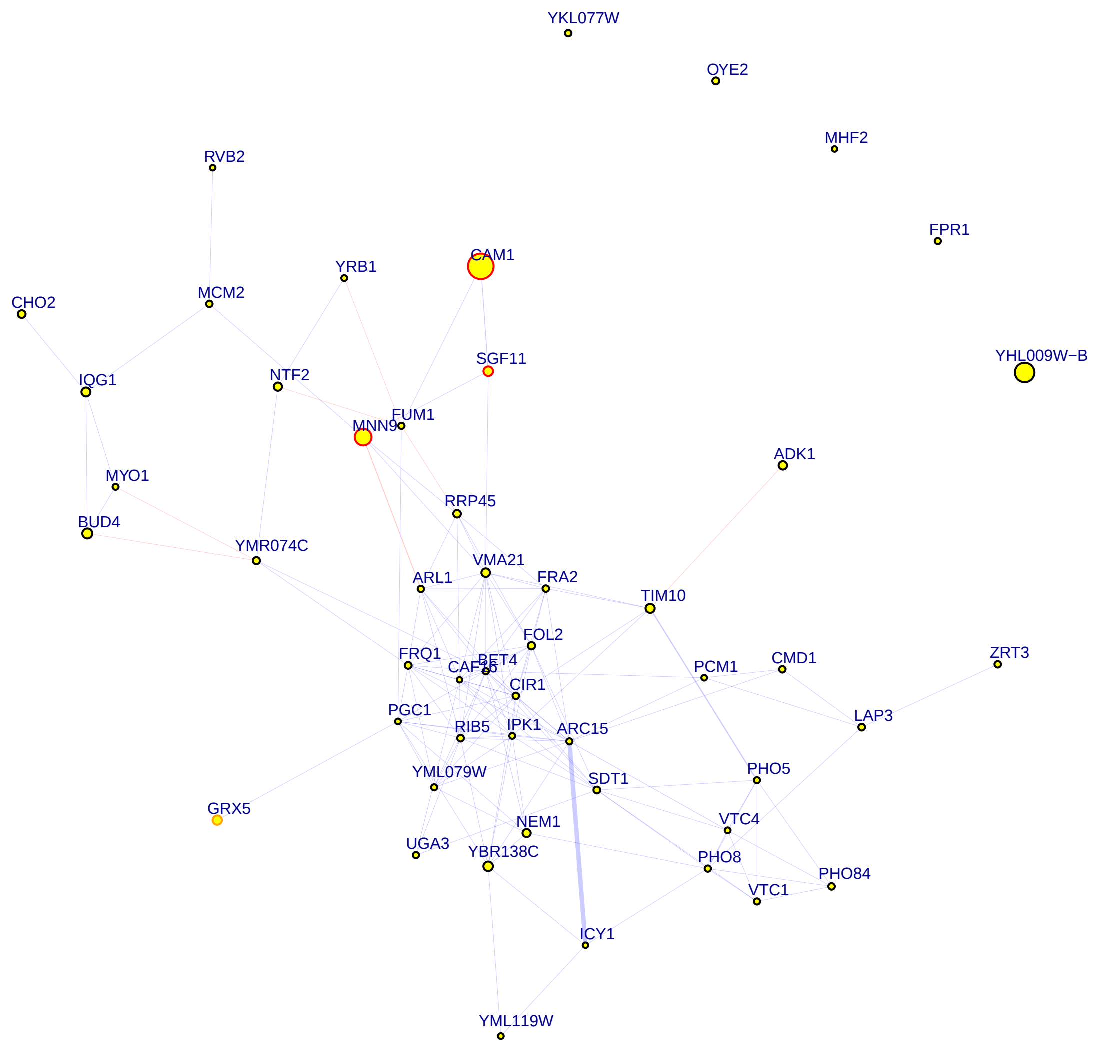

chrXVI:463543_G/A top 100 up and down genes

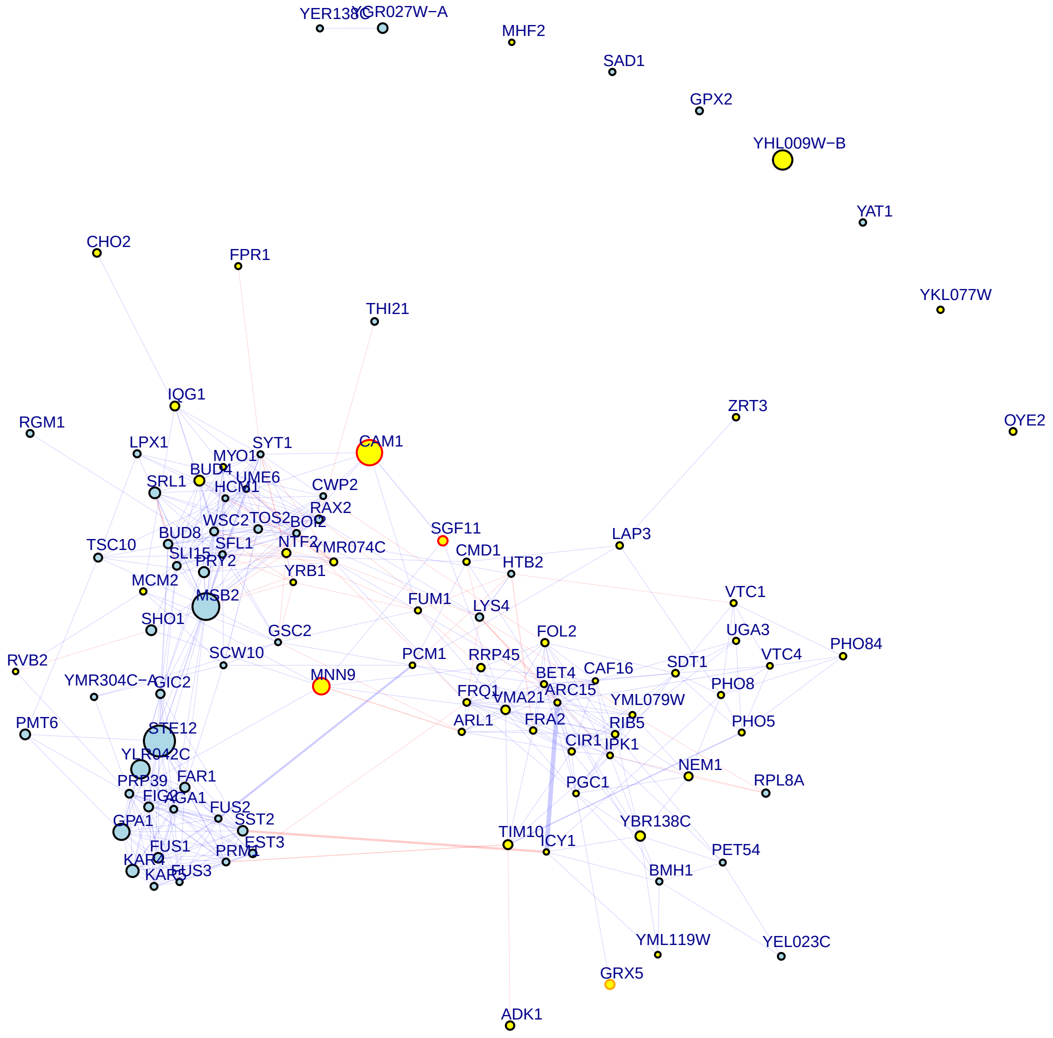

chrXVI:508199_C/T top 50 up genes

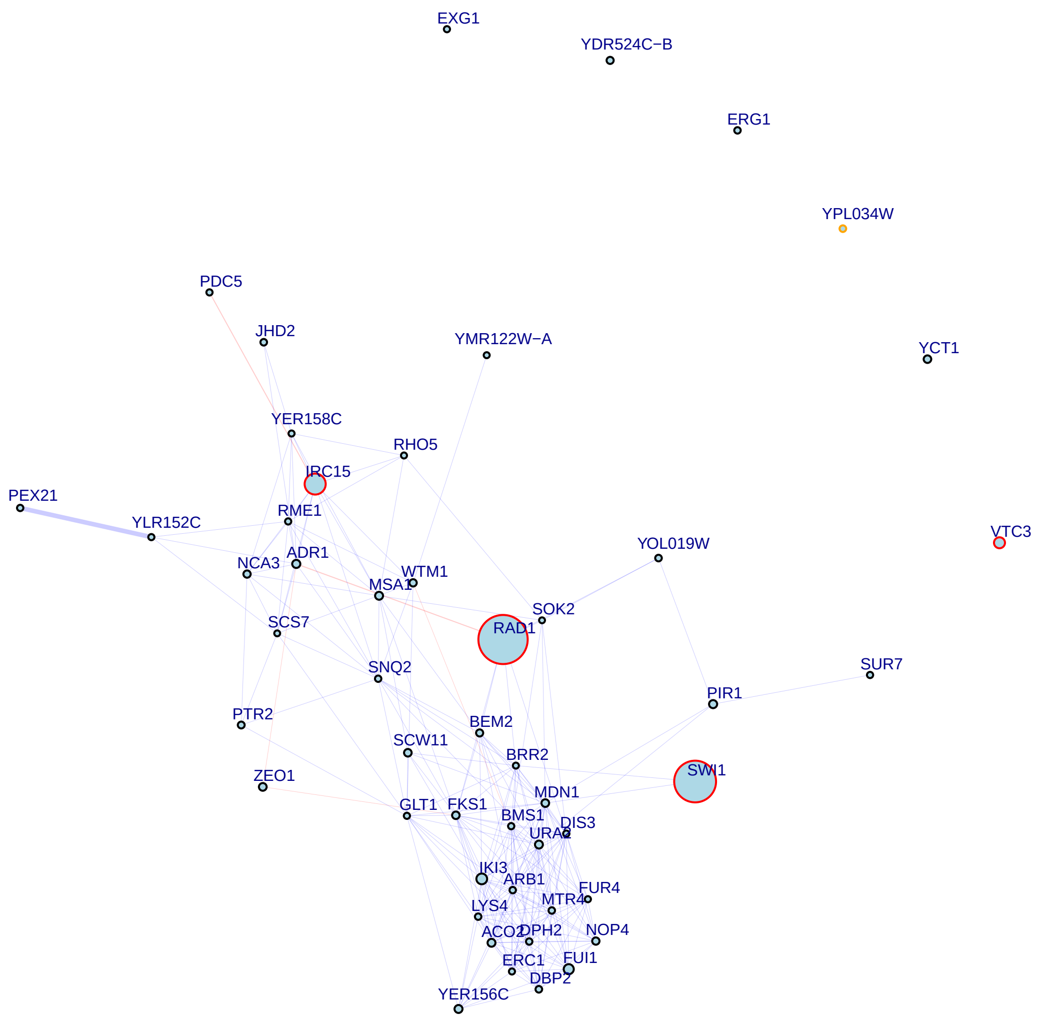

chrXVI:508199_C/T top 50 down genes

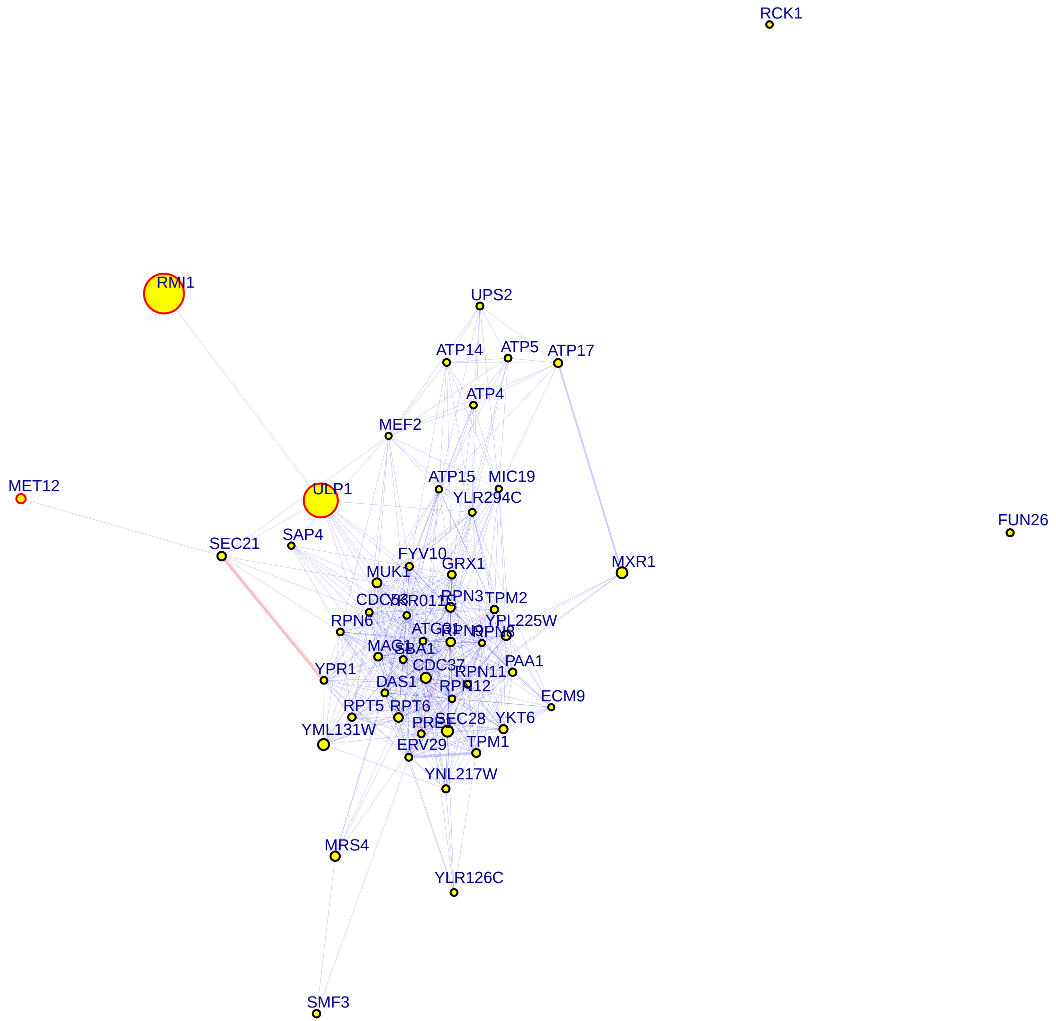

chrXVI:508199_C/T top 100 up and down genes

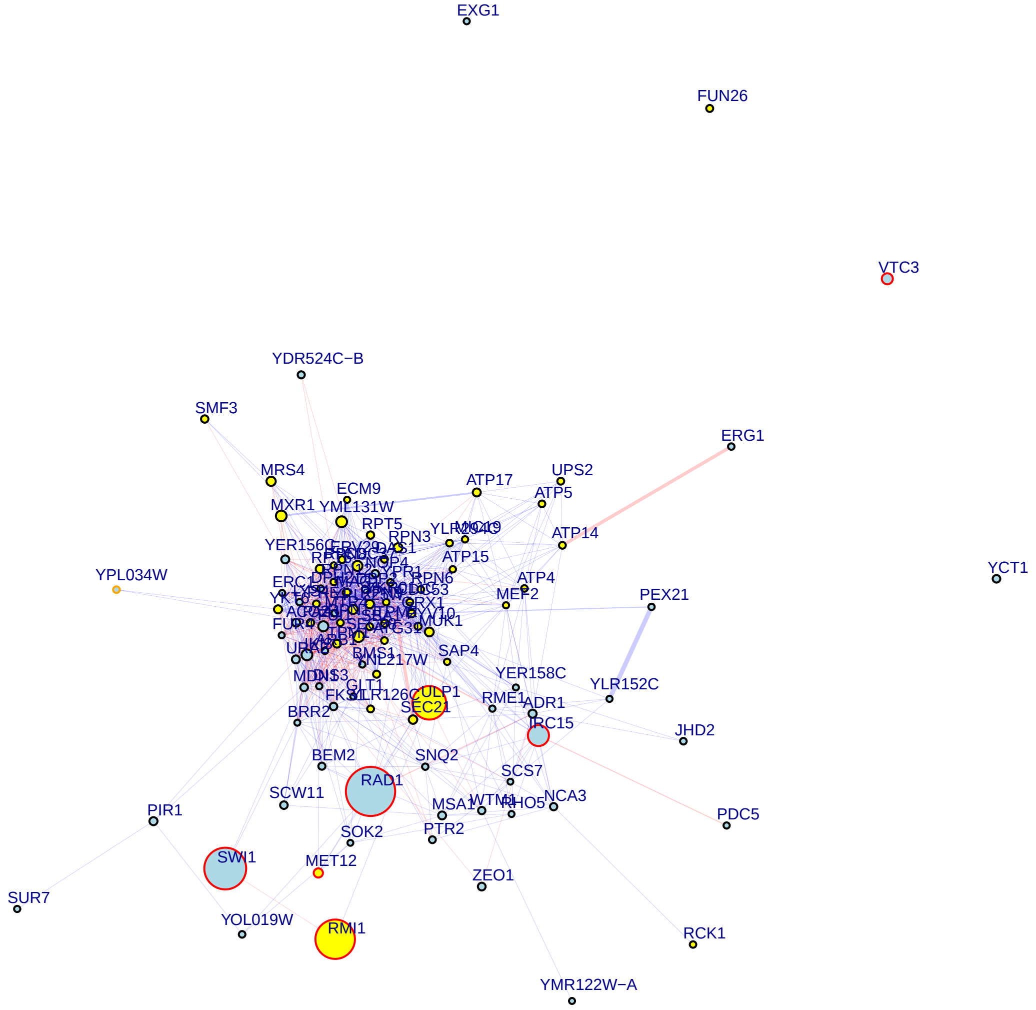

chrXVI:812990_T/C top 50 up genes

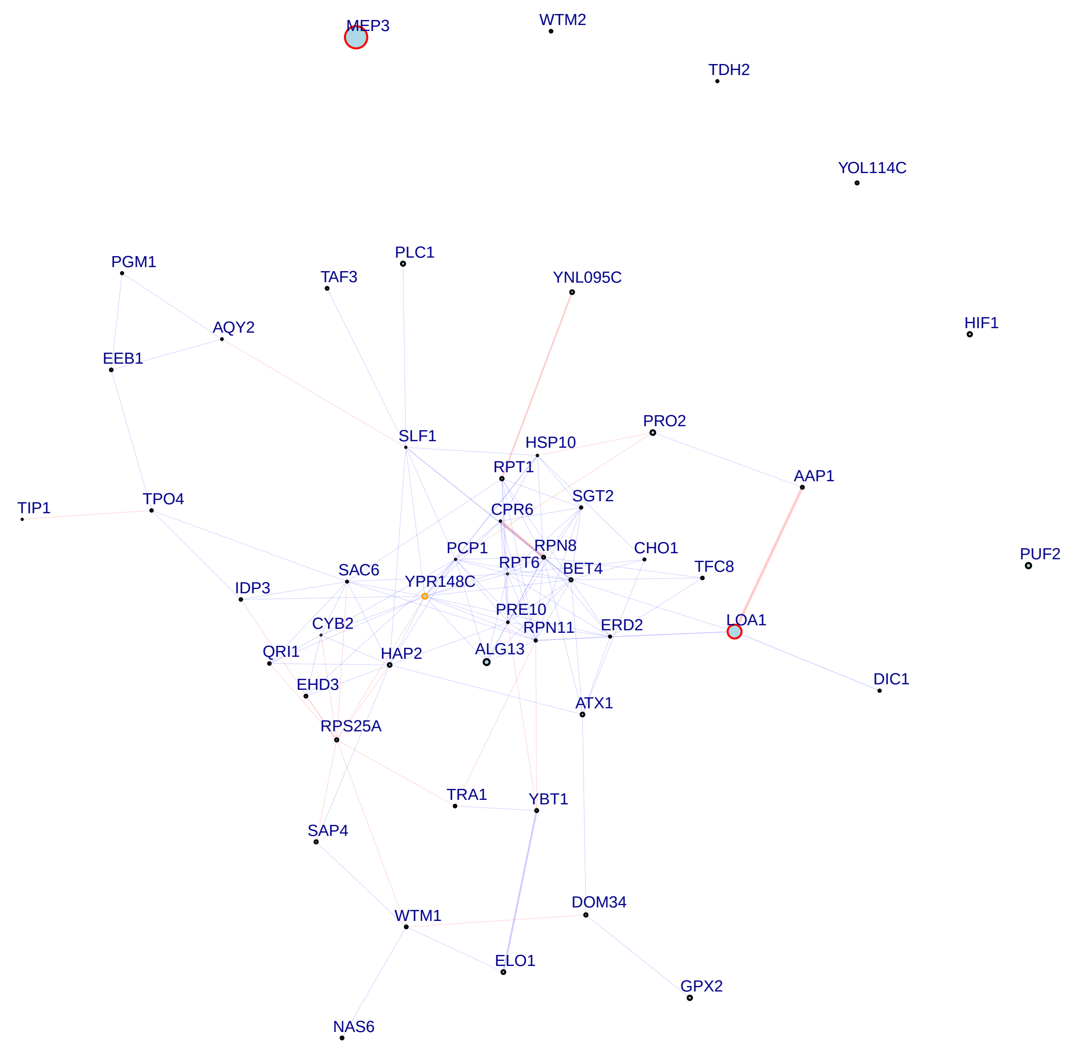

chrXVI:812990_T/C top 50 down genes

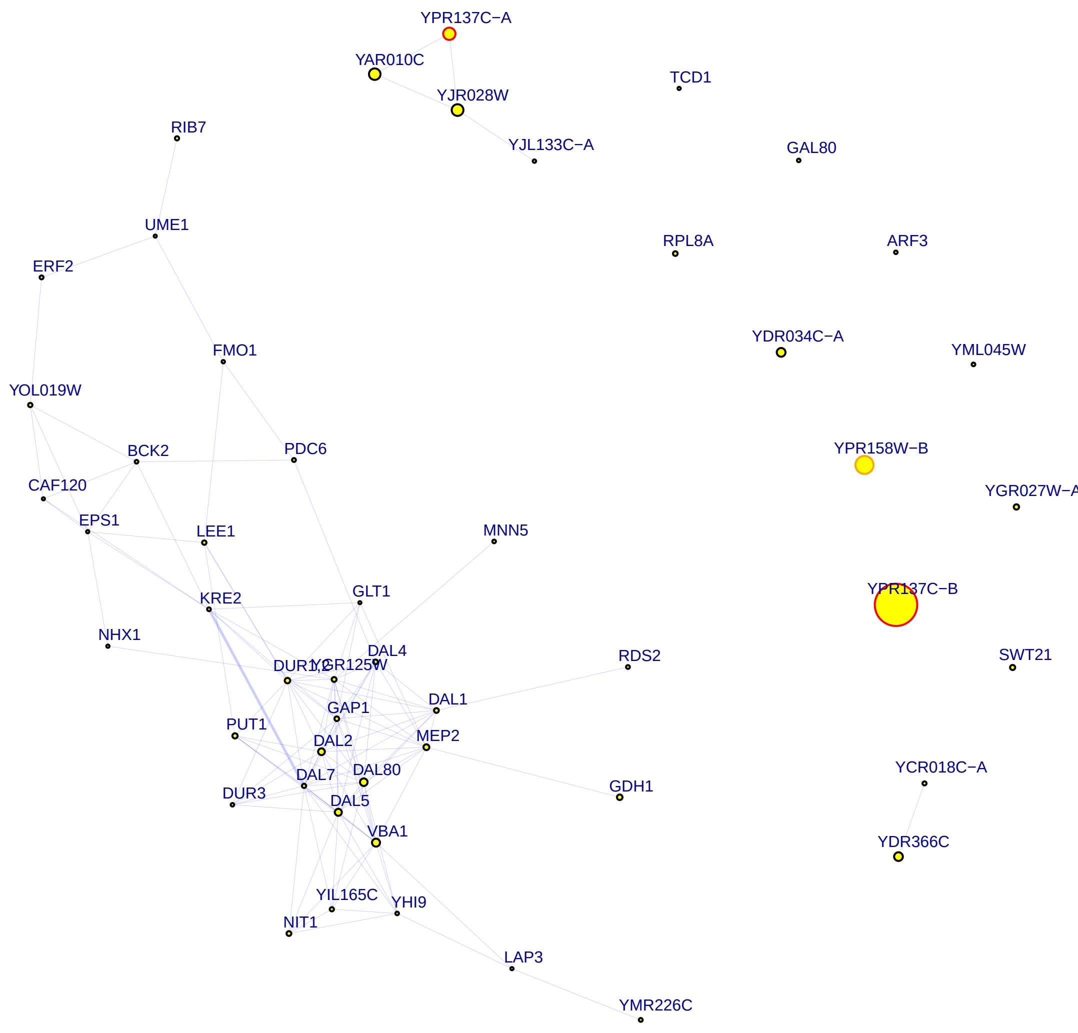

chrXVI:812990_T/C top 100 up and down genes

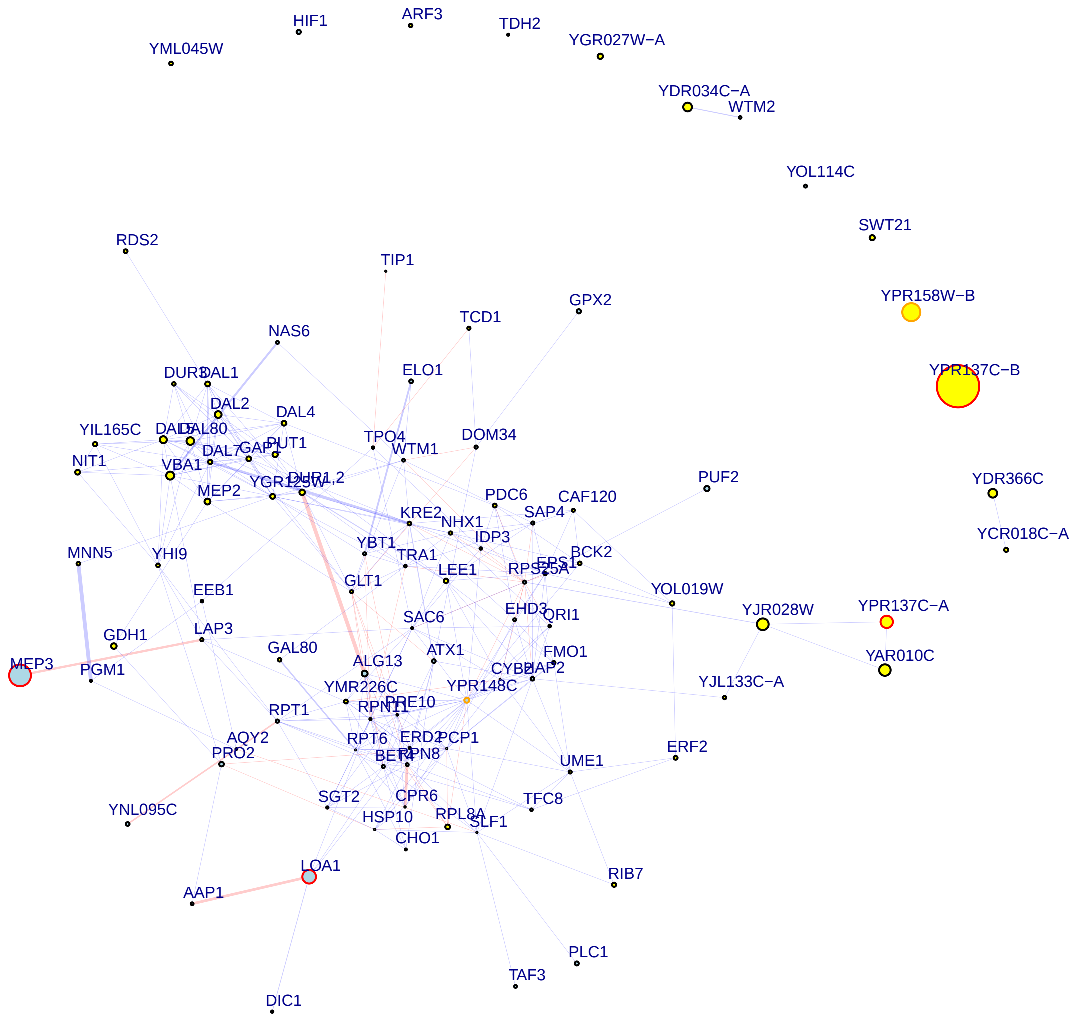

Supplement: Supplementary file 6. — This pdf file contains one page per hotspot region. Each page shows three gene co-expression networks. Each network displays connections among genes with the strongest linkages to the hotspot. The left plots show genes with higher expression in the presence of the RM allele. The middle plot shows genes with higher expression in the presence of the BY allele. The left and middle panels each show up to 50 genes with the strongest linkages to the hotspot in the given direction. If fewer than 50 genes were affected by the hotspot in the given direction, all genes affected in this direction are plotted, and their number indicated in the panel title. The right panel shows the joint network formed by the genes in the left and middle panel, with colors indicating up- or down-regulation by the hotspot. Genes with local eQTL located in the 95% confidence interval for hotspot location are indicated by red circles. Genes local eQTL located anywhere in the region tested by bootstraps are indicated by orange circles. See legend of Figure 4—figure supplement 4 for further details. [file elife-35471-supp6.pdf]
