# Supplementary material for: Genetics of trans-regulatory variation in gene expression
Source: eLife. 2018 Jul 17;7:e35471. doi: 10.7554/eLife.35471 (PMC6072440; doi:10.7554/eLife.35471)
Supplement: Supplementary file 7. — Likelihood ratios, degrees of freedom and p-values were computed using Type II analysis of variance as implemented in the R car package. (1) log2(TPM). [file elife-35471-supp7.docx]

**Table S7 – Multiple logistic regression of genes located in hotspots on various gene features.**

| Feature | Regression slope | Likelihood ratio | Degrees of freedom | p-value |
| --- | --- | --- | --- | --- |
| Expression level^1^ | 0.15 | 0.9 | 1 | 0.3 |
| Essential (yes/no) | -1.96 | 5.3 | 1 | 0.02 |
| dN/dS | -6.94 | 2.9 | 1 | 0.09 |
| Number of protein-protein interactions | 0.001 | 0.06 | 1 | 0.8 |
| Number of genetic interactions | 0.002 | 1.2 | 1 | 0.3 |
| Transcription factor (yes/no) | 2.73 | 21 | 1 | 4e-6 |
| Human homolog (yes/no) | -1.30 | 6.1 | 1 | 0.01 |
