# Supplementary material for: Genetics of trans-regulatory variation in gene expression
Source: eLife. 2018 Jul 17;7:e35471. doi: 10.7554/eLife.35471 (PMC6072440; doi:10.7554/eLife.35471)
Supplement: Supplementary file 8. — (1) Positive values indicate higher expression in RM compared to BY. [file elife-35471-supp8.docx]

**Table S8 – Strong eQTLs without pQTL**

| Gene | Chromosome | Position (bp) | eQTL LOD | eQTL effect^1^ | pQTL effect^1^ |
| --- | --- | --- | --- | --- | --- |
| *MMT1* | XII | 657,792 | 33.81 | -0.38 | -0.10 |
| *GPP2* | XII | 659,350 | 33.24 | 0.37 | 0.06 |
| *ATP5* | XII | 660,371 | 36.82 | 0.39 | 0.08 |
| *CDC10* | XII | 661,927 | 31.37 | 0.36 | 0.02 |
| *ILV6* | XIV | 376,313 | 61.21 | -0.49 | 0.19 |
| *UTP4* | XIV | 376,313 | 29.12 | 0.35 | -0.06 |
| *ARO8* | XIV | 377,751 | 39.94 | -0.41 | 0.04 |
| *TRP5* | XIV | 377,751 | 29.89 | -0.36 | -0.06 |
| *SOP4* | XIV | 393,050 | 32.33 | -0.37 | 0.00 |
| *STT3* | XIV | 449,640 | 33.23 | -0.37 | 0.06 |
| *CAM1* | XIV | 462,478 | 54.46 | -0.47 | -0.10 |
| *ADO1* | XIV | 466,588 | 31.96 | -0.37 | -0.06 |
| *ARO1* | XIV | 466,588 | 28.89 | 0.35 | -0.04 |
| *COX17* | XIV | 466,588 | 41.59 | 0.42 | 0.00 |
| *SEY1* | XIV | 466,588 | 145.00 | 0.70 | 0.04 |
| *PAT1* | XIV | 467,028 | 90.02 | 0.58 | 0.00 |
