# Supplementary material for: Genetics of trans-regulatory variation in gene expression
Source: eLife. 2018 Jul 17;7:e35471. doi: 10.7554/eLife.35471 (PMC6072440; doi:10.7554/eLife.35471)
Supplement: Supplementary file 9. — (1) Positive values indicate higher expression in RM compared to BY. [file elife-35471-supp9.docx]

**Table S9 – Strong mRNA and protein QTLs with opposite effect**

| Gene | Chromosome | Position (bp) | eQTL LOD | pQTL LOD | eQTL effect^1^ | pQTL effect^1^ |
| --- | --- | --- | --- | --- | --- | --- |
| *HEM1* | XII | 657,022 | 25.33 | 13.97 | -0.33 | 0.17 |
| *HXK2* | XII | 657,022 | 10.62 | 10.74 | 0.22 | -0.16 |
| *BDH1* | XII | 657,792 | 11.28 | 13.17 | 0.22 | -0.16 |
| *GPD1* | XII | 662,515 | 13.50 | 27.24 | 0.24 | -0.25 |
| *RPS17A* | XII | 662,515 | 5.75 | 19.3 | -0.16 | 0.22 |
| *MDH1* | XIII | 74,632 | 7.20 | 18.64 | 0.18 | -0.18 |
| *CYC1* | XIII | 338,431 | 9.43 | 28.95 | -0.20 | 0.27 |
| *NEW1* | XIV | 368,183 | 5.13 | 26.49 | 0.15 | -0.24 |
| *ILV6* | XIV | 464,117 | 8.04 | 38.18 | -0.19 | 0.31 |
| *OLE1* | XIV | 465,187 | 6.62 | 13.4 | 0.17 | -0.15 |
| *BDH1* | XIV | 466,588 | 16.14 | 22.36 | 0.27 | -0.21 |
| *RPL13B* | XIV | 466,588 | 17.14 | 29.06 | -0.27 | 0.24 |
| *RPL19A* | XIV | 466,588 | 15.48 | 36.39 | -0.26 | 0.28 |
| *RPS17A* | XIV | 466,588 | 11.10 | 18.55 | -0.22 | 0.19 |
| *RPS25A* | XIV | 466,588 | 13.54 | 13.09 | -0.24 | 0.19 |
| *SEC16* | XIV | 466,588 | 7.86 | 16.52 | 0.19 | -0.19 |
| *SLA1* | XIV | 466,588 | 18.64 | 23.48 | 0.29 | -0.21 |
| *GCV3* | XIV | 467,028 | 17.55 | 9.04 | -0.28 | 0.16 |
| *CPA2* | XIV | 469,224 | 10.41 | 70.15 | 0.22 | -0.50 |
