# Supplementary material for: Genetics of trans-regulatory variation in gene expression
Source: eLife. 2018 Jul 17;7:e35471. doi: 10.7554/eLife.35471 (PMC6072440; doi:10.7554/eLife.35471)
Supplement: Supplementary file 10. — (1) Positive values indicate higher expression in RM compared to BY. [file elife-35471-supp10.docx]

**Table S10 – Strong pQTLs without eQTL**

| Gene | Chromosome | Position (bp) | pQTL LOD | pQTL effect^1^ | eQTL effect^1^ |
| --- | --- | --- | --- | --- | --- |
| *RPS17A* | II | 137,197 | 38.83 | -0.30 | -0.01 |
| *TIP1* | V | 378,304 | 44.28 | 0.30 | 0.02 |
| *GPD1* | V | 504,305 | 123.79 | -0.54 | -0.02 |
| *TPO1* | VII | 475,295 | 51.83 | 0.35 | 0.03 |
| *TIP1* | XI | 269,056 | 39.08 | 0.30 | -0.07 |
| *SSA1* | XI | 270,756 | 95.23 | 0.50 | -0.02 |
| *LEU1* | XII | 676,798 | 123.29 | 0.57 | 0.04 |
| *CAR2* | XIII | 112,600 | 46.66 | -0.33 | -0.02 |
| *GCN1* | XIV | 457,698 | 94.83 | -0.48 | 0.03 |
| *ATP2* | XIV | 460,398 | 104.94 | 0.52 | 0.07 |
| *CDC60* | XIV | 464,098 | 45.72 | -0.31 | 0.05 |
| *GLN1* | XV | 167,400 | 43.03 | -0.31 | -0.03 |
